# Tiling microarray analysis of rice chromosome 10 to identify the transcriptome and relate its expression to chromosomal architecture

**DOI:** 10.1186/gb-2005-6-6-r52

**Published:** 2005-05-27

**Authors:** Lei Li, Xiangfeng Wang, Mian Xia, Viktor Stolc, Ning Su, Zhiyu Peng, Songgang Li, Jun Wang, Xiping Wang, Xing Wang Deng

**Affiliations:** 1 Department of Molecular, Cellular, and Developmental Biology, Yale University, New Haven, CT 06520, USA; 2 National Institute of Biological Sciences, Zhongguancun Life Science Park, Beijing 102206, China; 3 Peking-Yale Joint Research Center of Plant Molecular Genetics and Agrobiotechnology, College of Life Sciences, Peking University, Beijing 100871, China; 4 Beijing Institute of Genomics, Chinese Academy of Sciences, Beijing 101300, China; 5 National Center of Crop Design, China Bioway Biotech Group Co., LTD, Beijing 100085, China; 6 Genome Research Facility, NASA Ames Research Center, MS 239-11, Moffett Field, CA 94035, USA

## Abstract

**Background:**

Sequencing and annotation of the genome of rice (*Oryza sativa*) have generated gene models in numbers that top all other fully sequenced species, with many lacking recognizable sequence homology to known genes. Experimental evaluation of these gene models and identification of new models will facilitate rice genome annotation and the application of this knowledge to other more complex cereal genomes.

**Results:**

We report here an analysis of the chromosome 10 transcriptome of the two major rice subspecies, *japonica *and *indica*, using oligonucleotide tiling microarrays. This analysis detected expression of approximately three-quarters of the gene models without previous experimental evidence in both subspecies. Cloning and sequence analysis of the previously unsupported models suggests that the predicted gene structure of nearly half of those models needs improvement. Coupled with comparative gene model mapping, the tiling microarray analysis identified 549 new models for the *japonica *chromosome, representing an 18% increase in the annotated protein-coding capacity. Furthermore, an asymmetric distribution of genome elements along the chromosome was found that coincides with the cytological definition of the heterochromatin and euchromatin domains. The heterochromatin domain appears to associate with distinct chromosome level transcriptional activities under normal and stress conditions.

**Conclusion:**

These results demonstrated the utility of genome tiling microarray in evaluating annotated rice gene models and in identifying novel transcriptional units. The tiling microarray sanalysis further revealed a chromosome-wide transcription pattern that suggests a role for transposable element-enriched heterochromatin in shaping global transcription in response to environmental changes in rice.

## Abstract 2

A transcriptome analysis of chromosome 10 of 2 rice subspecies identifies 549 new gene models and gives experimental evidence for around 75% of the previously unsupported predicted genes.

## Background

As one of the most important crop species in the world and a model for the Gramineae family, rice (*Oryza sativa*) was selected as the first monocotyledonous plant to have its genome completely sequenced. Draft genome sequences of the two major subspecies of rice, *indica *and *japonica*, were made available in 2002 [1,2]. These were followed by the advanced sequences of *japonica *chromosomes 1, 4 and 10 [3-5]. The finish-quality whole-genome sequences of *indica *and *japonica *have recently been obtained [6-8].

Available rice sequences have been subjected to extensive annotation using *ab initio *gene prediction, comparative genomics, and a variety of other methods. These analyses revealed abundant compositional and structural features of the predicted rice genes that deviate from genes in other model organisms. For example, distinctive negative gradients of GC content, codon usage, and amino-acid usage along the direction of transcription were observed in many rice gene models [2,9]. On the other hand, many predicted rice genes that lack significant homology to genes in other organisms also exhibit characteristics such as unusual GC composition and distribution, suggesting that they might not be true genes [10,11]. Furthermore, the abundance and diversity of transposable elements (TEs) within the rice genome that possess a coding capacity pose an additional challenge to accurate annotation of the rice genome [10,12,13].

As such, our understanding of the rice genome is largely limited to the state-of-the-art gene prediction and annotation programs. This is probably best reflected by the lack of a consensus of the estimation of the total gene number in rice [6-8,10,11]. Estimated total gene number based on the draft sequences of *japonica *and *indica *ranged widely from 30,000 to 60,000 [1,2]. Finished sequences of chromosome 1, 4 and 10 allowed a more finely tuned estimate that placed the total number of rice genes between 57,000 and 62,500 [3-5]. These estimates included a large number of gene models that contain TE-related open reading frames (ORFs). Excluding the TE-related ORFs could reduce the gene number to about 45,000 [6-8]. Even then, between one third and one half of the predicted genes appear to have no recognizable homologs in the other model plant *Arabidopsis thaliana *[6-8]. Further, aggressive manual annotations of portions of the finished rice sequence have disqualified many of the low-homology gene models as TE-related or artifacts, arguing that there are no more than 40,000 nonredundant genes in rice [10].

Experimental evidence such as full-length cDNA sequences and expressed sequence tags (ESTs) is critical for evaluation and improvement of the genome annotation [14-16]. Large collections of rice full-length cDNA and ESTs are available [15,17]; however, given the large number of rice genes, current methods for collecting expressed sequences do not provide the necessary depth of coverage. For example, based on high-stringency alignments to EST sequences available at that time, only 24.7% of the 3,471 initially predicted genes of chromosome 10 were matched [5]. Conversely, other experiment-oriented approaches, such as massively parallel signature sequencing [18], are able to provide sufficient coverage of the transcriptome but by their nature are limited in their ability to define gene structures. Thus, it is important to survey the transcriptome using additional experimental means that permit detailed analyses of current gene models and the identification of new models.

Recent studies in several model organisms have demonstrated the utility of tiling microarrays in transcriptome identification [19-27]. Armed with new microarray technologies, it is now possible to prepare high-density oligonucleotide tiling microarrays to interrogate genomic sequences irrespective of their annotations. Consequently, results from these studies indicate that a significant portion of the transcriptome resides outside the predicted coding regions [19-21,24,25]. In addition, these studies show that tiling microarrays are able to improve or correct the predicted gene structures [19,23,26]. Based on considerations of feature density, versatility of modification, and compatibility with our existing conventional microarray facility, the maskless array synthesizer (MAS) platform [24,26,28,29] was chosen for our rice transcriptome analysis.

Here we report the construction and analysis of two independent sets of custom high-density oligonucleotide tiling microarrays with unique 36-mer probe sequences tiled throughout the nonrepetitive sequences of chromosome 10 for both *japonica *and *indica *rice. Hybridized with a mixed pool of cDNA targets, these tiling microarrays detected over 80% of the annotated nonredundant gene models in both *japonica *and *indica*, and identified a large number of transcriptionally active intergenic regions. These results, coupled with comparative gene model mapping and reverse transcription PCR (RT-PCR) analysis, allowed the first comprehensive identification and analysis of a rice chromosomal transcriptome. These results further revealed an association of chromosome 10 transcriptome regulation with the euchromatin-heterochromatin organization at the chromosomal level.

## Results

### Rice chromosome 10 oligonucleotide tiling microarrays

Based on recent studies using MAS oligonucleotide tiling microarrays to obtain gene expression and structure information [24,26,28,29], we designed two independent sets of 36-mer probes, with 10-nucleotide intervals, tiled throughout both strands of *japonica *and *indica *chromosome 10, respectively. After filtering out those probes that represent sequences with a high copy number or a high degree of complementarity, 750,282 and 838,816 probes were retained to interrogate the entire nonrepetitive sequences of *japonica *and *indica *chromosome 10 and were synthesized in two sets of MAS microarrays [24,26,29]. The arrays were hybridized with target cDNA prepared from equal amounts of four selected poly(A)^+ ^RNA populations (the N Arrays), namely, seedling roots, seedling shoots, panicles, and suspension cultured cells of the respective rice subspecies. In addition, a set of *japonica *arrays was hybridized to shoot poly(A)^+ ^RNA derived from seedlings with a mineral/nutrient disturbance (the S Arrays).

**Figure 1:**
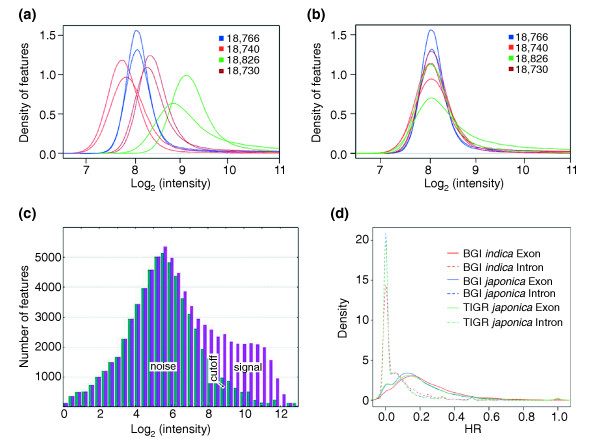
Processing the rice chromosome 10 tiling microarray hybridization data. **(a) **Distribution of fluorescence intensity of all positive and negative features of the four *indica *N Arrays. **(b) **All eight distributions were scaled to have a uniform intensity peak value at 8 (log_2_). **(c) **Mathematic model for determination of signal probes. A bimodal distribution of log_2 _background-adjusted intensity of all positive features is used to model the noise as a normal distribution by mirroring the distribution of low intensity (< 6 of log_2_). A cutoff value corresponding to a 90% confidence level to reject noise probes according to the modeled noise distribution is indicated. **(d) **Distribution of hybridization rate in the exonic and intronic regions of rice chromosome 10. Hybridization rate (HR) is calculated as the ratio of the number of signal probes against the total number of interrogating probes per kilobase of sequence.

**Figure 2:**
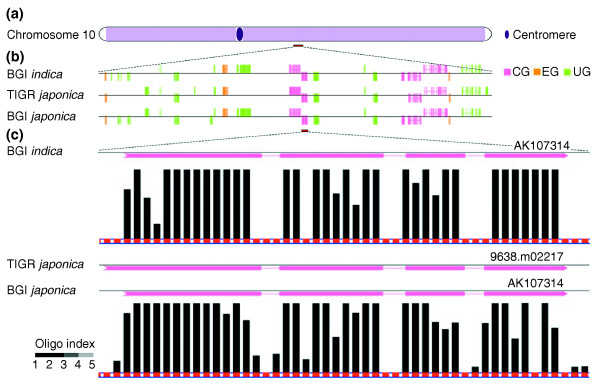
Tiling microarray analysis of the rice chromosome 10 transcriptome. **(a) **Schematic representation of rice chromosome 10. The purple oval denotes the centromere. **(b) **A region from the long arm of chromosome 10 displaying the three sets of gene models used: BGI *indica*; TIGR *japonica *and BGI *japonica*. The nonredundant protein-coding gene models are aligned to the chromosomal sequences and color-coded on the basis of their classification (see text). **(c) **Detailed tiling profile of one representative CG model. The model is represented here as block arrows, which point in the direction of transcription. Signal oligos are aligned according to their chromosomal coordinates. The fluorescence intensity value of each signal oligo, capped at 2,500, is depicted as a vertical bar. The shade of the bar represents the oligo index score (see Materials and methods). The red blocks underneath the bars indicate the presence of an interrogating oligo in the microarray.

Our MAS microarrays utilize a 'chessboard' design, meaning that each positive feature, which contains an interrogating probe, is surrounded by four negative features and vice versa [24,26]. Given that both positive and negative features contain a linker oligo to which the interrogating probes were synthesized, it was possible to determine signal probes (those that detect an RNA target) using a two-step procedure. After normalization (Figure 1a,b), positive features with fluorescence intensities lower than the mean intensity of the four surrounding negative features were masked. A characteristic bimodal intensity distribution of the remaining positive features was observed for each microarray (Figure 1c). Based on a statistical model to reject noise probes at a 90% confidence (see Materials and methods), signal probes and their normalized fluorescence intensities were determined (Figure 1c). Signal probes were correlated with the transcriptionally active regions (TARs) of the chromosome by alignment of the probes to the chromosomal coordinates (Figure 2). Experimental identification of the transcriptome was then achieved by systematically examining the expression of the annotated gene models and screening for intergenic TARs.

### Rice chromosome 10 gene models

Finished sequences have been determined for both *japonica *and *indica *chromosome 10 [5-8]. Initial annotation of *japonica *chromosome 10 produced 3,471 protein-coding gene models [5], which was updated to 3,856 in the release 2 of the Rice Pseudomolecules from The Institute for Genomic Research (TIGR) [8]. Of these, 829 (21.5%) were found to be TE-related models. Eight gene models were mapped to other chromosomes, and were not included in this study. Classification of the 3,019 nonredundant protein-coding gene models was based on alignments to the rice full-length cDNA and ESTs [15,17]. These analyses led to the identification of 935 (31.0%) cDNA-supported gene (CG) and 321 (10.6%) EST-supported gene (EG) models. The remaining 1763 (58.4%) models were classified as unsupported gene (UG) models. This model set is designated TIGR *japonica *(Table 1, Figure 2 and see Additional data file 1).

For comparison, the so-called BGI *japonica *gene models were included, whereby the *japonica *chromosome 10 sequence was independently annotated by the Beijing Genomics Institute (BGI) [6,30]. This model set, generated by the FGENESH output with limited full-length cDNA/EST input, contains 851 TE, 943 CG, 272 EG, and 1,549 UG models (Table 1, Figure 2). To analyze the *indica *chromosome 10 transcriptome, and for comparative analysis, the BGI *indica *models were also examined [2,6,30]. Classification of the *indica *models identified 574 TE, 821 CG, 328 EG, and 1,660 UG models (Table 1, Figure 2 and see Additional data file 2).

### Tiling microarray detection of rice chromosome 10 gene models

Analysis of the N arrays detected 2,428 out of 2,809 BGI *indica *(86.4%), 2,319 out of 2,764 BGI *japonica *(83.9%), and 2,472 out of 3,019 TIGR *japonica *(81.9%) nonredundant gene models (Table 1). Although no technical replication was performed, several observations indicate that tiling microarray analysis provides a reliable evaluation of the expression of the gene models. First, consistent with their classification, gene models with previous experimental support (CG and EG) showed a higher detection rate than the unsupported models (Table 1). For example, 93.2% and 90.7% of the TIGR *japonica *CG and EG models were detected, respectively, whereas only 74.3% of the UG models were (Table 1). Second, supported models (CG and EG) exhibited very similar array detection rates across the three sets of gene models. Because the same cDNA and ESTs were used to classify the three sets of gene models, this result implies a strong correlation between tiling microarray detection and expressed sequences. In supporting of this conclusion, TIGR *japonica *models with at least one match with rice EST sequences exhibited a 92.7% (1,010 of 1,089) detection rate whereas only 75.7% (1,458 of 1,925) models without a matching EST were detected. Third, examination of signal probe distribution, measured by hybridization rate (HR, see Materials and methods), in the annotated exonic and intronic regions indicates that the tiling microarrays detected transcription predominantly locate in the exons. Across the three annotations, the HRs of both the intronic regions (dashed lines) and exonic regions (solid lines) showed bimodal distributions, with their respective major peaks well separated (Figure 1d). The minor intronic HR peak likely reflects transcriptional activities of exons misidentified as introns or in uncharacterized splice variants. Conversely, the minor exonic HR peak is likely to be due to misinterpretation of introns as exons, or exons or genes not expressed at all in the RNA populations used (Figure 1d).

### Analysis of previously unsupported gene models

The relatively poor detection rate for the unsupported models suggests that their expression may be more restricted to specific cell types or developmental stages, thus eluding tiling array detection. Alternatively, some of these UG models might be false and do not represent real genes. For further analysis, gene models were classified as high homology (HH) and low homology (LH) models based on comparison using an expect value of e^-7 ^for predicted protein homology between rice and *Arabidopsis *[6]. It should be noted that the simple sequence alignment is likely to fail to detect some structural homology. However, this simple division is useful for separating two groups of gene models for expression comparison. For example, in the BGI *japonica *annotation, there are 589 UG/HH and 960 UG/LH models. By comparison, our tiling microarray detected 495 (84.0%) UG/HH models, but only 707 (73.7%) UG/LH models. Because the UG/LH models lack any previous supporting evidence (either homology or expression), concerns have been raised as to whether they represent real genes [10,11]; therefore, the expression properties of the UG/LH models are of particular interest for further evaluation.

To investigate the possibility that expression of some UG/LH models is restricted to special conditions, we analyzed the S Arrays with regard to UG model expression. Of the gene models in the BGI *japonica *annotation, 63.4% were detected in seedling shoots under a variety of stress conditions that are known to significantly alter gene expression profiles [31,32]. These included 39 (2 CG/HH, 2 EG/HH, 8 UG/HH, 2 CG/LH, 2 EG/LH and 23 UG/LH) models that eluded detection by the N Arrays. The enrichment of UG/LH models in S Arrays-specific models indicates that some UG/LH models indeed have specialized expression. Though it is entirely possible that additional UG/LH models could be detected under other stress conditions, the small number of UG/LH models specifically detected from the S Arrays (23 of 960, or 2.4%) suggests that specialized expression of UG/LH models alone may not account for the overall low detection rate of the UG/LH models.

**Figure 3:**
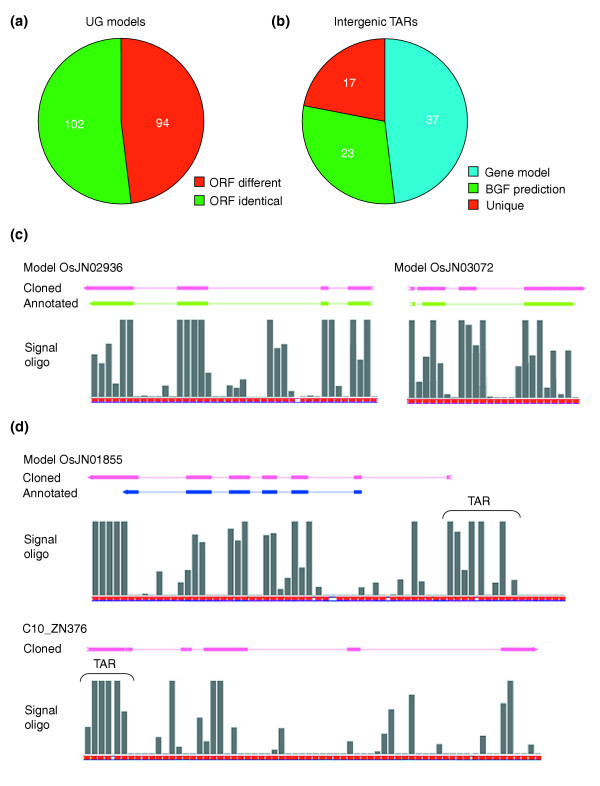
Cloning and sequence analysis of *japonica *chromosome 10 UG models and intergenic TARs. **(a) **Summary of RT-PCR analysis of selected UG models. ORF identical, annotated ORF is the same as determined from the cloned sequence; ORF different, annotated ORF is different from that in the cloned sequence. **(b) **Summary of RT-PCR analysis of selected intergenic TARs. Gene model, cloned TARs overlapping with TIGR models; BGF prediction, cloned TARs overlapping with BGF predictions; unique, cloned TARs not overlapping with any annotated feature. **(c) **Representative UG models whose cloned sequences either differ from (OsJN02936) or are the same as (OsJN03072) the annotated ones. **(d) **Representative intergenic TARs whose cloned sequences either overlap with a TIGR model (OsJN01855) or are completely intergenic (C10_ZN376). Representation of microarray data in this figure is the same as in Figure 2 except that the oligo index is omitted.

In a separate approach to verify UG model annotation, 589 UG models were randomly selected for a high throughput RT-PCR analysis. Overall, 196 (33.3%) of the selected UG models were cloned and sequence-confirmed from the same RNA samples used for the N Arrays (Figure 3a and Additional data file 3). Given that only 62% (49/79) of CG models were successfully cloned and sequence-confirmed in a control experiment, these results suggest that expression of approximately half (33% over 62%) of the UG models can be confirmed in our experimental conditions. Closer inspection of the confirmed UG transcripts showed that only 102 (52%) contain an identical ORF as predicted, whilst 94 (48%) exhibit different ORFs compared to the predictions (Figure 3a,c), suggesting that the gene structure of about half of the UG models need to be corrected or improved. Since the tiling microarrays used in this study have limited ability to pinpoint precise intron-exon junctions, transcript cloning and sequence analysis are still required to verify the annotated gene structures.

### Identification and analysis of intergenic TARs

**Figure 4:**
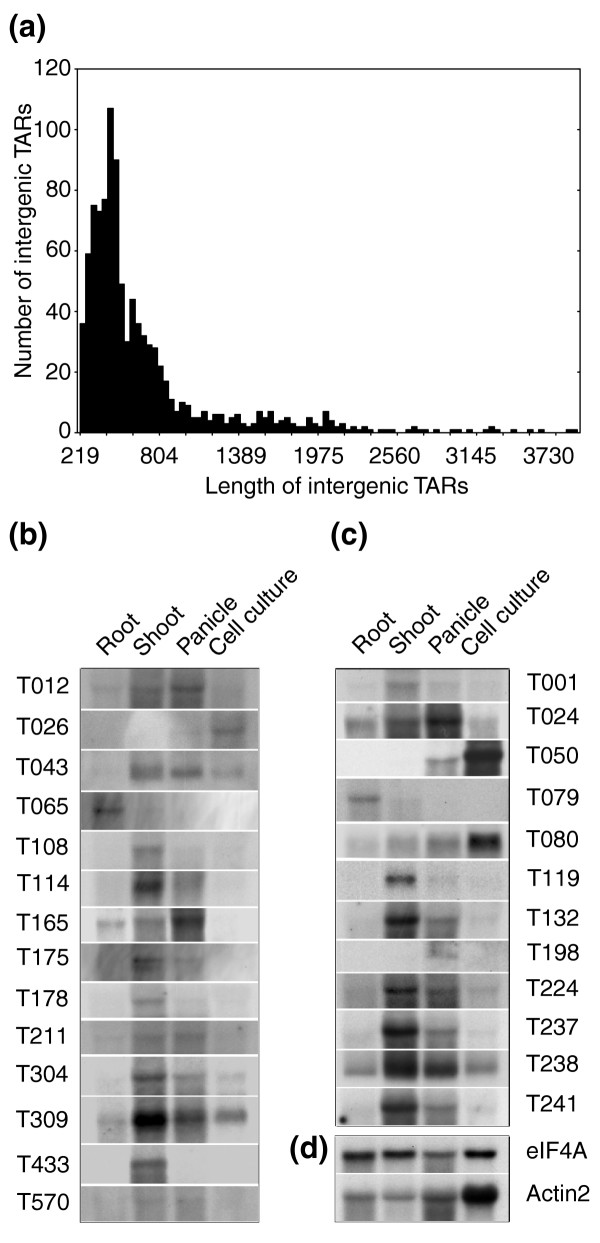
Analysis of intergenic TARs of *japonica *chromosome 10. **(a) **The 988 *japonica *chromosome 10 intergenic TARs distributed by length. **(b) **RNA gel blotting analysis of selected *japonica *intergenic TARs. Probes for the intergenic TARs shown in this panel were derived from corresponding PCR-amplified TAR sequences from *japonica *rice genomic DNA. **(c) **Probes shown in this panel were derived from RT-PCR amplification of the corresponding TARs from poly(A)^+ ^RNA. **(d) **The rice cDNAs for *eIF4A *and *actin2 *were used as loading controls. 5 μg of RNA from the four sources - root, shoot, panicle, and suspension cell culture - that were used for probing tiling microarrays were used for RNA blot analysis here.

We found that 10.26% and 11.75% of the probes in the *japonica *and *indica *N Arrays were considered signal probes, respectively (Figure 1c). Approximately 55% and 15% of these signal probes were found to locate in the intergenic and intronic regions, respectively, of the TIGR *japonica*, BGI *japonica*, and BGI *indica *annotations. These results indicate that, irrespective of different annotations, significant transcriptional activities locate in the annotated intergenic regions. A sliding-window-based approach was used to systematically identify intergenic TARs (see Materials and methods). Through this analysis, 574 and 522 intergenic TARs in *indica *and *japonica *were identified from the N Arrays, respectively. In addition, 466 unique intergenic TARs were identified from the S Arrays, bringing the total number of *japonica *intergenic TARs to 988. These TARs have a cumulative length of approximately 700 Kb or 3% of the chromosome. The average length of the intergenic TARs was about 700 bp (Figure 4a and Additional data file 4).

Several lines of evidence support the idea that the majority of intergenic TARs represent legitimate elements of the rice transcriptome. Sequence analysis revealed that 301 (55.0%) *indica *and 455 (46.0%) *japonica *intergenic TARs possess a significant coding capacity (more than 50 amino acids). Selected intergenic TARs were used as probes in RNA gel-blot analysis to confirm expression of these TARs. Overall, 26 out of 34 probes detected a discrete band, with tissue specificity, whereas the rest failed to detect any, suggesting that the majority of the intergenic TARs correspond to *in vivo *transcripts rather than being caused by cross hybridization (Figure 4b-d). A total of 280 intergenic TARs were selected for further analysis using an RT-PCR strategy designed to clone transcripts containing an intergenic TAR and its entire downstream (3') sequence (see Materials and methods and Additional data file 5). Of the 77 cloned transcripts whose sequences could be unambiguously confirmed, 37 overlap with existing gene models (Figure 3b,d), suggesting they are uncharacterized portions, such as 5' or 3' untranslated regions (UTRs), or splice variants of the neighboring gene models. The rest of the confirmed transcripts (40 out of 77) were located entirely in intergenic regions, suggesting that they likely represent independent novel transcriptional units (Figure 3b,d).

To further characterize the 988 *japonica *intergenic TARs, they were aligned to the output of the rice gene finder BGF [2,6,30] using the *japonica *chromosome 10 sequence, and 72 novel gene models were identified (Additional data file 1). Comparison with the cloned intergenic TARs showed that 23 of the 40 cloned novel transcripts (57.5%) were also predicted in the novel BGF models (Figure 3b), indicating that the BGF program was able to detect half of the potential novel genes represented by the intergenic TARs. However, the incomplete nature of the 17 unaccounted transcripts (Figure 3b) made it difficult to unambiguously determine whether they encode proteins.

### Tiling microarray-based gene model comparison and integration

**Figure 5:**
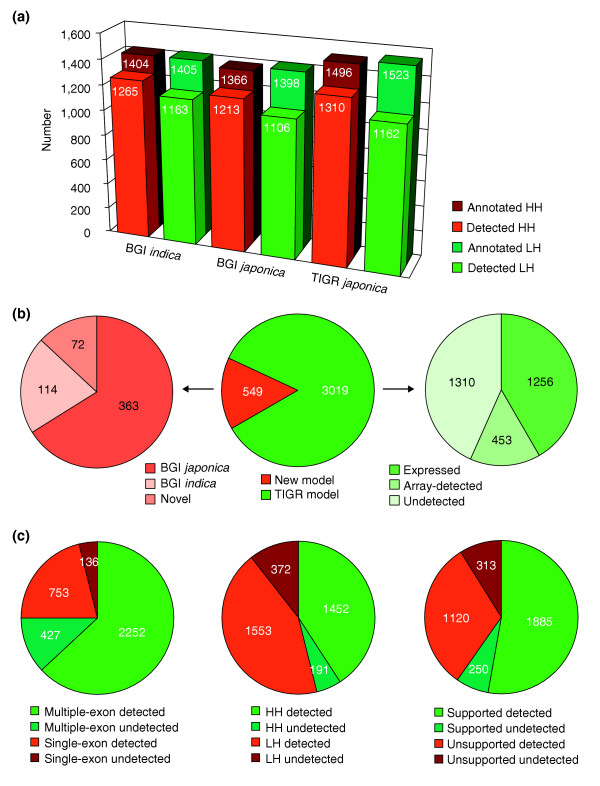
Comparison and integration of chromosome 10 gene models. **(a) **Number of annotated and array-detected high homology (HH) and low homology (LH) models in the BGI *indica*, BGI *japonica*, and TIGR *japonica *annotations. **(b) **The 549 new gene models were combined with the 3,019 TIGR models. Origins of the new models are shown on the left. Expression support for the TIGR models is shown on the right. Expressed, models matching full-length cDNA/EST; array-detected, models not supported by the expressed sequences but detected by microarray; undetected, models neither supported by expressed sequences nor detected by microarray. **(c) **Classification of integrated *japonica *chromosome 10 gene models based on tiling array detection and exon number (left), homology to *Arabidopsis *genes (middle), and previous expression or homology support to the models (right).

The TIGR model set contained 200-250 more gene models than the BGI sets (Table 1). These extra models were evenly distributed into HH and LH models (Figure 5a). The TIGR/HH models showed a similar array-detection rate, while the TIGR/LH models were detected at a lower rate (but of a similar number) in comparison with the two BGI sets (Figure 5a). This result suggests that the extra TIGR/LH models may be of low confidence and need to be further examined. Comparison of the BGI and TIGR *japonica *models indicates that there were 2323 (84.0%) and 2488 (82.4%) common to each annotation, respectively, based on ORF sequence overlaps (Additional data file 6). Meanwhile, 441 (16.1%) BGI models and 531 (17.6%) TIGR models were regarded as unique to each annotation (Additional data file 6). Naturally, the common models are more reliable, and were consequently enriched with expression- or homology-supported models. For example, only 64.5% of the unique TIGR models were detected by tiling microarrays. However, expression of 363 of the unique BGI models was confirmed by tiling array and/or cDNA and EST alignment, indicating that they are part of the *japonica *chromosome 10 transcriptome (Figure 5b).

**Figure 6:**
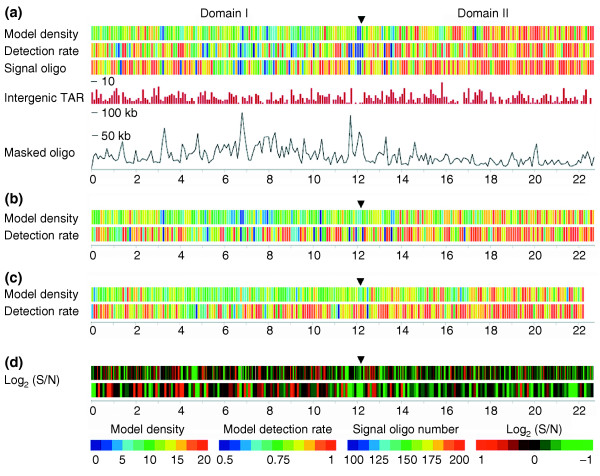
Rice chromosome 10 gene model distribution and expression. **(a) **Characterization of TIGR nonredundant protein-coding gene models. Model density, array detection rate, number of signal oligos, number of intergenic TARs, and cumulative length (in kilobases) of masked oligos are calculated in 100-kb windows along the length of chromosome 10, and are represented by color-coded vertical bars. A scale representing the physical length of chromosome 10 is shown at the bottom of the panel. The arrowhead delimits the division of domain I and domain II as indicated in the text. Note that the centromere is located at a position around 7 to 8 Mb in chromosome 10. **(b) **Gene model density and array detection rate of the BGI *japonica *annotation. **(c) **Gene model density and array detection rate of the BGI *indica *annotation. **(d) **Comparison of the S Arrays and the N Arrays using the BGI *japonica *annotation. Log_2 _(S/N) of the hybridization intensity was calculated for individual models (top) and the mean intensity of all models in 100-kb windows along the length of chromosome 10 (bottom).

The *indica *gene models were more evenly distributed along the chromosome, and the number and distribution of array-detected models was similar to that of *japonica *(Figure 6a-c). Exceptions were noted in certain regions, such as at approximately10 Mb, where *indica *models showed increased array detection rates. Such a disparity is likely to be caused by the skewed distance between corresponding *japonica*/*indica *model pairs (see below). Comparative gene model mapping indicates that 97.6% of the *japonica *chromosome10 CG/HH models had their counterparts in *indica*, while 98.3% of the *indica *CG/HH models were mapped to *japonica *(Additional data file 6 and data not shown). As the full-length cDNAs were derived from *japonica *[15], this result suggests that roughly 2% of either genome sequence was erroneous or incomplete, thereby disrupting the integrity of the affected genes such that they could not be recognized. However, only 85.3% and 88.1% of *japonica *and *indica *UG/LH models could be mapped to their reciprocal genomes. These results indicate that the unmapped UG models between *japonica *and *indica *were common but not recognized in the reciprocal genomes, or subspecies specific, or false predictions. Thus, identification of the first group of models would facilitate a better recognition of the transcriptome of both genomes. Indeed, 2,640 *indica *models were mapped to *japonica *chromosome 10 (Additional data file 7). Among those mapped *indica *models, 114 were detected by tiling array, with corresponding genome sequences that were more than 95% identical to that of *japonica *chromosome 10, but were not annotated in *japonica*. These results suggest that the counterparts of these 114 *indica *models may exist in the *japonica *chromosome 10 transcriptome (Figure 5b).

To provide a comprehensive representation of the *japonica *chromosome 10 transcriptome, the 549 new models, including 363 BGI *japonica *models, 114 BGI *indica *models, and 72 novel BGF models (see above), were integrated with the TIGR *japonica *gene models (Figure 5b). The resulting 3,568 nonredundant protein-coding gene models, including the 3,019 TIGR models, represent an 18% increase in the annotated coding capacity of *japonica *chromosome 10 (Figure 5b). The integrated models included 3005 (84.2%) that were detected by tiling arrays, of which, 1,120 (31.4%) were not previously supported by expression data or homology. Thus, 3,255 (91.2%) models in the integrated set now have at least one piece of supporting evidence (for example, expressed sequences, homology, or tiling microarray) (Figure 5c). Classification of the array-detected and undetected models, based on exon number, homology to *Arabidopsis *genes, and previous supporting evidence, indicates that detection by our tiling microarray was not biased regarding gene structure and was in general agreement with all other annotation information (Figure 5c). These results demonstrate tiling microarray analysis as a useful platform to validate and incorporate information from multiple sources to fully identify the rice transcriptome.

### Heterochromatin-associated regulation of chromosome-wide transcriptional activity

**Figure 7:**
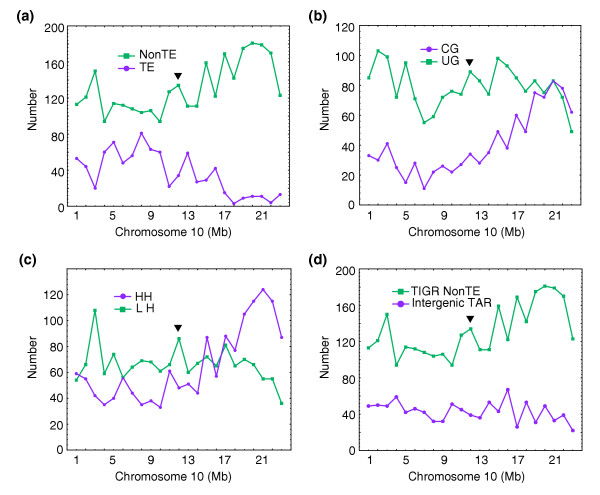
Chromosome-wide distribution of gene models and chromosomal elements. **(a) **Distribution of TIGR *japonica *nonredundant protein-coding gene models (non-TE) and transposable element-related models (TE) in 1-Mb windows across chromosome 10. The division between domain I and II is indicated by the arrowhead. Note that the centromere is located at around 7 to 8 Mb in chromosome 10. **(b) **Distribution of BGI *japonica *CG and UG models in 1-Mb windows across chromosome 10. **(c) **Distribution of BGI *japonica *HH and LH models in 1-Mb windows across chromosome 10. **(d) **Numbers of the TIGR *japonica *nonredundant protein-coding gene models (TIGR Non-TE) and tiling array-detected intergenic TARs in 1-Mb windows across chromosome 10.

We applied the tiling microarrays to study chromosomal position effects on gene expression. As shown in Figure 6, chromosome-wide gene model distribution and expression suggests that chromosome 10 can be divided into two roughly equal-sized domains, with domain I consisting of the short arm and the proximal end of the long arm, while domain II encompasses the rest of the chromosome. This division was based on transcriptional profiles of the two domains, as revealed by tiling microarray analysis (Figure 6). Domain II had a higher density of nonredundant gene models (Figure 7a). Under normal growth conditions (the N Arrays), it also contained more signal oligos and more array-detected models and thus was more transcriptionally active relative to domain I (Figure 6). Such a distinction between the two domains was further supported by the higher number of CG models in domain II, which are presumably highly expressed (Figure 7b). Interestingly, although only a small number of gene models were specifically detected from the S Arrays (see above), overall transcriptional activity in domain I was elevated under the examined stress conditions (Figure 6d). The activation was observed both at the individual gene model level and in 100 kb windows across domain I (Figure 6d). Such a general derepression of transcription under stress conditions may imply another layer of gene regulation at the chromosomal level in rice.

**Figure 8:**
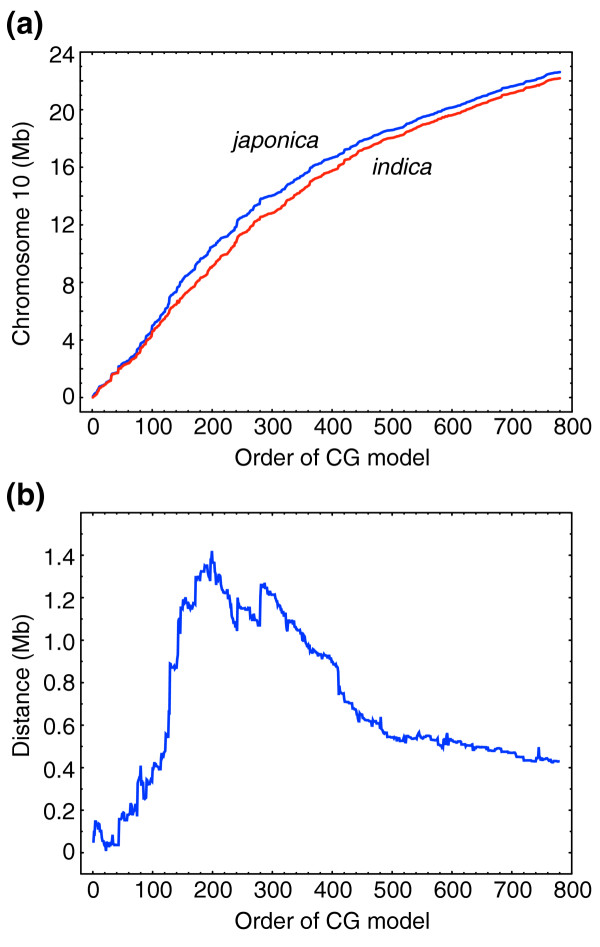
Colinearity of the CG models for chromosome 10 in *japonica *and *indica *rice. **(a) **Chromosomal positions of corresponding CG model pairs along chromosome 10 in *japonica *(blue) and *indica *(red) rice are plotted against the sequential orders of the CG pairs. **(b) **Physical distance between corresponding CG pairs is plotted against their sequential orders along the chromosome.

The observed transcriptional profiles of the two domains were associated with several architectural features of the chromosome. In general, domain I was more enriched with TE and LH models (Figure 7a,c). Domain I also harbored more repetitive sequence, as was evident from the greater number of oligos masked during array design (Figure 6a). To further examine the two domains, colinearity of the CG models in chromosome 10 of *japonica *and *indica *rice was calculated. Mapping chromosomal positions of corresponding orthologous CG model pairs along chromosome 10 of *japonica *(blue) and *indica *(red) against the sequential orders of the CG pairs resulted in two apparently smooth parallel curves (Figure 8a). This observation indicates that the order of CG models is well preserved between chromosome 10 of *japonica *and *indica *rice. However, calculation of the physical distance between corresponding *japonica *and *indica *CG models along the chromosome indicated that the positions of the CG models were more skewed in domain I, with many CG models shuffled more than 1 Mb away from their orthologous counterparts in the reciprocal chromosome (Figure 8b).

These results coincide with cytological data showing that domain I is primarily heterochromatin, whereas domain II is primarily euchromatin [5,33]. Although it remains to be seen whether the phenomena mentioned above are general features associated with the division of heterochromatin and euchromatin in rice, these results collectively indicate that the heterochromatic domain of chromosome 10 is more evolutionarily active and compositionally dynamic. Our results further indicate that the genomic characteristics of the heterochromatin domain are associated with its transcriptional activities (Figure 6).

## Discussion

Sequencing of the rice genome provides a cornerstone to understand the biology of this agriculturally important crop [1-8,34-36]. A first step in fully realizing the potential of available genome sequence is to understand its coding information and expression; however, current annotated gene models and other functional elements of a genome by and large represent hypotheses that must be experimentally tested and validated. Importantly, approximately 20,000 predicted rice genes exhibit no recognizable sequence homology to genes in other organisms, especially *Arabidopsis*, the first model plant sequenced [1-8]. The unusual compositional and structural features, as well as the lack of EST coverage for a large number of novel genes, require high-throughput experimental means that are not limited by the current annotations.

### Identification of the rice chromosome 10 transcriptome by tiling microarrays

In this study, we developed whole-chromosome oligonucleotide tiling microarrays, and demonstrated their utility in experimentally identifying the transcriptome of both *japonica *and *indica *chromosome 10. Because oligonucleotide tiling microarrays provide unbiased end-to-end coverage of the entire chromosome and measure transcriptional activity of gene models from multiple independent probes (Figure 2), they can detect the transcriptome in a comprehensive and unbiased way [19-21,23-25]. The tiling microarray analysis of rice chromosome 10 detected transcription of 86.4% BGI *indica *(2,428/2,809), 83.9% BGI *japonica *(2,319/2,764), and 81.9% TIGR *japonica *(2,472/3,019) gene models (Table 1). Using a set of the least reliable gene models (UG models, see below), RT-PCR analysis revealed disparity in gene structure of close to 50% of these models (Figure 3). These results are consistent with previous assessments of current computational gene finders, which can reliably locate a gene model in the correct chromosome locus, but are less than satisfactory to predict the fine gene structure [37,38].

Based on alignment to rice full-length cDNA and EST sequences, the gene models for both *japonica *and *indica *chromosome 10 were classified as UG, EG, and CG models (Table 1, Figure 2). This classification places the gene models in three groups with an ascending order of confidence, because the presence of an expressed sequence provides strong support to the corresponding model. In keeping with this idea, these three classes of gene models were also detected by tiling microarrays in an ascending order (Table 1). This result, together with the high detection rate of CG models, suggests that the chromosome 10 transcriptomes identified by the tiling microarrays are rather exhaustive. In support of this conclusion, tiling array analysis of rice seedlings which had undergone severe stress treatments only identified an additional 39 (less than 1.7% of the total detected) models. These results likely can be attributed to the high sensitivity of the tiling microarrays such that even if activation of certain genes is conditional, the basal level transcripts could still be detected by the tiling microarray.

Therefore, the UG models (particularly UG/LH) that failed to be detected by the tiling microarray need to be more closely inspected (Table 1, Figure 3). We did find that the gene models specifically detected following the stress treatments were enriched with UG/LH models (23/39), suggesting that some UG/LH might be stress responsive and their expression is not readily detectable under normal conditions. It should be noted that though redundant gene models such as those derived from long terminal repeat (LTR) retrotransposons and Pack-MULEs are generally under-represented in the expressed sequence collections [12,39], many are stress responsive and share similar *cis*-elements with plant defense genes [40]. Thus, it cannot be ruled out that some of the UG/LH models are related to low copy number retrotransposons with unusual structures.

Reasoning that the tiling microarray-detected transcriptome is both exhaustive and reliable, tiling microarray-supported gene models were mapped and integrated. This analysis identified 363 unique BGI *japonica*, 114 unique BGI *indica*, and 72 novel models that could be integrated into the TIGR *japonica *gene model set to comprehensively represent the *japonica *chromosome 10 transcriptome (Figure 5). Note that the added gene models do not necessarily increase the number of *japonica *chromosome 10 genes, even if their transcription was detected. As elaborated above, some of these gene models could be unrecognized TEs, uncharacterized UTRs or alternative exons. However, as all these extra gene models are transcribed, their identification will not only better represent the transcriptome, but further examination of these elements will also yield insight into rice genome composition and structure.

Extensive antisense transcription was observed for the rice chromosome 10 gene models. For instance, in a preliminary analysis whereby regions of the antisense strand covering the 3,019 TIGR *japonica *gene models were examined, excluding those that contain less than three signal oligos, 591 (19.6%) were found to have antisense expression. The proportion of rice gene models showing antisense transcription is consistent with that reported from tiling microarray analyses in *Arabidopsis *[23] and human [24,25], adding to an increasing body of evidence that indicates antisense transcription as an inherent property of the genomes. However, it should be cautioned that the potential effects of several experimental artifacts such as unintended second-strand synthesis, formation of specific RNA-DNA hybrids, or spurious priming events during target preparation have to be precisely assessed before a final conclusion on the nature and extent of antisense transcription in rice can be drawn.

Transcriptional activities outside the annotated gene models in the form of intergenic TARs, accounted for approximately 3% of the chromosome size (Figure 4a). RNA gel blotting and RT-PCR analyses confirmed only a portion of the selected TARs (Figure 3, 4), suggesting that the unconfirmed TARs could be experimental artifacts or correspond to transcripts of extreme low abundance [21,25,27]. Transcriptome components outside of previously annotated gene models are expected to correspond to: novel genes with unusual sequence composition; under-represented UTRs or exons of splice variants; nonprotein coding RNA transcripts; or uncharacterized transcribed TEs. RT-PCR analysis of selected *japonica *intergenic TARs suggests that the majority of the TARs belong to the first two groups (Figure 3b). This conclusion is consistent with the observation that the intergenic TARs were slightly enriched in regions of the chromosome with lower gene density (Figure 7d). A preliminary analysis whereby 214 plant miRNAs (including 122 from rice and 92 from *Arabidopsis*) [41,42] were used in a BLAST search against the intergenic TARs revealed no significant hits, suggesting that the TARs do not contain known plant microRNAs.

We thus focused our efforts on further analyzing the first two groups of TARs. For the current rice annotation, five different gene finders (primarily FGENESH) were used to generate gene models [8]. To annotate the intergenic TARs, we used the relatively new rice gene-finder program BGF [2,6,30], which identified 72 novel gene models (Figure 5). Sequence comparison between the 40 cloned intergenic TAR transcripts and the novel BGF models showed that 23 (57.5%) were predicted (Figure 3b), indicating that the BGF program was able to detect slightly more than half of the novel transcriptional units that might be represented by the intergenic TARs. Extrapolation from these observations suggests that there might be up to 2,000 novel genes yet to be recognized by current rice gene finders; however, the incomplete nature of the cloned transcripts made it difficult to unambiguously determine whether they encode proteins. Thus, it is possible that some of these transcripts may correspond to noncoding RNAs.

### Association of chromosomal architecture with transcriptional activity

Eukaryotic genomes contain heterochromatin as cytologically intensely staining nuclear materials that are thought to be composed mainly of noncoding DNA and silent transposons [33,43]. A salient feature of rice chromosome 10 is that its heterochromatin is not limited to the pericentric regions, but includes the entire short arm as well as the proximal portion of the long arm [33]. Comparison of cytological and sequence data suggests that this heterochromatin region is roughly 11-12 Mb in length [5,33]. Although recent genetic and microarray studies in plants have indicated a role for gene regulation by well defined small heterochromatin regions [44-47], virtually no data are available regarding the association of transcriptional activity with large-scale heterochromatin domains in regulating gene expression, chromosome behavior, and genome evolution.

Profiling the transcriptional activities of rice chromosome 10 using tiling microarrays revealed that gene expression in the heterochromatin region is generally low under normal growth conditions (the N Arrays) relative to the euchromatin (Figure 6a-c). Consistent with this observation, gene model distribution showed that the heterochromatin domain is relatively low in CG models but more abundant in UG models (Figure 7b). In support of the cytological data, an enrichment of TE models in the heterochromatin domain is evident (Figure 7a) [5]. Exclusion of the high copy number TEs and repetitive sequences from the tiling microarray analysis might contribute to the lower gene model density in the heterochromatin (Figure 7a-c); however, the generally lower detection rate of gene expression indicates that expression of many non-TE models is also somewhat repressed (Figure 7a-c). Interestingly, when plants were subjected to mineral or nutrient stresses, a general activation of transcription was observed in the heterochromatin (Figure 6d). These results are consistent with findings that heterochromatin stability and heterochromatin-mediated gene silencing can be regulated by development [48,49] or by modulating levels of specific transcription factors [50].

The distribution of TE and non-TE gene models in the heterochromatic and euchromatic regions was a near mirror image (Figure 7a). This result suggests that the heterochromatin and euchromatin may have similar capacities to accommodate protein-coding gene models (TE and non-TE), even though the heterochromatin is enriched with repetitive sequences (Figure 6a) [5]. Furthermore, the heterochromatin is relatively enriched with LH models and low in CG models compared with the euchromatin (Figure 7b, c). Thus it is likely that the differential packaging of genome elements in heterochromatin and euchromatin might enable rice to regulate and coordinate gene expression at the chromosomal level. Although the underlying molecular mechanism of this regulation is currently unknown, DNA methylation, histone modifications, and small interfering RNAs have all been implicated [51-55].

The distance between corresponding *japonica *and *indica *CG models along the chromosome was more skewed in the heterochromatin, with many CG genes shuffled more than 1 Mb in physical distance from the location of their orthologous counterparts. In contrast, the gene distance in the euchromatin is largely homogeneous (Figure 8). Previous studies have shown a mosaic organization of grass genomes where conserved sequences are disrupted by nonconserved sequences, and that gene amplification, movement, and activity of retrotransposons account for the bulk of the interspersing nonconserved sequences [56-58]. Thus, these results collectively indicate that the heterochromatin domain is more evolutionarily active and compositionally dynamic. Such a conclusion is in keeping with the genomic stress hypothesis that TEs are involved in host adaptation to environmental changes [39,40,59].

## Materials and methods

### Plant materials and treatments

*Oryza sativa *ssp. *japonica *cv. Nipponbare and *Oryza sativa *ssp. *indica *cv. *93-11 *were used for all experiments. Seeds were surface-sterilized, imbibed at 37°C for 2 days, and then transferred to MS medium (Invitrogen) solidified with 0.8% (w/v) agar. Seedlings were kept under continuous light at 28°C for seven days before harvest for total RNA isolation. Alternatively, 7-day-old seedlings were transferred to soil and maintained under long-day conditions (16 h light/8 h dark) at 26-28°C in the greenhouse until flowering. Heading and filling stage panicles were then collected from these plants. Suspension-cultured cells were prepared and maintained as previously described [60]. For stress treatment, *japonica *seedlings were grown for seven days on MS medium under four different conditions: MS medium deprived of nitrogen; MS medium deprived of phosphorus, or supplemented with 150 mM NaCl or 100 μM CdSO_4_. For RNA isolation, plant materials were frozen in liquid nitrogen and homogenized. Total RNA and mRNA were isolated using the RNeasy Plant Mini kit (Qiagen) and the Oligotex mRNA kit (Qiagen) according to the manufacturer's recommendations, respectively.

### MAS microarray design, production, and hybridization

Based on the MAS platform, a minimal tiling strategy was designed to effectively represent the nonrepetitive sequences of rice chromosome 10 [24,26]. Briefly, 36-mer oligonucleotides were designed using an algorithm based on sequence-dependent factors such as length, extent of complementarity, and the overall base composition. Oligos that could form a stem-loop structure with stem length greater than seven bases and those that have an oligo index score greater than 5 were excluded. To calculate the index score for each oligo, the 20 possible consecutive 17-mer sequences within each oligo were searched against the whole genome. The average copy number of the 17-mer sequences was scored as the oligo index. MAS microarray production was performed as previously described [24,26,29] using the sequences of chromosome 10 for *japonica *and *indica *rice as were available on 12 April, 2004 [8] and 1 August, 2003 [6,30], respectively. Oligos were synthesized at a density of 389,000 oligos per array in a chessboard design wherein each positive feature, which contains an interrogating oligo, was surrounded by four negative features and vice versa.

The *japonica *and *indica *N Arrays both included four individual MAS arrays that contain oligos representing other portions of the genome (other than chromosome 10) not analyzed in the current study. The N Arrays were hybridized to cDNA target mixtures derived in equal amounts from seedling roots, seedling shoots, panicles, and suspension-cultured cells of both *japonica *(cv. Nipponbare) and *indica *(cv. *93-11*) rice. Additionally, a set of two *japonica *arrays (S Arrays) were hybridized to targets derived from pooled poly(A)^+ ^RNA isolated from leaves of stress-treated *japonica *seedlings. Target preparation, array hybridization, and hybridization intensity value acquisition were carried out as previously described [24,26,29,61]. Tiling microarray design and experimental data are available in the National Center for Biotechnology Information (NCBI) Gene Expression Omnibus under series GSE2500.

### Chromosome 10 gene model compilation

The *japonica *(TIGR Rice Pseudomolecule released on 12 April 2004) [8] and *indica *(released by BGI on 1 August 2003) [6,30] chromosome 10 annotations were used in this study. In addition, the *japonica *chromosome 10 sequence was annotated using the BGI gene prediction flow to generate the BGI *japonica *gene model set. All gene models were aligned to a collection of rice full-length cDNA sequences [15] and all available rice EST sequences in GenBank [17] as of 15 April 2004 by the BLAT program [62] using cutoff criteria of 100 bp overlap and 90% identity over the entire length of each match. The predicted genes without matches to cDNA and EST sequences, excluding those with coding capacities of less than 50 amino acids, were classified as UG models.

### Determination of gene model expression and identification of intergenic TARs

Hybridization intensity of all positive and all negative features within each array was plotted separately and then scaled to have a peak log_2 _intensity of 8.0 (Figure 1a,b). Signal and noise probe determination is shown in Figure 1c and discussed in main text. Expression level of a given gene model was represented by the value of hybridization intensity (*HI*) of this model locus that takes into account two parameters: *FI*, which is the mean of fluorescence intensity of all signal probes of a given gene model, and hybridization rate (*HR*), which is defined as the percentage of signal probes over total interrogating probes per kilobase of genomic sequence. *HI *is calculated using the formula *HI *= *FI *+ *FI *× (*HR*_*E *_- *HR*_*M*_) in which *HR*_*E *_is *HR *of the exon regions whilst *HR*_*M *_is the mean *HR *of all intron regions. *HI *value of each model was then compared against a threshold designated as the mean fluorescence intensity plus twice the standard deviation (95% confidence) of all noise probes within each array.

To identify intergenic TARs, HR was calculated in a sliding window of 500 nucleotides across the intergenic regions of chromosome 10 with a bandwidth equal to an interrogating probe. Windows with HR above a threshold of 0.4 were considered positive. Contiguously transcribed regions (TARs) were generated by joining overlapping positive windows that were delineated by the 5' probe of the first window and 3' probe of the last. TARs less than 220 bp (five consecutive probes) long were discarded. The *japonica *intergenic TARs were first identified using the BGI *japonica *annotation, followed by comparison with TIGR models. TARs overlapping with TIGR models were masked. Sequences of all retained intergenic TARs were aligned to the BGF gene predictions, and were used to BLASTX search the nonredundant protein database SWISS-PROT. Those BGF-predicted genes that overlap more than 100 bp with the sequence of intergenic TARs on the same strand of DNA were considered positive.

### Cloning and verification of UG models and intergenic TARs

Selected UG models were cloned by means of RT-PCR. The PCR products were cloned into the pGEM-T vector (Promega) and sequenced. To clone intergenic TARs with downstream sequence, reverse transcription was performed on mixed poly(A)^+ ^RNA derived from seedling roots, seedling shoots, panicles and suspension-cultured cells of *japonica *rice using the primer RT-CPK (5'-TGCAGTCTAGCTGGAATGACCTCATTGCAGAAT_24_). The PCR procedure to clone the TARs was carried out using a cascade of thermal asymmetric interlaced PCR cycles [63,64] that employ three consecutively nested gene-specific primers to pair with primer RT-1 (5'-GCAGTCTAGCTGGAAT), RT-2 (5'-CTGGAATGACCTCATT), and RT-3 (5'-GCTGGAATGACCTCATTGCAGAAT), which anneal to overlapping regions of RT-CPK. Sequences of all the cloned PCR products were aligned back to *japonica *chromosome 10 using BLAT [62] to confirm their identify and to map their corresponding gene structure. RNA gel-blot analysis of intergenic TARs was conducted as previously described [65].

### Integration of *japonica *chromosome 10 gene models

All *japonica *chromosome 10 related gene models were sorted, and only those that met certain criteria were retained. The TIGR nonredundant gene models that can be mapped to the *japonica *chromosome 10 sequence were all retained. The additional models included BGI *japonica*, BGI *indica *models mapped to *japonica *chromosome 10, and tiling array-derived novel BGF models. From these models, those without previous full-length cDNA/EST or tiling microarray support, or those overlapping with TIGR models were discarded. All retained models were aligned back to the *japonica *chromosome 10 sequences to further confirm their identities and were combined with the TIGR *japonica *models.

## Additional data files

The following additional data files are available with the online verison of this paper. Additional data file 1 contains a table of integrated *japonica *chromosome 10 nonredundant gene models. Additional data file 2 contains a table of *indica *chromosome 10 nonredundant gene models. Additional data file 3 contains a table of the sequence analysis of cloned UG models. Additional data file 4 contains *japonica *chromosome 10 intergenic TARs. Additional data file 5 contains the sequence analysis of cloned intergenic TARs. Additional data file 6 contains a comparison of BGI and TIGR *japonica *chromosome 10 gene models. Additional data file 7 contains a comparison of BGI *indica *and *japonica *chromosome 10 gene models.

## Supplementary Material

Additional File 1 Table S1. Integrated *japonica *chromosome 10 nonredundant gene models. Integrated *japonica *chromosome 10 nonredundant gene models. Click here for file

Additional File 2 Table S2: *Indica *chromosome 10 nonredundant gene models. *Indica *chromosome 10 nonredundant gene models. Click here for file

Additional File 3 Table S3: Sequence analysis of cloned UG models. Sequence analysis of cloned UG models. Click here for file

Additional File 4 Table S4: *Japonica *chromosome 10 intergenic TARs. *Japonica *chromosome 10 intergenic TARs. Click here for file

Additional File 5 Table S5: Sequence analysis of cloned intergenic TARs. Sequence analysis of cloned intergenic TARs. Click here for file

Additional File 6 Table S6: Comparison of BGI and TIGR *japonica *chromosome 10 gene models. Comparison of BGI and TIGR *japonica *chromosome 10 gene models. Click here for file

Additional File 7 Table S7: Comparison of BGI *indica *and *japonica *chromosome 10 gene models. Comparison of BGI *indica *and *japonica *chromosome 10 gene models. Click here for file

## Figures and Tables

**Table 1:** Classification and array detection of rice chromosome 10 gene models

Annotation	Nonredundant protein-coding gene model				TE
	Type	Annotated	Detected	Percentage	
BGI *indica*	CG	821	784	95.5%	
	EG	328	290	88.4%	
	UG	1,660	1,354	81.6%	
	Total	2,809	2,428	86.4%	574
					
BGI *japonica*	CG	943	879	93.2%	
	EG	272	238	87.5%	
	UG	1,549	1,202	77.6%	
	Total	2,764	2,319	83.9%	851
					
TIGR *japonica*	CG	935	871	93.2%	
	EG	321	291	90.7%	
	UG	1,763	1,310	74.3%	
	Total	3,019	2,472	81.9%	829

Rice chromosome 10 protein-coding gene models were divided into TE and nonredundant models based on available annotations. Because of their repetitiveness, expression of TE models was not assessed. The nonredundant models were further divided into CG, EG and UG models based on their alignment to rice full-length cDNAs and ESTs and their expression assessed by tiling microarray analysis.
