# Supplementary material for: Tiling microarray analysis of rice chromosome 10 to identify the transcriptome and relate its expression to chromosomal architecture
Source: Genome Biol. 2005 May 27;6(6):R52. doi: 10.1186/gb-2005-6-6-r52 (PMC1175972; doi:10.1186/gb-2005-6-6-r52)
Supplement: Additional File 1 — Table S1. Integrated japonica chromosome 10 nonredundant gene models. Integrated japonica chromosome 10 nonredundant gene models. [file gb-2005-6-6-r52-S1.pdf]

Supplemental Table 1. Integrated *japonica* chromosome 10 non-redundant gene models

GeneName	Origin [1]	OldName	Type [2]	Homology [3]	Position [4]	Strand	Exon #	Length	Detection [5]	Intensity	ExonHR [6]	IntronHR [6]
OsJN00001	TIGR	9638.m02898	HP	LH	16899067	+	3	428	P	1381.454	0.571	0.333
OsJN00002	TIGR	9638.m03310	PP	LH	19349100	+	3	1331	P	1407.479	0.353	0.091
OsJN00003	TIGR	9638.m02919	HP	LH	17072667	+	1	224	P	1419.177	0.5	0
OsJN00004	TIGR	9638.m03265	HP	LH	19095925	+	3	2494	P	1180.638	0.286	0.103
OsJN00005	TIGR	9638.m03473	PP	HH	20094138	+	2	1308	A	661.139	0.08	0
OsJN00006	TIGR	9638.m02258	EP	HH	13276031	+	10	6221	P	2715.382	0.069	0.04
OsJN00007	TIGR	9638.m03717	HP	LH	21478957	+	3	1184	P	2055.196	0.2	0.2
OsJN00008	TIGR	9638.m03056	PP	LH	17974101	+	4	9737	P	905.204	0.429	0.153
OsJN00009	TIGR	9638.m02470	PP	HH	14623658	+	2	1068	P	2069.09	0.136	0
OsJN00010	TIGR	9638.m03271	EP	HH	19118686	+	6	4573	P	1223.589	0.194	0.081
OsJN00011	TIGR	9638.m02575	PP	HH	15100712	+	2	1074	P	1973.923	0.333	0
OsJN00012	TIGR	9638.m03358	PP	HH	19588964	+	8	2847	P	1361.592	0.139	0.115
OsJN00013	TIGR	9638.m02385	PP	HH	14177541	+	2	790	P	2403.444	0.364	0.286
OsJN00014	TIGR	9638.m03378	PP	HH	19672717	+	3	3521	P	1168.253	0.25	0.045
OsJN00015	TIGR	9638.m02925	PP	HH	17105875	+	4	5858	P	1460.625	0.134	0.333
OsJN00016	TIGR	9638.m02515	HP	LH	14801864	+	1	672	A	417.567	0.133	0
OsJN00017	TIGR	9638.m03038	HP	HH	17901275	+	2	1060	A	460.362	0.043	0
OsJN00018	TIGR	9638.m03611	PP	HH	20831717	+	1	978	P	1286.355	0.727	0
OsJN00019	TIGR	9638.m02960	PP	HH	17348361	+	3	2267	P	959.756	0.171	0.067
OsJN00020	TIGR	9638.m02026	HP	LH	11900560	+	2	756	A	0	0	0
OsJN00021	TIGR	9638.m03851	HP	LH	22170288	+	3	1177	P	1962.569	0.5	0
OsJN00022	TIGR	9638.m03694	HP	LH	21324215	+	2	264	P	1245.514	0.5	0
OsJN00023	TIGR	9638.m02999	HP	HH	17718544	+	1	732	P	1377.537	0.235	0
OsJN00024	TIGR	9638.m03324	PP	HH	19405522	+	18	5428	P	1123.277	0.197	0.018
OsJN00025	TIGR	9638.m02044	HP	LH	11993754	+	3	858	A	718.453	0.083	0.5
OsJN00026	TIGR	9638.m02508	PP	HH	14770908	+	2	1094	P	3105.513	0.045	0
OsJN00027	TIGR	9638.m03906	EP	HH	22488080	+	3	3125	P	1364.411	0.127	0
OsJN00028	TIGR	9638.m03878	PP	HH	22314900	+	2	830	P	1191.383	0.353	0
OsJN00029	TIGR	9638.m03862	PP	HH	22247315	+	5	2850	P	1177.18	0.047	0.053
OsJN00030	TIGR	9638.m03668	HP	LH	21162431	+	1	321	P	1600.435	0.286	0
OsJN00031	TIGR	9638.m03156	EP	HH	18552896	+	2	2213	P	1244.968	0.214	0.056
OsJN00032	TIGR	9638.m02173	HP	LH	12780117	+	2	498	P	1118.631	0.5	0.2
OsJN00033	TIGR	9638.m02267	HP	LH	13340389	+	11	5428	P	1395.643	0.033	0.053
OsJN00034	TIGR	9638.m03435	EP	HH	19969587	+	1	2253	P	1287.064	0.556	0
OsJN00035	TIGR	9638.m02041	HP	LH	11975167	+	2	893	A	385.567	0.091	0
OsJN00036	TIGR	9638.m03780	EP	HH	21781426	+	1	1551	P	1630.95	0.424	0
OsJN00037	TIGR	9638.m03510	EP	HH	20265555	+	4	8569	P	2340.806	0.154	0.17
OsJN00038	TIGR	9638.m03300	PP	HH	19307323	+	1	1788	P	1957.832	0.158	0
OsJN00039	TIGR	9638.m03847	PP	HH	22137982	+	12	5870	P	1613.326	0.224	0.027
OsJN00040	TIGR	9638.m03306	PP	HH	19329197	+	1	612	P	1884.92	0.286	0
OsJN00041	TIGR	9638.m02630	PP	HH	15428066	+	14	7932	P	1238.35	0.041	0.061
OsJN00042	TIGR	9638.m03606	HP	LH	20813414	+	4	3003	P	1087.299	0.138	0.054
OsJN00043	TIGR	9638.m02330	EP	HH	13827919	+	1	824	P	3079.817	0.316	0
OsJN00044	TIGR	9638.m03524	HP	LH	20330370	+	2	555	P	1545.099	0.455	1
OsJN00045	TIGR	9638.m03411	PP	HH	19852141	+	1	582	P	2459.949	0.462	0
OsJN00046	TIGR	9638.m03050	HP	LH	17946690	+	4	1889	A	504.178	0.167	0
OsJN00047	TIGR	9638.m02902	EP	HH	16936747	+	3	3194	P	1092.665	0.239	0
OsJN00048	TIGR	9638.m03259	EP	LH	19060877	+	4	1529	A	0	0	0
OsJN00049	TIGR	9638.m03815	EP	HH	21986314	+	1	2972	P	1981.267	0.25	0
OsJN00050	TIGR	9638.m02825	EP	LH	16521862	+	4	5118	P	1320.224	0.333	0.08
OsJN00051	TIGR	9638.m02613	EP	LH	15320261	+	2	540	A	0	0	0

OsJN00052	TIGR	9638.m03219	PP	HH	18853930	+	20	6377	P	2393.342	0.048	0.091
OsJN00053	TIGR	9638.m02409	PP	LH	14284383	+	2	1542	P	1863.994	0.406	0
OsJN00054	TIGR	9638.m01989	HP	HH	11720910	+	1	342	P	3153.127	0.111	0
OsJN00055	TIGR	9638.m03503	PP	HH	20222115	+	7	5950	P	1300.841	0.165	0
OsJN00056	TIGR	9638.m03472	PP	HH	20092133	+	2	1048	P	1600.114	0.318	0
OsJN00057	TIGR	9638.m02266	HP	LH	13337956	+	1	537	P	2806.492	0.833	0
OsJN00058	TIGR	9638.m02221	HP	LH	13018158	+	1	357	A	0	0	0
OsJN00059	TIGR	9638.m02991	HP	LH	17676454	+	1	957	P	2365.882	0.4	0
OsJN00060	TIGR	9638.m03941	PP	HH	22643551	+	6	2150	P	2629.093	0.286	0.05
OsJN00061	TIGR	9638.m03192	PP	HH	18712678	+	1	1619	P	845.181	0.206	0
OsJN00062	TIGR	9638.m02951	PP	HH	17266137	+	7	5168	P	747.137	0.194	0.025
OsJN00063	TIGR	9638.m03206	HP	LH	18788568	+	3	6343	P	1094.048	0.375	0.052
OsJN00064	TIGR	9638.m03108	EP	HH	18293608	+	3	3759	P	1027.564	0.118	0.069
OsJN00065	TIGR	9638.m02831	PP	HH	16562486	+	9	3388	P	1600.805	0.119	0.231
OsJN00066	TIGR	9638.m03295	EP	LH	19288113	+	2	1004	P	964.939	0.111	0.091
OsJN00067	TIGR	9638.m02811	HP	LH	16478288	+	3	1947	P	1745.828	0.435	0
OsJN00068	TIGR	9638.m02225	EP	HH	13040874	+	13	8219	A	530.436	0.071	0.098
OsJN00069	TIGR	9638.m03840	HP	LH	22092137	+	5	6249	P	2215.708	0.348	0.161
OsJN00070	TIGR	9638.m02992	PP	LH	17684210	+	3	1368	P	2178.326	0.115	0
OsJN00071	TIGR	9638.m02662	EP	HH	15620544	+	9	6171	P	1747.203	0.164	0.091
OsJN00072	TIGR	9638.m03890	PP	HH	22417576	+	3	2377	P	1781.494	0.207	0
OsJN00073	TIGR	9638.m02243	HP	LH	13143717	+	2	270	A	451.136	0.143	0
OsJN00074	TIGR	9638.m03061	HP	HH	18039616	+	1	2097	P	1529.425	0.174	0
OsJN00075	TIGR	9638.m03348	EP	HH	19528677	+	14	5605	P	2960.36	0.107	0.015
OsJN00076	TIGR	9638.m02775	HP	LH	16318826	+	2	1112	P	1461.546	0.333	0
OsJN00077	TIGR	9638.m03462	PP	HH	20056653	+	2	1116	P	1244.765	0.304	0
OsJN00078	TIGR	9638.m03854	PP	HH	22177145	+	8	2117	P	2669.663	0.133	0.063
OsJN00079	TIGR	9638.m02352	HP	LH	13951995	+	1	438	P	976.484	0.111	0
OsJN00080	TIGR	9638.m02011	HP	LH	11820675	+	2	865	P	1543.423	0.375	0.333
OsJN00081	TIGR	9638.m03174	HP	LH	18634482	+	2	1055	P	887.898	0.429	0.077
OsJN00082	TIGR	9638.m01999	HP	LH	11777601	+	2	850	P	2705.931	0.286	0
OsJN00083	TIGR	9638.m03931	PP	HH	22604560	+	2	976	P	1535.092	0.333	0
OsJN00084	TIGR	9638.m03766	PP	HH	21710174	+	3	1564	P	1936.303	0.385	0
OsJN00085	TIGR	9638.m02871	HP	LH	16743415	+	1	288	P	1630.938	0.429	0
OsJN00086	TIGR	9638.m03073	EP	LH	18097636	+	1	827	P	889.367	0.211	0
OsJN00087	TIGR	9638.m01974	EP	LH	11603746	+	4	2319	A	1	0	0
OsJN00088	TIGR	9638.m03651	HP	LH	21089858	+	2	1129	P	1962.177	0.12	0
OsJN00089	TIGR	9638.m02502	PP	HH	14755153	+	2	1290	P	1125.695	0.2	0
OsJN00090	TIGR	9638.m02364	PP	HH	14032109	+	18	10318	A	587.1	0.063	0.077
OsJN00091	TIGR	9638.m02645	PP	HH	15516101	+	14	4543	P	633.467	0.169	0.077
OsJN00092	TIGR	9638.m03552	PP	HH	20487986	+	1	1257	A	462.505	0.036	0
OsJN00093	TIGR	9638.m02296	HP	LH	13592898	+	6	2897	A	626.7	0.278	0.087
OsJN00094	TIGR	9638.m03200	HP	LH	18753810	+	2	628	P	2402.92	0.273	0.667
OsJN00095	TIGR	9638.m02868	HP	LH	16725820	+	1	471	P	857.276	0.091	0
OsJN00096	TIGR	9638.m03831	EP	HH	22055432	+	4	3508	P	888.527	0.227	0.073
OsJN00097	TIGR	9638.m03402	PP	HH	19792398	+	12	4127	P	1221.968	0.167	0.038
OsJN00098	TIGR	9638.m02434	EP	HH	14443875	+	10	3610	P	1702.007	0.22	0.143
OsJN00099	TIGR	9638.m02290	HP	LH	13514050	+	1	168	P	1542.376	0.6	0
OsJN00100	TIGR	9638.m02989	PP	HH	17668180	+	2	3196	P	2679.876	0.219	0.056
OsJN00101	TIGR	9638.m03225	HP	LH	18877008	+	7	7697	P	940.044	0.297	0.174
OsJN00102	TIGR	9638.m02928	EP	HH	17124885	+	3	3078	A	1	0	0
OsJN00103	TIGR	9638.m03392	EP	HH	19754106	+	6	3406	P	1725.966	0.303	0.073
OsJN00104	TIGR	9638.m03145	PP	HH	18497856	+	5	2239	A	615.825	0.118	0.133
OsJN00105	TIGR	9638.m03102	EP	HH	18246322	+	8	2986	P	1889.908	0.056	0.034
OsJN00106	TIGR	9638.m03425	HP	LH	19928278	+	2	509	A	0	0	0
OsJN00107	TIGR	9638.m03802	HP	LH	21940050	+	2	855	P	1031.81	0.4	0

OsJN00108	TIGR	9638.m03391	EP	HH	19749247	+	3	2425	P	1834.027	0.29	0.05
OsJN00109	TIGR	9638.m02863	HP	HH	16697711	+	6	2135	A	518.853	0.167	0
OsJN00110	TIGR	9638.m02154	EP	LH	12668536	+	1	424	P	2836.199	0.1	0
OsJN00111	TIGR	9638.m03578	HP	LH	20658287	+	3	418	P	2423.689	0.6	0.5
OsJN00112	TIGR	9638.m03357	PP	HH	19588799	+	8	3239	P	1228.557	0.178	0.115
OsJN00113	TIGR	9638.m03874	HP	LH	22300296	+	2	643	P	3444.921	0.25	0
OsJN00114	TIGR	9638.m03509	PP	HH	20261222	+	1	2025	P	668.368	0.159	0
OsJN00115	TIGR	9638.m02577	PP	HH	15113480	+	1	1894	P	1017.046	0.275	0
OsJN00116	TIGR	9638.m02748	HP	LH	16142803	+	3	1685	P	854.386	0.111	0.038
OsJN00117	TIGR	9638.m02588	HP	LH	15188043	+	2	1078	P	824.579	0.385	0
OsJN00118	TIGR	9638.m02012	EP	HH	11823424	+	12	9328	P	1005.253	0.098	0.028
OsJN00119	TIGR	9638.m03715	PP	HH	21468903	+	4	5186	P	1338.635	0.094	0.056
OsJN00120	TIGR	9638.m03246	PP	HH	19002714	+	2	3714	A	1	0	0
OsJN00121	TIGR	9638.m03658	HP	LH	21112581	+	2	324	P	882.848	0.143	0
OsJN00122	TIGR	9638.m02259	HP	LH	13283086	+	2	678	A	635.648	0.333	0.667
OsJN00123	TIGR	9638.m03137	PP	HH	18475066	+	6	3056	P	1058.946	0.256	0
OsJN00124	TIGR	9638.m03160	EP	HH	18577717	+	5	2945	P	2394.882	0.226	0.188
OsJN00125	TIGR	9638.m02354	HP	LH	13966259	+	1	408	P	1596.71	0.444	0
OsJN00126	TIGR	9638.m02514	EP	HH	14799867	+	1	617	P	4245.693	0.071	0
OsJN00127	TIGR	9638.m03168	PP	HH	18595889	+	4	3667	P	1772.151	0.171	0.067
OsJN00128	TIGR	9638.m02500	PP	HH	14744278	+	1	1466	P	2493.314	0.156	0
OsJN00129	TIGR	9638.m03707	PP	LH	21440423	+	2	1825	P	1340.563	0.2	0
OsJN00130	TIGR	9638.m02859	HP	HH	16685879	+	11	3288	P	1421.722	0.143	0.023
OsJN00131	TIGR	9638.m03903	PP	HH	22478738	+	4	4533	P	1853.093	0.3	0.135
OsJN00132	TIGR	9638.m02993	PP	HH	17687394	+	2	1212	P	1327.674	0.143	0
OsJN00133	TIGR	9638.m02142	HP	LH	12598240	+	2	529	A	0	0	0.25
OsJN00134	TIGR	9638.m03268	HP	HH	19112292	+	1	822	P	1883.751	0.313	0
OsJN00135	TIGR	9638.m02122	PP	LH	12487992	+	2	1363	P	1290.631	0.103	0
OsJN00136	TIGR	9638.m03436	EP	HH	19971865	+	1	2205	P	5652.405	0.792	0
OsJN00137	TIGR	9638.m02431	EP	HH	14421189	+	7	3112	P	759.289	0.235	0.091
OsJN00138	TIGR	9638.m03266	EP	HH	19104017	+	5	3885	P	1052.336	0.244	0.125
OsJN00139	TIGR	9638.m03143	PP	HH	18493781	+	4	1994	P	882.213	0.25	0.167
OsJN00140	TIGR	9638.m02607	EP	HH	15279868	+	13	7125	P	1705.427	0.227	0.027
OsJN00141	TIGR	9638.m02494	HP	LH	14723916	+	1	345	P	1852.838	0.333	0
OsJN00142	TIGR	9638.m03103	EP	HH	18249835	+	7	3227	P	1603.37	0.128	0.087
OsJN00143	TIGR	9638.m02696	EP	HH	15882630	+	2	3168	P	972.5	0.314	0.192
OsJN00144	TIGR	9638.m03379	PP	HH	19678755	+	5	4435	P	1784.176	0.212	0.049
OsJN00145	TIGR	9638.m02974	EP	HH	17500128	+	7	4941	P	1114.385	0.286	0.056
OsJN00146	TIGR	9638.m03383	HP	LH	19699827	+	2	2280	A	0	0	0.108
OsJN00147	TIGR	9638.m03009	PP	HH	17769385	+	5	4198	P	1376.493	0.212	0.051
OsJN00148	TIGR	9638.m03412	HP	LH	19855381	+	3	556	P	1242.238	0.222	0.333
OsJN00149	TIGR	9638.m02534	HP	LH	14906546	+	2	1189	A	0	0	0.227
OsJN00150	TIGR	9638.m03610	PP	HH	20828555	+	1	1164	P	2115.163	0.16	0
OsJN00151	TIGR	9638.m02023	HP	LH	11883013	+	1	531	P	2505.935	0.6	0
OsJN00152	TIGR	9638.m03545	PP	HH	20453948	+	1	1404	A	0	0	0
OsJN00153	TIGR	9638.m02486	PP	HH	14698705	+	1	1363	P	1239.16	0.167	0
OsJN00154	TIGR	9638.m01961	HP	LH	11524616	+	4	2529	P	1531.929	0.444	0.147
OsJN00155	TIGR	9638.m02676	EP	HH	15735023	+	5	3095	P	1035.151	0.196	0.111
OsJN00156	TIGR	9638.m02455	PP	HH	14543323	+	1	1107	P	1281.584	0.167	0
OsJN00157	TIGR	9638.m03331	HP	LH	19458494	+	2	668	A	0	0	0
OsJN00158	TIGR	9638.m02256	HP	LH	13263517	+	1	381	A	1	0	0
OsJN00159	TIGR	9638.m03405	PP	HH	19817144	+	7	5612	P	1859.501	0.121	0.188
OsJN00160	TIGR	9638.m02700	HP	LH	15906571	+	2	781	A	0	0	0
OsJN00161	TIGR	9638.m02351	HP	LH	13946948	+	1	531	P	1247.426	0.231	0
OsJN00162	TIGR	9638.m03693	EP	LH	21319334	+	1	1428	P	1687.979	0.357	0
OsJN00163	TIGR	9638.m02521	PP	HH	14823731	+	12	6738	P	1526.7	0.091	0.099

OsJN00164	TIGR	9638.m01987	EP	HH	11710884	+	2	1464	P	2631.846	0.167	0
OsJN00165	TIGR	9638.m03042	PP	LH	17914990	+	2	578	P	1490.782	0.4	0
OsJN00166	TIGR	9638.m02207	HP	LH	12953910	+	1	351	A	1	0	0
OsJN00167	TIGR	9638.m03788	PP	HH	21813068	+	3	4272	P	1626.394	0.362	0.022
OsJN00168	TIGR	9638.m03445	PP	HH	19985691	+	2	1251	P	1851.031	0.36	0
OsJN00169	TIGR	9638.m02983	PP	LH	17619817	+	4	3413	P	1409.692	0.154	0.042
OsJN00170	TIGR	9638.m02938	EP	LH	17201017	+	2	1123	P	522.692	0.5	0
OsJN00171	TIGR	9638.m03481	PP	HH	20113877	+	2	772	P	2075.72	0.188	0
OsJN00172	TIGR	9638.m03258	EP	LH	19058930	+	2	684	P	1484.062	0.214	0
OsJN00173	TIGR	9638.m02100	HP	LH	12340926	+	6	1401	A	387.169	0.05	0.091
OsJN00174	TIGR	9638.m03940	PP	HH	22643551	+	5	2150	P	2629.093	0.267	0.056
OsJN00175	TIGR	9638.m02149	EP	HH	12639633	+	5	2233	P	816.48	0.176	0.071
OsJN00176	TIGR	9638.m02027	HP	HH	11903380	+	6	3204	P	1280.473	0.122	0
OsJN00177	TIGR	9638.m02230	HP	LH	13061523	+	1	360	P	2001.948	0.5	0
OsJN00178	TIGR	9638.m02929	PP	LH	17132006	+	6	3800	A	1	0	0
OsJN00179	TIGR	9638.m03479	PP	HH	20107144	+	2	1257	P	1755.599	0.111	0
OsJN00180	TIGR	9638.m03540	HP	HH	20432182	+	2	672	P	3039.862	0.357	0
OsJN00181	TIGR	9638.m02932	HP	LH	17163361	+	4	1187	P	1285.252	0.059	0
OsJN00182	TIGR	9638.m03497	HP	LH	20174592	+	1	549	P	1498.319	0.273	0
OsJN00183	TIGR	9638.m03801	PP	LH	21932370	+	1	1563	P	1965.47	0.412	0
OsJN00184	TIGR	9638.m02942	PP	HH	17221740	+	2	2533	P	809.391	0.063	0.19
OsJN00185	TIGR	9638.m02689	HP	HH	15822699	+	4	3788	P	1393.856	0.167	0.178
OsJN00186	TIGR	9638.m03571	EP	LH	20602173	+	6	3815	P	1099.684	0.225	0.031
OsJN00187	TIGR	9638.m02363	HP	LH	14013503	+	3	1121	A	354.432	0.143	0
OsJN00188	TIGR	9638.m03705	HP	LH	21404686	+	9	7386	P	1051.716	0.24	0.037
OsJN00189	TIGR	9638.m02681	HP	LH	15771233	+	2	470	A	0	0	0
OsJN00190	TIGR	9638.m03332	PP	HH	19459858	+	9	8638	P	2398.535	0.108	0.087
OsJN00191	TIGR	9638.m03461	PP	HH	20053460	+	2	1406	P	2635.304	0.385	0
OsJN00192	TIGR	9638.m02031	HP	LH	11929760	+	1	669	P	2512.27	0.333	0
OsJN00193	TIGR	9638.m03647	HP	HH	21051684	+	5	2857	P	1171.89	0.25	0
OsJN00194	TIGR	9638.m03131	PP	LH	18422233	+	5	3298	A	0	0	0
OsJN00195	TIGR	9638.m02155	PP	HH	12669777	+	2	1467	P	1351.982	0.071	0
OsJN00196	TIGR	9638.m02532	HP	LH	14891312	+	1	762	P	1181.601	0.353	0
OsJN00197	TIGR	9638.m02390	HP	LH	14206740	+	3	2040	P	825.611	0.273	0.036
OsJN00198	TIGR	9638.m02242	HP	LH	13139255	+	2	646	P	2092.06	0.222	0
OsJN00199	TIGR	9638.m03153	HP	LH	18543656	+	2	1089	P	5048.131	0.143	0.059
OsJN00200	TIGR	9638.m02873	PP	HH	16750039	+	10	4817	P	1359.398	0.116	0.048
OsJN00201	TIGR	9638.m03830	PP	HH	22052833	+	5	2242	P	1050.371	0.314	0
OsJN00202	TIGR	9638.m03486	PP	HH	20130955	+	1	1051	P	1007.853	0.375	0
OsJN00203	TIGR	9638.m03239	EP	HH	18959436	+	1	1735	P	1568.979	0.297	0
OsJN00204	TIGR	9638.m02501	PP	HH	14748023	+	2	2145	P	1370.351	0.182	0.3
OsJN00205	TIGR	9638.m03760	PP	HH	21677847	+	1	1605	P	1635.392	0.147	0
OsJN00206	TIGR	9638.m03099	PP	HH	18232347	+	15	7665	P	1501.824	0.227	0.034
OsJN00207	TIGR	9638.m03570	HP	LH	20586124	+	2	3250	P	1215.316	0.167	0.065
OsJN00208	TIGR	9638.m03408	PP	LH	19837367	+	2	1679	P	2109.608	0.417	0.182
OsJN00209	TIGR	9638.m02278	HP	LH	13425616	+	1	1218	P	881.348	0.2	0
OsJN00210	TIGR	9638.m02454	HP	HH	14537937	+	4	2035	A	0	0	0.048
OsJN00211	TIGR	9638.m03393	PP	HH	19762286	+	2	2411	P	693.081	0.16	0
OsJN00212	TIGR	9638.m03296	PP	HH	19292633	+	1	4827	P	885.373	0.133	0
OsJN00213	TIGR	9638.m02870	HP	LH	16736010	+	1	270	P	1081.547	0.833	0
OsJN00214	TIGR	9638.m03551	PP	HH	20480110	+	1	1242	A	465.756	0.038	0
OsJN00215	TIGR	9638.m03446	PP	HH	19990396	+	2	869	P	2263.868	0.368	0
OsJN00216	TIGR	9638.m02776	EP	LH	16320423	+	2	3887	P	1891.634	0.135	0.095
OsJN00217	TIGR	9638.m02824	PP	LH	16518194	+	6	2479	P	1232.448	0.208	0.129
OsJN00218	TIGR	9638.m02152	PP	HH	12657629	+	2	3183	P	1303.779	0.137	0.056
OsJN00219	TIGR	9638.m03767	PP	HH	21713494	+	9	3847	P	1596.444	0.2	0.048

OsJN00220	TIGR	9638.m03460	PP	HH	20050728	+	2	970	P	1685.146	0.316	0
OsJN00221	TIGR	9638.m03795	HP	LH	21897557	+	4	1258	P	2094.887	0.188	0.083
OsJN00222	TIGR	9638.m03653	HP	LH	21097314	+	1	645	P	1555.828	0.615	0
OsJN00223	TIGR	9638.m03550	PP	HH	20476126	+	1	1149	P	3043.288	0.04	0
OsJN00224	TIGR	9638.m03169	PP	HH	18603141	+	2	1621	P	1611.699	0.242	0
OsJN00225	TIGR	9638.m02523	EP	LH	14837479	+	7	3053	P	1276.433	0.25	0.097
OsJN00226	TIGR	9638.m02566	HP	LH	15048048	+	2	2158	A	397.859	0.091	0
OsJN00227	TIGR	9638.m02099	HP	HH	12331920	+	10	6618	P	875.995	0.083	0.158
OsJN00228	TIGR	9638.m03394	PP	HH	19762679	+	1	2018	P	702.728	0.156	0
OsJN00229	TIGR	9638.m02554	PP	HH	15005837	+	1	1080	P	1099.507	0.08	0
OsJN00230	TIGR	9638.m03842	PP	HH	22113191	+	1	4575	P	664.932	0.25	0
OsJN00231	TIGR	9638.m03170	HP	LH	18607317	+	2	302	P	1764.83	0.2	0
OsJN00232	TIGR	9638.m03589	HP	HH	20687033	+	1	936	P	1436.649	0.238	0
OsJN00233	TIGR	9638.m03134	PP	HH	18445618	+	3	2975	P	714.655	0.105	0.143
OsJN00234	TIGR	9638.m03970	PP	HH	20509060	+	1	1218	P	1372.596	0.074	0
OsJN00235	TIGR	9638.m02944	PP	HH	17231869	+	12	3204	P	1042.16	0.093	0
OsJN00236	TIGR	9638.m03809	HP	HH	21964093	+	1	1590	P	1483.967	0.222	0
OsJN00237	TIGR	9638.m03736	EP	HH	21552074	+	6	1937	P	1880.531	0.294	0
OsJN00238	TIGR	9638.m03040	HP	LH	17907882	+	1	477	P	2057.814	0.455	0
OsJN00239	TIGR	9638.m03837	PP	LH	22079392	+	2	480	A	367.193	0.167	0
OsJN00240	TIGR	9638.m02730	HP	LH	16037007	+	1	609	P	1432.428	0.5	0
OsJN00241	TIGR	9638.m02526	EP	HH	14856112	+	1	888	P	3771.389	0.15	0
OsJN00242	TIGR	9638.m03088	EP	LH	18166328	+	4	2024	P	1488.221	0.231	0.107
OsJN00243	TIGR	9638.m02227	HP	LH	13055015	+	4	2229	P	1414.028	0.353	0.065
OsJN00244	TIGR	9638.m02448	PP	HH	14514795	+	1	1122	P	1153.245	0.24	0
OsJN00245	TIGR	9638.m02601	HP	LH	15241177	+	1	402	P	2653.674	0.5	0
OsJN00246	TIGR	9638.m02964	EP	HH	17413103	+	7	3942	P	1771.011	0.556	0.16
OsJN00247	TIGR	9638.m02344	PP	HH	13921452	+	2	3755	P	1870.792	0.167	0.109
OsJN00248	TIGR	9638.m03475	PP	HH	20096963	+	3	2018	P	2553.098	0.25	0.25
OsJN00249	TIGR	9638.m02975	PP	HH	17506246	+	4	2729	P	2760.428	0.118	0.073
OsJN00250	TIGR	9638.m03836	HP	LH	22075971	+	2	340	P	2227.251	0.167	0
OsJN00251	TIGR	9638.m03341	PP	HH	19511329	+	1	2376	P	1025.745	0.173	0
OsJN00252	TIGR	9638.m02513	PP	HH	14793321	+	2	1152	A	748.441	0.042	0
OsJN00253	TIGR	9638.m03933	PP	HH	22611755	+	5	3044	P	1708.586	0.196	0
OsJN00254	TIGR	9638.m02541	HP	LH	14943140	+	3	1326	P	1124.726	0.188	0.077
OsJN00255	TIGR	9638.m03587	PP	HH	20676615	+	5	1752	P	918.478	0.276	0.3
OsJN00256	TIGR	9638.m03617	HP	LH	20885292	+	3	1171	P	1089.5	0.167	0
OsJN00257	TIGR	9638.m03528	EP	HH	20339900	+	8	2083	P	1084.103	0.143	0.063
OsJN00258	TIGR	9638.m03886	PP	HH	22393740	+	2	3357	A	0	0	0
OsJN00259	TIGR	9638.m03596	HP	LH	20725837	+	1	999	P	956.127	0.227	0
OsJN00260	TIGR	9638.m02488	PP	HH	14704332	+	2	1043	P	1331.814	0.056	0
OsJN00261	TIGR	9638.m03307	PP	HH	19334941	+	1	1545	P	1952.545	0.176	0
OsJN00262	TIGR	9638.m03076	HP	LH	18106894	+	9	2837	A	0	0	0
OsJN00263	TIGR	9638.m02101	HP	LH	12343214	+	18	6607	P	798.625	0.077	0.065
OsJN00264	TIGR	9638.m02997	PP	HH	17707326	+	3	4883	P	983.306	0.167	0.167
OsJN00265	TIGR	9638.m03954	PP	HH	22687207	+	2	2142	P	875.758	0.185	0.056
OsJN00266	TIGR	9638.m02419	PP	HH	14339231	+	2	2243	P	1222.767	0.106	0
OsJN00267	TIGR	9638.m02432	EP	HH	14425282	+	7	3763	P	1624.075	0.157	0.032
OsJN00268	TIGR	9638.m03376	PP	HH	19663275	+	11	6855	P	1600.982	0.122	0.028
OsJN00269	TIGR	9638.m03257	EP	HH	19053287	+	3	2295	P	760.704	0.208	0.269
OsJN00270	TIGR	9638.m02917	PP	HH	17063440	+	3	7289	P	1652.712	0.189	0
OsJN00271	TIGR	9638.m03785	PP	HH	21803519	+	6	2165	P	970.349	0.348	0.08
OsJN00272	TIGR	9638.m03007	PP	HH	17751896	+	3	3423	P	1371.238	0.153	0.2
OsJN00273	TIGR	9638.m02257	EP	LH	13271044	+	2	384	A	1	0	0
OsJN00274	TIGR	9638.m03641	PP	HH	21003964	+	6	5799	A	0	0	0.083
OsJN00275	TIGR	9638.m03937	PP	HH	22629348	+	11	6519	P	2196.181	0.189	0.125

OsJN00276	TIGR	9638.m03648	EP	HH	21055248	+	7	5199	P	1441.909	0.115	0.037
OsJN00277	TIGR	9638.m03562	EP	HH	20524531	+	8	4759	P	1353.734	0.226	0.098
OsJN00278	TIGR	9638.m03892	HP	LH	22431966	+	3	1129	P	1858.78	0.5	0.154
OsJN00279	TIGR	9638.m03701	HP	LH	21369581	+	4	1539	P	3290.041	0.182	0.211
OsJN00280	TIGR	9638.m02854	HP	HH	16666013	+	2	410	P	1556.977	0.333	0
OsJN00281	TIGR	9638.m02540	HP	LH	14937901	+	1	477	A	0	0	0
OsJN00282	TIGR	9638.m03029	PP	LH	17851553	+	3	1931	P	2432.766	0.211	0.133
OsJN00283	TIGR	9638.m02254	HP	HH	13253623	+	10	7010	A	1	0	0
OsJN00284	TIGR	9638.m02901	PP	HH	16930667	+	2	1472	P	2253.235	0.387	0
OsJN00285	TIGR	9638.m02643	HP	LH	15498649	+	5	1839	A	0	0	0.038
OsJN00286	TIGR	9638.m03339	EP	LH	19508583	+	1	443	P	2910.452	0.2	0
OsJN00287	TIGR	9638.m02495	PP	HH	14727427	+	2	965	P	951.569	0.2	0
OsJN00288	TIGR	9638.m02439	EP	HH	14479139	+	1	793	P	1875.519	0.222	0
OsJN00289	TIGR	9638.m03927	HP	LH	22584343	+	2	221	P	1804.529	0.2	0
OsJN00290	TIGR	9638.m03154	HP	LH	18544773	+	2	333	P	4892.696	0.2	0.667
OsJN00291	TIGR	9638.m02466	PP	HH	14605972	+	2	1043	P	1809.589	0.2	0.125
OsJN00292	TIGR	9638.m02498	PP	HH	14738969	+	1	1092	P	1310.76	0.167	0
OsJN00293	TIGR	9638.m01958	PP	HH	11511814	+	3	1791	P	1641.783	0.3	0
OsJN00294	TIGR	9638.m02487	PP	HH	14698708	+	2	2724	P	1741.392	0.119	0
OsJN00295	TIGR	9638.m03053	PP	HH	17959022	+	3	2289	P	622.124	0.182	0
OsJN00296	TIGR	9638.m03848	PP	HH	22147068	+	10	4225	P	1441.344	0.158	0.086
OsJN00297	TIGR	9638.m03363	HP	LH	19606045	+	1	1218	P	978.379	0.148	0
OsJN00298	TIGR	9638.m03644	HP	LH	21023080	+	3	873	A	558.787	0.1	0
OsJN00299	TIGR	9638.m03106	PP	HH	18272776	+	14	3414	P	740.269	0.171	0
OsJN00300	TIGR	9638.m02794	HP	LH	16418497	+	1	714	A	0	0	0
OsJN00301	TIGR	9638.m02138	PP	HH	12586124	+	3	2231	P	1054.462	0.16	0.118
OsJN00302	TIGR	9638.m03281	HP	LH	19195802	+	2	1307	A	0	0	0.15
OsJN00303	TIGR	9638.m03001	HP	LH	17728617	+	2	527	P	2273.323	0.556	1
OsJN00304	TIGR	9638.m02215	EP	HH	12990033	+	13	3974	P	1224.919	0.169	0.036
OsJN00305	TIGR	9638.m03672	EP	HH	21176157	+	8	2337	P	1106.952	0.135	0
OsJN00306	TIGR	9638.m03220	HP	LH	18861148	+	3	968	A	725.065	0.25	0.182
OsJN00307	TIGR	9638.m03557	PP	HH	20505766	+	1	1122	P	748.949	0.167	0
OsJN00308	TIGR	9638.m03474	PP	HH	20097847	+	2	1134	P	2142.413	0.25	0
OsJN00309	TIGR	9638.m01937	PP	HH	11376074	+	2	3280	A	0	0	0
OsJN00310	TIGR	9638.m03452	PP	HH	20014548	+	2	4457	A	0	0	0
OsJN00311	TIGR	9638.m03081	PP	HH	18139454	+	2	1486	P	1162.474	0.421	0
OsJN00312	TIGR	9638.m02893	HP	LH	16866171	+	2	606	A	0	0	0.125
OsJN00313	TIGR	9638.m03387	HP	LH	19718488	+	2	754	P	807.034	0.308	0.5
OsJN00314	TIGR	9638.m02774	HP	LH	16314301	+	1	435	P	718.861	0.3	0
OsJN00315	TIGR	9638.m02780	PP	HH	16339299	+	11	5767	P	1255.615	0.191	0.051
OsJN00316	TIGR	9638.m02151	HP	LH	12656083	+	2	1058	P	1493.627	0.364	0.111
OsJN00317	TIGR	9638.m02175	HP	LH	12785109	+	2	714	P	1201.286	0.25	0.222
OsJN00318	TIGR	9638.m03501	EP	LH	20211569	+	1	1123	P	2349.592	0.208	0
OsJN00319	TIGR	9638.m03228	HP	LH	18901641	+	3	1215	P	821.998	0.167	0.111
OsJN00320	TIGR	9638.m03371	HP	LH	19642420	+	5	5296	P	1569.079	0.5	0.159
OsJN00321	TIGR	9638.m03561	PP	HH	20517099	+	3	1204	A	451.529	0.1	0
OsJN00322	TIGR	9638.m03727	HP	LH	21496690	+	2	3232	P	1464.294	0.444	0.031
OsJN00323	TIGR	9638.m02327	HP	LH	13810380	+	1	405	A	0	0	0
OsJN00324	TIGR	9638.m03353	PP	HH	19558031	+	22	9065	P	1568.5	0.141	0.041
OsJN00325	TIGR	9638.m03298	PP	HH	19301565	+	2	2470	P	855.941	0.171	0
OsJN00326	TIGR	9638.m03250	EP	LH	19022229	+	7	3426	P	1124.877	0.129	0.023
OsJN00327	TIGR	9638.m03034	HP	LH	17884738	+	1	225	P	1664.244	0.333	0
OsJN00328	TIGR	9638.m03828	HP	LH	22046212	+	2	1050	P	1168.12	0.222	1
OsJN00329	TIGR	9638.m02055	EP	LH	12071782	+	1	447	P	746.219	0.286	0
OsJN00330	TIGR	9638.m02972	PP	HH	17479566	+	2	2784	P	1570.549	0.093	0.143
OsJN00331	TIGR	9638.m03047	PP	HH	17933446	+	13	3887	P	1323.369	0.125	0.034

OsJN00332	TIGR	9638.m03797	PP	LH	21904501	+	18	7414	P	1249.439	0.14	0.033
OsJN00333	TIGR	9638.m02120	HP	LH	12478954	+	3	495	P	798.301	0.2	0
OsJN00334	TIGR	9638.m02388	HP	LH	14201311	+	3	1025	P	777.296	0.5	0.2
OsJN00335	TIGR	9638.m03894	EP	HH	22436020	+	3	1946	P	1260.391	0.412	0.038
OsJN00336	TIGR	9638.m03255	PP	HH	19038471	+	20	7450	P	2052.661	0.136	0.031
OsJN00337	TIGR	9638.m03166	EP	HH	18591810	+	5	2906	P	926.898	0.208	0.091
OsJN00338	TIGR	9638.m02941	HP	LH	17219556	+	3	666	P	2399.198	0.538	0.333
OsJN00339	TIGR	9638.m03297	EP	HH	19298102	+	2	2749	P	1262.551	0.082	0.167
OsJN00340	TIGR	9638.m03150	EP	HH	18527251	+	12	5135	P	1492.567	0.143	0.014
OsJN00341	TIGR	9638.m03835	EP	LH	22072271	+	2	1320	P	1785.684	0.333	0
OsJN00342	TIGR	9638.m03588	HP	LH	20683068	+	2	746	P	748.715	0.5	0.125
OsJN00343	TIGR	9638.m03471	PP	HH	20089577	+	2	1089	P	977.969	0.261	0
OsJN00344	TIGR	9638.m03922	PP	HH	22559512	+	2	3471	P	1796.806	0.187	0
OsJN00345	TIGR	9638.m02445	PP	HH	14502693	+	1	1149	P	2560.544	0.077	0
OsJN00346	TIGR	9638.m02945	PP	HH	17238493	+	15	5281	P	1124.082	0.136	0
OsJN00347	TIGR	9638.m02195	HP	LH	12891278	+	3	1136	A	390.568	0.222	0
OsJN00348	TIGR	9638.m02884	PP	LH	16824342	+	1	615	P	2090.05	0.5	0
OsJN00349	TIGR	9638.m03761	HP	LH	21683918	+	2	735	P	2180.711	0.154	0.333
OsJN00350	TIGR	9638.m03841	HP	LH	22103688	+	5	1846	A	395.93	0.273	0.087
OsJN00351	TIGR	9638.m03203	HP	LH	18762353	+	2	755	P	1474.793	0.4	0
OsJN00352	TIGR	9638.m03107	PP	HH	18283948	+	10	5673	P	1439.763	0.197	0.05
OsJN00353	TIGR	9638.m02160	PP	HH	12697135	+	4	6659	P	1878.874	0.25	0
OsJN00354	TIGR	9638.m02961	PP	HH	17357482	+	6	2241	P	978.579	0.167	0.135
OsJN00355	TIGR	9638.m02899	PP	HH	16914879	+	3	5209	P	1195.712	0.063	0.063
OsJN00356	TIGR	9638.m01944	PP	HH	11410611	+	4	4691	P	1220.789	0.143	0.25
OsJN00357	TIGR	9638.m02472	HP	LH	14631751	+	3	3142	P	1280.369	0.643	0.19
OsJN00358	TIGR	9638.m03133	PP	HH	18434853	+	10	7878	P	1424.618	0.068	0.048
OsJN00359	TIGR	9638.m02291	HP	LH	13535647	+	2	605	A	476.696	0.143	0
OsJN00360	TIGR	9638.m02860	HP	HH	16690577	+	2	578	A	0	0	0
OsJN00361	TIGR	9638.m02627	EP	HH	15410738	+	9	4193	P	2102.244	0.21	0.1
OsJN00362	TIGR	9638.m03496	PP	HH	20172978	+	2	609	P	1313.24	0.333	0
OsJN00363	TIGR	9638.m02590	EP	HH	15194948	+	2	777	P	1201.668	0.471	0
OsJN00364	TIGR	9638.m03049	HP	LH	17944349	+	3	1255	P	2146.819	0.524	0.333
OsJN00365	TIGR	9638.m03507	PP	HH	20241589	+	5	1918	P	1417.918	0.37	0.2
OsJN00366	TIGR	9638.m03485	PP	HH	20128212	+	1	732	P	1319.862	0.25	0
OsJN00367	TIGR	9638.m02039	HP	LH	11969697	+	3	1116	P	1888.87	0.286	0.4
OsJN00368	TIGR	9638.m02530	HP	LH	14875687	+	3	2188	A	0	0	0
OsJN00369	TIGR	9638.m03631	EP	HH	20962071	+	3	1836	P	1101.102	0.393	0
OsJN00370	TIGR	9638.m03447	PP	HH	19995068	+	2	1093	P	2411.633	0.273	0
OsJN00371	TIGR	9638.m03305	HP	LH	19326079	+	2	505	P	2254.728	0.273	1
OsJN00372	TIGR	9638.m02664	HP	LH	15630938	+	5	2158	A	0	0	0.091
OsJN00373	TIGR	9638.m03774	PP	HH	21751842	+	2	2353	P	1134.716	0.423	0.04
OsJN00374	TIGR	9638.m02343	HP	LH	13918545	+	2	906	P	1814.364	0.5	0.143
OsJN00375	TIGR	9638.m02014	HP	LH	11838604	+	1	708	P	2740.174	0.438	0
OsJN00376	TIGR	9638.m02950	PP	HH	17255394	+	14	7528	P	1498.587	0.079	0.04
OsJN00377	TIGR	9638.m03794	EP	HH	21885794	+	18	7135	P	2552.883	0.068	0.051
OsJN00378	TIGR	9638.m03654	HP	LH	21098657	+	1	315	P	2854.677	0.25	0
OsJN00379	TIGR	9638.m02631	PP	HH	15428066	+	14	7932	P	1238.35	0.041	0.061
OsJN00380	TIGR	9638.m03942	PP	HH	22643551	+	6	2150	P	2629.093	0.286	0.05
OsJN00381	TIGR	9638.m03567	EP	LH	20565954	+	6	6049	P	902.187	0.273	0.156
OsJN00382	TIGR	9638.m02119	PP	HH	12473384	+	15	5034	P	1637.829	0.061	0.119
OsJN00383	TIGR	9638.m02216	EP	HH	12990506	+	12	3501	P	721.811	0.123	0.05
OsJN00384	TIGR	9638.m03152	HP	LH	18537806	+	1	471	P	1098.713	0.364	0
OsJN00385	TIGR	9638.m03023	EP	HH	17815386	+	2	2317	A	610.231	0.059	0
OsJN00386	TIGR	9638.m02367	EP	LH	14058227	+	1	1213	P	813.234	0.296	0
OsJN00387	TIGR	9638.m03638	EP	HH	20990945	+	2	6111	P	580.255	0.111	0

OsJN00388	TIGR	9638.m03616	PP	HH	20865447	+	10	4717	P	1018.293	0.106	0.077
OsJN00389	TIGR	9638.m02867	PP	HH	16720974	+	5	2914	P	1740.177	0.19	0
OsJN00390	TIGR	9638.m02832	PP	HH	16570197	+	3	1552	P	1422.82	0.219	0
OsJN00391	TIGR	9638.m03753	PP	HH	21634016	+	9	4138	P	1152.701	0.098	0.033
OsJN00392	TIGR	9638.m02768	EP	LH	16266116	+	8	2145	P	1337.581	0.27	0
OsJN00393	TIGR	9638.m03226	PP	HH	18886165	+	3	7717	P	1391.956	0.059	0.03
OsJN00394	TIGR	9638.m02270	PP	LH	13360186	+	1	684	P	1951.847	0.214	0
OsJN00395	TIGR	9638.m03264	HP	LH	19094150	+	2	559	P	899.644	0.182	0
OsJN00396	TIGR	9638.m02077	HP	LH	12209307	+	2	1166	A	358.938	0.071	0
OsJN00397	TIGR	9638.m03199	HP	LH	18750752	+	2	607	P	1416.264	0.455	0
OsJN00398	TIGR	9638.m02677	HP	LH	15739029	+	2	2976	A	769.055	0.067	0.239
OsJN00399	TIGR	9638.m02263	HP	HH	13321377	+	6	5033	P	838.337	0.063	0.068
OsJN00400	TIGR	9638.m01959	EP	LH	11517713	+	5	2354	P	835.553	0.25	0.111
OsJN00401	TIGR	9638.m03041	PP	HH	17910573	+	2	2770	P	875.471	0.263	0.103
OsJN00402	TIGR	9638.m03146	PP	HH	18501353	+	5	1738	P	697.304	0.182	0.063
OsJN00403	TIGR	9638.m02597	HP	LH	15225719	+	1	657	P	2226.932	0.667	0
OsJN00404	TIGR	9638.m03336	HP	LH	19491378	+	2	473	A	396.444	0.2	0
OsJN00405	TIGR	9638.m02915	EP	HH	17044014	+	5	2940	P	789.381	0.185	0.056
OsJN00406	TIGR	9638.m03020	HP	LH	17804055	+	3	1629	P	1764.361	0.294	0.389
OsJN00407	TIGR	9638.m02920	HP	LH	17080296	+	3	1166	P	1487.413	0.182	0.308
OsJN00408	TIGR	9638.m03013	EP	LH	17784622	+	1	883	P	1602.885	0.278	0
OsJN00409	TIGR	9638.m01979	HP	LH	11628584	+	4	2438	A	1	0	0
OsJN00410	TIGR	9638.m02146	EP	HH	12620458	+	5	4320	P	1239.402	0.179	0.15
OsJN00411	TIGR	9638.m03171	PP	HH	18608015	+	2	2078	P	1242.133	0.114	0
OsJN00412	TIGR	9638.m02203	EP	HH	12935366	+	3	1507	P	1278.367	0.1	0
OsJN00413	TIGR	9638.m02639	HP	LH	15481604	+	7	5480	P	1797.454	0.176	0.06
OsJN00414	TIGR	9638.m03935	HP	LH	22621486	+	2	1618	P	1398.053	0.286	0.25
OsJN00415	TIGR	9638.m03737	HP	HH	21556289	+	2	1489	P	1199.267	0.188	0
OsJN00416	TIGR	9638.m03549	PP	HH	20468304	+	1	1401	P	1048.728	0.065	0
OsJN00417	TIGR	9638.m03082	EP	LH	18155568	+	3	1033	P	1320.724	0.105	0
OsJN00418	TIGR	9638.m02321	HP	LH	13780565	+	4	4363	P	1424.918	0.278	0.111
OsJN00419	TIGR	9638.m02953	EP	HH	17277775	+	32	14822	P	909.058	0.079	0.061
OsJN00420	TIGR	9638.m02503	PP	HH	14758818	+	3	1005	P	1249.88	0.143	0
OsJN00421	TIGR	9638.m03577	HP	LH	20652396	+	4	1887	P	1541.983	0.333	0.053
OsJN00422	TIGR	9638.m02912	HP	LH	17015576	+	2	3551	P	2288.836	0.5	0.051
OsJN00423	TIGR	9638.m02751	EP	HH	16163205	+	1	786	P	1404.919	0.111	0
OsJN00424	TIGR	9638.m03059	EP	LH	18003676	+	1	1458	P	1174.092	0.133	0
OsJN00425	TIGR	9638.m02946	PP	HH	17238493	+	15	5281	P	1124.082	0.138	0
OsJN00426	TIGR	9638.m02451	PP	HH	14525089	+	1	1125	P	810.787	0.125	0
OsJN00427	TIGR	9638.m03735	EP	HH	21543596	+	2	1914	P	1657.716	0.261	0.053
OsJN00428	TIGR	9638.m01951	PP	HH	11478101	+	4	3931	P	2059.421	0.283	0
OsJN00429	TIGR	9638.m03428	PP	HH	19934984	+	2	1045	P	2244.695	0.235	0
OsJN00430	TIGR	9638.m03415	EP	LH	19871328	+	1	1192	P	1917.403	0.192	0
OsJN00431	TIGR	9638.m02659	EP	LH	15605568	+	2	1083	P	1299.484	0.3	0
OsJN00432	TIGR	9638.m01936	HP	LH	11372859	+	1	204	A	0	0	0
OsJN00433	TIGR	9638.m03643	PP	HH	21017363	+	10	4888	P	670.12	0.179	0.108
OsJN00434	TIGR	9638.m03167	PP	HH	18595877	+	3	3786	P	1599.331	0.159	0.051
OsJN00435	TIGR	9638.m02667	HP	HH	15647789	+	5	1620	P	1230.46	0.13	0
OsJN00436	TIGR	9638.m02328	PP	HH	13816586	+	10	4008	P	1962.78	0.106	0.045
OsJN00437	TIGR	9638.m02528	HP	LH	14869044	+	3	1416	P	807.95	0.273	0.056
OsJN00438	TIGR	9638.m03304	PP	HH	19323896	+	2	1556	P	2856.336	0.147	0
OsJN00439	TIGR	9638.m03368	PP	HH	19627923	+	3	3258	P	1467.755	0.163	0.333
OsJN00440	TIGR	9638.m02582	HP	HH	15147736	+	2	391	A	0	0	0
OsJN00441	TIGR	9638.m02875	PP	HH	16761323	+	9	4527	P	1785.451	0.286	0
OsJN00442	TIGR	9638.m02904	HP	LH	16957009	+	2	577	A	446.303	0.083	0
OsJN00443	TIGR	9638.m03466	PP	HH	20071893	+	2	1288	A	562.284	0.154	0.5

OsJN00444	TIGR	9638.m03204	PP	HH	18775526	+	8	4988	P	1610.269	0.227	0.125
OsJN00445	TIGR	9638.m03490	PP	HH	20142995	+	1	1020	P	1035.761	0.182	0
OsJN00446	TIGR	9638.m03710	PP	HH	21456471	+	1	2238	P	1419.17	0.122	0
OsJN00447	TIGR	9638.m02458	HP	LH	14561093	+	1	591	A	0	0	0
OsJN00448	TIGR	9638.m02895	PP	HH	16873731	+	3	4584	P	1878.071	0.104	0.065
OsJN00449	TIGR	9638.m02973	HP	LH	17484596	+	2	481	A	496.903	0.286	0.5
OsJN00450	TIGR	9638.m02856	HP	HH	16674989	+	4	2783	P	1051.922	0.194	0.071
OsJN00451	TIGR	9638.m03112	PP	HH	18312675	+	8	6063	P	1106.343	0.145	0.081
OsJN00452	TIGR	9638.m02260	HP	LH	13286728	+	4	3468	P	1038.806	0.132	0.278
OsJN00453	TIGR	9638.m02578	PP	HH	15120629	+	4	3308	P	1903.548	0.341	0.129
OsJN00454	TIGR	9638.m02167	EP	LH	12762979	+	8	3711	P	927.286	0.083	0.035
OsJN00455	TIGR	9638.m03035	PP	HH	17885670	+	8	3931	P	2081.587	0.281	0.096
OsJN00456	TIGR	9638.m03459	PP	HH	20048273	+	2	1153	P	2455.34	0.167	0
OsJN00457	TIGR	9638.m02070	HP	LH	12165193	+	4	2314	A	582.546	0.091	0.121
OsJN00458	TIGR	9638.m03187	HP	LH	18688177	+	2	596	P	1633.585	0.125	0.333
OsJN00459	TIGR	9638.m01946	PP	HH	11431753	+	13	6795	P	1401.696	0.115	0.09
OsJN00460	TIGR	9638.m02006	PP	LH	11801356	+	4	1937	P	718.589	0.103	0
OsJN00461	TIGR	9638.m03021	HP	LH	17808113	+	2	2369	P	1178.032	0.25	0
OsJN00462	TIGR	9638.m03011	HP	LH	17780294	+	2	348	A	408.166	0.375	0
OsJN00463	TIGR	9638.m02739	EP	LH	16091572	+	1	793	P	3578.555	0.556	0
OsJN00464	TIGR	9638.m02622	PP	HH	15367804	+	14	7887	P	1659.032	0.14	0.095
OsJN00465	TIGR	9638.m03136	PP	HH	18469547	+	8	4741	P	1244.703	0.186	0.089
OsJN00466	TIGR	9638.m03317	PP	HH	19378463	+	1	1871	P	1464.117	0.098	0
OsJN00467	TIGR	9638.m02642	HP	LH	15497292	+	1	243	P	2682.745	0.333	0
OsJN00468	TIGR	9638.m03454	PP	HH	20025377	+	2	1223	P	2580.957	0.222	0.4
OsJN00469	TIGR	9638.m02571	HP	LH	15075500	+	6	2666	P	1569.84	0.208	0.1
OsJN00470	TIGR	9638.m03398	PP	HH	19778517	+	10	3557	P	1219.896	0.205	0.054
OsJN00471	TIGR	9638.m02754	HP	LH	16173320	+	2	729	P	2595.899	0.333	0
OsJN00472	TIGR	9638.m02229	HP	LH	13060123	+	2	802	P	2171.501	0.5	0.111
OsJN00473	TIGR	9638.m03309	PP	HH	19344228	+	2	1636	P	1734.349	0.212	0
OsJN00474	TIGR	9638.m02238	PP	LH	13095218	+	3	1980	P	2246.016	0.143	0
OsJN00475	TIGR	9638.m02404	HP	LH	14272187	+	1	261	P	4050.959	0.167	0
OsJN00476	TIGR	9638.m02905	PP	LH	16970599	+	7	3415	P	1095.031	0.321	0.071
OsJN00477	TIGR	9638.m02341	HP	LH	13901594	+	8	2705	P	1127.127	0.071	0
OsJN00478	TIGR	9638.m03231	HP	LH	18907870	+	2	932	P	1874.533	0.364	0
OsJN00479	TIGR	9638.m03095	PP	HH	18206848	+	13	3774	P	2096.467	0.14	0
OsJN00480	TIGR	9638.m03614	EP	HH	20847035	+	25	9181	P	1280.174	0.152	0.042
OsJN00481	TIGR	9638.m03536	PP	HH	20397196	+	3	2198	P	1591.422	0.25	0.071
OsJN00482	TIGR	9638.m03008	PP	HH	17759374	+	5	1920	P	1316.151	0.071	0.071
OsJN00483	TIGR	9638.m03128	PP	HH	18399359	+	4	3648	P	1384.243	0.175	0.053
OsJN00484	TIGR	9638.m02586	EP	HH	15172598	+	16	10015	P	1444.114	0.11	0.061
OsJN00485	TIGR	9638.m03130	PP	HH	18416103	+	6	3307	P	2140.113	0.229	0.086
OsJN00486	TIGR	9638.m03508	PP	HH	20253253	+	7	3812	P	1422.516	0.167	0.071
OsJN00487	TIGR	9638.m03337	PP	HH	19494853	+	8	4156	P	1013.231	0.135	0
OsJN00488	TIGR	9638.m03649	HP	HH	21067111	+	11	10118	P	1014.533	0.049	0.04
OsJN00489	TIGR	9638.m02251	PP	HH	13208496	+	6	2572	P	1681.572	0.045	0.029
OsJN00490	TIGR	9638.m03157	EP	HH	18555944	+	2	2497	P	1054.949	0.036	0
OsJN00491	TIGR	9638.m02198	HP	LH	12909854	+	6	3053	P	1806.328	0.233	0.214
OsJN00492	TIGR	9638.m02977	EP	HH	17531749	+	22	7729	P	1214.664	0.162	0.033
OsJN00493	TIGR	9638.m02147	EP	HH	12628668	+	4	1895	P	1281.745	0.2	0
OsJN00494	TIGR	9638.m03664	EP	HH	21149095	+	4	2973	P	1436.859	0.444	0.065
OsJN00495	TIGR	9638.m03275	HP	LH	19165672	+	2	467	A	340.536	0.125	0
OsJN00496	TIGR	9638.m03377	HP	LH	19670608	+	2	410	P	1569.737	0.375	0
OsJN00497	TIGR	9638.m03609	EP	HH	20825284	+	3	2423	P	2056.42	0.217	0.097
OsJN00498	TIGR	9638.m03581	PP	HH	20665555	+	2	737	P	987.474	0.25	0
OsJN00499	TIGR	9638.m02836	HP	LH	16586764	+	1	309	P	2115.106	0.429	0

OsJN00500	TIGR	9638.m02881	PP	HH	16811534	+	2	4617	P	1641.484	0.114	0
OsJN00501	TIGR	9638.m03825	PP	LH	22036406	+	5	2585	P	1277.286	0.281	0.056
OsJN00502	TIGR	9638.m03469	HP	LH	20083725	+	2	1135	P	2209.084	0.571	0
OsJN00503	TIGR	9638.m03476	PP	HH	20100824	+	2	831	P	3748.686	0.118	0
OsJN00504	TIGR	9638.m02191	HP	LH	12874126	+	1	1014	P	985.373	0.318	0
OsJN00505	TIGR	9638.m02672	PP	HH	15680070	+	6	4236	P	1623.061	0.467	0.097
OsJN00506	TIGR	9638.m03046	EP	LH	17932013	+	2	653	P	1434.256	0.769	0
OsJN00507	TIGR	9638.m03953	PP	HH	22683476	+	2	3134	P	1354.957	0.231	0.119
OsJN00508	TIGR	9638.m02350	PP	LH	13943937	+	3	1905	P	798.91	0.167	0.4
OsJN00509	TIGR	9638.m02797	PP	LH	16426176	+	4	2100	P	751.641	0.077	0.167
OsJN00510	TIGR	9638.m03172	PP	HH	18611293	+	2	1933	P	1761.727	0.146	0
OsJN00511	TIGR	9638.m03593	PP	HH	20716652	+	4	2497	P	755.121	0.182	0.033
OsJN00512	TIGR	9638.m02995	HP	LH	17700938	+	1	363	P	1183.81	0.333	0
OsJN00513	TIGR	9638.m03270	EP	HH	19118686	+	6	4573	P	1223.589	0.194	0.081
OsJN00514	TIGR	9638.m02857	PP	HH	16680032	+	4	2700	P	1851.363	0.278	0
OsJN00515	TIGR	9638.m03092	PP	HH	18196650	+	3	2497	P	2046.798	0.25	0.25
OsJN00516	TIGR	9638.m02211	HP	HH	12964831	+	4	1770	P	1834.481	0.229	0
OsJN00517	TIGR	9638.m02449	HP	LH	14517739	+	1	302	P	802.072	0.571	0
OsJN00518	TIGR	9638.m02827	HP	LH	16534030	+	3	3805	P	1162.219	0.267	0.097
OsJN00519	TIGR	9638.m03863	EP	HH	22251221	+	5	2442	P	955.15	0.222	0.111
OsJN00520	TIGR	9638.m03850	PP	HH	22166959	+	3	1613	A	521.071	0.083	0.083
OsJN00521	TIGR	9638.m02425	PP	HH	14373981	+	3	3115	P	1310.244	0.152	0
OsJN00522	TIGR	9638.m02520	HP	LH	14821481	+	2	546	A	0	0	1
OsJN00523	TIGR	9638.m02767	HP	LH	16263895	+	1	659	P	1402.915	0.4	0
OsJN00524	TIGR	9638.m03787	PP	HH	21805973	+	5	4930	P	849.239	0.452	0.091
OsJN00525	TIGR	9638.m02085	EP	LH	12249851	+	2	677	P	2079.971	0.222	0
OsJN00526	TIGR	9638.m02954	EP	HH	17293606	+	8	5363	P	1728.962	0.155	0.086
OsJN00527	TIGR	9638.m03189	HP	LH	18691349	+	5	2842	P	2003.194	0.357	0.106
OsJN00528	TIGR	9638.m02657	EP	LH	15591966	+	5	4145	P	1120.267	0.333	0.169
OsJN00529	TIGR	9638.m03857	PP	HH	22187851	+	5	4357	P	1343.566	0.208	0.043
OsJN00530	TIGR	9638.m02985	PP	HH	17640427	+	4	1288	A	0	0	0
OsJN00531	TIGR	9638.m02430	HP	LH	14419337	+	1	285	P	1686.868	0.429	0
OsJN00532	TIGR	9638.m02504	HP	LH	14761372	+	3	1727	P	1054.793	0.231	0.208
OsJN00533	TIGR	9638.m03135	PP	HH	18450783	+	11	6977	P	1492.385	0.075	0.064
OsJN00534	TIGR	9638.m03659	PP	HH	21115274	+	3	2401	P	1397.898	0.238	0.111
OsJN00535	TIGR	9638.m02874	EP	HH	16757466	+	5	3386	P	1906.305	0.323	0.023
OsJN00536	TIGR	9638.m03052	PP	HH	17955813	+	3	2958	P	2889.634	0.091	0.25
OsJN00537	TIGR	9638.m02666	EP	LH	15639172	+	2	4805	P	1614.902	0.222	0.078
OsJN00538	TIGR	9638.m02378	EP	LH	14116828	+	1	333	P	2273.364	0.5	0
OsJN00539	TIGR	9638.m03555	PP	HH	20501014	+	1	1260	A	620.604	0.107	0
OsJN00540	TIGR	9638.m03173	EP	HH	18628933	+	2	3626	P	1632.332	0.313	0.109
OsJN00541	TIGR	9638.m03745	PP	HH	21602429	+	11	3746	P	1743.976	0.196	0
OsJN00542	TIGR	9638.m03799	PP	HH	21921373	+	3	963	P	1123.541	0.176	0
OsJN00543	TIGR	9638.m03684	PP	HH	21246685	+	10	4826	P	2016.428	0.196	0.063
OsJN00544	TIGR	9638.m03360	EP	LH	19595450	+	1	1667	P	789.188	0.143	0
OsJN00545	TIGR	9638.m03607	PP	HH	20817251	+	4	1812	P	1160.797	0.167	0.063
OsJN00546	TIGR	9638.m03375	HP	LH	19661696	+	2	1077	P	1150.797	0.278	0
OsJN00547	TIGR	9638.m03416	PP	HH	19874260	+	4	2377	P	2125.447	0.163	0.333
OsJN00548	TIGR	9638.m03806	PP	LH	21950136	+	6	3418	P	1613.703	0.5	0.02
OsJN00549	TIGR	9638.m03808	EP	LH	21961422	+	6	2190	P	2324.016	0.179	0.1
OsJN00550	TIGR	9638.m01947	PP	HH	11443178	+	7	4735	P	1565.503	0.129	0.072
OsJN00551	TIGR	9638.m02716	HP	LH	15977471	+	3	1078	A	0	0	0.063
OsJN00552	TIGR	9638.m02600	EP	LH	15236861	+	3	1789	P	1030.007	0.143	0.182
OsJN00553	TIGR	9638.m01950	PP	HH	11478101	+	5	3931	P	2059.421	0.288	0
OsJN00554	TIGR	9638.m02118	EP	HH	12463798	+	15	6394	P	976.083	0.351	0.092
OsJN00555	TIGR	9638.m02756	PP	HH	16188023	+	15	4739	P	722.608	0.136	0.023

OsJN00556	TIGR	9638.m03399	PP	HH	19778496	+	11	3578	P	1125.713	0.231	0.053
OsJN00557	TIGR	9638.m02465	PP	HH	14601530	+	1	1735	P	1164.947	0.211	0
OsJN00558	TIGR	9638.m03449	PP	HH	20000066	+	7	6022	P	626.723	0.048	0.091
OsJN00559	TIGR	9638.m03754	PP	HH	21634016	+	10	4777	P	1128.555	0.1	0.029
OsJN00560	TIGR	9638.m02053	EP	HH	12064384	+	7	2729	A	575.119	0.067	0.136
OsJN00561	TIGR	9638.m03885	PP	HH	22388298	+	3	2138	P	1582.195	0.143	0.5
OsJN00562	TIGR	9638.m02358	PP	HH	13984363	+	7	5991	P	1944.323	0.093	0.107
OsJN00563	TIGR	9638.m03030	EP	HH	17856399	+	3	2570	P	1458.802	0.205	0
OsJN00564	TIGR	9638.m02307	PP	HH	13693092	+	3	2566	A	635.836	0.077	0.25
OsJN00565	TIGR	9638.m02783	HP	LH	16353393	+	1	579	A	471.682	0.083	0
OsJN00566	TIGR	9638.m03278	PP	HH	19174250	+	8	2745	P	1185.464	0.121	0.148
OsJN00567	TIGR	9638.m02491	PP	HH	14712957	+	1	1439	P	953.724	0.129	0
OsJN00568	TIGR	9638.m03164	EP	HH	18591732	+	4	2969	P	948.751	0.218	0.1
OsJN00569	TIGR	9638.m03563	EP	HH	20533305	+	1	2837	P	1912.087	0.217	0
OsJN00570	TIGR	9638.m02277	EP	HH	13417164	+	8	5705	P	1064.409	0.257	0.067
OsJN00571	TIGR	9638.m03529	PP	HH	20343347	+	2	1225	P	1636.083	0.222	0
OsJN00572	TIGR	9638.m02505	EP	HH	14764100	+	1	360	P	7013.119	0.125	0
OsJN00573	TIGR	9638.m02422	HP	LH	14359118	+	5	2759	P	2104.926	0.467	0.24
OsJN00574	TIGR	9638.m03725	PP	HH	21491206	+	1	1022	P	2102.649	0.333	0
OsJN00575	TIGR	9638.m03541	PP	HH	20433623	+	11	3866	P	2207.485	0.27	0.021
OsJN00576	TIGR	9638.m03936	PP	HH	22626055	+	2	2942	P	1914.334	0.34	1
OsJN00577	TIGR	9638.m03944	PP	HH	22653939	+	3	2705	P	1192.615	0.161	0
OsJN00578	TIGR	9638.m03864	EP	HH	22251221	+	4	2442	P	955.15	0.222	0.111
OsJN00579	TIGR	9638.m02914	HP	LH	17036326	+	2	1200	P	2540.685	0.3	0.267
OsJN00580	TIGR	9638.m03613	HP	HH	20837962	+	14	6752	P	1449.32	0.162	0.042
OsJN00581	TIGR	9638.m03535	PP	HH	20392770	+	3	1127	P	2750.705	0.227	0.333
OsJN00582	TIGR	9638.m02529	HP	LH	14873838	+	1	633	P	2669.286	0.333	0
OsJN00583	TIGR	9638.m02598	PP	HH	15229891	+	5	3390	P	1223.32	0.143	0.077
OsJN00584	TIGR	9638.m02623	PP	HH	15376948	+	1	2373	P	1550.53	0.176	0
OsJN00585	TIGR	9638.m03093	PP	HH	18200217	+	3	1373	P	1862.095	0.286	0.333
OsJN00586	TIGR	9638.m02956	EP	HH	17294492	+	8	4477	P	1452.563	0.119	0.026
OsJN00587	TIGR	9638.m02261	HP	LH	13302179	+	2	1274	P	2145.242	0.318	0.25
OsJN00588	TIGR	9638.m02955	EP	HH	17294492	+	8	4477	P	1452.563	0.121	0.025
OsJN00589	TIGR	9638.m03015	PP	HH	17790299	+	2	2935	A	581.479	0.053	0
OsJN00590	TIGR	9638.m03221	HP	LH	18863470	+	3	2319	P	2047.871	0.438	0.429
OsJN00591	TIGR	9638.m03314	HP	LH	19364693	+	1	891	P	1560.133	0.579	0
OsJN00592	TIGR	9638.m02235	PP	LH	13089286	+	2	623	P	2154.38	0.231	1
OsJN00593	TIGR	9638.m03603	EP	HH	20799032	+	8	3255	A	525.365	0.05	0.137
OsJN00594	TIGR	9638.m03629	EP	HH	20957085	+	1	605	P	2619.67	0.231	0
OsJN00595	TIGR	9638.m03755	EP	HH	21649709	+	3	1510	P	1783.612	0.167	0
OsJN00596	TIGR	9638.m02204	HP	LH	12940884	+	2	538	P	4274.413	0.2	0.143
OsJN00597	TIGR	9638.m03812	EP	HH	21975701	+	11	4868	P	1261.125	0.082	0.03
OsJN00598	TIGR	9638.m03431	EP	HH	19959833	+	1	1743	P	9109.731	0.667	0
OsJN00599	TIGR	9638.m02316	EP	HH	13753003	+	11	5080	P	1654.297	0.205	0.029
OsJN00600	TIGR	9638.m03698	EP	HH	21355914	+	4	2454	P	2238.162	0.389	0.118
OsJN00601	TIGR	9638.m03514	HP	LH	20281772	+	3	1153	P	713.686	0.25	0
OsJN00602	TIGR	9638.m02695	HP	LH	15879267	+	2	441	P	1672.401	0.167	0
OsJN00603	TIGR	9638.m03846	PP	HH	22133150	+	6	4010	P	1481.763	0.167	0.018
OsJN00604	TIGR	9638.m02370	EP	LH	14089367	+	1	861	A	547.016	0.1	0
OsJN00605	TIGR	9638.m02788	HP	LH	16382728	+	4	3025	A	462.783	0.125	0.079
OsJN00606	TIGR	9638.m02389	HP	LH	14204318	+	2	1255	A	481.626	0.167	0
OsJN00607	TIGR	9638.m03600	HP	HH	20768875	+	5	2775	P	2163.642	0.167	0.095
OsJN00608	TIGR	9638.m03384	PP	HH	19704516	+	5	2088	P	976.939	0.321	0.176
OsJN00609	TIGR	9638.m02148	HP	LH	12634465	+	2	485	P	4832.683	0.25	0
OsJN00610	TIGR	9638.m02543	HP	LH	14950266	+	6	4191	P	1387.737	0.217	0.197
OsJN00611	TIGR	9638.m03655	HP	LH	21099255	+	1	333	P	2409.207	0.429	0

OsJN00612	TIGR	9638.m02182	HP	LH	12831496	+	2	934	P	1871.305	0.4	0.273
OsJN00613	TIGR	9638.m03762	PP	HH	21687010	+	2	2616	P	1167.505	0.273	0
OsJN00614	TIGR	9638.m03057	PP	HH	17985534	+	20	10254	A	522.074	0.044	0.046
OsJN00615	TIGR	9638.m02603	EP	HH	15251260	+	2	1282	P	2823.661	0.118	0
OsJN00616	TIGR	9638.m02018	HP	LH	11859179	+	2	434	A	0	0	0
OsJN00617	TIGR	9638.m03912	EP	HH	22529264	+	8	2871	P	1598.54	0.171	0
OsJN00618	TIGR	9638.m03243	PP	HH	18986775	+	4	6436	A	0	0	0.25
OsJN00619	TIGR	9638.m02360	EP	HH	13997797	+	6	2225	P	1856.165	0.1	0.143
OsJN00620	TIGR	9638.m02347	PP	LH	13934102	+	3	1978	P	1590.662	0.211	0.4
OsJN00621	TIGR	9638.m03242	PP	LH	18983523	+	2	578	A	1	0	0
OsJN00622	TIGR	9638.m03834	EP	HH	22065961	+	7	4359	P	1211.028	0.19	0
OsJN00623	TIGR	9638.m03574	PP	HH	20620717	+	6	6161	P	883.507	0.257	0.099
OsJN00624	TIGR	9638.m02265	HP	LH	13330746	+	3	744	P	2826.414	0.545	0
OsJN00625	TIGR	9638.m03772	PP	HH	21747675	+	10	2868	A	635.672	0.093	0.1
OsJN00626	TIGR	9638.m02976	PP	HH	17511181	+	8	7020	P	1088.022	0.1	0.053
OsJN00627	TIGR	9638.m03521	HP	LH	20320429	+	2	857	P	2252.444	0.333	0
OsJN00628	TIGR	9638.m03283	PP	LH	19200960	+	10	8249	P	1138.516	0.189	0.081
OsJN00629	TIGR	9638.m02168	HP	LH	12766978	+	2	681	P	983.88	0.5	0.375
OsJN00630	TIGR	9638.m03742	EP	LH	21580883	+	4	4343	P	1618.001	0.161	0
OsJN00631	TIGR	9638.m03689	EP	HH	21295990	+	5	3688	P	1529.089	0.348	0.121
OsJN00632	TIGR	9638.m03594	EP	HH	20721479	+	4	1979	P	955.986	0.167	0.091
OsJN00633	TIGR	9638.m01965	HP	HH	11551578	+	15	9261	P	1763.229	0.02	0.114
OsJN00634	TIGR	9638.m02947	HP	LH	17243540	+	4	1716	P	1386.524	0.222	0.083
OsJN00635	TIGR	9638.m02792	HP	LH	16400882	+	3	1273	A	720.821	0.083	0
OsJN00636	TIGR	9638.m03322	HP	LH	19399130	+	1	333	P	1672.796	0.375	0
OsJN00637	TIGR	9638.m03248	PP	HH	19009688	+	17	4243	P	825.165	0.22	0
OsJN00638	TIGR	9638.m03158	EP	LH	18562141	+	11	4833	P	1794.014	0.171	0.086
OsJN00639	TIGR	9638.m03147	PP	HH	18504985	+	5	1707	P	897.569	0.217	0.071
OsJN00640	TIGR	9638.m03773	PP	HH	21751859	+	1	1630	P	1499.152	0.167	0
OsJN00641	TIGR	9638.m02210	EP	LH	12961446	+	1	447	P	1604.972	0.273	0
OsJN00642	TIGR	9638.m03397	PP	HH	19778496	+	11	3578	P	1125.713	0.22	0.056
OsJN00643	TIGR	9638.m02755	EP	HH	16174854	+	1	2289	P	1323.086	0.26	0
OsJN00644	TIGR	9638.m03747	PP	HH	21614195	+	5	3086	P	774.827	0.237	0
OsJN00645	TIGR	9638.m03100	PP	HH	18232359	+	16	8022	P	1396.035	0.222	0.056
OsJN00646	TIGR	9638.m03789	EP	HH	21817897	+	5	2990	P	1304.248	0.212	0.063
OsJN00647	TIGR	9638.m03031	PP	HH	17859541	+	23	12266	P	1462.538	0.073	0.045
OsJN00648	TIGR	9638.m03071	EP	HH	18091149	+	7	3508	A	1	0	0
OsJN00649	TIGR	9638.m02456	PP	LH	14546751	+	2	1746	P	1502.636	0.192	0
OsJN00650	TIGR	9638.m02035	HP	LH	11944057	+	1	792	P	2539.71	0.667	0
OsJN00651	TIGR	9638.m03318	PP	HH	19381213	+	1	4149	P	1163.159	0.165	0
OsJN00652	TIGR	9638.m02593	HP	LH	15202287	+	2	615	P	1898.465	0.077	1
OsJN00653	TIGR	9638.m02606	PP	LH	15273840	+	2	896	P	795.196	0.059	0
OsJN00654	TIGR	9638.m03646	HP	LH	21042460	+	5	1871	P	1277.718	0.176	0.04
OsJN00655	TIGR	9638.m03280	PP	HH	19184402	+	13	4270	A	642.646	0.073	0.02
OsJN00656	TIGR	9638.m02910	EP	LH	16990884	+	1	585	P	613.868	1	0
OsJN00657	TIGR	9638.m03843	PP	LH	22125025	+	1	888	P	2542.945	0.3	0
OsJN00658	TIGR	9638.m02492	PP	HH	14717524	+	1	1110	P	1417.956	0.13	0
OsJN00659	TIGR	9638.m03217	PP	HH	18847301	+	1	1524	P	1767.924	0.212	0
OsJN00660	TIGR	9638.m03911	PP	HH	22522873	+	11	5943	P	1185.926	0.185	0.068
OsJN00661	TIGR	9638.m02833	EP	HH	16573422	+	2	1147	P	1924.371	0.136	0
OsJN00662	TIGR	9638.m01962	PP	LH	11537618	+	2	1415	P	1507.477	0.192	0
OsJN00663	TIGR	9638.m03702	HP	LH	21376179	+	2	618	P	1208.611	0.333	0
OsJN00664	TIGR	9638.m03546	PP	HH	20458197	+	1	1194	P	1238.48	0.038	0
OsJN00665	TIGR	9638.m03413	EP	LH	19858351	+	3	3824	P	1623.084	0.333	0.061
OsJN00666	TIGR	9638.m02557	HP	LH	15020618	+	1	486	P	1023.519	0.083	0
OsJN00667	TIGR	9638.m02453	PP	HH	14535360	+	1	858	P	1815.424	0.15	0

OsJN00668	TIGR	9638.m02157	PP	HH	12677679	+	4	2601	A	412.139	0.047	0
OsJN00669	TIGR	9638.m02444	HP	LH	14501230	+	1	453	P	1953.249	0.6	0
OsJN00670	TIGR	9638.m03165	EP	HH	18591732	+	5	2983	P	948.751	0.222	0.091
OsJN00671	TIGR	9638.m03856	PP	HH	22187867	+	3	3133	P	1393.904	0.217	0.045
OsJN00672	TIGR	9638.m02236	EP	LH	13090440	+	1	1111	P	2316.923	0.12	0
OsJN00673	TIGR	9638.m02829	EP	HH	16547323	+	18	5380	P	1304.225	0.266	0.148
OsJN00674	TIGR	9638.m03579	EP	HH	20659905	+	2	362	P	1148.561	0.143	0
OsJN00675	TIGR	9638.m02828	PP	HH	16539314	+	11	5835	P	1439.222	0.172	0.088
OsJN00676	TIGR	9638.m03267	HP	LH	19109655	+	1	804	P	2266.679	0.235	0
OsJN00677	TIGR	9638.m02986	HP	LH	17644150	+	1	465	P	1694.216	0.182	0
OsJN00678	TIGR	9638.m03532	PP	HH	20383489	+	3	5793	P	1273.85	0.196	0.032
OsJN00679	TIGR	9638.m03325	HP	LH	19411255	+	2	675	P	1024.05	0.231	0
OsJN00680	TIGR	9638.m02411	EP	HH	14295119	+	11	4410	P	1612.756	0.216	0.029
OsJN00681	TIGR	9638.m02675	PP	HH	15700522	+	7	6675	P	1656.616	0.212	0.078
OsJN00682	TIGR	9638.m03312	PP	HH	19355847	+	1	1899	P	933.286	0.19	0
OsJN00683	TIGR	9638.m02584	EP	HH	15158748	+	5	1852	P	1517.413	0.091	0.143
OsJN00684	TIGR	9638.m01980	HP	LH	11640013	+	4	1134	A	1	0	0
OsJN00685	TIGR	9638.m02473	PP	HH	14637814	+	2	1602	P	784.381	0.182	0
OsJN00686	TIGR	9638.m02770	EP	HH	16274662	+	14	4268	P	1257.227	0.218	0
OsJN00687	TIGR	9638.m02250	PP	HH	13205607	+	2	1385	P	1799.971	0.273	0
OsJN00688	TIGR	9638.m02734	HP	LH	16061969	+	5	3704	P	1963.417	0.143	0.024
OsJN00689	TIGR	9638.m02480	PP	HH	14670838	+	2	1487	P	1085.964	0.263	0
OsJN00690	TIGR	9638.m03681	HP	HH	21218868	+	1	1392	P	1231.947	0.207	0
OsJN00691	TIGR	9638.m03554	PP	HH	20494441	+	1	1191	P	665.189	0.154	0
OsJN00692	TIGR	9638.m02668	HP	LH	15659009	+	1	408	P	1167.618	0.444	0
OsJN00693	TIGR	9638.m03599	PP	HH	20750648	+	5	2963	P	950.353	0.244	0.292
OsJN00694	TIGR	9638.m03027	EP	HH	17842063	+	12	9012	P	1653.853	0.181	0.075
OsJN00695	TIGR	9638.m02144	HP	LH	12608547	+	3	750	A	0	0	0.2
OsJN00696	TIGR	9638.m02374	EP	LH	14101813	+	1	867	A	726.35	0.053	0
OsJN00697	TIGR	9638.m03335	HP	LH	19488726	+	2	448	P	1169.704	0.3	0
OsJN00698	TIGR	9638.m03882	PP	HH	22354370	+	1	2496	P	1528.029	0.37	0
OsJN00699	TIGR	9638.m03320	PP	HH	19391837	+	4	2074	P	723.06	0.261	0.043
OsJN00700	TIGR	9638.m03110	EP	LH	18306846	+	6	4198	P	3009.886	0.118	0.075
OsJN00701	TIGR	9638.m01952	HP	HH	11485530	+	1	1005	P	2029.196	0.273	0
OsJN00702	TIGR	9638.m02800	EP	LH	16437643	+	7	5045	P	2023.356	0.125	0.05
OsJN00703	TIGR	9638.m03176	PP	HH	18645579	+	2	2064	P	2324.044	0.233	0
OsJN00704	TIGR	9638.m02628	HP	HH	15417555	+	1	1482	P	1772.661	0.281	0
OsJN00705	TIGR	9638.m02394	EP	HH	14230795	+	7	5084	P	1661.293	0.111	0.072
OsJN00706	TIGR	9638.m03400	PP	HH	19782538	+	12	4714	P	2705.945	0.118	0.095
OsJN00707	TIGR	9638.m02231	HP	LH	13063917	+	2	846	P	1414.966	0.077	0
OsJN00708	TIGR	9638.m02545	PP	HH	14962074	+	3	3342	A	557.794	0.095	0.083
OsJN00709	TIGR	9638.m03919	PP	HH	22541751	+	14	6049	P	871.987	0.143	0.04
OsJN00710	TIGR	9638.m02765	PP	HH	16250018	+	9	4440	P	1332.952	0.176	0.048
OsJN00711	TIGR	9638.m03564	PP	HH	20545367	+	6	4308	P	1148.971	0.242	0.071
OsJN00712	TIGR	9638.m03276	PP	HH	19168601	+	3	2125	P	1571.284	0.2	0.125
OsJN00713	TIGR	9638.m03498	PP	HH	20178200	+	9	7944	P	1341.972	0.157	0
OsJN00714	TIGR	9638.m02931	HP	LH	17155345	+	5	2209	P	800.909	0.105	0.107
OsJN00715	TIGR	9638.m03302	HP	LH	19320427	+	2	522	A	0	0	0
OsJN00716	TIGR	9638.m02484	HP	LH	14684371	+	6	5507	P	1259.346	0.138	0.043
OsJN00717	TIGR	9638.m03586	HP	LH	20673862	+	3	2325	P	1139.01	0.1	0.097
OsJN00718	TIGR	9638.m03777	EP	HH	21765659	+	2	2036	P	2331.203	0.318	0.136
OsJN00719	TIGR	9638.m03867	EP	HH	22263524	+	1	926	P	1650.2	0.45	0
OsJN00720	TIGR	9638.m02615	EP	LH	15326424	+	4	1388	P	2730.995	0.417	0.5
OsJN00721	TIGR	9638.m03140	PP	HH	18485762	+	6	2277	P	1251.988	0.184	0.083
OsJN00722	TIGR	9638.m02156	PP	HH	12672085	+	6	4320	P	1464.24	0.238	0.2
OsJN00723	TIGR	9638.m02190	HP	LH	12872613	+	1	636	P	1143.112	0.5	0

OsJN00724	TIGR	9638.m03534	EP	LH	20390710	+	6	1573	P	1318.753	0.304	0
OsJN00725	TIGR	9638.m03749	PP	HH	21621336	+	3	3515	P	2732.257	0.178	0.161
OsJN00726	TIGR	9638.m03660	HP	LH	21120926	+	2	587	P	1451.984	0.444	0.5
OsJN00727	TIGR	9638.m03138	PP	HH	18480421	+	8	3849	P	1328.805	0.306	0.067
OsJN00728	TIGR	9638.m03556	HP	LH	20503113	+	2	584	P	792.842	0.125	0
OsJN00729	TIGR	9638.m02320	EP	LH	13770727	+	11	6880	P	1059.603	0.067	0.086
OsJN00730	TIGR	9638.m03506	EP	HH	20234051	+	1	1324	P	1136.07	0.276	0
OsJN00731	TIGR	9638.m03238	PP	HH	18946839	+	10	4822	P	2237.177	0.156	0.034
OsJN00732	TIGR	9638.m02790	PP	HH	16392102	+	1	1976	P	1598.412	0.163	0
OsJN00733	TIGR	9638.m03195	PP	LH	18725956	+	6	2424	P	2471.132	0.22	0.077
OsJN00734	TIGR	9638.m03959	PP	HH	14140638	+	6	1639	A	472.286	0.167	0
OsJN00735	TIGR	9638.m01988	HP	HH	11719848	+	1	474	A	675.421	0.091	0
OsJN00736	TIGR	9638.m02228	HP	HH	13057885	+	2	1351	P	1448.016	0.235	0.077
OsJN00737	TIGR	9638.m02550	HP	LH	14991083	+	3	1973	P	1813.795	0.167	0.138
OsJN00738	TIGR	9638.m01942	HP	LH	11400168	+	1	231	P	5502.079	0.333	0
OsJN00739	TIGR	9638.m03193	PP	HH	18717653	+	16	4471	P	912.561	0.088	0.05
OsJN00740	TIGR	9638.m03464	HP	LH	20065717	+	2	720	P	1612.363	0.364	0
OsJN00741	TIGR	9638.m03591	EP	HH	20702763	+	3	1912	P	745.105	0.125	0
OsJN00742	TIGR	9638.m02943	EP	HH	17225785	+	3	3042	P	1337.156	0.222	0.033
OsJN00743	TIGR	9638.m03478	PP	HH	20104549	+	2	797	P	2347.977	0.235	0
OsJN00744	TIGR	9638.m02576	EP	LH	15102619	+	17	7246	P	860.491	0.119	0.061
OsJN00745	TIGR	9638.m01949	PP	HH	11476961	+	6	5071	P	2280.672	0.271	0.1
OsJN00746	TIGR	9638.m02241	HP	LH	13121828	+	1	411	P	1054.35	0.2	0
OsJN00747	TIGR	9638.m03920	EP	HH	22549297	+	4	2800	P	842.071	0.167	0
OsJN00748	TIGR	9638.m02283	PP	HH	13443463	+	7	5302	P	1858.048	0.118	0.026
OsJN00749	TIGR	9638.m03621	PP	HH	20914356	+	3	2539	P	1714.102	0.34	0.125
OsJN00750	TIGR	9638.m03178	EP	LH	18651394	+	1	631	P	1212.242	0.4	0
OsJN00751	TIGR	9638.m03055	PP	LH	17971596	+	5	2041	P	2763.217	0.061	0
OsJN00752	TIGR	9638.m02970	EP	LH	17467004	+	3	3448	P	1044.922	0.222	0.018
OsJN00753	TIGR	9638.m03491	HP	LH	20144149	+	3	918	P	1967.948	0.364	0.222
OsJN00754	TIGR	9638.m02003	HP	LH	11784993	+	2	1290	P	1654.665	0.857	0
OsJN00755	TIGR	9638.m03718	PP	HH	21481000	+	1	3379	P	1188.726	0.147	0
OsJN00756	TIGR	9638.m02843	EP	LH	16615614	+	2	4107	P	1469.511	0.333	0.041
OsJN00757	TIGR	9638.m03683	EP	LH	21228218	+	3	5659	A	537.23	0.113	0.048
OsJN00758	TIGR	9638.m02319	HP	LH	13768738	+	3	1118	P	1814.736	0.105	0.167
OsJN00759	TIGR	9638.m01940	PP	HH	11392445	+	3	3212	A	599.501	0.085	0
OsJN00760	TIGR	9638.m03918	PP	HH	22541751	+	14	6048	P	871.987	0.145	0.039
OsJN00761	TIGR	9638.m02980	PP	HH	17585533	+	18	6235	P	1104.449	0.203	0.018
OsJN00762	TIGR	9638.m02634	EP	HH	15445539	+	7	3820	P	1476.367	0.355	0.075
OsJN00763	TIGR	9638.m03247	PP	HH	19009688	+	17	4243	P	825.165	0.22	0
OsJN00764	TIGR	9638.m03612	PP	HH	20835372	+	2	1119	P	1292.634	0.25	0
OsJN00765	TIGR	9638.m02683	HP	LH	15790108	+	1	150	P	6217.682	0.25	0
OsJN00766	TIGR	9638.m03800	EP	HH	21922739	+	2	2109	P	1780.239	0.237	0
OsJN00767	TIGR	9638.m02177	HP	LH	12802635	+	5	2355	A	333.74	0.083	0.154
OsJN00768	TIGR	9638.m03222	HP	LH	18867689	+	3	3087	P	1540.614	0.368	0.067
OsJN00769	TIGR	9638.m03645	EP	LH	21027319	+	12	10739	P	1248.637	0.173	0.051
OsJN00770	TIGR	9638.m01963	PP	LH	11537777	+	1	1256	P	1507.477	0.192	0
OsJN00771	TIGR	9638.m03162	PP	LH	18583351	+	5	3362	P	1745.805	0.516	0.049
OsJN00772	TIGR	9638.m03590	EP	HH	20696942	+	9	4812	P	2497.71	0.143	0.1
OsJN00773	TIGR	9638.m02507	PP	HH	14767859	+	1	1107	P	761.925	0.16	0
OsJN00774	TIGR	9638.m03531	PP	HH	20360291	+	8	10409	P	1149.322	0.156	0.061
OsJN00775	TIGR	9638.m03778	PP	HH	21771623	+	6	3441	P	1557.078	0.175	0.057
OsJN00776	TIGR	9638.m02349	EP	LH	13941015	+	3	1718	P	1033.602	0.167	0
OsJN00777	TIGR	9638.m02365	EP	HH	14042867	+	18	5411	P	1363	0.078	0.024
OsJN00778	TIGR	9638.m02798	PP	HH	16428528	+	2	2598	A	551.166	0.063	0
OsJN00779	TIGR	9638.m03334	HP	LH	19471869	+	4	1855	P	1785.715	0.375	0.28

OsJN00780	TIGR	9638.m02872	PP	HH	16746922	+	4	2399	P	1030.675	0.2	0.087
OsJN00781	TIGR	9638.m03467	HP	LH	20076865	+	2	1135	P	4178.176	0.143	0
OsJN00782	TIGR	9638.m02158	PP	HH	12689391	+	9	5255	P	1148.823	0.141	0
OsJN00783	TIGR	9638.m03731	HP	LH	21517066	+	2	975	P	1114.478	0.19	0
OsJN00784	TIGR	9638.m03553	PP	HH	20490880	+	1	1194	A	407.533	0.12	0
OsJN00785	TIGR	9638.m02359	EP	HH	13990529	+	4	3486	P	770.099	0.129	0.133
OsJN00786	TIGR	9638.m03028	EP	HH	17842063	+	12	9012	P	1653.853	0.181	0.075
OsJN00787	TIGR	9638.m03060	PP	HH	18030161	+	23	8710	P	1370.235	0.071	0.011
OsJN00788	TIGR	9638.m03216	EP	LH	18843474	+	4	1930	P	1487.005	0.162	0.5
OsJN00789	TIGR	9638.m02143	PP	HH	12602049	+	5	3668	P	1282.863	0.167	0.043
OsJN00790	TIGR	9638.m03101	HP	LH	18244621	+	1	492	P	2519.242	0.364	0
OsJN00791	TIGR	9638.m02750	PP	HH	16151756	+	19	7745	P	988.967	0.183	0.037
OsJN00792	TIGR	9638.m03604	PP	HH	20805516	+	5	2952	P	1982.986	0.161	0.061
OsJN00793	TIGR	9638.m03111	PP	HH	18312675	+	7	6063	P	1106.343	0.128	0.094
OsJN00794	TIGR	9638.m02043	EP	HH	11983571	+	7	3561	P	789.108	0.057	0
OsJN00795	TIGR	9638.m02978	PP	HH	17551497	+	3	4686	P	1553.429	0.151	0.082
OsJN00796	TIGR	9638.m03323	PP	HH	19405522	+	18	5428	P	1123.277	0.197	0.018
OsJN00797	TIGR	9638.m02308	HP	LH	13698200	+	1	294	A	0	0	0
OsJN00798	TIGR	9638.m02464	HP	LH	14600051	+	1	354	P	1769.556	0.5	0
OsJN00799	TIGR	9638.m03232	HP	LH	18910409	+	2	859	P	1804.879	0.444	0
OsJN00800	TIGR	9638.m02282	HP	LH	13439332	+	2	386	P	2096.668	0.333	0
OsJN00801	TIGR	9638.m02813	PP	HH	16486526	+	3	1398	P	889.017	0.043	0
OsJN00802	TIGR	9638.m02923	PP	LH	17096560	+	6	6497	P	1470.711	0.433	0.132
OsJN00803	TIGR	9638.m03086	HP	LH	18163277	+	1	396	P	1193.008	0.444	0
OsJN00804	TIGR	9638.m03580	HP	LH	20662329	+	3	1818	A	651.164	0.071	0
OsJN00805	TIGR	9638.m02896	HP	LH	16887437	+	3	1434	P	1629.4	0.4	0.067
OsJN00806	TIGR	9638.m02117	HP	LH	12457796	+	2	2744	A	344.427	0.333	0.048
OsJN00807	TIGR	9638.m02806	PP	HH	16467094	+	2	999	A	0	0	0
OsJN00808	TIGR	9638.m03345	PP	HH	19522047	+	5	3420	P	1565.937	0.157	0.158
OsJN00809	TIGR	9638.m02981	PP	HH	17584810	+	19	6961	P	1409.167	0.229	0.045
OsJN00810	TIGR	9638.m01975	PP	LH	11606679	+	4	1885	A	1	0	0
OsJN00811	TIGR	9638.m03661	HP	LH	21126337	+	3	503	P	1312.851	0.1	0
OsJN00812	TIGR	9638.m03533	PP	HH	20383489	+	4	5793	P	1312.734	0.211	0.042
OsJN00813	TIGR	9638.m02008	HP	LH	11808390	+	2	352	P	919.631	0.333	0
OsJN00814	TIGR	9638.m02284	PP	HH	13451716	+	6	3385	P	1827.796	0.189	0.081
OsJN00815	TIGR	9638.m02663	EP	HH	15620544	+	14	9648	P	1399.901	0.125	0.079
OsJN00816	TIGR	9638.m03403	PP	HH	19804436	+	1	1620	P	2289.556	0.194	0
OsJN00817	TIGR	9638.m02373	HP	LH	14100369	+	1	270	P	1220.986	0.333	0
OsJN00818	TIGR	9638.m02379	EP	LH	14121097	+	1	318	P	2294.136	0.429	0
OsJN00819	TIGR	9638.m02647	EP	HH	15527131	+	1	1581	P	1068.805	0.059	0
OsJN00820	TIGR	9638.m03771	EP	LH	21741133	+	7	3117	P	922.676	0.063	0.056
OsJN00821	TIGR	9638.m02900	HP	HH	16922558	+	17	6899	A	0	0	0
OsJN00822	TIGR	9638.m02701	HP	HH	15912455	+	1	2376	P	1731.536	0.132	0
OsJN00823	TIGR	9638.m03465	HP	LH	20068845	+	3	1064	P	2052.09	0.4	0.6
OsJN00824	TIGR	9638.m03006	HP	LH	17746135	+	2	2040	P	2335.808	0.385	0.071
OsJN00825	TIGR	9638.m02771	PP	HH	16286890	+	26	13319	P	1175.396	0.115	0.072
OsJN00826	TIGR	9638.m03786	PP	HH	21806045	+	5	4858	P	849.239	0.483	0.091
OsJN00827	TIGR	9638.m03484	PP	HH	20126151	+	1	899	P	855.294	0.211	0
OsJN00828	TIGR	9638.m01933	HP	LH	11356350	+	2	1373	P	1170.953	0.444	0.143
OsJN00829	TIGR	9638.m03139	PP	HH	18485769	+	6	2267	P	1251.988	0.189	0.083
OsJN00830	TIGR	9638.m01943	HP	HH	11403810	+	2	1397	A	0	0	0.2
OsJN00831	TIGR	9638.m02822	HP	LH	16510747	+	1	330	P	1771.872	0.625	0
OsJN00832	TIGR	9638.m03194	PP	HH	18717653	+	19	5425	P	894.563	0.121	0.08
OsJN00833	TIGR	9638.m03367	PP	HH	19623474	+	2	2760	P	966.388	0.13	0
OsJN00834	TIGR	9638.m02090	PP	HH	12280268	+	1	375	A	425.589	0.111	0
OsJN00835	TIGR	9638.m03356	EP	LH	19584411	+	1	708	P	1235.08	0.188	0

OsJN00836	TIGR	9638.m03208	PP	HH	18802838	+	2	543	P	4616.65	0.167	0
OsJN00837	TIGR	9638.m02549	PP	HH	14985448	+	1	1188	P	772.68	0.148	0
OsJN00838	TIGR	9638.m03470	PP	HH	20086831	+	2	1087	P	866.413	0.227	0
OsJN00839	TIGR	9638.m02125	HP	HH	12502090	+	12	7627	P	1534.056	0.098	0.068
OsJN00840	TIGR	9638.m03129	PP	HH	18405580	+	13	8785	P	1856.06	0.193	0.019
OsJN00841	TIGR	9638.m02010	HP	LH	11816110	+	2	1537	P	819.988	0.087	0.222
OsJN00842	TIGR	9638.m03601	PP	HH	20790146	+	5	4397	P	824.75	0.313	0
OsJN00843	TIGR	9638.m03149	HP	HH	18515143	+	16	8927	P	1429.949	0.198	0.042
OsJN00844	TIGR	9638.m02585	EP	HH	15162907	+	3	934	P	1379.997	0.316	0.5
OsJN00845	TIGR	9638.m02489	HP	LH	14707257	+	4	1066	P	1364.175	0.462	0.3
OsJN00846	TIGR	9638.m03043	PP	HH	17918200	+	11	5948	P	1316.64	0.267	0.179
OsJN00847	TIGR	9638.m03155	HP	LH	18548197	+	2	1508	A	0	0	0.071
OsJN00848	TIGR	9638.m02619	HP	LH	15359150	+	1	456	A	595.578	0.1	0
OsJN00849	TIGR	9638.m01581	PP	HH	9158657	+	3	3853	P	1008.414	0.179	0.038
OsJN00850	TIGR	9638.m01116	HP	LH	6514280	+	1	339	P	901.843	0.571	0
OsJN00851	TIGR	9638.m00180	PP	HH	1067093	+	7	2038	P	1399.524	0.152	0
OsJN00852	TIGR	9638.m00428	EP	HH	2508118	+	1	483	A	448.866	0.091	0
OsJN00853	TIGR	9638.m01925	HP	LH	11318446	+	4	1546	A	587.158	0.19	0.077
OsJN00854	TIGR	9638.m01367	HP	LH	7873827	+	1	837	P	1045.017	0.333	0
OsJN00855	TIGR	9638.m00135	HP	LH	811250	+	1	294	A	0	0	0
OsJN00856	TIGR	9638.m01865	HP	LH	10935496	+	4	2728	P	1221.747	0.25	0.125
OsJN00857	TIGR	9638.m01750	HP	LH	10154816	+	3	1322	P	1384.837	0.1	0.111
OsJN00858	TIGR	9638.m01689	EP	HH	9789229	+	8	2357	P	1420.802	0.128	0
OsJN00859	TIGR	9638.m00950	HP	LH	5598743	+	3	2784	P	1065.146	0.174	0.139
OsJN00860	TIGR	9638.m01218	EP	HH	7126558	+	2	873	P	1476.657	0.474	0
OsJN00861	TIGR	9638.m01794	HP	LH	10499383	+	3	771	P	2347.369	0.615	0.5
OsJN00862	TIGR	9638.m00476	HP	LH	2799176	+	2	801	A	657.254	0.222	0.25
OsJN00863	TIGR	9638.m00942	EP	LH	5548251	+	1	489	P	747.088	0.5	0
OsJN00864	TIGR	9638.m01261	PP	HH	7342801	+	3	2386	P	862.98	0.102	0
OsJN00865	TIGR	9638.m00882	HP	LH	5167924	+	1	357	A	0	0	0
OsJN00866	TIGR	9638.m01466	PP	HH	8491565	+	3	2776	P	2113.182	0.176	0.16
OsJN00867	TIGR	9638.m01055	EP	LH	6141326	+	3	2263	A	412.904	0.133	0.03
OsJN00868	TIGR	9638.m01857	PP	HH	10894272	+	1	747	P	1197.869	0.188	0
OsJN00869	TIGR	9638.m00484	PP	HH	2869615	+	2	1016	A	428.589	0.071	0.333
OsJN00870	TIGR	9638.m01847	HP	LH	10838539	+	2	1524	A	396.763	0.105	0
OsJN00871	TIGR	9638.m01826	HP	LH	10666494	+	2	1174	A	0	0	0.083
OsJN00872	TIGR	9638.m01354	HP	LH	7820923	+	1	537	P	1338.698	0.25	0
OsJN00873	TIGR	9638.m00076	EP	HH	452711	+	3	3851	P	1211.305	0.343	0.063
OsJN00874	TIGR	9638.m01276	PP	LH	7400032	+	5	2064	P	1892.261	0.267	0
OsJN00875	TIGR	9638.m00748	EP	LH	4414319	+	1	566	P	1304.852	0.417	0
OsJN00876	TIGR	9638.m01327	HP	LH	7684510	+	1	444	P	1493.242	0.667	0
OsJN00877	TIGR	9638.m00522	EP	HH	3074743	+	3	6850	P	1314.456	0.077	0.034
OsJN00878	TIGR	9638.m01651	HP	LH	9575338	+	5	1258	P	1612.748	0.273	0
OsJN00879	TIGR	9638.m01091	PP	HH	6372361	+	2	1304	P	1509.727	0.071	0
OsJN00880	TIGR	9638.m01874	PP	HH	11005846	+	2	1941	P	1385.218	0.067	0
OsJN00881	TIGR	9638.m00786	HP	LH	4597267	+	4	1271	P	1873.525	0.048	0.143
OsJN00882	TIGR	9638.m00577	HP	HH	3387570	+	28	20374	P	1195.149	0.052	0.041
OsJN00883	TIGR	9638.m00755	EP	HH	4439981	+	9	5470	P	1008.591	0.135	0.145
OsJN00884	TIGR	9638.m00284	HP	LH	1688086	+	3	821	P	1548.89	0.273	0.286
OsJN00885	TIGR	9638.m01678	HP	LH	9726656	+	2	346	P	800.685	0.4	0
OsJN00886	TIGR	9638.m01160	HP	LH	6756040	+	6	2710	A	1	0	0
OsJN00887	TIGR	9638.m01761	HP	LH	10266360	+	3	814	A	0	0	0
OsJN00888	TIGR	9638.m01706	HP	HH	9877201	+	12	7805	P	1042.034	0.047	0.154
OsJN00889	TIGR	9638.m00993	PP	LH	5835489	+	4	4893	A	0	0	1
OsJN00890	TIGR	9638.m00907	HP	LH	5333705	+	4	1781	P	1305.06	0.1	0
OsJN00891	TIGR	9638.m01814	EP	HH	10584135	+	3	4021	A	548.806	0.056	0.063

OsJN00892	TIGR	9638.m01228	HP	LH	7188145	+	2	761	A	0	0	0
OsJN00893	TIGR	9638.m00085	HP	LH	512469	+	2	404	P	2359.782	0.2	0.25
OsJN00894	TIGR	9638.m01391	HP	HH	8037331	+	1	594	P	900.256	0.214	0
OsJN00895	TIGR	9638.m00982	EP	HH	5759864	+	5	4206	P	1460.593	0.354	0.073
OsJN00896	TIGR	9638.m01124	EP	HH	6560276	+	4	2837	A	638.507	0.143	0.103
OsJN00897	TIGR	9638.m01090	HP	LH	6366156	+	2	356	P	1058.588	0.667	1
OsJN00898	TIGR	9638.m01122	HP	LH	6547075	+	2	355	P	1250.809	0.333	0
OsJN00899	TIGR	9638.m00841	EP	LH	4890940	+	2	1136	P	1859.026	0.444	0.167
OsJN00900	TIGR	9638.m00840	EP	LH	4888241	+	4	1326	A	608.956	0.158	0.1
OsJN00901	TIGR	9638.m00235	HP	LH	1372952	+	2	382	P	1600.59	0.333	0.333
OsJN00902	TIGR	9638.m00959	PP	HH	5647708	+	2	6096	P	772.702	0.071	0
OsJN00903	TIGR	9638.m01518	HP	LH	8809498	+	2	711	P	2293.514	0.333	0.429
OsJN00904	TIGR	9638.m01198	PP	HH	7007809	+	4	5558	P	814.676	0.103	0
OsJN00905	TIGR	9638.m00012	PP	HH	79138	+	1	2652	P	1389.304	0.254	0
OsJN00906	TIGR	9638.m01089	HP	LH	6362154	+	3	1021	A	520.004	0.125	0.133
OsJN00907	TIGR	9638.m00270	HP	LH	1558041	+	1	249	P	886.101	0.4	0
OsJN00908	TIGR	9638.m00856	EP	LH	4977321	+	1	363	P	2087.051	0.556	0
OsJN00909	TIGR	9638.m01269	HP	LH	7378157	+	1	426	P	3572.307	0.4	0
OsJN00910	TIGR	9638.m01151	HP	LH	6709000	+	1	603	A	1	0	0
OsJN00911	TIGR	9638.m01045	HP	LH	6077783	+	1	207	P	1189.146	0.6	0
OsJN00912	TIGR	9638.m00263	EP	HH	1523609	+	1	888	P	1561.821	0.211	0
OsJN00913	TIGR	9638.m01579	EP	HH	9150653	+	3	1473	P	818.088	0.048	0
OsJN00914	TIGR	9638.m00262	HP	LH	1517101	+	3	3048	P	889.864	0.222	0.097
OsJN00915	TIGR	9638.m00008	PP	HH	51289	+	8	5851	P	790.928	0.125	0.066
OsJN00916	TIGR	9638.m01477	HP	LH	8559070	+	1	702	P	597.567	0.333	0
OsJN00917	TIGR	9638.m01315	HP	LH	7611234	+	8	3956	P	3065.795	0.083	0.183
OsJN00918	TIGR	9638.m01731	HP	LH	10065850	+	2	1120	A	409.831	0.6	0.05
OsJN00919	TIGR	9638.m00419	EP	HH	2476766	+	1	351	P	947.038	0.222	0
OsJN00920	TIGR	9638.m00383	PP	HH	2241637	+	7	5130	P	1185.558	0.135	0.017
OsJN00921	TIGR	9638.m01324	HP	LH	7659884	+	5	1921	P	981.059	0.111	0
OsJN00922	TIGR	9638.m01930	HP	LH	11341340	+	2	394	A	0	0	0
OsJN00923	TIGR	9638.m00249	PP	HH	1440098	+	1	1152	P	1564.37	0.13	0
OsJN00924	TIGR	9638.m01031	PP	HH	6026851	+	1	710	P	3883.772	0.625	0
OsJN00925	TIGR	9638.m01513	PP	HH	8777324	+	1	4575	P	923.703	0.5	0
OsJN00926	TIGR	9638.m01147	HP	LH	6687756	+	2	362	P	1182.168	0.286	1
OsJN00927	TIGR	9638.m00929	EP	HH	5477958	+	8	6923	P	1380.289	0.22	0.093
OsJN00928	TIGR	9638.m01838	EP	HH	10748176	+	8	3355	P	875.935	0.083	0
OsJN00929	TIGR	9638.m00839	HP	LH	4886178	+	3	1179	P	1172.422	0.5	0.1
OsJN00930	TIGR	9638.m00887	HP	LH	5215337	+	5	5293	A	426.685	0.067	0.113
OsJN00931	TIGR	9638.m00957	PP	HH	5636053	+	2	6111	A	365.84	0.053	0
OsJN00932	TIGR	9638.m01829	PP	HH	10682921	+	2	1509	P	915.392	0.16	0
OsJN00933	TIGR	9638.m01653	EP	HH	9585424	+	9	5559	P	1188.284	0.197	0.019
OsJN00934	TIGR	9638.m01432	HP	LH	8296323	+	3	2011	P	1317.555	0.094	0.125
OsJN00935	TIGR	9638.m00435	PP	LH	2571519	+	1	1296	P	693.244	0.207	0
OsJN00936	TIGR	9638.m01867	EP	LH	10956275	+	8	3790	P	1340.102	0.175	0
OsJN00937	TIGR	9638.m01179	PP	LH	6873649	+	2	2686	P	2305.7	0.375	0.25
OsJN00938	TIGR	9638.m00991	HP	LH	5821841	+	2	912	P	1048.818	0.4	0
OsJN00939	TIGR	9638.m01502	PP	LH	8704769	+	1	447	P	3267.699	0.5	0
OsJN00940	TIGR	9638.m01134	EP	HH	6603835	+	6	5114	A	615.211	0.08	0.035
OsJN00941	TIGR	9638.m00349	HP	HH	2062029	+	2	3199	P	1189.564	0.13	0.048
OsJN00942	TIGR	9638.m01572	HP	LH	9101423	+	2	1453	A	345.373	0.167	0
OsJN00943	TIGR	9638.m00734	HP	LH	4342392	+	2	803	P	3304.006	0.143	0.182
OsJN00944	TIGR	9638.m00773	HP	LH	4537834	+	7	3751	P	1199.957	0.244	0.333
OsJN00945	TIGR	9638.m01705	HP	LH	9872789	+	3	1765	P	1333.826	0.286	0.065
OsJN00946	TIGR	9638.m00896	HP	LH	5283647	+	2	2196	A	648.205	0.286	0.059
OsJN00947	TIGR	9638.m00478	PP	HH	2813310	+	2	1636	P	1228.88	0.292	0.25

OsJN00948	TIGR	9638.m00297	HP	LH	1752562	+	2	578	P	965.802	0.25	0
OsJN00949	TIGR	9638.m00239	EP	HH	1389875	+	1	1488	P	1626.448	0.182	0
OsJN00950	TIGR	9638.m01485	PP	HH	8590291	+	1	1176	A	569.182	0.115	0
OsJN00951	TIGR	9638.m00766	HP	LH	4499730	+	2	750	P	1571.794	0.333	0.375
OsJN00952	TIGR	9638.m00316	HP	LH	1869028	+	1	2364	P	1295.367	0.196	0
OsJN00953	TIGR	9638.m01622	HP	LH	9362289	+	4	3394	P	1664.353	0.333	0.226
OsJN00954	TIGR	9638.m01852	HP	LH	10868130	+	1	489	A	0	0	0
OsJN00955	TIGR	9638.m00642	HP	LH	3795354	+	3	1886	A	527.089	0.143	0.074
OsJN00956	TIGR	9638.m00108	HP	LH	647764	+	1	288	P	980.511	0.5	0
OsJN00957	TIGR	9638.m01800	HP	HH	10522496	+	1	1137	P	1657.75	0.36	0
OsJN00958	TIGR	9638.m01580	HP	LH	9155816	+	3	1716	P	1202.249	0.222	0.05
OsJN00959	TIGR	9638.m01238	HP	LH	7245363	+	2	279	A	576.015	0.4	0
OsJN00960	TIGR	9638.m01652	EP	HH	9585424	+	8	5559	P	1188.284	0.188	0.02
OsJN00961	TIGR	9638.m01556	HP	LH	8983535	+	2	870	A	0	0	0
OsJN00962	TIGR	9638.m00849	EP	LH	4934856	+	1	648	A	647.093	0.071	0
OsJN00963	TIGR	9638.m01380	PP	LH	7940006	+	2	3729	P	624.089	0.067	0
OsJN00964	TIGR	9638.m01760	HP	LH	10248874	+	3	760	P	1356.726	0.286	0
OsJN00965	TIGR	9638.m01568	HP	LH	9075444	+	3	886	A	0	0	0
OsJN00966	TIGR	9638.m00075	HP	LH	445414	+	2	509	A	0	0	0.167
OsJN00967	TIGR	9638.m00279	HP	LH	1662166	+	1	462	P	1473.294	0.182	0
OsJN00968	TIGR	9638.m00136	HP	LH	815020	+	2	242	P	2017.877	1	0
OsJN00969	TIGR	9638.m01067	HP	LH	6227346	+	2	2232	P	1362.34	0.3	0.114
OsJN00970	TIGR	9638.m00132	EP	HH	800396	+	1	954	A	628.43	0.143	0
OsJN00971	TIGR	9638.m00516	EP	HH	3045083	+	3	4612	P	1157.255	0.064	0
OsJN00972	TIGR	9638.m01841	HP	HH	10784084	+	2	2619	P	2648.693	0.396	0.111
OsJN00973	TIGR	9638.m00918	HP	HH	5387817	+	2	1020	P	831.431	0.222	0.071
OsJN00974	TIGR	9638.m00872	EP	HH	5069289	+	1	1749	P	1483.791	0.184	0
OsJN00975	TIGR	9638.m00583	HP	HH	3443306	+	3	1239	P	1280.481	0.154	0.308
OsJN00976	TIGR	9638.m00594	EP	HH	3511321	+	1	927	P	1077.13	0.286	0
OsJN00977	TIGR	9638.m01226	HP	LH	7175825	+	2	579	P	2163.539	0.778	0
OsJN00978	TIGR	9638.m01121	HP	LH	6539280	+	1	485	P	746.523	0.111	0
OsJN00979	TIGR	9638.m00507	HP	LH	2979517	+	2	403	P	2599.271	0.125	0
OsJN00980	TIGR	9638.m01389	HP	LH	8024736	+	1	462	P	811.51	0.091	0
OsJN00981	TIGR	9638.m00612	HP	LH	3612091	+	1	513	P	1904.896	0.455	0
OsJN00982	TIGR	9638.m00616	HP	LH	3625218	+	3	673	A	0	0	0
OsJN00983	TIGR	9638.m01769	HP	LH	10335089	+	2	611	P	17884.195	1	1
OsJN00984	TIGR	9638.m00970	HP	LH	5708278	+	3	2245	P	1181.445	0.25	0
OsJN00985	TIGR	9638.m00696	EP	HH	4134821	+	1	1801	P	1709.731	0.125	0
OsJN00986	TIGR	9638.m01303	HP	LH	7550527	+	1	396	P	908.823	0.625	0
OsJN00987	TIGR	9638.m01777	EP	HH	10371961	+	3	1419	P	6544.929	0.933	1
OsJN00988	TIGR	9638.m01715	HP	LH	9964860	+	2	770	P	1313.985	0.273	0
OsJN00989	TIGR	9638.m00381	HP	LH	2232037	+	2	635	P	2034.166	0.167	0
OsJN00990	TIGR	9638.m00933	EP	HH	5497938	+	4	2237	P	1363.933	0.353	0.2
OsJN00991	TIGR	9638.m01890	HP	LH	11089826	+	3	1227	P	1145.462	0.2	0
OsJN00992	TIGR	9638.m01512	PP	HH	8772643	+	1	780	A	0	0	0
OsJN00993	TIGR	9638.m01535	HP	LH	8901164	+	1	306	P	777.665	0.143	0
OsJN00994	TIGR	9638.m00665	EP	HH	3963275	+	3	4328	P	974.892	0.122	0.05
OsJN00995	TIGR	9638.m00400	EP	LH	2358806	+	3	2893	P	742.757	0.057	0.034
OsJN00996	TIGR	9638.m01423	HP	HH	8236407	+	10	3212	P	693.951	0.038	0.111
OsJN00997	TIGR	9638.m01770	EP	HH	10337305	+	1	1524	P	943.488	0.545	0
OsJN00998	TIGR	9638.m01342	HP	LH	7762764	+	2	798	P	1198.701	0.267	0
OsJN00999	TIGR	9638.m01123	HP	LH	6554022	+	1	203	A	508.505	0.2	0
OsJN01000	TIGR	9638.m00676	HP	LH	4030841	+	8	3215	P	1555.019	0.027	0
OsJN01001	TIGR	9638.m00165	HP	LH	982311	+	3	1287	P	1614.733	0.375	0.15
OsJN01002	TIGR	9638.m00983	EP	HH	5765812	+	2	1164	P	694.609	0.167	0
OsJN01003	TIGR	9638.m00350	EP	LH	2069273	+	3	1696	P	3042.072	0.6	0.111

OsJN01004	TIGR	9638.m00230	HP	LH	1343663	+	2	801	P	3745.75	0.267	0
OsJN01005	TIGR	9638.m00624	HP	LH	3710549	+	4	3717	P	1207.777	0.286	0.308
OsJN01006	TIGR	9638.m00268	PP	LH	1545550	+	1	762	P	2035.902	0.059	0
OsJN01007	TIGR	9638.m00084	HP	HH	510293	+	1	1185	P	1505.432	0.259	0
OsJN01008	TIGR	9638.m01285	HP	LH	7451809	+	1	612	A	384.619	0.125	0
OsJN01009	TIGR	9638.m00090	HP	LH	546821	+	3	2050	P	1948.478	0.156	0
OsJN01010	TIGR	9638.m01419	EP	HH	8213302	+	2	1956	P	1241.698	0.143	0
OsJN01011	TIGR	9638.m00401	EP	LH	2370375	+	1	1437	P	826.373	0.188	0
OsJN01012	TIGR	9638.m00425	HP	LH	2493664	+	2	392	A	0	0	0
OsJN01013	TIGR	9638.m00836	HP	LH	4869899	+	3	825	P	3335.131	0.143	0.286
OsJN01014	TIGR	9638.m03962	PP	LH	2180706	+	1	2409	P	1832.759	0.173	0
OsJN01015	TIGR	9638.m01595	HP	LH	9224999	+	2	337	A	514.402	0.429	0
OsJN01016	TIGR	9638.m01538	HP	LH	8910990	+	2	938	P	1033.989	0.316	0
OsJN01017	TIGR	9638.m00390	EP	LH	2301440	+	2	292	A	0	0	0
OsJN01018	TIGR	9638.m00833	HP	LH	4844145	+	1	441	P	1029.885	0.571	0
OsJN01019	TIGR	9638.m00250	HP	LH	1448841	+	3	1246	P	1873.897	0.462	0.231
OsJN01020	TIGR	9638.m01654	EP	HH	9585424	+	9	5559	P	1188.284	0.197	0.019
OsJN01021	TIGR	9638.m01839	HP	LH	10753395	+	2	831	P	1065.986	0.438	0
OsJN01022	TIGR	9638.m00701	PP	HH	4159447	+	1	552	P	971.603	0.077	0
OsJN01023	TIGR	9638.m00459	HP	LH	2689672	+	5	825	A	0	0	0
OsJN01024	TIGR	9638.m00926	HP	LH	5451288	+	2	1360	P	1341.009	0.2	0.067
OsJN01025	TIGR	9638.m01879	PP	LH	11021753	+	2	2217	P	967.933	0.3	0.125
OsJN01026	TIGR	9638.m01627	HP	LH	9389513	+	1	378	P	1466.004	0.444	0
OsJN01027	TIGR	9638.m01895	EP	HH	11120333	+	10	5211	P	1362.378	0.259	0.071
OsJN01028	TIGR	9638.m01528	EP	LH	8869080	+	4	1328	P	662.684	0.15	0
OsJN01029	TIGR	9638.m01041	HP	LH	6069570	+	1	402	P	2202.993	0.444	0
OsJN01030	TIGR	9638.m01774	PP	HH	10357109	+	1	1518	P	14801.292	1	0
OsJN01031	TIGR	9638.m01200	PP	LH	7016232	+	18	9179	P	1126.245	0.13	0.08
OsJN01032	TIGR	9638.m00626	HP	HH	3719788	+	2	396	A	0	0	0
OsJN01033	TIGR	9638.m00597	EP	LH	3535384	+	4	2375	P	1520.869	0.065	0.048
OsJN01034	TIGR	9638.m01828	EP	HH	10681571	+	1	647	P	1351.582	0.133	0
OsJN01035	TIGR	9638.m00768	PP	HH	4507020	+	7	3356	P	1113.679	0.127	0
OsJN01036	TIGR	9638.m00500	HP	LH	2946018	+	4	4229	A	0	0	0.07
OsJN01037	TIGR	9638.m00052	HP	LH	300898	+	5	1578	A	0	0	0.375
OsJN01038	TIGR	9638.m00408	HP	LH	2401425	+	2	1132	P	925.59	0.143	0
OsJN01039	TIGR	9638.m00097	HP	HH	578742	+	5	2807	A	519.367	0.049	0
OsJN01040	TIGR	9638.m00434	EP	HH	2554240	+	5	1295	P	1308.066	0.067	0.143
OsJN01041	TIGR	9638.m00294	HP	LH	1736068	+	2	897	P	1231.868	0.688	0.25
OsJN01042	TIGR	9638.m01849	HP	LH	10851871	+	2	916	P	2280.39	0.357	0
OsJN01043	TIGR	9638.m00286	HP	LH	1699958	+	1	192	P	734.689	0.4	0
OsJN01044	TIGR	9638.m01697	EP	HH	9833324	+	6	5290	P	1544.473	0.175	0.027
OsJN01045	TIGR	9638.m00513	EP	LH	3015764	+	2	1104	P	1412.307	0.273	0
OsJN01046	TIGR	9638.m01767	EP	HH	10312066	+	2	966	P	1254.63	1	0
OsJN01047	TIGR	9638.m01923	HP	LH	11297323	+	3	3963	P	1422.961	0.143	0
OsJN01048	TIGR	9638.m00932	HP	LH	5494507	+	2	727	P	1192.196	0.111	0.25
OsJN01049	TIGR	9638.m00393	HP	LH	2320921	+	2	738	P	3161.048	0.333	0
OsJN01050	TIGR	9638.m01764	HP	LH	10282709	+	1	312	A	360.521	0.125	0
OsJN01051	TIGR	9638.m00259	HP	LH	1507806	+	2	779	P	1536.735	0.571	0
OsJN01052	TIGR	9638.m01313	HP	LH	7601820	+	5	1779	A	684.347	0.091	0.071
OsJN01053	TIGR	9638.m00718	HP	LH	4269036	+	1	687	P	2230.925	0.5	0
OsJN01054	TIGR	9638.m01913	EP	LH	11253655	+	2	1527	P	762.267	0.25	0.083
OsJN01055	TIGR	9638.m00364	EP	LH	2142236	+	2	890	A	0	0	0.25
OsJN01056	TIGR	9638.m00873	HP	LH	5084165	+	1	315	A	0	0	0
OsJN01057	TIGR	9638.m01562	HP	LH	9035748	+	6	3254	P	806.257	0.3	0.191
OsJN01058	TIGR	9638.m01231	PP	HH	7204514	+	12	5850	P	1828.393	0.094	0.093
OsJN01059	TIGR	9638.m01326	HP	LH	7681797	+	2	964	P	2100.703	0.364	0.111

OsJN01060	TIGR	9638.m00956	HP	LH	5627736	+	4	896	P	971.624	0.2	0
OsJN01061	TIGR	9638.m00919	HP	HH	5391698	+	2	1217	P	2600.539	0.167	0.1
OsJN01062	TIGR	9638.m00077	HP	LH	458062	+	4	2139	P	1147.963	0.333	0.1
OsJN01063	TIGR	9638.m00326	HP	LH	1922658	+	8	6906	P	1275.907	0.167	0.095
OsJN01064	TIGR	9638.m01120	HP	HH	6530124	+	6	4617	P	1687.421	0.125	0.1
OsJN01065	TIGR	9638.m00861	HP	LH	5007959	+	1	1704	A	0	0	0
OsJN01066	TIGR	9638.m00843	EP	HH	4898887	+	4	3093	P	1280.788	0.091	0.029
OsJN01067	TIGR	9638.m01063	PP	HH	6197541	+	7	3447	P	1091.918	0.211	0.029
OsJN01068	TIGR	9638.m00370	PP	LH	2176203	+	3	2542	P	1515.478	0.27	0
OsJN01069	TIGR	9638.m00989	EP	HH	5799427	+	13	7800	P	1139.236	0.245	0.1
OsJN01070	TIGR	9638.m00675	HP	LH	4028258	+	1	681	P	1122.494	0.286	0
OsJN01071	TIGR	9638.m01786	PP	HH	10452293	+	1	2802	P	1655.561	0.065	0
OsJN01072	TIGR	9638.m01835	EP	LH	10740252	+	1	721	P	807.24	0.25	0
OsJN01073	TIGR	9638.m01929	HP	LH	11337078	+	4	1850	P	1141.325	0.188	0.045
OsJN01074	TIGR	9638.m00649	HP	LH	3847757	+	2	449	P	1299.685	0.333	0
OsJN01075	TIGR	9638.m00151	HP	HH	888494	+	5	2675	A	406.525	0.077	0.103
OsJN01076	TIGR	9638.m01310	HP	HH	7588061	+	2	1027	P	581.932	0.429	0
OsJN01077	TIGR	9638.m01834	PP	HH	10724310	+	9	5581	P	1102.781	0.243	0.278
OsJN01078	TIGR	9638.m00432	HP	LH	2539454	+	1	1167	A	446.215	0.074	0
OsJN01079	TIGR	9638.m00311	HP	LH	1825899	+	1	1095	P	2459.449	0.125	0
OsJN01080	TIGR	9638.m00091	HP	LH	551557	+	3	2410	P	1201.852	0.381	0.333
OsJN01081	TIGR	9638.m00441	HP	HH	2603645	+	3	3545	P	1162.719	0.105	0.096
OsJN01082	TIGR	9638.m00351	EP	HH	2071618	+	2	974	P	2328.838	0.231	0.2
OsJN01083	TIGR	9638.m00791	HP	LH	4613953	+	3	2092	P	1269.815	0.133	0.067
OsJN01084	TIGR	9638.m01536	HP	HH	8901702	+	2	434	P	3752.373	0.111	0
OsJN01085	TIGR	9638.m01284	PP	HH	7448816	+	5	2135	P	1349.115	0.087	0
OsJN01086	TIGR	9638.m00215	HP	LH	1276773	+	2	1500	A	593.318	0.176	0
OsJN01087	TIGR	9638.m00741	PP	HH	4382103	+	2	918	A	0	0	0
OsJN01088	TIGR	9638.m01695	EP	LH	9833324	+	1	1929	P	1596.045	0.14	0
OsJN01089	TIGR	9638.m01634	HP	LH	9470419	+	4	1630	P	1425.324	0.2	0.091
OsJN01090	TIGR	9638.m00610	EP	LH	3601827	+	1	455	P	3394.029	0.1	0
OsJN01091	TIGR	9638.m00105	HP	LH	627965	+	1	438	P	1429.784	0.5	0
OsJN01092	TIGR	9638.m01668	EP	HH	9666493	+	6	3926	P	1670.033	0.245	0.031
OsJN01093	TIGR	9638.m01901	EP	HH	11158542	+	2	3985	P	560.969	0.122	0
OsJN01094	TIGR	9638.m00244	HP	LH	1420419	+	1	423	P	1403.26	0.778	0
OsJN01095	TIGR	9638.m01458	EP	HH	8454295	+	11	5072	P	1145.148	0.111	0.042
OsJN01096	TIGR	9638.m00153	EP	HH	908137	+	4	2334	P	939.663	0.16	0.118
OsJN01097	TIGR	9638.m01686	HP	LH	9767056	+	1	492	P	1256.24	0.5	0
OsJN01098	TIGR	9638.m00344	HP	LH	2037601	+	1	426	P	1850.092	0.3	0
OsJN01099	TIGR	9638.m01474	HP	LH	8539751	+	1	1140	P	1063.716	0.238	0
OsJN01100	TIGR	9638.m01306	HP	LH	7564505	+	1	801	P	1852.83	0.444	0
OsJN01101	TIGR	9638.m00426	HP	LH	2495126	+	1	216	P	2491.544	0.333	0
OsJN01102	TIGR	9638.m00365	EP	LH	2147270	+	2	1443	P	702.26	0.174	0.1
OsJN01103	TIGR	9638.m01792	EP	HH	10484285	+	7	4325	P	2106.93	0.378	0.052
OsJN01104	TIGR	9638.m01185	HP	LH	6907873	+	7	2748	P	638.937	0.087	0
OsJN01105	TIGR	9638.m01009	PP	HH	5921912	+	2	895	P	676.624	0.25	0.125
OsJN01106	TIGR	9638.m01444	PP	HH	8366384	+	11	4299	P	1395.276	0.205	0.018
OsJN01107	TIGR	9638.m00352	EP	HH	2074938	+	1	873	P	640.592	0.211	0
OsJN01108	TIGR	9638.m01032	HP	LH	6030382	+	2	528	P	1468.362	0.429	0.4
OsJN01109	TIGR	9638.m01816	EP	HH	10606197	+	1	783	P	1205.819	0.278	0
OsJN01110	TIGR	9638.m01333	HP	HH	7712494	+	4	2433	P	1148.188	0.136	0
OsJN01111	TIGR	9638.m01516	HP	LH	8798792	+	3	754	P	1159.46	0.5	0.333
OsJN01112	TIGR	9638.m00925	EP	HH	5437591	+	18	8113	P	888.045	0.116	0.094
OsJN01113	TIGR	9638.m00212	EP	HH	1258751	+	3	1616	P	839.622	0.207	0
OsJN01114	TIGR	9638.m01898	HP	LH	11148325	+	1	336	A	0	0	0
OsJN01115	TIGR	9638.m00307	EP	HH	1803639	+	2	1064	A	523.482	0.048	0

OsJN01116	TIGR	9638.m01529	EP	HH	8871877	+	2	1294	P	1130.189	0.308	0
OsJN01117	TIGR	9638.m01350	HP	LH	7801800	+	2	1372	P	1161.54	0.077	0.067
OsJN01118	TIGR	9638.m00433	EP	LH	2544132	+	10	3997	P	1376.653	0.156	0.018
OsJN01119	TIGR	9638.m00152	EP	HH	895721	+	4	5337	P	1023.086	0.226	0.079
OsJN01120	TIGR	9638.m01854	EP	HH	10879029	+	5	5270	P	1301.759	0.364	0.06
OsJN01121	TIGR	9638.m01755	PP	HH	10195215	+	3	2640	A	486.765	0.145	0
OsJN01122	TIGR	9638.m00366	EP	LH	2153247	+	1	1443	P	1272.67	0.063	0
OsJN01123	TIGR	9638.m00041	EP	HH	211933	+	12	6813	P	645.895	0.116	0.02
OsJN01124	TIGR	9638.m00062	HP	LH	370434	+	1	759	P	590.252	0.375	0
OsJN01125	TIGR	9638.m00228	HP	LH	1339585	+	2	1070	P	1393.837	0.25	0
OsJN01126	TIGR	9638.m01049	PP	HH	6094883	+	3	6667	P	680.259	0.75	0
OsJN01127	TIGR	9638.m00096	EP	HH	575478	+	1	1344	P	1342.256	0.067	0
OsJN01128	TIGR	9638.m01260	HP	LH	7337315	+	2	298	A	0	0	0
OsJN01129	TIGR	9638.m01052	PP	HH	6124934	+	2	5478	P	762.365	0.333	0
OsJN01130	TIGR	9638.m00376	HP	LH	2207172	+	3	1843	P	1526.173	0.25	0.1
OsJN01131	TIGR	9638.m00620	EP	HH	3680944	+	2	1978	P	1208.296	0.185	0.063
OsJN01132	TIGR	9638.m00789	EP	HH	4603668	+	3	1470	A	563.051	0.161	0
OsJN01133	TIGR	9638.m00754	EP	HH	4439981	+	8	5448	P	1008.591	0.132	0.149
OsJN01134	TIGR	9638.m00450	HP	LH	2641494	+	3	1061	P	2742.868	0.125	0.083
OsJN01135	TIGR	9638.m00898	EP	HH	5289344	+	6	2535	P	1200.852	0.25	0.214
OsJN01136	TIGR	9638.m00862	HP	LH	5017473	+	2	546	P	1892.966	0.273	0
OsJN01137	TIGR	9638.m00651	HP	LH	3852652	+	1	501	P	1788.429	0.455	0
OsJN01138	TIGR	9638.m00137	HP	LH	817841	+	5	1801	A	716.626	0.077	0
OsJN01139	TIGR	9638.m00040	EP	LH	211933	+	12	6813	P	645.895	0.116	0.02
OsJN01140	TIGR	9638.m01029	HP	LH	6018610	+	1	372	A	0	0	0
OsJN01141	TIGR	9638.m00258	HP	LH	1500564	+	1	390	P	1579.153	0.333	0
OsJN01142	TIGR	9638.m00660	EP	LH	3917157	+	2	2769	P	1609.309	0.406	0.037
OsJN01143	TIGR	9638.m00412	EP	LH	2429825	+	2	2304	P	1789.64	0.118	0
OsJN01144	TIGR	9638.m01394	HP	LH	8052151	+	2	855	P	1857.584	0.222	0.364
OsJN01145	TIGR	9638.m00138	PP	HH	820899	+	2	2242	P	1208.347	0.237	0
OsJN01146	TIGR	9638.m00229	HP	HH	1342147	+	1	1182	A	462.776	0.077	0
OsJN01147	TIGR	9638.m00131	EP	LH	797131	+	1	721	P	836.67	0.313	0
OsJN01148	TIGR	9638.m01790	HP	LH	10476338	+	2	648	A	1	0	0.5
OsJN01149	TIGR	9638.m00666	PP	LH	3968267	+	1	525	P	1681.048	0.333	0
OsJN01150	TIGR	9638.m00278	HP	LH	1656434	+	3	2904	A	408.628	0.1	0.188
OsJN01151	TIGR	9638.m01102	EP	HH	6451062	+	7	2866	P	1716.781	0.138	0.031
OsJN01152	TIGR	9638.m00573	HP	LH	3371344	+	3	1097	A	0	0	0
OsJN01153	TIGR	9638.m00261	PP	HH	1514780	+	4	1321	P	1396.055	0.182	0
OsJN01154	TIGR	9638.m00411	HP	LH	2420646	+	4	2573	P	1605.773	0.233	0.28
OsJN01155	TIGR	9638.m00772	HP	LH	4534831	+	2	532	A	0	0	0.333
OsJN01156	TIGR	9638.m01182	HP	HH	6899709	+	3	595	A	0	0	0
OsJN01157	TIGR	9638.m00818	HP	LH	4753195	+	1	441	P	1784.25	0.143	0
OsJN01158	TIGR	9638.m00842	EP	HH	4894311	+	2	1125	P	1440.178	0.261	0
OsJN01159	TIGR	9638.m00652	HP	LH	3858731	+	3	559	A	0	0	0.25
OsJN01160	TIGR	9638.m00556	HP	LH	3272176	+	6	3191	P	948.297	0.057	0
OsJN01161	TIGR	9638.m00901	EP	LH	5302821	+	1	786	A	441.801	0.056	0
OsJN01162	TIGR	9638.m00579	EP	HH	3422740	+	1	546	P	1231.248	0.231	0
OsJN01163	TIGR	9638.m01840	PP	HH	10769839	+	29	11026	P	1027.347	0.139	0
OsJN01164	TIGR	9638.m01254	HP	LH	7311564	+	1	288	P	962.562	0.286	0
OsJN01165	TIGR	9638.m00417	EP	HH	2463597	+	14	4572	P	1327.942	0.048	0.028
OsJN01166	TIGR	9638.m00103	EP	HH	616304	+	1	1143	P	1454.912	0.077	0
OsJN01167	TIGR	9638.m01221	PP	HH	7138324	+	24	11231	P	1531.175	0.106	0.055
OsJN01168	TIGR	9638.m01548	HP	LH	8941994	+	8	3197	P	680.169	0.094	0.067
OsJN01169	TIGR	9638.m01641	HP	LH	9520339	+	5	2465	A	598.326	0.167	0.333
OsJN01170	TIGR	9638.m00310	PP	LH	1822921	+	1	1221	P	950.711	0.231	0
OsJN01171	TIGR	9638.m01062	PP	HH	6184258	+	3	3364	A	538.021	0.053	0.154

OsJN01172	TIGR	9638.m00236	HP	LH	1375551	+	2	426	A	406.474	0.111	0
OsJN01173	TIGR	9638.m01606	EP	HH	9278374	+	5	3308	P	1173.791	0.043	0.087
OsJN01174	TIGR	9638.m01823	HP	LH	10654424	+	3	1458	A	0	0	0.158
OsJN01175	TIGR	9638.m01785	HP	LH	10441269	+	3	1007	P	1355.292	0.429	0.125
OsJN01176	TIGR	9638.m01212	HP	LH	7105810	+	3	1211	A	394.477	0.091	0.067
OsJN01177	TIGR	9638.m00769	EP	HH	4513852	+	3	2856	A	553.463	0.053	0.333
OsJN01178	TIGR	9638.m00431	EP	LH	2534257	+	3	2606	P	1052.22	0.133	0.095
OsJN01179	TIGR	9638.m01148	PP	HH	6695706	+	3	5742	P	1526.562	0.077	0
OsJN01180	TIGR	9638.m00968	PP	HH	5698331	+	3	4573	A	0	0	0
OsJN01181	TIGR	9638.m01051	PP	HH	6113969	+	4	6671	P	2445.754	0.143	0
OsJN01182	TIGR	9638.m00609	EP	HH	3596408	+	1	999	P	1425.348	0.182	0
OsJN01183	TIGR	9638.m01610	PP	HH	9304751	+	9	5738	P	900.606	0.169	0.015
OsJN01184	TIGR	9638.m00345	PP	HH	2039399	+	3	2974	P	1036.994	0.19	0
OsJN01185	TIGR	9638.m00095	EP	HH	567170	+	2	1634	P	719.459	0.147	0.5
OsJN01186	TIGR	9638.m01921	HP	HH	11288102	+	2	940	P	968.453	0.095	0
OsJN01187	TIGR	9638.m01630	HP	LH	9416208	+	2	1173	P	1154.512	0.083	0.077
OsJN01188	TIGR	9638.m01508	PP	HH	8747060	+	1	2223	P	963.415	0.204	0
OsJN01189	TIGR	9638.m01615	HP	LH	9323424	+	2	2295	P	694.443	0.111	0.111
OsJN01190	TIGR	9638.m00964	HP	LH	5681224	+	6	3509	P	1406.63	0.209	0
OsJN01191	TIGR	9638.m00184	HP	LH	1092945	+	2	1168	P	2261.545	0.389	0.556
OsJN01192	TIGR	9638.m00908	HP	LH	5336887	+	2	949	P	1157.239	0.077	0
OsJN01193	TIGR	9638.m00490	EP	LH	2889281	+	7	3238	P	1103.512	0.35	0.238
OsJN01194	TIGR	9638.m00243	EP	HH	1415289	+	1	1266	P	2154.28	0.25	0
OsJN01195	TIGR	9638.m01713	HP	LH	9944205	+	4	3472	P	1982.086	0.125	0.231
OsJN01196	TIGR	9638.m01843	PP	HH	10797231	+	2	2379	P	965.395	0.22	0.182
OsJN01197	TIGR	9638.m00021	HP	LH	114886	+	2	874	P	1747.25	0.5	0.667
OsJN01198	TIGR	9638.m01443	EP	HH	8360583	+	1	1103	P	837.858	0.28	0
OsJN01199	TIGR	9638.m00618	HP	LH	3652869	+	2	442	P	966.794	0.333	0
OsJN01200	TIGR	9638.m01013	HP	LH	5939721	+	1	546	P	854.926	0.154	0
OsJN01201	TIGR	9638.m00319	PP	HH	1881931	+	3	2792	P	810.103	0.034	0
OsJN01202	TIGR	9638.m00525	HP	LH	3098317	+	2	300	A	0	0	0
OsJN01203	TIGR	9638.m00716	HP	HH	4258926	+	2	2323	P	1256.101	0.292	0
OsJN01204	TIGR	9638.m00667	HP	LH	3969757	+	2	657	P	1566.343	0.308	0
OsJN01205	TIGR	9638.m00946	HP	HH	5562659	+	2	2223	P	1704.376	0.053	0.071
OsJN01206	TIGR	9638.m01862	HP	LH	10921020	+	2	393	A	0	0	0
OsJN01207	TIGR	9638.m00046	EP	LH	244164	+	1	489	P	1301.349	0.417	0
OsJN01208	TIGR	9638.m00074	EP	HH	442876	+	2	1836	P	1483.916	0.444	0.048
OsJN01209	TIGR	9638.m00737	PP	HH	4357008	+	3	1533	P	957.837	0.125	0.2
OsJN01210	TIGR	9638.m00792	HP	LH	4618502	+	1	360	A	608.027	0.25	0
OsJN01211	TIGR	9638.m00186	HP	LH	1101003	+	2	808	P	798.824	0.176	1
OsJN01212	TIGR	9638.m01076	HP	LH	6278634	+	2	1018	P	3192.806	0.286	0
OsJN01213	TIGR	9638.m01111	EP	HH	6485567	+	1	3099	A	0	0	0
OsJN01214	TIGR	9638.m00193	HP	LH	1144095	+	2	1708	A	418.307	0.167	0.032
OsJN01215	TIGR	9638.m01273	HP	LH	7392319	+	3	1098	P	2750.084	0.1	0
OsJN01216	TIGR	9638.m01202	HP	LH	7039257	+	6	2467	P	2514.465	0.071	0.179
OsJN01217	TIGR	9638.m00974	HP	LH	5725574	+	2	1102	P	1456.105	0.429	0
OsJN01218	TIGR	9638.m00838	EP	LH	4877797	+	2	753	A	534.077	0.154	0
OsJN01219	TIGR	9638.m01878	HP	LH	11019992	+	2	1285	P	1082.835	0.308	0
OsJN01220	TIGR	9638.m01501	HP	LH	8697706	+	3	1361	P	1286.186	0.167	0.063
OsJN01221	TIGR	9638.m01456	HP	LH	8442091	+	5	2306	P	892.37	0.048	0.103
OsJN01222	TIGR	9638.m00846	HP	LH	4922200	+	1	210	P	1170.146	0.4	0
OsJN01223	TIGR	9638.m00372	PP	HH	2185659	+	8	5224	P	1024.644	0.119	0.07
OsJN01224	TIGR	9638.m00875	HP	LH	5092537	+	3	1983	P	1217.251	0.5	0.12
OsJN01225	TIGR	9638.m00866	EP	HH	5036476	+	2	1100	P	770.595	0.217	0
OsJN01226	TIGR	9638.m01788	HP	LH	10465237	+	2	1415	P	1831.799	0.167	0.067
OsJN01227	TIGR	9638.m01307	HP	HH	7569724	+	1	309	A	418.291	0.25	0

OsJN01228	TIGR	9638.m00266	HP	LH	1538898	+	1	207	P	1231.252	0.4	0
OsJN01229	TIGR	9638.m00296	EP	HH	1750493	+	1	876	P	942.787	0.3	0
OsJN01230	TIGR	9638.m01531	HP	LH	8879425	+	4	3005	P	1301.357	0.304	0.054
OsJN01231	TIGR	9638.m00851	HP	LH	4946710	+	3	1104	P	667.847	0.667	0.316
OsJN01232	TIGR	9638.m00627	EP	LH	3722458	+	1	978	P	956.201	0.111	0
OsJN01233	TIGR	9638.m01095	PP	HH	6386885	+	2	1791	A	423.369	0.158	0.105
OsJN01234	TIGR	9638.m00930	HP	LH	5488108	+	2	838	A	477.795	0.429	0.273
OsJN01235	TIGR	9638.m00195	HP	LH	1164499	+	2	498	P	2247.065	0.429	0
OsJN01236	TIGR	9638.m00949	PP	HH	5593487	+	4	4578	P	1036.349	0.103	0
OsJN01237	TIGR	9638.m00267	PP	LH	1540961	+	1	1374	P	1145.789	0.233	0
OsJN01238	TIGR	9638.m01701	EP	LH	9853758	+	2	720	A	0	0	0
OsJN01239	TIGR	9638.m00179	HP	LH	1063243	+	4	1003	P	2904.723	0.077	0.1
OsJN01240	TIGR	9638.m01775	EP	HH	10362651	+	1	963	P	933.36	1	0
OsJN01241	TIGR	9638.m01294	HP	LH	7493005	+	1	642	P	535.476	1	0
OsJN01242	TIGR	9638.m01328	HP	LH	7688963	+	2	293	P	1475.558	0.667	0
OsJN01243	TIGR	9638.m01483	HP	HH	8575950	+	1	912	P	1034.89	0.095	0
OsJN01244	TIGR	9638.m01321	EP	HH	7647878	+	1	710	A	570.533	0.25	0
OsJN01245	TIGR	9638.m00690	HP	HH	4099532	+	1	540	P	1140.886	0.154	0
OsJN01246	TIGR	9638.m00246	EP	HH	1428149	+	1	1309	P	1797.04	0.276	0
OsJN01247	TIGR	9638.m00691	EP	HH	4102132	+	2	1898	P	1081.031	0.167	0.25
OsJN01248	TIGR	9638.m01655	HP	LH	9593165	+	1	328	A	0	0	0
OsJN01249	TIGR	9638.m01629	PP	HH	9394334	+	37	19015	P	1464.289	0.138	0.097
OsJN01250	TIGR	9638.m00596	EP	HH	3522060	+	1	978	P	708.903	0.227	0
OsJN01251	TIGR	9638.m01757	PP	HH	10223266	+	7	3611	P	1003.56	0.102	0
OsJN01252	TIGR	9638.m00421	EP	HH	2479456	+	2	1338	P	708.141	0.182	0
OsJN01253	TIGR	9638.m00806	HP	LH	4700625	+	2	297	P	842.142	0.333	0
OsJN01254	TIGR	9638.m01188	HP	LH	6929288	+	5	1198	A	0	0	0
OsJN01255	TIGR	9638.m00217	PP	HH	1292594	+	1	2499	P	808.522	0.109	0
OsJN01256	TIGR	9638.m01488	PP	LH	8612480	+	1	1440	P	2005.306	0.129	0
OsJN01257	TIGR	9638.m01804	PP	LH	10546886	+	4	1319	P	1937.872	0.4	0.222
OsJN01258	TIGR	9638.m00397	PP	HH	2343000	+	10	9865	P	1373.821	0.162	0.033
OsJN01259	TIGR	9638.m00410	EP	LH	2414284	+	4	2515	P	714.938	0.2	0.05
OsJN01260	TIGR	9638.m01203	HP	LH	7043613	+	1	625	P	1272.513	0.133	0
OsJN01261	TIGR	9638.m01104	HP	LH	6456643	+	2	1219	P	1375.557	0.6	0.083
OsJN01262	TIGR	9638.m00233	HP	LH	1365837	+	2	826	P	2750.717	0.273	0.125
OsJN01263	TIGR	9638.m00607	HP	LH	3579267	+	7	5090	P	1041.321	0.182	0.067
OsJN01264	TIGR	9638.m01068	PP	HH	6234358	+	8	4364	P	1141.878	0.205	0.04
OsJN01265	TIGR	9638.m00251	PP	HH	1452879	+	1	1383	P	1206.712	0.194	0
OsJN01266	TIGR	9638.m00024	HP	LH	124663	+	1	264	P	1645.49	0.333	0
OsJN01267	TIGR	9638.m01781	PP	HH	10428188	+	13	6641	P	1010.299	0.089	0.095
OsJN01268	TIGR	9638.m00511	EP	LH	3001036	+	2	1159	P	1709.711	0.167	0
OsJN01269	TIGR	9638.m00440	EP	HH	2599279	+	4	1724	A	660.838	0.158	0.056
OsJN01270	TIGR	9638.m00164	EP	HH	976793	+	5	2317	P	947.679	0.182	0.25
OsJN01271	TIGR	9638.m00068	PP	HH	411600	+	5	3193	P	1704.557	0.103	0.065
OsJN01272	TIGR	9638.m00496	HP	LH	2932855	+	2	866	A	524.357	0.286	0.083
OsJN01273	TIGR	9638.m01077	PP	HH	6282926	+	4	3015	P	1055.709	0.136	0.5
OsJN01274	TIGR	9638.m00961	HP	LH	5627736	+	4	896	P	971.624	0.2	0
OsJN01275	TIGR	9638.m01135	HP	LH	6614869	+	2	1038	A	642.77	0.2	0.5
OsJN01276	TIGR	9638.m00765	EP	HH	4492638	+	20	6367	A	556.453	0.087	0.017
OsJN01277	TIGR	9638.m00747	HP	LH	4412589	+	4	1313	P	961.082	0.286	0.091
OsJN01278	TIGR	9638.m01532	PP	HH	8883001	+	17	7384	P	1600.726	0.149	0.023
OsJN01279	TIGR	9638.m01330	HP	LH	7696821	+	5	3295	P	1346.458	0.292	0.023
OsJN01280	TIGR	9638.m00837	EP	HH	4871972	+	2	840	P	1730.377	0.077	0
OsJN01281	TIGR	9638.m00043	HP	LH	227708	+	2	1022	P	1627.726	0.444	0.615
OsJN01282	TIGR	9638.m01504	PP	LH	8724585	+	2	1384	P	861.431	0.176	0.071
OsJN01283	TIGR	9638.m00590	EP	HH	3484712	+	2	3554	P	1754.637	0.079	0

OsJN01284	TIGR	9638.m01473	PP	LH	8534695	+	6	2068	P	2052.974	0.188	0
OsJN01285	TIGR	9638.m00424	HP	LH	2491509	+	1	582	P	801.152	0.462	0
OsJN01286	TIGR	9638.m00738	HP	LH	4365867	+	4	4847	A	0	0	0.047
OsJN01287	TIGR	9638.m01842	PP	HH	10796676	+	2	2934	P	1129.282	0.214	0.286
OsJN01288	TIGR	9638.m01702	HP	LH	9856050	+	2	1147	A	0	0	0.056
OsJN01289	TIGR	9638.m01118	HP	LH	6525623	+	3	1084	P	851.579	0.231	0
OsJN01290	TIGR	9638.m01756	PP	HH	10207343	+	4	4146	P	1372.017	0.195	0.049
OsJN01291	TIGR	9638.m01719	HP	LH	9999257	+	1	393	P	2043.937	0.3	0
OsJN01292	TIGR	9638.m01145	HP	LH	6676540	+	2	981	P	1674.13	0.455	0.4
OsJN01293	TIGR	9638.m00118	HP	LH	717014	+	9	3049	P	636.167	0.095	0
OsJN01294	TIGR	9638.m00087	HP	LH	525038	+	3	1741	P	2091.872	0.071	0.056
OsJN01295	TIGR	9638.m00923	HP	HH	5416451	+	4	7635	P	1212.709	0.215	0.049
OsJN01296	TIGR	9638.m00047	HP	HH	249250	+	4	2020	A	418.536	0.167	0
OsJN01297	TIGR	9638.m01280	HP	LH	7419025	+	2	669	A	0	0	0
OsJN01298	TIGR	9638.m01909	PP	HH	11219368	+	2	2935	P	2044.435	0.579	0.079
OsJN01299	TIGR	9638.m00337	HP	LH	1995146	+	1	882	P	662.417	0.211	0
OsJN01300	TIGR	9638.m00584	HP	LH	3447049	+	1	1125	P	1582.295	0.2	0
OsJN01301	TIGR	9638.m01392	HP	LH	8040956	+	5	1018	P	1424.036	0.067	0
OsJN01302	TIGR	9638.m01467	HP	LH	8496153	+	4	1872	P	1385.831	0.105	0
OsJN01303	TIGR	9638.m00368	PP	LH	2169439	+	3	3244	P	1504.907	0.178	0
OsJN01304	TIGR	9638.m01386	HP	LH	7996619	+	4	1972	A	0	0	0.121
OsJN01305	TIGR	9638.m00628	HP	LH	3728353	+	1	321	P	1548.273	0.625	0
OsJN01306	TIGR	9638.m01080	PP	HH	6296520	+	3	5561	P	1153.333	0.18	0.086
OsJN01307	TIGR	9638.m00142	EP	HH	850585	+	2	1947	P	1363.906	0.481	0
OsJN01308	TIGR	9638.m01765	HP	HH	10284941	+	5	2110	P	1063.522	0.143	0.077
OsJN01309	TIGR	9638.m00852	HP	LH	4951461	+	3	2257	P	1257.943	0.538	0.222
OsJN01310	TIGR	9638.m00864	HP	LH	5022153	+	1	822	P	1772.002	0.278	0
OsJN01311	TIGR	9638.m00533	EP	LH	3150278	+	1	441	P	1667.63	0.667	0
OsJN01312	TIGR	9638.m00512	EP	LH	3004848	+	2	1437	P	1134.903	0.375	0
OsJN01313	TIGR	9638.m00462	EP	LH	2704437	+	1	354	P	1199.469	0.778	0
OsJN01314	TIGR	9638.m01696	EP	LH	9833324	+	4	4783	P	1596.045	0.15	0.031
OsJN01315	TIGR	9638.m00470	HP	LH	2757182	+	3	1508	P	801.038	0.333	0
OsJN01316	TIGR	9638.m00567	EP	HH	3325822	+	1	1242	P	567.111	0.222	0
OsJN01317	TIGR	9638.m00951	HP	LH	5602904	+	5	872	A	0	0	0
OsJN01318	TIGR	9638.m00073	EP	HH	440133	+	1	1305	P	937.319	0.241	0
OsJN01319	TIGR	9638.m00602	EP	HH	3561388	+	8	5811	P	1364.671	0.103	0.133
OsJN01320	TIGR	9638.m01293	HP	LH	7487352	+	1	498	P	1815.252	0.5	0
OsJN01321	TIGR	9638.m00027	EP	LH	149921	+	2	307	A	600.437	0.25	0
OsJN01322	TIGR	9638.m01039	PP	HH	6061261	+	1	1283	P	1078.319	0.172	0
OsJN01323	TIGR	9638.m01015	HP	HH	5944412	+	3	3049	P	1400.222	0.225	0.04
OsJN01324	TIGR	9638.m01395	HP	LH	8061842	+	3	1869	P	1971.365	0.143	0.222
OsJN01325	TIGR	9638.m00418	EP	HH	2468856	+	11	4864	P	843.12	0.135	0.031
OsJN01326	TIGR	9638.m01278	HP	LH	7410345	+	3	968	P	1333.798	0.222	0.125
OsJN01327	TIGR	9638.m00987	PP	HH	5791581	+	2	1743	P	1710.781	0.061	0.2
OsJN01328	TIGR	9638.m00169	EP	LH	1003644	+	7	2481	A	526.675	0.136	0.065
OsJN01329	TIGR	9638.m01870	HP	LH	10971703	+	4	1743	P	1186.869	0.222	0
OsJN01330	TIGR	9638.m00300	HP	HH	1767471	+	1	654	A	499.914	0.143	0
OsJN01331	TIGR	9638.m00173	HP	HH	1037827	+	2	460	P	1068.9	0.714	0
OsJN01332	TIGR	9638.m00029	HP	LH	159650	+	5	2567	P	1872.663	0.069	0.071
OsJN01333	TIGR	9638.m01728	HP	LH	10057393	+	1	366	P	3368.304	0.111	0
OsJN01334	TIGR	9638.m01336	PP	HH	7728086	+	8	6654	P	1106.125	0.133	0.12
OsJN01335	TIGR	9638.m00373	EP	LH	2196165	+	1	232	P	1691.716	0.5	0
OsJN01336	TIGR	9638.m01736	HP	HH	10084444	+	3	1011	P	1030.72	0.111	0.231
OsJN01337	TIGR	9638.m00948	PP	HH	5573240	+	2	1988	P	995.753	0.121	0
OsJN01338	TIGR	9638.m01608	PP	HH	9291094	+	3	3559	A	483.161	0.014	0
OsJN01339	TIGR	9638.m00759	HP	LH	4471525	+	5	1971	P	1057.501	0.333	0.031

OsJN01340	TIGR	9638.m00727	PP	LH	4312388	+	12	6181	P	1266.982	0.298	0.013
OsJN01341	TIGR	9638.m00216	HP	LH	1285136	+	1	342	P	1843.818	0.286	0
OsJN01342	TIGR	9638.m01256	HP	HH	7323287	+	2	1496	P	726.886	0.219	0
OsJN01343	TIGR	9638.m01649	HP	LH	9569169	+	2	1007	P	1464.374	0.273	0.1
OsJN01344	TIGR	9638.m01074	PP	HH	6268954	+	5	3860	P	819.581	0.083	0
OsJN01345	TIGR	9638.m00479	EP	HH	2821529	+	1	2565	P	1439.972	0.281	0
OsJN01346	TIGR	9638.m00844	EP	HH	4904164	+	3	482	P	1510.568	0.111	0
OsJN01347	TIGR	9638.m00721	HP	LH	4281965	+	4	3063	P	1425.746	0.182	0.054
OsJN01348	TIGR	9638.m01234	PP	HH	7222548	+	3	3377	P	1064.74	0.069	0
OsJN01349	TIGR	9638.m00697	HP	LH	4137645	+	2	494	P	944.49	0.25	0
OsJN01350	TIGR	9638.m01831	HP	LH	10707568	+	2	837	A	0	0	0.167
OsJN01351	TIGR	9638.m00722	PP	HH	4286070	+	5	6834	P	1147.422	0.229	0.081
OsJN01352	TIGR	9638.m01036	HP	LH	6051063	+	1	474	P	710.225	0.5	0
OsJN01353	TIGR	9638.m00732	PP	HH	4337650	+	2	1977	P	1225.524	0.148	0
OsJN01354	TIGR	9638.m01073	PP	LH	6263970	+	2	1493	P	1018.549	0.182	0
OsJN01355	TIGR	9638.m00361	EP	LH	2131119	+	2	1526	P	1327.792	0.097	0
OsJN01356	TIGR	9638.m01910	PP	LH	11227533	+	1	966	P	1258.759	0.25	0
OsJN01357	TIGR	9638.m01525	EP	LH	8849408	+	1	886	P	1103.621	0.45	0
OsJN01358	TIGR	9638.m00608	HP	LH	3585909	+	1	453	A	0	0	0
OsJN01359	TIGR	9638.m01108	PP	HH	6473048	+	3	3454	A	0	0	0.5
OsJN01360	TIGR	9638.m00453	HP	LH	2658956	+	1	432	P	1457.06	0.7	0
OsJN01361	TIGR	9638.m00272	EP	HH	1579197	+	4	3560	P	1668.494	0.25	0.065
OsJN01362	TIGR	9638.m00358	EP	LH	2112992	+	3	2828	P	852.442	0.029	0
OsJN01363	TIGR	9638.m01801	HP	LH	10529888	+	1	222	A	0	0	0
OsJN01364	TIGR	9638.m01393	HP	LH	8048160	+	1	390	P	1034.842	0.444	0
OsJN01365	TIGR	9638.m01387	HP	LH	8005986	+	3	1311	P	2532.424	0.182	0
OsJN01366	TIGR	9638.m00622	EP	LH	3700565	+	3	832	A	624.628	0.071	0
OsJN01367	TIGR	9638.m01405	HP	LH	8129157	+	1	300	P	2139.378	0.571	0
OsJN01368	TIGR	9638.m00330	HP	LH	1945917	+	5	2121	P	1101.328	0.211	0.036
OsJN01369	TIGR	9638.m01515	PP	HH	8792677	+	4	2626	P	2051.82	0.154	0
OsJN01370	TIGR	9638.m00889	HP	LH	5227623	+	5	3287	A	459.926	0.063	0.037
OsJN01371	TIGR	9638.m00143	EP	HH	853661	+	2	1380	P	1685.714	0.231	0
OsJN01372	TIGR	9638.m01741	HP	LH	10100426	+	1	309	P	969.861	0.286	0
OsJN01373	TIGR	9638.m01860	EP	LH	10910256	+	3	2764	P	1261.995	0.111	0.2
OsJN01374	TIGR	9638.m01144	HP	LH	6675235	+	1	198	A	404.799	0.4	0
OsJN01375	TIGR	9638.m01057	HP	LH	6153864	+	3	1042	P	1814.313	0.214	0
OsJN01376	TIGR	9638.m01640	EP	LH	9518488	+	1	814	P	1478.316	0.529	0
OsJN01377	TIGR	9638.m01808	HP	LH	10554512	+	2	1353	A	566.035	0.25	0.053
OsJN01378	TIGR	9638.m01472	PP	HH	8531689	+	1	1368	P	2346.469	0.214	0
OsJN01379	TIGR	9638.m01006	PP	HH	5901318	+	6	5570	P	1646.786	0.231	0.054
OsJN01380	TIGR	9638.m01005	PP	HH	5901318	+	6	5570	P	1646.786	0.231	0.054
OsJN01381	TIGR	9638.m00591	HP	LH	3494407	+	3	1792	A	0	0	0.097
OsJN01382	TIGR	9638.m00503	EP	LH	2962569	+	7	3084	P	1459.242	0.152	0.029
OsJN01383	TIGR	9638.m01468	HP	HH	8514317	+	4	4352	P	1372.77	0.156	0.031
OsJN01384	TIGR	9638.m01061	PP	HH	6176072	+	5	4593	P	1075.351	0.179	0
OsJN01385	TIGR	9638.m03963	PP	LH	2180706	+	2	2409	P	1867.239	0.189	0.133
OsJN01386	TIGR	9638.m01768	PP	HH	10322827	+	9	8725	P	3349.952	0.528	0.423
OsJN01387	TIGR	9638.m00984	EP	LH	5768369	+	7	3543	P	2065.473	0.038	0.059
OsJN01388	TIGR	9638.m01514	PP	HH	8785050	+	2	2773	A	0	0	0
OsJN01389	TIGR	9638.m01855	EP	LH	10886560	+	1	508	P	1895.161	0.583	0
OsJN01390	TIGR	9638.m00848	HP	LH	4932102	+	2	651	P	1059.049	0.231	0
OsJN01391	TIGR	9638.m01459	EP	HH	8459521	+	2	375	P	1514.782	0.25	0
OsJN01392	TIGR	9638.m00133	EP	HH	802846	+	4	3185	P	630.236	0.139	0.121
OsJN01393	TIGR	9638.m00480	EP	HH	2824220	+	7	4984	P	1648.482	0.316	0.102
OsJN01394	TIGR	9638.m00414	EP	LH	2442197	+	2	544	P	1450.861	0.455	0
OsJN01395	TIGR	9638.m01932	HP	LH	11349757	+	2	1639	A	0	0	0.111

OsJN01396	TIGR	9638.m01658	HP	LH	9603747	+	1	552	P	2080.31	0.3	0
OsJN01397	TIGR	9638.m00360	EP	LH	2127565	+	1	984	P	2080.773	0.091	0
OsJN01398	TIGR	9638.m01926	HP	LH	11321990	+	2	413	P	2114.129	0.25	0
OsJN01399	TIGR	9638.m01526	HP	LH	8853116	+	5	3553	P	1013.664	0.116	0.091
OsJN01400	TIGR	9638.m00389	HP	LH	2291946	+	2	252	A	0	0	0
OsJN01401	TIGR	9638.m01235	PP	HH	7231431	+	3	6096	A	0	0	0
OsJN01402	TIGR	9638.m01918	HP	LH	11275901	+	2	710	P	1196.729	0.4	0
OsJN01403	TIGR	9638.m00394	HP	LH	2323744	+	4	1094	P	851.326	0.235	0.143
OsJN01404	TIGR	9638.m01249	HP	LH	7293256	+	1	264	A	0	0	0
OsJN01405	TIGR	9638.m00764	PP	HH	4488786	+	3	2996	P	1474.502	0.444	0.106
OsJN01406	TIGR	9638.m01533	HP	LH	8896078	+	1	345	P	1295.18	0.111	0
OsJN01407	TIGR	9638.m01072	HP	LH	6255892	+	3	483	P	1411.382	0.5	0.2
OsJN01408	TIGR	9638.m01827	HP	LH	10669095	+	1	339	P	1452.4	0.625	0
OsJN01409	TIGR	9638.m00905	HP	LH	5320913	+	1	357	P	2002.009	0.875	0
OsJN01410	TIGR	9638.m00452	EP	HH	2651212	+	2	2085	P	2166.358	0.068	0
OsJN01411	TIGR	9638.m01215	HP	LH	7122036	+	3	1007	A	0	0	0
OsJN01412	TIGR	9638.m00587	EP	HH	3456305	+	6	3030	P	728.75	0.179	0
OsJN01413	TIGR	9638.m00056	EP	HH	328500	+	8	3350	P	1015.094	0.167	0.079
OsJN01414	TIGR	9638.m01881	PP	HH	11032364	+	3	2316	P	1204.44	0.238	0.107
OsJN01415	TIGR	9638.m01797	PP	LH	10509923	+	6	2906	P	1007.813	0.12	0.111
OsJN01416	TIGR	9638.m00505	HP	LH	2970022	+	2	1355	A	703.344	0.111	0.091
OsJN01417	TIGR	9638.m00952	EP	LH	5605567	+	1	695	P	2408.662	0.5	0
OsJN01418	TIGR	9638.m01850	HP	LH	10854890	+	9	4053	P	1421.016	0.108	0.077
OsJN01419	TIGR	9638.m01322	HP	LH	7653767	+	5	1872	P	1012.619	0.182	0.05
OsJN01420	TIGR	9638.m01584	HP	LH	9166862	+	2	505	A	0	0	0
OsJN01421	TIGR	9638.m00995	HP	LH	5849600	+	2	380	P	1107.356	0.286	1
OsJN01422	TIGR	9638.m01078	PP	HH	6285986	+	6	3138	A	0	0	0.143
OsJN01423	TIGR	9638.m00369	PP	LH	2176203	+	2	2365	P	1609.59	0.25	0
OsJN01424	TIGR	9638.m00731	HP	LH	4331682	+	1	1230	P	1313.87	0.179	0
OsJN01425	TIGR	9638.m00072	EP	HH	432816	+	4	4291	P	692.609	0.176	0.111
OsJN01426	TIGR	9638.m00357	EP	LH	2106744	+	2	1142	P	2033.094	0.04	0
OsJN01427	TIGR	9638.m01159	HP	LH	6754694	+	1	435	A	1	0	0
OsJN01428	TIGR	9638.m00026	EP	LH	144061	+	1	291	A	530.105	0.143	0
OsJN01429	TIGR	9638.m01465	HP	HH	8482348	+	7	3536	P	929.197	0.091	0
OsJN01430	TIGR	9638.m01024	PP	HH	5984225	+	1	4500	A	0	0	0
OsJN01431	TIGR	9638.m01152	HP	LH	6710742	+	1	435	A	1	0	0
OsJN01432	TIGR	9638.m00436	PP	HH	2576662	+	3	4120	P	679.401	0.175	0.296
OsJN01433	TIGR	9638.m01591	PP	HH	9201117	+	3	3271	A	606.499	0.039	0
OsJN01434	TIGR	9638.m01471	PP	HH	8526381	+	5	4713	P	1458.119	0.289	0.109
OsJN01435	TIGR	9638.m00427	EP	HH	2502112	+	5	2519	P	950.956	0.063	0.125
OsJN01436	TIGR	9638.m00398	HP	LH	2354226	+	1	246	P	1625.612	1	0
OsJN01437	TIGR	9638.m00303	PP	HH	1776362	+	1	876	A	531.883	0.05	0
OsJN01438	TIGR	9638.m01302	HP	LH	7547351	+	1	150	A	528.459	0.25	0
OsJN01439	TIGR	9638.m01217	EP	HH	7126582	+	3	2891	P	1484.789	0.222	0.192
OsJN01440	TIGR	9638.m01858	HP	LH	10900652	+	4	2313	P	1631.946	0.129	0.053
OsJN01441	TIGR	9638.m00188	HP	LH	1115173	+	1	318	A	616.789	0.143	0
OsJN01442	TIGR	9638.m00699	HP	HH	4154507	+	1	903	P	2131.175	0.2	0
OsJN01443	TIGR	9638.m01614	HP	LH	9322092	+	1	195	A	584.059	0.6	0
OsJN01444	TIGR	9638.m00467	EP	LH	2736671	+	2	616	P	2129.414	0.778	0.4
OsJN01445	TIGR	9638.m00066	HP	LH	402845	+	2	353	P	752.193	0.167	0.5
OsJN01446	TIGR	9638.m00963	PP	HH	5673674	+	2	2171	P	1760.513	0.311	0
OsJN01447	TIGR	9638.m00605	HP	LH	3572311	+	2	989	P	1120.768	0.2	0.077
OsJN01448	TIGR	9638.m01017	HP	LH	5961091	+	3	1193	A	621.551	0.4	0.059
OsJN01449	TIGR	9638.m01422	HP	LH	8233501	+	3	1164	A	0	0	0.125
OsJN01450	TIGR	9638.m00891	HP	LH	5249741	+	7	2385	A	505.916	0.071	0.043
OsJN01451	TIGR	9638.m00793	HP	LH	4623254	+	5	3475	P	1588.204	0.333	0.143

OsJN01452	TIGR	9638.m01208	HP	LH	7082713	+	2	486	A	503.483	0.333	0
OsJN01453	TIGR	9638.m00274	EP	HH	1626712	+	1	4206	P	1337.902	0.132	0
OsJN01454	TIGR	9638.m01460	EP	HH	8461897	+	3	1111	P	1065.287	0.333	0
OsJN01455	TIGR	9638.m00506	EP	LH	2975064	+	2	778	P	1699.825	0.267	0
OsJN01456	TIGR	9638.m00455	EP	HH	2666402	+	3	2077	P	1777.899	0.071	0.25
OsJN01457	TIGR	9638.m01659	HP	LH	9606323	+	2	2093	P	1485.003	0.174	0.087
OsJN01458	TIGR	9638.m00248	HP	LH	1436507	+	3	1036	P	1346.878	0.1	0.154
OsJN01459	TIGR	9638.m01296	HP	LH	7502423	+	1	381	A	1	0	0
OsJN01460	TIGR	9638.m00363	EP	LH	2135412	+	1	1452	A	581.367	0.063	0
OsJN01461	TIGR	9638.m01875	PP	HH	11009292	+	4	2526	P	948.125	0.024	0.111
OsJN01462	TIGR	9638.m00749	HP	LH	4419601	+	1	465	P	4675.957	0.091	0
OsJN01463	TIGR	9638.m00592	PP	HH	3499929	+	2	2865	P	607.598	0.097	0
OsJN01464	TIGR	9638.m01779	PP	HH	10401855	+	3	3588	P	1123.192	0.667	0.6
OsJN01465	TIGR	9638.m01495	HP	LH	8673334	+	1	831	P	1084.958	0.158	0
OsJN01466	TIGR	9638.m00880	HP	LH	5142626	+	1	675	P	923.376	0.125	0
OsJN01467	TIGR	9638.m00656	HP	LH	3887829	+	3	1217	A	532.415	0.091	0.077
OsJN01468	TIGR	9638.m00086	HP	LH	520104	+	4	2069	P	2079.662	0.133	0.087
OsJN01469	TIGR	9638.m01066	HP	LH	6224234	+	2	2822	P	1671.176	0.375	0.083
OsJN01470	TIGR	9638.m01314	HP	LH	7608172	+	3	576	A	498.063	0.286	0
OsJN01471	TIGR	9638.m01390	EP	HH	8028352	+	1	1036	P	885.008	0.304	0
OsJN01472	TIGR	9638.m00899	PP	HH	5292865	+	18	6711	P	1185.528	0.194	0.051
OsJN01473	TIGR	9638.m00225	HP	LH	1325906	+	1	1044	P	803.396	0.091	0
OsJN01474	TIGR	9638.m01088	PP	HH	6355500	+	4	3432	P	1076.575	0.121	0.024
OsJN01475	TIGR	9638.m00473	HP	LH	2786075	+	2	409	P	1139.169	0.167	0.5
OsJN01476	TIGR	9638.m01889	HP	LH	11084695	+	5	2457	P	1904.018	0.071	0.094
OsJN01477	TIGR	9638.m00187	EP	HH	1108979	+	7	5244	P	1213.146	0.163	0.042
OsJN01478	TIGR	9638.m01500	HP	LH	8692591	+	4	2656	P	1824.376	0.125	0.071
OsJN01479	TIGR	9638.m01421	EP	HH	8225863	+	9	5357	P	886.632	0.385	0.145
OsJN01480	TIGR	9638.m00196	HP	LH	1167711	+	1	390	A	659.002	0.2	0
OsJN01481	TIGR	9638.m01680	HP	LH	9733787	+	4	1697	A	0	0	0.053
OsJN01482	TIGR	9638.m01229	EP	HH	7193986	+	1	578	P	1122.748	0.385	0
OsJN01483	TIGR	9638.m00451	EP	HH	2646458	+	1	1953	P	889.952	0.14	0
OsJN01484	TIGR	9638.m01880	PP	HH	11024984	+	6	6353	P	1206.607	0.135	0.09
OsJN01485	TIGR	9638.m01071	HP	LH	6250945	+	5	1686	P	1282.671	0.533	0.267
OsJN01486	TIGR	9638.m01749	HP	LH	10151481	+	2	649	P	1525.266	0.333	0
OsJN01487	TIGR	9638.m01700	HP	LH	9849842	+	4	2987	P	2216.941	0.353	0.021
OsJN01488	TIGR	9638.m01141	EP	HH	6649974	+	5	5838	P	1093.445	0.083	0.087
OsJN01489	TIGR	9638.m00714	HP	LH	4249256	+	8	2762	P	1097.458	0.156	0.034
OsJN01490	TIGR	9638.m01625	HP	LH	9379721	+	5	1838	A	331.935	0.071	0.042
OsJN01491	TIGR	9638.m01441	HP	HH	8349270	+	2	739	P	1669.017	0.25	0
OsJN01492	TIGR	9638.m01046	HP	LH	6078946	+	1	462	P	1526.833	0.273	0
OsJN01493	TIGR	9638.m00586	PP	LH	3452203	+	1	1140	P	1397.623	0.292	0
OsJN01494	TIGR	9638.m00996	HP	HH	5851679	+	6	2683	P	897.877	0.119	0
OsJN01495	TIGR	9638.m01919	HP	LH	11278454	+	2	787	P	1941.534	0.286	0
OsJN01496	TIGR	9638.m01197	HP	LH	6995955	+	2	964	A	1	0	0
OsJN01497	TIGR	9638.m01412	HP	LH	8167610	+	1	517	A	636.626	0.125	0
OsJN01498	TIGR	9638.m01603	HP	HH	9261960	+	3	2185	P	1184.093	0.25	0.108
OsJN01499	TIGR	9638.m00865	PP	HH	5025970	+	1	387	A	0	0	0
OsJN01500	TIGR	9638.m03548	HP	LH	20466927	-	3	827	A	0	0	0.167
OsJN01501	TIGR	9638.m03792	HP	HH	21848359	-	21	13596	P	930.655	0.072	0.067
OsJN01502	TIGR	9638.m03585	PP	HH	20667664	-	4	3970	P	1413.873	0.151	0.091
OsJN01503	TIGR	9638.m03482	PP	HH	20116429	-	2	1159	P	1804.009	0.261	0.5
OsJN01504	TIGR	9638.m03869	PP	HH	22272019	-	2	1848	P	1323.45	0.24	0
OsJN01505	TIGR	9638.m03738	EP	HH	21559425	-	4	3474	P	1795.626	0.308	0.1
OsJN01506	TIGR	9638.m01981	HP	HH	11642704	-	3	1507	A	1	0	0
OsJN01507	TIGR	9638.m03197	PP	HH	18735424	-	10	5555	P	1413.703	0.123	0.111

OsJN01508	TIGR	9638.m02796	HP	LH	16424851	-	2	707	P	2257.252	0.5	0
OsJN01509	TIGR	9638.m02400	EP	HH	14251238	-	7	2354	P	1701.975	0.72	0.111
OsJN01510	TIGR	9638.m02107	PP	HH	12375349	-	3	888	P	1445.876	0.071	0
OsJN01511	TIGR	9638.m02121	HP	LH	12485279	-	1	303	P	1109.791	0.143	0
OsJN01512	TIGR	9638.m03333	EP	LH	19468890	-	2	836	P	1440.198	0.222	1
OsJN01513	TIGR	9638.m02760	PP	HH	16204788	-	10	2981	P	1325.888	0.207	0.114
OsJN01514	TIGR	9638.m02417	HP	LH	14323449	-	2	236	P	901.393	0.167	0
OsJN01515	TIGR	9638.m02086	EP	HH	12251345	-	10	4169	P	902.358	0.333	0.024
OsJN01516	TIGR	9638.m03884	HP	HH	22366727	-	4	2557	P	2142.909	0.14	0
OsJN01517	TIGR	9638.m03695	EP	HH	21327217	-	3	1539	P	1959.891	0.1	0.333
OsJN01518	TIGR	9638.m02878	PP	HH	16779479	-	9	6122	P	849.011	0.033	0
OsJN01519	TIGR	9638.m02736	HP	LH	16074620	-	2	2586	P	2849.062	0.353	0
OsJN01520	TIGR	9638.m02682	HP	LH	15776305	-	2	369	P	1025.567	0.429	0
OsJN01521	TIGR	9638.m02252	HP	HH	13219585	-	2	381	A	0	0	0
OsJN01522	TIGR	9638.m02697	HP	HH	15887727	-	2	4975	P	1480.424	0.17	0
OsJN01523	TIGR	9638.m03516	PP	HH	20288151	-	5	4311	P	1311.979	0.149	0.105
OsJN01524	TIGR	9638.m03722	PP	HH	21488343	-	4	2409	P	1424.821	0.091	0
OsJN01525	TIGR	9638.m03213	PP	HH	18818401	-	3	3434	A	1	0	0
OsJN01526	TIGR	9638.m03420	PP	HH	19886709	-	1	2541	P	761.621	0.091	0
OsJN01527	TIGR	9638.m02102	HP	LH	12351243	-	1	483	P	1336.336	0.2	0
OsJN01528	TIGR	9638.m02092	HP	LH	12282466	-	4	1551	P	3566.78	0.091	0.227
OsJN01529	TIGR	9638.m02745	EP	LH	16125096	-	1	733	P	2245.193	0.647	0
OsJN01530	TIGR	9638.m03328	PP	HH	19426081	-	6	5787	P	1516.868	0.373	0.209
OsJN01531	TIGR	9638.m03893	EP	LH	22434965	-	3	891	A	0	0	0
OsJN01532	TIGR	9638.m03622	HP	HH	20921427	-	3	1270	A	0	0	0.053
OsJN01533	TIGR	9638.m02471	HP	LH	14628756	-	3	380	A	0	0	0
OsJN01534	TIGR	9638.m02962	HP	HH	17387771	-	1	615	P	724.719	0.25	0
OsJN01535	TIGR	9638.m02114	PP	HH	12418901	-	16	5475	P	1111.556	0.197	0.082
OsJN01536	TIGR	9638.m03538	EP	LH	20426682	-	5	4965	P	1260.997	0.185	0.077
OsJN01537	TIGR	9638.m02559	HP	LH	14946997	-	2	602	P	1628.157	0.143	0.333
OsJN01538	TIGR	9638.m02620	HP	LH	15360618	-	1	267	P	2789.297	0.143	0
OsJN01539	TIGR	9638.m03344	HP	HH	19519818	-	1	411	P	3508.15	0.667	0
OsJN01540	TIGR	9638.m03572	PP	HH	20606521	-	15	4692	P	2003.823	0.125	0.045
OsJN01541	TIGR	9638.m03489	HP	LH	20141468	-	2	481	P	2163.423	0.182	0
OsJN01542	TIGR	9638.m03230	PP	HH	18906758	-	2	796	P	740.779	0.188	0
OsJN01543	TIGR	9638.m02446	PP	HH	14505428	-	1	1119	P	1267.506	0.12	0
OsJN01544	TIGR	9638.m02612	PP	HH	15312368	-	8	3676	A	664.364	0.087	0.035
OsJN01545	TIGR	9638.m01986	HP	LH	11686842	-	2	463	A	1	0	0
OsJN01546	TIGR	9638.m03741	EP	HH	21578332	-	7	1928	P	1477.501	0.238	0.1
OsJN01547	TIGR	9638.m02522	HP	LH	14831170	-	3	1163	P	1425.388	0.5	0.5
OsJN01548	TIGR	9638.m02847	PP	HH	16619916	-	10	4091	P	1116.618	0.21	0
OsJN01549	TIGR	9638.m02653	HP	LH	15551443	-	3	704	A	525.496	0.077	0
OsJN01550	TIGR	9638.m02375	PP	LH	14106078	-	2	878	P	1803.811	0.2	0.5
OsJN01551	TIGR	9638.m02726	HP	LH	16015168	-	1	766	P	2056.675	0.389	0
OsJN01552	TIGR	9638.m02786	HP	LH	16373485	-	1	1431	P	860.605	0.167	0
OsJN01553	TIGR	9638.m03442	EP	HH	19980587	-	1	711	P	1471.225	1	0
OsJN01554	TIGR	9638.m03407	PP	HH	19831806	-	5	4353	P	3185.268	0.161	0.023
OsJN01555	TIGR	9638.m03406	HP	LH	19824435	-	2	1178	P	1633.846	0.083	0.071
OsJN01556	TIGR	9638.m03123	EP	LH	18382440	-	1	678	P	2607.772	0.25	0
OsJN01557	TIGR	9638.m03024	HP	HH	17818028	-	1	1481	P	2788.515	0.406	0
OsJN01558	TIGR	9638.m02329	HP	LH	13822993	-	12	4670	P	1348.437	0.125	0.058
OsJN01559	TIGR	9638.m03196	PP	HH	18729323	-	11	2929	P	1444.415	0.125	0.032
OsJN01560	TIGR	9638.m02510	PP	HH	14779227	-	1	1206	P	1212.565	0.192	0
OsJN01561	TIGR	9638.m03784	PP	HH	21797911	-	1	4829	A	1	0	0
OsJN01562	TIGR	9638.m02862	EP	LH	16691618	-	4	3317	P	1929.736	0.179	0
OsJN01563	TIGR	9638.m02552	HP	HH	14999521	-	2	945	P	3889.565	0.125	0.077

OsJN01564	TIGR	9638.m01960	HP	LH	11519873	-	2	708	P	2382.993	0.111	0.5
OsJN01565	TIGR	9638.m03530	PP	HH	20344978	-	5	3406	P	1238.829	0.4	0.16
OsJN01566	TIGR	9638.m03957	PP	HH	17811154	-	8	2230	P	1511.832	0.147	0.063
OsJN01567	TIGR	9638.m03925	PP	HH	22576527	-	8	2643	P	1618.101	0.216	0
OsJN01568	TIGR	9638.m02331	HP	LH	13829919	-	3	955	P	1495.556	0.118	0.25
OsJN01569	TIGR	9638.m03218	HP	LH	18850136	-	2	1084	P	1654.685	0.333	0
OsJN01570	TIGR	9638.m02141	EP	HH	12588335	-	9	5623	P	1980.083	0.089	0.026
OsJN01571	TIGR	9638.m03704	PP	HH	21381479	-	48	19169	P	735.329	0.051	0.045
OsJN01572	TIGR	9638.m02300	HP	LH	13638913	-	2	967	P	1020.254	0.4	0.118
OsJN01573	TIGR	9638.m02281	HP	LH	13437401	-	3	883	P	1879.142	0.294	0
OsJN01574	TIGR	9638.m03124	PP	LH	18384529	-	4	3257	P	1961.63	0.364	0.093
OsJN01575	TIGR	9638.m01991	HP	LH	11732973	-	2	1486	P	1263.55	0.063	0
OsJN01576	TIGR	9638.m03098	EP	LH	18223936	-	1	553	P	1337.643	0.364	0
OsJN01577	TIGR	9638.m02626	HP	HH	15398831	-	2	3256	P	1052.827	0.222	0.119
OsJN01578	TIGR	9638.m02908	EP	HH	16984172	-	6	5907	P	1191.184	0.118	0.114
OsJN01579	TIGR	9638.m02561	HP	LH	15031993	-	2	646	A	1	0	0
OsJN01580	TIGR	9638.m02485	PP	HH	14691136	-	3	1849	A	0	0	0
OsJN01581	TIGR	9638.m02420	HP	LH	14347812	-	1	339	P	1193.88	0.5	0
OsJN01582	TIGR	9638.m02271	PP	HH	13364368	-	9	2096	P	1165.657	0.5	0.071
OsJN01583	TIGR	9638.m02217	EP	HH	12994506	-	4	2130	P	1492.701	0.833	0
OsJN01584	TIGR	9638.m03279	HP	LH	19180453	-	2	813	P	1215.075	0.235	0
OsJN01585	TIGR	9638.m02128	PP	LH	12514448	-	8	4697	P	1530.925	0.133	0.024
OsJN01586	TIGR	9638.m03039	EP	LH	17903310	-	4	3599	P	1981.242	0.182	0.056
OsJN01587	TIGR	9638.m02183	HP	LH	12836639	-	1	363	P	2308.192	0.222	0
OsJN01588	TIGR	9638.m03605	PP	HH	20808646	-	4	3829	P	1859.334	0.143	0.122
OsJN01589	TIGR	9638.m02968	EP	LH	17459747	-	4	3259	P	1515.761	0.292	0.043
OsJN01590	TIGR	9638.m02933	PP	HH	17172652	-	2	2297	P	866.851	0.146	0
OsJN01591	TIGR	9638.m02309	HP	LH	13723231	-	1	384	P	1625.258	0.75	0
OsJN01592	TIGR	9638.m02608	EP	HH	15288999	-	2	2739	P	2170.441	0.176	0.053
OsJN01593	TIGR	9638.m02493	HP	LH	14720747	-	4	1435	P	3885.924	0.167	0.05
OsJN01594	TIGR	9638.m03829	HP	LH	22049426	-	1	447	P	1378.772	0.273	0
OsJN01595	TIGR	9638.m02280	HP	LH	13435035	-	3	1652	P	1287.603	0.1	0.074
OsJN01596	TIGR	9638.m01955	HP	LH	11497322	-	1	279	P	2914.575	0.714	0
OsJN01597	TIGR	9638.m02851	HP	HH	16652217	-	1	1821	P	4467.253	0.154	0
OsJN01598	TIGR	9638.m02842	EP	HH	16611277	-	7	3443	P	2092	0.432	0.128
OsJN01599	TIGR	9638.m03025	EP	LH	17820181	-	16	10658	P	862.562	0.116	0.053
OsJN01600	TIGR	9638.m03895	PP	HH	22440721	-	1	1461	P	2199.164	0.125	0
OsJN01601	TIGR	9638.m03184	PP	HH	18670812	-	2	2300	P	1995.013	0.417	0.038
OsJN01602	TIGR	9638.m02646	EP	HH	15521767	-	1	2011	P	1253.882	0.295	0
OsJN01603	TIGR	9638.m01945	HP	LH	11427570	-	6	2429	P	1794.393	0.154	0.158
OsJN01604	TIGR	9638.m02812	PP	HH	16480727	-	6	2205	P	980.803	0.52	0
OsJN01605	TIGR	9638.m02757	PP	HH	16192834	-	6	3943	P	681.965	0.259	0.052
OsJN01606	TIGR	9638.m03813	PP	HH	21979257	-	8	4095	P	1462.012	0.133	0
OsJN01607	TIGR	9638.m02807	HP	LH	16468802	-	3	1965	P	1195.42	0.154	0.107
OsJN01608	TIGR	9638.m02753	PP	HH	16165087	-	10	7585	P	1441.555	0.27	0.169
OsJN01609	TIGR	9638.m03517	EP	HH	20293390	-	3	2438	P	1543.305	0.277	0
OsJN01610	TIGR	9638.m03359	EP	HH	19592659	-	1	944	P	1182.445	0.19	0
OsJN01611	TIGR	9638.m02990	HP	LH	17672010	-	5	3354	P	1693.257	0.073	0.061
OsJN01612	TIGR	9638.m02850	EP	HH	16640208	-	2	4886	P	2338.305	0.104	0.035
OsJN01613	TIGR	9638.m03679	PP	HH	21204045	-	11	3992	P	829.03	0.122	0.079
OsJN01614	TIGR	9638.m03729	PP	HH	21509633	-	4	1715	P	1454.472	0.069	0
OsJN01615	TIGR	9638.m03504	HP	HH	20226789	-	1	666	P	1753.707	0.375	0
OsJN01616	TIGR	9638.m03838	HP	LH	22080901	-	3	2004	P	1517.242	0.4	0
OsJN01617	TIGR	9638.m03539	EP	LH	20426682	-	6	4965	P	1260.997	0.188	0.074
OsJN01618	TIGR	9638.m03083	EP	LH	18156742	-	1	687	P	1579.279	0.188	0
OsJN01619	TIGR	9638.m02396	PP	HH	14246098	-	3	3322	P	1546.039	0.231	0.071

OsJN01620	TIGR	9638.m03883	EP	HH	22358853	-	13	6921	P	1268.833	0.129	0.015
OsJN01621	TIGR	9638.m03287	PP	HH	19230802	-	2	1195	P	984.137	0.174	0
OsJN01622	TIGR	9638.m03652	PP	HH	21092086	-	10	4217	P	1543.243	0.176	0.053
OsJN01623	TIGR	9638.m03602	PP	HH	20795743	-	3	3025	P	1194.373	0.195	0.08
OsJN01624	TIGR	9638.m02295	PP	HH	13589387	-	2	2181	A	1	0	0
OsJN01625	TIGR	9638.m03396	HP	LH	19775359	-	1	192	P	4617.381	0.25	0
OsJN01626	TIGR	9638.m03207	PP	HH	18798644	-	1	303	P	4350.833	0.286	0
OsJN01627	TIGR	9638.m03441	EP	HH	19979593	-	1	744	P	7579.448	1	0
OsJN01628	TIGR	9638.m03716	PP	HH	21476170	-	4	1831	P	1615.773	0.25	0
OsJN01629	TIGR	9638.m03075	HP	LH	18103944	-	2	776	P	1677.948	0.308	0
OsJN01630	TIGR	9638.m02479	PP	HH	14668586	-	1	1566	P	1290.379	0.147	0
OsJN01631	TIGR	9638.m03618	PP	HH	20886888	-	9	9707	P	1177.24	0.067	0.033
OsJN01632	TIGR	9638.m02517	EP	HH	14803169	-	3	5402	P	1139.441	0.216	0.169
OsJN01633	TIGR	9638.m03483	PP	HH	20119517	-	2	1058	P	2346.569	0.304	0
OsJN01634	TIGR	9638.m03820	HP	HH	22008059	-	1	339	P	1335.736	0.429	0
OsJN01635	TIGR	9638.m02834	PP	HH	16575370	-	3	1882	A	0	0	0.048
OsJN01636	TIGR	9638.m03365	HP	LH	19610977	-	2	481	P	947.805	0.1	0
OsJN01637	TIGR	9638.m03148	PP	HH	18510156	-	1	3120	P	1667.234	0.212	0
OsJN01638	TIGR	9638.m03234	PP	LH	18912750	-	9	3075	P	1789.191	0.2	0.03
OsJN01639	TIGR	9638.m02005	HP	LH	11792974	-	2	809	P	2062.894	0.333	0.111
OsJN01640	TIGR	9638.m01972	EP	HH	11586196	-	8	8387	A	1	0	0
OsJN01641	TIGR	9638.m03340	HP	LH	19509169	-	1	267	A	0	0	0
OsJN01642	TIGR	9638.m02679	HP	LH	15758780	-	1	564	P	2154.644	0.308	0
OsJN01643	TIGR	9638.m03494	PP	HH	20157825	-	4	2551	P	1272.816	0.108	0
OsJN01644	TIGR	9638.m02711	HP	LH	15955817	-	2	655	P	3897.321	0.615	1
OsJN01645	TIGR	9638.m02897	HP	LH	16892616	-	3	2210	P	1673.599	0.5	0
OsJN01646	TIGR	9638.m02474	HP	LH	14640985	-	4	1317	A	521.729	0.1	0.071
OsJN01647	TIGR	9638.m02408	EP	HH	14275752	-	7	2529	P	857.857	0.161	0.04
OsJN01648	TIGR	9638.m02174	PP	LH	12781479	-	1	1422	P	1091.902	0.161	0
OsJN01649	TIGR	9638.m03116	HP	HH	18345886	-	4	2255	P	1428.3	0.118	0.077
OsJN01650	TIGR	9638.m01992	HP	HH	11735405	-	2	1959	A	0	0	0.091
OsJN01651	TIGR	9638.m03783	EP	HH	21791798	-	9	2909	P	1243.154	0.205	0.08
OsJN01652	TIGR	9638.m03730	PP	HH	21512271	-	5	2755	P	1100.528	0.12	0.088
OsJN01653	TIGR	9638.m02427	EP	HH	14396408	-	2	3790	P	1701.628	0.182	0
OsJN01654	TIGR	9638.m02558	HP	LH	15023563	-	1	855	P	1265.161	0.167	0
OsJN01655	TIGR	9638.m03623	HP	HH	20924133	-	3	1274	A	0	0	0.071
OsJN01656	TIGR	9638.m02483	PP	HH	14682063	-	1	1188	P	1823.811	0.222	0
OsJN01657	TIGR	9638.m02574	PP	HH	15092332	-	2	1123	A	798.373	0.05	0
OsJN01658	TIGR	9638.m03879	PP	LH	22318524	-	5	1288	P	2079.43	0.182	0
OsJN01659	TIGR	9638.m03816	EP	LH	21994989	-	1	1251	P	1325.961	0.269	0
OsJN01660	TIGR	9638.m02918	HP	LH	17071436	-	2	767	P	973.362	0.615	0
OsJN01661	TIGR	9638.m02963	EP	LH	17405575	-	9	4369	P	2207.464	0.208	0.088
OsJN01662	TIGR	9638.m03943	PP	HH	22645790	-	15	4506	A	649.549	0.125	0.06
OsJN01663	TIGR	9638.m03175	PP	HH	18641278	-	1	1365	P	1127.93	0.1	0
OsJN01664	TIGR	9638.m02793	PP	LH	16409099	-	5	2436	P	1708.74	0.417	0.1
OsJN01665	TIGR	9638.m02361	PP	HH	14000081	-	2	3419	P	1510.243	0.152	0.125
OsJN01666	TIGR	9638.m02869	EP	HH	16726630	-	4	2013	P	2320.537	0.529	0.074
OsJN01667	TIGR	9638.m03096	HP	LH	18214054	-	2	440	P	1192.085	0.1	0
OsJN01668	TIGR	9638.m03699	PP	LH	21360069	-	4	2593	P	1601.098	0.227	0.036
OsJN01669	TIGR	9638.m03354	PP	HH	19567885	-	9	3374	P	1246.774	0.217	0
OsJN01670	TIGR	9638.m02269	HP	LH	13354773	-	1	396	P	2220.355	0.571	0
OsJN01671	TIGR	9638.m02289	HP	LH	13507066	-	4	3998	P	2133.239	0.125	0.039
OsJN01672	TIGR	9638.m03214	PP	HH	18827514	-	1	597	P	1644.345	0.071	0
OsJN01673	TIGR	9638.m03650	HP	HH	21082070	-	6	2601	P	1985.524	0.257	0
OsJN01674	TIGR	9638.m02861	EP	LH	16691618	-	4	3317	P	1929.736	0.179	0
OsJN01675	TIGR	9638.m02650	EP	HH	15535679	-	1	1692	P	780.857	0.25	0

OsJN01676	TIGR	9638.m03896	HP	LH	22444729	-	1	396	P	1451.678	0.222	0
OsJN01677	TIGR	9638.m03759	PP	HH	21671774	-	5	1971	P	1434.776	0.333	0
OsJN01678	TIGR	9638.m03338	PP	HH	19498250	-	2	3433	P	1052.508	0.308	0.031
OsJN01679	TIGR	9638.m02381	HP	LH	14133559	-	2	314	P	1399.086	0.75	0
OsJN01680	TIGR	9638.m02624	PP	HH	15384241	-	14	8638	P	2416.756	0.049	0.056
OsJN01681	TIGR	9638.m02297	HP	LH	13610929	-	1	243	P	1543.041	0.5	0
OsJN01682	TIGR	9638.m03926	PP	HH	22580662	-	5	2982	P	1386.499	0.364	0.048
OsJN01683	TIGR	9638.m03227	HP	LH	18899032	-	2	373	A	0	0	0
OsJN01684	TIGR	9638.m03037	PP	HH	17891413	-	1	2025	P	2208.984	0.116	0
OsJN01685	TIGR	9638.m02115	EP	LH	12434156	-	3	1490	A	681.123	0.267	0.111
OsJN01686	TIGR	9638.m03682	PP	HH	21221496	-	7	2747	P	1791.678	0.182	0
OsJN01687	TIGR	9638.m02740	EP	LH	16098761	-	1	834	P	2050.991	0.526	0
OsJN01688	TIGR	9638.m03003	PP	HH	17739712	-	6	2787	P	2382.637	0.45	0.15
OsJN01689	TIGR	9638.m03790	PP	HH	21821576	-	4	2973	P	1542.987	0.189	0.074
OsJN01690	TIGR	9638.m03163	HP	LH	18586969	-	2	847	A	0	0	0
OsJN01691	TIGR	9638.m02109	EP	HH	12388005	-	2	2333	P	2725.433	0.368	0.037
OsJN01692	TIGR	9638.m02971	HP	LH	17476418	-	3	1371	P	1635.34	0.455	0.091
OsJN01693	TIGR	9638.m03109	PP	HH	18297783	-	18	7334	P	1710.137	0.123	0.117
OsJN01694	TIGR	9638.m03868	PP	HH	22269980	-	1	738	A	0	0	0
OsJN01695	TIGR	9638.m02891	PP	HH	16857690	-	1	3205	A	613.485	0.071	0
OsJN01696	TIGR	9638.m03634	PP	HH	20970659	-	2	3647	P	1754.137	0.325	0
OsJN01697	TIGR	9638.m03240	HP	LH	18961197	-	4	4192	A	678.967	0.273	0.104
OsJN01698	TIGR	9638.m02614	HP	LH	15322898	-	2	381	P	2457.296	0.111	0
OsJN01699	TIGR	9638.m02551	PP	HH	14996114	-	2	2470	P	826.869	0.118	0
OsJN01700	TIGR	9638.m03205	PP	HH	18787073	-	1	303	P	3415.738	0.286	0
OsJN01701	TIGR	9638.m03733	EP	HH	21521229	-	16	10884	P	1043.689	0.172	0.071
OsJN01702	TIGR	9638.m03313	PP	HH	19360644	-	2	2268	P	1434.749	0.182	0.167
OsJN01703	TIGR	9638.m03274	PP	HH	19153326	-	3	4223	A	1	0	0
OsJN01704	TIGR	9638.m02629	PP	HH	15420399	-	5	2854	P	2341.531	0.194	0.192
OsJN01705	TIGR	9638.m02095	PP	HH	12311670	-	2	3121	P	1184.776	0.167	0
OsJN01706	TIGR	9638.m03776	HP	LH	21758933	-	1	639	P	1484.004	0.333	0
OsJN01707	TIGR	9638.m02429	PP	HH	14403836	-	4	7212	P	1095.312	0.119	0
OsJN01708	TIGR	9638.m02338	HP	LH	13892186	-	1	417	P	2529.111	0.9	0
OsJN01709	TIGR	9638.m03595	PP	LH	20724365	-	4	1444	P	2039.961	0.333	0.188
OsJN01710	TIGR	9638.m03349	HP	LH	19534891	-	1	327	P	2229.074	0.125	0
OsJN01711	TIGR	9638.m02193	PP	HH	12884574	-	1	1464	P	1367.356	0.25	0
OsJN01712	TIGR	9638.m03685	PP	HH	21254839	-	2	4425	P	2673.444	0.216	0.045
OsJN01713	TIGR	9638.m01954	HP	LH	11492774	-	1	210	P	1145.714	0.6	0
OsJN01714	TIGR	9638.m03934	PP	HH	22615028	-	8	4960	P	1328.296	0.167	0.11
OsJN01715	TIGR	9638.m02165	HP	LH	12758600	-	1	246	P	1557.29	0.333	0
OsJN01716	TIGR	9638.m03889	PP	HH	22407527	-	12	5734	P	1706.928	0.244	0.054
OsJN01717	TIGR	9638.m03597	PP	HH	20728661	-	3	1486	P	1425.87	0.393	0
OsJN01718	TIGR	9638.m03183	HP	LH	18668587	-	2	994	P	1690.815	0.25	0.4
OsJN01719	TIGR	9638.m01934	HP	LH	11359888	-	2	1422	P	1780.301	0.172	0
OsJN01720	TIGR	9638.m03569	HP	LH	20581139	-	3	937	P	4263.124	0.417	0.444
OsJN01721	TIGR	9638.m03426	PP	HH	19929070	-	2	1260	P	1379.505	0.111	0
OsJN01722	TIGR	9638.m02988	HP	HH	17650344	-	10	5686	P	1627.057	0.219	0.046
OsJN01723	TIGR	9638.m03026	HP	HH	17832213	-	8	6631	P	1876.854	0.022	0.073
OsJN01724	TIGR	9638.m02042	EP	LH	11976664	-	6	4498	P	1434.276	0.156	0.077
OsJN01725	TIGR	9638.m03062	PP	HH	18044260	-	4	963	A	0	0	0
OsJN01726	TIGR	9638.m02098	HP	LH	12329935	-	3	1014	P	2890.212	0.455	0.429
OsJN01727	TIGR	9638.m03224	PP	HH	18871259	-	15	5383	P	1047.196	0.145	0.119
OsJN01728	TIGR	9638.m01953	HP	LH	11489788	-	2	782	A	0	0	0.143
OsJN01729	TIGR	9630.m04098	HP	LH	15938929	-	2	845	A	1	0	0
OsJN01730	TIGR	9638.m03256	HP	LH	19048255	-	2	853	P	2641.109	0.333	0.154
OsJN01731	TIGR	9638.m03765	HP	LH	21703705	-	3	1283	P	1684.125	0.143	0

OsJN01732	TIGR	9638.m02104	HP	LH	12357889	-	3	1104	A	0	0	0
OsJN01733	TIGR	9638.m01995	PP	HH	11756611	-	1	1581	P	1293.081	0.143	0
OsJN01734	TIGR	9638.m03665	PP	LH	21152443	-	1	872	P	1707.264	0.15	0
OsJN01735	TIGR	9638.m03104	EP	HH	18253244	-	6	3900	P	1695.656	0.324	0.043
OsJN01736	TIGR	9638.m03728	HP	LH	21504150	-	2	787	P	1388.985	0.364	0.429
OsJN01737	TIGR	9638.m01977	HP	LH	11615579	-	1	588	A	1	0	0
OsJN01738	TIGR	9638.m03048	PP	HH	17937849	-	2	3597	P	2206.992	0.212	0.022
OsJN01739	TIGR	9638.m03522	PP	HH	20322197	-	2	2137	P	1471.249	0.079	0
OsJN01740	TIGR	9638.m02395	EP	HH	14239163	-	3	2849	P	2475.461	0.379	0.097
OsJN01741	TIGR	9638.m02333	EP	HH	13839302	-	26	11469	P	1170.287	0.084	0.041
OsJN01742	TIGR	9638.m03805	EP	HH	21945959	-	1	186	P	11403.093	1	0
OsJN01743	TIGR	9638.m02150	PP	HH	12648115	-	7	3111	P	1067.753	0.233	0.086
OsJN01744	TIGR	9638.m03758	PP	HH	21671774	-	5	1971	P	1434.776	0.333	0
OsJN01745	TIGR	9638.m03277	HP	LH	19173133	-	1	687	P	1507.54	0.375	0
OsJN01746	TIGR	9638.m03121	PP	LH	18371529	-	5	3804	P	1411.294	0.289	0.133
OsJN01747	TIGR	9638.m03115	PP	HH	18336802	-	8	5459	P	891.092	0.171	0
OsJN01748	TIGR	9638.m03233	PP	LH	18912700	-	9	3125	P	1789.191	0.2	0.029
OsJN01749	TIGR	9638.m02747	HP	LH	16138228	-	2	1758	P	2055.934	0.667	0.1
OsJN01750	TIGR	9638.m02727	HP	LH	16018306	-	1	582	P	2707.38	0.538	0
OsJN01751	TIGR	9638.m03518	PP	HH	20298813	-	4	4705	P	1355.236	0.296	0.063
OsJN01752	TIGR	9638.m02020	PP	LH	11865354	-	4	4471	P	1570.94	0.02	0.022
OsJN01753	TIGR	9638.m03827	PP	HH	22041917	-	2	3892	P	852.399	0.043	0.059
OsJN01754	TIGR	9638.m03955	EP	HH	22689528	-	4	2530	P	1026.302	0.29	0.043
OsJN01755	TIGR	9638.m03632	PP	HH	20964670	-	8	4553	P	1442.253	0.078	0.043
OsJN01756	TIGR	9638.m02377	HP	LH	14112611	-	3	2583	P	1899.082	0.333	0
OsJN01757	TIGR	9638.m03657	PP	LH	21109766	-	2	673	P	1318.376	0.111	0.429
OsJN01758	TIGR	9638.m01967	EP	HH	11574214	-	5	2196	P	1234.391	0.063	0
OsJN01759	TIGR	9638.m03908	PP	HH	22502605	-	2	2122	P	1761.598	0.516	0.133
OsJN01760	TIGR	9638.m02876	PP	HH	16766526	-	2	5366	P	1324.392	0.2	0.05
OsJN01761	TIGR	9638.m03362	HP	LH	19601510	-	7	1958	A	370.844	0.167	0
OsJN01762	TIGR	9638.m01984	HP	LH	11671252	-	2	1069	A	1	0	0
OsJN01763	TIGR	9638.m02591	HP	HH	15197561	-	3	1747	P	1243.969	0.278	0.5
OsJN01764	TIGR	9638.m02171	HP	LH	12775283	-	1	492	A	671.681	0.091	0
OsJN01765	TIGR	9638.m02262	HP	LH	13309262	-	2	1494	P	1894.21	0.333	0.111
OsJN01766	TIGR	9638.m02426	PP	HH	14388122	-	2	3550	P	1571.445	0.127	0
OsJN01767	TIGR	9638.m03125	HP	LH	18389534	-	3	1357	P	2199.462	0.2	0.095
OsJN01768	TIGR	9638.m02512	HP	HH	14789613	-	4	2603	P	1788.382	0.323	0.318
OsJN01769	TIGR	9638.m02922	HP	LH	17090636	-	4	937	P	1069.991	0.25	0.222
OsJN01770	TIGR	9638.m03782	PP	HH	21787004	-	5	3622	P	682.45	0.205	0.051
OsJN01771	TIGR	9638.m03499	EP	HH	20189641	-	3	3649	P	2240.881	0.163	0.028
OsJN01772	TIGR	9638.m02317	EP	HH	13758451	-	8	2653	P	844.089	0.233	0.071
OsJN01773	TIGR	9638.m03676	PP	HH	21194112	-	1	730	P	1272.498	0.429	0
OsJN01774	TIGR	9638.m03493	EP	LH	20155534	-	3	1843	P	2249.694	0.389	0.786
OsJN01775	TIGR	9638.m03263	HP	LH	19090832	-	2	1252	P	1196.054	0.143	0.231
OsJN01776	TIGR	9638.m03161	PP	HH	18581551	-	2	746	P	1235.397	0.313	0
OsJN01777	TIGR	9638.m03714	PP	HH	21465986	-	4	1577	P	858.772	0.111	0
OsJN01778	TIGR	9638.m03775	PP	HH	21755012	-	4	2486	P	1854.466	0.421	0
OsJN01779	TIGR	9638.m02013	HP	LH	11833695	-	2	842	A	0	0	0.25
OsJN01780	TIGR	9638.m02848	EP	LH	16631195	-	1	783	P	2507.032	0.118	0
OsJN01781	TIGR	9638.m02804	HP	LH	16456509	-	2	1386	A	0	0	0.067
OsJN01782	TIGR	9638.m03873	HP	LH	22286742	-	2	632	P	1287.371	0.667	0.125
OsJN01783	TIGR	9638.m02952	EP	LH	17275534	-	2	978	P	1907.805	0.45	0
OsJN01784	TIGR	9638.m03330	HP	HH	19452034	-	2	2335	P	1974.229	0.2	0
OsJN01785	TIGR	9638.m02145	EP	HH	12610015	-	4	2479	P	1385.3	0.297	0
OsJN01786	TIGR	9638.m02462	HP	LH	14581908	-	2	1276	A	640.413	0.5	0
OsJN01787	TIGR	9638.m03211	HP	LH	18810256	-	3	951	P	3752.768	0.125	0.167

OsJN01788	TIGR	9638.m02738	EP	HH	16089571	-	2	840	P	851.118	0.211	0
OsJN01789	TIGR	9638.m03326	EP	HH	19412772	-	1	1266	P	1478.812	0.357	0
OsJN01790	TIGR	9638.m03817	HP	LH	21999681	-	1	234	P	1691.239	0.5	0
OsJN01791	TIGR	9638.m03687	EP	LH	21284689	-	5	2619	P	2058.196	0.116	0
OsJN01792	TIGR	9638.m03066	HP	LH	18064055	-	1	489	P	1974.834	0.25	0
OsJN01793	TIGR	9638.m02205	HP	HH	12946456	-	4	1729	P	1137.337	0.188	0
OsJN01794	TIGR	9638.m02934	HP	LH	17176586	-	2	730	P	2064.129	0.286	0
OsJN01795	TIGR	9638.m02698	HP	LH	15895592	-	2	4211	P	1936.485	0.65	0.076
OsJN01796	TIGR	9638.m02638	PP	HH	15476377	-	6	3421	P	1837.68	0.137	0.211
OsJN01797	TIGR	9638.m03939	HP	LH	22640020	-	4	1553	P	1642.244	0.25	0
OsJN01798	TIGR	9638.m03686	PP	HH	21271271	-	7	2323	P	822.168	0.179	0.077
OsJN01799	TIGR	9638.m03350	HP	LH	19542012	-	2	1302	P	1645.366	0.148	0
OsJN01800	TIGR	9638.m03159	PP	HH	18570269	-	3	1945	P	1647.704	0.19	0
OsJN01801	TIGR	9638.m03090	EP	HH	18182038	-	3	2591	P	2203.776	0.213	0.111
OsJN01802	TIGR	9638.m03914	PP	HH	22532404	-	7	4368	P	1279.093	0.435	0.031
OsJN01803	TIGR	9638.m02140	EP	HH	12588735	-	9	5223	P	2237.018	0.081	0.026
OsJN01804	TIGR	9638.m01956	HP	LH	11501918	-	4	3765	P	2677.189	0.13	0.296
OsJN01805	TIGR	9638.m03721	PP	HH	21486268	-	3	1588	P	663.788	0.2	0.4
OsJN01806	TIGR	9638.m03575	HP	LH	20630821	-	2	945	P	1736.178	0.4	0.167
OsJN01807	TIGR	9638.m02339	EP	HH	13896421	-	2	1502	P	3159.258	0.087	0
OsJN01808	TIGR	9638.m03712	PP	HH	21462480	-	1	870	P	1012.101	0.211	0
OsJN01809	TIGR	9638.m03752	HP	LH	21631985	-	4	1805	A	475.469	0.143	0.188
OsJN01810	TIGR	9638.m03624	PP	HH	20931281	-	9	3818	P	2654.679	0.034	0.057
OsJN01811	TIGR	9638.m02094	HP	LH	12289994	-	4	1050	A	414.744	0.077	0.1
OsJN01812	TIGR	9638.m02741	EP	LH	16107081	-	1	713	P	2300.446	0.625	0
OsJN01813	TIGR	9638.m02539	HP	LH	14935866	-	4	13298	P	711.806	0.182	0.163
OsJN01814	TIGR	9638.m02845	PP	HH	16619916	-	10	4091	P	1116.618	0.21	0
OsJN01815	TIGR	9638.m02166	PP	HH	12761141	-	2	1657	P	1558.667	0.294	0
OsJN01816	TIGR	9638.m03619	HP	LH	20900338	-	2	475	P	1417.388	0.429	0
OsJN01817	TIGR	9638.m02791	PP	HH	16395956	-	1	2118	P	1399.778	0.178	0
OsJN01818	TIGR	9638.m02759	PP	HH	16204224	-	11	3545	P	1623.579	0.314	0.122
OsJN01819	TIGR	9638.m03625	PP	HH	20937329	-	10	3355	P	1400.521	0.167	0.026
OsJN01820	TIGR	9638.m03284	HP	LH	19211395	-	1	252	P	927.564	0.333	0
OsJN01821	TIGR	9638.m03598	EP	LH	20741788	-	4	2358	P	1391.435	0.212	0.143
OsJN01822	TIGR	9638.m02762	HP	LH	16241227	-	2	581	A	535.932	0.167	0
OsJN01823	TIGR	9638.m02076	HP	LH	12201727	-	5	4625	P	685.399	0.192	0.108
OsJN01824	TIGR	9638.m03635	PP	HH	20976487	-	7	2591	P	1217.247	0.033	0.083
OsJN01825	TIGR	9638.m03290	EP	HH	19253847	-	22	9768	P	1215.808	0.165	0.016
OsJN01826	TIGR	9638.m03151	PP	HH	18532943	-	6	2436	P	1486.933	0.242	0.2
OsJN01827	TIGR	9638.m02998	PP	HH	17713613	-	3	3220	P	1380.28	0.353	0.029
OsJN01828	TIGR	9638.m02921	EP	HH	17084212	-	2	1572	A	620.679	0.194	0
OsJN01829	TIGR	9638.m03512	EP	HH	20276682	-	8	3976	P	1167.104	0.118	0.139
OsJN01830	TIGR	9638.m03236	PP	HH	18928208	-	13	8703	P	1264.395	0.107	0.025
OsJN01831	TIGR	9638.m03182	PP	HH	18665380	-	3	2486	P	1042.471	0.321	0
OsJN01832	TIGR	9638.m02135	EP	LH	12567049	-	2	1125	P	1467.2	0.571	0
OsJN01833	TIGR	9638.m03852	EP	LH	22172770	-	3	878	A	0	0	0
OsJN01834	TIGR	9638.m03019	EP	HH	17800496	-	2	3307	P	1527.297	0.204	0.087
OsJN01835	TIGR	9638.m03732	PP	HH	21516775	-	4	3678	P	1660.445	0.145	0
OsJN01836	TIGR	9638.m03544	HP	LH	20466656	-	2	832	A	0	0	0
OsJN01837	TIGR	9638.m02245	EP	HH	13156023	-	2	1754	P	1731.898	0.216	1
OsJN01838	TIGR	9638.m02345	PP	HH	13925575	-	9	3995	P	1051.172	0.256	0.104
OsJN01839	TIGR	9638.m02602	HP	LH	15243470	-	5	2284	P	837.573	0.188	0.086
OsJN01840	TIGR	9638.m03861	PP	HH	22239089	-	6	3786	P	1738.609	0.282	0.116
OsJN01841	TIGR	9638.m03636	PP	HH	20979802	-	5	3561	P	2223.416	0.125	0.048
OsJN01842	TIGR	9638.m01966	PP	HH	11563731	-	13	5871	P	1051.054	0.283	0.099
OsJN01843	TIGR	9638.m03272	PP	HH	19128335	-	1	1015	P	1703.567	0.5	0

OsJN01844	TIGR	9638.m03311	PP	HH	19353708	-	1	1539	P	1568.147	0.121	0
OsJN01845	TIGR	9638.m02279	EP	HH	13430332	-	6	2122	P	1139.872	0.222	0.15
OsJN01846	TIGR	9638.m03711	PP	HH	21459462	-	1	801	P	979.376	0.167	0
OsJN01847	TIGR	9638.m02687	HP	LH	15812251	-	1	567	P	2420.362	0.385	0
OsJN01848	TIGR	9638.m02670	HP	HH	15665585	-	3	918	P	1346.169	0.111	0
OsJN01849	TIGR	9638.m02386	EP	HH	14181390	-	1	3108	P	1894.868	0.179	0
OsJN01850	TIGR	9638.m03395	EP	LH	19768950	-	1	528	P	800.942	0.538	0
OsJN01851	TIGR	9638.m03560	EP	HH	20516359	-	1	608	P	1961.136	0.455	0
OsJN01852	TIGR	9638.m03670	PP	HH	21167820	-	1	396	A	345.637	0.111	0
OsJN01853	TIGR	9638.m03822	HP	LH	22017283	-	7	4755	P	1700.943	0.188	0.077
OsJN01854	TIGR	9638.m02853	PP	HH	16658114	-	5	4254	P	1633.727	0.159	0.02
OsJN01855	TIGR	9638.m02176	PP	HH	12787128	-	24	13664	P	1646.171	0.129	0.081
OsJN01856	TIGR	9638.m02418	HP	LH	14335137	-	8	3384	P	5013.006	0.053	0.054
OsJN01857	TIGR	9638.m03141	PP	HH	18488369	-	5	1805	P	1997.376	0.074	0
OsJN01858	TIGR	9638.m02826	EP	LH	16527481	-	3	3897	P	1413.539	0.5	0.096
OsJN01859	TIGR	9638.m03523	HP	LH	20328041	-	2	1706	P	1693.623	0.176	0
OsJN01860	TIGR	9638.m02412	PP	HH	14301960	-	1	1944	P	944.824	0.163	0
OsJN01861	TIGR	9638.m02136	EP	LH	12577948	-	6	3159	P	1278.785	0.103	0
OsJN01862	TIGR	9638.m03342	HP	LH	19514353	-	1	237	P	1662.872	0.6	0
OsJN01863	TIGR	9638.m03289	EP	HH	19248713	-	10	3512	P	2085.846	0.225	0.056
OsJN01864	TIGR	9638.m03891	HP	HH	22421207	-	17	8531	P	1659.833	0.078	0.084
OsJN01865	TIGR	9638.m02007	PP	HH	11803869	-	5	3682	P	1391.276	0.243	0
OsJN01866	TIGR	9638.m03905	EP	HH	22483909	-	7	3041	P	1175.776	0.2	0.032
OsJN01867	TIGR	9638.m03181	PP	HH	18658235	-	3	3326	P	1372.882	0.367	0.043
OsJN01868	TIGR	9638.m03511	EP	HH	20276704	-	8	3954	P	1167.104	0.128	0.128
OsJN01869	TIGR	9638.m03429	HP	LH	19942341	-	1	483	P	1343.478	0.455	0
OsJN01870	TIGR	9638.m02690	HP	LH	15835518	-	5	4505	P	1470.588	0.308	0.037
OsJN01871	TIGR	9638.m02437	EP	LH	14459854	-	3	2293	P	1587.539	0.139	0
OsJN01872	TIGR	9638.m03656	HP	LH	21109250	-	2	321	P	3869.089	0.429	0
OsJN01873	TIGR	9638.m02172	PP	LH	12776586	-	3	1369	P	2081.982	0.267	0.133
OsJN01874	TIGR	9638.m02533	PP	HH	14901869	-	5	3254	P	1254.63	0.243	0.091
OsJN01875	TIGR	9638.m03537	PP	HH	20413225	-	1	939	P	1833.169	0.35	0
OsJN01876	TIGR	9638.m02131	HP	LH	12540180	-	3	4077	P	1666.033	0.318	0.089
OsJN01877	TIGR	9638.m03077	PP	HH	18122065	-	3	5036	P	1716.644	0.25	0
OsJN01878	TIGR	9638.m02332	HP	LH	13831347	-	3	704	P	1800.426	0.111	0
OsJN01879	TIGR	9638.m02401	EP	HH	14256393	-	5	2637	P	2440.856	0.071	0.1
OsJN01880	TIGR	9638.m02398	PP	HH	14245374	-	4	3722	P	1557.559	0.231	0.054
OsJN01881	TIGR	9638.m03764	PP	HH	21695395	-	4	3841	P	2028.14	0.207	0.154
OsJN01882	TIGR	9638.m03547	HP	LH	20463700	-	4	1612	P	1649.194	0.4	0.059
OsJN01883	TIGR	9638.m01970	HP	HH	11582180	-	3	1257	P	1752.496	0.208	0
OsJN01884	TIGR	9638.m03928	EP	HH	22585161	-	11	5313	P	1493.723	0.188	0.064
OsJN01885	TIGR	9638.m02789	PP	HH	16386944	-	3	3098	P	2134.173	0.244	0.524
OsJN01886	TIGR	9638.m03519	HP	LH	20309043	-	6	4955	P	1816.954	0.476	0.169
OsJN01887	TIGR	9638.m03329	HP	LH	19439048	-	2	1984	P	2625.337	0.182	0.148
OsJN01888	TIGR	9638.m02838	PP	LH	16594452	-	2	3333	P	960.629	0.207	0
OsJN01889	TIGR	9638.m02186	EP	HH	12850337	-	5	4233	P	1643.519	0.286	0.036
OsJN01890	TIGR	9638.m03929	PP	HH	22592338	-	14	4444	P	967.226	0.231	0.044
OsJN01891	TIGR	9638.m03751	PP	HH	21625546	-	3	3177	P	1701.387	0.531	0.081
OsJN01892	TIGR	9638.m03713	PP	HH	21464476	-	1	786	P	1264.317	0.167	0
OsJN01893	TIGR	9638.m02879	HP	LH	16789968	-	3	699	A	552.075	0.111	0
OsJN01894	TIGR	9638.m02810	HP	LH	16476340	-	2	1386	A	0	0	0
OsJN01895	TIGR	9638.m02478	PP	HH	14666045	-	1	1110	P	1071.87	0.042	0
OsJN01896	TIGR	9638.m02108	EP	HH	12382294	-	2	1898	P	1880.209	0.231	0.133
OsJN01897	TIGR	9638.m03361	HP	LH	19599263	-	3	1994	A	0	0	0.111
OsJN01898	TIGR	9638.m02749	HP	LH	16148089	-	2	1168	P	1905.891	0.267	0.091
OsJN01899	TIGR	9638.m03291	HP	LH	19270466	-	2	1176	A	0	0	0.053

OsJN01900	TIGR	9638.m03543	HP	LH	20446144	-	5	1333	P	1690.115	0.5	0.167
OsJN01901	TIGR	9638.m02957	EP	LH	17298660	-	2	1214	P	1880.82	0.083	0.077
OsJN01902	TIGR	9638.m02132	HP	LH	12549147	-	4	1713	A	0	0	0.2
OsJN01903	TIGR	9638.m02305	PP	HH	13668560	-	2	1544	P	1386.655	0.552	0.167
OsJN01904	TIGR	9638.m02181	HP	LH	12824458	-	2	1060	P	1144.309	0.5	0.053
OsJN01905	TIGR	9638.m03818	PP	HH	22001585	-	15	5441	A	469.449	0.111	0.047
OsJN01906	TIGR	9638.m03370	HP	HH	19637955	-	4	3879	P	1262.358	0.176	0.029
OsJN01907	TIGR	9638.m03576	PP	HH	20640755	-	10	3212	P	1334.804	0.128	0.038
OsJN01908	TIGR	9638.m03091	PP	LH	18186922	-	11	5469	P	1280.846	0.468	0.028
OsJN01909	TIGR	9638.m02655	PP	LH	15563122	-	1	375	P	2464.546	0.375	0
OsJN01910	TIGR	9638.m02318	HP	LH	13831347	-	3	704	P	1800.426	0.111	0
OsJN01911	TIGR	9638.m02781	PP	HH	16346677	-	3	1747	P	2257.231	0.417	0
OsJN01912	TIGR	9638.m02892	PP	HH	16864299	-	1	1788	A	412.379	0.103	0
OsJN01913	TIGR	9638.m03002	EP	HH	17730714	-	6	4855	P	1331.993	0.288	0.067
OsJN01914	TIGR	9638.m03262	HP	LH	19077581	-	4	3123	P	2940.833	0.4	0.235
OsJN01915	TIGR	9638.m02982	PP	HH	17601945	-	3	6091	P	1701.727	0.18	0.146
OsJN01916	TIGR	9638.m02609	EP	HH	15295549	-	2	3609	P	1755.216	0.057	0.073
OsJN01917	TIGR	9638.m03089	PP	HH	18169035	-	14	9023	P	1217.679	0.221	0.029
OsJN01918	TIGR	9638.m02421	HP	HH	14350071	-	3	2388	P	1578.282	0.1	0
OsJN01919	TIGR	9638.m03938	HP	LH	22638975	-	2	425	P	2503.889	0.375	0
OsJN01920	TIGR	9638.m03122	HP	LH	18379216	-	4	2457	P	2530.403	0.222	0.057
OsJN01921	TIGR	9638.m03692	HP	LH	21315454	-	2	1391	P	2417.782	0.556	0
OsJN01922	TIGR	9638.m02356	PP	HH	13972059	-	9	3981	P	1247.322	0.024	0.044
OsJN01923	TIGR	9638.m02268	PP	HH	13346577	-	12	3171	A	488.025	0.024	0.034
OsJN01924	TIGR	9638.m02935	PP	HH	17181920	-	1	1920	P	915.782	0.19	0
OsJN01925	TIGR	9638.m02560	HP	LH	15030937	-	1	411	P	1214.78	0.3	0
OsJN01926	TIGR	9638.m02553	HP	LH	15001649	-	2	1814	P	3016.921	0.417	0.148
OsJN01927	TIGR	9638.m02162	PP	HH	12717404	-	5	5894	P	1143.865	0.276	0.04
OsJN01928	TIGR	9638.m03915	PP	HH	22532404	-	6	4325	P	1260.58	0.431	0
OsJN01929	TIGR	9638.m02496	HP	LH	14728955	-	8	3366	P	806.776	0.32	0.041
OsJN01930	TIGR	9638.m03744	HP	LH	21600316	-	1	426	A	0	0	0
OsJN01931	TIGR	9638.m02841	HP	LH	16607282	-	1	441	P	2076.45	0.222	0
OsJN01932	TIGR	9638.m03666	PP	HH	21155137	-	1	793	P	1106.965	0.353	0
OsJN01933	TIGR	9638.m02820	EP	LH	16505302	-	1	684	P	1273.582	0.429	0
OsJN01934	TIGR	9638.m03680	PP	HH	21216592	-	2	829	A	684.869	0.154	0
OsJN01935	TIGR	9638.m02808	EP	HH	16470932	-	5	5158	P	1136.445	0.25	0.023
OsJN01936	TIGR	9638.m03016	HP	LH	17793737	-	2	2519	P	834.84	0.078	0
OsJN01937	TIGR	9638.m02852	HP	HH	16654775	-	1	1941	P	2161.573	0.143	0
OsJN01938	TIGR	9638.m02580	PP	HH	15137173	-	13	6463	P	1562.428	0.104	0.043
OsJN01939	TIGR	9638.m03410	HP	LH	19849264	-	4	2494	A	0	0	0.065
OsJN01940	TIGR	9638.m02927	PP	HH	17118461	-	3	4482	P	1351.199	0.18	0.023
OsJN01941	TIGR	9638.m03064	PP	HH	18052009	-	18	6376	P	1253.596	0.108	0.031
OsJN01942	TIGR	9638.m02353	HP	LH	13959862	-	2	812	P	783.365	0.333	0.5
OsJN01943	TIGR	9638.m02310	PP	HH	13725368	-	2	3783	P	812.592	0.13	0.222
OsJN01944	TIGR	9638.m02255	HP	LH	13262179	-	2	726	A	1	0	0
OsJN01945	TIGR	9638.m02224	PP	HH	13034780	-	18	4890	P	1406.022	0.207	0.063
OsJN01946	TIGR	9638.m02129	EP	LH	12527121	-	1	827	P	1054.517	0.389	0
OsJN01947	TIGR	9638.m03502	HP	LH	20216467	-	4	3334	P	1679.839	0.075	0.242
OsJN01948	TIGR	9638.m03488	HP	LH	20140120	-	1	456	P	2272.572	0.2	0
OsJN01949	TIGR	9638.m02801	HP	LH	16443127	-	2	449	P	1834.675	0.375	0.5
OsJN01950	TIGR	9638.m03347	PP	HH	19525769	-	4	2513	P	1048.724	0.684	0.083
OsJN01951	TIGR	9638.m02611	EP	HH	15305924	-	2	4365	P	1641.831	0.143	0.061
OsJN01952	TIGR	9638.m03819	PP	HH	22000492	-	16	6534	A	369.843	0.036	0.093
OsJN01953	TIGR	9638.m03708	EP	LH	21449392	-	1	587	P	2606.009	0.286	0
OsJN01954	TIGR	9638.m03946	EP	HH	22657524	-	6	1949	P	1699.91	0.136	0.118
OsJN01955	TIGR	9638.m03527	EP	LH	20334499	-	3	1477	P	1486.628	0.417	0

OsJN01956	TIGR	9638.m02482	HP	LH	14676371	-	3	848	P	886.27	0.231	0.8
OsJN01957	TIGR	9638.m02397	PP	HH	14246098	-	2	2998	P	1695.769	0.276	0.065
OsJN01958	TIGR	9638.m03763	HP	LH	21690189	-	1	783	P	1846.914	0.563	0
OsJN01959	TIGR	9638.m02137	EP	LH	12579352	-	4	1755	P	811.686	0.087	0
OsJN01960	TIGR	9638.m03779	EP	LH	21775740	-	6	2681	P	1253.522	0.333	0.071
OsJN01961	TIGR	9638.m03913	PP	HH	22532404	-	7	4368	P	1314.616	0.433	0.059
OsJN01962	TIGR	9638.m03033	HP	LH	17881376	-	2	980	P	2920.108	0.222	0
OsJN01963	TIGR	9638.m03849	PP	LH	22155356	-	2	2202	P	1625.254	0.4	0.292
OsJN01964	TIGR	9638.m02633	HP	LH	15444167	-	2	393	P	1346.083	0.5	0
OsJN01965	TIGR	9638.m03726	PP	HH	21492938	-	4	2874	P	1330.453	0.111	0.063
OsJN01966	TIGR	9638.m01985	EP	HH	11677280	-	2	1707	A	1	0	0
OsJN01967	TIGR	9638.m03417	PP	HH	19876644	-	10	6518	P	1444.408	0.264	0.02
OsJN01968	TIGR	9638.m03688	HP	LH	21290441	-	2	1744	A	365.469	0.25	0.143
OsJN01969	TIGR	9638.m02886	PP	HH	16829974	-	2	4217	A	599.206	0.064	0.143
OsJN01970	TIGR	9638.m03251	PP	HH	19027227	-	2	2164	P	1880.811	0.185	0
OsJN01971	TIGR	9638.m02240	EP	HH	13109058	-	2	2379	P	1482.86	0.313	0.056
OsJN01972	TIGR	9638.m03769	PP	HH	21727277	-	10	6077	P	1863.127	0.279	0.043
OsJN01973	TIGR	9638.m02702	HP	LH	15917390	-	3	1328	P	2592.655	0.438	0.167
OsJN01974	TIGR	9638.m02542	HP	LH	14946997	-	2	602	P	1628.157	0.143	0.333
OsJN01975	TIGR	9638.m02336	HP	LH	13867106	-	5	4608	P	727.372	0.083	0.056
OsJN01976	TIGR	9638.m03198	EP	LH	18744714	-	3	983	P	2541.399	0.167	0.1
OsJN01977	TIGR	9638.m02423	HP	LH	14363691	-	1	279	P	1535.77	0.286	0
OsJN01978	TIGR	9638.m03542	PP	HH	20438051	-	7	6977	P	1252.968	0.128	0.034
OsJN01979	TIGR	9638.m03626	EP	LH	20941395	-	1	1155	P	1887.874	0.12	0
OsJN01980	TIGR	9638.m03299	PP	LH	19304360	-	6	2848	P	1243.459	0.486	0.185
OsJN01981	TIGR	9638.m03796	PP	HH	21900383	-	2	3597	P	2115.624	0.2	0
OsJN01982	TIGR	9638.m03254	EP	LH	19036745	-	1	895	A	401.061	0.053	0
OsJN01983	TIGR	9638.m03921	PP	HH	22552954	-	11	3582	P	2057.24	0.075	0
OsJN01984	TIGR	9638.m03871	EP	HH	22278987	-	1	2454	P	1120.532	0.333	0
OsJN01985	TIGR	9638.m03079	PP	HH	18134494	-	1	702	P	1240.402	0.067	0
OsJN01986	TIGR	9638.m02773	EP	HH	16304971	-	4	4949	P	632.552	0.058	0.167
OsJN01987	TIGR	9638.m02293	HP	LH	13572092	-	4	1183	A	0	0	0.385
OsJN01988	TIGR	9638.m02015	HP	LH	11845271	-	3	3972	P	1191.73	0.095	0.231
OsJN01989	TIGR	9638.m02546	PP	HH	14966224	-	3	1645	P	2141.085	0.188	0.2
OsJN01990	TIGR	9638.m02097	HP	LH	12323958	-	8	4348	A	783.302	0.05	0.083
OsJN01991	TIGR	9638.m03566	EP	HH	20555181	-	3	3184	P	2056.538	0.227	0.25
OsJN01992	TIGR	9638.m03068	PP	HH	18071763	-	6	3023	P	1923.4	0.083	0
OsJN01993	TIGR	9638.m02178	HP	LH	12809723	-	1	156	A	0	0	0
OsJN01994	TIGR	9638.m02025	HP	HH	11891236	-	8	3164	P	1507.424	0.06	0
OsJN01995	TIGR	9638.m01939	HP	LH	11383532	-	1	151	P	992.774	0.25	0
OsJN01996	TIGR	9638.m02940	HP	LH	17215584	-	1	234	P	1709.287	0.5	0
OsJN01997	TIGR	9638.m03901	EP	LH	22461143	-	2	1537	P	1972.539	0.273	0
OsJN01998	TIGR	9638.m02909	EP	HH	16983997	-	7	6082	P	1191.184	0.124	0.095
OsJN01999	TIGR	9638.m02742	HP	LH	16113111	-	1	579	P	1658.205	0.615	0
OsJN02000	TIGR	9638.m03495	HP	LH	20165618	-	2	458	A	0	0	0
OsJN02001	TIGR	9638.m02948	EP	HH	17245892	-	11	3446	P	1054.234	0.258	0.023
OsJN02002	TIGR	9638.m03427	PP	HH	19931804	-	2	844	P	1301.427	0.176	0
OsJN02003	TIGR	9638.m02438	HP	LH	14474290	-	2	1084	P	1471.566	0.25	0.25
OsJN02004	TIGR	9638.m02967	HP	LH	17449125	-	6	7074	P	1220.249	0.091	0.033
OsJN02005	TIGR	9638.m03235	EP	LH	18922599	-	1	682	P	1051.783	0.357	0
OsJN02006	TIGR	9638.m02866	HP	LH	16712161	-	3	1111	P	1319.724	0.167	0.25
OsJN02007	TIGR	9638.m03127	HP	LH	18397641	-	1	423	P	819.776	0.111	0
OsJN02008	TIGR	9638.m03880	EP	HH	22326513	-	3	1749	P	2407.301	0.346	0.091
OsJN02009	TIGR	9638.m02372	HP	LH	14094237	-	1	309	P	752.41	0.429	0
OsJN02010	TIGR	9638.m03269	HP	HH	19114550	-	1	1128	P	1783.582	0.25	0
OsJN02011	TIGR	9638.m02803	PP	HH	16450173	-	6	6086	P	1405.358	0.162	0.032

OsJN02012	TIGR	9638.m02604	EP	HH	15254145	-	2	1017	P	1654.811	0.263	0
OsJN02013	TIGR	9638.m03615	EP	LH	20857122	-	1	967	P	1463.588	0.333	0
OsJN02014	TIGR	9638.m02368	PP	HH	14060085	-	2	4919	P	1698.476	0.278	0.077
OsJN02015	TIGR	9638.m03520	HP	LH	20314200	-	1	390	P	1058.483	0.286	0
OsJN02016	TIGR	9638.m02285	HP	LH	13462633	-	2	1041	A	0	0	0.056
OsJN02017	TIGR	9638.m03369	EP	HH	19632495	-	4	4333	P	1617.428	0.317	0.019
OsJN02018	TIGR	9638.m02903	PP	HH	16948268	-	10	5861	P	1404.155	0.19	0.015
OsJN02019	TIGR	9638.m02088	HP	HH	12266095	-	4	1486	P	1162.677	0.08	0
OsJN02020	TIGR	9638.m03845	HP	LH	22132043	-	1	819	P	1250.463	0.167	0
OsJN02021	TIGR	9638.m02111	EP	LH	12395280	-	4	2787	P	1303.853	0.273	0
OsJN02022	TIGR	9638.m02752	PP	HH	16165087	-	10	7585	P	1441.555	0.27	0.169
OsJN02023	TIGR	9638.m03691	EP	HH	21306334	-	1	1682	P	2347.634	0.143	0
OsJN02024	TIGR	9638.m02037	HP	LH	11956592	-	5	1541	A	685.899	0.08	0.167
OsJN02025	TIGR	9638.m03678	PP	HH	21204045	-	12	3992	P	829.03	0.115	0.086
OsJN02026	TIGR	9638.m03078	HP	LH	18127739	-	4	3285	P	1932.897	0.143	0.045
OsJN02027	TIGR	9638.m02782	HP	HH	16351306	-	1	1521	P	2089.657	0.121	0
OsJN02028	TIGR	9638.m02649	HP	LH	15531601	-	1	378	P	900.718	0.111	0
OsJN02029	TIGR	9638.m02476	HP	LH	14645542	-	2	919	P	1951.122	0.111	0.083
OsJN02030	TIGR	9638.m03807	PP	HH	21954773	-	4	3047	P	1501.181	0.15	0
OsJN02031	TIGR	9638.m03439	EP	HH	19976718	-	2	1380	P	4549.014	1	1
OsJN02032	TIGR	9638.m03014	HP	LH	17787150	-	2	386	A	0	0	0
OsJN02033	TIGR	9638.m02599	EP	HH	15233352	-	4	2828	A	791.945	0.029	0.038
OsJN02034	TIGR	9638.m03916	PP	HH	22532404	-	7	4325	P	1260.58	0.438	0
OsJN02035	TIGR	9638.m03315	HP	LH	19366904	-	2	354	P	1381.576	0.2	0.333
OsJN02036	TIGR	9638.m02163	PP	HH	12728668	-	38	23477	P	1335.367	0.064	0.032
OsJN02037	TIGR	9638.m03191	PP	HH	18700639	-	15	5201	P	2948.825	0.127	0.121
OsJN02038	TIGR	9638.m03907	PP	LH	22494796	-	1	1395	P	1485.987	0.433	0
OsJN02039	TIGR	9638.m03690	EP	HH	21298894	-	6	2483	A	480.459	0.091	0.1
OsJN02040	TIGR	9638.m03902	EP	LH	22461158	-	1	1522	P	1972.539	0.265	0
OsJN02041	TIGR	9638.m02322	EP	HH	13786225	-	3	2137	P	1344.538	0.13	0.042
OsJN02042	TIGR	9638.m02106	PP	HH	12366828	-	7	3151	P	1300.009	0.333	0.053
OsJN02043	TIGR	9638.m02447	PP	HH	14509932	-	3	1659	P	1088.807	0.172	0
OsJN02044	TIGR	9638.m01978	HP	LH	11618732	-	2	283	A	1	0	0
OsJN02045	TIGR	9638.m03811	PP	HH	21970564	-	9	3702	P	1056.453	0.098	0
OsJN02046	TIGR	9638.m03421	PP	HH	19891860	-	6	4403	A	693.826	0.073	0.056
OsJN02047	TIGR	9638.m02965	EP	HH	17422868	-	3	2691	P	1161.72	0.29	0.074
OsJN02048	TIGR	9638.m02916	HP	HH	17047234	-	1	363	P	1200.809	0.333	0
OsJN02049	TIGR	9638.m02164	PP	HH	12755717	-	4	818	P	1741.774	0.429	0.2
OsJN02050	TIGR	9638.m02656	EP	HH	15583742	-	4	2220	P	1495.949	0.4	0
OsJN02051	TIGR	9638.m01976	EP	HH	11609372	-	2	2866	A	1	0	0
OsJN02052	TIGR	9638.m03513	EP	HH	20276680	-	8	3978	P	1167.104	0.118	0.139
OsJN02053	TIGR	9638.m02744	HP	LH	16120401	-	1	576	P	3773.699	0.615	0
OsJN02054	TIGR	9638.m02618	HP	LH	15357452	-	2	1093	P	1253.104	0.25	0.118
OsJN02055	TIGR	9638.m03374	PP	HH	19658075	-	3	1443	P	839.785	0.263	0.231
OsJN02056	TIGR	9638.m03087	PP	HH	18165564	-	1	450	P	3204.652	0.364	0
OsJN02057	TIGR	9638.m02814	HP	LH	16488609	-	3	1965	A	735.905	0.231	0.103
OsJN02058	TIGR	9638.m02234	HP	HH	13085049	-	2	1308	A	571.574	0.077	0
OsJN02059	TIGR	9638.m02275	PP	HH	13402329	-	2	1523	P	1177.273	0.438	0
OsJN02060	TIGR	9638.m03833	PP	HH	22060426	-	11	4385	P	1505.571	0.258	0.033
OsJN02061	TIGR	9638.m03637	PP	HH	20983902	-	9	2899	A	562.13	0.061	0
OsJN02062	TIGR	9638.m02355	HP	LH	13967357	-	2	557	A	0	0	0.25
OsJN02063	TIGR	9638.m03114	PP	HH	18332443	-	1	2660	P	827.991	0.103	0
OsJN02064	TIGR	9638.m02984	HP	LH	17628823	-	2	584	P	1596.805	0.357	0
OsJN02065	TIGR	9638.m03201	PP	HH	18754755	-	4	2448	P	863.593	0.125	0.033
OsJN02066	TIGR	9638.m02340	HP	LH	13900470	-	1	471	P	1432.131	0.182	0
OsJN02067	TIGR	9638.m03945	EP	HH	22657987	-	4	1486	P	2270.809	0.2	0

OsJN02068	TIGR	9638.m02450	PP	HH	14519185	-	1	1188	P	1160.352	0.115	0
OsJN02069	TIGR	9638.m02821	HP	LH	16507116	-	5	3135	A	554.348	0.041	0.053
OsJN02070	TIGR	9638.m03032	HP	LH	17874652	-	2	2260	P	1291.656	0.35	0.074
OsJN02071	TIGR	9638.m03308	PP	HH	19337681	-	1	1848	P	1870.91	0.268	0
OsJN02072	TIGR	9638.m03630	HP	LH	20959416	-	2	982	P	2249.454	0.167	0.125
OsJN02073	TIGR	9638.m03709	PP	HH	21452746	-	1	1609	P	869.861	0.083	0
OsJN02074	TIGR	9638.m02709	HP	LH	15948965	-	3	620	P	1677.64	0.571	0
OsJN02075	TIGR	9638.m02334	PP	HH	13854269	-	11	3820	P	1319.187	0.26	0.03
OsJN02076	TIGR	9638.m03888	PP	HH	22408015	-	11	4867	P	1747.138	0.264	0.061
OsJN02077	TIGR	9638.m03418	PP	HH	19874096	-	11	8983	P	1642.755	0.254	0.045
OsJN02078	TIGR	9638.m03734	EP	HH	21536541	-	10	4057	P	1303.059	0.138	0.033
OsJN02079	TIGR	9638.m02724	HP	LH	16006935	-	1	579	P	1847.384	0.538	0
OsJN02080	TIGR	9638.m03036	PP	HH	17889034	-	2	4404	P	1300.395	0.288	0.071
OsJN02081	TIGR	9638.m02911	PP	LH	16999432	-	4	2900	P	2849.925	0.263	0.111
OsJN02082	TIGR	9638.m03582	PP	HH	20666398	-	8	5236	P	1279.51	0.305	0.074
OsJN02083	TIGR	9638.m03253	HP	LH	19031070	-	2	2160	P	1796.04	0.7	0.067
OsJN02084	TIGR	9638.m02855	HP	LH	16668529	-	2	2008	P	1373.961	0.357	0.071
OsJN02085	TIGR	9638.m03105	HP	LH	18268678	-	2	3056	P	1233.271	0.152	0.294
OsJN02086	TIGR	9638.m03700	HP	LH	21363826	-	1	759	P	2056.007	0.412	0
OsJN02087	TIGR	9638.m02435	EP	HH	14454289	-	3	5046	A	500.546	0.067	0.12
OsJN02088	TIGR	9638.m03346	PP	HH	19525769	-	2	896	P	1048.915	0.611	0
OsJN02089	TIGR	9638.m03292	HP	LH	19272664	-	4	862	P	2241.479	0.231	0.333
OsJN02090	TIGR	9638.m03252	PP	HH	19028488	-	1	903	P	1683.976	0.2	0
OsJN02091	TIGR	9638.m03210	HP	LH	18807637	-	1	675	P	1587.709	0.667	0
OsJN02092	TIGR	9638.m02669	HP	LH	15660634	-	4	2277	A	514.166	0.125	0.122
OsJN02093	TIGR	9638.m03249	PP	HH	19014022	-	16	8479	P	1731.588	0.052	0.022
OsJN02094	TIGR	9638.m02661	HP	LH	15614214	-	2	386	A	0	0	0.333
OsJN02095	TIGR	9638.m03321	PP	HH	19396169	-	2	1819	P	1322.683	0.405	0
OsJN02096	TIGR	9638.m03703	PP	HH	21381479	-	50	19169	P	735.329	0.05	0.046
OsJN02097	TIGR	9638.m02001	HP	LH	11782522	-	1	450	A	1	0	0
OsJN02098	TIGR	9638.m03897	PP	HH	22445958	-	1	1260	A	575.918	0.107	0
OsJN02099	TIGR	9638.m02625	EP	HH	15393335	-	2	1998	P	1905.363	0.303	0.1
OsJN02100	TIGR	9638.m03675	PP	HH	21190939	-	1	627	P	1482.284	0.286	0
OsJN02101	TIGR	9638.m02987	PP	HH	17645315	-	2	3397	P	3460.653	0.267	0.034
OsJN02102	TIGR	9638.m03870	PP	HH	22274300	-	3	2891	P	1282.673	0.314	0.036
OsJN02103	TIGR	9638.m03065	HP	LH	18059989	-	1	504	P	1192.203	0.25	0
OsJN02104	TIGR	9638.m02889	PP	HH	16844867	-	3	4128	P	1015.259	0.077	0.05
OsJN02105	TIGR	9638.m02286	HP	LH	13475700	-	3	993	P	1335.997	0.3	0.083
OsJN02106	TIGR	9638.m02239	EP	HH	13097731	-	17	5935	P	1148.213	0.094	0.077
OsJN02107	TIGR	9638.m03924	EP	HH	22572459	-	8	3671	P	2239.888	0.071	0.031
OsJN02108	TIGR	9638.m03316	HP	LH	19374665	-	2	544	P	2925.198	0.333	1
OsJN02109	TIGR	9638.m02392	HP	LH	14217214	-	2	323	A	0	0	0
OsJN02110	TIGR	9638.m03627	EP	HH	20942942	-	22	7701	P	2319.077	0.088	0.046
OsJN02111	TIGR	9638.m02799	PP	LH	16432079	-	5	3020	P	2800.321	0.077	0
OsJN02112	TIGR	9638.m02641	HP	LH	15493239	-	1	957	P	2472.21	0.333	0
OsJN02113	TIGR	9638.m03327	PP	HH	19426081	-	4	3441	P	1542.429	0.4	0.429
OsJN02114	TIGR	9638.m01938	HP	LH	11379822	-	4	2328	P	1579.185	0.235	0.034
OsJN02115	TIGR	9638.m02475	PP	HH	14642978	-	2	2331	P	1554.131	0.16	0
OsJN02116	TIGR	9638.m02880	HP	HH	16797634	-	3	1298	P	1229.892	0.095	0
OsJN02117	TIGR	9638.m02311	HP	LH	13734618	-	1	270	P	2653.498	0.714	0
OsJN02118	TIGR	9638.m01982	HP	LH	11645980	-	2	950	A	1	0	0
OsJN02119	TIGR	9638.m02565	PP	HH	15045786	-	2	1170	A	440.98	0.042	0
OsJN02120	TIGR	9638.m02169	PP	LH	12768023	-	3	2159	P	1345.582	0.289	0
OsJN02121	TIGR	9638.m02000	HP	LH	11781230	-	4	1094	A	1	0	0
OsJN02122	TIGR	9638.m03388	PP	HH	19722365	-	9	6282	P	1314.775	0.171	0.074
OsJN02123	TIGR	9638.m03859	PP	HH	22226786	-	7	4814	P	1677.161	0.474	0.049

OsJN02124	TIGR	9638.m03826	HP	LH	22039057	-	2	571	P	1767.102	0.571	1
OsJN02125	TIGR	9638.m03608	HP	HH	20819851	-	10	4583	P	1448.29	0.167	0
OsJN02126	TIGR	9638.m02632	HP	HH	15437715	-	3	4095	P	972.526	0.167	0.082
OsJN02127	TIGR	9638.m02573	HP	LH	15085797	-	2	568	P	1142.186	0.667	0
OsJN02128	TIGR	9638.m03113	PP	HH	18322733	-	1	1652	P	1043.325	0.176	0
OsJN02129	TIGR	9638.m03000	PP	HH	17724356	-	3	2004	P	1767.916	0.324	0.143
OsJN02130	TIGR	9638.m02846	PP	HH	16619916	-	9	4091	P	1116.618	0.206	0
OsJN02131	TIGR	9638.m02837	PP	HH	16590837	-	2	2898	A	0	0	0
OsJN02132	TIGR	9638.m02274	PP	HH	13393710	-	9	4300	P	1269.38	0.103	0.093
OsJN02133	TIGR	9638.m03260	HP	LH	19065749	-	2	689	P	2362.409	0.556	0.6
OsJN02134	TIGR	9638.m03671	PP	HH	21171711	-	1	706	A	675.895	0.067	0
OsJN02135	TIGR	9638.m02497	HP	HH	14733126	-	2	414	P	3573.058	0.333	0
OsJN02136	TIGR	9638.m03069	EP	LH	18079053	-	1	736	P	749.178	0.267	0
OsJN02137	TIGR	9638.m01941	HP	LH	11396182	-	5	1525	P	1189.971	0.125	0.125
OsJN02138	TIGR	9638.m03142	PP	HH	18488369	-	4	2101	P	1997.376	0.056	0
OsJN02139	TIGR	9638.m02743	HP	LH	16115896	-	1	288	P	910.958	0.286	0
OsJN02140	TIGR	9638.m02937	PP	HH	17195606	-	3	2457	A	369.193	0.125	0.143
OsJN02141	TIGR	9638.m02105	EP	HH	12363893	-	7	1968	P	1383.19	0.037	0.118
OsJN02142	TIGR	9638.m02894	HP	LH	16868468	-	4	642	A	656.371	0.429	0
OsJN02143	TIGR	9638.m02555	PP	HH	15014088	-	1	1068	A	425.029	0.042	0
OsJN02144	TIGR	9638.m02658	EP	HH	15596121	-	1	1702	P	1798.455	0.541	0
OsJN02145	TIGR	9638.m02371	HP	LH	14092178	-	1	531	P	3002.841	0.333	0
OsJN02146	TIGR	9638.m02218	HP	HH	12999085	-	1	1956	P	1842.923	0.395	0
OsJN02147	TIGR	9638.m02089	HP	LH	12267925	-	2	574	A	0	0	0
OsJN02148	TIGR	9638.m02525	EP	HH	14841156	-	13	8868	P	1248.59	0.145	0.019
OsJN02149	TIGR	9638.m02197	EP	HH	12899943	-	1	785	P	1017.631	0.188	0
OsJN02150	TIGR	9638.m02996	HP	LH	17702307	-	2	3064	P	1890.212	0.233	0
OsJN02151	TIGR	9638.m03422	HP	LH	19904475	-	1	591	P	2054.364	0.462	0
OsJN02152	TIGR	9638.m02226	EP	LH	13050128	-	1	468	P	1650.853	0.636	0
OsJN02153	TIGR	9638.m02728	HP	LH	16021771	-	1	420	A	0	0	0
OsJN02154	TIGR	9638.m02346	HP	LH	13931681	-	1	759	P	1634.888	0.059	0
OsJN02155	TIGR	9638.m03720	PP	HH	21485658	-	5	2198	P	624.399	0.243	0.182
OsJN02156	TIGR	9638.m03719	PP	HH	21485658	-	4	2198	P	624.399	0.237	0.2
OsJN02157	TIGR	9638.m03120	PP	HH	18364652	-	9	2058	A	484.384	0.077	0.05
OsJN02158	TIGR	9638.m03674	PP	HH	21187371	-	1	781	P	1697.926	0.529	0
OsJN02159	TIGR	9638.m03380	HP	HH	19682714	-	5	3776	P	2554.364	0.158	0.095
OsJN02160	TIGR	9638.m03045	HP	LH	17928541	-	1	627	P	1943.005	0.071	0
OsJN02161	TIGR	9638.m03748	EP	HH	21618859	-	2	2036	P	1634.483	0.167	0
OsJN02162	TIGR	9638.m03505	PP	HH	20228284	-	7	2835	P	1676.207	0.268	0.05
OsJN02163	TIGR	9638.m02538	PP	HH	14924100	-	7	2602	P	1908.121	0.188	0.24
OsJN02164	TIGR	9638.m02222	HP	HH	13021373	-	2	1787	P	1554.409	0.235	0
OsJN02165	TIGR	9638.m02761	EP	HH	16209456	-	8	3633	P	806.17	0.157	0
OsJN02166	TIGR	9638.m03952	HP	LH	22682215	-	4	1005	P	2602.667	0.333	0.273
OsJN02167	TIGR	9638.m03565	PP	LH	20552503	-	1	768	P	1706.183	0.438	0
OsJN02168	TIGR	9638.m02587	HP	LH	15183263	-	1	714	P	1488.035	0.294	0
OsJN02169	TIGR	9638.m02424	HP	LH	14369184	-	7	2242	P	820.776	0.235	0
OsJN02170	TIGR	9638.m02139	EP	HH	12588735	-	9	5249	P	2237.018	0.081	0.026
OsJN02171	TIGR	9638.m03628	PP	HH	20951096	-	8	5710	P	923.453	0.069	0.062
OsJN02172	TIGR	9638.m02357	HP	LH	13983303	-	1	555	P	2456.392	0.6	0
OsJN02173	TIGR	9638.m03865	HP	LH	22254368	-	2	782	P	1177.897	0.143	0
OsJN02174	TIGR	9638.m02046	HP	LH	12011231	-	2	473	A	0	0	0
OsJN02175	TIGR	9638.m03706	PP	HH	21414450	-	3	4262	P	3002.935	0.058	0
OsJN02176	TIGR	9638.m03640	HP	LH	21002421	-	2	291	A	0	0	0
OsJN02177	TIGR	9638.m03293	PP	HH	19275640	-	3	1574	P	1447.423	0.222	0.143
OsJN02178	TIGR	9638.m03456	HP	LH	20029441	-	2	1718	P	2215.389	0.381	0.167
OsJN02179	TIGR	9638.m02383	EP	HH	14157480	-	2	1018	P	1293.428	0.333	0

OsJN02180	TIGR	9638.m03620	EP	LH	20903968	-	1	1081	P	734.335	0.136	0
OsJN02181	TIGR	9638.m03223	PP	HH	18871259	-	13	4939	P	1078.116	0.101	0.03
OsJN02182	TIGR	9638.m03202	HP	LH	18758308	-	2	1637	P	1404.535	0.545	0.1
OsJN02183	TIGR	9638.m02273	PP	HH	13379712	-	2	2726	P	1511.554	0.325	0.056
OsJN02184	TIGR	9638.m02907	PP	HH	16974774	-	15	8413	P	1494.912	0.141	0.06
OsJN02185	TIGR	9638.m03005	EP	LH	17743669	-	3	2741	P	1252.181	0.135	0
OsJN02186	TIGR	9638.m03457	PP	HH	20034774	-	2	2775	P	910.091	0.033	0
OsJN02187	TIGR	9638.m02660	EP	LH	15609264	-	2	2114	P	1012.441	0.263	0.25
OsJN02188	TIGR	9638.m03917	PP	HH	22532404	-	7	4325	P	1260.58	0.438	0
OsJN02189	TIGR	9638.m02499	HP	LH	14742678	-	2	1068	P	2178.281	0.333	0.167
OsJN02190	TIGR	9638.m02844	PP	HH	16616561	-	5	2532	P	1367.887	0.161	0.083
OsJN02191	TIGR	9638.m03876	PP	HH	22305402	-	1	411	A	459.287	0.333	0
OsJN02192	TIGR	9638.m03910	HP	LH	22516098	-	4	775	P	976.134	0.308	0
OsJN02193	TIGR	9638.m03697	EP	HH	21344882	-	4	1902	P	827.579	0.419	0
OsJN02194	TIGR	9638.m02640	HP	LH	15489052	-	2	1195	P	1686.274	0.429	0.421
OsJN02195	TIGR	9638.m02835	HP	LH	16577945	-	2	969	P	1461.344	0.444	0.083
OsJN02196	TIGR	9638.m02264	HP	HH	13328154	-	2	700	P	1179.359	0.125	0
OsJN02197	TIGR	9638.m03179	HP	LH	18653636	-	3	2077	A	376.153	0.077	0.094
OsJN02198	TIGR	9638.m03904	EP	HH	22483909	-	6	3037	P	1175.776	0.2	0.032
OsJN02199	TIGR	9638.m02805	PP	HH	16480726	-	6	2206	P	980.803	0.52	0
OsJN02200	TIGR	9638.m03004	PP	HH	17739712	-	7	2787	P	2382.637	0.409	0.158
OsJN02201	TIGR	9638.m02123	HP	HH	12493969	-	6	2867	P	1308.048	0.13	0.105
OsJN02202	TIGR	9638.m02816	HP	LH	16495741	-	2	2677	P	966.574	0.167	0.175
OsJN02203	TIGR	9638.m03070	HP	HH	18087544	-	3	2495	A	1	0	0
OsJN02204	TIGR	9638.m02979	HP	LH	17561707	-	2	1106	P	946.05	0.5	0.25
OsJN02205	TIGR	9638.m02562	HP	HH	15038514	-	2	1208	A	393.889	0.08	0
OsJN02206	TIGR	9638.m03923	EP	HH	22570122	-	1	2215	P	1773.268	0.25	0
OsJN02207	TIGR	9638.m03947	PP	HH	22662234	-	2	2730	A	0	0	0.143
OsJN02208	TIGR	9638.m02819	PP	LH	16502861	-	4	2159	P	1017.711	0.235	0.333
OsJN02209	TIGR	9638.m03355	HP	HH	19579124	-	1	1599	P	1898.248	0.147	0
OsJN02210	TIGR	9638.m02779	PP	HH	16335190	-	3	1019	A	515.468	0.105	0
OsJN02211	TIGR	9638.m01990	HP	LH	11721656	-	1	309	A	734.243	0.143	0
OsJN02212	TIGR	9638.m03440	EP	HH	19978553	-	1	246	P	31048.058	1	0
OsJN02213	TIGR	9638.m03404	HP	HH	19812544	-	2	3158	A	0	0	0.035
OsJN02214	TIGR	9638.m03084	PP	HH	18157596	-	2	2007	P	1340.122	0.381	0.05
OsJN02215	TIGR	9638.m02391	HP	LH	14210208	-	2	2164	P	1172.259	0.364	0.091
OsJN02216	TIGR	9638.m03724	PP	HH	21489101	-	3	1651	P	1424.821	0.129	0
OsJN02217	TIGR	9638.m02865	PP	HH	16702859	-	18	7034	P	1141.836	0.283	0.034
OsJN02218	TIGR	9638.m03500	EP	LH	20199153	-	3	1437	P	1350.318	0.292	0
OsJN02219	TIGR	9638.m03438	EP	HH	19975116	-	1	1512	P	1198.759	0.656	0
OsJN02220	TIGR	9638.m03372	PP	HH	19651629	-	11	4636	P	1944.623	0.135	0.041
OsJN02221	TIGR	9638.m03186	HP	HH	18684163	-	5	2918	A	0	0	0.031
OsJN02222	TIGR	9638.m02416	EP	LH	14321899	-	1	724	P	1753.455	0.235	0
OsJN02223	TIGR	9638.m03757	EP	HH	21663947	-	1	1963	P	1115.96	0.268	0
OsJN02224	TIGR	9638.m02605	PP	HH	15266691	-	5	5226	A	502.604	0.083	0.292
OsJN02225	TIGR	9638.m02535	HP	LH	14909779	-	1	813	P	1631.127	0.278	0
OsJN02226	TIGR	9638.m03085	PP	LH	18160607	-	3	1722	P	2278.605	0.583	0.091
OsJN02227	TIGR	9638.m03241	HP	LH	18968406	-	7	3956	P	1721.582	0.391	0.14
OsJN02228	TIGR	9638.m03018	PP	HH	17796894	-	2	2283	P	1731.287	0.277	0
OsJN02229	TIGR	9638.m02648	HP	LH	15529918	-	3	505	P	2636.882	0.545	1
OsJN02230	TIGR	9638.m03662	PP	HH	21132571	-	3	2073	P	1456.162	0.15	0.333
OsJN02231	TIGR	9638.m02589	EP	HH	15189497	-	11	2715	P	1681.421	0.194	0
OsJN02232	TIGR	9638.m03583	PP	HH	20666398	-	7	5236	P	1279.51	0.295	0.077
OsJN02233	TIGR	9638.m03419	HP	LH	19884949	-	3	981	A	0	0	0.125
OsJN02234	TIGR	9638.m02249	HP	LH	13203392	-	2	658	P	1235.364	0.636	0.25
OsJN02235	TIGR	9638.m02966	HP	HH	17438323	-	2	1483	P	2256.749	0.281	1

OsJN02236	TIGR	9638.m02939	HP	LH	17208382	-	3	1159	P	1975.855	0.286	0.077
OsJN02237	TIGR	9638.m02399	PP	HH	14245375	-	4	3721	P	1557.559	0.209	0.061
OsJN02238	TIGR	9638.m02548	HP	LH	14981628	-	2	420	A	0	0	1
OsJN02239	TIGR	9638.m02737	EP	LH	16080776	-	1	847	P	2128.842	0.474	0
OsJN02240	TIGR	9638.m02579	EP	HH	15128730	-	14	6399	P	792.875	0.068	0.039
OsJN02241	TIGR	9638.m03770	PP	HH	21733977	-	6	5158	P	1327.708	0.152	0.092
OsJN02242	TIGR	9638.m02949	PP	LH	17250912	-	2	1678	P	1104.069	0.194	0
OsJN02243	TIGR	9638.m02787	PP	HH	16376520	-	4	5181	P	1180.27	0.05	0
OsJN02244	TIGR	9638.m02913	HP	LH	17019269	-	2	466	P	1077.537	0.444	0
OsJN02245	TIGR	9629.m03533	HP	LH	15938929	-	2	845	A	1	0	0
OsJN02246	TIGR	9638.m03097	HP	HH	18220249	-	2	1778	P	2113.993	0.214	0.19
OsJN02247	TIGR	9638.m01964	PP	HH	11544030	-	2	4849	P	1679.952	0.393	0.101
OsJN02248	TIGR	9638.m02188	EP	LH	12856866	-	3	3436	P	1012.558	0.229	0.231
OsJN02249	TIGR	9638.m03423	EP	HH	19909094	-	6	4153	P	1152.236	0.176	0.077
OsJN02250	TIGR	9638.m02777	PP	HH	16325861	-	2	4821	P	1327.155	0.048	0
OsJN02251	TIGR	9638.m02882	EP	LH	16816128	-	1	935	P	1717.951	0.143	0
OsJN02252	TIGR	9638.m02563	HP	LH	15040010	-	3	1962	P	2161.624	0.273	0.125
OsJN02253	TIGR	9638.m03798	HP	LH	21913014	-	6	2093	P	2714.236	0.519	0.455
OsJN02254	TIGR	9638.m03058	HP	LH	17996851	-	2	1120	A	0	0	0.167
OsJN02255	TIGR	9638.m02091	HP	LH	12281532	-	1	537	P	2720.52	0.462	0
OsJN02256	TIGR	9638.m02823	HP	LH	16513743	-	2	1208	A	0	0	0.333
OsJN02257	TIGR	9638.m03832	EP	HH	22059302	-	3	826	A	721.127	0.118	0
OsJN02258	TIGR	9638.m02116	PP	HH	12436572	-	11	6116	P	1949.824	0.107	0.052
OsJN02259	TIGR	9638.m03273	PP	HH	19137880	-	11	5156	P	715.879	0.132	0.034
OsJN02260	TIGR	9638.m03285	HP	HH	19215271	-	6	1930	P	1905.024	0.292	0.056
OsJN02261	TIGR	9638.m02073	HP	LH	12176482	-	1	594	A	1	0	0
OsJN02262	TIGR	9638.m03492	PP	HH	20149531	-	1	2409	P	1644.314	0.189	0
OsJN02263	TIGR	9638.m02403	HP	LH	14267118	-	4	1454	P	3766.841	0.059	0.083
OsJN02264	TIGR	9638.m03633	PP	HH	20964670	-	9	5161	P	1343.951	0.068	0.075
OsJN02265	TIGR	9638.m03209	PP	HH	18806482	-	1	592	P	3290.132	0.231	0
OsJN02266	TIGR	9638.m02840	EP	HH	16600171	-	3	5665	P	1680.091	0.162	0.1
OsJN02267	TIGR	9638.m01996	HP	LH	11759345	-	2	1128	A	0	0	0.056
OsJN02268	TIGR	9638.m03409	PP	HH	19842179	-	1	1889	P	908.507	0.366	0
OsJN02269	TIGR	9638.m03044	HP	LH	17925186	-	1	636	P	865.436	0.308	0
OsJN02270	TIGR	9638.m03930	PP	HH	22597423	-	5	2895	P	1236.229	0.476	0.098
OsJN02271	TIGR	9638.m03288	EP	HH	19244315	-	2	2396	P	1615.585	0.409	0.033
OsJN02272	TIGR	9638.m02019	HP	LH	11860562	-	1	390	P	2466.457	0.75	0
OsJN02273	TIGR	9638.m02930	PP	LH	17145416	-	15	4480	P	1036.137	0.069	0.053
OsJN02274	TIGR	9638.m03881	EP	HH	22333744	-	7	4058	P	672.343	0.227	0.089
OsJN02275	TIGR	9638.m03696	EP	HH	21332994	-	3	1719	P	1276.236	0.2	0
OsJN02276	TIGR	9638.m03855	EP	HH	22179349	-	6	2466	P	953.562	0.109	0
OsJN02277	TIGR	9638.m03866	HP	LH	22256434	-	3	1736	P	2192.667	0.313	0.136
OsJN02278	TIGR	9638.m02746	EP	LH	16133772	-	1	818	P	2278.41	0.667	0
OsJN02279	TIGR	9638.m02237	HP	LH	13091806	-	5	2707	P	2243.525	0.458	0.097
OsJN02280	TIGR	9638.m03740	EP	LH	21566714	-	2	2796	P	2136.285	0.267	0.034
OsJN02281	TIGR	9638.m02170	HP	LH	12771612	-	3	2631	P	1841.492	0.462	0.143
OsJN02282	TIGR	9638.m03746	EP	LH	21609357	-	2	1424	P	1885.294	0.333	0
OsJN02283	TIGR	9638.m03743	PP	LH	21596684	-	1	881	P	1676.337	0.263	0
OsJN02284	TIGR	9638.m03592	EP	LH	20705435	-	2	1140	P	2218.182	0.313	0.25
OsJN02285	TIGR	9638.m03559	EP	HH	20513675	-	1	1735	P	1437.071	0.289	0
OsJN02286	TIGR	9638.m02710	HP	LH	15953280	-	2	1072	P	2135.67	0.714	0.667
OsJN02287	TIGR	9638.m02126	PP	HH	12511998	-	2	1891	P	2165.433	0.125	0
OsJN02288	TIGR	9638.m03294	EP	LH	19281130	-	1	532	P	870.731	0.25	0
OsJN02289	TIGR	9638.m03010	HP	LH	17775832	-	1	789	P	1818.332	0.111	0
OsJN02290	TIGR	9638.m02936	HP	HH	17187869	-	2	626	P	3358.61	0.333	0
OsJN02291	TIGR	9638.m03814	PP	HH	21979257	-	9	4323	P	1365.866	0.087	0.167

OsJN02292	TIGR	9638.m03526	EP	LH	20334499	-	3	1477	P	1486.628	0.417	0
OsJN02293	TIGR	9638.m03080	PP	HH	18136847	-	2	1793	P	1548.088	0.222	0
OsJN02294	TIGR	9638.m02407	EP	HH	14275768	-	6	2506	P	944.324	0.16	0.067
OsJN02295	TIGR	9638.m03385	EP	HH	19708018	-	6	3335	P	2900.713	0.104	0
OsJN02296	TIGR	9638.m03012	HP	LH	17780671	-	2	754	P	1176.645	0.5	0
OsJN02297	TIGR	9638.m03932	EP	LH	22609055	-	3	1824	P	1085.06	0.278	0
OsJN02298	TIGR	9638.m03756	EP	HH	21658229	-	6	3890	P	2123.507	0.333	0.043
OsJN02299	TIGR	9638.m02766	PP	HH	16257389	-	4	2763	P	1775.262	0.265	0.208
OsJN02300	TIGR	9638.m03373	PP	HH	19651629	-	11	4636	P	1944.623	0.123	0.045
OsJN02301	TIGR	9638.m02769	EP	HH	16268248	-	8	2755	A	632.124	0.111	0.061
OsJN02302	TIGR	9638.m02758	HP	LH	16202041	-	1	342	P	2004.688	0.25	0
OsJN02303	TIGR	9638.m03067	HP	LH	18068237	-	2	1530	P	3463.243	0.2	0.125
OsJN02304	TIGR	9638.m02410	HP	LH	14289427	-	3	450	P	2588.682	0.25	0
OsJN02305	TIGR	9638.m03781	PP	HH	21785474	-	1	971	P	2474.401	0.318	0
OsJN02306	TIGR	9638.m01948	HP	LH	11457882	-	1	237	A	474.439	0.167	0
OsJN02307	TIGR	9638.m03860	PP	HH	22234236	-	8	2943	P	1247.085	0.25	0.067
OsJN02308	TIGR	9638.m03909	PP	HH	22505705	-	3	3161	P	2116.526	0.1	0.081
OsJN02309	TIGR	9638.m03844	HP	LH	22126862	-	4	3036	P	916.255	0.364	0
OsJN02310	TIGR	9638.m03667	PP	HH	21159517	-	1	766	A	460.47	0.125	0
OsJN02311	TIGR	9638.m03094	EP	LH	18202226	-	6	3067	P	1092.442	0.405	0
OsJN02312	TIGR	9638.m03017	PP	HH	17796894	-	2	2283	P	1731.287	0.277	0
OsJN02313	TIGR	9638.m02564	HP	LH	15044191	-	1	285	P	1511.997	0.667	0
OsJN02314	TIGR	9638.m02729	EP	LH	16030256	-	1	570	P	2007.248	0.462	0
OsJN02315	TIGR	9638.m03118	EP	HH	18351457	-	12	4843	P	1244.041	0.119	0.044
OsJN02316	TIGR	9638.m02323	HP	HH	13790128	-	2	750	P	1792.887	0.313	0
OsJN02317	TIGR	9638.m02809	EP	HH	16470932	-	5	5158	P	1136.445	0.25	0.023
OsJN02318	TIGR	9638.m02735	HP	LH	16066472	-	1	564	P	2479.018	0.385	0
OsJN02319	TIGR	9638.m02342	HP	LH	13906565	-	1	231	P	2524.08	1	0
OsJN02320	TIGR	9638.m02415	HP	LH	14320138	-	2	721	P	882.216	0.125	0
OsJN02321	TIGR	9638.m03791	EP	HH	21830394	-	10	5515	P	1253.418	0.154	0.06
OsJN02322	TIGR	9638.m03669	PP	HH	21164383	-	1	620	A	538.236	0.071	0
OsJN02323	TIGR	9638.m02926	PP	LH	17115499	-	1	984	P	1774.543	0.238	0
OsJN02324	TIGR	9638.m03515	EP	LH	20283658	-	2	1249	P	1705.39	0.125	0
OsJN02325	TIGR	9638.m02651	HP	LH	15540400	-	4	4125	P	1569.285	0.292	0.111
OsJN02326	TIGR	9638.m03364	HP	LH	19608136	-	1	1251	P	1427.437	0.107	0
OsJN02327	TIGR	9638.m03286	PP	HH	19225722	-	1	630	P	1697.048	0.286	0
OsJN02328	TIGR	9638.m03584	PP	HH	20666398	-	6	5236	P	1279.51	0.29	0.078
OsJN02329	TIGR	9638.m03229	HP	LH	18904940	-	1	1464	P	2583.85	0.219	0
OsJN02330	TIGR	9638.m03215	PP	HH	18832398	-	1	276	P	1072.464	0.571	0
OsJN02331	TIGR	9638.m02778	HP	HH	16333745	-	2	1213	A	0	0	0.071
OsJN02332	TIGR	9638.m03463	HP	LH	20062739	-	2	566	P	2937.008	0.4	0
OsJN02333	TIGR	9638.m02402	HP	HH	14262912	-	3	1340	P	1495.937	0.148	0
OsJN02334	TIGR	9638.m02969	HP	HH	17465159	-	2	1268	P	1877.943	0.185	0
OsJN02335	TIGR	9638.m03824	HP	LH	22028340	-	7	2534	P	1704.865	0.31	0
OsJN02336	TIGR	9638.m03424	EP	HH	19924037	-	6	3513	P	1241.529	0.028	0
OsJN02337	TIGR	9638.m02481	HP	LH	14675780	-	1	360	A	424.743	0.333	0
OsJN02338	TIGR	9638.m02888	HP	LH	16838011	-	5	2781	P	3090.702	0.125	0.091
OsJN02339	TIGR	9638.m02616	HP	LH	15330007	-	2	1108	P	2383.453	0.783	0
OsJN02340	TIGR	9638.m02093	HP	LH	12286085	-	2	540	A	456.723	0.273	0.5
OsJN02341	TIGR	9638.m02436	EP	HH	14455458	-	2	3877	A	500.546	0.071	0.214
OsJN02342	TIGR	9638.m03723	PP	HH	21488343	-	4	2409	P	1424.821	0.089	0
OsJN02343	TIGR	9638.m02785	HP	HH	16361121	-	1	1407	P	822.906	0.2	0
OsJN02344	TIGR	9638.m02405	HP	LH	14273015	-	4	2093	P	2142.744	0.222	0.139
OsJN02345	TIGR	9638.m02200	HP	HH	12920354	-	1	486	P	890.626	0.083	0
OsJN02346	TIGR	9638.m03477	HP	LH	20103220	-	3	745	P	3353.409	0.333	0
OsJN02347	TIGR	9638.m02858	EP	HH	16683585	-	2	1533	P	1806.29	0.611	0.125

OsJN02348	TIGR	9638.m02506	PP	HH	14765835	-	1	471	P	1178.759	0.3	0
OsJN02349	TIGR	9638.m02406	EP	HH	14275689	-	7	2585	P	857.857	0.161	0.038
OsJN02350	TIGR	9638.m02038	HP	LH	11958466	-	2	843	A	0	0	0.111
OsJN02351	TIGR	9638.m02074	EP	LH	12185348	-	1	1010	P	1714.295	0.136	0
OsJN02352	TIGR	9638.m02994	HP	LH	17695285	-	7	2837	P	912.167	0.039	0.083
OsJN02353	TIGR	9638.m02725	EP	HH	16008358	-	12	3645	P	1167.797	0.065	0
OsJN02354	TIGR	9638.m03190	PP	HH	18697801	-	5	1985	P	1509.031	0.281	0.083
OsJN02355	TIGR	9638.m02272	EP	HH	13367418	-	3	4483	P	1792.857	0.108	0.067
OsJN02356	TIGR	9638.m02017	HP	LH	11854696	-	6	3314	P	1756.611	0.139	0
OsJN02357	TIGR	9638.m02849	PP	HH	16632424	-	11	4692	P	849.347	0.208	0.056
OsJN02358	TIGR	9638.m03677	PP	HH	21197784	-	14	4079	P	1266.534	0.244	0
OsJN02359	TIGR	9638.m02556	HP	LH	15016902	-	3	1047	P	1042.465	0.227	0
OsJN02360	TIGR	9638.m02452	HP	LH	14533663	-	3	535	A	635.505	0.286	0
OsJN02361	TIGR	9638.m02362	PP	HH	14005714	-	10	3459	P	1132.181	0.091	0.032
OsJN02362	TIGR	9638.m03185	PP	LH	18674318	-	16	6583	P	1749.989	0.154	0.068
OsJN02363	TIGR	9638.m02883	PP	HH	16819534	-	2	1979	A	502.113	0.029	0.125
OsJN02364	TIGR	9638.m02366	EP	HH	14048510	-	13	6304	P	1471.34	0.25	0.048
OsJN02365	TIGR	9638.m03188	PP	HH	18689186	-	3	4319	P	1080.265	0.2	0.203
OsJN02366	TIGR	9638.m03072	EP	LH	18095353	-	2	807	P	1573.158	0.5	0
OsJN02367	TIGR	9638.m02187	EP	HH	12850337	-	5	4269	P	1219.92	0.3	0.05
OsJN02368	TIGR	9638.m02516	EP	HH	14803169	-	4	5402	P	1139.441	0.222	0.167
OsJN02369	TIGR	9638.m03177	EP	HH	18646826	-	3	3144	P	1132.223	0.273	0.077
OsJN02370	TIGR	9638.m03739	HP	LH	21563787	-	1	618	P	1749.511	0.643	0
OsJN02371	TIGR	9638.m02592	HP	LH	15199777	-	2	832	P	1591.386	0.417	0.5
OsJN02372	TIGR	9638.m02440	HP	LH	14484037	-	1	576	P	1160.054	0.308	0
OsJN02373	TIGR	9638.m03414	EP	HH	19862393	-	5	2942	P	1538.515	0.225	0.12
OsJN02374	TIGR	9638.m03793	HP	LH	21869648	-	4	2093	P	1485.27	0.346	0.263
OsJN02375	TIGR	9638.m02233	HP	LH	13079847	-	7	2839	P	2566.07	0.1	0.333
OsJN02376	TIGR	9638.m02864	PP	HH	16700791	-	2	1562	P	760.368	0.222	0.2
OsJN02377	TIGR	9638.m02718	HP	LH	15983682	-	2	1260	P	1686.299	0.6	0
OsJN02378	TIGR	9638.m03898	PP	HH	22447519	-	2	1966	A	0	0	0
OsJN02379	TIGR	9638.m03382	PP	HH	19693675	-	13	4724	P	1136.864	0.119	0.114
OsJN02380	TIGR	9638.m00007	HP	HH	39277	-	9	3152	A	0	0	0.056
OsJN02381	TIGR	9638.m00664	EP	LH	3947510	-	7	5195	P	425.95	0.095	0.014
OsJN02382	TIGR	9638.m00509	HP	LH	2987756	-	2	579	A	0	0	0
OsJN02383	TIGR	9638.m00850	EP	LH	4942988	-	1	414	A	0	0	0
OsJN02384	TIGR	9638.m00123	HP	LH	738438	-	3	547	A	0	0	0
OsJN02385	TIGR	9638.m01723	HP	HH	10026895	-	1	2184	P	651.704	0.25	0
OsJN02386	TIGR	9638.m01457	HP	LH	8451036	-	2	289	P	554.163	0.25	0
OsJN02387	TIGR	9638.m00071	HP	LH	428684	-	2	1026	P	921.511	0.143	0.25
OsJN02388	TIGR	9638.m00515	HP	LH	3037733	-	3	686	A	0	0	0.143
OsJN02389	TIGR	9638.m01065	HP	LH	6212668	-	4	2069	P	845.279	0.304	0.059
OsJN02390	TIGR	9638.m00348	HP	LH	2058301	-	2	2234	P	277.66	0.063	0.036
OsJN02391	TIGR	9638.m00406	EP	LH	2397027	-	1	444	A	0	0	0
OsJN02392	TIGR	9638.m00063	EP	LH	374929	-	2	1428	P	495.171	0.05	0
OsJN02393	TIGR	9638.m00456	EP	LH	2671494	-	2	293	A	0	0	0
OsJN02394	TIGR	9638.m01232	HP	LH	7211051	-	2	2272	P	1174.604	0.2	0.172
OsJN02395	TIGR	9638.m01190	PP	HH	6950390	-	4	6621	P	229.878	0.25	0
OsJN02396	TIGR	9638.m00955	HP	LH	5619509	-	2	2559	A	0	0	0.12
OsJN02397	TIGR	9638.m00606	HP	LH	3573921	-	1	258	A	0	0	0
OsJN02398	TIGR	9638.m00928	EP	LH	5473670	-	2	409	P	1126.625	0.4	0
OsJN02399	TIGR	9638.m00111	PP	HH	664245	-	3	3229	P	560.908	0.147	0.029
OsJN02400	TIGR	9638.m00779	HP	LH	4568098	-	2	769	P	337.784	0.143	0
OsJN02401	TIGR	9638.m00211	EP	HH	1249432	-	1	1185	A	0	0	0
OsJN02402	TIGR	9638.m00257	HP	LH	1498960	-	1	495	P	558.036	0.25	0
OsJN02403	TIGR	9638.m00767	HP	HH	4504702	-	1	453	P	366.059	0.1	0

OsJN02404	TIGR	9638.m01720	HP	LH	10007767	-	2	1036	A	0	0	0
OsJN02405	TIGR	9638.m01230	HP	LH	7200144	-	3	1527	P	217.674	0.167	0.143
OsJN02406	TIGR	9638.m00382	PP	HH	2233527	-	7	2852	P	428.226	0.02	0
OsJN02407	TIGR	9638.m01675	HP	HH	9712967	-	3	1206	A	0	0	0.125
OsJN02408	TIGR	9638.m01253	EP	HH	7305748	-	6	1974	A	0	0	0.036
OsJN02409	TIGR	9638.m01209	HP	LH	7085173	-	2	2258	A	0	0	0.042
OsJN02410	TIGR	9638.m00613	HP	LH	3613545	-	1	222	P	420.562	0.167	0
OsJN02411	TIGR	9638.m00109	HP	LH	660395	-	2	296	A	0	0	0
OsJN02412	TIGR	9638.m00729	HP	LH	4325522	-	3	1365	P	770.087	0.3	0.125
OsJN02413	TIGR	9638.m00966	HP	LH	5692050	-	2	1883	A	0	0	0
OsJN02414	TIGR	9638.m00314	PP	HH	1855156	-	3	4527	P	1058.916	0.024	0
OsJN02415	TIGR	9638.m01100	PP	LH	6439518	-	7	2670	P	243.433	0.045	0
OsJN02416	TIGR	9638.m01906	PP	HH	11187083	-	5	3898	P	493.593	0.143	0
OsJN02417	TIGR	9638.m00226	HP	LH	1331908	-	3	1290	A	0	0	0.143
OsJN02418	TIGR	9638.m01114	EP	LH	6503034	-	1	732	P	379.647	0.188	0
OsJN02419	TIGR	9638.m00711	HP	LH	4237764	-	2	462	P	518.68	0.571	0.25
OsJN02420	TIGR	9638.m01807	PP	HH	10549079	-	3	4613	P	760.904	0.07	0.018
OsJN02421	TIGR	9638.m01523	HP	LH	8840389	-	4	1790	A	0	0	0.04
OsJN02422	TIGR	9638.m01335	HP	HH	7726273	-	2	956	P	633.287	0.19	0
OsJN02423	TIGR	9638.m01243	EP	LH	7273357	-	3	1160	P	887.211	0.053	0
OsJN02424	TIGR	9638.m00510	EP	LH	2996988	-	2	1109	P	660.035	0.083	0
OsJN02425	TIGR	9638.m00646	PP	HH	3833353	-	2	2441	P	773.714	0.211	0
OsJN02426	TIGR	9638.m01766	EP	HH	10303203	-	1	1110	P	564.523	0.652	0
OsJN02427	TIGR	9638.m01670	EP	HH	9676369	-	2	7218	P	986.674	0.1	0.043
OsJN02428	TIGR	9638.m00673	HP	HH	4024183	-	1	420	P	365.821	0.111	0
OsJN02429	TIGR	9638.m01732	HP	LH	10069623	-	1	255	A	0	0	0
OsJN02430	TIGR	9638.m00910	PP	HH	5351078	-	2	3542	P	375.86	0.029	0
OsJN02431	TIGR	9638.m01382	HP	LH	7956832	-	3	1028	P	432.313	0.154	0
OsJN02432	TIGR	9638.m00362	HP	LH	2132829	-	6	1283	P	847.36	0.167	0.1
OsJN02433	TIGR	9638.m01227	HP	LH	7183475	-	2	1785	P	448.846	0.105	0.167
OsJN02434	TIGR	9638.m00519	EP	LH	3062432	-	1	192	P	969.386	0.2	0
OsJN02435	TIGR	9638.m01222	HP	LH	7151650	-	4	4714	P	667.991	0.143	0.11
OsJN02436	TIGR	9638.m00444	HP	LH	2613606	-	1	384	A	0	0	0
OsJN02437	TIGR	9638.m00501	PP	HH	2951067	-	2	802	P	294.577	0.188	0
OsJN02438	TIGR	9638.m00255	EP	HH	1482399	-	1	918	P	947.228	0.05	0
OsJN02439	TIGR	9638.m00380	EP	LH	2225168	-	2	4868	P	657.728	0.118	0
OsJN02440	TIGR	9638.m01776	EP	HH	10370691	-	1	348	P	980.709	0.875	0
OsJN02441	TIGR	9638.m01771	EP	HH	10342093	-	3	2253	P	467.67	0.087	0.05
OsJN02442	TIGR	9638.m00039	HP	LH	207703	-	1	447	P	453.454	0.182	0
OsJN02443	TIGR	9638.m00802	HP	LH	4681446	-	5	2605	P	445.382	0.1	0.106
OsJN02444	TIGR	9638.m01016	PP	HH	5955087	-	4	2661	P	1030.128	0.024	0.111
OsJN02445	TIGR	9638.m00359	HP	LH	2117316	-	2	336	P	758.332	0.167	0.333
OsJN02446	TIGR	9638.m00405	EP	LH	2395858	-	3	716	A	0	0	0
OsJN02447	TIGR	9638.m01008	PP	HH	5915385	-	7	4305	P	524.583	0.111	0.111
OsJN02448	TIGR	9638.m00593	EP	HH	3509938	-	2	970	P	348.081	0.176	0
OsJN02449	TIGR	9638.m00655	HP	LH	3876059	-	4	4110	P	647.899	0.105	0.114
OsJN02450	TIGR	9638.m00292	PP	HH	1727723	-	4	3411	A	0	0	0
OsJN02451	TIGR	9638.m01564	HP	LH	9047854	-	3	2272	P	748.502	0.176	0.222
OsJN02452	TIGR	9638.m01139	EP	HH	6641811	-	1	1914	A	0	0	0
OsJN02453	TIGR	9638.m00160	HP	LH	950041	-	8	3204	P	300.399	0.021	0
OsJN02454	TIGR	9638.m00733	HP	LH	4339861	-	4	2161	A	204.39	0.067	0.125
OsJN02455	TIGR	9638.m01451	HP	LH	8413402	-	5	1774	P	659.617	0.071	0
OsJN02456	TIGR	9638.m01711	EP	HH	9923647	-	4	4162	P	624.755	0.132	0
OsJN02457	TIGR	9638.m00927	EP	HH	5456944	-	4	2791	A	0	0	0
OsJN02458	TIGR	9638.m00457	HP	LH	2675507	-	2	1033	P	650.533	0.25	0.5
OsJN02459	TIGR	9638.m01546	HP	HH	8933692	-	1	702	P	657.813	0.067	0

OsJN02460	TIGR	9638.m00576	HP	LH	3381946	-	1	438	P	983.154	0.2	0
OsJN02461	TIGR	9638.m01868	HP	LH	10959961	-	5	1600	A	0	0	0
OsJN02462	TIGR	9638.m00743	HP	LH	4392502	-	5	1426	P	240.943	0.063	0
OsJN02463	TIGR	9638.m01911	HP	LH	11238563	-	2	634	P	464.689	0.231	1
OsJN02464	TIGR	9638.m01030	HP	LH	6022332	-	3	3473	P	469.675	0.059	0.156
OsJN02465	TIGR	9638.m01064	HP	HH	6209172	-	1	2589	P	529.108	0.158	0
OsJN02466	TIGR	9638.m00603	HP	LH	3568737	-	1	186	P	457.199	0.5	0
OsJN02467	TIGR	9638.m01397	HP	LH	8069544	-	4	1803	P	908.857	0.067	0
OsJN02468	TIGR	9638.m01274	HP	LH	7394876	-	2	1231	A	0	0	0
OsJN02469	TIGR	9638.m01201	HP	LH	7032029	-	3	1075	P	386.109	0.214	0
OsJN02470	TIGR	9638.m01176	HP	LH	6857773	-	2	584	P	891.259	0.6	0
OsJN02471	TIGR	9638.m00502	HP	LH	2954959	-	2	1264	P	592.372	0.5	0.125
OsJN02472	TIGR	9638.m01519	PP	HH	8812256	-	10	7669	P	616.629	0.066	0.049
OsJN02473	TIGR	9638.m00994	HP	LH	5844172	-	3	1203	P	504.098	0.357	0
OsJN02474	TIGR	9638.m00719	HP	LH	4270640	-	4	1040	A	0	0	0.143
OsJN02475	TIGR	9638.m01018	PP	LH	5962678	-	2	1353	P	568.188	0.133	0
OsJN02476	TIGR	9638.m01635	EP	LH	9488180	-	2	516	A	0	0	0
OsJN02477	TIGR	9638.m00446	HP	LH	2619762	-	1	219	P	737.29	0.4	0
OsJN02478	TIGR	9638.m01524	HP	LH	8846261	-	1	750	P	342.737	0.059	0
OsJN02479	TIGR	9638.m01795	HP	LH	10503202	-	5	2595	P	473.954	0.154	0
OsJN02480	TIGR	9638.m00324	HP	LH	1916844	-	2	1186	P	516.432	0.042	0
OsJN02481	TIGR	9638.m01259	HP	LH	7335841	-	2	599	A	0	0	0
OsJN02482	TIGR	9638.m00189	EP	LH	1120859	-	1	264	A	0	0	0
OsJN02483	TIGR	9638.m00491	PP	HH	2896901	-	2	1960	P	620.927	0.195	0
OsJN02484	TIGR	9638.m01631	EP	HH	9416838	-	10	10530	P	621.494	0.111	0.027
OsJN02485	TIGR	9638.m00057	EP	HH	332968	-	13	9192	P	553.625	0.104	0.043
OsJN02486	TIGR	9638.m00050	PP	HH	269442	-	3	1661	P	1306.191	0.158	0.067
OsJN02487	TIGR	9638.m00799	HP	LH	4662306	-	1	537	P	865.57	0.091	0
OsJN02488	TIGR	9638.m00177	EP	HH	1051224	-	3	3162	P	452.79	0.02	0
OsJN02489	TIGR	9638.m01219	PP	HH	7129804	-	4	4137	P	531.105	0.098	0.022
OsJN02490	TIGR	9638.m01836	HP	LH	10741873	-	1	438	P	696.995	0.455	0
OsJN02491	TIGR	9638.m00070	EP	HH	419567	-	2	2579	P	701.253	0.083	0
OsJN02492	TIGR	9638.m01907	PP	LH	11194934	-	19	14566	P	539.629	0.103	0.047
OsJN02493	TIGR	9638.m01054	HP	LH	6137006	-	1	630	A	0	0	0
OsJN02494	TIGR	9638.m00499	PP	HH	2943327	-	2	777	P	692.852	0.188	0
OsJN02495	TIGR	9638.m00911	PP	HH	5350481	-	3	4189	P	375.86	0.025	0
OsJN02496	TIGR	9638.m01233	PP	HH	7215071	-	3	4466	P	611.562	0.078	0
OsJN02497	TIGR	9638.m01872	HP	HH	10990696	-	5	3329	P	655.458	0.037	0
OsJN02498	TIGR	9638.m01341	HP	LH	7759638	-	5	1476	P	1168.467	0.125	0
OsJN02499	TIGR	9638.m01007	PP	HH	5908862	-	2	2356	P	361.97	0.071	0
OsJN02500	TIGR	9638.m01445	HP	LH	8371377	-	1	276	A	0	0	0
OsJN02501	TIGR	9638.m00712	HP	LH	4240781	-	2	453	P	797.981	0.455	0
OsJN02502	TIGR	9638.m01021	PP	HH	5975636	-	1	4221	A	0	0	0
OsJN02503	TIGR	9638.m00685	HP	LH	4061651	-	1	291	P	665.216	0.286	0
OsJN02504	TIGR	9638.m00787	HP	LH	4602482	-	2	664	P	631.658	0.273	0.333
OsJN02505	TIGR	9638.m00475	HP	LH	2797726	-	1	255	P	405.413	0.333	0
OsJN02506	TIGR	9638.m00283	PP	HH	1684479	-	4	2626	P	433.211	0.053	0.05
OsJN02507	TIGR	9638.m00388	PP	LH	2280853	-	2	4010	P	1080.321	0.081	0
OsJN02508	TIGR	9638.m01873	HP	HH	11001783	-	2	1388	P	448.552	0.118	0
OsJN02509	TIGR	9638.m00980	HP	LH	5752331	-	2	1688	P	1067.648	0.105	0
OsJN02510	TIGR	9638.m00619	HP	LH	3667977	-	2	1973	P	822.613	0.158	0.19
OsJN02511	TIGR	9638.m00181	EP	HH	1069315	-	8	4343	P	628.685	0.145	0.025
OsJN02512	TIGR	9638.m01845	PP	HH	10819652	-	11	11353	P	626.344	0.045	0.013
OsJN02513	TIGR	9638.m01897	HP	HH	11144969	-	4	1793	P	246.525	0.063	0.042
OsJN02514	TIGR	9638.m00785	HP	HH	4593986	-	3	1748	P	1077.09	0.125	0.043
OsJN02515	TIGR	9638.m01183	HP	HH	6900487	-	2	613	P	379.215	0.133	0

OsJN02516	TIGR	9638.m01541	HP	HH	8919637	-	1	867	P	395.601	0.105	0
OsJN02517	TIGR	9638.m01044	PP	HH	6071669	-	4	4578	P	903.728	0.031	0
OsJN02518	TIGR	9638.m00154	PP	HH	910834	-	1	3333	P	784.084	0.068	0
OsJN02519	TIGR	9638.m01207	PP	HH	7068853	-	13	5743	P	281.013	0.115	0.016
OsJN02520	TIGR	9638.m00458	EP	LH	2682949	-	5	3626	P	479.216	0.154	0.108
OsJN02521	TIGR	9638.m00227	HP	LH	1336787	-	6	1313	A	0	0	0.111
OsJN02522	TIGR	9638.m01107	PP	HH	6463584	-	5	5832	P	530.195	0.091	0
OsJN02523	TIGR	9638.m01752	HP	HH	10160677	-	2	1489	A	0	0	0
OsJN02524	TIGR	9638.m00256	PP	HH	1485426	-	3	2038	P	218.269	0.037	0
OsJN02525	TIGR	9638.m01821	HP	LH	10643007	-	3	2500	P	761.008	0.2	0.025
OsJN02526	TIGR	9638.m00639	EP	HH	3783617	-	6	4347	P	927.326	0.125	0.032
OsJN02527	TIGR	9638.m00145	HP	LH	864508	-	1	219	P	430.54	0.4	0
OsJN02528	TIGR	9638.m01463	PP	HH	8473923	-	3	2585	P	278.377	0.045	0
OsJN02529	TIGR	9638.m00469	EP	HH	2747753	-	5	867	P	268.746	0.167	0
OsJN02530	TIGR	9638.m01707	EP	HH	9886827	-	6	5741	P	696.534	0.036	0.059
OsJN02531	TIGR	9638.m01223	PP	HH	7159831	-	2	1140	P	544.908	0.087	0
OsJN02532	TIGR	9638.m01886	PP	LH	11061944	-	4	2749	P	766.756	0.25	0.296
OsJN02533	TIGR	9638.m01353	HP	LH	7818175	-	2	997	P	288.695	0.1	0.091
OsJN02534	TIGR	9638.m00906	HP	HH	5326976	-	2	882	P	489.493	0.167	0
OsJN02535	TIGR	9638.m01871	PP	HH	10984069	-	4	4145	P	1076.946	0.063	0
OsJN02536	TIGR	9638.m01571	HP	LH	9095844	-	5	1779	A	0	0	0
OsJN02537	TIGR	9638.m01193	HP	LH	6972390	-	2	465	P	964.472	0.143	0
OsJN02538	TIGR	9638.m01891	EP	HH	11092807	-	3	2863	P	482.198	0.1	0.075
OsJN02539	TIGR	9638.m01113	HP	LH	6493787	-	6	4484	P	527.075	0.16	0.082
OsJN02540	TIGR	9638.m00485	PP	HH	2869441	-	2	4484	P	853.112	0.115	0.029
OsJN02541	TIGR	9638.m00031	HP	HH	171555	-	1	1059	P	648.515	0.261	0
OsJN02542	TIGR	9638.m00644	PP	HH	3816195	-	13	9198	P	708.685	0.03	0.039
OsJN02543	TIGR	9638.m00493	PP	HH	2909381	-	10	8828	P	883.641	0.071	0.015
OsJN02544	TIGR	9638.m01255	HP	LH	7315646	-	2	808	P	906.854	0.412	0
OsJN02545	TIGR	9638.m00471	EP	LH	2764617	-	3	2656	P	445.9	0.24	0
OsJN02546	TIGR	9638.m01096	HP	LH	6389640	-	8	7295	P	245.683	0.029	0
OsJN02547	TIGR	9638.m00010	EP	HH	65260	-	1	1084	P	622.498	0.083	0
OsJN02548	TIGR	9638.m01332	HP	LH	7709217	-	1	906	P	365.027	0.158	0
OsJN02549	TIGR	9638.m00276	PP	HH	1640758	-	3	2639	A	0	0	0
OsJN02550	TIGR	9638.m00082	EP	HH	493772	-	2	1165	A	0	0	0
OsJN02551	TIGR	9638.m01059	HP	LH	6168331	-	4	1574	P	899.32	0.286	0.286
OsJN02552	TIGR	9638.m00264	PP	HH	1524941	-	4	5610	P	613.127	0.102	0.075
OsJN02553	TIGR	9638.m01540	HP	LH	8917966	-	1	555	A	202.341	0.083	0
OsJN02554	TIGR	9638.m01087	HP	LH	6344691	-	3	2182	P	251.67	0.059	0.115
OsJN02555	TIGR	9638.m00203	EP	HH	1207806	-	2	3370	A	205.103	0.014	0
OsJN02556	TIGR	9638.m01673	EP	LH	9699633	-	2	676	P	825.616	0.063	0
OsJN02557	TIGR	9638.m01734	PP	HH	10073757	-	2	1147	A	0	0	0.067
OsJN02558	TIGR	9638.m00377	EP	HH	2211497	-	1	2784	P	616.915	0.016	0
OsJN02559	TIGR	9638.m00130	EP	HH	790213	-	5	3652	P	275.176	0.034	0
OsJN02560	TIGR	9638.m00260	EP	HH	1509659	-	1	828	P	713.151	0.158	0
OsJN02561	TIGR	9638.m01851	PP	HH	10859662	-	4	6400	P	958.678	0.047	0
OsJN02562	TIGR	9638.m01225	PP	LH	7168379	-	3	1374	P	837.82	0.25	0
OsJN02563	TIGR	9638.m00166	HP	HH	984473	-	4	2329	P	980.261	0.192	0.208
OsJN02564	TIGR	9638.m00460	EP	HH	2693260	-	2	2802	P	295.078	0.028	0
OsJN02565	TIGR	9638.m00464	PP	LH	2721190	-	3	4823	P	364.439	0.105	0.03
OsJN02566	TIGR	9638.m01455	HP	LH	8436944	-	4	2693	P	866.804	0.057	0.059
OsJN02567	TIGR	9638.m01098	HP	LH	6412476	-	4	1764	A	0	0	0.083
OsJN02568	TIGR	9638.m00148	HP	LH	875108	-	2	1484	P	333.226	0.176	0
OsJN02569	TIGR	9638.m00611	EP	HH	3604709	-	3	2340	P	573.178	0.059	0.235
OsJN02570	TIGR	9638.m00481	PP	HH	2833780	-	5	6177	P	1031.175	0.18	0.014
OsJN02571	TIGR	9638.m01320	HP	HH	7643298	-	2	999	A	0	0	0

OsJN02572	TIGR	9638.m00757	HP	LH	4457271	-	1	210	A	0	0	0
OsJN02573	TIGR	9638.m01859	HP	LH	10905286	-	2	372	P	593.88	0.333	0
OsJN02574	TIGR	9638.m01281	PP	HH	7431429	-	8	6443	P	513.052	0.127	0.014
OsJN02575	TIGR	9638.m00508	EP	LH	2983491	-	2	1893	P	265.654	0.136	0
OsJN02576	TIGR	9638.m01220	HP	LH	7135265	-	4	2021	P	524.023	0.111	0.083
OsJN02577	TIGR	9638.m01184	HP	LH	6901873	-	2	463	A	0	0	0
OsJN02578	TIGR	9638.m01791	PP	LH	10479210	-	2	1567	A	0	0	0
OsJN02579	TIGR	9638.m00617	PP	HH	3643768	-	1	1850	P	546.241	0.15	0
OsJN02580	TIGR	9638.m00720	HP	LH	4276964	-	3	956	A	0	0	0
OsJN02581	TIGR	9638.m01783	HP	LH	10436503	-	1	285	P	495.547	0.5	0
OsJN02582	TIGR	9638.m00182	EP	HH	1069315	-	8	4343	P	628.685	0.145	0.025
OsJN02583	TIGR	9638.m01475	HP	LH	8542472	-	2	2911	A	0	0	0.227
OsJN02584	TIGR	9638.m00900	EP	LH	5301551	-	1	888	P	597.784	0.3	0
OsJN02585	TIGR	9638.m00631	HP	LH	3735724	-	2	767	P	707.384	0.4	0
OsJN02586	TIGR	9638.m00997	EP	HH	5855649	-	7	6598	P	249.953	0.038	0
OsJN02587	TIGR	9638.m01922	HP	LH	11292170	-	3	2038	P	872.31	0.212	0.364
OsJN02588	TIGR	9638.m00877	EP	HH	5118133	-	8	5839	A	0	0	0.043
OsJN02589	TIGR	9638.m00977	PP	HH	5736702	-	9	7875	P	387.923	0.105	0.009
OsJN02590	TIGR	9638.m00346	PP	LH	2042794	-	3	2127	P	286.989	0.074	0.05
OsJN02591	TIGR	9638.m00049	HP	LH	266297	-	1	414	P	857.046	0.3	0
OsJN02592	TIGR	9638.m01883	PP	HH	11039037	-	4	2937	P	477.276	0.19	0.071
OsJN02593	TIGR	9638.m01439	PP	HH	8329571	-	4	993	P	324.559	0.077	0
OsJN02594	TIGR	9638.m01166	HP	LH	6799327	-	1	603	A	1	0	0
OsJN02595	TIGR	9638.m00912	HP	LH	5357080	-	2	1325	A	0	0	0
OsJN02596	TIGR	9638.m01239	HP	LH	7246447	-	2	821	P	1055.564	0.333	1
OsJN02597	TIGR	9638.m01199	HP	LH	7013662	-	3	516	A	0	0	0
OsJN02598	TIGR	9638.m01511	HP	LH	8764482	-	2	1257	P	822.114	0.318	0.667
OsJN02599	TIGR	9638.m00140	PP	HH	832316	-	1	2041	A	0	0	0
OsJN02600	TIGR	9638.m01924	HP	LH	11303268	-	16	12495	P	816.886	0.054	0.064
OsJN02601	TIGR	9638.m00827	HP	LH	4808775	-	2	344	A	0	0	0
OsJN02602	TIGR	9638.m01642	HP	LH	9524445	-	3	1229	P	273.186	0.1	0.167
OsJN02603	TIGR	9638.m00125	PP	HH	755832	-	5	3764	P	826.918	0.085	0
OsJN02604	TIGR	9638.m01431	HP	LH	8293269	-	2	1071	A	0	0	0
OsJN02605	TIGR	9638.m01267	HP	LH	7368768	-	3	1950	A	0	0	0
OsJN02606	TIGR	9638.m00992	PP	HH	5823853	-	22	7478	P	462.328	0.091	0.052
OsJN02607	TIGR	9638.m00742	HP	LH	4389716	-	4	550	P	911.962	0.167	0
OsJN02608	TIGR	9638.m01863	HP	HH	10923676	-	6	5166	P	440.514	0.056	0.125
OsJN02609	TIGR	9638.m00857	HP	LH	4978221	-	2	1706	P	258.563	0.222	0.138
OsJN02610	TIGR	9638.m01820	HP	LH	10625522	-	5	3352	P	476.279	0.1	0
OsJN02611	TIGR	9638.m00392	HP	LH	2315909	-	1	354	P	403.255	0.25	0
OsJN02612	TIGR	9638.m01137	PP	HH	6623585	-	5	5495	P	465.41	0.111	0
OsJN02613	TIGR	9638.m00728	EP	HH	4320128	-	10	3596	P	783.292	0.195	0.027
OsJN02614	TIGR	9638.m00657	PP	HH	3892928	-	1	2415	P	437.313	0.057	0
OsJN02615	TIGR	9638.m01343	HP	LH	7766087	-	2	710	A	0	0	0
OsJN02616	TIGR	9638.m00692	HP	LH	4106651	-	3	2781	P	622.545	0.211	0.222
OsJN02617	TIGR	9638.m00745	HP	LH	4400546	-	2	787	P	981.483	0.4	0
OsJN02618	TIGR	9638.m00375	HP	LH	2204639	-	1	927	P	354.941	0.4	0
OsJN02619	TIGR	9638.m00016	HP	HH	100190	-	1	393	P	863.233	0.222	0
OsJN02620	TIGR	9638.m00527	PP	HH	3097980	-	8	5041	P	893.247	0.111	0.12
OsJN02621	TIGR	9638.m00486	PP	HH	2871500	-	1	2429	P	853.112	0.135	0
OsJN02622	TIGR	9638.m01469	HP	LH	8519459	-	5	995	A	0	0	0
OsJN02623	TIGR	9638.m00976	PP	HH	5736910	-	9	7666	P	387.923	0.113	0.009
OsJN02624	TIGR	9638.m00947	HP	LH	5567624	-	3	516	A	0	0	0.25
OsJN02625	TIGR	9638.m01305	HP	LH	7555576	-	1	498	P	530.003	0.4	0
OsJN02626	TIGR	9638.m00487	HP	LH	2878545	-	3	1634	P	774.974	0.143	0
OsJN02627	TIGR	9638.m00149	HP	LH	879912	-	1	402	P	480.025	0.25	0

OsJN02628	TIGR	9638.m01908	HP	LH	11212494	-	2	1315	P	434.164	0.4	0.118
OsJN02629	TIGR	9638.m01140	EP	HH	6647162	-	1	1131	A	0	0	0
OsJN02630	TIGR	9638.m01811	HP	LH	10570320	-	2	584	A	0	0	0.6
OsJN02631	TIGR	9638.m00659	HP	LH	3910456	-	1	447	P	791.919	0.1	0
OsJN02632	TIGR	9638.m00530	HP	LH	3119558	-	2	353	A	0	0	0
OsJN02633	TIGR	9638.m01438	EP	LH	8321154	-	3	4026	P	774.553	0.323	0.158
OsJN02634	TIGR	9638.m01733	HP	LH	10070947	-	2	541	P	887.323	0.429	0.6
OsJN02635	TIGR	9638.m01543	HP	LH	8924400	-	1	492	P	464.717	0.182	0
OsJN02636	TIGR	9638.m00170	EP	HH	1018078	-	4	3664	P	415.621	0.185	0.067
OsJN02637	TIGR	9638.m00595	HP	LH	3514179	-	5	3225	P	363.308	0.278	0.1
OsJN02638	TIGR	9638.m00860	PP	HH	4998057	-	8	5309	P	384.315	0.125	0
OsJN02639	TIGR	9638.m01539	HP	LH	8915299	-	4	949	P	678.556	0.286	0.154
OsJN02640	TIGR	9638.m01900	HP	LH	11155686	-	2	1751	P	224.864	0.125	0
OsJN02641	TIGR	9638.m01661	PP	HH	9616424	-	1	2424	P	380.309	0.038	0
OsJN02642	TIGR	9638.m00293	PP	HH	1729183	-	2	1951	A	0	0	0
OsJN02643	TIGR	9638.m00835	PP	HH	4855675	-	4	5704	P	535.268	0.061	0
OsJN02644	TIGR	9638.m00265	HP	LH	1531990	-	5	3714	P	684.185	0.3	0.07
OsJN02645	TIGR	9638.m01490	PP	HH	8635758	-	7	3206	P	476.568	0.157	0
OsJN02646	TIGR	9638.m00551	HP	LH	3254719	-	6	3191	P	260.666	0.026	0
OsJN02647	TIGR	9638.m00700	HP	LH	4157826	-	2	409	P	382.091	0.444	0
OsJN02648	TIGR	9638.m00347	PP	LH	2046640	-	1	1512	P	587.362	0.091	0
OsJN02649	TIGR	9638.m00201	HP	LH	1200148	-	1	426	A	0	0	0
OsJN02650	TIGR	9638.m01793	PP	HH	10490644	-	9	4966	P	658.191	0.192	0.055
OsJN02651	TIGR	9638.m00061	EP	HH	365126	-	16	4270	P	537.212	0.083	0.063
OsJN02652	TIGR	9638.m00194	PP	HH	1147063	-	2	1567	P	333.415	0.088	0
OsJN02653	TIGR	9638.m00585	HP	LH	3448695	-	1	354	P	1084.344	0.375	0
OsJN02654	TIGR	9638.m00442	HP	LH	2613606	-	1	384	A	0	0	0
OsJN02655	TIGR	9638.m00013	EP	HH	84583	-	7	2905	P	411.122	0.122	0
OsJN02656	TIGR	9638.m00124	EP	HH	745327	-	8	4537	P	560.075	0.075	0
OsJN02657	TIGR	9638.m00299	PP	HH	1759251	-	10	7435	P	842.743	0.095	0.062
OsJN02658	TIGR	9638.m01042	HP	LH	6070382	-	1	609	P	243.16	0.071	0
OsJN02659	TIGR	9638.m01694	HP	LH	9825883	-	2	1791	P	454.458	0.389	0.118
OsJN02660	TIGR	9638.m00304	HP	LH	1784205	-	4	2038	A	0	0	0.059
OsJN02661	TIGR	9638.m01522	HP	LH	8838313	-	1	546	P	1115.85	0.091	0
OsJN02662	TIGR	9638.m00190	PP	HH	1124341	-	2	1232	P	442.611	0.074	0
OsJN02663	TIGR	9638.m00162	EP	HH	923230	-	1	1318	P	620.089	0.138	0
OsJN02664	TIGR	9638.m01129	HP	LH	6583732	-	1	570	P	1306.892	0.615	0
OsJN02665	TIGR	9638.m00367	EP	HH	2162755	-	7	4488	P	707.379	0.146	0.054
OsJN02666	TIGR	9638.m01806	PP	HH	10549027	-	2	2079	P	760.904	0.075	0
OsJN02667	TIGR	9638.m01563	HP	LH	9046584	-	4	1072	A	0	0	0
OsJN02668	TIGR	9638.m01403	HP	LH	8104750	-	3	1387	P	880.709	0.444	0.091
OsJN02669	TIGR	9638.m00962	HP	LH	5668298	-	3	2015	P	1321.022	0.333	0
OsJN02670	TIGR	9638.m00528	PP	HH	3097980	-	9	5041	P	893.247	0.15	0.065
OsJN02671	TIGR	9638.m00638	EP	HH	3778100	-	4	1048	P	464.325	0.211	0
OsJN02672	TIGR	9638.m00855	EP	HH	4971726	-	4	3642	P	968.666	0.176	0.067
OsJN02673	TIGR	9638.m00407	EP	LH	2400238	-	2	400	P	366.741	0.222	0
OsJN02674	TIGR	9638.m01683	EP	HH	9745232	-	5	3278	P	502.847	0.125	0.093
OsJN02675	TIGR	9638.m00717	HP	LH	4262163	-	2	287	P	1098.449	0.5	0
OsJN02676	TIGR	9638.m01510	PP	LH	8761627	-	1	330	A	0	0	0
OsJN02677	TIGR	9638.m01506	HP	LH	8736659	-	1	306	P	709.008	0.286	0
OsJN02678	TIGR	9638.m00981	HP	LH	5755669	-	2	1790	P	787.07	0.286	0.032
OsJN02679	TIGR	9638.m00829	HP	LH	4818941	-	2	634	P	842.847	0.3	0
OsJN02680	TIGR	9638.m01928	HP	LH	11331811	-	5	2351	P	732.802	0.194	0.308
OsJN02681	TIGR	9638.m00472	PP	HH	2778782	-	2	1698	P	651.847	0.241	0
OsJN02682	TIGR	9638.m00886	EP	HH	5196574	-	5	4635	A	0	0	0
OsJN02683	TIGR	9638.m01165	HP	LH	6796629	-	1	804	A	1	0	0

OsJN02684	TIGR	9638.m01461	HP	LH	8469239	-	3	1592	P	687.432	0.353	0.105
OsJN02685	TIGR	9638.m00874	HP	LH	5086047	-	1	342	A	0	0	0
OsJN02686	TIGR	9638.m00916	PP	LH	5374380	-	1	1251	P	897.014	0.179	0
OsJN02687	TIGR	9638.m01866	PP	HH	10941533	-	1	483	P	223.768	0.091	0
OsJN02688	TIGR	9638.m01596	HP	LH	9230692	-	2	771	P	1057.733	0.25	0
OsJN02689	TIGR	9638.m00706	HP	LH	4200470	-	2	1059	P	942.97	0.412	0
OsJN02690	TIGR	9638.m01329	HP	LH	7694539	-	2	672	P	429.093	0.231	0
OsJN02691	TIGR	9638.m00183	PP	HH	1076942	-	4	4157	P	639.82	0.114	0.018
OsJN02692	TIGR	9638.m01746	PP	HH	10133941	-	2	2674	P	385.797	0.065	0.083
OsJN02693	TIGR	9638.m01040	HP	LH	6065954	-	5	875	A	0	0	0
OsJN02694	TIGR	9638.m00878	HP	LH	5126536	-	1	207	P	496.451	0.2	0
OsJN02695	TIGR	9638.m00005	HP	LH	22818	-	5	1413	P	1109.118	0.067	0
OsJN02696	TIGR	9638.m01833	PP	HH	10719088	-	4	3083	P	362.56	0.115	0.1
OsJN02697	TIGR	9638.m01577	HP	LH	9141754	-	2	362	P	1573.636	0.167	0
OsJN02698	TIGR	9638.m00884	EP	LH	5177948	-	7	3166	P	246.766	0.026	0
OsJN02699	TIGR	9638.m01679	EP	HH	9727749	-	6	2638	P	762.937	0.095	0.067
OsJN02700	TIGR	9638.m00416	EP	LH	2455726	-	2	875	P	753.134	0.333	0
OsJN02701	TIGR	9638.m01585	HP	LH	9171482	-	2	915	A	0	0	0
OsJN02702	TIGR	9638.m01798	HP	LH	10513286	-	2	595	P	224.739	0.143	0
OsJN02703	TIGR	9638.m00488	EP	HH	2881764	-	2	1443	A	206.145	0.037	0
OsJN02704	TIGR	9638.m01639	EP	HH	9508046	-	17	5154	P	579.794	0.108	0.026
OsJN02705	TIGR	9638.m00356	EP	LH	2091903	-	2	1431	A	0	0	0
OsJN02706	TIGR	9638.m01213	HP	LH	7108400	-	4	2276	A	0	0	0.286
OsJN02707	TIGR	9638.m01718	HP	LH	9989342	-	2	397	A	0	0	0
OsJN02708	TIGR	9638.m00285	PP	HH	1691965	-	9	6771	A	0	0	0.025
OsJN02709	TIGR	9638.m01034	HP	HH	6043150	-	2	1752	A	0	0	0.063
OsJN02710	TIGR	9638.m00273	PP	HH	1606379	-	12	12141	P	264.927	0.037	0.042
OsJN02711	TIGR	9638.m00495	EP	HH	2926287	-	2	826	P	360.816	0.125	0
OsJN02712	TIGR	9638.m01271	HP	HH	7385725	-	2	364	A	0	0	0
OsJN02713	TIGR	9638.m00042	PP	HH	223326	-	4	2977	P	211.91	0.045	0.171
OsJN02714	TIGR	9638.m00859	EP	HH	4991531	-	2	1434	P	565.775	0.12	0.143
OsJN02715	TIGR	9638.m01103	HP	LH	6454427	-	1	1344	P	575.661	0.069	0
OsJN02716	TIGR	9638.m00463	PP	LH	2715883	-	2	2574	P	960.878	0.081	0
OsJN02717	TIGR	9638.m01853	PP	HH	10873085	-	10	5604	A	0	0	0
OsJN02718	TIGR	9638.m01146	PP	HH	6679997	-	7	6338	P	672.209	0.063	0.054
OsJN02719	TIGR	9638.m01869	HP	LH	10967428	-	2	1039	P	1247.112	0.353	0.25
OsJN02720	TIGR	9638.m00015	EP	HH	94369	-	3	2281	P	527.001	0.129	0.105
OsJN02721	TIGR	9638.m00998	EP	HH	5865779	-	13	5160	P	1030.875	0.054	0
OsJN02722	TIGR	9638.m00336	EP	HH	1982984	-	3	983	P	419.677	0.053	0
OsJN02723	TIGR	9638.m01210	HP	HH	7088253	-	3	2026	A	0	0	0
OsJN02724	TIGR	9638.m00892	EP	HH	5254407	-	20	12659	P	539.391	0.054	0.041
OsJN02725	TIGR	9638.m01772	PP	HH	10351613	-	1	426	A	0	0	0
OsJN02726	TIGR	9638.m00327	PP	HH	1933446	-	2	3515	P	1282.749	0.051	0.067
OsJN02727	TIGR	9638.m01818	EP	HH	10615835	-	3	1775	P	258.2	0.118	0.045
OsJN02728	TIGR	9638.m01813	HP	LH	10580144	-	1	393	P	681.759	0.111	0
OsJN02729	TIGR	9638.m00234	HP	LH	1368467	-	2	1256	P	484.232	0.143	0.111
OsJN02730	TIGR	9638.m01709	PP	HH	9907094	-	12	7287	P	347.566	0.086	0.02
OsJN02731	TIGR	9638.m00640	EP	HH	3785240	-	4	2724	P	1154.004	0.12	0.057
OsJN02732	TIGR	9638.m01787	HP	LH	10460893	-	3	2073	P	665.646	0.111	0.037
OsJN02733	TIGR	9638.m00399	HP	LH	2356329	-	2	795	A	0	0	0
OsJN02734	TIGR	9638.m01224	HP	LH	7163336	-	2	667	P	1144.435	0.125	0.2
OsJN02735	TIGR	9638.m01505	HP	LH	8733650	-	2	1077	A	0	0	0.125
OsJN02736	TIGR	9638.m00566	HP	LH	3324700	-	1	447	P	1047.019	0.182	0
OsJN02737	TIGR	9638.m01690	HP	LH	9793021	-	5	1526	A	0	0	0.043
OsJN02738	TIGR	9638.m00669	EP	LH	3988654	-	2	639	P	735.743	0.222	0
OsJN02739	TIGR	9638.m00938	EP	HH	5530973	-	1	666	P	683.285	0.267	0

OsJN02740	TIGR	9638.m00782	EP	HH	4585985	-	2	1903	P	633.709	0.143	0
OsJN02741	TIGR	9638.m00378	HP	LH	2215292	-	2	425	P	603.929	0.286	0
OsJN02742	TIGR	9638.m00570	PP	HH	3342167	-	4	2533	P	923.947	0.226	0
OsJN02743	TIGR	9638.m01567	HP	LH	9068466	-	4	1345	A	0	0	0
OsJN02744	TIGR	9638.m01270	PP	HH	7381863	-	9	2868	A	0	0	0
OsJN02745	TIGR	9638.m01086	PP	HH	6337099	-	2	6150	A	0	0	0
OsJN02746	TIGR	9638.m00006	HP	LH	29403	-	5	2730	P	615.598	0.118	0
OsJN02747	TIGR	9638.m00781	HP	HH	4576576	-	1	447	A	0	0	0
OsJN02748	TIGR	9638.m00035	HP	LH	185392	-	6	1803	P	437.134	0.074	0
OsJN02749	TIGR	9638.m01601	HP	LH	9257836	-	1	357	A	0	0	0
OsJN02750	TIGR	9638.m01681	EP	LH	9737299	-	1	465	A	0	0	0
OsJN02751	TIGR	9638.m00280	PP	HH	1665239	-	4	3134	P	638.427	0.049	0.04
OsJN02752	TIGR	9638.m00174	HP	LH	1038975	-	4	2222	P	1176.619	0.042	0.08
OsJN02753	TIGR	9638.m01888	PP	HH	11077671	-	3	1675	P	540.279	0.129	0
OsJN02754	TIGR	9638.m01762	HP	LH	10275679	-	2	616	A	0	0	0.375
OsJN02755	TIGR	9638.m01699	HP	LH	9845753	-	2	763	P	873.631	0.357	0
OsJN02756	TIGR	9638.m01674	HP	LH	9704364	-	2	2243	P	1115.019	0.333	0.111
OsJN02757	TIGR	9638.m00328	EP	HH	1938792	-	3	2310	P	852.895	0.143	0.136
OsJN02758	TIGR	9638.m00339	HP	LH	2016910	-	2	353	P	1492.013	0.333	0.5
OsJN02759	TIGR	9638.m00127	EP	HH	772269	-	5	2887	P	633.419	0.175	0.056
OsJN02760	TIGR	9638.m00578	HP	LH	3418413	-	1	255	A	0	0	0
OsJN02761	TIGR	9638.m01101	HP	LH	6446645	-	6	2579	P	537.749	0.111	0.205
OsJN02762	TIGR	9638.m01885	HP	LH	11051203	-	8	5911	P	716.412	0.086	0.063
OsJN02763	TIGR	9638.m01737	HP	LH	10087090	-	2	268	A	0	0	0
OsJN02764	TIGR	9638.m00725	EP	HH	4296746	-	16	4298	P	542.551	0.356	0.061
OsJN02765	TIGR	9638.m00155	EP	HH	923230	-	2	1780	P	620.089	0.114	0
OsJN02766	TIGR	9638.m00067	HP	LH	409018	-	1	402	P	663.561	0.6	0
OsJN02767	TIGR	9638.m00986	PP	HH	5783273	-	3	2312	A	0	0	0
OsJN02768	TIGR	9638.m01597	HP	LH	9234018	-	4	2251	P	549.621	0.3	0.077
OsJN02769	TIGR	9638.m01119	HP	LH	6528794	-	2	317	P	982.529	0.143	0
OsJN02770	TIGR	9638.m00117	HP	LH	708907	-	2	947	P	601.529	0.111	0.167
OsJN02771	TIGR	9638.m01544	EP	HH	8927169	-	2	2282	P	594.361	0.121	0
OsJN02772	TIGR	9638.m01496	HP	LH	8674841	-	1	198	P	462.199	0.2	0
OsJN02773	TIGR	9638.m00423	HP	LH	2484960	-	2	715	P	753.006	0.6	0
OsJN02774	TIGR	9638.m01462	EP	HH	8471192	-	3	1115	P	529.998	0.091	0
OsJN02775	TIGR	9638.m01415	HP	LH	8188580	-	1	465	A	0	0	0
OsJN02776	TIGR	9638.m01893	PP	HH	11101059	-	3	3842	P	621.277	0.094	0.065
OsJN02777	TIGR	9638.m00917	PP	LH	5377574	-	1	1347	A	0	0	0
OsJN02778	TIGR	9638.m00825	HP	LH	4797774	-	2	913	P	243.083	0.083	0.143
OsJN02779	TIGR	9638.m01729	PP	HH	10059070	-	1	719	A	0	0	0
OsJN02780	TIGR	9638.m01609	HP	LH	9300142	-	1	192	P	1398.562	0.25	0
OsJN02781	TIGR	9638.m00979	HP	LH	5749531	-	3	1022	P	398.949	0.375	0.143
OsJN02782	TIGR	9638.m00637	PP	LH	3769862	-	2	1683	P	283.233	0.045	0
OsJN02783	TIGR	9638.m00033	HP	LH	178208	-	1	345	P	304.156	0.5	0
OsJN02784	TIGR	9638.m01482	HP	LH	8572568	-	4	1899	P	803.015	0.286	0.08
OsJN02785	TIGR	9638.m01283	PP	HH	7434065	-	6	3807	P	515.174	0.139	0.021
OsJN02786	TIGR	9638.m01204	PP	HH	7045196	-	3	4776	P	676.889	0.119	0.017
OsJN02787	TIGR	9638.m01799	HP	LH	10517077	-	3	3574	P	504.25	0.25	0.038
OsJN02788	TIGR	9638.m00903	HP	LH	5311375	-	4	1984	P	735.443	0.111	0
OsJN02789	TIGR	9638.m03958	EP	HH	285959	-	10	6236	P	947.625	0.143	0.033
OsJN02790	TIGR	9638.m01896	PP	HH	11126047	-	10	4263	P	726.615	0.231	0
OsJN02791	TIGR	9638.m00466	PP	LH	2727507	-	3	5043	P	635.56	0.139	0.013
OsJN02792	TIGR	9638.m01214	PP	HH	7112837	-	10	7039	A	0	0	0.086
OsJN02793	TIGR	9638.m00890	EP	HH	5234208	-	19	9081	P	518.148	0.074	0
OsJN02794	TIGR	9638.m00526	PP	HH	3097980	-	9	5041	P	960.198	0.163	0.07
OsJN02795	TIGR	9638.m00289	HP	LH	1710567	-	1	822	P	480.492	0.176	0

OsJN02796	TIGR	9638.m01832	PP	HH	10717214	-	3	1635	A	0	0	0
OsJN02797	TIGR	9638.m00978	PP	HH	5747742	-	3	1493	A	0	0	0.056
OsJN02798	TIGR	9638.m00396	HP	LH	2334551	-	4	4102	P	760.302	0.137	0.026
OsJN02799	TIGR	9638.m00069	EP	HH	416542	-	2	1127	P	898.823	0.045	0
OsJN02800	TIGR	9638.m01682	EP	HH	9738564	-	5	1370	P	903.255	0.059	0.077
OsJN02801	TIGR	9638.m01453	HP	LH	8426030	-	3	1132	A	0	0	0
OsJN02802	TIGR	9638.m00275	PP	LH	1631135	-	5	3078	A	0	0	0.132
OsJN02803	TIGR	9638.m01864	HP	HH	10930098	-	9	2980	P	486.179	0.104	0
OsJN02804	TIGR	9638.m00858	EP	HH	4982295	-	6	4409	P	585.115	0.128	0
OsJN02805	TIGR	9638.m01861	HP	LH	10915689	-	5	1578	P	225.746	0.053	0
OsJN02806	TIGR	9638.m01617	HP	LH	9337036	-	2	271	A	0	0	0
OsJN02807	TIGR	9638.m00387	EP	LH	2273329	-	3	3587	P	553.541	0.043	0.018
OsJN02808	TIGR	9638.m01574	EP	HH	9117063	-	3	1248	A	0	0	0
OsJN02809	TIGR	9638.m01877	HP	LH	11018978	-	1	306	P	611.549	0.143	0
OsJN02810	TIGR	9638.m01745	PP	HH	10121855	-	7	4332	P	923.808	0.122	0.04
OsJN02811	TIGR	9638.m01698	HP	LH	9839861	-	2	252	P	1552.709	0.167	0
OsJN02812	TIGR	9638.m01657	PP	HH	9597716	-	1	2403	P	844.629	0.019	0
OsJN02813	TIGR	9638.m01012	EP	HH	5929404	-	5	3950	P	504.412	0.113	0
OsJN02814	TIGR	9638.m01669	EP	HH	9670470	-	3	2722	P	986.074	0.077	0
OsJN02815	TIGR	9638.m00661	EP	LH	3925347	-	2	695	P	942.97	0.143	0
OsJN02816	TIGR	9638.m00429	PP	HH	2509203	-	11	6474	P	514.377	0.045	0.042
OsJN02817	TIGR	9638.m00937	EP	HH	5523383	-	1	639	A	0	0	0
OsJN02818	TIGR	9638.m00572	EP	HH	3361679	-	1	978	P	1395.868	0.091	0
OsJN02819	TIGR	9638.m00385	HP	LH	2262697	-	2	895	P	1071.675	0.2	0.1
OsJN02820	TIGR	9638.m00812	HP	LH	4720189	-	2	1395	P	311.075	0.053	0
OsJN02821	TIGR	9638.m00172	EP	LH	1032793	-	3	1831	A	203.453	0.042	0
OsJN02822	TIGR	9638.m00668	HP	HH	3975934	-	2	1641	P	434.093	0.176	0.118
OsJN02823	TIGR	9638.m00342	HP	LH	2027814	-	2	553	A	0	0	0
OsJN02824	TIGR	9638.m00114	HP	LH	679327	-	2	563	A	0	0	0
OsJN02825	TIGR	9638.m01058	HP	LH	6156774	-	2	506	P	612.79	0.1	0
OsJN02826	TIGR	9638.m00621	EP	LH	3685750	-	2	1763	P	894.494	0.294	0
OsJN02827	TIGR	9638.m00902	HP	LH	5304520	-	3	3382	A	0	0	0.03
OsJN02828	TIGR	9638.m00139	EP	HH	824276	-	7	3721	P	548.694	0.077	0
OsJN02829	TIGR	9638.m00529	EP	HH	3106166	-	3	5163	P	428.502	0.021	0.016
OsJN02830	TIGR	9638.m01191	HP	LH	6960575	-	1	249	A	0	0	0
OsJN02831	TIGR	9638.m00126	EP	LH	766450	-	5	1497	P	589.866	0.167	0.143
OsJN02832	TIGR	9638.m00756	HP	HH	4447478	-	1	1803	P	543.07	0.158	0
OsJN02833	TIGR	9638.m00681	HP	LH	4049772	-	5	2851	P	669.353	0.237	0
OsJN02834	TIGR	9638.m01517	HP	LH	8804694	-	1	212	A	0	0	0
OsJN02835	TIGR	9638.m01323	HP	LH	7657237	-	2	729	A	192.699	0.063	1
OsJN02836	TIGR	9638.m01084	PP	HH	6321922	-	7	5875	P	336.863	0.125	0.2
OsJN02837	TIGR	9638.m00931	EP	HH	5490339	-	1	657	P	386.448	0.133	0
OsJN02838	TIGR	9638.m00876	PP	HH	5098977	-	3	6735	A	0	0	0.029
OsJN02839	TIGR	9638.m01002	HP	LH	5890380	-	5	3064	P	325.181	0.13	0.07
OsJN02840	TIGR	9638.m00914	PP	LH	5367469	-	1	975	P	566.014	0.182	0
OsJN02841	TIGR	9638.m01545	HP	LH	8930231	-	1	216	P	1235.669	0.8	0
OsJN02842	TIGR	9638.m00708	HP	LH	4219282	-	2	1451	P	605.45	0.286	0.133
OsJN02843	TIGR	9638.m01370	HP	LH	7888606	-	1	642	A	0	0	0
OsJN02844	TIGR	9638.m01033	PP	HH	6042009	-	1	513	P	956.875	0.091	0
OsJN02845	TIGR	9638.m00636	PP	LH	3763786	-	3	1982	P	303.699	0.031	0
OsJN02846	TIGR	9638.m00517	HP	LH	3052054	-	2	763	P	1220.119	0.2	0
OsJN02847	TIGR	9638.m01050	PP	HH	6107114	-	5	5686	P	479.72	0.07	0.333
OsJN02848	TIGR	9638.m00780	EP	HH	4571481	-	3	2642	P	904.839	0.103	0.37
OsJN02849	TIGR	9638.m00975	PP	HH	5736903	-	9	7673	P	387.923	0.113	0.009
OsJN02850	TIGR	9638.m00807	HP	LH	4701346	-	2	647	A	0	0	0
OsJN02851	TIGR	9638.m00489	HP	LH	2886197	-	1	351	A	0	0	0

OsJN02852	TIGR	9638.m01735	PP	HH	10083245	-	1	662	A	0	0	0
OsJN02853	TIGR	9638.m01656	HP	LH	9594229	-	3	1341	P	543.538	0.063	0
OsJN02854	TIGR	9638.m00113	PP	HH	673620	-	5	2374	P	885.845	0.098	0
OsJN02855	TIGR	9638.m00828	PP	HH	4814544	-	4	3147	P	562.725	0.077	0.034
OsJN02856	TIGR	9638.m00302	HP	LH	1773889	-	3	1094	A	0	0	0
OsJN02857	TIGR	9638.m00790	HP	LH	4608765	-	5	3224	P	1044.599	0.16	0.044
OsJN02858	TIGR	9638.m01628	HP	LH	9391785	-	2	1460	P	617.338	0.222	0.167
OsJN02859	TIGR	9638.m01357	EP	HH	7833602	-	8	9351	P	363.445	0.018	0.069
OsJN02860	TIGR	9638.m01530	HP	LH	8876222	-	2	1040	P	880.517	0.167	0
OsJN02861	TIGR	9638.m00199	EP	HH	1177087	-	10	7355	P	525.128	0.087	0.018
OsJN02862	TIGR	9638.m01542	HP	HH	8923078	-	2	606	P	949.366	0.214	0
OsJN02863	TIGR	9638.m01489	PP	HH	8622327	-	7	6088	P	412.989	0.082	0.012
OsJN02864	TIGR	9638.m01887	HP	HH	11066943	-	5	5568	P	765.62	0.21	0.034
OsJN02865	TIGR	9638.m01856	HP	LH	10888660	-	7	3908	P	719.348	0.136	0.032
OsJN02866	TIGR	9638.m00078	HP	LH	461661	-	2	318	A	0	0	0
OsJN02867	TIGR	9638.m01805	PP	HH	10549027	-	3	4665	P	760.904	0.068	0.018
OsJN02868	TIGR	9638.m01710	HP	LH	9915499	-	1	264	P	414.063	0.167	0
OsJN02869	TIGR	9638.m00771	HP	LH	4524759	-	2	340	P	568.874	0.5	0
OsJN02870	TIGR	9638.m00422	HP	LH	2481496	-	2	273	A	0	0	0
OsJN02871	TIGR	9638.m00245	HP	LH	1422416	-	2	1059	P	864.958	0.5	0.111
OsJN02872	TIGR	9638.m00200	HP	LH	1198322	-	2	689	A	0	0	0
OsJN02873	TIGR	9638.m01356	EP	HH	7829195	-	9	13824	P	542.761	0.016	0.084
OsJN02874	TIGR	9638.m01258	EP	LH	7332558	-	1	377	P	689.722	0.111	0
OsJN02875	TIGR	9638.m01133	HP	LH	6602105	-	1	235	P	469.819	0.6	0
OsJN02876	TIGR	9638.m01730	PP	HH	10062435	-	1	414	A	0	0	0
OsJN02877	TIGR	9638.m00291	HP	LH	1725135	-	1	391	P	448.363	0.4	0
OsJN02878	TIGR	9638.m00282	PP	LH	1679067	-	4	2935	P	515.023	0.091	0
OsJN02879	TIGR	9638.m01812	HP	LH	10573733	-	2	733	A	0	0	0.1
OsJN02880	TIGR	9638.m01778	PP	HH	10377387	-	4	2457	P	1759.975	1	1
OsJN02881	TIGR	9638.m00447	EP	LH	2627495	-	3	1851	P	447.557	0.077	0.333
OsJN02882	TIGR	9638.m01902	PP	LH	11162722	-	7	6697	P	455.2	0.14	0
OsJN02883	TIGR	9638.m01413	HP	LH	8174943	-	2	2503	P	546.435	0.25	0.038
OsJN02884	TIGR	9638.m01240	EP	HH	7254406	-	3	1151	P	707.059	0.13	0
OsJN02885	TIGR	9638.m00404	EP	LH	2392841	-	3	549	P	474.214	0.1	0
OsJN02886	TIGR	9638.m00247	PP	HH	1431711	-	13	3785	P	760.002	0.043	0.028
OsJN02887	TIGR	9638.m00298	HP	LH	1755158	-	5	2171	A	0	0	0
OsJN02888	TIGR	9638.m00936	HP	LH	5519529	-	3	1843	P	814.779	0.111	0.067
OsJN02889	TIGR	9638.m00465	PP	LH	2724538	-	1	1418	P	415.378	0.097	0
OsJN02890	TIGR	9638.m00497	PP	LH	2934404	-	3	785	P	360.135	0.071	0
OsJN02891	TIGR	9638.m00036	HP	LH	193043	-	1	528	P	930.561	0.538	0
OsJN02892	TIGR	9638.m01789	PP	LH	10469041	-	2	2439	P	614.46	0.25	0
OsJN02893	TIGR	9638.m00492	EP	LH	2905913	-	2	1913	A	0	0	0
OsJN02894	TIGR	9638.m00354	EP	LH	2082436	-	1	1434	P	668.615	0.063	0
OsJN02895	TIGR	9638.m00454	HP	LH	2660546	-	2	1968	P	1829.314	0.333	0.214
OsJN02896	TIGR	9638.m01590	HP	LH	9197096	-	3	1619	P	307.926	0.063	0
OsJN02897	TIGR	9638.m01216	HP	LH	7123283	-	2	942	A	0	0	0
OsJN02898	TIGR	9638.m01126	HP	LH	6577764	-	1	327	P	734.394	0.2	0
OsJN02899	TIGR	9638.m00746	HP	LH	4405013	-	1	273	P	286.201	0.143	0
OsJN02900	TIGR	9638.m01691	EP	HH	9797586	-	8	2740	P	686.643	0.083	0
OsJN02901	TIGR	9638.m00915	HP	LH	5371065	-	3	1682	A	0	0	0.074
OsJN02902	TIGR	9638.m00017	HP	LH	101922	-	2	668	P	653.604	0.2	0.4
OsJN02903	TIGR	9638.m01192	PP	HH	6965317	-	11	4002	P	731.651	0.087	0.098
OsJN02904	TIGR	9638.m01093	HP	LH	6380431	-	2	661	P	706.446	0.444	0
OsJN02905	TIGR	9638.m00317	PP	HH	1872637	-	6	5504	P	572.188	0.111	0.051
OsJN02906	TIGR	9638.m01716	HP	LH	9969758	-	2	875	P	1293.512	0.182	0.111
OsJN02907	TIGR	9638.m00468	EP	LH	2744760	-	5	1308	P	365.164	0.071	0.154

OsJN02908	TIGR	9638.m01802	PP	LH	10531620	-	6	4923	P	750.675	0.089	0.081
OsJN02909	TIGR	9638.m01162	EP	HH	6771742	-	1	1409	A	1	0	0
OsJN02910	TIGR	9638.m00800	HP	LH	4667130	-	5	3422	A	0	0	0.135
OsJN02911	TIGR	9638.m01773	EP	HH	10354814	-	1	1543	P	1133.664	1	0
OsJN02912	TIGR	9638.m01917	HP	LH	11275079	-	1	468	P	476.241	0.182	0
OsJN02913	TIGR	9638.m00301	PP	HH	1769228	-	4	2452	P	259.07	0.057	0
OsJN02914	TIGR	9638.m01494	HP	LH	8672190	-	1	825	P	554.873	0.056	0
OsJN02915	TIGR	9638.m01748	HP	HH	10149288	-	2	1671	P	1282.78	0.077	0.083
OsJN02916	TIGR	9638.m01602	HP	LH	9258369	-	2	2441	P	812.591	0.308	0.122
OsJN02917	TIGR	9638.m00176	EP	LH	1045166	-	1	439	A	0	0	0
OsJN02918	TIGR	9638.m00107	PP	HH	639763	-	3	3279	P	374.609	0.281	0.031
OsJN02919	TIGR	9638.m00461	EP	LH	2700245	-	3	2800	P	642.026	0.036	0
OsJN02920	TIGR	9638.m01882	HP	LH	11036133	-	1	411	P	875.683	0.1	0
OsJN02921	TIGR	9638.m01666	HP	LH	9643102	-	2	691	P	725.604	0.2	0
OsJN02922	TIGR	9638.m00028	HP	LH	153665	-	4	2079	A	0	0	0
OsJN02923	TIGR	9638.m00053	EP	LH	305385	-	1	174	P	920.767	0.4	0
OsJN02924	TIGR	9638.m01759	HP	LH	10245123	-	3	2540	P	601.728	0.188	0.061
OsJN02925	TIGR	9638.m01611	PP	HH	9310929	-	7	1724	P	617.837	0.038	0
OsJN02926	TIGR	9638.m00309	PP	HH	1817351	-	1	1578	P	313.615	0.086	0
OsJN02927	TIGR	9638.m00320	PP	HH	1888518	-	2	4946	P	703.855	0.069	0
OsJN02928	TIGR	9638.m00129	EP	HH	790183	-	5	3715	P	305.962	0.023	0.03
OsJN02929	TIGR	9638.m01503	HP	LH	8717589	-	2	1070	P	1083.944	0.167	0.118
OsJN02930	TIGR	9638.m00157	EP	LH	943510	-	2	594	A	0	0	0
OsJN02931	TIGR	9638.m01844	EP	HH	10801330	-	12	11687	P	481.047	0.051	0.035
OsJN02932	TIGR	9638.m00402	HP	LH	2385812	-	4	2751	P	374.719	0.056	0
OsJN02933	TIGR	9638.m00784	HP	LH	4589521	-	2	700	P	614.882	0.444	0
OsJN02934	TIGR	9638.m00662	EP	LH	3933607	-	2	582	P	474.409	0.154	0
OsJN02935	TIGR	9638.m00629	HP	LH	3729737	-	2	560	A	0	0	0
OsJN02936	TIGR	9638.m00198	EP	LH	1173961	-	4	1975	P	616.637	0.227	0
OsJN02937	TIGR	9638.m00653	HP	LH	3859839	-	3	1846	A	0	0	0
OsJN02938	TIGR	9638.m00065	EP	HH	390910	-	8	9285	P	451.193	0.112	0.01
OsJN02939	TIGR	9638.m00281	PP	HH	1672478	-	3	2894	P	751.699	0.024	0
OsJN02940	TIGR	9638.m01435	HP	LH	8313455	-	1	387	A	0	0	0
OsJN02941	TIGR	9638.m00830	HP	HH	4820177	-	2	1489	P	520.553	0.083	0.125
OsJN02942	TIGR	9638.m00355	HP	LH	2085311	-	2	469	P	530.898	0.2	0
OsJN02943	TIGR	9638.m00269	PP	LH	1546755	-	5	2728	P	744.317	0.049	0
OsJN02944	TIGR	9638.m00318	HP	LH	1880223	-	2	358	A	0	0	0
OsJN02945	TIGR	9638.m00881	PP	HH	5143743	-	4	3436	P	728.447	0.105	0.056
OsJN02946	TIGR	9638.m01809	HP	LH	10557760	-	3	1105	P	245.316	0.111	0.2
OsJN02947	TIGR	9638.m01092	EP	LH	6377445	-	4	2514	P	1122.774	0.2	0.059
OsJN02948	TIGR	9638.m01079	HP	LH	6290790	-	5	2351	P	635.407	0.071	0
OsJN02949	TIGR	9638.m01372	HP	LH	7896039	-	1	642	A	0	0	0
OsJN02950	TIGR	9638.m00474	PP	HH	2788682	-	5	2971	P	873.304	0.3	0.069
OsJN02951	TIGR	9638.m00329	HP	LH	1943063	-	1	159	P	464.341	0.5	0
OsJN02952	TIGR	9638.m00134	EP	HH	808972	-	4	1484	P	599.268	0.095	0
OsJN02953	TIGR	9638.m00448	EP	LH	2631639	-	2	1254	P	1016.821	0.063	0.1
OsJN02954	TIGR	9638.m00287	HP	LH	1701314	-	1	561	P	826.12	0.462	0
OsJN02955	TIGR	9638.m01481	HP	LH	8567654	-	9	3187	A	0	0	0
OsJN02956	TIGR	9638.m00536	HP	LH	3172469	-	4	1421	P	517.601	0.038	0
OsJN02957	TIGR	9638.m00724	EP	HH	4296746	-	16	4298	P	542.551	0.356	0.061
OsJN02958	TIGR	9638.m00504	EP	LH	2966002	-	2	717	A	0	0	0
OsJN02959	TIGR	9638.m01143	PP	LH	6664074	-	6	5610	P	265.025	0.03	0.045
OsJN02960	TIGR	9638.m00009	HP	LH	59580	-	2	896	P	1208.926	0.25	0.167
OsJN02961	TIGR	9638.m01257	HP	HH	7326772	-	1	285	A	0	0	0
OsJN02962	TIGR	9638.m00809	HP	HH	4712055	-	1	414	A	0	0	0
OsJN02963	TIGR	9638.m00290	EP	HH	1713490	-	9	10783	P	709.998	0.108	0.03

OsJN02964	TIGR	9638.m00775	HP	LH	4547591	-	1	201	A	0	0	0
OsJN02965	TIGR	9638.m00110	PP	HH	661284	-	4	1743	P	507.351	0.207	0
OsJN02966	TIGR	9638.m01920	HP	LH	11280847	-	5	2004	P	227.195	0.1	0
OsJN02967	TIGR	9638.m00163	HP	LH	965066	-	1	131	P	1167.352	0.5	0
OsJN02968	TIGR	9638.m00059	PP	HH	356643	-	2	3219	P	695.222	0.261	0.045
OsJN02969	TIGR	9638.m00271	HP	LH	1572005	-	4	2640	P	1008.034	0.133	0.095
OsJN02970	TIGR	9638.m01082	PP	HH	6307194	-	4	5408	P	292.193	0.095	0.054
OsJN02971	TIGR	9638.m01650	PP	HH	9572074	-	2	2181	A	1	0	0
OsJN02972	TIGR	9638.m00776	HP	LH	4554884	-	5	1624	A	0	0	0.167
OsJN02973	TIGR	9638.m01317	HP	LH	7626423	-	2	1003	P	293.549	0.111	0.1
OsJN02974	TIGR	9638.m00044	HP	HH	234964	-	1	615	P	1219.137	0.143	0
OsJN02975	TIGR	9638.m00308	PP	HH	1812944	-	1	1581	P	557.736	0.086	0
OsJN02976	TIGR	9638.m00783	EP	HH	4586937	-	1	953	P	553.107	0.19	0
OsJN02977	TIGR	9638.m00753	HP	LH	4432442	-	5	5279	P	501.746	0.4	0.132
OsJN02978	TIGR	9638.m00413	EP	HH	2438081	-	1	1789	P	549.208	0.154	0
OsJN02979	TIGR	9638.m00797	EP	LH	4651232	-	1	661	P	774.348	0.286	0
OsJN02980	TIGR	9638.m01487	PP	LH	8596750	-	1	1647	P	731.898	0.139	0
OsJN02981	TIGR	9638.m01894	HP	LH	11113730	-	1	134	P	701.904	0.5	0
OsJN02982	TIGR	9638.m00633	PP	HH	3750090	-	2	1380	P	765.077	0.125	0.043
OsJN02983	TIGR	9638.m00205	EP	HH	1227625	-	5	2183	P	910.371	0.022	0
OsJN02984	TIGR	9635.m01435	HP	LH	236921	-	1	267	P	1361.872	0.167	0
OsJN02985	TIGR	9638.m01815	HP	LH	10590143	-	8	5652	P	391.292	0.19	0.033
OsJN02986	TIGR	9638.m01830	EP	HH	10687879	-	4	3656	P	554.964	0.045	0.063
OsJN02987	TIGR	9638.m00645	HP	LH	3829131	-	1	300	P	1208.217	0.714	0
OsJN02988	TIGR	9638.m01837	HP	LH	10743884	-	1	273	P	1578.924	0.143	0
OsJN02989	TIGR	9638.m00080	PP	HH	473085	-	1	534	P	488.355	0.182	0
OsJN02990	TIGR	9638.m01520	PP	HH	8829707	-	2	4346	A	0	0	0
OsJN02991	TIGR	9638.m00128	EP	HH	776905	-	4	6610	P	726.79	0.073	0.037
OsJN02992	TIGR	9638.m01824	HP	LH	10658799	-	1	195	A	0	0	0
OsJN02993	TIGR	9638.m01803	PP	LH	10539904	-	2	3681	P	256.428	0.133	0.063
OsJN02994	TIGR	9638.m01136	PP	HH	6619413	-	1	837	A	0	0	0
OsJN02995	TIGR	9638.m00045	HP	LH	236921	-	1	267	P	1361.872	0.167	0
OsJN02996	TIGR	9638.m00847	EP	LH	4925855	-	2	1639	P	817.093	0.231	0.059
OsJN02997	TIGR	9638.m00521	HP	LH	3068711	-	3	935	P	248.267	0.125	0.077
OsJN02998	TIGR	9638.m01527	PP	HH	8857142	-	4	4745	P	226.148	0.088	0.143
OsJN02999	TIGR	9638.m01899	PP	HH	11150642	-	4	4358	P	554.315	0.067	0
OsJN03000	TIGR	9638.m00379	EP	LH	2223365	-	2	1626	P	282.339	0.067	0.048
OsJN03001	TIGR	9638.m00335	HP	LH	1971334	-	2	2000	P	1265.995	0.067	0.069
OsJN03002	TIGR	9638.m01420	HP	LH	8216784	-	4	2500	A	0	0	0.194
OsJN03003	TIGR	9638.m00574	EP	HH	3378090	-	1	978	P	498.839	0.091	0
OsJN03004	TIGR	9638.m00588	EP	HH	3459984	-	6	2261	P	669.91	0.111	0.043
OsJN03005	TIGR	9638.m00409	PP	LH	2406220	-	3	4493	P	306.528	0.111	0
OsJN03006	TIGR	9638.m01060	PP	LH	6170689	-	10	2976	A	194.41	0.027	0.037
OsJN03007	TIGR	9638.m00420	HP	LH	2478021	-	1	867	P	865.052	0.421	0
OsJN03008	TIGR	9638.m01796	HP	LH	10507098	-	1	276	P	547.845	0.286	0
OsJN03009	TIGR	9638.m00879	HP	HH	5127664	-	2	544	P	279.852	0.071	0
OsJN03010	TIGR	9638.m00832	HP	LH	4837710	-	1	486	P	398.91	0.143	0
OsJN03011	TIGR	9638.m01751	EP	HH	10158372	-	2	1037	P	582.932	0.111	0.2
OsJN03012	TIGR	9638.m00252	HP	LH	1463841	-	1	513	P	782.94	0.364	0
OsJN03013	TIGR	9638.m01671	HP	LH	9686009	-	17	4456	P	349.823	0.018	0
OsJN03014	TIGR	9638.m01282	PP	HH	7431408	-	8	6464	P	550.88	0.091	0.055
OsJN03015	TIGR	9638.m01717	PP	HH	9977982	-	4	3902	P	755.097	0.196	0
OsJN03016	TIGR	9638.m01763	HP	LH	10279627	-	1	393	P	330.828	0.222	0
OsJN03017	TIGR	9638.m01117	HP	HH	6517479	-	5	4319	P	578.013	0.128	0.036
OsJN03018	TIGR	9638.m00985	PP	HH	5776773	-	5	2931	P	437.143	0.029	0
OsJN03019	TIGR	9634.m04452	PP	LH	8100810	-	2	2181	P	193.004	1	0

OsJN03020	BGIJ	Chr10_2661	UG	LH	17463041	+	2	906	P	4486.217	0.286	0.833
OsJN03021	BGIJ	Chr10_2314	UG	HH	15070243	+	3	1893	P	1063.293	0.219	0
OsJN03022	BGIJ	Chr10_2443	UG	LH	15964099	+	1	963	P	2003.331	0.727	0
OsJN03023	BGIJ	AK106572	CG	HH	14917859	+	9	4874	P	734.541	0.075	0.037
OsJN03024	BGIJ	AK101865	CG	HH	18047253	+	14	4523	P	1141.3	0.104	0.059
OsJN03025	BGIJ	Chr10_2419	UG	HH	15794156	+	4	2265	P	978.525	0.154	0.083
OsJN03026	BGIJ	Chr10_2915	UG	LH	19077593	+	1	288	P	1242.257	0.429	0
OsJN03027	BGIJ	AK071802	CG	LH	22570803	+	1	1612	P	1730.888	0.4	0
OsJN03028	BGIJ	Chr10_2406	UG	HH	15669823	+	4	2971	P	2287.582	0.138	0.029
OsJN03029	BGIJ	AK066946	CG	LH	16504643	+	1	570	P	738.356	0.583	0
OsJN03030	BGIJ	AK059629	CG	LH	17359074	+	13	7435	P	785.938	0.135	0.032
OsJN03031	BGIJ	AK066654	CG	LH	13687358	+	1	3937	P	1332.013	0.141	0
OsJN03032	BGIJ	AK073483	CG	HH	21450580	+	1	2018	P	1337.458	0.089	0
OsJN03033	BGIJ	Chr10_3279	UG	HH	21274561	+	2	1080	P	1359.634	0.182	0
OsJN03034	BGIJ	Chr10_2243	UG	HH	14708528	+	2	1277	P	1455.205	0.278	0
OsJN03035	BGIJ	AK066147	CG	LH	16844810	+	3	4332	P	1461.227	0.147	0.071
OsJN03036	BGIJ	AK059482	CG	LH	19090069	+	2	1286	P	1294.192	0.2	0
OsJN03037	BGIJ	Chr10_1848	UG	LH	12031295	+	6	2152	P	799.486	0.19	0
OsJN03038	BGIJ	Chr10_2936	UG	LH	19219796	+	2	246	P	1304.96	0.333	0
OsJN03039	BGIJ	AK063546	CG	LH	21510184	+	3	4212	A	613.021	0.132	0.056
OsJN03040	BGIJ	Chr10_3082	UG	LH	20105980	+	2	702	P	1883.972	0.2	0
OsJN03041	BGIJ	AK070800	CG	LH	18759574	+	6	13715	A	0	0	0.063
OsJN03042	BGIJ	AK107989	CG	LH	21710998	+	1	382	P	1047.516	0.111	0
OsJN03043	BGIJ	AK070073	CG	LH	22023996	+	8	5790	P	1579.586	0.113	0.137
OsJN03044	BGIJ	AK062625	CG	LH	16932179	+	2	1372	A	446.202	0.133	0.375
OsJN03045	BGIJ	AK101468	CG	LH	13892230	+	2	2753	P	1030.352	0.147	0
OsJN03046	BGIJ	Chr10_2625	UG	LH	17136339	+	4	2063	P	3208.105	0.111	0.136
OsJN03047	BGIJ	Chr10_3216	UG	HH	20883874	+	1	687	P	3406.52	0.214	0
OsJN03048	BGIJ	Chr10_2671	UG	LH	17576978	+	2	178	P	3564.227	0.5	0
OsJN03049	BGIJ	AK066785	CG	LH	21079245	+	5	2239	P	1556.088	0.129	0.059
OsJN03050	BGIJ	AK063684	CG	LH	21283176	+	2	920	P	1181.341	0.333	0
OsJN03051	BGIJ	Chr10_1909	UG	LH	12399537	+	5	2134	P	1139.376	0.286	0
OsJN03052	BGIJ	Chr10_2995	EG	LH	19612040	+	1	1116	P	945.049	0.24	0
OsJN03053	BGIJ	Chr10_3091	UG	HH	20136501	+	4	3095	P	2722.652	0.158	0
OsJN03054	BGIJ	Chr10_2352	UG	LH	15304103	+	2	280	P	3922.715	0.667	0
OsJN03055	BGIJ	Chr10_1844	UG	HH	12000946	+	9	6501	P	1700.729	0.139	0.13
OsJN03056	BGIJ	Chr10_2598	UG	LH	16899235	+	2	838	P	1968.343	0.333	0.417
OsJN03057	BGIJ	Chr10_2154	UG	LH	14162696	+	2	297	P	4185.334	0.25	0.667
OsJN03058	BGIJ	Chr10_2262	UG	HH	14773730	+	6	3896	P	1593.133	0.185	0.067
OsJN03059	BGIJ	AK106252	CG	LH	16089564	+	1	1400	P	658.389	0.194	0
OsJN03060	BGIJ	Chr10_2802	EG	HH	18369052	+	3	2738	P	1453.212	0.071	0.089
OsJN03061	BGIJ	Chr10_1874	UG	LH	12190470	+	6	3037	P	965.193	0.097	0.053
OsJN03062	BGIJ	AK110909	CG	LH	19534890	+	1	1652	P	729.071	0.162	0
OsJN03063	BGIJ	Chr10_3166	UG	LH	20567528	+	4	1498	P	1064.557	0.077	0.1
OsJN03064	BGIJ	AK064083	CG	LH	22013116	+	2	1907	P	1522.011	0.156	0
OsJN03065	BGIJ	Chr10_1999	UG	HH	12971441	+	3	3777	P	1318.617	0.148	0
OsJN03066	BGIJ	AK061311	CG	LH	14967662	+	4	2774	P	1145.359	0.172	0.156
OsJN03067	BGIJ	Chr10_2161	UG	HH	14225091	+	1	4836	P	1816.695	0.181	0
OsJN03068	BGIJ	AK109772	CG	LH	14668634	+	3	1534	P	1310.643	0.143	0
OsJN03069	BGIJ	Chr10_3390	EG	LH	22008081	+	3	2277	P	1282.865	0.556	0.083
OsJN03070	BGIJ	Chr10_2522	UG	HH	16444579	+	3	2381	P	940.812	0.118	0.053
OsJN03071	BGIJ	Chr10_1953	UG	LH	12712101	+	2	1052	P	1358.586	0.286	0
OsJN03072	BGIJ	Chr10_2377	UG	LH	15468489	+	3	1047	P	1292.01	0.308	0
OsJN03073	BGIJ	Chr10_2325	UG	HH	15149505	+	8	6190	P	1891.906	0.304	0.045
OsJN03074	BGIJ	AK109287	CG	LH	13734722	+	3	2204	P	1085.132	0.238	0.037
OsJN03075	BGIJ	Chr10_1809	UG	HH	11812789	+	2	1070	P	2919.758	0.238	0

OsJN03076	BGIJ	Chr10_3406	UG	HH	22085882	+	1	2436	P	883.564	0.056	0
OsJN03077	BGIJ	Chr10_2362	UG	HH	15363241	+	4	4362	P	1557.265	0.048	0.031
OsJN03078	BGIJ	Chr10_2515	UG	LH	16408555	+	3	2205	P	950.661	0.067	0.161
OsJN03079	BGIJ	Chr10_1773	EG	HH	11585887	+	5	2462	A	1	0	0
OsJN03080	BGIJ	Chr10_2887	UG	HH	18896012	+	3	2012	P	1695.499	0.114	0
OsJN03081	BGIJ	Chr10_2599	UG	LH	16910686	+	1	504	P	843.413	0.083	0
OsJN03082	BGIJ	Chr10_2924	UG	LH	19128695	+	3	1158	P	1533.301	0.571	0.364
OsJN03083	BGIJ	Chr10_2439	UG	HH	15944514	+	4	3008	P	1836.296	0.222	0
OsJN03084	BGIJ	AK061153	CG	LH	17950142	+	4	3291	P	1115.862	0.171	0
OsJN03085	BGIJ	Chr10_3493	UG	LH	22638017	+	2	741	P	931.648	0.067	1
OsJN03086	BGIJ	Chr10_3123	UG	LH	20309443	+	2	1321	P	1452.263	0.5	0.111
OsJN03087	BGIJ	Chr10_2861	UG	LH	18715025	+	1	459	P	1506.516	0.1	0
OsJN03088	BGIJ	AK070882	CG	LH	20927399	+	2	1247	P	1653.849	0.158	0
OsJN03089	BGIJ	Chr10_3193	UG	LH	20738544	+	1	366	P	2793.937	0.75	0
OsJN03090	BGIJ	Chr10_2663	EG	LH	17472868	+	2	1284	P	2831.554	0.182	0.133
OsJN03091	BGIJ	AK064207	CG	LH	21594329	+	1	1951	P	1337.656	0.05	0
OsJN03092	BGIJ	Chr10_3407	UG	LH	22089620	+	2	366	P	2371.053	0.333	0
OsJN03093	BGIJ	Chr10_3110	UG	LH	20238803	+	2	89	P	2394.709	0.5	0
OsJN03094	BGIJ	AK106568	CG	LH	14860400	+	10	3758	P	1602.915	0.286	0.026
OsJN03095	BGIJ	Chr10_2028	UG	LH	13161027	+	2	349	P	978.186	0.333	0.4
OsJN03096	BGIJ	AK100517	CG	LH	21971294	+	5	3482	P	906.95	0.101	0
OsJN03097	BGIJ	Chr10_3348	UG	HH	21721751	+	4	2948	P	736.104	0.054	0.037
OsJN03098	BGIJ	Chr10_2615	UG	LH	17073664	+	5	1559	P	985.354	0.111	0.048
OsJN03099	BGIJ	Chr10_3196	UG	LH	20765971	+	2	346	P	2093.421	0.333	0
OsJN03100	BGIJ	AK069463	CG	LH	14781734	+	1	2824	P	1109.89	0.05	0
OsJN03101	BGIJ	Chr10_1998	UG	HH	12970185	+	1	468	P	1329.553	0.273	0
OsJN03102	BGIJ	AK106808	CG	LH	20556272	+	4	5371	P	1994.341	0.231	0.057
OsJN03103	BGIJ	Chr10_2150	UG	HH	14129567	+	4	2928	P	792.637	0.038	0.083
OsJN03104	BGIJ	AK070183	CG	LH	22552204	+	1	2574	P	1288.99	0.071	0
OsJN03105	BGIJ	Chr10_2415	UG	HH	15760228	+	9	9380	P	1544.536	0.139	0.132
OsJN03106	BGIJ	Chr10_2094	UG	LH	13767561	+	3	704	P	1006.369	0.111	0
OsJN03107	BGIJ	AK103845	CG	LH	20289939	+	1	3009	P	1431.017	0.077	0
OsJN03108	BGIJ	Chr10_633	UG	HH	4094630	+	1	1323	P	1275.631	0.069	0
OsJN03109	BGIJ	AK069171	CG	HH	1864082	+	1	4720	P	1697.006	0.18	0
OsJN03110	BGIJ	Chr10_829	UG	HH	5340156	+	10	5844	P	1076.76	0.108	0.066
OsJN03111	BGIJ	Chr10_632	UG	HH	4089460	+	2	1725	P	1458.189	0.108	0
OsJN03112	BGIJ	AK062606	CG	LH	11168804	+	2	727	P	1799.358	0.154	0
OsJN03113	BGIJ	AK063055	CG	LH	10575918	+	2	7282	P	2097.107	0.167	0.153
OsJN03114	BGIJ	Chr10_202	UG	LH	1302207	+	8	2461	P	1016.808	0.171	0
OsJN03115	BGIJ	Chr10_1634	UG	HH	10620340	+	7	4260	P	1064.195	0.152	0.102
OsJN03116	BGIJ	Chr10_1009	UG	HH	6574159	+	6	2554	P	1084.377	0.029	0.182
OsJN03117	BGIJ	Chr10_816	UG	LH	5270897	+	5	3475	P	1086.827	0.239	0.077
OsJN03118	BGIJ	Chr10_597	UG	LH	3849595	+	2	285	P	2843.595	0.333	0.333
OsJN03119	BGIJ	AK107108	CG	LH	29072	+	1	791	P	3027.9	0.353	0
OsJN03120	BGIJ	AK064425	CG	LH	2035583	+	1	1780	P	861.378	0.132	0
OsJN03121	BGIJ	Chr10_1064	UG	LH	6937775	+	6	2235	P	1202.524	0.154	0.043
OsJN03122	BGIJ	Chr10_1010	UG	LH	6582164	+	4	1211	P	1058.106	0.235	0.5
OsJN03123	BGIJ	Chr10_650	UG	LH	4225875	+	4	1725	P	1281.92	0.125	0.125
OsJN03124	BGIJ	AK068977	CG	LH	4401082	+	6	3091	P	647.837	0.14	0
OsJN03125	BGIJ	AK111323	CG	LH	2297027	+	1	782	P	1256.835	0.294	0
OsJN03126	BGIJ	Chr10_1661	UG	HH	10842358	+	15	6386	P	1141.613	0.083	0
OsJN03127	BGIJ	AK109165	CG	LH	9426843	+	1	556	P	1767.603	0.25	0
OsJN03128	BGIJ	Chr10_295	UG	HH	1839251	+	15	10420	P	1230.623	0.087	0.181
OsJN03129	BGIJ	AK069162	CG	LH	2538126	+	3	3441	P	1732.887	0.167	0.102
OsJN03130	BGIJ	Chr10_130	UG	LH	847134	+	2	226	P	3076.72	0.25	0.5
OsJN03131	BGIJ	AK060146	CG	LH	8919628	+	1	1105	P	942.173	0.083	0

OsJN03132	BGIJ	AK108086	CG	LH	1199351	+	1	600	A	527.577	0.071	0
OsJN03133	BGIJ	AK061084	CG	LH	11035944	+	1	996	P	851.839	0.273	0
OsJN03134	BGIJ	Chr10_271	UG	LH	1706334	+	2	627	P	3358.785	0.333	0.333
OsJN03135	BGIJ	Chr10_1698	UG	LH	11062873	+	1	264	P	835.343	0.333	0
OsJN03136	BGIJ	AK066625	CG	LH	774552	+	1	970	P	686.854	0.091	0
OsJN03137	BGIJ	Chr10_1513	UG	HH	9754733	+	6	3405	P	887.525	0.045	0.059
OsJN03138	BGIJ	Chr10_1013	UG	LH	6593982	+	6	2315	P	971.792	0.146	0
OsJN03139	BGIJ	Chr10_1077	UG	LH	7054090	+	5	2187	P	1732.217	0.118	0.032
OsJN03140	BGIJ	AK103056	CG	LH	513574	+	5	4175	P	709.69	0.069	0
OsJN03141	BGIJ	Chr10_1683	UG	LH	10969398	+	5	3968	P	2271.44	0.25	0.172
OsJN03142	BGIJ	AK065698	CG	LH	7315696	+	2	6512	P	1121.521	0.143	0.122
OsJN03143	BGIJ	AK107098	CG	LH	1463677	+	2	2462	P	1617.906	0.135	0.188
OsJN03144	BGIJ	AK066528	CG	LH	5371249	+	1	1784	P	585.822	0.135	0
OsJN03145	BGIJ	Chr10_1448	UG	LH	9288320	+	2	860	P	1700.79	0.143	0.375
OsJN03146	BGIJ	AK070457	CG	HH	5952500	+	7	5970	P	859.58	0.222	0.021
OsJN03147	BGIJ	Chr10_1469	UG	HH	9430532	+	6	3639	P	941.172	0.152	0
OsJN03148	BGIJ	AK063350	CG	HH	1218214	+	15	7604	P	969.515	0.082	0.054
OsJN03149	BGIJ	AK062736	CG	LH	842857	+	1	408	P	1610.961	0.75	0
OsJN03150	BGIJ	Chr10_169	UG	HH	1099003	+	6	1761	P	1096.009	0.037	0
OsJN03151	BGIJ	Chr10_639	UG	LH	4132540	+	2	508	P	1671.249	0.5	0.111
OsJN03152	BGIJ	Chr10_1699	UG	LH	11064244	+	1	606	P	1114.594	0.385	0
OsJN03153	BGIJ	AK069512	CG	LH	9658857	+	2	2077	P	1052.35	0.163	0
OsJN03154	BGIJ	AK102469	CG	HH	2003051	+	8	7708	P	1116.078	0.194	0.015
OsJN03155	BGIJ	AK066649	CG	LH	4893000	+	1	817	P	845.556	0.333	0
OsJN03156	BGIJ	Chr10_877	UG	LH	5660807	+	1	228	P	1510.195	0.167	0
OsJN03157	BGIJ	Chr10_448	UG	HH	2860113	+	5	6412	P	710.638	0.083	0.071
OsJN03158	BGIJ	Chr10_1696	UG	LH	11044334	+	2	1477	P	1625.844	0.118	0
OsJN03159	BGIJ	Chr10_654	UG	HH	4245476	+	6	3500	P	1436.291	0.103	0
OsJN03160	BGIJ	AK072061	CG	LH	4328091	+	2	2618	P	999.953	0.2	0
OsJN03161	BGIJ	AK064417	CG	HH	3412378	+	5	4186	P	805.592	0.143	0.086
OsJN03162	BGIJ	Chr10_600	UG	LH	3862434	+	10	6367	P	1103.525	0.154	0.134
OsJN03163	BGIJ	Chr10_1287	UG	HH	8316897	+	1	900	P	1028.756	0.3	0
OsJN03164	BGIJ	Chr10_133	UG	LH	857826	+	1	663	P	1471.101	0.571	0
OsJN03165	BGIJ	Chr10_1506	UG	HH	9722664	+	2	1046	P	943.472	0.15	0
OsJN03166	BGIJ	Chr10_16	UG	LH	104816	+	3	1253	P	1750.849	0.5	0.2
OsJN03167	BGIJ	Chr10_1192	UG	LH	7726339	+	1	714	P	2184.577	0.5	0
OsJN03168	BGIJ	Chr10_1735	UG	LH	11325668	+	3	1405	P	1931.368	0.143	0.111
OsJN03169	BGIJ	AK069896	CG	LH	3358147	+	1	882	P	1030.345	0.25	0
OsJN03170	BGIJ	AK062808	CG	LH	9317957	+	1	643	P	1326.828	0.333	0
OsJN03171	BGIJ	Chr10_21	UG	HH	137335	+	2	1368	P	1400.655	0.043	0
OsJN03172	BGIJ	AK111448	CG	LH	8104825	+	1	1679	P	965.797	0.353	0
OsJN03173	BGIJ	AK102683	CG	LH	1897477	+	2	2496	P	1102.945	0.173	0
OsJN03174	BGIJ	Chr10_1078	UG	HH	7058257	+	11	7044	P	765.026	0.087	0.104
OsJN03175	BGIJ	AK065387	CG	LH	3635329	+	4	2320	P	1196.829	0.065	0
OsJN03176	BGIJ	Chr10_1505	UG	HH	9721434	+	2	684	P	1255.632	0.188	0
OsJN03177	BGIJ	AK063188	CG	LH	6212641	+	1	385	A	0	0	0
OsJN03178	BGIJ	Chr10_726	UG	LH	4648345	+	2	332	P	1162.433	0.667	0
OsJN03179	BGIJ	AK106577	CG	LH	10443931	+	2	3151	P	1226.264	0.095	0
OsJN03180	BGIJ	Chr10_1357	UG	HH	8764618	+	3	3192	P	1644.869	0.148	0.222
OsJN03181	BGIJ	AK062541	CG	LH	9230978	+	2	696	P	2035.091	0.077	0
OsJN03182	BGIJ	Chr10_1108	UG	HH	7266098	+	9	4692	P	1053.48	0.143	0.06
OsJN03183	BGIJ	Chr10_1290	UG	HH	8339210	+	4	3327	P	1081.049	0.109	0.167
OsJN03184	BGIJ	Chr10_349	UG	HH	2199523	+	5	4343	P	1286.771	0.153	0.043
OsJN03185	BGIJ	Chr10_1579	UG	HH	10162863	+	11	9052	P	1620.687	0.075	0.069
OsJN03186	BGIJ	Chr10_1235	UG	HH	7946050	+	1	4431	P	1054.689	0.188	0
OsJN03187	BGIJ	Chr10_988	UG	HH	6406483	+	8	4553	P	1088.487	0.101	0.182

OsJN03188	BGIJ	Chr10_1351	UG	LH	8718575	+	2	318	P	1939.426	0.167	0
OsJN03189	BGIJ	Chr10_595	UG	HH	3843161	+	1	2295	P	1403.87	0.039	0
OsJN03190	BGIJ	AK072115	CG	LH	7065646	+	4	6849	P	1269.808	0.087	0.039
OsJN03191	BGIJ	AK059264	CG	LH	10035873	+	5	4874	A	405.986	0.048	0.043
OsJN03192	BGIJ	Chr10_808	UG	LH	5190890	+	2	324	P	2801.639	0.25	0.25
OsJN03193	BGIJ	Chr10_2814	UG	LH	18431726	-	2	790	P	1592.454	0.417	0.4
OsJN03194	BGIJ	Chr10_2508	UG	LH	16356000	-	1	621	P	1653.972	0.727	0
OsJN03195	BGIJ	Chr10_2239	UG	LH	14686021	-	1	243	P	1351.03	0.333	0
OsJN03196	BGIJ	Chr10_3478	UG	HH	22566814	-	1	1896	P	1773.148	0.095	0
OsJN03197	BGIJ	AK069676	CG	LH	14012538	-	4	7121	P	1233.294	0.148	0.062
OsJN03198	BGIJ	Chr10_3257	UG	HH	21141927	-	2	4520	P	1295.258	0.133	0
OsJN03199	BGIJ	Chr10_2949	UG	LH	19309459	-	2	1680	P	803.586	0.2	0.125
OsJN03200	BGIJ	AK100194	CG	LH	13187331	-	2	1559	P	1954.512	0.375	0
OsJN03201	BGIJ	Chr10_2290	UG	HH	14958163	-	1	675	P	1615.028	0.2	0
OsJN03202	BGIJ	AK062550	CG	LH	22196203	-	1	722	P	1095.34	0.25	0
OsJN03203	BGIJ	AK108900	CG	LH	20142815	-	1	966	P	1786.714	0.333	0
OsJN03204	BGIJ	Chr10_2757	UG	HH	18100616	-	7	3360	P	723.945	0.073	0.105
OsJN03205	BGIJ	AK111394	CG	LH	18565367	-	3	4610	A	528.446	0.176	0.078
OsJN03206	BGIJ	Chr10_2514	UG	LH	16401084	-	5	4667	P	2079.258	0.152	0.103
OsJN03207	BGIJ	Chr10_2799	UG	LH	18349247	-	1	285	P	962.136	0.286	0
OsJN03208	BGIJ	Chr10_1995	UG	HH	12960040	-	1	927	P	2554.664	0.333	0
OsJN03209	BGIJ	Chr10_3297	UG	LH	21428316	-	2	1123	P	3356.438	0.4	0.222
OsJN03210	BGIJ	Chr10_1821	UG	HH	11877688	-	3	1660	P	1285.619	0.097	0
OsJN03211	BGIJ	Chr10_3013	UG	LH	19715655	-	1	561	P	2123.176	0.667	0
OsJN03212	BGIJ	Chr10_3053	UG	HH	19981966	-	2	1181	P	1750.575	0.333	0.286
OsJN03213	BGIJ	Chr10_1743	UG	HH	11364680	-	2	1319	P	765.591	0.125	0.111
OsJN03214	BGIJ	AK111426	CG	LH	22563529	-	6	3284	P	3274.422	0.139	0.028
OsJN03215	BGIJ	Chr10_1795	UG	LH	11752194	-	2	1918	P	1322.114	0.615	0.462
OsJN03216	BGIJ	Chr10_3048	EG	HH	19946156	-	2	949	P	930.012	0.357	0
OsJN03217	BGIJ	AK107522	CG	LH	17234563	-	4	8875	P	1334.097	0.235	0.055
OsJN03218	BGIJ	AK064129	CG	LH	11747894	-	3	3063	P	2781.626	0.2	0.087
OsJN03219	BGIJ	Chr10_2122	UG	HH	13938668	-	3	1453	P	776.164	0.111	0
OsJN03220	BGIJ	AK064310	CG	LH	15928134	-	1	1065	P	5999.324	0.917	0
OsJN03221	BGIJ	Chr10_1871	UG	HH	12173029	-	2	1389	P	1143.947	0.067	0.067
OsJN03222	BGIJ	AK111392	CG	LH	20653271	-	1	769	P	1476.212	0.059	0
OsJN03223	BGIJ	Chr10_2619	UG	LH	17100591	-	1	234	P	2773.392	0.167	0
OsJN03224	BGIJ	Chr10_3462	UG	HH	22449777	-	2	1110	P	775.6	0.091	0
OsJN03225	BGIJ	Chr10_3421	UG	HH	22174285	-	2	1933	P	1333.575	0.069	0
OsJN03226	BGIJ	Chr10_3055	UG	LH	19987103	-	1	180	P	1301.488	0.4	0
OsJN03227	BGIJ	Chr10_2759	UG	HH	18112425	-	16	7949	P	1508.915	0.09	0.024
OsJN03228	BGIJ	Chr10_2649	UG	HH	17319735	-	6	4637	P	962.155	0.077	0.028
OsJN03229	BGIJ	Chr10_1851	UG	HH	12056411	-	4	2004	P	1254.353	0.032	0.1
OsJN03230	BGIJ	Chr10_2672	UG	LH	17579960	-	1	306	P	3016.855	0.375	0
OsJN03231	BGIJ	AK069167	CG	LH	19789836	-	1	1198	P	1800.565	0.286	0
OsJN03232	BGIJ	AK111429	CG	HH	20130381	-	1	1401	P	2330.25	0.129	0
OsJN03233	BGIJ	Chr10_2449	EG	LH	15993808	-	1	558	P	3733.542	0.692	0
OsJN03234	BGIJ	Chr10_2497	UG	HH	16300755	-	3	2563	P	1652.216	0.157	0.167
OsJN03235	BGIJ	Chr10_2653	UG	HH	17372691	-	7	4049	P	1601.37	0.241	0.02
OsJN03236	BGIJ	Chr10_3039	UG	LH	19897719	-	2	772	P	2145.983	0.571	0
OsJN03237	BGIJ	Chr10_2927	UG	LH	19162371	-	3	3633	P	901.72	0.444	0.134
OsJN03238	BGIJ	Chr10_2996	UG	LH	19621603	-	2	1113	P	1094.186	0.182	0
OsJN03239	BGIJ	AK108669	CG	HH	13817156	-	1	1645	P	914.261	0.139	0
OsJN03240	BGIJ	AK070735	CG	LH	14927587	-	5	3311	P	1501.549	0.219	0
OsJN03241	BGIJ	AK066983	CG	LH	15699147	-	1	980	P	1319.48	0.3	0
OsJN03242	BGIJ	AK103373	CG	HH	21966055	-	7	2349	P	1893.43	0.159	0
OsJN03243	BGIJ	Chr10_2448	EG	LH	15990685	-	1	558	P	3293.287	0.769	0

OsJN03244	BGIJ	AK107092	CG	LH	20094080	-	1	2091	P	2187.936	0.222	0
OsJN03245	BGIJ	Chr10_2451	EG	LH	16000201	-	1	813	P	1563.361	0.412	0
OsJN03246	BGIJ	AK100196	CG	LH	17852626	-	2	867	P	1312.481	0.389	0
OsJN03247	BGIJ	AK058488	CG	LH	12683787	-	2	737	P	1183.193	0.143	0
OsJN03248	BGIJ	Chr10_2650	UG	LH	17326668	-	9	2492	P	1925.659	0.194	0
OsJN03249	BGIJ	Chr10_2336	UG	HH	15203082	-	4	3271	P	1153.866	0.13	0
OsJN03250	BGIJ	Chr10_1952	UG	LH	12708057	-	2	1905	P	1045.034	0.152	0
OsJN03251	BGIJ	Chr10_3162	UG	LH	20536665	-	2	1704	P	1258.019	0.25	0.103
OsJN03252	BGIJ	AK063328	CG	LH	15985619	-	10	815	P	3404.59	0.667	0
OsJN03253	BGIJ	AK101643	CG	LH	15653982	-	7	5240	P	2294.163	0.091	0.05
OsJN03254	BGIJ	AK068897	CG	LH	16533031	-	2	1382	P	705.415	0.345	0
OsJN03255	BGIJ	AK106383	CG	LH	18199609	-	2	1788	P	1149.075	0.282	0
OsJN03256	BGIJ	Chr10_3443	UG	LH	22322064	-	1	225	P	2231.145	0.4	0
OsJN03257	BGIJ	AK072513	CG	LH	13032381	-	2	1048	P	1526.092	0.125	0
OsJN03258	BGIJ	Chr10_2316	UG	HH	15079441	-	7	3591	P	1403.146	0.019	0
OsJN03259	BGIJ	Chr10_2293	UG	LH	14971933	-	2	1011	P	1518.635	0.25	0.143
OsJN03260	BGIJ	AK103350	CG	LH	15975419	-	1	934	P	3824.383	0.8	0
OsJN03261	BGIJ	AK068643	CG	LH	12533579	-	3	3013	P	1070.333	0.208	0
OsJN03262	BGIJ	Chr10_1983	UG	HH	12894007	-	3	4052	P	2104.136	0.085	0.167
OsJN03263	BGIJ	Chr10_2538	UG	LH	16525150	-	1	312	P	2222.538	0.5	0
OsJN03264	BGIJ	AK064476	CG	LH	11836813	-	8	5086	P	636.89	0.225	0.176
OsJN03265	BGIJ	AK107924	CG	LH	19095617	-	1	639	P	1945.165	0.133	0
OsJN03266	BGIJ	AK058209	CG	HH	13820820	-	2	1275	P	2400.838	0.615	0.067
OsJN03267	BGIJ	AK062758	CG	LH	17473747	-	2	1322	P	1553.449	0.083	0.077
OsJN03268	BGIJ	Chr10_1868	UG	HH	12154465	-	8	3797	P	1306.333	0.21	0.048
OsJN03269	BGIJ	Chr10_2057	UG	HH	13410639	-	9	6210	P	1207.083	0.14	0.098
OsJN03270	BGIJ	Chr10_1870	UG	LH	12168351	-	1	756	P	2141.325	0.118	0
OsJN03271	BGIJ	Chr10_2724	UG	LH	17915568	-	2	946	P	1909.866	0.333	0.385
OsJN03272	BGIJ	Chr10_2026	UG	HH	13148210	-	2	718	P	963.971	0.071	0
OsJN03273	BGIJ	AK070851	CG	LH	15925301	-	3	902	P	4384.722	0.786	1
OsJN03274	BGIJ	Chr10_2450	UG	LH	15997194	-	1	558	P	3173.02	0.769	0
OsJN03275	BGIJ	Chr10_3214	UG	LH	20871322	-	1	558	P	1726.183	0.385	0
OsJN03276	BGIJ	AK102661	CG	LH	15980351	-	1	1035	P	4266.26	0.739	0
OsJN03277	BGIJ	AK067954	CG	HH	22199336	-	7	4834	P	1619.046	0.125	0.061
OsJN03278	BGIJ	Chr10_2146	UG	HH	14108856	-	3	1514	P	2009.468	0.208	0
OsJN03279	BGIJ	AK063227	CG	LH	17689948	-	1	789	P	1998.803	0.529	0
OsJN03280	BGIJ	Chr10_3275	UG	HH	21239335	-	1	549	P	2082.803	0.083	0
OsJN03281	BGIJ	AK107069	CG	LH	14717318	-	1	2474	P	1425.233	0.059	0
OsJN03282	BGIJ	AK110769	CG	LH	18579932	-	1	1404	A	0	0	0
OsJN03283	BGIJ	Chr10_1926	EG	HH	12537079	-	1	939	A	0	0	0
OsJN03284	BGIJ	Chr10_3169	UG	LH	20583367	-	2	1008	P	1845.269	0.467	0.5
OsJN03285	BGIJ	AK067745	CG	HH	16162885	-	1	1183	P	1183.98	0.185	0
OsJN03286	BGIJ	Chr10_149	UG	HH	960884	-	2	1087	P	727.631	0.042	0
OsJN03287	BGIJ	AK109143	CG	LH	8746894	-	2	3515	P	437.662	0.057	0
OsJN03288	BGIJ	AK108427	CG	LH	5225558	-	1	1100	P	510.489	0.08	0
OsJN03289	BGIJ	AK064524	CG	LH	145145	-	2	886	A	0	0	0
OsJN03290	BGIJ	AK064164	CG	LH	7689260	-	1	555	P	443.673	0.25	0
OsJN03291	BGIJ	Chr10_1277	UG	LH	8254494	-	3	1029	P	504.739	0.067	0
OsJN03292	BGIJ	Chr10_1379	UG	HH	8903647	-	4	3878	P	459.805	0.043	0.077
OsJN03293	BGIJ	AK070752	CG	LH	3737544	-	3	3911	P	411.279	0.059	0
OsJN03294	BGIJ	Chr10_1183	UG	HH	7666325	-	4	3750	P	315.321	0.058	0.455
OsJN03295	BGIJ	Chr10_172	UG	LH	1115068	-	1	267	P	1116.739	0.143	0
OsJN03296	BGIJ	Chr10_1501	UG	HH	9693446	-	7	3561	P	295.932	0.036	0
OsJN03297	BGIJ	Chr10_609	UG	HH	3934702	-	9	7845	P	850.213	0.078	0.206
OsJN03298	BGIJ	Chr10_181	UG	HH	1170493	-	2	2309	P	728.567	0.082	0
OsJN03299	BGIJ	Chr10_73	UG	HH	466830	-	6	4981	P	1157.031	0.065	0

OsJN03300	BGIJ	Chr10_480	UG	LH	3064239	-	2	1911	P	798.755	0.059	0.167
OsJN03301	BGIJ	Chr10_1254	UG	LH	8090174	-	3	1681	P	638.604	0.133	0.136
OsJN03302	BGIJ	Chr10_83	UG	HH	544492	-	2	1256	P	713.889	0.1	0
OsJN03303	BGIJ	Chr10_740	UG	HH	4714996	-	4	1674	P	730.207	0.138	0
OsJN03304	BGIJ	AK107154	CG	LH	11233348	-	3	1598	P	824.513	0.156	0
OsJN03305	BGIJ	Chr10_1318	UG	LH	8522613	-	2	187	P	407.892	0.25	0
OsJN03306	BGIJ	Chr10_1471	UG	LH	9490591	-	1	426	P	717.041	0.25	0
OsJN03307	BGIJ	Chr10_1142	UG	HH	7417980	-	1	408	P	585.527	0.111	0
OsJN03308	BGIJ	Chr10_1296	UG	LH	8376110	-	3	1216	P	445.542	0.063	0
OsJN03309	BGIJ	AK102437	CG	LH	4238486	-	1	825	P	1022.947	0.111	0
OsJN03310	BGIJ	AK111071	CG	LH	10249081	-	2	760	P	433.576	0.25	0
OsJN03311	BGIJ	Chr10_865	UG	LH	5591744	-	1	207	P	580.506	0.4	0
OsJN03312	BGIJ	Chr10_360	UG	LH	2265116	-	2	1756	P	611.647	0.25	0
OsJN03313	BGIJ	Chr10_584	UG	HH	3753511	-	4	2845	P	537.689	0.093	0.111
OsJN03314	BGIJ	AK063510	CG	LH	1733353	-	1	1917	A	0	0	0
OsJN03315	BGIJ	Chr10_881	UG	LH	5685704	-	3	1767	P	601.426	0.25	0.077
OsJN03316	BGIJ	Chr10_457	UG	HH	2920103	-	6	3848	P	726.599	0.103	0
OsJN03317	BGIJ	Chr10_1414	UG	LH	9109014	-	1	390	P	934.789	0.25	0
OsJN03318	BGIJ	Chr10_762	UG	HH	4846814	-	5	6173	P	368.458	0.094	0.167
OsJN03319	BGIJ	Chr10_54	UG	HH	361695	-	4	679	P	1080.203	0.083	0.25
OsJN03320	BGIJ	AK100686	CG	LH	991811	-	10	5254	P	586.042	0.159	0
OsJN03321	BGIJ	Chr10_1285	UG	HH	8309151	-	5	3998	P	500.433	0.083	0.091
OsJN03322	BGIJ	Chr10_414	UG	HH	2616802	-	2	1392	P	496.743	0.074	0
OsJN03323	BGIJ	Chr10_488	UG	LH	3125469	-	3	820	P	303.153	0.1	0
OsJN03324	BGIJ	Chr10_1417	UG	HH	9124710	-	6	4881	P	761.637	0.094	0
OsJN03325	BGIJ	Chr10_201	UG	HH	1295671	-	9	4941	P	710.49	0.081	0.088
OsJN03326	BGIJ	Chr10_1457	UG	HH	9347814	-	5	2821	P	479.859	0.1	0
OsJN03327	BGIJ	AK064432	CG	LH	9816225	-	1	1931	P	326.71	0.083	0
OsJN03328	BGIJ	Chr10_108	UG	HH	705810	-	2	464	P	370.779	0.083	0
OsJN03329	BGIJ	Chr10_694	UG	HH	4458551	-	2	3307	P	403.665	0.016	0
OsJN03330	BGIJ	AK063230	CG	LH	10583961	-	1	640	P	761.439	0.2	0
OsJN03331	BGIJ	AK099670	CG	LH	7574364	-	6	4432	P	399.593	0.153	0
OsJN03332	BGIJ	AK066810	CG	LH	8327419	-	1	2221	P	452.806	0.082	0
OsJN03333	BGIJ	Chr10_75	UG	HH	484510	-	9	6844	P	1214.259	0.027	0.018
OsJN03334	BGIJ	Chr10_1259	UG	LH	8136758	-	1	387	P	384.085	0.625	0
OsJN03335	BGIJ	AK110742	CG	HH	1739609	-	4	2608	A	0	0	0
OsJN03336	BGIJ	Chr10_544	UG	LH	3467606	-	1	792	P	746.148	0.375	0
OsJN03337	BGIJ	Chr10_92	UG	LH	586384	-	3	2799	P	943.531	0.054	0
OsJN03338	BGIJ	AK106742	CG	HH	8654525	-	12	5963	P	502.183	0.043	0.099
OsJN03339	BGIJ	Chr10_932	UG	LH	6030483	-	3	2958	P	980.868	0.273	0.065
OsJN03340	BGIJ	Chr10_1659	UG	LH	10835862	-	1	525	P	289.119	0.1	0
OsJN03341	BGIJ	Chr10_475	UG	HH	3031255	-	5	3117	P	333.172	0.208	0.077
OsJN03342	BGIJ	Chr10_1347	UG	HH	8686311	-	7	5414	P	525.393	0.195	0.067
OsJN03343	BGIJ	Chr10_1462	UG	LH	9377959	-	1	573	P	264.678	0.091	0
OsJN03344	BGIJ	Chr10_1410	UG	HH	9077850	-	3	2757	P	339.933	0.061	0
OsJN03345	BGIJ	AK106942	CG	LH	10884619	-	2	1518	P	559.438	0.088	0
OsJN03346	BGIJ	Chr10_287	UG	HH	1790876	-	6	2734	P	232.937	0.083	0
OsJN03347	BGIJ	Chr10_530	UG	LH	3380064	-	1	126	P	284.152	0.667	0
OsJN03348	BGIJ	Chr10_789	UG	HH	5018661	-	7	3278	P	588.369	0.131	0.1
OsJN03349	BGIJ	AK111078	CG	LH	5794791	-	4	4530	P	884.757	0.235	0.083
OsJN03350	BGIJ	AK068970	CG	LH	78970	-	1	2639	P	775.952	0.224	0
OsJN03351	BGIJ	Chr10_484	UG	HH	3094722	-	4	2764	P	434.026	0.034	0
OsJN03352	BGIJ	Chr10_1411	UG	HH	9081776	-	2	1098	P	561.717	0.071	0
OsJN03353	BGIJ	Chr10_1050	UG	HH	6849847	-	7	6227	P	682.145	0.107	0.071
OsJN03354	BGIJ	AK106938	CG	LH	10894009	-	1	1000	P	671.05	0.19	0
OsJN03355	BGIJ	Chr10_617	UG	HH	4013395	-	3	2508	P	333.108	0.091	0

OsJN03356	BGIJ	Chr10_1253	UG	HH	8083978	-	6	3236	P	296.998	0.067	0.027
OsJN03357	BGIJ	Chr10_77	EG	HH	505356	-	7	3548	A	0	0	0
OsJN03358	BGIJ	AK105548	CG	LH	8050179	-	2	2669	P	491.093	0.077	0
OsJN03359	BGIJ	AK072615	CG	HH	344808	-	7	6206	P	562.572	0.11	0.032
OsJN03360	BGIJ	Chr10_1416	UG	LH	9119221	-	10	3583	P	311.795	0.104	0
OsJN03361	BGIJ	Chr10_1349	UG	LH	8700975	-	2	828	P	947.592	0.077	0
OsJN03362	BGIJ	Chr10_868	UG	LH	5599762	-	1	402	P	477.896	0.222	0
OsJN03363	BGIJ	Chr10_969	EG	HH	6238988	-	16	7692	P	485.785	0.026	0.06
OsJN03364	BGIJ	Chr10_1362	UG	LH	8797660	-	2	1820	P	415.017	0.3	0.033
OsJN03365	BGIJ	Chr10_260	UG	HH	1644918	-	12	10584	P	848.013	0.099	0.095
OsJN03366	BGIJ	Chr10_87	UG	HH	558727	-	12	6564	P	372.867	0.074	0.048
OsJN03367	BGIJ	Chr10_574	UG	LH	3708379	-	2	782	P	743.663	0.667	0.571
OsJN03368	BGIJ	Chr10_673	UG	LH	4344702	-	7	2098	P	424.974	0.087	0
OsJN03369	BGIJ	Chr10_483	UG	HH	3089630	-	3	1547	P	674.432	0.071	0
OsJN03370	BGIJ	Chr10_912	UG	HH	5894214	-	7	4572	P	349.412	0.05	0
OsJN03371	BGIJ	AK106693	CG	LH	10424294	-	6	3363	A	0	0	0
OsJN03372	BGIJ	Chr10_1313	UG	LH	8480107	-	1	396	P	325.909	0.4	0
OsJN03373	BGIJ	Chr10_492	UG	HH	3161977	-	8	7268	P	409.171	0.048	0.042
OsJN03374	BGIJ	Chr10_1334	UG	HH	8594033	-	3	1583	P	551.117	0.143	0
OsJN03375	BGIJ	Chr10_443	UG	HH	2800491	-	7	3280	P	371.503	0.025	0.1
OsJN03376	BGIJ	AK103313	CG	LH	2321517	-	2	2795	P	488.834	0.059	0.037
OsJN03377	BGIJ	AK111067	CG	LH	2578860	-	2	2199	A	0	0	0
OsJN03378	BGIJ	Chr10_957	UG	HH	6149037	-	4	2174	P	897.578	0.037	0
OsJN03379	BGIJ	AK064175	CG	LH	1626564	-	5	2441	P	637.777	0.156	0
OsJN03380	BGIJ	Chr10_1658	UG	HH	10832227	-	3	2627	P	927.914	0.023	0.071
OsJN03381	BGIJ	Chr10_1167	UG	LH	7584304	-	6	2871	P	450.766	0.053	0.143
OsJN03382	BGIJ	Chr10_839	UG	LH	5396126	-	5	3868	P	339.042	0.091	0.139
OsJN03383	BGII	Chr10_2144	UG	LH	14583084	-	1	513	P	2644.722	0.75	0
OsJN03384	BGII	Chr10_2031	UG	LH	13866439	-	1	441	P	2001.977	0.4	0
OsJN03385	BGII	Chr10_2291	UG	LH	15596769	-	1	420	P	1907.864	0.1	0
OsJN03386	BGII	Chr10_3231	UG	LH	21674848	-	1	327	P	1320.896	0.25	0
OsJN03387	BGII	Chr10_2465	UG	LH	16969849	-	2	741	P	1376.713	0.25	0.25
OsJN03388	BGII	Chr10_1681	UG	LH	11565304	-	1	228	P	5151.415	0.667	0
OsJN03389	BGII	Chr10_1341	UG	LH	9323932	-	2	684	P	3806.866	0.571	0.5
OsJN03390	BGII	Chr10_2928	UG	LH	19789135	-	2	1156	P	2312.105	0.429	0.579
OsJN03391	BGII	Chr10_3026	UG	LH	20376106	-	4	1310	P	1150.756	0.3	0.053
OsJN03392	BGII	Chr10_2127	UG	LH	14494231	-	2	573	P	1882.445	0.417	0
OsJN03393	BGII	Chr10_1093	UG	LH	7541712	-	1	549	P	3067.483	0.385	0
OsJN03394	BGII	Chr10_1039	UG	LH	7198458	-	3	1184	P	3500.421	0.333	0.118
OsJN03395	BGII	Chr10_1206	UG	LH	8315624	-	8	2992	P	1510.651	0.103	0.056
OsJN03396	BGII	Chr10_1448	UG	LH	10049322	-	2	1113	P	1741.599	0.6	0.2
OsJN03397	BGII	Chr10_1572	UG	HH	10850398	-	6	2679	P	1536.909	0.067	0.125
OsJN03398	BGII	Chr10_1179	UG	LH	8066388	-	1	483	P	2237.824	0.3	0
OsJN03399	BGII	Chr10_1260	UG	LH	8692173	-	1	312	P	752.7	0.429	0
OsJN03400	BGII	Chr10_3112	UG	HH	20946678	-	6	5952	P	1033.82	0.072	0.016
OsJN03401	BGII	Chr10_1433	UG	LH	9949626	-	3	1952	P	4219.848	0.333	0.194
OsJN03402	BGII	Chr10_1620	UG	LH	11128215	-	1	570	P	6479.041	0.615	0
OsJN03403	BGII	Chr10_2903	EG	LH	19627896	-	2	1003	P	4611.178	0.471	0.333
OsJN03404	BGII	Chr10_1817	UG	LH	12525912	-	2	1062	P	7602.336	0.4	0.474
OsJN03405	BGII	Chr10_3220	UG	LH	21616324	-	2	888	P	1077.718	0.263	0
OsJN03406	BGII	Chr10_1059	UG	LH	7310142	-	2	389	P	5686.22	0.111	0
OsJN03407	BGII	Chr10_1005	UG	LH	6964428	-	2	459	P	4929.905	0.222	0
OsJN03408	BGII	Chr10_1305	UG	LH	8993251	-	1	390	P	5927.815	1	0
OsJN03409	BGII	Chr10_2332	UG	LH	15842617	-	2	731	P	4331.81	0.6	0.333
OsJN03410	BGII	Chr10_1387	UG	LH	9672320	-	1	225	P	2437.119	0.333	0
OsJN03411	BGII	Chr10_2806	UG	HH	19106182	-	6	3074	P	788.256	0.111	0.111

OsJN03412	BGII	Chr10_1327	UG	LH	9181654	-	2	451	P	3069.987	0.5	0
OsJN03413	BGII	Chr10_1182	UG	LH	8076091	-	1	489	P	5151.847	0.182	0
OsJN03414	BGII	Chr10_3265	UG	LH	21870701	-	3	1041	P	1757.093	0.333	0
OsJN03415	BGII	Chr10_1202	UG	LH	8293703	-	4	1496	P	3029.592	0.545	0.043
OsJN03416	BGII	Chr10_149	UG	LH	1054971	+	3	1186	P	5623.583	0.286	0
OsJN03417	BGII	Chr10_816	UG	LH	5648676	+	5	4871	P	6912.784	0.063	0.071
OsJN03418	BGII	Chr10_565	UG	LH	3878174	+	1	555	P	3128.988	0.308	0
OsJN03419	BGII	Chr10_788	UG	LH	5443297	+	1	600	P	4569.686	0.429	0
OsJN03420	BGII	Chr10_593	UG	LH	4041272	+	5	1745	P	1649.649	0.238	0.235
OsJN03421	BGII	Chr10_148	UG	LH	1051100	+	1	525	P	5513.747	0.333	0
OsJN03422	BGII	Chr10_913	EG	LH	6364621	+	1	252	P	1939.332	0.667	0
OsJN03423	BGII	Chr10_553	UG	LH	3791413	+	1	141	P	6198.228	0.5	0
OsJN03424	BGII	Chr10_499	UG	LH	3407547	+	1	234	P	2231.227	0.5	0
OsJN03425	BGII	Chr10_352	UG	HH	2346311	+	5	4408	P	5479.606	0.125	0.035
OsJN03426	BGII	Chr10_803	UG	LH	5539596	+	3	2311	P	2261.456	0.1	0.025
OsJN03427	BGII	Chr10_844	UG	LH	5851677	+	1	261	P	3631.409	0.833	0
OsJN03428	BGII	Chr10_875	UG	HH	6106378	+	2	1844	P	6594.673	0.067	0.292
OsJN03429	BGII	Chr10_1011	UG	LH	7024784	+	4	2377	P	2088.974	0.235	0.067
OsJN03430	BGII	Chr10_2434	UG	LH	16618387	+	3	2233	P	2046.92	0.222	0.026
OsJN03431	BGII	Chr10_1111	UG	LH	7656199	+	2	773	P	2751.354	0.111	0.375
OsJN03432	BGII	Chr10_1626	UG	HH	11182849	+	3	2019	P	2247.292	0.2	0.133
OsJN03433	BGII	Chr10_1197	UG	LH	8257572	+	1	423	P	2167.895	0.7	0
OsJN03434	BGII	Chr10_1308	UG	HH	9032294	+	3	1762	P	1015.156	0.071	0.091
OsJN03435	BGII	Chr10_2081	UG	LH	14202830	+	4	1508	P	1980.31	0.462	0.3
OsJN03436	BGII	Chr10_1684	UG	LH	11603829	+	2	691	P	4140.001	0.111	0
OsJN03437	BGII	Chr10_3226	EG	LH	21648428	+	2	527	P	9365.482	0.286	0
OsJN03438	BGII	Chr10_1374	UG	LH	9601060	+	3	1377	P	2912.968	0.167	0.227
OsJN03439	BGII	Chr10_1299	UG	HH	8949002	+	2	2951	P	2440.752	0.04	0.042
OsJN03440	BGII	Chr10_1424	UG	LH	9890610	+	2	312	P	5123.314	0.667	0
OsJN03441	BGII	Chr10_2464	UG	LH	16958237	+	3	2738	P	5990.016	0.357	0.152
OsJN03442	BGII	Chr10_1159	UG	LH	7942354	+	2	1677	P	1178.369	0.2	0.04
OsJN03443	BGII	Chr10_2762	UG	LH	18813287	+	1	735	P	1616.539	0.235	0
OsJN03444	BGII	Chr10_1678	UG	LH	11553157	+	2	691	P	6339.178	0.5	0.25
OsJN03445	BGII	Chr10_1839	UG	LH	12674208	+	2	334	P	5185.599	0.333	0
OsJN03446	BGII	Chr10_2417	UG	LH	16501423	+	2	935	P	1037.061	0.167	0.667
OsJN03447	BGII	Chr10_2037	UG	LH	13910004	+	2	1113	P	1617.934	0.2	0
OsJN03448	BGII	Chr10_2631	UG	LH	17943989	+	4	1446	P	2573.411	0.125	0.043
OsJN03449	BGII	Chr10_2142	UG	LH	14566680	+	1	261	P	1390.209	0.143	0
OsJN03450	BGII	Chr10_1955	UG	HH	13483302	+	1	1116	P	757.53	0.12	0
OsJN03451	BGII	Chr10_2173	UG	LH	14828082	+	1	483	P	4778.395	0.455	0
OsJN03452	BGII	Chr10_1400	UG	LH	9751159	+	1	282	P	5420.417	0.429	0
OsJN03453	BGII	Chr10_1259	UG	LH	8689737	+	1	285	P	4799.368	0.333	0
OsJN03454	BGII	Chr10_1933	UG	LH	13367568	+	2	1216	P	2420.591	0.375	0.091
OsJN03455	BGII	Chr10_1343	UG	LH	9334579	+	4	1407	P	2286.901	0.063	0
OsJN03456	BGII	Chr10_1660	UG	LH	11430270	+	2	1049	P	3865.763	0.583	0
OsJN03457	BGII	Chr10_1890	UG	LH	13040037	+	5	1877	P	5740.313	0.5	0.125
OsJN03458	BGII	Chr10_1505	UG	LH	10419706	+	2	500	P	4481.67	0.5	0.286
OsJN03459	BGII	Chr10_1420	UG	LH	9860880	+	1	537	P	885.551	0.231	0
OsJN03460	BGII	Chr10_2163	UG	HH	14740786	+	4	4979	P	2012.546	0.25	0.155
OsJN03461	BGII	Chr10_3164	UG	LH	21235372	+	3	2698	P	1392.201	0.2	0.074
OsJN03462	BGII	Chr10_2807	UG	HH	19113209	+	2	1041	P	908.442	0.267	0.143
OsJN03463	BGII	Chr10_1074	UG	LH	7385155	+	2	534	P	7086.435	0.5	0
OsJN03464	BGII	Chr10_1163	UG	LH	7964009	+	2	1605	P	1534.733	0.1	0.043
OsJN03465	BGII	Chr10_1428	UG	LH	9926620	+	3	1103	P	2249.664	0.286	0.143
OsJN03466	BGII	Chr10_3087	UG	LH	20752899	+	1	684	P	2915.756	0.25	0
OsJN03467	BGII	Chr10_1032	UG	LH	7155106	+	2	614	P	1994.402	0.125	0.5

OsJN03468	BGII	Chr10_322	UG	LH	2186208	-	3	972	P	3734.058	0.333	0.111
OsJN03469	BGII	Chr10_339	UG	LH	2268474	-	2	943	P	4243.058	0.385	0
OsJN03470	BGII	Chr10_238	UG	LH	1646882	-	1	354	P	1711.574	0.375	0
OsJN03471	BGII	Chr10_348	UG	LH	2324215	-	1	474	P	5614.862	0.909	0
OsJN03472	BGII	Chr10_785	UG	LH	5411647	-	2	1662	P	7976.494	0.273	0.04
OsJN03473	BGII	Chr10_824	UG	LH	5707692	-	2	398	P	4473.483	0.25	1
OsJN03474	BGII	Chr10_342	UG	LH	2289798	-	3	1477	P	2520.325	0.375	0.2
OsJN03475	BGII	Chr10_104	UG	LH	774214	-	1	384	P	2189.012	0.444	0
OsJN03476	BGII	Chr10_562	UG	LH	3858615	-	1	477	P	5658.865	0.545	0
OsJN03477	BGII	Chr10_684	UG	LH	4645686	-	2	594	P	1614.465	0.417	0
OsJN03478	BGII	Chr10_123	UG	LH	912059	-	1	576	P	1457.251	0.385	0
OsJN03479	BGII	Chr10_615	UG	LH	4170960	-	3	2134	P	3249.768	0.6	0.27
OsJN03480	BGII	Chr10_169	UG	LH	1215696	-	5	2902	P	3928.135	0.333	0.229
OsJN03481	BGII	Chr10_117	UG	LH	871401	-	1	1188	P	3208.403	0.444	0
OsJN03482	BGII	Chr10_675	UG	LH	4584460	-	1	330	P	6664.774	0.875	0
OsJN03483	BGII	Chr10_464	UG	LH	3122079	-	2	810	P	4323.495	0.5	0.091
OsJN03484	BGII	Chr10_574	UG	LH	3918710	-	1	171	P	4705.434	0.5	0
OsJN03485	BGII	Chr10_219	UG	LH	1498671	-	3	1159	P	2369.487	0.333	0.118
OsJN03486	BGII	Chr02_3836	UG	none	1021371	+	1	381	P	1873.119	0.7	0
OsJN03487	BGII	Chr07_2073	UG	none	7105701	-	3	652	P	610.368	0.222	0
OsJN03488	BGII	Chr01_1857	UG	none	10060883	+	2	732	P	2534.706	0.4	0.091
OsJN03489	BGII	Chr01_5504	UG	none	14527276	-	1	120	P	890.733	0.333	0
OsJN03490	BGII	AK062821	CG	none	22691507	-	1	501	P	1053.93	0.167	0
OsJN03491	BGII	Chr01_3140	UG	none	15070243	+	2	343	P	1773.369	0.4	0
OsJN03492	BGII	Chr05_1451	UG	none	3462750	-	4	5022	P	827.857	0.188	0.098
OsJN03493	BGII	Chr03_4880	UG	none	2499331	-	5	1642	P	698.849	0.333	0.273
OsJN03494	BGII	Chr01_2613	UG	none	22696411	-	1	1087	P	1311.296	0.2	0
OsJN03495	BGII	AK058403	CG	none	13598599	+	6	4958	P	858.434	0.172	0.038
OsJN03496	BGII	Chr05_2505	UG	none	20618140	-	3	1533	P	1214.313	0.129	0
OsJN03497	BGF	Bgf_Chr10_995	UG	LH	6229140	-	3	2230	P	\	\	\
OsJN03498	BGF	Bgf_Chr10_993	UG	LH	6215989	-	2	3966	P	\	\	\
OsJN03499	BGF	Bgf_Chr10_877	UG	LH	5462660	-	1	660	P	\	\	\
OsJN03500	BGF	Bgf_Chr10_834	UG	LH	5132562	+	1	339	P	\	\	\
OsJN03501	BGF	Bgf_Chr10_828	UG	LH	5076711	+	4	1818	P	\	\	\
OsJN03502	BGF	Bgf_Chr10_794	UG	LH	4878773	+	3	2312	P	\	\	\
OsJN03503	BGF	Bgf_Chr10_752	UG	LH	4664267	-	1	708	P	\	\	\
OsJN03504	BGF	Bgf_Chr10_700	UG	LH	4361161	-	2	355	P	\	\	\
OsJN03505	BGF	Bgf_Chr10_668	UG	LH	4145360	+	2	1806	P	\	\	\
OsJN03506	BGF	Bgf_Chr10_641	UG	LH	4004975	-	1	288	P	\	\	\
OsJN03507	BGF	Bgf_Chr10_629	UG	LH	3913624	-	2	1037	P	\	\	\
OsJN03508	BGF	Bgf_Chr10_615	UG	LH	3810280	+	1	468	P	\	\	\
OsJN03509	BGF	Bgf_Chr10_560	UG	LH	3425521	+	2	1416	P	\	\	\
OsJN03510	BGF	Bgf_Chr10_491	UG	LH	3012548	-	3	764	P	\	\	\
OsJN03511	BGF	Bgf_Chr10_48	UG	LH	259058	+	1	396	P	\	\	\
OsJN03512	BGF	Bgf_Chr10_476	UG	LH	2939357	+	3	1657	P	\	\	\
OsJN03513	BGF	Bgf_Chr10_473	UG	LH	2930432	-	1	441	P	\	\	\
OsJN03514	BGF	Bgf_Chr10_418	UG	LH	2586866	-	4	3931	P	\	\	\
OsJN03515	BGF	Bgf_Chr10_414	UG	LH	2565038	+	1	405	P	\	\	\
OsJN03516	BGF	Bgf_Chr10_3530	UG	LH	22376129	-	1	681	P	\	\	\
OsJN03517	BGF	Bgf_Chr10_3494	UG	LH	22101514	+	2	602	P	\	\	\
OsJN03518	BGF	Bgf_Chr10_3348	UG	LH	21182803	-	2	883	P	\	\	\
OsJN03519	BGF	Bgf_Chr10_3239	UG	LH	20496950	-	1	774	P	\	\	\
OsJN03520	BGF	Bgf_Chr10_3227	UG	LH	20447637	-	1	384	P	\	\	\
OsJN03521	BGF	Bgf_Chr10_3089	UG	LH	19614806	-	2	1131	P	\	\	\
OsJN03522	BGF	Bgf_Chr10_3079	UG	LH	19574805	-	2	515	P	\	\	\
OsJN03523	BGF	Bgf_Chr10_3053	UG	LH	19369318	+	2	2905	P	\	\	\

OsJN03524	BGF	Bgf_Chr10_2754	UG	LH	17616800	+	1	597	P	\	\	\
OsJN03525	BGF	Bgf_Chr10_2674	UG	LH	16851321	-	3	1669	P	\	\	\
OsJN03526	BGF	Bgf_Chr10_2515	UG	LH	15970291	+	2	502	P	\	\	\
OsJN03527	BGF	Bgf_Chr10_2494	UG	LH	15800197	-	2	1441	P	\	\	\
OsJN03528	BGF	Bgf_Chr10_2483	UG	LH	15696803	+	1	678	P	\	\	\
OsJN03529	BGF	Bgf_Chr10_2465	UG	LH	15579923	+	2	1993	P	\	\	\
OsJN03530	BGF	Bgf_Chr10_2401	UG	LH	15186074	+	2	532	P	\	\	\
OsJN03531	BGF	Bgf_Chr10_2225	UG	LH	14173962	+	2	2085	P	\	\	\
OsJN03532	BGF	Bgf_Chr10_2165	UG	LH	13795746	-	1	450	P	\	\	\
OsJN03533	BGF	Bgf_Chr10_2133	UG	LH	13526420	+	1	303	P	\	\	\
OsJN03534	BGF	Bgf_Chr10_2117	UG	LH	13400390	+	1	207	P	\	\	\
OsJN03535	BGF	Bgf_Chr10_2089	UG	LH	13125579	+	1	264	P	\	\	\
OsJN03536	BGF	Bgf_Chr10_2027	UG	LH	12810702	-	2	1450	P	\	\	\
OsJN03537	BGF	Bgf_Chr10_1996	UG	LH	12616976	-	1	297	P	\	\	\
OsJN03538	BGF	Bgf_Chr10_175	UG	LH	1103077	-	1	570	P	\	\	\
OsJN03539	BGF	Bgf_Chr10_1688	UG	LH	10610891	+	1	321	P	\	\	\
OsJN03540	BGF	Bgf_Chr10_1606	UG	LH	9994042	-	1	444	P	\	\	\
OsJN03541	BGF	Bgf_Chr10_1478	UG	LH	9133400	+	1	867	P	\	\	\
OsJN03542	BGF	Bgf_Chr10_1433	UG	LH	8890640	-	3	2339	P	\	\	\
OsJN03543	BGF	Bgf_Chr10_1366	UG	LH	8509741	-	1	285	P	\	\	\
OsJN03544	BGF	Bgf_Chr10_1296	UG	LH	8124768	-	2	2475	P	\	\	\
OsJN03545	BGF	Bgf_Chr10_1193	UG	LH	7524927	-	1	237	P	\	\	\
OsJN03546	BGF	Bgf_Chr10_1176	UG	LH	7424183	+	1	303	P	\	\	\
OsJN03547	BGF	Bgf_Chr10_1082	UG	LH	6880992	-	2	675	P	\	\	\
OsJN03548	BGF	Bgf_Chr10_994	UG	LH	6223787	+	1	497	P	\	\	\
OsJN03549	BGF	Bgf_Chr10_778	UG	LH	4795492	+	2	581	P	\	\	\
OsJN03550	BGF	Bgf_Chr10_610	UG	LH	3767479	+	2	709	P	\	\	\
OsJN03551	BGF	Bgf_Chr10_523	UG	LH	3219301	-	2	2326	P	\	\	\
OsJN03552	BGF	Bgf_Chr10_452	UG	LH	2786261	+	2	223	P	\	\	\
OsJN03553	BGF	Bgf_Chr10_405	UG	LH	2527016	-	1	396	P	\	\	\
OsJN03554	BGF	Bgf_Chr10_3420	UG	LH	21647539	+	1	300	P	\	\	\
OsJN03555	BGF	Bgf_Chr10_3155	UG	LH	20045216	-	2	762	P	\	\	\
OsJN03556	BGF	Bgf_Chr10_3017	UG	LH	19163533	-	3	753	P	\	\	\
OsJN03557	BGF	Bgf_Chr10_2865	UG	LH	18217317	+	2	1306	P	\	\	\
OsJN03558	BGF	Bgf_Chr10_2684	UG	LH	16942001	-	3	1703	P	\	\	\
OsJN03559	BGF	Bgf_Chr10_256	UG	LH	1577373	+	2	1298	P	\	\	\
OsJN03560	BGF	Bgf_Chr10_2361	UG	LH	14980969	-	2	449	P	\	\	\
OsJN03561	BGF	Bgf_Chr10_2135	UG	LH	13565980	-	3	545	P	\	\	\
OsJN03562	BGF	Bgf_Chr10_2003	UG	LH	12662145	-	2	609	P	\	\	\
OsJN03563	BGF	Bgf_Chr10_1877	UG	LH	11917458	+	1	408	P	\	\	\
OsJN03564	BGF	Bgf_Chr10_1709	UG	LH	10744128	-	2	2937	P	\	\	\
OsJN03565	BGF	Bgf_Chr10_1610	UG	LH	10022370	+	2	1771	P	\	\	\
OsJN03566	BGF	Bgf_Chr10_15	UG	LH	102279	+	1	411	P	\	\	\
OsJN03567	BGF	Bgf_Chr10_1353	UG	LH	8451626	+	2	831	P	\	\	\
OsJN03568	BGF	Bgf_Chr10_1219	UG	LH	7688963	+	1	138	P	\	\	\

[1] TIGR, TIGR japonica chromosome 10 gene models, BGIJ, BGI japonica chromosome 10 gene models, BGII, BGI indica gene models, BGF, BGF preditions mateched with japonica chromosome 10 intergenic TARs; see main text for detail; [2] see legend of Supplemental Table 1 for the classification of gene models; [3] Homology to Arabidopsis genes; [4] Starting position of the gene models on chromosome 10 of the release 2 of the TIGR Rice Pseudomolecule; [5] P, tiling array detected, A, tiling array undetected; [6] HR, hybridization rate, see main text for detail.
