# Supplementary material for: Tiling microarray analysis of rice chromosome 10 to identify the transcriptome and relate its expression to chromosomal architecture
Source: Genome Biol. 2005 May 27;6(6):R52. doi: 10.1186/gb-2005-6-6-r52 (PMC1175972; doi:10.1186/gb-2005-6-6-r52)
Supplement: Additional File 2 — Table S2: Indica chromosome 10 nonredundant gene models. Indica chromosome 10 nonredundant gene models. [file gb-2005-6-6-r52-S2.pdf]

Supplemental Table 2. *Indica* chromosome 10 non-redundant gene models

GeneName	Type [1]	Homology [2]	Position [3]	Strand	Length	Exon #	Detection [4]	Intensity	ExonHR [5]	IntronHR [5]
AK102556	CG	HH	4405	+	5866	8	P	1888.896	0.063	0.016
Chr10_2	UG	LH	10557	-	1792	3	P	5105.6	0.545	0.08
Chr10_5	UG	HH	43475	+	3034	6	P	2951.916	0.027	0.077
Chr10_6	UG	LH	51651	+	291	1	P	4509.645	0.167	0
AK064524	CG	LH	52722	-	887	3	A	1184.463	0.053	0
Chr10_7	UG	LH	60240	+	1237	3	A	535.041	0.1	0.111
Chr10_8	UG	LH	63312	-	2014	5	P	1812.91	0.087	0.056
Chr10_9	UG	LH	68606	+	6602	12	A	527.564	0.028	0.039
Chr10_10	UG	LH	76846	+	1329	4	P	3226.017	0.111	0.083
AK066584	CG	LH	80820	-	1488	2	P	4013.32	0.353	0
Chr10_12	UG	LH	85741	+	1745	6	A	0	0	0
Chr10_13	UG	HH	89291	-	2241	2	A	1484.874	0.059	0.094
AK060176	CG	LH	122235	-	1069	2	P	4347.017	0.318	1
AK106544	CG	HH	139432	-	7219	11	P	3726.53	0.302	0.14
Chr10_19	UG	LH	165185	-	165	1	P	4474.735	0.75	0
Chr10_23	UG	HH	196873	+	5471	9	P	1725.182	0.111	0.075
AK101265	CG	HH	203689	-	6790	9	P	3020.895	0.167	0.104
AK072615	CG	HH	215412	-	6196	7	P	2117.548	0.315	0.164
AK103627	CG	HH	259143	-	9306	8	P	2241.504	0.318	0.132
Chr10_30	UG	HH	278167	+	2971	5	P	6593.383	0.029	0.065
Chr10_31	UG	HH	282714	-	1517	2	P	1935.893	0.281	1
Chr10_32	UG	HH	287221	-	1946	2	P	2664.893	0.387	0
Chr10_33	UG	LH	296852	-	1040	2	P	2111.105	0.429	0.313
Chr10_34	UG	HH	301773	+	872	2	P	1772.789	0.077	0
Chr10_35	UG	LH	305054	-	1039	3	A	1314.089	0.059	0.167
Chr10_36	UG	HH	313069	+	1591	2	P	5023.109	0.136	0
Chr10_37	UG	HH	317680	+	1368	1	P	3877.873	0.2	0
Chr10_38	UG	LH	320422	+	1776	4	P	2451.054	0.25	0.056
Chr10_39	UG	LH	322904	+	495	2	A	913.042	0.167	0.167
AK105379	CG	LH	324585	+	423	2	P	2691.581	0.455	0
Chr10_40	UG	HH	333099	+	3889	2	P	3143.226	0.162	0.021
Chr10_41	UG	LH	340175	-	3163	6	P	2270.016	0.174	0.063
Chr10_42	UG	HH	347237	-	993	1	P	2249.458	0.409	0
Chr10_44	UG	HH	361249	-	4993	8	P	1707.506	0.167	0.023
Chr10_46	UG	LH	395822	+	441	2	A	0	0	0.143
Chr10_47	UG	HH	396836	-	2930	11	P	1538.503	0.167	0.19
Chr10_48	UG	HH	401137	+	1185	1	P	1480.657	0.192	0
Chr10_49	UG	LH	403329	+	404	3	P	3216.016	0.333	0
AK103056	CG	LH	404445	+	4163	7	A	715.426	0.011	0
Chr10_52	UG	HH	424438	-	4972	9	P	1579.98	0.214	0.17
AK060060	CG	HH	431273	+	1623	2	P	3169.732	0.059	0.5
Chr10_55	UG	HH	435230	+	729	1	P	2357.848	0.294	0
Chr10_56	UG	HH	444239	+	1344	1	P	3249.333	0.067	0
Chr10_59	UG	LH	454069	-	2995	4	P	1681.965	0.186	0.273
Chr10_61	UG	HH	472845	+	2458	4	P	5070.152	0.029	0
Chr10_63	EG	HH	491977	-	2817	3	P	1755.005	0.304	0.086
Chr10_66	UG	HH	516010	-	2375	5	P	2532.057	0.31	0.182
AK099448	CG	HH	527646	-	1704	4	P	1658.732	0.393	0
Chr10_68	UG	HH	530568	-	7278	9	P	3542.424	0.254	0.082
Chr10_69	UG	HH	539936	-	2374	5	P	3007.166	0.286	0.182
Chr10_70	UG	LH	545610	-	287	2	A	0	0	0.25
Chr10_74	UG	LH	562569	-	1259	4	P	1737.703	0.278	0.5
Chr10_76	UG	LH	586534	+	2928	3	P	3760.481	0.429	0.047
Chr10_77	UG	LH	594858	-	542	2	P	4307.591	0.375	0.25
Chr10_79	UG	HH	615887	-	951	3	P	2886.29	0.188	0.167
Chr10_80	UG	LH	619245	-	1168	3	A	642.15	0.083	0.3
Chr10_81	UG	HH	627326	-	3695	6	P	4170.763	0.279	0
Chr10_82	EG	HH	641289	+	2446	3	P	2872.082	0.063	0.1

Chr10_83	UG	LH	645045	-	3185	4	P	2336.501	0.158	0.064
AK070621	CG	HH	659728	-	4654	6	P	3066.187	0.288	0.087
AK068679	CG	HH	669341	-	3236	7	P	4084.982	0.34	0.087
AK066625	CG	LH	671825	+	971	2	P	6493.604	0.136	0
Chr10_87	UG	HH	681221	-	420	3	P	2187.099	0.429	0
Chr10_88	UG	HH	683083	-	5185	4	P	1832.684	0.208	0.082
Chr10_90	UG	HH	695981	+	4370	3	P	1737.123	0.059	0.023
Chr10_91	UG	LH	702074	-	189	1	P	2654.967	0.6	0
Chr10_92	UG	LH	703207	+	1435	4	P	1336.914	0.125	0
Chr10_93	UG	LH	705469	-	378	1	P	5915.223	0.875	0
AK065457	CG	HH	718635	-	3055	14	P	3222.026	0.2	0.375
Chr10_97	UG	LH	740011	-	3461	3	P	4456.387	0.118	0.185
Chr10_98	UG	LH	747514	-	1003	3	A	898.897	0.091	0
Chr10_99	UG	LH	754923	+	455	2	P	4091.298	0.25	0.333
Chr10_100	UG	HH	757335	-	838	3	P	4313.045	0.538	0.167
Chr10_101	UG	HH	760297	+	1822	5	P	3334.177	0.139	0
Chr10_102	UG	HH	765315	+	2028	2	P	1542.458	0.184	0
AK099727	CG	HH	768332	-	3736	11	P	3417.837	0.304	0
Chr10_104	UG	LH	774214	-	384	1	P	2189.012	0.444	0
AK105108	CG	HH	776800	-	2040	2	P	1767.07	0.149	0
AK102609	CG	HH	796484	+	18261	6	P	2793.436	0.167	0.062
Chr10_107	UG	HH	804139	+	966	3	P	5870.33	0.267	0
AK103772	CG	HH	815791	+	1457	2	P	4531.52	0.185	0
Chr10_110	UG	LH	827658	-	219	1	A	1322.397	0.833	0
Chr10_114	UG	LH	848665	+	263	2	A	0	0	0
Chr10_115	UG	LH	853666	+	5429	8	P	3826.984	0.286	0.053
AK107387	CG	HH	862762	+	1488	5	P	2122.913	0.063	0
Chr10_117	UG	LH	871401	-	1188	1	P	3208.403	0.444	0
AK063329	CG	HH	875016	+	2544	4	P	7392.187	0.067	0.091
Chr10_119	UG	HH	877782	-	3333	1	P	4391.931	0.205	0
Chr10_120	EG	HH	882009	+	3564	6	P	8536.985	0.15	0.132
Chr10_121	EG	HH	892709	-	1090	2	P	2430.02	0.375	0
Chr10_123	UG	LH	912059	-	576	1	P	1457.251	0.385	0
AK063643	CG	HH	915270	-	1774	9	P	2625.564	0.524	0
Chr10_125	UG	LH	926093	+	1251	3	P	5838.964	0.556	0.105
Chr10_126	UG	HH	929173	-	1431	2	P	4373.421	0.3	0
Chr10_127	UG	LH	932613	+	500	3	P	4746.973	0.6	0
Chr10_128	UG	LH	933755	-	1853	2	P	9253.664	0.571	0.265
AK100686	CG	LH	940011	-	5289	12	P	3082.894	0.386	0.03
Chr10_130	UG	LH	951973	+	2346	7	A	714.668	0.043	0
Chr10_131	UG	LH	955787	-	210	1	P	1642.9	0.4	0
Chr10_132	UG	HH	966515	-	3291	6	P	1777.905	0.179	0.097
Chr10_134	UG	LH	978377	+	865	3	P	5778.183	0.308	0
Chr10_135	EG	LH	980305	-	6552	11	P	2462.113	0.282	0.197
Chr10_136	UG	HH	989831	-	3211	6	P	1978.889	0.246	0.1
Chr10_137	UG	LH	995718	-	1531	2	P	5745.656	0.5	0.174
Chr10_138	UG	LH	998004	+	4153	7	P	2909.2	0.097	0
Chr10_142	UG	LH	1015754	+	1213	3	A	1019.924	0.059	0.1
Chr10_143	UG	LH	1020108	+	1017	3	A	0	0	0
AK110804	CG	HH	1022335	+	2026	7	P	4993.618	0.067	0
AK060266	CG	HH	1024599	-	3414	9	A	933.104	0.109	0.161
Chr10_146	EG	HH	1032689	-	3569	4	P	4064.302	0.174	0.073
Chr10_147	UG	LH	1048686	+	853	2	P	3928.905	0.462	0.571
Chr10_148	UG	LH	1051100	+	525	1	P	5513.747	0.333	0
Chr10_149	UG	LH	1054971	+	1186	3	P	5623.583	0.286	0
Chr10_150	UG	LH	1058356	+	1443	3	P	3359.407	0.316	0
AK108670	CG	HH	1066862	+	3654	6	P	2501.538	0.073	0
Chr10_152	UG	HH	1071239	+	2873	3	P	1426.841	0.158	0
Chr10_156	UG	LH	1094477	-	261	1	A	1456.488	0.286	0
AK069721	CG	HH	1097696	-	1489	4	P	2647.682	0.3	0.333
AK069308	CG	HH	1110931	-	1587	2	P	1977.766	0.412	0
Chr10_159	UG	LH	1130320	-	383	2	A	0	0	0

Chr10_160	UG	LH	1132087	-	1860	4	P	3296.307	0.235	0.042
AK058386	CG	HH	1135507	-	1447	4	P	2411.796	0.233	0.667
AK108086	CG	LH	1160071	+	600	1	P	1171.666	0.071	0
Chr10_162	UG	HH	1162051	-	3370	2	P	1214.607	0.137	0
AK063350	CG	HH	1172113	+	7644	15	P	1616.256	0.042	0.018
Chr10_164	UG	HH	1181196	-	4966	7	A	863.146	0.061	0.074
Chr10_165	UG	LH	1191113	+	426	1	P	4269.556	0.4	0
Chr10_166	UG	LH	1192775	+	642	1	P	2451.909	0.667	0
Chr10_167	EG	HH	1201873	+	2309	3	P	1636.418	0.106	0
Chr10_168	UG	LH	1207223	+	416	2	P	3365.066	0.143	0
Chr10_169	UG	LH	1215696	-	2902	5	P	3928.135	0.333	0.229
Chr10_170	UG	LH	1222407	+	519	2	P	2805.921	0.333	0
Chr10_171	UG	HH	1225838	+	1794	2	A	0	0	0.154
Chr10_172	UG	LH	1229466	-	258	1	A	720.793	0.143	0
Chr10_174	UG	HH	1232944	-	8601	9	P	2233.053	0.179	0.087
Chr10_175	UG	LH	1244421	+	888	2	P	4665.713	0.438	1
Chr10_177	UG	LH	1250332	+	838	2	P	3055.987	0.2	0.333
Chr10_178	UG	LH	1252882	-	1298	2	P	4426.054	0.222	0.167
Chr10_179	UG	LH	1257674	-	436	2	A	579.054	0.143	0.25
Chr10_182	EG	HH	1270578	+	1479	1	P	2528.414	0.061	0
Chr10_184	UG	LH	1296002	-	835	2	P	2449.517	0.167	0.167
Chr10_187	UG	LH	1308425	-	1127	2	P	7695.505	0.4	0.222
Chr10_188	UG	HH	1314860	+	1256	2	P	2061.199	0.25	0
Chr10_189	UG	HH	1320068	+	4604	5	P	3529.438	0.128	0.021
Chr10_190	EG	HH	1336005	+	8296	5	P	2052.848	0.059	0.069
Chr10_191	UG	HH	1347799	-	918	1	A	1297.195	0.19	0
Chr10_192	UG	LH	1351615	-	542	2	P	2389.782	0.308	0
Chr10_193	UG	HH	1355041	-	1014	1	P	2273.461	0.217	0
Chr10_194	UG	LH	1359504	-	550	2	A	0	0	0.286
Chr10_195	UG	LH	1366824	-	600	1	P	4781.36	0.571	0
Chr10_196	UG	HH	1371667	+	1479	3	P	3247.854	0.182	0
Chr10_197	UG	HH	1379419	+	1308	2	P	3248.505	0.154	0.071
Chr10_198	UG	LH	1390125	+	2062	3	P	2862.634	0.267	0.133
Chr10_199	UG	LH	1398324	-	168	1	A	0	0	0
Chr10_200	UG	LH	1406358	-	343	2	P	6299.149	0.667	0.5
Chr10_201	UG	LH	1407657	+	1158	2	A	611.461	0.083	0.214
Chr10_202	UG	HH	1411318	+	2927	4	P	3452.607	0.143	0
Chr10_204	UG	HH	1420974	+	2725	3	P	1761.354	0.114	0.154
Chr10_205	UG	HH	1432474	-	1906	2	P	2323.367	0.5	0
Chr10_206	EG	LH	1438161	+	3050	3	P	2481.915	0.139	0.065
Chr10_207	EG	LH	1444350	+	1509	3	P	1437.011	0.182	0
Chr10_208	UG	HH	1448850	-	845	2	P	1809.593	0.176	0
Chr10_209	UG	LH	1451931	-	1626	2	P	2654.234	0.292	0
Chr10_210	EG	HH	1457962	+	1376	4	A	0	0	0.19
Chr10_211	UG	LH	1460421	+	3135	4	P	1838.861	0.073	0.036
Chr10_212	UG	HH	1466315	+	906	1	P	4903.382	0.15	0
Chr10_213	UG	HH	1467665	-	4901	4	P	1533.908	0.235	0.032
Chr10_214	UG	HH	1477272	-	4494	5	P	1673.006	0.164	0.12
Chr10_215	UG	LH	1482957	-	201	1	P	5380.168	0.8	0
Chr10_216	EG	LH	1485752	+	534	1	P	4638.655	0.077	0
Chr10_217	UG	LH	1490483	+	450	1	P	1464.052	0.2	0
AK064648	CG	LH	1491368	-	2733	6	A	1136.827	0.098	0.3
Chr10_219	UG	LH	1498671	-	1159	3	P	2369.487	0.333	0.118
Chr10_221	UG	LH	1518876	-	1150	2	P	5410.168	0.647	0.333
Chr10_223	UG	LH	1526027	-	657	1	P	5305.625	0.867	0
Chr10_225	UG	LH	1538768	-	2739	4	P	4097.489	0.438	0.333
AK060187	CG	HH	1556365	-	2540	8	P	1802.903	0.175	0
Chr10_227	UG	HH	1564629	+	3632	4	P	3911.704	0.261	0.041
Chr10_228	EG	LH	1591456	-	9171	11	P	2039.19	0.216	0.084
Chr10_229	UG	HH	1601631	-	2967	4	P	1808.353	0.296	0.111
Chr10_230	UG	HH	1605097	+	2348	3	P	1945.68	0.156	0.2
Chr10_231	UG	HH	1609913	-	5879	8	P	2112.475	0.196	0.169

Chr10_232	UG	LH	1616492	+	2115	3	P	6812.72	0.231	0.059
Chr10_233	UG	LH	1621483	+	462	1	P	2350.435	0.2	0
AK064296	CG	HH	1624548	-	3119	8	P	2716.972	0.283	0.16
AK102940	CG	HH	1632000	-	2890	4	P	2833.21	0.214	0.091
Chr10_236	UG	HH	1638852	-	1400	5	P	2245.73	0.19	0.111
AK101761	CG	HH	1643321	-	2922	6	P	5152.855	0.178	0.143
Chr10_238	UG	LH	1646882	-	354	1	P	1711.574	0.375	0
AK106453	CG	HH	1648705	-	2649	5	P	1779.5	0.225	0.158
Chr10_240	UG	LH	1652334	+	821	3	P	2183.576	0.2	0.333
AK063758	CG	LH	1658429	-	1891	3	A	1170.738	0.056	0.087
Chr10_242	UG	LH	1662814	+	192	1	A	0	0	0
Chr10_243	UG	LH	1664341	-	453	1	P	4432.688	0.6	0
Chr10_244	UG	LH	1673078	-	825	1	P	5093.048	0.471	0
AK072882	CG	HH	1676328	-	10775	11	P	2924.024	0.318	0.096
Chr10_246	UG	LH	1689069	-	1630	2	P	4951.196	0.636	0.083
AK101231	CG	HH	1691725	-	3382	4	P	1670.945	0.238	0.065
AK063510	CG	LH	1697328	-	1916	2	P	2735.331	0.205	0
AK063461	CG	LH	1699290	-	992	2	P	4354.442	0.217	0
AK111012	CG	LH	1700480	+	474	1	P	2904.84	0.455	0
AK110742	CG	HH	1703555	-	2608	4	P	1915.678	0.2	0.136
Chr10_250	UG	HH	1710724	+	879	1	A	721.31	0.05	0
Chr10_251	UG	LH	1714315	+	1028	3	P	2942.271	0.2	0
Chr10_252	EG	HH	1716842	-	2033	3	P	2150.355	0.233	0
Chr10_253	EG	HH	1720167	+	654	1	P	1410.604	0.133	0
Chr10_254	EG	LH	1722254	-	1123	3	A	1304.543	0.182	0.1
Chr10_255	UG	HH	1723721	-	4117	5	P	2377.344	0.158	0.062
Chr10_256	UG	HH	1729520	-	1534	3	P	4158.45	0.095	0
Chr10_257	UG	HH	1735741	+	1059	2	P	1384.714	0.048	0
Chr10_259	UG	LH	1752718	-	438	1	P	2119.291	0.273	0
Chr10_260	UG	LH	1758167	-	369	2	P	2094.008	0.8	0
Chr10_261	UG	HH	1762859	+	1236	3	A	999.929	0.043	0.25
Chr10_262	UG	HH	1772578	-	1581	1	P	2161.424	0.286	0
Chr10_263	UG	HH	1776985	-	3831	2	P	2649.891	0.236	0.364
Chr10_264	EG	LH	1784063	+	1074	1	P	1271.265	0.087	0
Chr10_265	UG	LH	1787028	+	1095	2	P	4015.531	0.174	0
Chr10_268	UG	HH	1804770	+	2830	4	A	0	0	0.077
Chr10_269	UG	LH	1812696	+	1855	4	P	2557.94	0.034	0.182
Chr10_271	UG	HH	1824843	-	5566	7	P	2452.67	0.157	0.2
Chr10_272	UG	HH	1834853	-	2429	3	P	4345.465	0.19	0.333
Chr10_273	UG	HH	1842694	-	3209	3	P	1631.857	0.183	0
Chr10_274	UG	HH	1849083	+	1894	4	A	0	0	0
Chr10_275	EG	HH	1858807	-	5043	4	P	2028.987	0.162	0.162
Chr10_276	UG	HH	1868918	+	4539	3	P	2440.201	0.054	0.049
AK107010	CG	LH	1882065	+	1782	5	P	4067.968	0.05	0
Chr10_278	UG	LH	1898525	-	2855	2	P	9261.353	0.333	0.045
Chr10_279	UG	HH	1903080	+	4187	2	P	2752.628	0.281	0
Chr10_280	UG	HH	1912635	+	3061	3	P	3892.515	0.407	0.025
Chr10_281	UG	HH	1916469	+	873	1	P	2346.915	0.368	0
Chr10_282	EG	LH	1921969	-	1434	1	P	2006.078	0.094	0
Chr10_284	UG	LH	1931852	-	2151	2	P	2010.494	0.556	0.129
Chr10_285	UG	LH	1939965	-	1429	3	A	1293.126	0.111	0.25
Chr10_286	UG	LH	1951001	+	1431	1	A	1010.816	0.063	0
Chr10_287	UG	LH	1957835	+	2827	2	A	0	0	0
Chr10_288	UG	LH	1962156	-	336	2	P	3988.939	0.4	0.333
Chr10_289	UG	LH	1969803	-	2490	5	P	2257.5	0.24	0.071
Chr10_290	UG	LH	1976369	+	1446	1	P	2800.592	0.063	0
Chr10_291	EG	LH	1979436	+	1446	1	A	0	0	0
Chr10_292	UG	LH	1981146	-	1595	7	P	3472.448	0.409	0.429
Chr10_293	UG	LH	1983726	+	1452	1	P	1412.528	0.061	0
Chr10_294	UG	LH	1989483	+	1446	1	P	2307.171	0.031	0
Chr10_295	UG	LH	1994593	+	1446	1	P	2861.059	0.063	0
Chr10_296	UG	LH	2000697	+	1262	2	P	1688.208	0.036	0

AK068217	CG	HH	2004997	-	4264	8	P	2966.622	0.366	0.075
AK072221	CG	LH	2011471	+	3599	7	P	4383.072	0.184	0
AK069878	CG	LH	2017908	+	2381	2	P	3229.972	0.194	0
AK099992	CG	LH	2022763	+	5482	6	P	4199.684	0.094	0.05
AK066939	CG	HH	2024519	+	11426	13	P	1625.53	0.073	0.059
Chr10_302	UG	LH	2040255	-	1298	2	P	1574.293	0.179	1
Chr10_303	EG	LH	2043542	-	1589	2	P	3079.854	0.357	0
Chr10_304	UG	HH	2047475	-	2885	7	P	1561.584	0.255	0.143
Chr10_305	UG	LH	2051502	+	2720	3	P	2290.413	0.25	0.068
Chr10_306	UG	HH	2054898	+	4923	7	P	2152.612	0.061	0.034
Chr10_307	UG	LH	2063571	-	1258	2	P	5492.174	0.269	0
Chr10_308	UG	LH	2070958	-	3891	3	P	2479.871	0.31	0.161
Chr10_309	UG	HH	2078480	+	885	1	P	2137.643	0.15	0
Chr10_310	UG	LH	2082117	-	5671	3	P	3331.248	0.353	0.112
Chr10_311	UG	LH	2091868	+	667	3	A	0	0	0
Chr10_312	UG	LH	2094186	+	964	3	P	5268.445	0.267	0
AK103313	CG	LH	2139095	-	2783	3	P	3959.567	0.25	0.038
Chr10_317	UG	LH	2149322	-	3367	3	P	2729.344	0.351	0.135
AK103597	CG	HH	2164553	+	4089	8	P	3015.997	0.183	0
Chr10_319	UG	LH	2172402	-	795	2	A	558.991	0.125	0.167
Chr10_320	EG	LH	2174889	+	2893	4	P	2442.499	0.1	0
AK107242	CG	LH	2181389	+	1636	1	P	2018.792	0.028	0
Chr10_322	UG	LH	2186208	-	972	3	P	3734.058	0.333	0.111
Chr10_323	UG	LH	2198602	-	2697	4	A	827.957	0.125	0.24
Chr10_324	UG	LH	2202343	+	169	2	A	930.17	0.2	0
Chr10_325	UG	LH	2204504	-	1674	3	A	1081.57	0.077	0.083
Chr10_326	UG	LH	2208646	-	716	3	P	3214.904	0.308	0
Chr10_327	UG	LH	2209815	-	444	1	P	2519.722	0.2	0
Chr10_328	UG	LH	2213023	-	400	2	P	3049.13	0.4	0
Chr10_329	UG	LH	2214210	+	1132	2	P	1285.999	0.143	0.053
AK107379	CG	LH	2219015	-	4416	3	P	2334.207	0.297	0.018
AK066925	CG	HH	2230205	+	1926	10	P	1209.309	0.14	0
AK098858	CG	HH	2235588	+	3058	8	P	3513.893	0.231	0.036
AK060173	CG	HH	2241397	+	3248	7	P	1121.872	0.071	0.023
Chr10_335	UG	HH	2245391	-	4329	13	P	4093.696	0.25	0.19
Chr10_336	UG	LH	2252392	-	856	2	P	4244.014	0.462	0.571
Chr10_337	EG	HH	2254902	-	1179	4	A	696.706	0.222	0
Chr10_338	UG	LH	2261423	+	2070	3	A	0	0	0.056
Chr10_339	UG	LH	2268474	-	943	2	P	4243.058	0.385	0
AK063615	CG	LH	2269606	+	1377	2	P	1366.422	0.111	0
AK069016	CG	LH	2276460	+	2322	4	P	2226.529	0.147	0
AK067458	CG	HH	2285449	-	1789	1	P	3543.43	0.41	0
Chr10_342	UG	LH	2289798	-	1477	3	P	2520.325	0.375	0.2
Chr10_344	UG	LH	2303164	-	875	2	P	3470.364	0.579	1
AK069822	CG	HH	2308069	+	12525	29	P	1735.616	0.143	0.07
AK100609	CG	HH	2323405	+	4179	3	P	1402.706	0.069	0.15
Chr10_348	UG	LH	2324215	-	474	1	P	5614.862	0.909	0
Chr10_350	EG	LH	2335297	+	576	1	P	2610.011	0.385	0
Chr10_351	UG	HH	2342303	+	3509	3	P	2461.261	0.191	0.125
Chr10_352	UG	HH	2346311	+	4408	5	P	5479.606	0.125	0.035
AK063564	CG	LH	2355274	+	2126	2	P	3675.307	0.114	0
AK069162	CG	LH	2359883	+	3450	4	P	4226.878	0.12	0.02
Chr10_354	EG	LH	2365904	+	3705	9	P	2225.136	0.107	0.019
Chr10_355	UG	LH	2370373	-	1334	2	P	5090.577	0.538	0.267
AK071673	CG	HH	2373462	+	4537	7	P	1415.134	0.1	0.059
AK072788	CG	LH	2389217	+	1293	1	P	3193.327	0.034	0
Chr10_358	EG	HH	2395752	+	1950	1	A	0	0	0
AK111067	CG	LH	2396118	-	2187	5	A	882.137	0.143	0.033
Chr10_360	UG	HH	2405884	+	4695	2	P	2659.198	0.041	0.113
Chr10_361	UG	HH	2420707	-	5967	9	P	2216.574	0.261	0.175
Chr10_363	UG	LH	2431107	+	1100	2	P	4276.876	0.8	0.091
Chr10_364	UG	HH	2433897	+	660	1	A	1072.893	0.125	0

Chr10_365	UG	HH	2438422	-	617	2	P	2091.04	0.364	0
Chr10_366	UG	LH	2442178	-	1032	2	P	4264.486	0.476	1
Chr10_368	EG	HH	2460207	-	2752	2	P	2590.983	0.229	0.04
Chr10_369	UG	HH	2464425	-	4361	2	P	3566.174	0.182	0.167
Chr10_370	UG	LH	2469708	-	4130	5	P	2222.919	0.333	0.1
Chr10_371	UG	LH	2475249	+	384	1	P	5588.717	0.778	0
AK110870	CG	LH	2487446	-	2572	3	P	4882.217	0.263	0
Chr10_373	UG	LH	2496186	-	1192	3	P	2137.318	0.4	0.2
AK103298	CG	LH	2498967	-	5043	3	P	1527.854	0.257	0.053
Chr10_375	UG	LH	2507927	+	616	2	P	4070.935	0.444	0.6
Chr10_376	UG	LH	2517864	-	1757	5	P	4192.353	0.381	0.059
Chr10_377	UG	LH	2521454	-	3573	4	P	2995.905	0.2	0.161
Chr10_382	UG	LH	2548902	+	525	2	P	2861.4	0.417	0
Chr10_383	UG	HH	2554980	-	11683	9	P	2987.957	0.157	0.181
Chr10_384	UG	LH	2572755	-	817	2	P	2409.135	0.083	0.4
Chr10_385	UG	LH	2579772	+	3579	6	A	0	0	0
Chr10_386	UG	HH	2583541	-	937	2	P	3887.14	0.176	1
AK111104	CG	LH	2592288	+	1222	2	P	1147.006	0.267	0.167
Chr10_388	UG	HH	2599710	+	9717	13	P	3459.081	0.196	0.072
Chr10_390	UG	LH	2620227	-	1456	3	P	2360.329	0.556	0.227
Chr10_391	UG	HH	2624841	+	4750	2	P	1087.135	0.086	0.029
AK059657	CG	LH	2646442	+	1851	4	P	9756.204	0.179	0.071
AK102295	CG	HH	2652657	-	6214	7	P	4407.791	0.404	0.091
AK105620	CG	HH	2675301	-	4151	8	P	3257.043	0.348	0.036
Chr10_396	EG	HH	2675521	+	531	2	A	785.79	0.1	0
AK099480	CG	HH	2685650	-	1372	7	P	2336.51	0.379	0
AK071590	CG	LH	2697733	-	2035	9	P	1477.58	0.212	0
Chr10_401	EG	HH	2701488	-	8035	8	P	2520.841	0.2	0.085
Chr10_402	UG	LH	2711825	+	378	1	P	4398.137	0.222	0
Chr10_405	UG	LH	2732279	-	738	2	P	3254.85	0.4	0
Chr10_406	EG	HH	2736984	+	750	2	P	5596.411	0.188	0
Chr10_407	UG	LH	2742268	+	1653	5	P	3490.934	0.167	0.056
Chr10_408	EG	LH	2747018	-	1491	2	P	3177.193	0.286	0.263
Chr10_409	UG	LH	2750946	-	579	2	A	933.752	0.125	0.167
Chr10_411	UG	LH	2766470	-	3147	4	P	1796.19	0.258	0
AK061529	CG	LH	2772374	-	1090	2	P	3307.451	0.5	0
AK072923	CG	LH	2776468	+	1148	4	P	2046.572	0.16	0
Chr10_414	EG	LH	2780305	+	1043	2	P	1745.945	0.118	0
AK061299	CG	LH	2790107	+	1044	5	P	2616.496	0.217	0
AK100747	CG	LH	2793644	+	2320	11	P	3799.281	0.057	0
Chr10_417	UG	LH	2798192	-	177	1	P	6860.373	0.6	0
Chr10_418	UG	HH	2811179	-	4557	7	P	4801.777	0.179	0.087
Chr10_424	UG	LH	2843572	-	692	3	P	3316.929	0.222	0.143
Chr10_425	UG	HH	2850706	+	4608	3	A	484.105	0.021	0
Chr10_426	UG	LH	2859850	-	1132	4	P	7610.596	0.286	0.176
Chr10_427	UG	HH	2862526	+	1066	3	A	0	0	0
Chr10_429	UG	HH	2871197	+	3666	4	P	1696.755	0.087	0.075
Chr10_430	UG	LH	2876248	+	2579	4	P	1353.472	0.154	0
Chr10_431	UG	LH	2882304	-	2284	2	P	5628.152	0.75	0.2
Chr10_433	UG	LH	2902172	-	192	1	P	7187.552	0.4	0
Chr10_434	UG	LH	2903961	-	1911	2	P	1942.862	0.176	0
Chr10_435	UG	LH	2908408	-	846	3	A	930.189	0.143	0.083
Chr10_436	UG	HH	2913198	+	807	1	A	0	0	0
Chr10_437	UG	HH	2917725	+	2871	4	A	606.638	0.032	0.065
Chr10_438	UG	LH	2924518	-	581	2	P	7836.317	1	0.222
AK066347	CG	HH	2927084	-	5006	15	P	2580.199	0.344	0.2
Chr10_439	EG	LH	2927416	+	308	2	P	2875.933	0.125	0
Chr10_441	UG	HH	2936456	-	5290	4	P	1913.196	0.2	0.062
Chr10_443	UG	HH	2959623	-	3923	9	P	2456.936	0.222	0.125
Chr10_445	UG	LH	2975286	+	987	1	A	750.542	0.045	0
Chr10_448	UG	LH	2996263	-	510	1	P	2349.926	0.417	0
Chr10_449	EG	HH	2997410	+	981	1	P	1967.059	0.136	0

Chr10_450	UG	LH	3006071	+	2203	3	A	0	0	0.034
Chr10_451	UG	HH	3009594	+	978	1	P	2297.342	0.136	0
AK100212	CG	HH	3014894	-	2487	5	P	1943.123	0.351	0.111
AK069896	CG	LH	3018871	+	896	2	P	3028.411	0.1	0
Chr10_453	UG	HH	3022420	-	978	1	P	3308.101	0.227	0
AK064417	CG	HH	3066134	+	4190	6	P	1741.714	0.103	0.029
Chr10_456	UG	LH	3072185	-	255	1	A	0	0	0
Chr10_457	EG	HH	3076229	+	546	1	P	1180.031	0.308	0
Chr10_458	UG	HH	3087520	+	1450	4	P	6214.101	0.188	0.1
AK108770	CG	LH	3091410	+	1436	1	P	3497.301	0.25	0
Chr10_460	EG	LH	3093136	-	354	1	P	7718.777	0.5	0
AK070930	CG	LH	3096592	+	1307	1	P	3326.466	0.207	0
Chr10_462	UG	HH	3101326	+	3056	7	P	2730.753	0.05	0
Chr10_463	EG	HH	3104967	-	1936	5	P	1467.706	0.308	0.125
Chr10_464	UG	LH	3122079	-	810	2	P	4323.495	0.5	0.091
Chr10_465	UG	HH	3128156	+	3554	2	P	3403.495	0.117	0
Chr10_466	UG	LH	3147198	+	1963	4	P	2820.575	0.154	0.1
Chr10_467	UG	HH	3151064	-	984	1	P	1422.103	0.364	0
Chr10_468	EG	HH	3152757	+	2829	2	A	712.567	0.033	0
Chr10_469	UG	HH	3163007	-	975	1	P	5407.64	0.136	0
Chr10_470	UG	HH	3164361	+	984	1	P	1885.315	0.045	0
Chr10_471	UG	LH	3168723	-	1581	2	A	1402.708	0.2	0.1
Chr10_472	UG	HH	3175071	+	978	1	P	5465.166	0.045	0
Chr10_473	UG	HH	3185477	+	2208	1	P	1915.256	0.146	0
AK111040	CG	LH	3192090	+	455	1	P	5650.512	0.1	0
Chr10_474	UG	HH	3194974	-	2292	2	P	2421.771	0.184	0.308
Chr10_475	UG	LH	3202413	+	573	1	P	4418.296	0.385	0
Chr10_476	UG	LH	3203880	-	222	1	P	5048.858	0.5	0
Chr10_477	UG	LH	3205968	-	3305	3	P	4708.71	0.467	0.32
Chr10_478	UG	LH	3210765	+	339	1	P	4447.663	0.375	0
Chr10_483	UG	HH	3238435	-	2941	4	P	2213.005	0.244	0.143
Chr10_485	UG	LH	3252149	+	3453	4	P	5982.735	0.364	0.079
Chr10_487	UG	LH	3265288	+	9829	9	P	3852.394	0.108	0.08
Chr10_488	UG	LH	3275818	-	258	1	A	604.777	0.143	0
Chr10_489	UG	LH	3280837	+	5087	11	P	3341.137	0.06	0.068
Chr10_490	UG	LH	3287477	+	453	1	P	3275.729	0.3	0
Chr10_492	UG	LH	3314035	+	1926	2	P	3450.593	0.5	0.059
Chr10_493	UG	LH	3321501	-	1040	2	P	2744.953	0.4	0.182
AK060990	CG	HH	3336242	+	1690	4	P	3620.786	0.077	0
Chr10_495	UG	LH	3343191	+	659	2	A	0	0	0
Chr10_496	UG	HH	3364089	+	1973	2	P	4711.967	0.071	0.063
Chr10_497	UG	LH	3373105	-	204	1	A	0	0	0
Chr10_498	UG	LH	3395308	+	2049	3	P	3151.879	0.235	0.25
Chr10_499	UG	LH	3407547	+	234	1	P	2231.227	0.5	0
Chr10_500	UG	LH	3412451	+	492	1	A	0	0	0
Chr10_501	EG	HH	3418578	-	14357	17	P	2787.342	0.242	0.132
AK065882	CG	HH	3436916	-	30848	11	P	2370.891	0.395	0.177
Chr10_502	UG	LH	3443497	-	410	2	P	4190.948	0.4	0.5
Chr10_503	EG	LH	3448230	-	4359	4	P	2906.873	0.32	0.123
AK107162	CG	LH	3455063	-	1680	5	P	2272.957	0.24	0
Chr10_506	UG	LH	3474916	+	318	1	P	6077.432	0.625	0
AK101446	CG	HH	3496383	-	4236	9	P	4487.083	0.103	0.127
AK073394	CG	HH	3507533	-	2465	10	P	4488.57	0.386	0.067
Chr10_511	UG	HH	3518796	-	4802	4	P	1804.884	0.4	0.039
Chr10_512	UG	LH	3525319	+	255	1	P	1320.698	0.167	0
Chr10_513	UG	LH	3527376	-	1352	2	P	4600.358	0.308	0.4
Chr10_514	UG	LH	3531064	+	345	2	P	3279.483	0.286	0
Chr10_515	UG	HH	3534786	-	5915	3	P	1845.568	0.364	0.049
Chr10_516	UG	HH	3548542	+	2691	7	P	2238.71	0.061	0
Chr10_518	EG	HH	3562169	+	2745	1	P	1405.866	0.098	0
Chr10_519	UG	LH	3566259	-	1699	2	A	855.869	0.143	0.1
Chr10_520	UG	LH	3573496	+	181	2	A	0	0	0

Chr10_521	UG	LH	3574992	+	948	2	A	0	0	0
Chr10_523	UG	LH	3584705	-	1851	4	A	907.717	0.143	0.077
Chr10_524	UG	LH	3590005	+	3642	4	P	2310.718	0.286	0.309
AK100367	CG	LH	3596098	+	3036	7	P	2494.966	0.143	0
Chr10_526	UG	LH	3601822	+	1446	5	P	3193.503	0.091	0.105
Chr10_527	UG	LH	3606171	+	6209	10	P	2871.325	0.077	0.092
Chr10_528	UG	LH	3622728	-	1216	3	A	895.651	0.5	0.368
Chr10_529	UG	LH	3630836	+	1217	3	P	4544.046	0.077	0
AK102113	CG	HH	3635042	-	4361	3	P	2081.681	0.191	0
Chr10_532	UG	LH	3654127	-	758	2	P	2828.858	0.429	0
Chr10_533	UG	LH	3661258	+	2129	2	P	2149.013	0.167	0
Chr10_534	EG	LH	3669296	-	382	2	P	2861.109	0.75	0
Chr10_535	UG	HH	3672128	+	2628	4	A	0	0	0.042
AK062627	CG	LH	3680229	-	566	3	P	2981.605	0.769	0
Chr10_537	UG	LH	3684140	+	2988	2	P	2309.726	0.15	0.231
AK107618	CG	LH	3694463	-	1243	5	P	3051.602	0.483	0
AK064809	CG	HH	3704675	+	4165	4	P	3035.552	0.055	0.053
Chr10_540	UG	HH	3717143	-	1635	2	P	2578.887	0.611	0.294
Chr10_541	UG	LH	3731614	-	363	1	P	3703.763	0.5	0
AK069428	CG	HH	3747669	+	6285	4	P	2216.408	0.072	0.091
Chr10_543	UG	LH	3747987	-	896	3	A	0	0	0.333
Chr10_545	UG	LH	3756197	+	1782	3	A	0	0	0
Chr10_546	UG	HH	3761847	-	490	2	P	4527.855	0.167	0
Chr10_547	UG	LH	3764891	-	436	2	P	3448.434	0.556	0
Chr10_548	EG	LH	3766792	-	2741	3	P	2477.321	0.538	0.106
AK105147	CG	HH	3770525	-	987	4	P	2578.149	0.313	0
Chr10_550	UG	HH	3773469	-	1834	3	P	1723.885	0.19	0.053
Chr10_551	UG	LH	3783906	-	408	2	A	0	0	0
Chr10_552	UG	LH	3790417	+	510	2	P	4805.783	0.5	0
Chr10_553	UG	LH	3791413	+	141	1	P	6198.228	0.5	0
Chr10_554	UG	HH	3792837	+	1215	1	P	2338.931	0.296	0
Chr10_555	EG	HH	3806981	+	5491	2	P	1054.096	0.114	0.129
Chr10_556	EG	HH	3813998	-	6315	8	P	1579.62	0.136	0.258
Chr10_560	UG	LH	3846685	-	396	1	P	10777.85	0.889	0
Chr10_561	UG	LH	3852240	-	1059	2	P	5598.79	0.667	0
Chr10_562	UG	LH	3858615	-	477	1	P	5658.865	0.545	0
Chr10_565	UG	LH	3878174	+	555	1	P	3128.988	0.308	0
Chr10_566	UG	LH	3880395	-	261	1	P	3079.577	0.714	0
Chr10_567	UG	LH	3882200	+	2854	8	P	1297.406	0.184	0
Chr10_569	UG	LH	3894112	-	468	2	P	5859.119	0.571	0.25
Chr10_570	UG	LH	3897121	-	234	1	P	5012.117	0.667	0
Chr10_571	UG	HH	3901634	+	3499	4	A	782.254	0.079	0
Chr10_572	UG	LH	3905413	+	2771	8	P	2017.223	0.111	0
AK108519	CG	HH	3912851	+	2945	2	P	1625.561	0.081	0
Chr10_574	UG	LH	3918710	-	171	1	P	4705.434	0.5	0
Chr10_575	UG	HH	3925202	+	1680	3	P	3181.212	0.323	0
Chr10_576	UG	LH	3930935	-	1040	5	P	2339.701	0.1	0
Chr10_577	UG	LH	3937432	-	955	3	A	1575.53	0.25	0
Chr10_578	UG	HH	3947164	+	1220	2	P	2738.378	0.182	0
Chr10_579	EG	HH	3950695	+	1744	3	P	1113.915	0.105	0.05
AK073662	CG	HH	3955417	-	4542	18	P	4912.217	0.755	0.14
Chr10_584	UG	LH	3991021	+	6183	12	P	3481.87	0.211	0.013
AK111418	CG	HH	3998554	-	3917	11	P	3483.468	0.367	0.139
Chr10_586	UG	LH	4004173	-	1431	3	P	3846.041	0.417	0.167
AK072061	CG	LH	4006679	+	2613	3	P	2195.621	0.174	0
Chr10_588	UG	LH	4010288	+	1227	2	P	2414.412	0.074	0
Chr10_589	UG	HH	4016650	+	1836	2	P	2937.869	0.125	0
Chr10_592	UG	HH	4036505	+	1531	2	A	0	0	0
Chr10_593	UG	LH	4041272	+	1745	5	P	1649.649	0.238	0.235
Chr10_594	UG	HH	4044826	+	5023	2	A	0	0	0.021
Chr10_597	UG	LH	4067035	-	2984	4	P	5733.511	0.143	0.107
Chr10_599	UG	LH	4077256	-	882	2	P	6152.96	0.545	0.111

AK068977	CG	LH	4077969	+	3085	8	P	1235.832	0.091	0.04
AK107891	CG	LH	4091256	+	492	1	P	1059.56	0.364	0
Chr10_601	UG	LH	4092426	+	494	2	A	774.319	0.125	0
Chr10_602	UG	LH	4096691	+	234	1	P	5980.754	0.167	0
Chr10_603	UG	LH	4100134	-	3479	4	P	7238.228	0.222	0.209
AK067281	CG	HH	4105784	+	5447	9	P	2035.666	0.122	0.055
Chr10_605	UG	HH	4113278	-	1803	1	P	4237.855	0.282	0
Chr10_606	UG	LH	4123100	-	210	1	P	1950.085	0.167	0
Chr10_607	UG	HH	4124380	-	3568	3	P	2630.416	0.167	0.19
Chr10_608	UG	LH	4128694	+	1971	5	P	2763.053	0.167	0.031
Chr10_611	EG	HH	4145202	+	2624	3	A	0	0	0.043
AK068119	CG	HH	4148912	+	6326	20	A	597.828	0.024	0
Chr10_613	UG	HH	4160993	-	453	1	P	2893.405	0.364	0
Chr10_614	UG	HH	4164948	+	2856	3	P	2079.945	0.167	0.333
Chr10_615	UG	LH	4170960	-	2134	3	P	3249.768	0.6	0.27
Chr10_616	UG	LH	4177153	+	529	3	A	0	0	0
Chr10_617	UG	LH	4190142	+	3726	6	P	2276.971	0.143	0.25
Chr10_618	UG	HH	4194642	-	1231	3	A	984.564	0.313	0.167
Chr10_623	EG	LH	4221419	-	2616	2	A	735.625	0.182	0.25
AK070240	CG	HH	4230971	-	1489	3	P	2491.596	0.25	0
Chr10_626	UG	HH	4236305	-	453	1	A	1055.624	0.3	0
AK058441	CG	HH	4249513	-	1953	4	P	2372.689	0.135	0
Chr10_629	UG	LH	4252894	-	315	1	P	5225.13	0.625	0
Chr10_630	UG	HH	4254857	-	743	2	P	6127.693	0.286	0.1
Chr10_631	UG	HH	4267507	+	2860	4	P	3994.057	0.147	0.037
Chr10_632	UG	HH	4291994	+	2450	4	P	2220.366	0.2	0.097
Chr10_633	UG	HH	4298954	+	5252	6	P	4389.651	0.136	0.014
Chr10_634	UG	HH	4306589	+	1725	4	A	991.587	0.05	0
Chr10_635	UG	LH	4310662	+	2903	5	A	0	0	0.058
Chr10_636	UG	HH	4316972	-	9275	4	P	3175.281	0.167	0.049
Chr10_637	UG	LH	4327435	+	754	2	P	2679.028	0.214	0
Chr10_638	UG	HH	4347871	+	4978	5	P	2503.927	0.177	0.044
Chr10_639	UG	LH	4355193	-	589	2	P	6035.061	0.7	0.25
Chr10_640	UG	HH	4356628	+	3212	2	A	0	0	0.111
Chr10_641	UG	HH	4365527	-	5554	3	P	1825.898	0.136	0.039
Chr10_642	UG	LH	4372585	+	201	1	A	583.85	0.2	0
Chr10_643	UG	HH	4376326	+	3427	4	P	2691.248	0.195	0.03
Chr10_644	UG	HH	4380552	-	13707	15	P	2612.586	0.247	0.175
Chr10_647	UG	LH	4413637	+	1526	3	P	2538.494	0.263	0.143
Chr10_648	UG	LH	4418044	+	714	1	P	2626.496	0.75	0
Chr10_649	UG	LH	4419225	-	1481	3	P	1976.993	0.389	0.5
Chr10_653	UG	HH	4455576	-	3811	4	P	3392.718	0.136	0.091
Chr10_654	UG	HH	4461748	-	1905	4	P	1781.337	0.296	0
Chr10_655	UG	HH	4464319	+	3595	7	P	2793.861	0.077	0.04
Chr10_656	UG	LH	4470546	-	616	2	A	1305.356	0.375	0.167
Chr10_657	UG	HH	4473209	+	1625	2	P	2994.315	0.063	0.2
Chr10_658	UG	LH	4478666	+	757	2	A	0	0	0.333
Chr10_659	UG	LH	4483472	+	483	2	A	0	0	0
Chr10_661	UG	LH	4496140	+	300	1	P	3017.091	0.571	0
Chr10_662	UG	HH	4507535	+	1125	2	A	1024.143	0.04	0
Chr10_663	UG	HH	4510354	+	4815	6	P	2403.311	0.111	0.045
Chr10_664	UG	HH	4517353	+	482	3	A	0	0	0
Chr10_666	UG	LH	4535449	+	651	2	P	3479.832	0.154	0.5
Chr10_667	EG	LH	4538283	+	567	1	A	0	0	0
Chr10_668	UG	LH	4545643	-	414	1	P	4791.777	0.2	0
Chr10_669	UG	LH	4549383	+	1105	3	P	2397.733	0.375	0.118
Chr10_670	UG	LH	4556840	-	414	1	P	5283.475	0.2	0
Chr10_671	UG	LH	4557843	+	572	2	A	0	0	0.333
Chr10_673	UG	HH	4576586	-	3659	5	P	3377.931	0.263	0.073
Chr10_674	UG	LH	4582196	+	363	1	P	4616.668	0.444	0
Chr10_675	UG	LH	4584460	-	330	1	P	6664.774	0.875	0
AK064016	CG	HH	4587174	-	4449	7	P	2652.671	0.262	0.091

Chr10_677	UG	HH	4594826	-	3134	4	P	3041.863	0.235	0.176
AK064459	CG	HH	4607047	-	2448	6	P	3188.116	0.162	0.313
Chr10_679	UG	LH	4614639	+	768	1	P	3840.554	0.235	0
Chr10_680	UG	HH	4619400	+	387	1	A	0	0	0
AK106781	CG	HH	4629843	+	979	2	A	903.782	0.15	0
AK066178	CG	HH	4643106	+	1716	1	P	2064.931	0.211	0
Chr10_684	UG	LH	4645686	-	594	2	P	1614.465	0.417	0
Chr10_685	UG	LH	4650049	-	342	1	A	0	0	0
Chr10_686	UG	LH	4656544	+	1985	4	P	5924.437	0.4	0.043
Chr10_687	UG	HH	4663084	-	5836	6	P	2087.713	0.075	0.092
AK065327	CG	HH	4672728	+	2101	10	P	4619.484	0.211	0
AK060644	CG	LH	4675389	-	2112	7	P	2869.776	0.308	0
Chr10_690	UG	HH	4681340	+	4377	1	P	1441.356	0.083	0
Chr10_691	UG	LH	4693690	-	1333	2	A	938.954	0.12	0
Chr10_692	UG	HH	4704688	-	8742	10	P	2383.901	0.1	0.062
Chr10_697	UG	LH	4729148	-	854	2	P	3528.217	0.417	0.571
AK070803	CG	HH	4734302	-	35590	5	P	1291.062	0.22	0.153
Chr10_699	UG	LH	4751673	+	1428	1	A	784.64	0.156	0
Chr10_701	UG	LH	4769495	-	200	2	A	0	0	0
Chr10_703	UG	HH	4797720	-	225	2	A	0	0	0
Chr10_704	UG	LH	4799272	-	2311	3	A	545.337	0.071	0.027
Chr10_705	UG	LH	4809968	+	2428	5	P	1634.678	0.091	0
AK066480	CG	HH	4812957	-	9107	21	P	1863.092	0.27	0.037
Chr10_707	UG	LH	4829443	-	755	2	P	3094.516	0.231	0.75
AK059127	CG	LH	4842157	+	2545	9	P	1632.575	0.086	0
AK068615	CG	HH	4844898	-	14710	20	P	2974.732	0.164	0.087
Chr10_710	UG	LH	4863001	+	1080	2	P	3644.263	0.333	0.25
Chr10_711	UG	LH	4864966	+	2910	4	P	2484.935	0.258	0.103
AK066368	CG	HH	4883938	+	10584	22	P	2481.719	0.114	0.08
Chr10_716	UG	LH	4896291	-	888	1	P	3429.799	0.65	0
Chr10_717	UG	LH	4897561	+	789	1	A	956.642	0.111	0
Chr10_718	UG	LH	4899264	-	3381	3	A	759.533	0.125	0.061
Chr10_720	UG	HH	4923092	-	882	2	P	2589.896	0.368	0
Chr10_721	UG	LH	4929821	+	4128	6	A	761.968	0.1	0.017
Chr10_722	UG	HH	4936264	+	5844	10	P	2748.975	0.061	0.048
AK065484	CG	HH	4946596	-	4188	3	P	1286.657	0.165	0
Chr10_724	UG	LH	4953194	-	1325	2	A	0	0	0.136
Chr10_725	UG	LH	4956395	-	174	1	A	1412.328	0.2	0
Chr10_726	UG	LH	4959247	-	969	2	A	1448.477	0.286	0.2
Chr10_728	UG	LH	4967729	-	977	2	P	4677.061	0.19	0
Chr10_729	EG	LH	4971341	-	1681	4	P	2416.811	0.4	0.217
AK066528	CG	LH	4971524	+	1784	1	P	1224.368	0.075	0
Chr10_730	UG	LH	4974655	-	1260	1	P	2916.869	0.345	0
Chr10_731	UG	LH	4977856	-	1356	1	P	1688.555	0.233	0
AK099711	CG	HH	4999657	+	5388	3	P	1635.564	0.156	0.081
Chr10_737	UG	LH	5008140	-	793	3	A	0	0	0.125
Chr10_738	UG	LH	5011785	+	197	2	A	0	0	0
Chr10_739	EG	HH	5015989	+	2923	2	P	4979.922	0.064	0
Chr10_740	UG	LH	5019482	-	403	2	A	0	0	0.2
Chr10_741	UG	HH	5022504	+	1273	3	P	1152.507	0.091	0.167
AK058856	CG	HH	5040002	+	867	3	P	1764.636	0.353	0
Chr10_743	UG	LH	5045831	+	1360	2	P	3820.247	0.357	0.067
Chr10_745	UG	LH	5072200	-	327	1	P	3236.376	0.75	0
Chr10_747	UG	HH	5093098	+	4229	5	P	3181.036	0.16	0.03
Chr10_748	UG	LH	5100445	+	840	2	A	0	0	0.083
Chr10_749	UG	HH	5102678	-	657	1	P	2766.989	0.4	0
Chr10_750	UG	LH	5106846	+	727	2	P	2412.508	0.25	0.125
AK065091	CG	LH	5110273	+	2244	3	P	3524.043	0.35	0.1
Chr10_752	UG	HH	5118900	+	3460	6	P	2456.758	0.087	0.167
Chr10_753	UG	LH	5123149	-	894	2	A	0	0	0.214
Chr10_754	UG	HH	5126077	-	639	1	P	2465.054	0.286	0
Chr10_755	UG	HH	5133761	-	666	1	P	5100.656	0.533	0

Chr10_759	UG	HH	5154946	+	645	1	A	499.09	0.067	0
Chr10_760	UG	HH	5163944	+	1988	3	P	2119.151	0.088	0
Chr10_761	UG	LH	5167109	+	1381	4	A	0	0	0
AK061320	CG	HH	5232959	+	1037	2	P	4441.893	0.208	0
Chr10_763	UG	HH	5236497	+	2599	3	P	2661.354	0.048	0.167
Chr10_764	UG	HH	5240129	+	1310	3	A	876.892	0.04	0.2
Chr10_765	UG	LH	5242229	-	1680	3	P	2022.712	0.286	0.125
Chr10_766	UG	HH	5246359	+	4092	7	P	6797.49	0.171	0.063
Chr10_767	UG	LH	5251933	+	510	1	P	2164.075	0.167	0
AK108031	CG	LH	5256216	+	1275	4	P	2822.659	0.207	0
AK071062	CG	LH	5269602	-	5386	6	P	2931.458	0.469	0.141
Chr10_770	UG	LH	5283482	-	435	1	P	3372.346	0.3	0
Chr10_771	UG	LH	5290103	-	558	1	P	2949.31	0.462	0
Chr10_772	UG	LH	5296504	+	904	2	P	1403.514	0.286	0.2
AK070718	CG	HH	5313407	+	5749	6	A	0	0	0.072
Chr10_776	UG	LH	5320535	+	3414	7	A	681.115	0.043	0.039
Chr10_777	UG	HH	5327176	-	8240	6	P	2697.409	0.156	0.169
AK070874	CG	HH	5346043	+	5925	11	P	2667.946	0.133	0.06
Chr10_779	UG	LH	5365100	+	3868	4	P	2208.151	0.429	0.136
AK064893	CG	HH	5370154	-	7358	23	P	2795.5	0.419	0.111
Chr10_781	UG	LH	5381512	-	1203	3	P	6235.528	0.389	0.333
Chr10_782	UG	HH	5389039	+	3140	6	P	1821.628	0.017	0.1
Chr10_784	UG	LH	5409386	-	258	1	A	1365.007	0.143	0
Chr10_785	UG	LH	5411647	-	1662	2	P	7976.494	0.273	0.04
Chr10_786	UG	HH	5414474	-	8854	9	P	3110.535	0.224	0.282
AK066828	CG	HH	5437851	-	2356	3	P	1955.887	0.4	0.045
Chr10_788	UG	LH	5443297	+	600	1	P	4569.686	0.429	0
Chr10_789	UG	HH	5444378	-	4155	6	A	1097.63	0.222	0.256
Chr10_790	UG	HH	5450463	+	1593	3	P	2900.035	0.154	0.2
AK070455	CG	HH	5454485	-	3947	5	P	1561.65	0.292	0.045
Chr10_792	UG	HH	5465970	+	3049	3	P	3947.485	0.2	0.038
AK070457	CG	HH	5474116	+	5969	7	P	7840.607	0.081	0.032
Chr10_794	UG	HH	5476702	-	2661	4	P	3673.655	0.179	0.158
Chr10_795	UG	LH	5481909	-	3670	5	P	4149.926	0.259	0.103
Chr10_797	UG	HH	5507894	+	692	2	A	0	0	0
Chr10_800	EG	HH	5522483	+	531	1	P	1619.88	0.25	0
Chr10_801	UG	LH	5526019	-	2961	3	P	4317.465	0.417	0.211
Chr10_802	UG	HH	5536135	-	2108	3	P	2114.727	0.174	0.13
Chr10_803	UG	LH	5539596	+	2311	3	P	2261.456	0.1	0.025
Chr10_804	EG	HH	5542985	+	1008	1	P	1439.018	0.227	0
Chr10_805	UG	LH	5547797	+	453	1	P	1894.917	0.2	0
Chr10_809	UG	HH	5589922	-	2435	4	P	2018.409	0.27	0.143
AK107022	CG	LH	5594961	+	13638	3	P	4966.714	0.111	0.048
Chr10_811	UG	LH	5609878	-	171	1	P	2011.944	0.5	0
Chr10_812	UG	LH	5612500	-	2712	8	P	1592.412	0.147	0.077
Chr10_814	UG	LH	5634246	-	1974	4	A	0	0	0.229
AK068221	CG	HH	5641633	+	3344	7	P	1129.951	0.077	0.088
Chr10_816	UG	LH	5648676	+	4871	5	P	6912.784	0.063	0.071
Chr10_817	UG	HH	5654290	-	3454	2	P	2864.453	0.265	0.5
AK063188	CG	LH	5659740	+	385	1	A	722.014	0.222	0
Chr10_818	UG	LH	5659767	-	1869	2	P	8501.396	0.8	0.259
Chr10_819	UG	LH	5666303	+	5489	6	A	0	0	0.053
Chr10_820	UG	LH	5676748	+	2307	2	A	1045.45	0.2	0.079
AK106877	CG	HH	5683757	+	8385	8	P	2574.7	0.068	0.098
Chr10_822	UG	LH	5689252	+	2608	6	P	4007.876	0.267	0.083
Chr10_824	UG	LH	5707692	-	398	2	P	4473.483	0.25	1
Chr10_826	UG	HH	5719372	+	862	2	P	3992.499	0.083	0
Chr10_827	UG	LH	5722748	-	705	2	P	2323.11	0.167	0.125
Chr10_828	UG	HH	5725746	+	1012	3	P	4340.958	0.174	0
AK100207	CG	HH	5756119	+	6242	4	P	2456.338	0.1	0.059
Chr10_833	EG	HH	5794450	+	3893	4	P	2013.413	0.105	0
Chr10_834	UG	HH	5800126	+	1053	2	P	7927.221	0.167	0

Chr10_836	UG	HH	5804996	-	1343	2	P	2516.146	0.407	0
Chr10_837	UG	HH	5807845	+	697	2	P	3134.217	0.313	0
Chr10_838	UG	LH	5816606	-	3689	5	P	3380.079	0.333	0.117
Chr10_839	UG	LH	5821541	-	1320	2	P	3720.206	0.217	0.5
Chr10_840	UG	HH	5824551	-	2041	6	P	3245.015	0.242	0.167
Chr10_841	UG	LH	5827773	-	1350	1	P	1693.029	0.3	0
AK074024	CG	LH	5833417	-	2383	4	P	3518.508	0.158	0.394
Chr10_843	UG	LH	5843722	-	703	2	P	2061.959	0.375	0
Chr10_844	UG	LH	5851677	+	261	1	P	3631.409	0.833	0
Chr10_845	UG	HH	5857853	-	6184	6	P	2476.284	0.162	0.179
AK068249	CG	HH	5866511	+	9864	7	P	3859.739	0.179	0.064
Chr10_848	EG	HH	5913443	-	1167	4	A	0	0	0
Chr10_849	UG	LH	5920413	-	444	2	P	4689.878	0.167	0
Chr10_850	UG	LH	5927537	-	355	2	P	2527.768	0.625	0
Chr10_851	UG	LH	5935074	-	3531	5	A	822.249	0.167	0.212
Chr10_855	UG	HH	5975765	-	2570	4	P	1836.552	0.308	0.25
Chr10_856	UG	HH	5982227	-	6309	8	P	2696.91	0.3	0.067
Chr10_857	UG	LH	5989245	+	1043	2	P	3165.394	0.571	0.063
Chr10_858	UG	LH	5996022	-	2316	7	P	1995.174	0.179	0.167
Chr10_859	UG	LH	5998806	-	842	2	A	1134.606	0.278	0
Chr10_860	UG	LH	6001356	+	552	1	P	7180.577	0.75	0
Chr10_861	UG	LH	6002303	-	1210	3	P	2772.248	0.167	0.25
Chr10_862	UG	HH	6006260	-	2554	4	P	2486.158	0.053	0.056
Chr10_864	UG	HH	6033340	+	417	2	A	0	0	0
Chr10_865	UG	HH	6034009	-	730	2	P	2544.804	0.353	0
Chr10_866	UG	LH	6035512	-	463	2	A	555.425	0.1	0
Chr10_867	UG	LH	6041577	+	2520	4	P	4624.786	0.044	0.1
Chr10_869	UG	LH	6053695	+	3931	6	A	0	0	0.02
Chr10_871	UG	HH	6060272	+	1891	5	A	0	0	0
AK100514	CG	HH	6075587	-	153446	14	P	3021.884	0.271	0.158
Chr10_874	UG	LH	6102552	-	624	3	A	1122.833	0.364	0
Chr10_875	UG	HH	6106378	+	1844	2	P	6594.673	0.067	0.292
AK064659	CG	LH	6128234	+	9257	17	P	4392.479	0.083	0.015
Chr10_878	UG	LH	6144361	+	2487	2	P	6168.323	0.167	0.069
Chr10_882	UG	LH	6162626	-	967	2	A	936.928	0.167	0
Chr10_884	UG	LH	6173972	+	1547	2	P	1680.106	0.067	0.053
Chr10_885	EG	HH	6176918	-	4127	4	P	3702.82	0.355	0.119
Chr10_887	UG	LH	6185361	+	532	2	P	4149.678	0.3	0
Chr10_888	UG	HH	6189175	+	4611	7	P	1928.428	0.014	0.037
Chr10_889	UG	LH	6194112	+	809	2	P	1848.947	0.333	0.167
AK103839	CG	HH	6198013	-	5620	15	P	1874.882	0.557	0.098
Chr10_894	UG	LH	6232105	-	465	2	P	2085.279	0.667	0
Chr10_897	UG	LH	6252055	-	942	2	P	1707.871	0.143	0
AK108741	CG	HH	6255321	+	2889	4	P	7369.289	0.081	0.077
AK107374	CG	HH	6258736	-	3945	5	P	1760.442	0.526	0.067
Chr10_900	UG	LH	6263756	-	1663	3	P	1881.691	0.125	0.095
AK064534	CG	HH	6274157	+	4332	6	P	1749.456	0.068	0.02
Chr10_902	UG	LH	6280368	-	4702	4	P	2564.919	0.45	0.218
Chr10_903	EG	HH	6288627	-	941	2	P	3645.955	0.368	0.5
Chr10_904	UG	LH	6297015	-	1376	3	P	3999.727	0.5	0.053
Chr10_905	UG	LH	6303068	-	1015	4	A	1293.324	0.111	0.071
Chr10_906	UG	LH	6312191	-	1946	2	P	4959.411	0.304	0.263
Chr10_907	UG	LH	6316935	+	761	2	A	0	0	0
AK101612	CG	HH	6329857	+	5854	12	P	2754.798	0.094	0.053
Chr10_909	UG	LH	6335976	-	1063	2	P	1866.444	0.167	0
Chr10_910	UG	HH	6340563	-	9761	6	P	2866.962	0.205	0.25
Chr10_911	UG	HH	6353305	+	3376	2	P	2033.048	0.042	0.5
Chr10_912	UG	LH	6361345	+	210	2	A	0	0	0.5
Chr10_913	EG	LH	6364621	+	252	1	P	1939.332	0.667	0
AK064393	CG	HH	6376145	-	995	4	P	2386.246	0.19	0
Chr10_918	UG	HH	6401613	+	3612	6	P	3171.597	0.032	0.063
AK108231	CG	LH	6407139	-	1160	3	P	3958.859	0.158	0

Chr10_920	UG	LH	6417395	+	2526	3	A	0	0	0
Chr10_925	UG	HH	6434362	-	2520	6	A	1153.183	0.118	0.105
AK065698	CG	LH	6444852	+	6542	6	P	2022.697	0.146	0.045
Chr10_926	UG	LH	6444896	-	724	2	P	3019.28	0.625	0
AK060372	CG	HH	6453081	+	3427	6	A	711.36	0.079	0.053
AK068630	CG	HH	6455761	-	6315	2	P	2318.043	0.333	0.09
Chr10_930	UG	LH	6470368	+	2692	3	A	907.573	0.273	0.303
Chr10_932	UG	LH	6480284	+	426	1	P	10226.37	0.333	0
Chr10_933	EG	HH	6484012	-	1753	8	P	1781.973	0.192	0
Chr10_934	UG	HH	6487821	-	379	2	A	0	0	0
AK067578	CG	HH	6520464	-	6411	11	P	2075.069	0.4	0.11
Chr10_938	UG	HH	6530361	-	237	2	P	3689.087	0.333	0
Chr10_939	UG	HH	6536359	+	5880	9	P	1681.325	0.094	0.083
Chr10_941	UG	LH	6552546	+	228	1	A	0	0	0
Chr10_943	UG	LH	6579036	+	410	2	P	4038.806	0.333	0
Chr10_944	UG	HH	6579625	-	4386	2	P	2878.842	0.152	0.25
AK109197	CG	LH	6589822	-	9985	4	P	1476.772	0.4	0.138
Chr10_946	UG	LH	6594099	+	671	2	P	2664.418	0.308	0
AK064164	CG	LH	6599492	+	556	2	P	3410.468	0.286	0
Chr10_947	UG	HH	6603272	+	414	1	A	0	0	0
Chr10_949	UG	LH	6623933	+	823	2	P	7709.303	0.125	0
Chr10_950	UG	LH	6625315	-	399	1	P	4880.414	0.333	0
AK069438	CG	LH	6626286	+	7255	9	P	2881.21	0.119	0.053
Chr10_956	EG	LH	6648442	+	2359	3	A	1060.797	0.143	0
Chr10_961	UG	LH	6688322	-	495	2	P	4576.696	0.667	0.6
Chr10_964	UG	LH	6702853	-	3051	5	P	2859.788	0.091	0.071
AK063872	CG	HH	6716368	+	710	1	P	10383.182	0.063	0
Chr10_966	EG	LH	6722802	+	2642	4	P	2291.479	0.375	0.1
Chr10_967	UG	LH	6727957	-	3871	4	A	1264.108	0.091	0
Chr10_968	UG	LH	6732829	+	2543	6	P	3091.673	0.1	0.133
Chr10_969	UG	HH	6740610	-	885	1	A	914.821	0.2	0
Chr10_970	UG	HH	6741753	-	1158	2	A	1537.152	0.087	0
Chr10_971	UG	HH	6743708	-	5167	4	P	4274.11	0.151	0.243
Chr10_972	UG	LH	6754634	+	1004	2	A	0	0	0.154
Chr10_974	EG	LH	6761413	-	1481	6	P	3291.334	0.462	0.056
Chr10_975	UG	LH	6769771	-	1417	3	P	1558.191	0.286	0.111
Chr10_976	UG	HH	6772392	-	309	1	P	2410.748	0.25	0
Chr10_977	EG	HH	6774808	-	666	3	P	3546.054	0.545	0.75
Chr10_978	UG	HH	6777322	-	2426	5	P	2249.627	0.167	0.091
Chr10_979	UG	HH	6782157	+	3846	7	P	3210.013	0.042	0.121
Chr10_980	UG	LH	6788920	-	997	2	A	1100.166	0.2	0
Chr10_981	UG	LH	6791666	+	537	1	A	0	0	0
AK065772	CG	HH	6800125	-	14574	9	P	2481.173	0.153	0.133
Chr10_993	UG	LH	6871787	+	594	2	A	0	0	0
AK070442	CG	HH	6875294	+	9962	2	P	1126.736	0.289	0.051
AK068235	CG	LH	6887464	+	3204	5	P	2698.894	0.043	0
Chr10_996	UG	LH	6896064	+	390	1	P	811.807	0.222	0
AK105548	CG	LH	6898079	-	2618	4	P	1120.33	0.3	0
Chr10_997	UG	LH	6900000	+	855	2	P	2146.662	0.5	0
Chr10_1001	UG	HH	6921736	-	13149	10	P	2130.53	0.233	0.165
AK111448	CG	LH	6955902	+	1673	2	P	2175.677	0.324	0
Chr10_1005	UG	LH	6964428	-	459	2	P	4929.905	0.222	0
Chr10_1006	UG	LH	6977432	+	300	1	P	2782.413	0.625	0
Chr10_1007	UG	LH	6989663	-	438	1	P	4199.295	0.3	0
Chr10_1009	UG	LH	7008972	-	465	1	A	338.508	0.091	0
AK064736	CG	HH	7020991	+	1957	3	P	1999.629	0.116	0
Chr10_1011	UG	LH	7024784	+	2377	4	P	2088.974	0.235	0.067
Chr10_1013	EG	HH	7036879	-	3413	4	P	1686.054	0.133	0.233
Chr10_1014	UG	HH	7043005	-	395	2	P	2713.78	0.333	0
Chr10_1015	UG	LH	7047341	-	2485	4	P	2209.508	0.2	0.12
Chr10_1016	UG	LH	7053166	-	2573	2	P	1306.858	0.313	0.056
AK072694	CG	HH	7061708	+	5336	10	P	882.451	0.359	0.128

Chr10_1018	UG	LH	7072705	+	2731	7	P	1635.032	0.109	0
Chr10_1022	UG	LH	7101486	+	1959	3	P	1685.571	0.294	0
Chr10_1023	UG	HH	7103722	-	1473	2	A	0	0	0
Chr10_1025	UG	LH	7119241	-	2366	3	P	2278.256	0.355	0.182
Chr10_1026	UG	LH	7123901	-	468	1	P	1141.783	0.273	0
Chr10_1027	UG	HH	7126283	-	588	1	P	878.397	0.077	0
Chr10_1028	UG	HH	7128172	+	670	2	P	2729.627	0.267	0
Chr10_1029	EG	LH	7132602	-	2612	2	P	2293.504	0.4	0.222
AK066810	CG	LH	7137867	-	2225	3	P	1462.444	0.102	0
Chr10_1030	EG	HH	7140023	-	992	4	P	3735.101	0.25	0.2
Chr10_1031	UG	HH	7146286	+	4621	5	P	2757.183	0.114	0.135
Chr10_1032	UG	LH	7155106	+	614	2	P	1994.402	0.125	0.5
AK106038	CG	HH	7156625	+	21846	2	P	2816.867	0.216	0.073
Chr10_1036	UG	LH	7183240	+	4280	11	P	3072.718	0.189	0.053
Chr10_1037	UG	LH	7188238	-	276	1	A	0	0	0
Chr10_1038	UG	HH	7192676	-	1216	4	P	1263.949	0.111	0
Chr10_1039	UG	LH	7198458	-	1184	3	P	3500.421	0.333	0.118
AK110835	CG	LH	7223896	+	19158	11	P	2431.46	0.204	0.101
Chr10_1042	UG	LH	7224447	-	1053	3	P	814.754	0.111	0.133
Chr10_1044	UG	LH	7235364	-	2503	4	P	2863.649	0.182	0
Chr10_1046	UG	LH	7248160	-	396	1	P	1000.53	0.625	0
Chr10_1048	UG	LH	7253113	-	2462	3	P	2900.012	0.231	0.133
Chr10_1051	UG	LH	7271975	-	3328	9	P	3461.189	0.156	0.038
AK071372	CG	HH	7278140	+	5883	13	P	516.554	0.204	0.039
AK070595	CG	HH	7285714	+	1106	3	P	1715.881	0.409	0
Chr10_1054	UG	LH	7292076	-	2564	5	P	3037.677	0.353	0.231
AK100089	CG	HH	7295042	-	1068	6	P	4189.604	0.167	0
Chr10_1056	UG	HH	7297721	-	2589	5	P	1444.06	0.087	0
Chr10_1059	UG	LH	7310142	-	389	2	P	5686.22	0.111	0
Chr10_1060	UG	HH	7320766	-	4752	4	P	2051.083	0.2	0.092
Chr10_1067	UG	LH	7356946	-	294	1	P	4457.343	0.833	0
AK066144	CG	HH	7361409	+	4729	3	P	2373.332	0.156	0.014
Chr10_1069	UG	LH	7366255	-	1330	7	A	524.794	0.059	0
Chr10_1070	UG	LH	7368357	-	1557	2	A	765.273	0.25	0
Chr10_1071	EG	HH	7373696	+	4328	7	P	1650.723	0.143	0.172
Chr10_1072	UG	HH	7379066	+	1236	1	P	3107.453	0.37	0
AK060392	CG	LH	7382017	+	2045	6	P	2663.683	0.3	0
Chr10_1074	UG	LH	7385155	+	534	2	P	7086.435	0.5	0
Chr10_1075	UG	LH	7390118	+	456	1	P	1907.862	0.273	0
Chr10_1076	UG	LH	7393395	+	297	1	P	700.559	0.571	0
Chr10_1078	UG	HH	7419006	+	4696	3	P	1192.675	0.08	0.02
AK107248	CG	LH	7433275	-	1672	3	P	1441.289	0.211	0
Chr10_1080	UG	LH	7437344	-	1507	2	P	1223.501	0.385	0.053
AK063471	CG	LH	7457000	+	1658	3	P	3743.77	0.143	0.5
Chr10_1083	UG	HH	7466883	-	6099	7	P	1466.071	0.184	0.012
Chr10_1084	UG	HH	7480574	-	3212	7	P	1329.808	0.188	0.045
Chr10_1086	UG	LH	7490362	-	585	1	P	6221.817	0.917	0
AK106742	CG	HH	7507679	-	7456	13	P	1196.08	0.077	0.099
Chr10_1091	UG	LH	7534159	-	2884	5	P	3243.041	0.333	0.5
AK100836	CG	LH	7537627	+	6333	5	P	3144.31	0.158	0.11
Chr10_1093	UG	LH	7541712	-	549	1	P	3067.483	0.385	0
Chr10_1095	EG	LH	7550073	+	447	1	P	2230.54	0.5	0
Chr10_1096	UG	LH	7563281	-	1070	2	P	1183.206	0.5	0.111
Chr10_1097	UG	LH	7570211	+	1382	2	P	819.724	0.063	0.071
AK109143	CG	LH	7597615	-	3505	4	P	2779.738	0.073	0
AK070180	CG	HH	7597780	+	2222	2	P	3567.607	0.08	0
Chr10_1102	UG	LH	7605138	-	330	1	P	2135.697	0.143	0
Chr10_1103	UG	LH	7608703	-	3764	5	P	3371.469	0.25	0.096
AK070755	CG	HH	7631312	+	5088	8	P	2079.892	0.264	0.034
Chr10_1108	UG	LH	7639851	+	447	1	A	0	0	0
AK103051	CG	LH	7641958	+	2862	7	P	747.419	0.183	0
Chr10_1109	UG	LH	7650435	+	785	2	P	2851.866	0.667	0.333

Chr10_1110	EG	HH	7653179	-	2004	8	P	2065.505	0.088	0
Chr10_1111	UG	LH	7656199	+	773	2	P	2751.354	0.111	0.375
Chr10_1112	UG	HH	7659224	-	1649	3	P	2007.56	0.217	0.077
Chr10_1113	UG	LH	7672310	-	732	1	P	1063.457	0.118	0
AK104034	CG	LH	7675267	+	747	2	P	2623.339	0.412	0
AK103827	CG	LH	7691042	+	5776	6	P	674.895	0.079	0.034
AK099421	CG	HH	7698287	+	1228	2	P	2221.702	0.44	1
Chr10_1120	UG	HH	7703093	+	3489	4	P	913.763	0.105	0.167
Chr10_1121	UG	HH	7709243	+	7056	18	P	2241.573	0.147	0
Chr10_1122	UG	LH	7722291	+	309	1	A	0	0	0
Chr10_1125	UG	HH	7730979	+	434	2	P	4812.689	0.2	0
Chr10_1126	UG	HH	7732964	-	4333	6	P	1477.652	0.121	0.036
Chr10_1127	UG	LH	7740779	+	938	2	P	2326.632	0.278	0.333
Chr10_1128	UG	LH	7745755	-	471	2	A	566.168	0.125	0
Chr10_1129	UG	LH	7747942	-	552	1	A	380.41	0.077	0
AK060146	CG	LH	7749599	+	1098	3	P	1054.98	0.077	0
Chr10_1130	EG	HH	7749608	-	3866	3	P	4298.418	0.25	0.023
Chr10_1131	UG	LH	7754322	-	339	1	P	4279.709	0.375	0
Chr10_1134	UG	HH	7769174	-	3283	3	P	4404.447	0.282	0.031
Chr10_1135	UG	HH	7775531	-	870	1	P	910.88	0.105	0
Chr10_1142	EG	LH	7828867	+	881	3	P	765.699	0.077	0
Chr10_1143	EG	LH	7848748	+	1114	3	P	4538.673	0.133	0
Chr10_1144	UG	LH	7853866	+	1716	3	P	1048.926	0.316	0
AK073900	CG	HH	7856695	+	3840	6	P	1219.185	0.241	0.019
Chr10_1148	UG	HH	7875568	+	1241	2	A	0	0	0
Chr10_1149	UG	HH	7877608	+	892	2	A	486.007	0.154	0
Chr10_1151	EG	HH	7896439	-	1173	3	A	382.819	0.063	0
Chr10_1153	UG	LH	7906811	-	486	1	P	2200.527	0.727	0
Chr10_1154	UG	LH	7921422	+	1260	1	P	2134.22	0.25	0
Chr10_1157	UG	LH	7931766	+	1004	2	P	2224.589	0.167	0
Chr10_1158	EG	LH	7933233	-	6289	7	P	1874.548	0.167	0.063
Chr10_1159	UG	LH	7942354	+	1677	2	P	1178.369	0.2	0.04
Chr10_1160	UG	LH	7947166	+	337	2	P	5808.099	0.143	0
Chr10_1161	UG	LH	7952883	-	818	2	P	2000.476	0.688	0
AK062541	CG	LH	7953169	+	743	3	P	2181.474	0.133	0
Chr10_1162	UG	LH	7956259	-	3739	5	P	2592.842	0.296	0.2
Chr10_1163	UG	LH	7964009	+	1605	2	P	1534.733	0.1	0.043
Chr10_1165	UG	LH	7970891	-	514	2	P	3980.309	0.333	0.167
Chr10_1166	EG	HH	7983408	+	6252	6	A	0	0	0.035
Chr10_1167	UG	LH	7996023	-	483	2	A	0	0	0.25
Chr10_1168	UG	HH	7998186	+	3338	2	A	269.59	0.014	0
AK073496	CG	HH	8012854	+	5733	11	P	1257.476	0.207	0
Chr10_1171	UG	HH	8019446	-	1303	5	P	1709.01	0.056	0
AK062808	CG	LH	8026086	+	647	2	P	2393.401	0.375	0
Chr10_1173	UG	LH	8027563	-	393	1	A	608.31	0.222	0
Chr10_1174	UG	LH	8034454	+	1219	2	P	773.924	0.192	0
Chr10_1176	UG	LH	8051117	-	271	2	A	0	0	0
Chr10_1177	UG	HH	8053197	-	1841	3	P	1288.017	0.133	0.091
Chr10_1178	UG	HH	8056475	-	4666	6	P	1592.075	0.107	0.07
Chr10_1179	UG	LH	8066388	-	483	1	P	2237.824	0.3	0
Chr10_1181	UG	LH	8074540	+	427	2	P	2143.233	0.5	0.333
Chr10_1182	UG	LH	8076091	-	489	1	P	5151.847	0.182	0
Chr10_1184	UG	LH	8088475	-	1789	3	P	3664.601	0.308	0.24
AK066876	CG	HH	8108658	+	6981	19	P	805.322	0.171	0.025
Chr10_1187	UG	LH	8116439	-	699	3	A	563.323	0.3	0.5
Chr10_1188	EG	LH	8118034	+	1167	2	P	1776.957	0.333	0.071
AK101675	CG	HH	8118827	-	10361	10	P	2146.695	0.101	0.039
AK109165	CG	LH	8128663	+	556	1	P	1762.198	0.385	0
AK111390	CG	LH	8192489	+	2027	5	P	3123.392	0.217	0.087
Chr10_1193	UG	LH	8212905	-	1318	2	P	2871.521	0.455	0
Chr10_1194	UG	LH	8221594	+	456	1	P	2192.197	0.5	0
Chr10_1195	UG	HH	8229525	-	4977	17	P	2182.565	0.101	0.026

Chr10_1196	UG	LH	8249678	+	6646	8	P	2757.374	0.176	0.111
Chr10_1197	UG	LH	8257572	+	423	1	P	2167.895	0.7	0
Chr10_1198	EG	LH	8260894	+	1347	6	P	2913.485	0.167	0
AK069071	CG	HH	8271135	+	5220	9	P	1994.878	0.172	0.019
Chr10_1200	UG	LH	8277527	+	3399	4	P	3239.079	0.464	0.098
Chr10_1201	UG	HH	8284937	-	2403	1	P	927.769	0.057	0
Chr10_1202	UG	LH	8293703	-	1496	4	P	3029.592	0.545	0.043
Chr10_1203	UG	HH	8303743	-	2423	3	P	965.66	0.16	0
Chr10_1206	UG	LH	8315624	-	2992	8	P	1510.651	0.103	0.056
Chr10_1207	UG	LH	8319896	+	633	1	A	676.015	0.071	0
AK069512	CG	LH	8334827	+	2088	7	P	2426.303	0.229	0
AK100724	CG	HH	8342663	+	3866	11	P	2090.316	0.185	0.088
AK108244	CG	HH	8346610	-	2724	4	P	2731.033	0.24	0.057
AK110467	CG	HH	8352513	-	8247	3	P	3502.122	0.246	0.131
Chr10_1213	UG	LH	8363521	-	672	2	P	7370.85	0.067	0
Chr10_1214	UG	LH	8366699	-	1978	4	P	2981.022	0.765	0.13
Chr10_1216	UG	LH	8374796	-	177	1	A	690.636	0.4	0
Chr10_1217	EG	HH	8380163	-	1030	2	P	1109.565	0.111	0.333
Chr10_1218	UG	LH	8384017	-	537	1	P	7287.22	0.75	0
AK071027	CG	HH	8388796	-	2254	3	P	3337.174	0.083	0
Chr10_1220	UG	HH	8398271	+	684	2	A	641.893	0.143	0
Chr10_1221	UG	LH	8399588	+	959	2	A	535.501	0.048	0
Chr10_1222	UG	LH	8403490	+	1003	2	P	1387.415	0.5	0.063
AK105578	CG	HH	8404588	-	2617	8	P	2841.049	0.091	0
Chr10_1224	UG	HH	8412811	+	3664	5	P	1202.825	0.273	0.114
Chr10_1226	UG	LH	8429182	-	471	1	P	4257.069	0.636	0
AK073245	CG	HH	8439364	+	2667	9	P	1993.881	0.091	0
Chr10_1228	UG	LH	8446337	-	1540	5	P	1231.092	0.091	0.043
AK069792	CG	HH	8450913	-	2719	9	P	901.45	0.146	0
AK064432	CG	LH	8470942	-	2021	6	P	2105.356	0.106	0
Chr10_1232	UG	LH	8481555	-	1127	2	P	2852	0.154	0.273
Chr10_1233	EG	LH	8488515	+	4491	5	P	3356.774	0.258	0.06
Chr10_1235	UG	LH	8499377	-	729	2	P	3117.312	0.583	0.25
Chr10_1236	UG	LH	8503365	+	2999	4	P	4169.318	0.474	0.022
Chr10_1237	EG	LH	8507762	+	416	2	A	0	0	0
Chr10_1238	UG	LH	8509981	+	1139	2	P	767.905	0.143	0
AK106542	CG	HH	8528200	+	5224	10	P	657.667	0.194	0.078
AK110474	CG	HH	8536075	-	9653	10	P	1051.49	0.13	0.04
Chr10_1242	UG	LH	8538331	+	1750	4	A	352.243	0.071	0
Chr10_1244	EG	HH	8564147	-	3837	7	A	595.629	0.053	0.046
Chr10_1247	UG	LH	8578936	+	369	1	P	4603.368	0.222	0
Chr10_1248	UG	HH	8583208	-	2175	1	P	1849.345	0.292	0
Chr10_1249	EG	HH	8588445	-	3438	6	P	6475.968	0.208	0.167
AK102558	CG	LH	8593371	-	2580	5	P	1730.187	0.183	0
AK073552	CG	HH	8601504	-	4142	4	P	2090.677	0.216	0.019
Chr10_1251	UG	LH	8612346	+	1358	3	P	1568.76	0.125	0.167
Chr10_1253	UG	LH	8629592	+	1375	2	P	3210.035	0.364	0.056
Chr10_1254	UG	LH	8634161	-	1212	2	P	1509.716	0.4	0.227
AK059934	CG	LH	8637323	-	444470	9	A	539.776	0.111	0.103
Chr10_1256	UG	LH	8661587	-	1542	1	P	1236.719	0.059	0
Chr10_1258	UG	LH	8676894	+	3628	5	P	3070.64	0.303	0.256
Chr10_1259	UG	LH	8689737	+	285	1	P	4799.368	0.333	0
Chr10_1260	UG	LH	8692173	-	312	1	P	752.7	0.429	0
Chr10_1261	UG	HH	8697685	-	3914	4	P	2097.329	0.291	0.107
Chr10_1262	UG	LH	8708512	-	203	2	P	1063.64	0.333	0
Chr10_1263	UG	LH	8710158	-	485	2	P	3551.559	0.714	0.333
Chr10_1266	EG	HH	8734399	+	947	2	P	1347.793	0.143	0
Chr10_1267	UG	HH	8739360	+	6166	7	P	1655.64	0.131	0.056
AK072565	CG	HH	8753620	+	5306	5	P	1577.099	0.172	0.057
Chr10_1269	UG	HH	8760451	-	4589	4	P	1744.338	0.139	0.023
Chr10_1270	UG	LH	8766809	-	255	1	A	0	0	0
Chr10_1271	UG	HH	8768327	-	1108	2	P	860.119	0.333	0.063

AK062848	CG	HH	8776478	-	665	2	P	1602.768	0.188	0
Chr10_1273	UG	HH	8777651	+	1008	3	P	1476.542	0.444	0
Chr10_1274	UG	LH	8780460	-	279	2	P	3469.541	0.167	0
Chr10_1284	UG	LH	8827936	-	5204	8	P	1798.157	0.135	0.132
AK070298	CG	HH	8836241	-	4430	3	P	2768.254	0.181	0.167
Chr10_1286	UG	HH	8851895	-	1671	2	P	6131.148	0.077	0.083
Chr10_1287	UG	LH	8857407	+	1321	2	A	551.55	0.167	0.091
Chr10_1288	UG	HH	8860961	-	1037	2	P	1794.036	0.211	0.2
Chr10_1289	UG	HH	8863269	-	1490	2	P	668.301	0.333	0
Chr10_1291	UG	LH	8886039	+	1311	3	P	5898.814	0.4	0.176
AK102659	CG	HH	8900711	+	16428	7	P	1064.041	0.233	0.12
Chr10_1294	UG	LH	8910681	+	1239	3	P	1007.933	0.154	0.2
Chr10_1297	UG	HH	8930890	+	2046	3	P	2188.046	0.081	0.25
AK070631	CG	HH	8944796	+	3617	9	P	1103.225	0.06	0
Chr10_1299	UG	HH	8949002	+	2951	2	P	2440.752	0.04	0.042
Chr10_1300	UG	LH	8956505	-	330	1	A	330.819	0.25	0
Chr10_1301	UG	HH	8959825	-	2537	3	P	2135.535	0.308	0.125
Chr10_1304	UG	LH	8988872	-	345	1	P	1880.8	0.375	0
Chr10_1305	UG	LH	8993251	-	390	1	P	5927.815	1	0
AK062380	CG	HH	9014199	+	714	1	P	889.999	0.438	0
Chr10_1306	UG	LH	9016858	-	529	2	P	3910.317	0.429	0.4
AK106577	CG	LH	9024055	+	44022	3	P	2588.879	0.159	0.126
Chr10_1308	UG	HH	9032294	+	1762	3	P	1015.156	0.071	0.091
AK106693	CG	LH	9045203	-	3373	9	P	3105.043	0.217	0.034
AK064907	CG	HH	9049086	+	6789	14	P	879.141	0.115	0.055
Chr10_1311	UG	LH	9058992	+	6578	12	P	2292.623	0.25	0.186
Chr10_1312	UG	LH	9072475	+	7358	15	P	1337.794	0.147	0.086
Chr10_1313	UG	LH	9084675	+	600	2	P	843.512	0.125	0.167
Chr10_1314	UG	HH	9088900	+	1875	3	P	2723.403	0.105	0
Chr10_1315	UG	LH	9091979	-	2810	5	P	1181.583	0.1	0.097
Chr10_1316	UG	LH	9098277	-	668	2	P	1925.986	0.2	0
Chr10_1317	UG	LH	9102984	+	4824	4	P	3331.065	0.071	0.039
AK068930	CG	HH	9119396	+	4324	7	P	2429.525	0.314	0.083
AK101176	CG	HH	9122344	-	6098	6	P	2579.666	0.129	0.082
Chr10_1320	UG	LH	9128751	-	1967	3	A	613.38	0.111	0.313
Chr10_1321	UG	LH	9133841	-	459	1	P	1945.064	0.1	0
Chr10_1322	UG	LH	9140537	+	1012	3	P	2228.584	0.667	0.5
Chr10_1323	UG	LH	9144500	-	2616	5	P	1898.802	0.205	0
Chr10_1324	UG	HH	9155123	+	4923	4	P	3149.114	0.176	0.041
Chr10_1325	UG	LH	9162520	+	222	1	A	664.36	0.4	0
AK072682	CG	LH	9166717	-	4936	7	P	2758.974	0.114	0.031
Chr10_1327	UG	LH	9181654	-	451	2	P	3069.987	0.5	0
Chr10_1328	EG	HH	9183095	-	3912	3	P	1113.215	0.161	0.196
AK106326	CG	LH	9186725	-	1970	5	P	1026.305	0.064	0
Chr10_1336	UG	LH	9249571	-	576	2	P	3699.192	0.111	0.5
AK063055	CG	LH	9255108	+	14087	2	P	1112.448	0.214	0.153
AK063230	CG	LH	9269957	-	640	1	P	2481.156	0.267	0
Chr10_1337	UG	HH	9270131	+	4021	3	P	702.533	0.083	0
Chr10_1338	UG	HH	9310922	+	285	1	A	401.152	0.143	0
Chr10_1339	UG	LH	9314709	+	1140	2	P	1227.686	0.176	0.111
Chr10_1340	UG	LH	9318176	+	3933	4	A	0	0	0.065
Chr10_1341	UG	LH	9323932	-	684	2	P	3806.866	0.571	0.5
Chr10_1342	UG	HH	9329095	+	4257	7	P	1665.226	0.029	0.034
Chr10_1343	UG	LH	9334579	+	1407	4	P	2286.901	0.063	0
Chr10_1344	UG	LH	9342202	-	865	3	A	0	0	0.111
AK058583	CG	HH	9346348	+	749	1	P	1764.282	0.294	0
Chr10_1347	UG	LH	9363298	+	339	1	P	2528.474	0.625	0
Chr10_1348	EG	HH	9375775	+	492	1	P	1037.019	0.25	0
Chr10_1349	UG	HH	9377124	+	2828	3	P	2045.904	0.161	0.083
Chr10_1350	EG	HH	9382411	-	10749	15	P	4567.053	0.138	0.088
Chr10_1352	UG	LH	9417226	-	1895	3	P	3572.268	0.235	0
AK108642	CG	LH	9455931	+	699	4	P	1350.848	0.167	0

Chr10_1355	UG	LH	9457527	-	438	1	P	3014.453	0.455	0
Chr10_1356	UG	LH	9459538	-	273	1	A	562.316	0.143	0
AK066204	CG	HH	9462514	+	4584	8	P	2788.645	0.097	0.036
AK058817	CG	HH	9498301	+	1250	3	P	1198.804	0.321	0
Chr10_1360	UG	HH	9504333	+	1029	1	P	1159.417	0.261	0
Chr10_1361	UG	HH	9514606	-	612	2	P	1773.883	0.429	0.143
Chr10_1362	EG	HH	9517061	+	1428	1	P	2135.061	0.25	0
Chr10_1363	EG	HH	9520518	-	9025	7	P	1945.606	0.065	0.014
Chr10_1364	UG	HH	9529992	-	2189	6	P	3242.522	0.235	0.129
Chr10_1365	UG	HH	9551769	-	5063	5	P	1706.203	0.182	0.023
Chr10_1366	UG	LH	9560820	+	1258	2	P	3435.401	0.238	0.143
Chr10_1367	UG	HH	9567286	+	2533	3	P	1616.915	0.044	0
AK109451	CG	LH	9572225	+	31058	13	P	2526.272	0.125	0.13
AK106942	CG	LH	9577306	-	1510	4	P	1192.735	0.147	0
Chr10_1369	EG	LH	9579276	+	667	2	P	3971.003	0.4	0.4
Chr10_1370	EG	LH	9581337	-	3906	6	P	1787.699	0.15	0.015
Chr10_1371	UG	HH	9587211	+	318	1	P	13647.526	0.429	0
Chr10_1372	UG	LH	9593370	+	2741	5	P	2407.843	0.205	0
Chr10_1373	UG	LH	9598066	-	372	2	P	1412.826	0.667	0
Chr10_1374	UG	LH	9601060	+	1377	3	P	2912.968	0.167	0.227
AK100083	CG	LH	9605348	+	2721	5	P	3866.487	0.091	0.167
Chr10_1376	UG	LH	9609747	-	2610	6	P	2521.324	0.1	0
Chr10_1377	UG	HH	9619739	-	715	2	A	0	0	0.5
Chr10_1379	UG	HH	9631207	-	5642	13	P	995.729	0.058	0.053
Chr10_1381	UG	HH	9645302	-	483	1	P	2647.692	0.182	0
AK073721	CG	LH	9655222	+	3614	7	P	1009.758	0.105	0.024
Chr10_1384	UG	LH	9658732	-	1680	5	P	847.109	0.059	0.05
Chr10_1386	UG	LH	9670110	-	1039	2	P	4199.101	0.667	1
Chr10_1387	UG	LH	9672320	-	225	1	P	2437.119	0.333	0
Chr10_1391	UG	LH	9698819	+	156	1	P	4122.359	0.75	0
Chr10_1400	UG	LH	9751159	+	282	1	P	5420.417	0.429	0
Chr10_1401	UG	LH	9753132	+	2214	2	P	3470.051	0.15	0.091
Chr10_1402	UG	HH	9758694	+	1116	3	P	3102.238	0.125	0.143
Chr10_1404	UG	HH	9767240	+	1848	2	P	2162.047	0.125	0
AK105198	CG	HH	9771303	+	1310	3	P	930.37	0.182	0
Chr10_1406	UG	LH	9773662	-	459	1	P	6473.15	0.364	0
Chr10_1407	EG	HH	9777002	-	2590	4	P	2889.535	0.333	0.089
Chr10_1408	UG	LH	9782032	+	1375	2	P	2849.771	0.133	0.125
Chr10_1409	UG	LH	9788828	-	3935	5	P	1435.423	0.043	0.148
Chr10_1410	UG	HH	9798392	-	5174	10	P	1628.002	0.184	0.031
Chr10_1412	UG	LH	9819997	-	693	1	P	2351.255	0.375	0
Chr10_1413	UG	LH	9822348	+	606	1	P	948.143	0.214	0
AK072810	CG	HH	9827239	-	16434	7	P	1296	0.123	0.132
Chr10_1415	UG	HH	9832413	-	3988	4	P	2552.498	0.222	0.118
Chr10_1417	UG	LH	9847177	+	1708	4	P	1503.075	0.167	0.16
Chr10_1418	UG	LH	9852304	+	1227	3	P	1735.396	0.28	0
AK071370	CG	LH	9854958	-	3311	7	P	1361.553	0.105	0
Chr10_1420	UG	LH	9860880	+	537	1	P	885.551	0.231	0
AK058352	CG	HH	9865459	-	1925	3	P	2902.867	0.125	0
Chr10_1422	UG	HH	9877261	+	2268	6	P	2865.86	0.316	0.107
Chr10_1423	UG	LH	9886974	-	834	2	P	1809.683	1	0.615
Chr10_1424	UG	LH	9890610	+	312	2	P	5123.314	0.667	0
AK069323	CG	HH	9894286	+	5069	11	P	2055.172	0.208	0.071
AK100049	CG	HH	9899528	-	4829	11	P	2526.042	0.234	0.036
AK100740	CG	HH	9923683	-	9567	7	P	1446.821	0.081	0.029
Chr10_1428	UG	LH	9926620	+	1103	3	P	2249.664	0.286	0.143
AK067524	CG	HH	9936520	+	3995	6	P	1359.069	0.082	0
AK065000	CG	LH	9940723	-	6688	7	P	1961.818	0.14	0.021
AK062606	CG	LH	9946802	+	717	2	P	7160.327	0.071	0
Chr10_1433	UG	LH	9949626	-	1952	3	P	4219.848	0.333	0.194
AK065895	CG	HH	9952687	-	3795	7	P	1904.485	0.174	0
AK060558	CG	LH	9960442	-	5246	11	P	1558.191	0.203	0.056

Chr10_1436	UG	LH	9977432	-	1733	2	A	313.564	0.111	0.31
Chr10_1437	UG	HH	9983191	-	5311	6	P	987.806	0.125	0.037
Chr10_1441	UG	LH	10010252	-	2519	2	P	13592.718	0.6	0.143
Chr10_1442	UG	LH	10019980	-	4786	9	P	1160.246	0.133	0.019
AK070069	CG	HH	10026546	+	2713	2	P	1506.218	0.421	0.158
Chr10_1444	UG	HH	10033219	+	1843	4	P	1444.487	0.192	0
Chr10_1445	UG	HH	10035627	-	1414	3	P	1616.856	0.08	0
Chr10_1446	UG	HH	10039126	+	966	1	P	2626.847	0.238	0
Chr10_1447	UG	LH	10046001	+	1619	3	P	1818.029	0.273	0.2
Chr10_1448	UG	LH	10049322	-	1113	2	P	1741.599	0.6	0.2
Chr10_1449	UG	LH	10061094	+	1804	4	P	1502.853	0.188	0.261
Chr10_1450	UG	LH	10070542	+	711	1	P	1458.545	0.313	0
Chr10_1451	UG	LH	10073842	+	841	2	P	2145.305	0.533	0
Chr10_1452	UG	LH	10075876	-	2365	6	A	618.449	0.091	0
Chr10_1453	UG	HH	10082968	+	1293	2	P	2225.391	0.231	0
Chr10_1454	UG	LH	10086999	-	1573	3	P	3352.76	0.313	0.188
Chr10_1455	UG	LH	10091393	+	2297	3	P	1573.757	0.143	0
AK064552	CG	LH	10096051	-	4203	10	P	972.024	0.109	0.043
Chr10_1457	UG	LH	10110108	+	1546	4	P	1162.832	0.143	0.077
AK108902	CG	LH	10113545	+	969	2	P	2524.852	0.286	0
Chr10_1459	UG	LH	10117339	+	1405	3	P	1384.556	0.333	0.1
Chr10_1460	UG	LH	10129554	+	1084	2	P	1844.096	0.273	0.077
Chr10_1462	UG	HH	10148686	-	1186	3	A	0	0	0
Chr10_1463	UG	HH	10151128	+	4802	6	A	542.908	0.043	0.13
Chr10_1465	UG	LH	10161679	-	288	1	P	1417.921	0.286	0
Chr10_1466	UG	LH	10162524	+	279	1	P	2622.784	0.857	0
Chr10_1467	UG	LH	10165929	+	1593	3	P	4100.317	0.308	0.13
Chr10_1468	UG	LH	10170322	-	1422	1	P	3303.763	0.226	0
Chr10_1469	UG	HH	10175337	-	1139	4	P	1001.662	0.118	0
Chr10_1471	UG	LH	10186158	+	204	1	P	1083.08	0.2	0
AK067540	CG	HH	10189161	-	14289	6	P	2068.687	0.045	0.102
Chr10_1472	UG	HH	10189382	+	3268	2	P	2224.617	0.028	0
Chr10_1475	UG	HH	10215063	+	3212	3	P	1437.104	0.056	0
Chr10_1476	UG	LH	10218782	-	1525	5	P	4840.277	0.2	0.182
Chr10_1477	UG	LH	10222757	+	231	1	P	4814.071	0.333	0
Chr10_1478	UG	HH	10228472	+	632	2	P	717.579	0.077	0
Chr10_1479	UG	HH	10235342	+	4294	2	P	2147.734	0.186	0.22
AK099935	CG	LH	10247718	-	3201	13	P	2071.835	0.133	0.125
AK105100	CG	LH	10249683	-	367	3	P	1956.724	0.1	0
Chr10_1481	UG	LH	10250505	+	315	1	P	1711.484	0.25	0
Chr10_1484	UG	LH	10269284	-	257	2	P	2752.661	0.143	0
AK101479	CG	HH	10271097	+	7214	16	P	1444.423	0.195	0.071
AK068318	CG	HH	10280893	+	3235	5	A	527.922	0.056	0.075
Chr10_1487	EG	HH	10324325	+	1997	4	P	3107.936	0.2	0
Chr10_1491	UG	LH	10342897	+	1529	2	P	2387.644	0.5	0.3
Chr10_1492	UG	HH	10347420	-	1457	2	P	1891.906	0.13	0
Chr10_1493	UG	LH	10358471	-	463	2	P	3944.067	0.3	0
Chr10_1494	EG	HH	10368045	+	525	1	P	2865.898	0.417	0
Chr10_1495	UG	HH	10370726	+	1005	1	P	6295.016	0.091	0
Chr10_1496	UG	LH	10374984	-	462	1	A	0	0	0
Chr10_1497	UG	LH	10377999	-	210	1	P	1113.984	0.6	0
Chr10_1498	UG	LH	10381614	-	279	1	P	4890.074	0.571	0
Chr10_1499	UG	LH	10385924	-	3531	3	P	3600.556	0.13	0.212
AK058809	CG	HH	10395837	+	4575	4	P	3043.431	0.25	0.094
Chr10_1502	UG	LH	10402994	+	2216	3	P	2606.598	0.385	0.235
AK111349	CG	LH	10412675	+	2681	6	P	5365.273	0.167	0.125
Chr10_1504	UG	LH	10415155	-	603	2	P	6870.565	0.667	0.333
Chr10_1505	UG	LH	10419706	+	500	2	P	4481.67	0.5	0.286
Chr10_1506	EG	HH	10433142	+	3793	4	P	2522.937	0.273	0.026
AK104132	CG	HH	10442150	-	4738	4	P	2825.878	0.464	0.093
Chr10_1508	UG	HH	10449658	+	8321	15	P	1598.566	0.085	0.093
AK099380	CG	HH	10460902	-	5707	16	P	2748.283	0.2	0.072

Chr10_1512	UG	LH	10484135	-	4698	6	A	582.065	0.071	0.052
Chr10_1513	UG	HH	10493196	+	3243	3	A	0	0	0
Chr10_1515	UG	HH	10501203	-	1285	3	P	2887.82	0.167	0
Chr10_1516	EG	LH	10504919	+	2243	4	P	777.174	0.154	0.114
Chr10_1517	EG	HH	10507784	-	5426	7	P	2517.97	0.171	0.048
AK059907	CG	HH	10520475	+	3814	6	P	2032.669	0.167	0.055
AK059720	CG	HH	10524951	-	2697	4	P	3962.255	0.103	0.091
Chr10_1521	UG	LH	10530913	-	588	1	P	4585.558	0.538	0
AK102892	CG	LH	10533351	-	2706	2	P	763.371	0.075	0
Chr10_1524	UG	LH	10541967	+	1516	3	P	3209.318	0.176	0.438
Chr10_1525	UG	LH	10552485	+	1134	3	A	631.562	0.15	0
Chr10_1526	UG	HH	10555176	-	1507	4	P	1185.487	0.042	0
Chr10_1527	UG	LH	10558365	-	1050	2	P	4424.463	0.3	0.077
Chr10_1528	UG	HH	10573777	-	3472	3	A	304.272	0.111	0.071
Chr10_1529	UG	LH	10602226	+	581	2	P	4353.059	0.333	0
Chr10_1530	EG	HH	10611987	+	1402	2	P	1001.356	0.133	0
Chr10_1531	UG	LH	10622412	+	687	2	P	5095.311	0.286	0
Chr10_1533	UG	HH	10647762	+	429	1	P	4030.993	0.2	0
Chr10_1534	UG	HH	10649863	+	342	1	P	4387.089	0.375	0
Chr10_1535	UG	HH	10652438	+	381	1	P	2021.827	0.222	0
Chr10_1536	UG	LH	10654631	+	666	3	P	2046.536	0.4	0.4
Chr10_1539	UG	HH	10670649	+	342	1	P	3901.867	0.375	0
Chr10_1540	UG	LH	10673552	+	342	1	P	4339.037	0.222	0
Chr10_1542	UG	HH	10684769	-	1580	2	P	2374.665	0.182	0
Chr10_1543	UG	LH	10687509	-	1130	2	A	0	0	0
Chr10_1545	UG	LH	10705336	+	833	2	P	1168.896	0.6	0.231
Chr10_1549	UG	LH	10720747	-	809	2	P	1176.932	0.25	0.1
Chr10_1550	UG	HH	10730021	+	3802	6	P	2934.68	0.154	0
AK060203	CG	HH	10735224	-	2299	5	P	2765.449	0.182	0
Chr10_1552	UG	HH	10739631	+	5693	4	P	2109.945	0.179	0.203
Chr10_1553	UG	HH	10747944	+	1070	2	P	2734.924	0.217	0
Chr10_1554	UG	LH	10751183	+	1423	2	A	0	0	0.222
Chr10_1555	UG	LH	10755781	+	868	2	P	4694.921	0.176	0.667
AK069060	CG	HH	10758520	+	9133	12	P	2178.8	0.172	0.035
Chr10_1557	UG	LH	10768817	-	846	2	P	836.248	0.2	0.167
AK064476	CG	LH	10771943	-	4811	10	P	938.625	0.15	0.113
AK106391	CG	LH	10779705	-	1895	4	P	2344.729	0.219	0
Chr10_1561	UG	LH	10796658	-	9130	10	P	808.816	0.08	0.01
Chr10_1563	UG	LH	10816427	-	1995	3	P	815.302	0.176	0.059
Chr10_1564	UG	LH	10820634	+	1241	2	P	1301.083	0.24	0
Chr10_1566	UG	LH	10827608	-	1926	5	P	1177.853	0.077	0
Chr10_1568	UG	LH	10838013	+	738	2	A	647.462	0.067	0
Chr10_1569	UG	LH	10841090	+	504	1	A	0	0	0
Chr10_1572	UG	HH	10850398	-	2679	6	P	1536.909	0.067	0.125
Chr10_1577	UG	LH	10869754	-	1272	2	A	658.551	0.13	0
Chr10_1579	UG	LH	10882345	+	2394	5	P	1121.242	0.125	0
Chr10_1581	UG	LH	10889688	-	2463	6	P	2509.465	0.074	0.154
Chr10_1582	UG	LH	10895384	+	687	2	A	0	0	0
Chr10_1583	UG	HH	10897463	+	1805	5	P	743.921	0.031	0
Chr10_1584	UG	LH	10899511	-	1089	2	P	2976.609	0.067	0
Chr10_1585	UG	LH	10903217	+	3257	4	P	3102.326	0.25	0.153
Chr10_1586	UG	HH	10910913	+	9525	9	P	1582.389	0.105	0.029
Chr10_1588	UG	LH	10931287	+	1611	2	A	604.065	0.111	0.04
AK071078	CG	LH	10933318	-	30028	6	P	1404.983	0.111	0.133
Chr10_1592	UG	LH	10950036	-	1308	2	P	2238.688	0.333	0.318
Chr10_1593	UG	LH	10953765	-	1028	3	P	1324.26	0.364	0.167
Chr10_1594	UG	HH	10962085	+	6564	9	P	2859.124	0.149	0.184
Chr10_1595	UG	LH	10972449	-	2591	5	A	0	0	0.119
Chr10_1597	UG	LH	10982565	+	2590	6	P	731.451	0.2	0
Chr10_1599	UG	HH	11002093	-	927	3	P	1161.259	0.211	0
Chr10_1600	UG	LH	11007691	-	4971	6	P	2038.689	0.233	0.132
Chr10_1604	UG	LH	11047201	+	2880	3	P	4926.391	0.167	0.078

AK099943	CG	HH	11050924	-	4141	11	P	737.864	0.292	0.023
Chr10_1606	UG	HH	11067103	-	904	2	P	946.699	0.053	0
Chr10_1607	UG	HH	11068353	-	1486	4	A	664.246	0.04	0
Chr10_1608	UG	LH	11070183	-	574	2	A	0	0	0
Chr10_1609	UG	LH	11074920	-	1580	2	A	0	0	0.08
Chr10_1610	UG	LH	11078867	-	2030	4	A	0	0	0.029
Chr10_1611	UG	HH	11082950	+	872	2	A	405.175	0.2	0
Chr10_1612	UG	LH	11084212	-	471	1	P	8846.605	0.364	0
Chr10_1613	UG	LH	11085159	-	4889	4	P	1673.38	0.087	0.16
Chr10_1614	UG	HH	11090503	-	7458	6	P	1795.829	0.13	0.411
Chr10_1615	UG	LH	11103609	+	2088	4	A	0	0	0.091
Chr10_1616	EG	LH	11114909	+	1440	4	A	370.849	0.118	0.071
Chr10_1617	UG	HH	11117740	-	1062	2	A	609.013	0.111	0.25
Chr10_1618	UG	HH	11119516	+	1398	3	A	468.442	0.063	0
Chr10_1619	UG	LH	11125989	+	1123	2	A	0	0	0.059
Chr10_1620	UG	LH	11128215	-	570	1	P	6479.041	0.615	0
Chr10_1621	UG	LH	11131380	-	1018	4	A	0	0	0.083
Chr10_1622	UG	HH	11146672	+	9057	13	P	924.923	0.07	0.103
Chr10_1625	UG	LH	11179742	-	483	1	P	1630.311	0.182	0
Chr10_1626	UG	HH	11182849	+	2019	3	P	2247.292	0.2	0.133
AK101651	CG	HH	11192939	-	1968	8	A	475.345	0.107	0.176
AK065782	CG	HH	11195894	-	3129	9	P	1648.317	0.207	0.098
Chr10_1629	UG	HH	11209144	-	3411	4	P	3376.42	0.333	0.051
Chr10_1630	EG	HH	11217375	-	1879	3	P	2671.786	0.182	0
Chr10_1631	EG	LH	11226770	+	4459	14	P	609.33	0.25	0.081
Chr10_1632	UG	HH	11231937	-	7186	8	P	1637.195	0.115	0.038
Chr10_1633	UG	HH	11239641	-	2320	2	P	2904.493	0.368	0.032
AK066066	CG	LH	11246476	-	2787	4	P	2575.7	0.265	0
Chr10_1636	UG	LH	11250734	+	2134	5	A	603.39	0.121	0.071
Chr10_1637	EG	HH	11263878	-	4888	15	P	1340.648	0.123	0.06
Chr10_1638	UG	LH	11278455	-	1727	5	P	1359.922	0.056	0.053
Chr10_1639	EG	HH	11281462	-	4751	11	P	5619.242	0.046	0.051
Chr10_1640	EG	LH	11291618	-	1181	3	A	0	0	0
Chr10_1641	UG	LH	11297193	+	442	2	P	3440.123	0.182	0
AK102488	CG	LH	11306326	+	4549	12	P	1307.592	0.371	0.108
AK107029	CG	HH	11317348	+	5047	17	P	1797.927	0.149	0.063
Chr10_1644	EG	LH	11323197	+	495	3	P	3668.798	0.182	0
Chr10_1645	UG	HH	11329217	+	1370	5	A	0	0	0.333
Chr10_1646	UG	LH	11335990	-	303	1	P	912.326	0.25	0
AK110269	CG	LH	11338695	+	1357	3	P	1308.432	0.2	0
Chr10_1648	EG	HH	11344649	-	2896	7	A	418.209	0.231	0.056
AK063378	CG	LH	11358685	+	2081	4	P	1800.54	0.205	0
Chr10_1651	EG	HH	11363120	-	1479	1	P	3367.548	0.455	0
Chr10_1652	EG	LH	11365553	-	4298	8	P	2932.694	0.189	0.098
AK107854	CG	LH	11377826	-	827	1	P	1333.534	0.421	0
AK100298	CG	LH	11382755	-	4546	5	P	1880.573	0.17	0.083
Chr10_1654	UG	LH	11389206	-	606	1	P	3167.269	0.5	0
Chr10_1655	UG	LH	11391255	-	4077	3	P	3198.244	0.591	0.065
Chr10_1656	UG	LH	11400222	-	1713	4	A	0	0	0.217
AK061602	CG	LH	11417675	-	1126	3	P	2407.913	0.364	0
Chr10_1660	UG	LH	11430270	+	1049	2	P	3865.763	0.583	0
AK108539	CG	HH	11436020	+	2234	3	P	2790.315	0.269	0.053
AK068830	CG	HH	11438311	-	5599	10	P	2221.701	0.1	0.016
Chr10_1664	UG	HH	11451951	+	3576	5	P	1502.844	0.172	0
AK072853	CG	HH	11459272	-	2478	6	P	2163.302	0.167	0
Chr10_1666	UG	HH	11468540	+	492	2	P	2373.702	0.222	0
Chr10_1667	UG	HH	11474755	+	6174	5	P	1962.806	0.179	0.14
AK108245	CG	HH	11484831	+	1895	4	P	2960.24	0.265	0
Chr10_1669	UG	LH	11490628	+	485	2	A	387.617	0.2	0.333
AK110763	CG	HH	11495808	+	2233	5	P	1527.241	0.343	0
AK066208	CG	HH	11502413	-	5100	11	P	3339.551	0.154	0.035
Chr10_1672	UG	HH	11513822	+	3183	2	P	3559.546	0.192	0

Chr10_1673	UG	LH	11518693	-	249	1	A	404.295	0.167	0
AK100807	CG	HH	11524731	+	12334	4	P	1667.181	0.128	0.207
Chr10_1677	UG	LH	11550729	-	1413	3	P	4546.467	0.182	0.333
Chr10_1678	UG	LH	11553157	+	691	2	P	6339.178	0.5	0.25
Chr10_1679	UG	LH	11557422	-	3410	3	P	866.747	0.267	0.107
Chr10_1680	UG	LH	11562972	+	1062	2	A	360.411	0.143	0.063
Chr10_1681	UG	LH	11565304	-	228	1	P	5151.415	0.667	0
Chr10_1682	EG	HH	11568290	-	5894	5	P	2256.586	0.259	0.049
Chr10_1683	EG	HH	11579477	-	23499	34	P	1585.805	0.072	0.043
Chr10_1684	UG	LH	11603829	+	691	2	P	4140.001	0.111	0
AK067046	CG	LH	11606376	-	1063	2	P	3110.904	0.35	0
Chr10_1686	EG	HH	11614040	-	1655	2	P	7506.966	0.235	0
Chr10_1687	EG	LH	11617230	+	1991	7	P	862.966	0.133	0
Chr10_1688	UG	LH	11619744	-	1927	2	P	2236.575	0.265	0.375
Chr10_1689	UG	LH	11624447	-	629	2	P	2646.64	0.385	0
Chr10_1690	UG	LH	11630196	-	492	1	P	2111.06	0.182	0
Chr10_1691	UG	LH	11631461	-	799	2	P	1845.933	0.333	0
AK064116	CG	LH	11636223	-	1422	1	P	1212.648	0.226	0
Chr10_1693	UG	LH	11638533	+	2627	6	P	2989.381	0.353	0.4
AK070796	CG	HH	11646333	-	5628	13	P	3444.217	0.048	0.035
Chr10_1695	UG	LH	11675739	-	1195	3	A	0	0	0.105
Chr10_1696	UG	HH	11677179	-	264	1	A	705.219	0.167	0
AK102029	CG	HH	11693111	-	4270	5	P	2427.419	0.226	0.033
Chr10_1701	EG	LH	11699923	-	1937	2	P	1931.893	0.135	0
Chr10_1702	UG	LH	11718530	+	1005	1	P	1389.403	0.409	0
Chr10_1703	UG	LH	11725519	+	1110	3	P	1305.617	0.333	0.056
Chr10_1704	EG	HH	11728226	-	1685	3	P	1801.105	0.375	0
Chr10_1705	UG	LH	11731425	-	1469	2	A	771.163	0.167	0.08
Chr10_1706	EG	LH	11734669	+	422	3	A	0	0	0
Chr10_1707	UG	HH	11735879	-	2328	4	A	759.686	0.08	0.08
Chr10_1708	UG	HH	11740381	+	773	3	P	1963.632	0.2	0
Chr10_1709	UG	HH	11747889	-	4233	3	P	1094.954	0.06	0.024
Chr10_1710	UG	LH	11759332	-	1206	2	P	1392.402	0.222	0
Chr10_1711	UG	LH	11763863	+	471	2	P	3425.842	0.222	0
Chr10_1714	UG	HH	11778907	+	1754	2	P	2481.327	0.139	0
Chr10_1715	UG	LH	11780946	-	6031	6	P	3583.044	0.156	0.086
AK108922	CG	HH	11788911	-	916	2	P	4265.556	0.111	0
Chr10_1721	EG	HH	11808381	-	486	1	P	1645.791	0.083	0
Chr10_1722	UG	LH	11812379	-	1570	3	P	1857.861	0.267	0.15
Chr10_1723	UG	LH	11816030	-	1201	3	P	1739.383	0.067	0.083
Chr10_1724	UG	HH	11822500	+	2210	2	P	2897.376	0.167	0.308
Chr10_1725	UG	HH	11825272	+	2610	4	P	4437.85	0.156	0
Chr10_1726	UG	LH	11831897	+	540	2	P	1864.644	0.333	0.143
Chr10_1728	UG	HH	11841335	-	1724	4	P	2174.082	0.2	0.143
Chr10_1729	UG	LH	11844658	+	378	1	P	3538.327	0.444	0
Chr10_1730	EG	HH	11849753	+	1770	4	P	1810.673	0.235	0
Chr10_1731	UG	HH	11854958	+	4200	4	P	1277.634	0.198	0.5
AK067274	CG	HH	11874381	+	3970	14	P	2277.921	0.121	0.103
AK107314	CG	HH	11878942	-	2039	4	P	6965.996	0.875	0
Chr10_1735	UG	HH	11883425	-	1956	1	P	2967.585	0.488	0
Chr10_1739	UG	LH	11904476	+	276	1	P	5321.493	0.714	0
Chr10_1742	UG	LH	11921278	+	357	1	A	0	0	0
Chr10_1743	UG	HH	11926111	-	1509	1	P	1843.166	0.375	0
AK072513	CG	LH	11936860	-	1040	3	P	1982.894	0.16	0
AK068126	CG	HH	11939257	-	4892	18	P	1408.668	0.211	0.04
AK072620	CG	HH	11945146	+	8779	21	P	998.607	0.098	0.088
Chr10_1746	EG	LH	11954765	-	243	1	P	3606.438	0.667	0
Chr10_1747	UG	HH	11958843	+	4218	5	P	2925.209	0.182	0.068
Chr10_1748	UG	LH	11963945	+	802	2	P	4313.539	0.75	0.3
Chr10_1749	UG	LH	11965349	+	597	1	P	2808.605	0.231	0
Chr10_1750	UG	LH	11966212	-	974	2	P	2200.134	0.455	0.667
Chr10_1754	UG	LH	11991827	+	2107	3	P	3684.423	0.313	0.24

Chr10_1755	EG	LH	11994726	+	815	2	P	1702.931	0.214	0.2
Chr10_1756	UG	LH	11995831	-	2783	5	P	4036.209	0.435	0.086
Chr10_1757	UG	LH	11999320	+	1980	3	P	1766.658	0.185	0
Chr10_1759	UG	HH	12012846	-	5576	6	P	2182.573	0.214	0.205
Chr10_1761	EG	LH	12034084	-	2071	2	P	3664.28	0.333	0.087
Chr10_1763	UG	LH	12059507	+	521	2	P	3280.564	0.75	0.25
AK100194	CG	LH	12062313	-	47843	4	P	4178.207	0.286	0.122
Chr10_1765	UG	HH	12072482	-	934	2	P	940.42	0.368	0
Chr10_1766	UG	HH	12080553	-	1751	2	P	2097.383	0.324	0
Chr10_1767	UG	HH	12083924	-	3321	5	P	1302.886	0.206	0.2
Chr10_1768	UG	LH	12090175	+	348	2	P	1447.564	0.5	0.25
Chr10_1769	UG	LH	12094919	+	594	2	A	499.792	0.222	0
Chr10_1774	UG	LH	12133365	-	658	2	P	2989.615	0.545	0.25
Chr10_1775	EG	HH	12135584	+	5463	5	P	2510.135	0.16	0.033
Chr10_1776	UG	HH	12149532	-	378	2	P	1492.13	0.143	0
Chr10_1778	UG	LH	12166230	+	384	2	P	682.36	0.429	0
AK071452	CG	HH	12171274	+	9451	7	P	2070.741	0.111	0.057
Chr10_1780	UG	LH	12183747	+	690	2	P	1601.238	0.417	0.667
Chr10_1781	UG	LH	12187916	+	2225	3	P	2096.858	0.122	0.25
Chr10_1782	UG	LH	12206503	+	1274	2	P	3636.665	0.583	0
Chr10_1783	UG	LH	12213660	-	1494	2	P	2459.324	0.292	0.111
AK100413	CG	HH	12225638	+	2949	3	P	2425.931	0.18	0
Chr10_1785	UG	HH	12232916	-	646	2	A	0	0	0
AK066928	CG	LH	12235310	+	2913	5	P	2605.626	0.609	0.093
Chr10_1787	UG	LH	12243265	+	7862	11	P	4555.621	0.162	0.071
AK065250	CG	HH	12251528	-	4397	14	P	3258.852	0.05	0.028
Chr10_1789	UG	LH	12266751	+	684	1	P	4838.315	0.2	0
AK072672	CG	HH	12270895	-	2095	9	P	1553.324	0.156	0
Chr10_1791	EG	HH	12274174	-	4172	3	P	1309.173	0.138	0.082
AK068588	CG	HH	12293414	-	2651	5	P	2547.379	0.303	0.36
Chr10_1793	EG	HH	12307395	-	4106	9	A	465.754	0.083	0.019
Chr10_1794	EG	HH	12318020	-	273	1	P	8948.22	0.167	0
Chr10_1796	UG	LH	12330988	-	1212	1	P	2980.103	0.111	0
Chr10_1797	UG	LH	12334478	-	1233	1	A	371.962	0.111	0
Chr10_1798	EG	LH	12339312	+	2865	7	P	1912.991	0.273	0.049
Chr10_1799	UG	LH	12344093	-	2145	5	P	3606.977	0.27	0.182
AK065200	CG	HH	12347334	+	5668	9	P	1649.094	0.222	0.068
Chr10_1801	UG	LH	12355729	+	1217	2	P	3878.162	0.154	0
AK101494	CG	HH	12360312	-	2321	6	P	1647.547	0.367	0.19
AK070968	CG	LH	12364963	-	1994	3	P	856.26	0.25	0.036
AK101309	CG	LH	12367436	-	1057	4	P	1047.26	0.318	0
Chr10_1805	UG	LH	12369491	+	386	2	P	3278.443	0.125	0
Chr10_1806	UG	HH	12376254	-	7742	16	P	2043.262	0.137	0.116
Chr10_1807	UG	HH	12391120	+	5120	7	P	2730.388	0.094	0.05
Chr10_1808	EG	HH	12399261	+	3390	6	P	1530.633	0.2	0.026
Chr10_1809	UG	LH	12408904	+	1361	3	A	0	0	0
Chr10_1813	UG	LH	12488788	+	840	2	A	0	0	0
Chr10_1814	UG	LH	12492396	+	634	2	P	3281.844	0.429	0
Chr10_1815	UG	LH	12495277	+	1182	3	A	0	0	0.053
AK068671	CG	HH	12521584	-	7730	12	P	1812.844	0.154	0.167
Chr10_1817	UG	LH	12525912	-	1062	2	P	7602.336	0.4	0.474
AK072572	CG	HH	12556737	+	3925	10	P	1041.067	0.111	0.087
AK108669	CG	HH	12557222	-	1647	2	P	1067.8	0.056	0
AK058209	CG	HH	12560888	-	1276	2	P	1977.478	0.714	0.133
AK059434	CG	HH	12568004	+	742	2	P	3308.772	0.588	0
Chr10_1822	UG	LH	12569849	-	798	2	A	0	0	0.125
Chr10_1823	UG	LH	12571329	-	2813	5	A	355.268	0.071	0
Chr10_1824	EG	LH	12574774	+	1948	4	A	0	0	0
Chr10_1825	UG	LH	12578003	-	333	1	P	2975.133	0.625	0
Chr10_1826	UG	LH	12584788	-	1118	3	P	4678.558	0.053	0
AK066864	CG	HH	12588671	-	4412	12	P	1486.741	0.068	0
AK102338	CG	HH	12603977	-	8406	13	P	2385.227	0.203	0.044

Chr10_1829	UG	LH	12619356	+	309	1	P	1926.574	0.429	0
AK101468	CG	LH	12621939	+	2746	5	P	1638.683	0.189	0.1
Chr10_1830	UG	HH	12631719	+	598	2	P	922.387	0.091	0
Chr10_1831	UG	LH	12633747	-	2033	5	P	1874.782	0.1	0.2
Chr10_1833	UG	LH	12644720	+	544	4	A	0	0	0
Chr10_1834	UG	LH	12645849	-	708	2	P	3362.802	0.429	0.444
AK110572	CG	HH	12650275	-	1508	3	P	2374.657	0.25	0.1
AK070592	CG	LH	12654125	+	4131	9	P	1914.931	0.182	0.037
Chr10_1837	UG	LH	12660409	-	231	1	P	4401.007	0.8	0
Chr10_1838	UG	LH	12672363	+	700	2	P	3101.926	0.313	0
Chr10_1839	UG	LH	12674208	+	334	2	P	5185.599	0.333	0
Chr10_1840	UG	HH	12675277	+	1748	2	P	4076.289	0.25	0
Chr10_1841	UG	HH	12678595	+	474	1	P	2725.771	0.364	0
AK061548	CG	HH	12679619	-	3855	7	P	1517.985	0.194	0.038
Chr10_1843	UG	LH	12685535	-	762	1	P	3579.203	0.176	0
AK109904	CG	LH	12687948	+	27213	7	P	1970.251	0.143	0.141
Chr10_1845	UG	LH	12692976	+	495	2	A	519.41	0.4	0
Chr10_1846	UG	LH	12703368	+	1987	3	A	0	0	0.179
Chr10_1847	UG	HH	12706624	+	1035	1	P	3713.938	0.043	0
Chr10_1848	UG	LH	12708151	+	1216	2	P	3866.67	0.364	0.438
Chr10_1850	UG	HH	12718084	-	1064	2	P	1084.825	0.043	0
AK110942	CG	LH	12720431	+	1722	6	P	2392.772	0.162	0
Chr10_1852	UG	LH	12723346	+	1904	3	P	1798.832	0.211	0.5
Chr10_1853	UG	LH	12731358	+	438	1	P	2838.775	0.111	0
Chr10_1854	UG	LH	12739593	-	483	1	P	2006.069	0.455	0
Chr10_1855	EG	LH	12745662	+	408	1	P	1952.78	0.4	0
Chr10_1856	UG	LH	12746763	-	555	2	A	0	0	0.125
AK067754	CG	HH	12751260	-	3995	10	P	914.802	0.093	0.089
AK064079	CG	HH	12765651	+	17020	7	P	1480.723	0.167	0.098
AK063266	CG	HH	12769118	+	5997	7	P	1268.48	0.111	0.093
AK100246	CG	HH	12775290	+	3468	4	P	3052.116	0.148	0
AK068567	CG	HH	12782805	-	3387	2	P	2625.504	0.123	0.125
AK108799	CG	HH	12787698	-	3078	10	A	690.774	0.071	0
AK069676	CG	LH	12798748	-	7123	5	P	1029.813	0.231	0.054
AK070876	CG	HH	12818211	+	10248	20	P	1110.848	0.158	0.083
AK066318	CG	HH	12828899	+	5411	20	P	4084.94	0.068	0.043
Chr10_1865	EG	HH	12834696	-	6034	13	P	2509.279	0.143	0.039
AK071453	CG	LH	12844270	+	1211	2	P	1474.523	0.214	0
AK073016	CG	HH	12845930	-	1031	2	P	2103.158	0.174	0
Chr10_1868	EG	HH	12850087	-	747	1	P	835.531	0.235	0
Chr10_1869	UG	LH	12862961	+	345	1	A	307.206	0.222	0
AK059767	CG	LH	12874472	+	1069	1	A	0	0	0
Chr10_1871	EG	HH	12877305	+	2767	7	P	1569.531	0.118	0.125
Chr10_1872	UG	LH	12881619	+	742	2	P	1286.543	0.125	0
Chr10_1873	UG	HH	12882989	-	2878	3	P	3748.788	0.375	0.118
Chr10_1874	UG	LH	12902669	+	270	1	A	379.194	0.333	0
AK060033	CG	LH	12904086	+	1134	1	P	786.135	0.28	0
Chr10_1876	UG	LH	12906749	-	1891	2	P	4469.115	0.2	0
Chr10_1878	UG	LH	12924127	-	2472	4	P	2568.569	0.333	0
Chr10_1879	UG	LH	12928344	+	333	1	P	2599.772	0.625	0
Chr10_1880	UG	HH	12935656	+	3589	6	P	1297.066	0.078	0.043
Chr10_1881	UG	LH	12940308	-	314	2	P	2169.69	0.8	0
AK069385	CG	HH	12946058	+	7033	10	P	2209.242	0.268	0.133
AK067822	CG	HH	12974302	-	1653	4	P	2211.785	0.457	0
Chr10_1884	EG	HH	12995205	+	790	2	P	4060.97	0.364	0.286
AK109037	CG	HH	12998913	-	1500	2	P	2920.989	0.206	0
Chr10_1886	UG	HH	13004095	-	1314	1	P	2177.167	0.133	0
Chr10_1887	UG	LH	13007580	-	1495	2	P	1387.794	0.25	0.294
Chr10_1888	UG	HH	13011024	-	1255	2	P	2198.051	0.077	0
Chr10_1889	UG	LH	13029946	-	501	1	P	2531.539	0.167	0
Chr10_1890	UG	LH	13040037	+	1877	5	P	5740.313	0.5	0.125
Chr10_1891	UG	LH	13044689	+	1807	2	P	2841.315	0.444	0.172

AK063081	CG	LH	13055097	-	943	4	A	0	0	0.1
AK064344	CG	HH	13063094	+	5340	8	P	2445.417	0.114	0.104
Chr10_1894	UG	HH	13071730	-	2909	3	P	3303.912	0.357	0.032
Chr10_1895	EG	HH	13078949	-	2490	2	P	1545.153	0.32	0.034
AK103834	CG	HH	13087449	-	2341	8	P	934.746	0.346	0.12
AK071316	CG	HH	13092606	-	2629	6	P	1197.942	0.2	0.107
Chr10_1898	UG	HH	13100375	-	1340	3	P	1809.086	0.167	0
Chr10_1899	UG	LH	13104964	-	1457	3	P	3285.946	0.214	0
Chr10_1900	UG	LH	13111135	+	213	1	P	2744.824	0.6	0
Chr10_1901	UG	LH	13111729	-	3174	5	P	3046.411	0.267	0.02
AK106414	CG	HH	13115554	-	2506	6	P	3505.294	0.08	0.067
Chr10_1903	UG	HH	13125041	+	6015	6	P	1073.138	0.078	0.385
Chr10_1905	UG	LH	13134035	-	726	2	P	1750.765	0.091	0
AK110922	CG	LH	13139672	+	2071	4	P	2809.248	0.389	0.375
AK107671	CG	HH	13152625	+	2158	8	P	840.862	0.139	0
Chr10_1908	UG	HH	13157185	-	2097	2	P	1828.769	0.111	0
Chr10_1909	UG	LH	13162819	-	258	1	A	616.934	0.286	0
Chr10_1910	UG	LH	13179042	-	6865	8	P	1651.195	0.05	0.016
Chr10_1911	UG	HH	13186674	+	2244	3	P	1421.216	0.089	0
Chr10_1912	UG	LH	13195271	-	465	1	P	2409.909	0.273	0
Chr10_1913	UG	HH	13197695	-	2530	3	P	1395.077	0.157	0
Chr10_1914	UG	LH	13204027	+	4858	5	P	1613.884	0.182	0.033
Chr10_1915	UG	HH	13212096	+	2369	2	P	1512.706	0.12	0
Chr10_1916	UG	HH	13217215	+	2108	2	P	2425.559	0.13	0
Chr10_1918	UG	HH	13239282	-	1131	2	P	4700.12	0.059	0
Chr10_1919	UG	LH	13245426	-	2254	5	P	944.449	0.083	0
Chr10_1920	UG	LH	13248447	+	2580	3	P	1761.005	0.148	0
Chr10_1921	UG	LH	13254578	-	1888	2	P	778.936	0.154	0.174
Chr10_1922	UG	LH	13261121	+	411	1	A	395.575	0.222	0
Chr10_1923	EG	HH	13262375	-	10440	5	P	1640.38	0.194	0.079
AK063303	CG	HH	13273819	-	1941	2	P	963.575	0.056	0
Chr10_1925	UG	LH	13290085	+	252	1	P	1852.76	0.333	0
AK103743	CG	HH	13291893	+	3114	8	P	848.116	0.257	0.176
Chr10_1927	UG	HH	13296010	+	4230	7	P	2407.785	0.245	0.07
Chr10_1928	UG	HH	13306666	+	3612	10	P	1906.749	0.214	0.111
AK109893	CG	LH	13322640	-	2293	3	P	2244.166	0.111	0
Chr10_1930	UG	HH	13338940	-	6378	6	P	2204.228	0.137	0.048
AK109674	CG	LH	13358122	-	1734	4	P	1799.879	0.297	0
AK072418	CG	HH	13363382	+	793	1	P	2846.12	0.333	0
AK103624	CG	LH	13367029	-	2721	5	P	2673.683	0.3	0.3
Chr10_1933	UG	LH	13367568	+	1216	2	P	2420.591	0.375	0.091
Chr10_1934	UG	HH	13373820	+	1133	2	P	4351.09	0.083	0
Chr10_1935	UG	HH	13376570	-	1119	1	P	1675.703	0.154	0
Chr10_1936	UG	HH	13380396	+	1134	1	P	2119.442	0.24	0
Chr10_1937	UG	HH	13385131	+	1088	2	P	3583.888	0.208	0
Chr10_1938	UG	HH	13389162	+	1086	1	P	1445.438	0.208	0
Chr10_1939	UG	LH	13391981	-	1316	2	P	4336.718	0.364	0.053
Chr10_1940	UG	LH	13395762	+	597	2	P	5160.611	0.286	0
Chr10_1941	UG	HH	13397966	-	1197	1	P	2143.939	0.154	0
Chr10_1942	UG	HH	13404220	+	1125	1	P	2473.526	0.2	0
Chr10_1943	UG	LH	13412594	-	535	3	A	778.842	0.111	0
Chr10_1944	UG	HH	13414188	+	989	2	P	1224.601	0.227	0
Chr10_1946	UG	HH	13424729	+	858	1	A	458.109	0.2	0
Chr10_1948	UG	HH	13437124	+	1107	1	A	393.857	0.04	0
Chr10_1949	UG	LH	13440554	+	2387	3	P	1310.582	0.189	0.133
Chr10_1950	UG	LH	13447928	+	354	1	P	3113.031	0.625	0
Chr10_1952	UG	LH	13470675	+	381	1	P	3516.551	0.778	0
AK071408	CG	HH	13472149	+	1267	3	P	1503.443	0.233	0
Chr10_1954	UG	HH	13477870	+	2750	4	P	1014.279	0.25	0.125
Chr10_1955	UG	HH	13483302	+	1116	1	P	757.53	0.12	0
Chr10_1956	EG	HH	13485744	+	1068	1	P	1852.451	0.25	0
Chr10_1957	UG	HH	13490784	+	1247	2	P	2986.742	0.115	0

Chr10_1958	UG	HH	13496764	+	1236	1	P	986.308	0.222	0
Chr10_1959	UG	HH	13499832	+	951	1	P	1240.443	0.19	0
Chr10_1962	EG	HH	13518251	+	3151	2	P	1691.178	0.063	0
Chr10_1963	UG	LH	13524212	-	705	2	P	1723.348	0.333	0.222
Chr10_1964	UG	LH	13527962	+	231	1	P	4382.279	0.6	0
Chr10_1965	EG	HH	13534497	+	3604	5	P	1451.684	0.169	0
Chr10_1966	EG	LH	13539149	-	2018	6	P	6186.024	0.111	0.074
Chr10_1968	UG	HH	13552404	+	3439	2	P	1827.721	0.032	0
Chr10_1969	UG	HH	13561001	+	1501	2	P	2106.319	0.125	0
AK107342	CG	HH	13563147	+	1670	4	P	1506.615	0.176	0
Chr10_1971	UG	LH	13569580	+	1070	2	P	2368.962	0.417	0.083
Chr10_1972	UG	HH	13573267	-	1092	1	P	1382.623	0.25	0
Chr10_1973	UG	LH	13577105	+	492	1	A	0	0	0
Chr10_1974	UG	HH	13578320	+	432	1	P	3645.633	0.1	0
Chr10_1975	UG	HH	13581680	+	1106	2	P	2434.186	0.167	0.2
Chr10_1976	UG	HH	13584417	+	920	2	A	458.532	0.048	0
Chr10_1977	UG	LH	13585651	-	384	1	P	1123.108	0.333	0
Chr10_1978	UG	HH	13587587	+	1534	2	P	1516.97	0.12	0
Chr10_1979	UG	HH	13597508	-	1113	1	P	2768.675	0.16	0
Chr10_1980	UG	HH	13599648	-	1355	2	A	703.806	0.036	0
Chr10_1981	UG	HH	13601164	+	1101	3	P	1590.205	0.136	0
Chr10_1982	UG	LH	13603063	-	894	2	A	693.288	0.125	0
Chr10_1983	UG	HH	13604801	-	1183	2	P	1272.33	0.115	0
Chr10_1984	UG	LH	13613820	-	1518	2	P	6359.006	0.667	0.214
Chr10_1986	UG	HH	13622275	+	3747	8	P	1939.268	0.135	0.133
Chr10_1987	UG	HH	13633014	+	813	1	P	1659.853	0.333	0
Chr10_1992	UG	HH	13656643	+	2349	4	P	1214.61	0.321	0.045
AK109862	CG	HH	13661960	+	1439	1	A	391.585	0.031	0
Chr10_1994	UG	HH	13664456	+	1174	2	P	1435.104	0.273	0
Chr10_1995	UG	LH	13666352	-	1329	4	P	1543.081	0.364	0.118
Chr10_1996	UG	HH	13669287	+	968	2	P	1523.217	0.136	0
Chr10_1997	UG	HH	13673356	+	1095	1	P	1796.564	0.167	0
Chr10_1998	UG	HH	13680119	+	1086	1	P	713.507	0.2	0
Chr10_1999	UG	HH	13682192	-	555	1	P	2507.197	0.308	0
Chr10_2000	UG	HH	13684954	-	2522	4	P	5165.055	0.139	0.1
AK100213	CG	HH	13702626	+	1416	4	P	1685.938	0.156	0
Chr10_2006	UG	HH	13719622	+	2346	2	P	1159.384	0.188	0
Chr10_2007	UG	LH	13723164	+	672	1	P	3406.974	0.267	0
AK069040	CG	HH	13724467	-	4985	6	P	2572.977	0.205	0.097
AK069549	CG	LH	13733625	+	3689	7	P	1597.879	0.121	0.106
AK072713	CG	LH	13737587	+	13667	7	P	2150.332	0.133	0.175
AK062794	CG	LH	13738774	-	679	1	P	3646.318	0.467	0
Chr10_2010	UG	LH	13744256	+	317	2	P	1015.296	0.571	0
AK072819	CG	LH	13752524	+	3053	7	P	1715.026	0.282	0.036
Chr10_2013	EG	HH	13756559	-	8330	14	P	1805.129	0.149	0.028
Chr10_2014	EG	LH	13771173	+	1865	4	P	2121.727	0.444	0.217
AK106568	CG	LH	13775384	+	3759	12	P	1977.82	0.209	0.05
Chr10_2016	UG	LH	13784382	+	1565	4	P	3322.286	0.231	0.105
Chr10_2017	UG	LH	13789093	+	719	2	P	4083.523	0.235	0
Chr10_2018	UG	LH	13790689	-	249	1	P	2885.273	0.333	0
Chr10_2019	UG	LH	13792656	-	536	2	P	1691.263	0.222	0.333
Chr10_2020	UG	LH	13794943	+	848	3	A	270.904	0.167	0
Chr10_2021	UG	LH	13804375	+	762	1	P	3001.491	0.294	0
AK106265	CG	HH	13815089	-	3124	5	P	2670.477	0.297	0
Chr10_2023	UG	LH	13819691	+	275	2	A	353.242	0.167	0
Chr10_2024	UG	LH	13823043	-	813	1	P	3881.177	0.389	0
Chr10_2025	EG	HH	13826772	+	4499	12	P	699.495	0.045	0.074
AK108240	CG	HH	13833207	-	2579	9	P	2913.212	0.258	0.115
AK070735	CG	LH	13836660	-	3314	7	P	1964.591	0.207	0.045
Chr10_2027	UG	LH	13844336	-	1146	2	P	1669.122	0.16	0
AK072370	CG	HH	13852981	+	349295	11	P	1908.877	0.208	0.132
Chr10_2029	UG	LH	13860155	+	1416	3	P	3991.473	0.25	0.143

Chr10_2030	EG	HH	13864299	-	405	1	P	6090.411	0.3	0
Chr10_2031	UG	LH	13866439	-	441	1	P	2001.977	0.4	0
AK065401	CG	LH	13873298	+	3388	7	P	1790.45	0.197	0
Chr10_2033	UG	HH	13883503	+	3346	3	P	1065.896	0.095	0.111
Chr10_2034	UG	HH	13887656	-	2046	2	A	464.26	0.107	0.647
AK061311	CG	LH	13889094	+	2774	4	P	1397.604	0.3	0.129
Chr10_2035	UG	HH	13903697	-	2473	2	P	2151.44	0.114	0.053
Chr10_2036	UG	HH	13906923	-	1129	2	A	0	0	0.056
Chr10_2037	UG	LH	13910004	+	1113	2	P	1617.934	0.2	0
Chr10_2038	UG	HH	13912346	+	1080	1	P	1854.672	0.04	0
AK106669	CG	HH	13921134	-	4441	5	P	1873.33	0.049	0
Chr10_2042	UG	LH	13930872	-	1717	2	P	2974.946	0.4	0
Chr10_2043	UG	LH	13946279	-	411	1	P	1773.066	0.4	0
Chr10_2044	UG	LH	13957595	-	633	1	P	2504.277	0.286	0
Chr10_2047	UG	LH	13983089	+	1687	3	P	2935.743	0.214	0.167
Chr10_2048	UG	HH	13989450	-	1208	2	P	813.424	0.192	0
Chr10_2050	UG	LH	13995374	+	780	2	P	3078.346	0.692	0
Chr10_2051	UG	HH	13996825	-	1170	1	A	0	0	0
Chr10_2052	UG	HH	13999397	-	2556	3	P	1189.52	0.08	0.407
Chr10_2053	UG	HH	14014582	+	1893	3	P	1035.613	0.189	0
Chr10_2054	UG	LH	14019699	+	2666	5	P	1901.101	0.176	0.25
Chr10_2056	UG	HH	14033422	-	1057	2	A	0	0	0
Chr10_2057	EG	HH	14041285	+	4328	5	P	2137.015	0.278	0.073
AK100938	CG	HH	14050096	+	1891	3	P	1874.969	0.419	0
AK071270	CG	HH	14057220	+	3299	5	P	2318.016	0.415	0.031
AK100205	CG	HH	14067283	-	6401	15	P	1238.547	0.102	0.013
AK066936	CG	HH	14075358	-	6832	14	P	2200.469	0.144	0.017
Chr10_2062	UG	HH	14086184	+	391	2	P	1416.58	0.333	0
Chr10_2064	UG	HH	14095931	+	1873	6	P	1856.439	0.091	0.222
AK107152	CG	HH	14110736	+	934	3	P	1847.729	0.316	0.5
AK062774	CG	LH	14128645	+	2060	2	P	2792.126	0.211	0.042
Chr10_2067	UG	LH	14131104	-	714	1	P	5106.068	0.25	0
Chr10_2068	UG	LH	14137551	+	1082	2	P	1525.062	0.231	0
Chr10_2069	UG	HH	14139009	-	2715	10	P	3012.285	0.226	0
Chr10_2070	UG	HH	14144491	+	778	2	P	3228.796	0.438	0.5
Chr10_2071	UG	HH	14147097	-	1735	4	P	2824.862	0.344	0.2
Chr10_2072	UG	HH	14158925	-	1520	3	P	2451.94	0.045	0.091
Chr10_2073	UG	HH	14164850	+	3489	3	P	814.084	0.159	0.25
AK065175	CG	HH	14171179	+	2466	2	P	1317.445	0.148	0
Chr10_2076	UG	LH	14178744	+	588	1	P	2585.303	0.769	0
AK103093	CG	HH	14183234	+	3371	5	P	1396.403	0.206	0.051
AK065583	CG	HH	14186695	-	2825	4	P	933.181	0.086	0.074
AK106662	CG	LH	14190201	+	1797	3	P	2916.633	0.154	0.174
AK063228	CG	LH	14194892	+	616	3	P	2382.109	0.2	0
Chr10_2081	UG	LH	14202830	+	1508	4	P	1980.31	0.462	0.3
Chr10_2082	UG	HH	14207997	+	1283	2	P	2429.25	0.059	0
Chr10_2083	UG	HH	14210854	-	1017	2	P	1627.188	0.444	0
Chr10_2084	UG	LH	14223243	-	561	1	A	727.569	0.077	0
Chr10_2085	UG	LH	14225754	+	869	2	P	1064.578	0.111	0
AK067419	CG	HH	14231734	+	7456	17	P	2236.116	0.143	0.025
Chr10_2087	UG	HH	14240992	-	2741	2	P	1189.865	0.167	0.048
Chr10_2088	UG	HH	14247492	-	3607	2	A	497.631	0.057	0.024
Chr10_2089	UG	LH	14255862	+	280	2	P	2460.844	0.5	0.333
Chr10_2090	UG	HH	14257971	-	4046	2	P	1746.092	0.257	0.098
Chr10_2091	UG	HH	14264113	-	4734	8	P	1678.506	0.115	0.065
Chr10_2092	UG	LH	14272120	+	544	2	A	0	0	0
Chr10_2093	UG	LH	14274866	-	381	2	P	1107.711	0.25	0
Chr10_2094	EG	LH	14278365	+	1388	4	P	2940.031	0.48	0.4
Chr10_2095	UG	LH	14281943	-	1021	2	P	4377.006	0.682	0
Chr10_2096	UG	HH	14311066	-	4577	3	P	2468.042	0.3	0.149
Chr10_2097	UG	LH	14326717	+	456	1	P	1382.523	0.1	0
Chr10_2098	UG	HH	14330725	+	3725	5	P	2492.923	0.074	0.037

AK070200	CG	HH	14336055	+	4260	9	P	1626.235	0.25	0.069
AK105860	CG	HH	14341053	+	2261	1	P	1727.057	0.14	0
AK071359	CG	HH	14344192	+	2370	2	P	2827.771	0.245	0
Chr10_2101	UG	LH	14349130	+	1853	3	P	2639.131	0.077	0.143
Chr10_2102	UG	LH	14351331	+	1941	2	P	2560.96	0.524	0.25
Chr10_2103	EG	HH	14353655	-	7999	13	P	1960.344	0.039	0.041
Chr10_2104	UG	HH	14362686	-	2042	2	P	2789.919	0.313	0.091
Chr10_2105	UG	LH	14366871	-	3561	6	A	0	0	0.034
Chr10_2107	UG	HH	14378684	-	3233	2	A	432.74	0.167	0.106
Chr10_2109	UG	LH	14392804	-	1148	2	P	3794	0.667	0.286
Chr10_2110	UG	HH	14395071	+	349	2	P	4136.727	0.167	0.5
Chr10_2111	UG	HH	14395679	-	909	2	P	2506.084	0.167	0
Chr10_2112	EG	HH	14399810	-	1134	4	P	3810.489	0.222	0.286
AK058908	CG	HH	14410873	+	1242	3	P	1625.415	0.333	0
AK066106	CG	HH	14413600	+	8010	14	P	2197.75	0.198	0.118
Chr10_2115	EG	HH	14416480	-	2138	4	P	2381.625	0.194	0.125
Chr10_2116	EG	HH	14425140	+	7692	14	P	1541.457	0.043	0.063
Chr10_2117	UG	HH	14434963	-	3889	4	P	2163.179	0.208	0.136
Chr10_2118	UG	LH	14441272	-	211	2	P	1758.044	0.4	0
Chr10_2119	UG	LH	14443497	-	1543	3	P	1627.736	0.161	0
Chr10_2120	EG	HH	14446676	+	2682	7	P	773.256	0.1	0.034
Chr10_2121	UG	HH	14450235	-	878	2	P	1288.344	0.214	0
AK063397	CG	HH	14452923	+	3381	7	P	2745.992	0.276	0.023
AK071645	CG	LH	14475812	+	3457	14	P	3350.405	0.216	0.056
Chr10_2125	UG	HH	14485708	+	1998	8	P	2531.146	0.12	0
Chr10_2126	UG	HH	14488331	-	1802	3	P	1741.917	0.286	0
Chr10_2127	UG	LH	14494231	-	573	2	P	1882.445	0.417	0
Chr10_2128	EG	HH	14496338	+	4223	14	P	662.88	0.1	0.075
Chr10_2129	UG	HH	14502129	-	1542	1	P	657.062	0.2	0
Chr10_2130	UG	HH	14507468	+	1581	1	P	1717.558	0.114	0
Chr10_2131	UG	LH	14509794	-	912	3	P	4237.313	0.333	0.455
Chr10_2132	UG	HH	14516472	-	482	2	A	599.01	0.1	0
AK069054	CG	HH	14518186	-	2705	2	P	2249.774	0.14	0.143
Chr10_2135	UG	HH	14533481	+	3788	3	P	1122.312	0.081	0
Chr10_2136	UG	LH	14539262	+	1365	1	P	1609.509	0.103	0
Chr10_2137	UG	HH	14541402	-	1472	2	A	514.661	0.034	0
Chr10_2138	UG	LH	14546079	-	4115	2	P	1913.758	0.444	0.154
Chr10_2139	UG	LH	14554112	-	453	1	P	2404.56	0.3	0
Chr10_2140	UG	LH	14558284	-	1052	4	P	2713.119	0.077	0.125
Chr10_2142	UG	LH	14566680	+	261	1	P	1390.209	0.143	0
Chr10_2143	UG	LH	14570056	-	375	1	P	3049.079	0.333	0
Chr10_2144	UG	LH	14583084	-	513	1	P	2644.722	0.75	0
AK067013	CG	HH	14588626	-	11860	6	P	2785.387	0.276	0.072
AK059279	CG	LH	14606453	+	4166	8	P	3553.591	0.12	0.197
Chr10_2147	EG	HH	14610871	-	3881	2	P	4855.622	0.36	0.152
AK109236	CG	LH	14624110	+	1186	3	P	2877.324	0.429	0.091
Chr10_2149	UG	LH	14629069	-	1730	2	P	2314.917	0.345	0.444
Chr10_2150	UG	LH	14635682	+	856	3	P	9496.637	0.286	0.25
AK069924	CG	LH	14637727	-	2114	2	P	1843.472	0.316	0.333
Chr10_2152	UG	LH	14642674	-	387	2	A	0	0	0
Chr10_2153	EG	HH	14649185	+	12426	19	P	2778.213	0.103	0.115
Chr10_2155	UG	LH	14669613	+	438	1	P	1658.487	0.1	0
AK059905	CG	HH	14669894	+	9198	2	A	629.028	0.083	0.09
AK101643	CG	LH	14683866	-	5226	12	P	5434.326	0.137	0.071
Chr10_2157	UG	LH	14688885	+	864	2	P	6011.949	0.5	0
Chr10_2158	UG	HH	14692867	+	4238	6	P	1957.256	0.176	0.075
AK066993	CG	HH	14704690	+	4082	7	P	1686.71	0.215	0.273
Chr10_2162	EG	LH	14739029	+	252	1	P	1933.266	0.5	0
Chr10_2163	UG	HH	14740786	+	4979	4	P	2012.546	0.25	0.155
AK066983	CG	LH	14741404	-	980	1	P	6163.233	0.19	0
AK072242	CG	HH	14745193	+	35100	4	P	1615.013	0.224	0.084
Chr10_2164	EG	HH	14747987	+	5171	7	P	2116.592	0.169	0.064

Chr10_2166	UG	LH	14781206	+	2975	3	P	1036.184	0.211	0.227
Chr10_2168	UG	LH	14797894	+	162	1	P	1541.807	0.5	0
Chr10_2169	UG	LH	14802499	+	7305	8	P	3616.248	0.344	0.112
Chr10_2170	UG	HH	14811324	+	6431	5	P	2238.39	0.105	0.06
Chr10_2171	UG	LH	14819377	+	2316	3	A	468.092	0.045	0
Chr10_2172	UG	LH	14825635	-	415	2	A	351.735	0.167	0
Chr10_2173	UG	LH	14828082	+	483	1	P	4778.395	0.455	0
Chr10_2174	UG	LH	14833410	+	150	1	P	3052.588	0.25	0
Chr10_2175	UG	LH	14836467	-	279	1	P	3053.141	0.571	0
Chr10_2176	UG	LH	14842778	+	150	1	P	1996.1	0.25	0
Chr10_2177	UG	LH	14849904	-	567	1	P	5245.105	0.357	0
Chr10_2179	EG	LH	14868089	+	1961	2	P	1579.677	0.083	0.2
Chr10_2180	UG	HH	14899160	+	3794	2	P	1412.084	0.313	0.082
Chr10_2182	UG	LH	14925754	-	402	1	P	3912.6	0.6	0
AK063548	CG	HH	14938035	+	1701	5	P	1235.111	0.143	0.25
Chr10_2184	UG	HH	14941424	-	4429	2	P	1396	0.295	0
Chr10_2187	EG	LH	14962926	-	3477	3	A	638.417	0.071	0.016
Chr10_2188	UG	LH	14970953	+	1754	4	P	2852.139	0.333	0.048
Chr10_2189	UG	LH	14974113	-	1533	4	P	4744.005	0.5	0.05
Chr10_2190	UG	HH	14984016	-	1198	2	P	746.452	0.154	0
Chr10_2191	UG	LH	14990055	+	2474	3	P	1201.768	0.135	0
Chr10_2192	UG	LH	14997364	-	1092	1	P	5203.491	0.583	0
Chr10_2195	UG	LH	15017479	-	1104	2	P	6831.113	0.444	0
AK103350	CG	LH	15024505	-	934	1	P	3624.779	0.65	0
AK102661	CG	LH	15029423	-	1034	2	P	4705.097	0.625	0
AK063328	CG	LH	15038647	-	800	3	P	5037.53	0.45	0
Chr10_2199	EG	LH	15056668	-	558	1	P	4771.854	0.769	0
Chr10_2200	UG	LH	15060050	-	558	1	P	5295.497	0.769	0
Chr10_2201	EG	LH	15062556	-	1626	2	P	2700.651	0.538	0
Chr10_2202	UG	LH	15070174	-	567	1	P	5881.079	0.538	0
AK070158	CG	HH	15071590	-	3747	12	P	2106.278	0.188	0
Chr10_2204	EG	LH	15078571	-	570	1	P	5502.229	0.462	0
Chr10_2205	UG	LH	15081486	-	582	1	P	7352.885	0.385	0
Chr10_2206	UG	LH	15092321	+	2606	5	P	2046.057	0.375	0.196
Chr10_2207	EG	LH	15098547	-	1981	2	P	4610.179	0.318	0.273
Chr10_2208	UG	LH	15106678	+	609	1	P	3316.799	0.571	0
Chr10_2209	UG	LH	15110102	+	423	1	P	830.088	0.3	0
Chr10_2210	UG	LH	15111330	-	564	1	P	2049.142	0.462	0
Chr10_2211	UG	LH	15117209	-	585	1	P	4480.169	0.571	0
Chr10_2212	EG	LH	15123572	-	576	1	P	3904.69	0.538	0
AK106252	CG	LH	15132265	+	1418	2	P	1305.379	0.375	0
Chr10_2213	UG	HH	15132272	-	840	2	P	960.626	0.316	0
Chr10_2214	EG	LH	15134319	+	570	1	P	4392.177	0.615	0
Chr10_2215	UG	LH	15138639	-	999	2	P	4102.999	0.214	0
AK073080	CG	LH	15143510	-	835	2	P	7855.531	0.4	0
Chr10_2217	EG	LH	15147005	-	1650	2	P	3521.636	0.6	0.053
Chr10_2218	EG	LH	15154724	-	576	1	P	5189.068	0.615	0
AK109095	CG	LH	15161015	-	757	1	P	3971.346	0.444	0
Chr10_2220	UG	LH	15168180	-	576	1	P	3126.335	0.615	0
Chr10_2221	EG	LH	15173377	-	576	1	P	6507.5	0.462	0
Chr10_2223	EG	LH	15178811	-	576	1	P	3370.23	0.615	0
Chr10_2224	UG	LH	15183044	-	579	1	P	4740.342	0.462	0
Chr10_2225	UG	LH	15184382	-	318	1	P	4774.219	0.5	0
Chr10_2226	UG	LH	15197745	-	1597	2	P	2582.764	0.438	0.333
AK072120	CG	HH	15202050	+	8587	29	P	1388.55	0.215	0.092
Chr10_2228	UG	LH	15217494	+	943	3	P	1311.264	0.25	0.125
Chr10_2229	UG	HH	15218971	+	786	1	P	4945.638	0.222	0
AK099701	CG	HH	15220866	-	7401	11	P	3591.668	0.185	0.076
Chr10_2231	UG	LH	15228865	+	731	2	P	4779.941	0.333	0.2
AK101916	CG	HH	15230406	+	2289	1	P	1499.82	0.333	0
Chr10_2233	EG	HH	15241604	+	416	2	P	1397.163	0.25	0
AK099882	CG	HH	15246582	+	6669	19	P	1034.111	0.136	0.03

AK102843	CG	HH	15253321	-	3943	6	P	2017.739	0.259	0.034
AK104810	CG	HH	15275436	-	3548	13	P	2796.475	0.316	0.073
AK064571	CG	HH	15280723	-	2327	6	P	1457.591	0.044	0
AK103490	CG	HH	15315422	+	4435	9	P	2258.191	0.162	0.05
Chr10_2241	UG	HH	15320541	-	5787	5	P	2686.561	0.342	0.133
AK063553	CG	LH	15332391	+	2144	8	P	1792.037	0.324	0
AK059744	CG	HH	15334522	-	2747	9	P	1133.667	0.107	0.091
AK072460	CG	HH	15340902	+	4147	15	P	2086.108	0.154	0
Chr10_2245	UG	HH	15348429	-	2087	8	A	0	0	0
Chr10_2246	UG	LH	15354504	-	472	2	P	6030.588	0.4	0
AK068079	CG	LH	15359992	+	21784	26	P	1380.71	0.1	0.12
Chr10_2250	UG	HH	15384719	-	1710	1	P	3243.957	0.079	0
Chr10_2251	UG	HH	15388277	-	1110	1	P	1276.304	0.167	0
Chr10_2253	UG	HH	15395487	-	1651	3	P	2142.999	0.088	0
Chr10_2254	UG	HH	15397570	-	1155	2	A	664.363	0.08	0
Chr10_2255	UG	LH	15402199	+	1465	2	P	2404.347	0.25	0.1
Chr10_2256	UG	LH	15407733	+	3440	4	P	2061.945	0.242	0.025
Chr10_2257	UG	HH	15412768	-	4807	5	A	600.336	0.065	0
Chr10_2258	UG	LH	15418224	-	1158	4	A	0	0	0
Chr10_2259	UG	HH	15423189	-	1213	2	A	517.878	0.071	0.071
Chr10_2260	UG	LH	15424635	-	977	3	P	1974.489	0.067	0
AK101347	CG	HH	15428754	+	5717	11	P	1791.369	0.239	0.063
Chr10_2262	UG	HH	15436113	-	1759	3	P	3612.032	0.528	0
Chr10_2263	UG	HH	15440775	-	1521	1	P	1888.008	0.088	0
Chr10_2264	UG	LH	15442862	+	579	1	P	2770.675	0.462	0
Chr10_2265	UG	HH	15445507	-	1407	1	P	1341.196	0.226	0
Chr10_2266	UG	LH	15457855	+	520	2	P	2713.287	0.417	0
Chr10_2267	UG	HH	15464671	+	2189	2	P	2472.346	0.235	0
AK110328	CG	HH	15469122	-	19737	2	P	2804.196	0.191	0.144
Chr10_2268	UG	HH	15469258	-	1788	1	P	2748.175	0.205	0
Chr10_2269	UG	LH	15474244	-	4691	5	P	3631.371	0.129	0.113
AK110981	CG	LH	15481451	-	1546	3	P	3036.769	0.278	0
Chr10_2270	UG	LH	15481697	+	2193	2	P	3120.188	0.077	0.281
AK070542	CG	LH	15503355	+	17779	9	P	1948.885	0.102	0.066
Chr10_2278	EG	HH	15528745	-	6793	9	P	2476.553	0.143	0.063
Chr10_2279	EG	HH	15538952	-	1754	6	P	1742.224	0.25	0.043
Chr10_2281	EG	LH	15546396	-	1371	3	P	7106.934	0.167	0.056
Chr10_2282	UG	LH	15548087	-	1385	2	P	2332.578	0.1	0.15
AK059274	CG	LH	15552200	-	1633	5	P	733.609	0.5	0.091
Chr10_2285	EG	LH	15558677	-	1963	3	P	3907.612	0.308	0.1
Chr10_2286	UG	LH	15563251	+	1069	3	P	858.674	0.118	0
AK066946	CG	LH	15575367	+	565	2	P	1645.057	0.538	0
AK062813	CG	LH	15575992	-	727	2	P	3299.789	0.25	0
AK073745	CG	LH	15577658	-	3239	6	P	1785.074	0.082	0
Chr10_2288	UG	LH	15581456	+	330	1	P	5968.243	0.5	0
Chr10_2289	UG	LH	15587212	-	1603	4	P	1536.484	0.143	0.158
Chr10_2290	UG	LH	15592142	+	2479	6	P	3664.742	0.125	0.1
Chr10_2291	UG	LH	15596769	-	420	1	P	1907.864	0.1	0
AK108436	CG	LH	15598974	+	1967	3	A	300.138	0.083	0.125
Chr10_2292	UG	LH	15599082	-	312	1	P	2315.305	0.625	0
Chr10_2293	EG	LH	15601699	-	3774	5	P	1122.606	0.143	0.118
AK068897	CG	LH	15606853	-	1415	10	P	2866.464	0.091	0
AK059107	CG	HH	15626253	+	2045	4	P	1102.527	0.227	0.043
AK069617	CG	HH	15628788	+	5376	20	P	1970.503	0.355	0.179
Chr10_2296	UG	HH	15636102	+	3391	9	P	1630.182	0.15	0.103
AK108201	CG	HH	15643799	+	1530	7	P	2178.922	0.361	0
AK109194	CG	HH	15647025	+	1148	3	P	1255.751	0.261	0
Chr10_2299	UG	HH	15648974	-	1903	4	A	0	0	0.091
Chr10_2300	UG	HH	15665566	-	3112	3	P	3022.696	0.095	0.143
Chr10_2301	UG	HH	15671904	-	4523	5	P	1561.395	0.103	0.074
Chr10_2303	UG	LH	15693858	-	408	1	P	2030.98	0.444	0
AK102352	CG	HH	15697774	-	3343	7	P	1310.794	0.257	0.128

Chr10_2305	EG	LH	15702101	+	378	1	P	2083.765	0.333	0
AK103475	CG	HH	15703049	-	2531	5	P	2386.849	0.194	0.042
AK068906	CG	HH	15706403	-	13490	12	P	4352.455	0.15	0.097
Chr10_2309	UG	LH	15722696	+	2254	2	A	617.022	0.059	0
Chr10_2310	UG	HH	15725558	-	3764	4	P	2137.511	0.135	0.25
Chr10_2311	EG	LH	15732230	-	555	1	P	1402.753	0.308	0
AK066663	CG	HH	15733260	-	4676	16	P	1663.408	0.118	0.109
Chr10_2313	EG	HH	15740917	-	1965	1	P	1391.481	0.14	0
Chr10_2314	UG	HH	15751906	-	1821	1	P	3864.85	0.1	0
Chr10_2315	UG	HH	15754466	-	1938	1	P	2219.669	0.256	0
AK072090	CG	HH	15757083	-	4840	4	P	1614.644	0.229	0.035
Chr10_2317	UG	HH	15765780	+	410	2	P	4665.53	0.125	0
Chr10_2318	UG	LH	15769990	+	2477	2	P	1529.185	0.083	0.132
Chr10_2319	UG	LH	15775761	-	492	1	P	2296.179	0.273	0
Chr10_2320	UG	LH	15778702	+	756	1	P	1224.007	0.412	0
AK067249	CG	HH	15783802	+	2430	8	P	1392.314	0.288	0.25
Chr10_2322	EG	HH	15793652	-	4913	10	A	471.59	0.125	0.028
AK110879	CG	HH	15799305	+	3528	11	P	1983.329	0.133	0.031
Chr10_2324	UG	HH	15804108	+	600	2	P	3861.625	0.25	0
AK073137	CG	LH	15805169	-	3316	4	P	1598.168	0.161	0.063
Chr10_2326	UG	LH	15811009	+	2091	6	P	3052.423	0.063	0.097
Chr10_2327	UG	HH	15814361	-	468	1	A	644.241	0.091	0
AK064778	CG	HH	15816103	-	6986	23	P	1801.076	0.222	0.089
Chr10_2329	UG	HH	15825898	+	1178	3	P	1693.659	0.111	0.188
Chr10_2330	UG	LH	15829125	-	1533	3	P	2300.832	0.188	0.111
AK069224	CG	HH	15839544	+	1822	5	P	1612.45	0.176	0
Chr10_2332	UG	LH	15842617	-	731	2	P	4331.81	0.6	0.333
Chr10_2333	EG	HH	15844263	-	1781	4	P	1617.36	0.462	0.111
AK108440	CG	LH	15849411	+	9642	2	P	1990.219	0.286	0.077
Chr10_2335	UG	LH	15858481	+	270	1	P	2051.594	0.714	0
AK107743	CG	LH	15867401	+	887	1	P	2446.206	0.526	0
Chr10_2338	UG	HH	15871901	+	1654	2	P	874.609	0.25	0.118
AK071253	CG	HH	15874414	+	4822	11	P	3019.713	0.068	0.048
Chr10_2340	EG	HH	15886383	+	2690	5	P	4040.077	0.375	0.023
AK072259	CG	HH	15890115	+	4556	10	P	2276.229	0.395	0.056
Chr10_2342	EG	HH	15895361	-	5705	2	P	2088.332	0.162	0
Chr10_2346	UG	LH	15918381	-	1723	4	P	1125.462	0.308	0.24
Chr10_2347	UG	LH	15926154	-	558	1	P	4454.376	0.308	0
Chr10_2348	UG	LH	15927473	-	602	2	P	3833.045	0.154	0
Chr10_2349	UG	HH	15931031	-	3000	1	P	1330.978	0.046	0
Chr10_2350	UG	LH	15936079	-	1321	4	A	0	0	0.091
Chr10_2351	UG	HH	15938067	-	2940	2	P	1006.734	0.113	0.333
Chr10_2352	UG	HH	15942489	-	3031	2	P	1593.138	0.086	0
Chr10_2353	EG	HH	15948406	-	3107	2	A	632.726	0.045	0
AK110897	CG	LH	15961114	+	2026	1	P	1327.453	0.044	0
Chr10_2355	UG	HH	15969178	-	2827	3	A	752.771	0.065	0.2
Chr10_2356	EG	HH	15986269	+	4199	2	P	2432.822	0.152	0
Chr10_2357	UG	LH	15991388	+	249	1	A	376.121	0.333	0
Chr10_2358	UG	HH	15997530	-	2793	2	P	1016.445	0.07	0
Chr10_2359	UG	HH	16005707	-	3420	1	P	1594.452	0.067	0
Chr10_2361	UG	LH	16022787	+	6527	9	P	2486.197	0.195	0.029
Chr10_2362	UG	LH	16030225	+	3216	5	P	1429.866	0.175	0.065
Chr10_2363	UG	LH	16038659	-	964	3	A	467.513	0.167	0
Chr10_2366	UG	HH	16065153	+	511	2	A	628.826	0.167	0
Chr10_2367	UG	HH	16067759	+	4601	4	P	1833.459	0.225	0.15
Chr10_2368	UG	HH	16075064	-	3012	2	P	702.745	0.079	0
Chr10_2369	EG	HH	16081932	-	1726	2	P	807.52	0.071	0.111
AK105737	CG	HH	16087496	+	47273	6	P	1300.251	0.208	0.081
Chr10_2371	EG	HH	16090357	+	1482	1	P	1071.89	0.212	0
Chr10_2372	UG	HH	16108538	-	1725	1	P	1395.409	0.026	0
Chr10_2373	EG	HH	16111238	-	1764	2	P	937.19	0.031	0
Chr10_2374	UG	LH	16113970	+	3518	4	P	940.326	0.036	0

Chr10_2375	UG	HH	16118738	+	3258	5	A	0	0	0.063
AK066165	CG	HH	16122817	-	1785	1	P	1493.017	0.077	0
Chr10_2378	UG	LH	16141759	+	838	2	P	3237.638	0.333	0.462
AK101055	CG	HH	16157458	+	5261	3	P	1105.741	0.245	0.078
Chr10_2380	UG	LH	16165980	+	290	2	A	0	0	0
Chr10_2381	EG	LH	16178448	+	918	1	P	2514.139	0.476	0
AK062625	CG	LH	16179406	+	1372	2	P	654.442	0.286	0.25
AK070210	CG	HH	16185963	+	1291	3	P	1047.136	0.207	0
Chr10_2383	EG	HH	16191618	-	1811	4	P	1510.863	0.053	0.067
AK100751	CG	HH	16198430	-	5761	12	P	1515.348	0.133	0.048
Chr10_2385	UG	LH	16206617	+	1016	2	P	1950.619	0.091	0.182
AK105134	CG	LH	16222869	+	2891	4	P	2804.499	0.45	0.048
Chr10_2387	EG	HH	16226666	-	8314	16	P	3419.322	0.123	0.082
AK067894	CG	HH	16235713	-	5904	9	P	2805.969	0.147	0.028
AK073804	CG	LH	16249430	-	3450	3	P	7490.897	0.2	0.083
Chr10_2390	UG	HH	16257164	-	2155	5	P	1731.353	0.179	0
Chr10_2392	UG	LH	16276527	-	411	1	P	2960.943	0.333	0
Chr10_2393	EG	HH	16310388	+	922	2	A	485.5	0.278	0
Chr10_2394	UG	HH	16311681	-	300	1	P	2119.864	0.286	0
Chr10_2395	UG	HH	16330060	+	626	2	P	1362.58	0.143	0
Chr10_2396	EG	HH	16332723	+	1856	3	P	2035.208	0.294	0.043
Chr10_2397	UG	LH	16335779	-	270	1	P	920.031	0.333	0
Chr10_2399	UG	LH	16348368	-	234	1	A	0	0	0
AK067795	CG	HH	16360841	-	8149	20	P	2585.135	0.108	0.023
Chr10_2402	UG	HH	16384022	+	6345	6	P	2579.262	0.157	0.167
Chr10_2403	UG	LH	16393643	-	984	1	P	3749.543	0.318	0
Chr10_2404	EG	HH	16397488	-	1695	1	A	637.978	0.132	0
Chr10_2405	UG	HH	16400443	-	4575	6	P	2399.641	0.273	0.111
Chr10_2406	UG	LH	16411114	-	7866	17	P	2060.474	0.156	0.047
Chr10_2407	UG	LH	16421107	+	5060	12	P	1046.474	0.061	0.131
Chr10_2409	UG	HH	16449691	-	2393	2	P	4480.24	0.089	0
Chr10_2410	UG	LH	16453726	-	730	2	P	2967.949	0.308	0.333
Chr10_2411	EG	HH	16458919	-	2171	3	P	1219.689	0.154	0
Chr10_2412	UG	HH	16464097	-	1293	3	P	4424.902	0.308	0.067
Chr10_2417	UG	LH	16501423	+	935	2	P	1037.061	0.167	0.667
Chr10_2418	UG	LH	16509561	+	1496	5	P	5001.231	0.294	0.286
AK101853	CG	HH	16511824	+	2480	2	P	1931.725	0.1	0.333
AK065629	CG	HH	16515806	+	3035	5	P	1193.476	0.171	0.033
Chr10_2421	UG	HH	16521896	+	3204	12	P	1657.925	0.093	0
AK070314	CG	LH	16528472	+	1625	7	P	5390.975	0.171	0
Chr10_2423	UG	LH	16533639	+	108	2	A	0	0	0
Chr10_2424	UG	LH	16534736	+	1554	3	P	3895.926	0.2	0.154
AK072826	CG	HH	16537280	-	3352	13	P	1774.456	0.179	0.07
AK110990	CG	LH	16542188	-	1678	2	P	1816.182	0.314	0
AK067811	CG	HH	16546354	+	7835	16	P	2248.327	0.239	0.056
AK070445	CG	HH	16557397	+	5160	8	P	2272.237	0.212	0.049
AK103437	CG	HH	16568526	-	31500	7	P	3634.034	0.161	0.088
AK106419	CG	HH	16573795	+	15072	36	P	1983.253	0.068	0.075
AK067258	CG	HH	16589876	+	5344	9	P	1722.839	0.167	0.088
Chr10_2432	UG	HH	16596718	-	2025	2	A	577.123	0.111	0
Chr10_2434	UG	LH	16618387	+	2233	3	P	2046.92	0.222	0.026
Chr10_2435	UG	HH	16644239	+	1772	4	P	1999.029	0.238	0.111
AK102336	CG	HH	16652860	+	14610	38	P	2492.132	0.115	0.06
Chr10_2437	UG	HH	16668341	-	4253	6	P	2620.572	0.407	0.017
AK072265	CG	LH	16701972	-	4948	14	P	1553.106	0.14	0.09
AK066396	CG	HH	16709951	+	23341	9	P	1658.333	0.622	0.119
AK109011	CG	HH	16719736	-	2718	8	P	2200.809	0.265	0.111
Chr10_2442	UG	HH	16740999	-	1212	2	P	2556.614	0.435	0.5
Chr10_2447	EG	LH	16763409	-	11876	21	P	1535.005	0.056	0.049
AK073485	CG	LH	16780923	-	4002	15	P	2259.461	0.271	0.091
Chr10_2449	UG	LH	16784213	+	906	2	P	5365.291	0.333	0.786
Chr10_2450	UG	HH	16786292	-	435	1	P	2341.636	0.1	0

AK062836	CG	LH	16787810	+	3546	7	P	1326.034	0.391	0.07
Chr10_2451	EG	LH	16793717	+	1254	2	P	2536.287	0.455	0.133
AK062758	CG	LH	16794568	-	1311	3	P	1952.705	0.214	0
Chr10_2452	EG	HH	16800540	+	2784	3	P	1789.787	0.179	0.2
AK108805	CG	HH	16825476	+	4915	8	P	1630.392	0.286	0.069
AK063255	CG	HH	16831568	+	2732	4	P	1680.309	0.211	0.122
AK103346	CG	HH	16836503	+	6855	9	P	1612.967	0.071	0.057
Chr10_2455	EG	HH	16847262	+	1603	4	P	1509.196	0.333	0
AK107572	CG	LH	16868921	+	2069	5	P	1110.662	0.182	0
Chr10_2457	EG	HH	16882298	+	4328	4	P	4140.989	0.213	0.043
Chr10_2459	UG	LH	16906376	-	309	1	P	4812.87	0.429	0
Chr10_2461	UG	LH	16929683	-	309	1	P	3125.071	0.625	0
AK102603	CG	HH	16934538	+	6964	26	P	2619.845	0.274	0.043
AK066524	CG	HH	16952747	-	6087	7	P	2392.577	0.189	0.183
Chr10_2464	UG	LH	16958237	+	2738	3	P	5990.016	0.357	0.152
Chr10_2465	UG	LH	16969849	-	741	2	P	1376.713	0.25	0.25
Chr10_2466	UG	HH	16974943	+	522	1	A	521.903	0.167	0
Chr10_2467	UG	LH	16992939	+	1789	7	P	777.379	0.059	0
Chr10_2468	UG	LH	16996440	-	459	1	P	1079.672	0.182	0
Chr10_2469	UG	HH	17000487	-	360	1	P	6079.492	0.444	0
Chr10_2470	EG	HH	17001583	-	6257	10	P	1424.836	0.188	0.088
Chr10_2471	UG	HH	17023932	+	3382	3	P	3715.813	0.333	0.158
Chr10_2472	UG	LH	17029332	+	974	3	A	311.933	0.167	0.063
Chr10_2473	UG	HH	17033780	+	1356	3	P	2643.436	0.389	0.167
Chr10_2474	UG	LH	17035644	-	2959	6	P	1590.605	0.17	0.2
Chr10_2475	UG	LH	17041506	-	3609	3	P	896.355	0.119	0.1
Chr10_2476	UG	LH	17046757	+	881	2	P	5838.755	0.2	0.5
Chr10_2477	UG	LH	17054031	+	1458	2	P	2395.512	0.129	0
AK063227	CG	LH	17059761	-	783	1	P	3446.477	0.5	0
Chr10_2479	UG	LH	17065205	+	764	2	P	1581.724	0.2	0
Chr10_2480	UG	LH	17068221	-	2021	5	P	1231.81	0.103	0
Chr10_2481	UG	LH	17074410	-	3065	2	P	2051.768	0.279	0
Chr10_2485	UG	HH	17093920	-	3225	3	P	2254.976	0.394	0.027
Chr10_2486	UG	LH	17099158	+	360	1	P	1445.236	0.333	0
Chr10_2487	UG	HH	17104442	-	3290	6	P	3678.19	0.313	0.095
AK071332	CG	HH	17111855	-	4867	8	P	3381.434	0.262	0.064
Chr10_2489	EG	HH	17121083	-	2137	6	P	1169.454	0.294	0.1
AK071436	CG	LH	17125082	-	2745	3	P	2643.299	0.189	0
Chr10_2491	UG	LH	17133929	+	1228	3	P	2464.824	0.304	0
Chr10_2492	UG	HH	17135276	-	1385	4	A	668.714	0.222	0
Chr10_2493	UG	LH	17139734	-	1156	2	P	3605.198	0.5	0
Chr10_2494	UG	LH	17144196	+	412	2	A	0	0	0
AK107329	CG	HH	17144866	-	3309	5	P	2379.897	0.192	0.043
Chr10_2496	UG	LH	17148426	+	1638	3	P	2408.819	0.412	0.316
Chr10_2497	UG	HH	17151179	-	2702	4	P	2776.687	0.12	0
Chr10_2498	UG	LH	17157074	+	2253	1	P	2862.377	0.204	0
AK067463	CG	HH	17159884	-	2472	11	P	986.054	0.2	0.067
AK105510	CG	HH	17164372	+	2279	2	P	1761.573	0.091	0.059
Chr10_2501	EG	HH	17167210	-	1287	1	P	3631.48	0.571	0
AK101641	CG	LH	17169169	-	11894	16	P	1466.309	0.14	0.044
AK063971	CG	HH	17182182	-	4461	6	A	632.071	0.03	0.063
Chr10_2506	EG	HH	17202763	+	8703	14	P	1797.566	0.14	0.061
Chr10_2507	UG	LH	17213542	+	1700	2	A	348.084	0.083	0.294
AK070826	CG	HH	17222530	+	2570	4	P	2184.691	0.175	0.063
Chr10_2509	UG	HH	17225671	+	12264	23	P	1585.13	0.065	0.044
Chr10_2510	UG	LH	17240758	-	2266	2	P	3783.299	0.391	0.074
Chr10_2511	UG	LH	17247516	-	979	2	P	2730.377	0.444	0
Chr10_2512	UG	LH	17250873	+	243	1	P	809.702	0.5	0
AK106675	CG	LH	17251698	+	3259	4	P	3501.321	0.22	0.276
Chr10_2514	EG	HH	17255764	-	3692	3	P	1905.152	0.189	0.068
Chr10_2515	UG	HH	17265437	+	2926	2	A	579.89	0.143	0.093
Chr10_2516	EG	LH	17269438	-	3844	4	P	2201.558	0.227	0.033

AK063786	CG	HH	17278778	+	2727	2	P	1118.548	0.368	0.122
Chr10_2518	UG	LH	17282590	-	1451	2	P	3496.153	0.5	0.067
Chr10_2519	UG	LH	17288018	+	581	2	P	2419.896	0.4	0
Chr10_2520	UG	LH	17289003	-	543	2	P	3872.973	0.333	0
AK063937	CG	HH	17292809	+	4654	6	P	2952.398	0.267	0.153
Chr10_2522	UG	LH	17298211	-	853	2	P	871.295	0.333	0
Chr10_2524	UG	LH	17304145	-	573	1	P	2708.716	0.154	0
Chr10_2525	UG	LH	17306970	+	1090	3	P	2826.987	0.545	0
AK069561	CG	HH	17308980	+	3878	13	P	1615.198	0.155	0.037
AK064407	CG	HH	17313376	-	3597	2	P	4956.237	0.235	0
AK109038	CG	LH	17321643	+	3532	3	P	1663.798	0.226	0.065
AK061153	CG	LH	17326660	+	3294	5	P	3403.314	0.222	0
AK109184	CG	LH	17346764	+	1826	2	P	1225.554	0.375	0
AK102742	CG	LH	17350874	+	9530	3	P	2318.977	0.105	0.107
AK100635	CG	HH	17364077	+	10247	20	P	1192.163	0.061	0.051
Chr10_2537	UG	LH	17378312	-	912	3	A	0	0	0.143
Chr10_2538	UG	LH	17379922	-	948	3	A	0	0	0.286
AK108465	CG	LH	17385878	+	833	2	P	1770.418	0.222	0
AK102087	CG	HH	17412828	+	6994	22	P	1950.231	0.119	0
AK069090	CG	HH	17422966	+	1415	2	P	3109.785	0.069	0
Chr10_2542	UG	HH	17426826	-	969	4	P	848.586	0.059	0
AK101865	CG	HH	17429804	+	4520	16	P	1972.096	0.143	0.039
Chr10_2544	EG	HH	17434973	-	5894	17	P	2832.539	0.106	0.048
Chr10_2545	UG	LH	17442290	-	504	1	A	629.142	0.417	0
Chr10_2546	UG	LH	17446647	-	489	1	P	3247.337	0.25	0
Chr10_2547	UG	LH	17450256	-	446	2	P	4181.477	0.545	0
Chr10_2548	UG	LH	17451363	-	579	2	P	5011.826	0.25	0.2
Chr10_2549	EG	HH	17456511	-	4129	7	P	2111.68	0.071	0.022
Chr10_2550	UG	LH	17472929	+	576	1	P	3617.95	0.357	0
Chr10_2551	UG	LH	17478256	+	576	1	P	4276.497	0.308	0
Chr10_2552	UG	HH	17481186	+	2381	5	P	847.056	0.029	0
AK072753	CG	HH	17484189	+	1037	3	A	348.285	0.105	0.75
AK070942	CG	HH	17486876	+	2129	3	P	2521.242	0.5	0.167
AK108069	CG	HH	17489242	-	2705	6	P	2411.804	0.382	0
Chr10_2556	EG	HH	17493057	+	2918	7	P	1092.064	0.048	0
AK102387	CG	LH	17499692	+	858	2	P	1474.035	0.474	0
Chr10_2558	UG	LH	17502101	-	579	1	P	6877.062	0.231	0
Chr10_2559	UG	LH	17502989	-	1008	2	P	3286.819	0.273	0
Chr10_2560	UG	LH	17505938	-	642	1	A	614.318	0.071	0
Chr10_2561	UG	LH	17509540	-	1067	2	P	3241.325	0.176	0
Chr10_2562	UG	HH	17511414	-	687	1	P	3989.545	0.067	0
Chr10_2563	EG	HH	17513744	-	693	1	P	2898.256	0.133	0
AK100816	CG	HH	17516344	+	1481	2	P	2422.355	0.263	0
Chr10_2566	EG	LH	17532889	+	1402	3	P	5290.129	0.111	0.091
Chr10_2567	UG	HH	17536459	-	954	2	P	779.89	0.188	0.167
Chr10_2568	UG	LH	17538624	+	496	3	P	2800.094	0.4	0.167
Chr10_2569	UG	HH	17542835	-	489	1	A	690.831	0.182	0
Chr10_2570	UG	HH	17544322	-	1602	4	P	5059.416	0.381	0.083
Chr10_2571	UG	LH	17546969	+	1815	5	P	2445.398	0.222	0.091
Chr10_2572	EG	LH	17550008	+	1805	4	P	3968.057	0.357	0
Chr10_2573	EG	HH	17552652	-	9293	17	P	1282.407	0.2	0.079
AK109476	CG	HH	17565473	-	2514	4	P	1918.882	0.222	0.2
Chr10_2575	EG	LH	17570842	-	5025	12	P	2724.942	0.324	0.054
Chr10_2576	EG	HH	17579990	+	2554	3	P	1896.488	0.404	0.111
AK106383	CG	LH	17583006	-	1795	3	P	2068.883	0.244	0
Chr10_2577	EG	HH	17583769	+	982	3	P	3200.629	0.263	0.333
Chr10_2578	EG	LH	17585961	-	2376	5	A	296.913	0.05	0.067
Chr10_2579	EG	HH	17590320	+	3521	12	P	1842.934	0.216	0
AK100638	CG	HH	17598583	-	3785	8	P	3633.946	0.091	0.1
AK065202	CG	LH	17604243	-	1494	5	P	2700.919	0.216	0
AK068613	CG	HH	17613669	+	8014	17	P	2376.945	0.298	0.12
Chr10_2583	UG	LH	17626872	+	506	2	A	0	0	0.333

Chr10_2584	UG	LH	17629450	+	480	1	P	5064.254	0.364	0
Chr10_2585	UG	HH	17631370	-	5558	9	P	2150.849	0.048	0.043
AK108599	CG	HH	17637872	+	2984	10	P	926.315	0.105	0
AK071193	CG	HH	17642849	+	1663	4	A	302.522	0.083	0
AK106067	CG	HH	17644794	-	3900	7	P	3461.825	0.359	0.043
Chr10_2589	UG	LH	17660224	-	3065	3	P	1751.762	0.235	0.242
Chr10_2590	UG	HH	17664330	+	3415	11	P	2303.417	0.139	0.026
AK072452	CG	HH	17675151	+	5667	11	P	3640.798	0.18	0.016
Chr10_2592	UG	HH	17684849	+	3106	3	P	1537.158	0.171	0
Chr10_2593	EG	HH	17688630	-	4960	18	P	3159.461	0.059	0
Chr10_2594	EG	LH	17694257	+	6896	4	P	4480.805	0.313	0.107
AK066573	CG	HH	17703268	+	6033	8	P	1229.57	0.191	0.063
AK101819	CG	HH	17715344	-	3498	5	P	1269.244	0.256	0.237
Chr10_2597	UG	LH	17719154	-	341	2	P	5323.782	0.429	0
Chr10_2598	UG	HH	17720170	-	3541	9	P	3566.265	0.189	0.12
AK070563	CG	LH	17733476	-	1483	2	P	2057.65	0.29	1
Chr10_2600	UG	LH	17737264	-	4931	7	P	2470.759	0.25	0.136
Chr10_2601	UG	HH	17743295	-	805	2	P	883.294	0.125	0
Chr10_2602	UG	HH	17744985	+	6111	7	P	1661.27	0.067	0
Chr10_2603	UG	HH	17751529	-	937	2	P	1321.805	0.15	0
AK103856	CG	HH	17754257	-	5469	10	P	2442.404	0.169	0
Chr10_2605	EG	HH	17763359	-	3635	4	P	2485.569	0.195	0.077
Chr10_2606	EG	HH	17768919	-	4842	13	P	3701.455	0.15	0.043
Chr10_2607	EG	HH	17774611	-	11485	25	P	1622.784	0.112	0.05
Chr10_2608	EG	HH	17788007	+	2750	3	P	2632.924	0.214	0.087
Chr10_2609	UG	LH	17791833	-	822	1	P	1756.834	0.222	0
Chr10_2610	UG	LH	17801461	-	678	1	P	1725.259	0.467	0
Chr10_2611	UG	LH	17803541	-	3244	5	P	3193.226	0.417	0.048
AK068143	CG	HH	17816787	+	6469	18	P	1169.868	0.205	0.071
AK108318	CG	HH	17836317	+	3291	7	P	2461.68	0.242	0.079
Chr10_2616	UG	HH	17852165	+	1539	2	P	1921.897	0.031	0
Chr10_2617	UG	LH	17855812	-	790	2	P	3855.263	0.385	0.4
AK064770	CG	HH	17858829	+	6990	13	P	2325.038	0.098	0
Chr10_2619	UG	HH	17869814	+	2376	1	A	492.663	0.019	0
AK107213	CG	LH	17886141	+	1911	5	P	919.762	0.091	0
Chr10_2622	EG	HH	17908432	+	3733	8	P	1146.165	0.244	0.073
AK071225	CG	HH	17913006	+	3057	6	P	1857.876	0.282	0.038
Chr10_2624	UG	HH	17918440	+	3642	7	P	3465.965	0.25	0.041
Chr10_2625	UG	HH	17925093	-	793	3	P	944.364	0.063	0
Chr10_2626	EG	HH	17926040	+	1500	4	P	737.945	0.143	0.1
AK071470	CG	HH	17928127	-	1803	6	P	992.638	0.069	0
Chr10_2628	EG	HH	17931154	+	3535	8	P	1204.764	0.135	0.049
AK060338	CG	HH	17936170	+	1954	6	P	733.847	0.133	0
Chr10_2630	EG	HH	17940868	+	2266	6	P	1236.204	0.12	0.083
Chr10_2631	UG	LH	17943989	+	1446	4	P	2573.411	0.125	0.043
AK071378	CG	HH	17944729	-	2685	2	P	1680.533	0.125	0
AK102105	CG	HH	17954649	+	5485	9	P	1245.837	0.141	0
AK058916	CG	LH	17966530	+	1694	5	A	290.542	0.071	0.042
AK108732	CG	HH	17968590	-	2357	6	P	3582.404	0.265	0.059
Chr10_2636	UG	HH	17978219	-	1998	6	P	1864.389	0.32	0.263
AK106856	CG	LH	17999524	+	1025	1	P	2791.922	0.304	0
AK061011	CG	HH	18009025	+	1065	1	P	935.873	0.333	0
AK069398	CG	HH	18010932	+	2497	2	P	4452.089	0.019	0
AK100791	CG	LH	18016929	+	3256	11	A	323.278	0.024	0.091
AK111394	CG	LH	18020371	-	7553	5	A	534.449	0.1	0.096
AK099535	CG	LH	18020825	+	4194	4	P	3067.237	0.115	0.235
Chr10_2643	UG	HH	18028216	-	1940	3	P	1310.904	0.45	0
Chr10_2644	EG	HH	18035332	+	2362	5	P	2864.514	0.211	0.152
AK110769	CG	LH	18037322	-	1404	1	A	0	0	0
AK109295	CG	HH	18038942	-	736	4	P	2400.637	0.438	1
AK101839	CG	LH	18040762	+	3278	6	P	2533.044	0.3	0.073
AK103175	CG	HH	18048492	+	2966	6	P	1569.998	0.263	0.2

AK067982	CG	HH	18052634	+	3783	4	P	1786.422	0.267	0.077
Chr10_2649	EG	HH	18058030	+	3544	6	P	916.848	0.138	0.2
Chr10_2650	UG	LH	18062632	-	1727	3	A	0	0	0
AK071551	CG	HH	18065014	+	1659	8	P	1465.526	0.205	0
Chr10_2652	EG	HH	18068183	+	1625	2	P	1890.919	0.143	0
AK058495	CG	HH	18085407	+	3475	3	P	3296.515	0.323	0.174
Chr10_2654	UG	HH	18099288	-	1365	1	P	955.584	0.129	0
Chr10_2655	EG	LH	18103795	+	591	2	P	2573.899	0.222	0
Chr10_2656	UG	HH	18105010	+	560	2	P	2473.892	0.333	0
AK099827	CG	HH	18106008	-	2666	4	P	3249.702	0.423	0
AK063028	CG	LH	18110064	+	615	3	P	1590.135	0.5	0
Chr10_2659	UG	LH	18112354	-	1963	3	A	560.175	0.083	0.065
AK099393	CG	HH	18116953	-	3219	8	P	2707.006	0.269	0.045
Chr10_2661	EG	HH	18124623	-	1957	3	P	2160.492	0.313	0
Chr10_2662	UG	LH	18129475	+	320	2	P	3009.962	0.333	0
AK072055	CG	HH	18131631	-	4374	12	P	965.68	0.333	0.021
Chr10_2664	UG	HH	18136305	-	9830	17	P	1406.133	0.074	0.061
AK072008	CG	HH	18147124	-	2065	3	P	1949.236	0.156	0
AK071258	CG	HH	18150795	-	47299	3	P	1771.889	0.139	0.117
AK072520	CG	HH	18159398	-	1982	7	P	4405.033	0.156	0
AK072481	CG	HH	18162233	-	5180	16	P	3311.524	0.161	0.103
Chr10_2669	EG	HH	18174299	+	1428	1	P	3321.798	0.25	0
Chr10_2670	UG	LH	18176540	+	659	2	P	2155.67	0.111	0
AK101024	CG	HH	18179381	+	5377	20	P	1239.867	0.227	0.12
Chr10_2672	UG	HH	18185754	+	2771	5	P	1110.678	0.145	0
AK107822	CG	HH	18189766	-	861	2	P	2970.696	0.3	0
AK109284	CG	LH	18189813	+	981	3	P	5034.901	0.273	0
Chr10_2674	EG	HH	18194844	-	2753	4	P	3568.003	0.333	0.14
AK103857	CG	HH	18203887	-	2942	12	P	1983.003	0.194	0.069
AK073085	CG	HH	18210029	-	5441	11	P	5037.397	0.145	0.079
AK073955	CG	LH	18217297	-	1781	4	P	2717.825	0.28	0.071
Chr10_2677	UG	LH	18224009	+	1059	2	P	3131.376	0.444	0.167
Chr10_2678	UG	LH	18227493	+	270	1	P	4711.808	0.571	0
AK111107	CG	HH	18228081	-	2446	5	P	1169.415	0.08	0
AK070800	CG	LH	18232872	+	14022	8	P	773.43	0.088	0.068
AK064441	CG	LH	18252198	+	1857	4	P	2403.273	0.25	0
Chr10_2681	UG	HH	18261156	-	297	1	P	5332.996	0.429	0
AK063117	CG	LH	18265391	+	543	2	P	4613.412	0.167	0
Chr10_2683	UG	LH	18268566	-	1073	3	P	3055.182	0.231	0.25
Chr10_2684	EG	HH	18276937	-	300	1	P	5157.368	0.429	0
Chr10_2685	EG	HH	18280079	-	282	1	A	465.463	0.286	0
Chr10_2686	UG	HH	18285594	-	300	1	P	15982.501	0.143	0
Chr10_2687	UG	LH	18294924	+	1455	3	P	2050.465	0.571	0.333
AK062588	CG	LH	18297365	+	1934	5	P	3108.327	0.27	0.2
Chr10_2689	UG	HH	18301259	+	1527	1	P	3559.821	0.212	0
Chr10_2690	EG	LH	18304259	-	851	2	P	4185.773	0.4	0.111
Chr10_2691	UG	HH	18307372	+	6884	21	P	1154.365	0.093	0.133
Chr10_2692	UG	HH	18315096	+	15333	19	P	2707.431	0.352	0.152
AK067969	CG	HH	18331348	-	10089	18	P	2188.14	0.107	0.133
Chr10_2695	UG	LH	18341915	+	429	1	P	1584.583	0.3	0
Chr10_2696	UG	HH	18342910	-	1196	4	P	12994.889	0.1	0.231
Chr10_2697	UG	HH	18347515	-	4664	4	P	1334.023	0.152	0
Chr10_2698	UG	HH	18357826	+	5743	8	P	1570.913	0.114	0.057
Chr10_2699	UG	HH	18370492	+	8693	13	P	1798.427	0.102	0.121
Chr10_2700	UG	LH	18379836	-	1726	3	P	3891.271	0.286	0.077
Chr10_2701	UG	HH	18384104	-	4038	3	P	2019.016	0.132	0.059
Chr10_2702	UG	LH	18390043	+	848	2	P	3488.56	0.444	0
AK058656	CG	LH	18392366	-	2952	12	P	3225.939	0.188	0.088
Chr10_2704	EG	LH	18402784	-	405	1	P	1568.637	0.444	0
AK066499	CG	HH	18408267	-	8120	15	P	1467.727	0.111	0.061
Chr10_2706	UG	HH	18418455	+	4464	8	P	3087.279	0.191	0.061
Chr10_2707	UG	LH	18423462	-	470	3	A	0	0	0

Chr10_2708	UG	HH	18431075	+	1456	2	P	3972.05	0.258	0
Chr10_2709	EG	HH	18435110	+	963	2	P	5218.465	0.235	0.5
Chr10_2710	UG	LH	18436421	-	4204	4	A	0	0	0.114
Chr10_2711	UG	LH	18443639	-	6062	5	P	1102.411	0.083	0.096
AK066207	CG	HH	18460597	+	4242	20	P	3367.529	0.13	0
Chr10_2713	EG	HH	18465161	-	7513	16	P	2054.861	0.076	0.035
AK103246	CG	LH	18473648	+	2721	6	P	2009.006	0.211	0.024
AK060639	CG	HH	18478139	-	2163	2	P	1485.852	0.407	0.05
AK110739	CG	LH	18487785	-	895	1	P	1145.74	0.15	0
AK105809	CG	HH	18496193	+	5251	14	P	1569.759	0.077	0.047
Chr10_2719	UG	LH	18503729	-	620	2	P	3273.366	0.273	0
AK067663	CG	HH	18509583	+	2426	5	P	944.47	0.174	0
Chr10_2721	EG	HH	18515508	+	3201	8	P	3233.34	0.125	0.022
Chr10_2722	UG	LH	18522327	-	689	2	P	3715.191	0.5	0
Chr10_2724	UG	LH	18536632	+	288	1	P	2505.987	0.286	0
AK059482	CG	LH	18547474	+	1288	2	P	2657.625	0.167	0
AK105348	CG	LH	18548831	-	705	2	P	861.286	0.25	0
AK107924	CG	LH	18557195	-	641	2	P	1568.308	0.063	0
Chr10_2726	UG	LH	18557505	+	2486	3	P	1253.174	0.375	0.024
AK070531	CG	HH	18565313	+	3639	7	P	1797.933	0.278	0.093
Chr10_2728	UG	LH	18570998	+	816	1	P	3835.044	0.353	0
Chr10_2729	UG	HH	18574010	+	2958	2	P	2131.572	0.35	0.07
Chr10_2730	UG	HH	18578412	-	1128	1	P	3565.823	0.44	0
AK100601	CG	HH	18582558	+	4569	6	P	1412.645	0.177	0.081
AK109491	CG	HH	18592165	-	1003	4	P	2592.055	0.308	0
Chr10_2732	UG	LH	18592526	+	3623	4	P	4831.444	0.438	0.148
Chr10_2733	EG	HH	18600487	-	4454	12	A	502.019	0.026	0.017
Chr10_2734	UG	LH	18608566	-	405	1	P	3229.634	0.3	0
Chr10_2735	UG	HH	18632287	+	2128	3	P	1971.949	0.313	0
Chr10_2736	UG	LH	18636936	-	657	1	P	2198.012	0.533	0
AK070496	CG	HH	18637923	+	5244	14	P	1645.007	0.113	0.016
Chr10_2738	UG	LH	18644088	-	405	1	P	1803.165	0.3	0
AK073197	CG	HH	18648171	+	4939	16	P	1162.348	0.078	0
AK060521	CG	LH	18670610	+	1807	3	P	1046.069	0.053	0
Chr10_2741	UG	HH	18674607	-	4267	15	P	2140.842	0.17	0.026
AK067847	CG	HH	18687086	-	6221	4	P	2615.785	0.149	0.037
AK067203	CG	LH	18705620	-	2304	2	P	3275.06	0.212	0
AK067223	CG	HH	18710056	-	3522	11	P	3864.534	0.163	0.118
Chr10_2746	EG	HH	18714891	-	11454	24	P	870.507	0.151	0.037
Chr10_2747	UG	LH	18734026	-	808	3	P	1603.692	0.5	0.167
Chr10_2748	UG	HH	18737053	-	6053	3	P	1960.946	0.182	0.061
Chr10_2749	UG	LH	18754695	-	1200	2	P	7339.121	0.25	0.263
AK102890	CG	HH	18762545	+	2647	9	P	2529.023	0.14	0.167
Chr10_2751	UG	HH	18766019	+	1606	2	A	534.584	0.118	1
Chr10_2752	UG	LH	18771606	-	1053	2	P	1244.183	0.125	0.067
Chr10_2753	EG	HH	18776270	+	1569	1	P	2060.436	0.324	0
Chr10_2754	UG	HH	18779857	+	597	1	P	2583.604	0.286	0
Chr10_2755	UG	HH	18784406	+	1560	1	P	2475.185	0.286	0
AK070291	CG	HH	18787066	-	3284	9	P	2492.561	0.244	0.111
Chr10_2757	UG	HH	18792202	+	2606	2	P	901.886	0.065	0
Chr10_2758	UG	HH	18798459	-	1539	1	P	1822.843	0.206	0
AK105678	CG	HH	18801064	+	1892	4	P	1467.13	0.163	0
Chr10_2760	UG	HH	18805360	-	1572	1	P	2162.437	0.2	0
Chr10_2761	UG	LH	18811625	+	778	2	P	3541.673	0.5	0.143
Chr10_2762	UG	LH	18813287	+	735	1	P	1616.539	0.235	0
AK106424	CG	HH	18816600	+	1858	5	P	2147.671	0.25	0
AK062851	CG	HH	18825852	+	1972	5	P	1038.109	0.381	0
Chr10_2765	EG	HH	18830651	-	1166	2	P	4863.128	0.36	0
Chr10_2766	UG	HH	18833657	-	3064	5	P	1576.457	0.164	0.143
Chr10_2767	UG	LH	18841718	-	5319	6	P	1799.676	0.209	0.232
AK065934	CG	HH	18854710	+	5411	22	P	1475.134	0.103	0.065
AK103655	CG	HH	18862371	-	1177	3	P	3371.76	0.269	0

Chr10_2770	UG	HH	18872334	+	2540	4	P	2544.006	0.222	0.047
AK059055	CG	LH	18884033	-	1554	4	P	2501.65	0.353	0
Chr10_2774	UG	HH	18911298	-	2328	2	P	6633.146	0.114	0.063
Chr10_2775	UG	LH	18917600	+	668	2	P	728.105	0.083	0
AK070811	CG	HH	18918962	+	8503	12	P	1936.458	0.179	0.077
AK107070	CG	LH	18928000	-	845	3	P	4918.719	0.167	1
Chr10_2778	UG	LH	18950443	+	450	1	P	1981.713	0.636	0
Chr10_2779	UG	LH	18953183	+	404	2	P	5400.499	0.25	0.167
Chr10_2780	EG	HH	18957566	+	3089	8	A	386.126	0.097	0.054
AK100465	CG	HH	18960841	-	3445	3	P	2018.174	0.342	0.057
AK063213	CG	LH	18970203	+	443	1	P	6126.204	0.1	0
Chr10_2782	UG	HH	18975015	-	564	1	P	4485.443	0.583	0
AK068787	CG	HH	18977238	+	3421	5	P	2197.092	0.137	0.136
AK058780	CG	HH	18981119	-	526	2	P	972.528	0.273	0
AK099732	CG	HH	18983892	+	5610	15	P	2269.211	0.189	0
AK110909	CG	LH	18990096	+	1652	1	P	1813.432	0.108	0
Chr10_2785	UG	LH	18990097	-	327	1	P	6687.34	0.125	0
Chr10_2786	UG	LH	18997720	-	799	2	P	4619.369	0.25	0.167
AK100288	CG	HH	19007915	+	9634	23	P	1549.731	0.207	0.057
AK070764	CG	HH	19017817	-	3374	9	P	2069.261	0.152	0
Chr10_2789	EG	HH	19029071	-	1608	1	P	2053.072	0.294	0
Chr10_2790	UG	LH	19034369	+	3102	4	P	916.239	0.071	0.093
AK109263	CG	LH	19034388	+	707	2	P	2291.905	0.278	0
AK103488	CG	HH	19038767	+	3237	8	P	1238.448	0.205	0.074
AK107812	CG	HH	19042625	-	944	1	P	1412.293	0.19	0
AK108172	CG	LH	19045419	+	1658	2	P	2229.614	0.162	0
Chr10_2794	UG	LH	19052249	+	1038	1	P	1728.458	0.13	0
Chr10_2795	UG	LH	19054161	-	1236	1	P	2672.552	0.143	0
Chr10_2796	UG	LH	19056369	-	566	2	P	1689.131	0.308	0
Chr10_2797	UG	LH	19060096	+	1104	1	P	1518.51	0.24	0
Chr10_2798	UG	HH	19061834	-	473	2	A	0	0	0
Chr10_2799	EG	LH	19065047	+	1124	2	P	2191.576	0.208	1
Chr10_2800	UG	LH	19074163	-	1317	3	P	819.816	0.125	0.167
Chr10_2801	UG	HH	19076292	+	2907	2	P	1968.523	0.138	0
Chr10_2802	UG	HH	19081299	+	2583	3	P	2092.918	0.216	0.25
AK071110	CG	HH	19085181	-	4323	5	P	2669.113	0.302	0.113
AK103797	CG	HH	19090437	-	4183	5	P	2466.819	0.108	0.037
Chr10_2805	UG	HH	19097807	+	2806	8	P	2229.903	0.156	0.069
Chr10_2806	UG	HH	19106182	-	3074	6	P	788.256	0.111	0.111
Chr10_2807	UG	HH	19113209	+	1041	2	P	908.442	0.267	0.143
AK108163	CG	HH	19119181	-	4576	12	P	2208.32	0.173	0.042
AK064063	CG	HH	19125573	-	1436	7	P	1294.744	0.13	0.231
Chr10_2810	EG	HH	19131017	+	6227	10	P	3690.045	0.125	0.121
Chr10_2811	UG	LH	19140167	+	960	2	P	1995.615	0.455	0
AK109013	CG	HH	19142351	+	3444	3	P	2416.059	0.25	0.04
AK105796	CG	HH	19148304	+	3684	6	P	1662.115	0.361	0
AK065223	CG	HH	19151870	-	4356	10	P	2033.323	0.17	0.024
Chr10_2815	EG	HH	19163483	-	5034	13	A	329.207	0.024	0.077
AK061038	CG	HH	19174011	+	2142	5	P	2532.406	0.241	0.056
Chr10_2817	EG	HH	19177820	-	2873	6	P	2504.314	0.122	0
Chr10_2818	UG	LH	19183479	-	861	2	P	2867.598	0.8	0
Chr10_2819	UG	LH	19186301	+	339	1	P	5247.57	0.5	0
Chr10_2820	UG	LH	19188827	+	3084	3	P	1630.333	0.357	0.263
Chr10_2821	UG	LH	19196051	-	4763	9	P	2393.692	0.164	0.116
AK071847	CG	HH	19220226	+	1378	4	P	3011.105	0.333	0
AK072155	CG	LH	19225040	+	5798	12	P	2305.086	0.137	0.094
Chr10_2825	EG	HH	19229334	+	6064	4	P	2302.033	0.229	0.152
AK068326	CG	LH	19239828	-	557	1	P	1556.961	0.308	0
AK072776	CG	LH	19245850	-	569	1	A	537.122	0.308	0
AK062244	CG	HH	19250416	+	2677	12	A	442.095	0.034	0.033
AK069466	CG	HH	19259514	+	4143	17	P	1342.941	0.293	0.058
Chr10_2830	UG	HH	19271736	+	1593	1	P	2924.467	0.257	0

Chr10_2831	UG	LH	19298513	+	1896	2	P	2439.706	0.5	0.04
Chr10_2832	UG	LH	19301501	+	981	2	P	1068.356	0.286	0.143
AK099858	CG	HH	19305020	-	1702	2	P	4960.817	0.256	0
Chr10_2834	UG	LH	19311914	-	2431	4	A	325.469	0.125	0.067
Chr10_2835	UG	LH	19317185	+	2327	3	P	4235.974	0.318	0.069
Chr10_2836	EG	HH	19320239	-	2267	5	P	4159.808	0.154	0.167
Chr10_2837	UG	HH	19325603	-	4378	3	P	1620.615	0.138	0
Chr10_2838	UG	HH	19331604	+	5955	18	P	1193.536	0.115	0.132
AK106233	CG	LH	19349340	+	4035	6	P	2643.027	0.268	0.167
AK073567	CG	HH	19353598	-	2893	6	P	1991.072	0.22	0.167
AK063244	CG	LH	19363212	+	541	2	P	4587.344	0.692	0
AK073777	CG	HH	19369796	+	2299	5	P	1890.415	0.149	0.25
AK073012	CG	HH	19372460	-	6177	13	P	1849.116	0.238	0
Chr10_2845	UG	LH	19380511	-	645	2	P	2375.039	0.071	0
AK069238	CG	HH	19382262	-	2650	2	P	1790.127	0.125	0
Chr10_2847	EG	HH	19387765	-	4209	6	P	1086.14	0.083	0.018
Chr10_2851	EG	HH	19415066	-	624	2	A	358.067	0.111	0
Chr10_2852	UG	HH	19422171	+	1073	2	P	2680.571	0.435	0
Chr10_2853	UG	LH	19425724	+	832	2	P	3096.141	0.667	0.4
Chr10_2854	EG	HH	19427103	-	3504	6	A	588.31	0.056	0.032
Chr10_2855	UG	LH	19431335	+	510	2	A	0	0	0.167
AK067309	CG	HH	19432128	-	1170	5	P	4649.986	0.148	0
Chr10_2857	UG	LH	19435054	+	899	3	P	2054.106	0.5	0
Chr10_2858	UG	HH	19436151	-	851	2	P	1834.672	0.176	0
Chr10_2859	UG	HH	19439533	+	1827	3	P	3077.49	0.211	0.053
Chr10_2860	UG	LH	19443487	-	509	2	A	0	0	0.167
Chr10_2861	EG	HH	19450596	-	894	2	P	4345.592	0.4	0
Chr10_2862	UG	LH	19452131	-	1751	4	P	3763.938	0.111	0.158
AK107435	CG	HH	19455098	+	1264	7	P	1772.389	0.345	0
Chr10_2864	UG	LH	19456578	-	857	3	P	2935.213	0.375	0.273
Chr10_2865	UG	HH	19459766	+	1367	2	P	2423.102	0.4	0.2
AK063773	CG	HH	19463805	+	3647	7	P	3890.155	0.2	0.056
AK059927	CG	HH	19472297	+	1106	1	P	2742.865	0.8	0
AK110794	CG	HH	19481678	-	1537	1	P	1852.68	0.588	0
Chr10_2870	UG	LH	19488032	+	1804	2	P	2215.863	0.25	0
AK110508	CG	HH	19496387	-	2770	4	P	739.68	0.111	0
Chr10_2872	EG	HH	19501809	+	767	2	P	6195.053	0.25	0
Chr10_2873	UG	HH	19504368	+	711	2	P	3651.197	0.25	0.125
Chr10_2874	UG	LH	19506130	+	2943	4	P	2059.078	0.138	0.125
Chr10_2875	UG	LH	19512247	-	475	2	P	11132.203	0.143	0
AK059726	CG	HH	19520486	+	1222	3	P	3536.003	0.185	0
AK103309	CG	HH	19523796	+	1082	4	P	2850.117	0.292	0
Chr10_2878	UG	LH	19528043	-	828	1	P	2252.157	0.158	0
Chr10_2879	UG	HH	19529843	+	3144	8	P	2351.575	0.222	0.367
Chr10_2880	UG	HH	19534865	+	1104	3	P	2820.172	0.111	0
Chr10_2881	UG	HH	19537156	+	845	2	P	2985.53	0.294	0
Chr10_2882	UG	LH	19538735	+	716	2	P	1831.321	0.333	0
AK103453	CG	HH	19541459	+	1220	4	P	1902.745	0.32	0
AK061304	CG	HH	19544650	+	1026	2	P	2447.527	0.318	0
AK102196	CG	HH	19559638	+	1037	2	P	2549.483	0.227	0
Chr10_2886	EG	HH	19562158	+	831	2	P	2875.697	0.353	1
Chr10_2887	EG	HH	19564215	+	793	2	P	3522.439	0.214	0.25
Chr10_2888	UG	HH	19566999	+	831	2	P	2250.44	0.25	0
Chr10_2889	UG	HH	19570474	+	798	2	P	1038.192	0.118	0
Chr10_2891	UG	HH	19580247	+	772	2	P	1293.421	0.25	0
AK105926	CG	HH	19582792	-	1148	2	P	7371.778	0.208	0.5
Chr10_2893	EG	HH	19586091	-	795	1	P	5199.988	0.444	0
AK109314	CG	HH	19592286	+	896	2	P	1579.26	0.25	0
Chr10_2895	UG	HH	19594040	+	852	1	P	3813.244	0.211	0
AK111429	CG	HH	19596191	-	1395	1	P	3061.493	0.161	0
AK059760	CG	HH	19596762	+	1043	3	P	962.167	0.32	0
AK107032	CG	HH	19602869	+	1257	2	P	3986.834	0.222	0

Chr10_2898	UG	HH	19609096	+	2843	6	P	3578.145	0.132	0
Chr10_2899	UG	LH	19612527	-	2029	3	P	1500.986	0.32	0.211
AK108900	CG	LH	19615195	-	967	2	P	4138.816	0.182	0
AK061233	CG	HH	19615375	+	951	2	P	1690.801	0.143	0
Chr10_2901	UG	LH	19616850	+	591	2	P	3086.587	0.5	0
Chr10_2902	UG	HH	19620907	-	2409	1	P	2072.33	0.226	0
Chr10_2903	EG	LH	19627896	-	1003	2	P	4611.178	0.471	0.333
AK108607	CG	HH	19629223	-	2560	6	P	1663.04	0.27	0
AK106313	CG	HH	19644936	+	6432	4	P	2382.282	0.258	0.04
AK065403	CG	LH	19656609	+	4033	3	P	1295.036	0.16	0
AK107095	CG	HH	19667660	-	3636	4	P	1597.864	0.227	0.167
AK069934	CG	LH	19677200	-	1331	3	P	3183.515	0.261	0.143
AK060309	CG	LH	19689690	+	1060	1	P	3093.561	0.333	0
Chr10_2911	UG	HH	19696872	+	771	1	A	470.849	0.118	0
Chr10_2912	UG	LH	19698096	-	3396	4	P	4596.769	0.077	0.049
Chr10_2913	UG	HH	19703544	+	4604	8	P	1048.905	0.136	0.078
AK099073	CG	HH	19708350	-	914	1	P	3480.405	0.238	0
Chr10_2915	EG	HH	19710205	-	2664	7	P	2259.121	0.308	0
AK106036	CG	HH	19715790	+	1326	3	P	2950.656	0.267	0
Chr10_2917	UG	HH	19722891	+	1796	5	P	3381.684	0.375	0.063
Chr10_2918	EG	HH	19734517	+	2893	6	P	1479.027	0.138	0.088
Chr10_2919	UG	LH	19737761	-	1290	3	P	2848.468	0.111	0
Chr10_2920	UG	HH	19742307	+	2025	1	P	1017.998	0.133	0
AK104667	CG	HH	19746633	+	3543	5	P	1570.552	0.286	0.08
AK071890	CG	HH	19753124	-	3949	10	P	3230.486	0.085	0.098
AK105216	CG	LH	19760119	-	1249	2	P	2989.018	0.185	0
AK070172	CG	HH	19764545	-	4477	5	P	1383.173	0.125	0.5
AK103845	CG	LH	19766407	+	3005	2	P	2869.794	0.149	0
Chr10_2925	UG	HH	19770324	-	1897	3	P	4518.077	0.296	0.25
AK068029	CG	HH	19774863	-	4709	6	P	2850.495	0.236	0.063
Chr10_2927	UG	LH	19785741	+	770	2	P	2481.859	0.647	0
Chr10_2928	UG	LH	19789135	-	1156	2	P	2312.105	0.429	0.579
Chr10_2929	UG	LH	19797026	+	857	2	P	1982.049	0.583	0
Chr10_2930	UG	HH	19801847	-	4850	5	P	1085.827	0.125	0.04
Chr10_2931	UG	LH	19807305	-	1706	3	P	1238.5	0.067	0.25
Chr10_2932	UG	LH	19809635	+	555	1	P	3220.34	0.385	0
Chr10_2933	UG	HH	19823051	+	868	3	P	2932.952	0.333	0.286
AK105227	CG	HH	19823239	+	3189	6	P	1038.374	0.364	0.229
Chr10_2935	UG	HH	19826964	-	1224	2	P	1296.114	0.278	0
Chr10_2936	EG	HH	19829559	-	1733	8	P	1459.742	0.211	0.053
Chr10_2937	EG	LH	19847829	-	731	2	P	2796.126	0.25	0
AK070135	CG	HH	19850830	+	10468	9	P	1724.641	0.294	0.063
AK061357	CG	HH	19874028	+	6248	6	P	817.422	0.385	0.052
Chr10_2940	EG	HH	19878274	+	1619	3	P	3703.403	0.25	0
AK066849	CG	LH	19881822	+	1515	6	P	2877.926	0.3	0
Chr10_2942	UG	HH	19883829	+	1127	3	P	2249.357	0.174	0.5
AK105708	CG	HH	19888260	+	2212	4	P	4135.419	0.242	0.125
Chr10_2944	UG	HH	19905089	-	939	1	P	4622.63	0.238	0
AK068850	CG	LH	19916584	-	4951	5	P	1063.918	0.148	0.037
Chr10_2946	UG	HH	19922070	+	672	2	P	2484.301	0.429	0
AK101318	CG	HH	19923524	+	3864	12	P	2422.352	0.194	0.083
AK103560	CG	HH	19927727	-	4464	6	P	2147.329	0.11	0
Chr10_2949	UG	HH	19943366	+	1194	1	A	420.667	0.038	0
Chr10_2950	UG	LH	19948862	-	1183	2	P	7744.155	0.2	0
AK061277	CG	HH	19953742	+	1435	7	P	1682.712	0.139	0
Chr10_2953	UG	HH	19961265	+	1149	1	P	1135.424	0.12	0
Chr10_2954	UG	HH	19965243	+	1242	1	P	4085.401	0.074	0
Chr10_2955	UG	HH	19972741	+	1194	1	A	477.904	0.038	0
Chr10_2956	UG	HH	19976304	+	1191	1	A	548.386	0.115	0
Chr10_2957	UG	HH	19983158	+	1260	1	P	1135.681	0.036	0
Chr10_2958	UG	LH	19985111	+	584	2	A	0	0	0
Chr10_2959	UG	HH	19987767	+	1123	2	P	1126.626	0.167	0

Chr10_2962	UG	HH	20003826	+	1218	1	A	0	0	0
AK071222	CG	HH	20008444	-	1739	2	P	2126.475	0.385	0
AK059098	CG	HH	20011165	-	527	2	P	4316.438	0.5	0
Chr10_2965	UG	HH	20012418	+	1231	2	A	0	0	0.273
AK065456	CG	HH	20019210	+	4749	8	P	1458.174	0.635	0.078
Chr10_2967	EG	HH	20028025	+	2193	1	P	1358.27	0.208	0
Chr10_2968	UG	LH	20031082	-	1683	2	P	882.986	0.125	0.069
AK102640	CG	HH	20040921	+	4241	9	P	1831.04	0.281	0.103
AK063094	CG	LH	20047864	-	1084	3	P	3071.445	0.727	0.333
AK074016	CG	HH	20050708	-	3196	5	P	2831.414	0.179	0.25
Chr10_2972	EG	LH	20054812	+	713	3	P	2472.978	0.154	0
Chr10_2973	UG	LH	20066335	-	800	2	P	1748.226	0.455	0.2
AK069384	CG	HH	20067971	+	3216	4	P	1519.211	0.034	0.429
Chr10_2975	UG	HH	20073198	-	1077	1	P	2305.249	0.12	0
Chr10_2976	UG	LH	20075688	+	348	1	P	3899.877	0.444	0
Chr10_2977	UG	LH	20078648	+	2414	3	P	2147.954	0.133	0.029
Chr10_2978	UG	LH	20082249	+	474	1	P	2857.809	0.455	0
AK069597	CG	HH	20085324	+	4611	9	P	2443.143	0.128	0.087
AK072078	CG	LH	20098372	+	3814	9	P	4242.463	0.146	0.059
AK072272	CG	HH	20102719	-	4763	19	P	1401.89	0.186	0
AK063496	CG	HH	20114592	+	3740	7	P	2772.883	0.13	0.111
Chr10_2983	UG	LH	20121059	-	4747	6	P	2705.997	0.24	0.11
Chr10_2984	UG	HH	20131208	-	3211	10	P	780.976	0.135	0.032
Chr10_2985	UG	HH	20141735	+	1948	3	P	2328.921	0.087	0.05
AK111392	CG	LH	20143705	-	769	1	P	3559.111	0.059	0
Chr10_2987	UG	HH	20158933	+	246	1	P	5917.18	0.333	0
Chr10_2992	UG	LH	20179105	+	528	1	A	570.771	0.333	0
AK063504	CG	HH	20181492	+	739	3	P	2616.877	0.688	0
AK065246	CG	HH	20182715	-	4823	8	P	3935.124	0.196	0.093
AK068088	CG	HH	20192537	+	1733	5	P	1619.123	0.31	0.222
Chr10_2996	UG	HH	20205149	+	936	1	P	2319.518	0.182	0
Chr10_2997	EG	HH	20214704	+	4547	9	P	2748.732	0.279	0.057
Chr10_2998	EG	HH	20220644	+	1859	3	P	682.676	0.158	0.053
Chr10_2999	UG	LH	20224452	+	1126	3	P	1379.414	0.167	0.105
AK106264	CG	HH	20227715	+	2186	6	P	1994.933	0.176	0.083
Chr10_3001	UG	LH	20230265	-	1190	2	P	4600.356	0.4	0.133
Chr10_3002	UG	LH	20234023	+	999	1	P	2037.577	0.227	0
Chr10_3003	EG	HH	20236500	-	1756	4	P	1281.952	0.44	0.231
AK063669	CG	LH	20249848	-	2368	5	P	1644.971	0.222	0
AK073941	CG	HH	20258735	+	2868	5	P	1704.315	0.325	0.348
Chr10_3007	UG	LH	20274012	+	346	2	A	0	0	0
Chr10_3008	UG	HH	20276941	+	4649	7	P	2090.922	0.24	0.066
Chr10_3009	EG	HH	20292898	+	3347	4	P	2547.643	0.148	0.065
AK069472	CG	HH	20297866	-	2939	3	P	862.288	0.179	0.038
Chr10_3011	EG	HH	20301109	+	3182	10	P	1130.887	0.154	0.136
AK061779	CG	HH	20307624	+	2839	7	P	1327.754	0.219	0.063
AK065156	CG	HH	20310853	-	3641	5	P	1993.452	0.273	0.104
Chr10_3014	UG	LH	20315480	+	2992	4	P	1525.909	0.067	0.029
Chr10_3015	EG	HH	20319350	+	1771	4	P	2329.874	0.19	0.059
Chr10_3016	UG	HH	20322287	-	4201	11	P	2775.552	0.167	0.02
AK073024	CG	LH	20326792	+	2854	3	P	2037.888	0.156	0
AK103222	CG	HH	20330607	+	1164	1	P	2513.428	0.231	0
Chr10_3020	UG	HH	20337875	+	1120	2	P	1220.672	0.25	0
Chr10_3021	EG	HH	20341577	+	6753	14	P	2024.481	0.114	0.014
AK103033	CG	HH	20350646	+	9181	27	P	1674.543	0.16	0.07
AK069919	CG	LH	20360736	-	866	1	P	2129.972	0.263	0
AK065689	CG	HH	20368969	+	4713	11	P	927.389	0.106	0.038
Chr10_3025	UG	LH	20374127	-	1288	2	P	1414.199	0.5	0.231
Chr10_3026	UG	LH	20376106	-	1310	4	P	1150.756	0.3	0.053
Chr10_3028	UG	HH	20386075	+	2008	2	P	2918.512	0.333	0
Chr10_3029	UG	HH	20392358	-	8467	8	P	1687.755	0.057	0.038
Chr10_3030	UG	LH	20405389	+	1741	3	A	0	0	0.103

AK060129	CG	LH	20407928	-	1084	3	P	1062.935	0.231	0
AK070040	CG	HH	20416994	+	2541	3	P	2620.281	0.348	0.111
Chr10_3033	UG	HH	20424038	-	3971	5	P	7358.442	0.1	0.079
Chr10_3034	UG	LH	20431866	-	1926	3	A	0	0	0.094
Chr10_3035	UG	HH	20434311	-	4349	10	P	1263.087	0.034	0.111
AK068549	CG	HH	20440909	-	3347	11	P	3592.666	0.054	0
AK107224	CG	LH	20444982	-	1141	1	P	956.733	0.308	0
AK073764	CG	HH	20454535	-	5671	8	A	542.701	0.017	0.03
Chr10_3039	EG	HH	20460484	+	483	1	P	6685.847	0.364	0
Chr10_3040	UG	LH	20462817	-	1461	2	P	6324.72	0.286	0.235
AK070187	CG	HH	20465895	+	1790	5	P	1359.744	0.333	0
AK065488	CG	HH	20468579	-	4443	8	P	1391.429	0.095	0
AK072270	CG	HH	20474458	-	3649	3	P	3063.707	0.273	0.333
Chr10_3044	UG	HH	20480282	-	2603	7	P	4597.945	0.129	0.077
AK067162	CG	HH	20483609	-	16881	5	P	2305.261	0.293	0.163
Chr10_3046	UG	LH	20489018	+	2277	3	P	1635.512	0.188	0.207
Chr10_3047	UG	LH	20492964	-	1107	2	P	3050.384	0.3	0
AK100377	CG	HH	20501031	-	2924	9	A	750.642	0.061	0.032
AK070309	CG	LH	20507872	-	873	1	A	428.916	0.053	0
Chr10_3052	UG	HH	20516525	+	2016	4	P	2502.508	0.029	0
Chr10_3056	EG	HH	20530290	+	4586	10	P	849.927	0.194	0.079
Chr10_3057	EG	LH	20536013	+	17783	21	P	1915.334	0.082	0.081
AK065729	CG	HH	20560882	+	5199	7	P	2368.503	0.129	0.034
Chr10_3059	EG	HH	20572046	+	11436	18	P	1553.938	0.049	0.029
AK067767	CG	HH	20586446	-	4894	8	P	2280.262	0.118	0.07
AK066785	CG	LH	20598279	+	2239	5	P	3677.238	0.286	0.048
Chr10_3062	UG	HH	20601104	-	2625	6	P	2868.027	0.25	0.048
AK109724	CG	LH	20608834	+	1447	1	P	2279.957	0.156	0
AK064698	CG	HH	20610383	-	5072	14	P	2451.745	0.059	0.033
Chr10_3065	UG	HH	20617582	+	8218	9	P	2672.679	0.167	0.14
Chr10_3066	UG	HH	20628641	-	758	2	P	5709.56	0.111	0.375
Chr10_3067	UG	LH	20630818	+	1059	2	A	0	0	0.105
Chr10_3068	EG	HH	20634291	+	2035	3	P	2922.817	0.294	0.2
AK106259	CG	LH	20639568	+	6442	7	P	2215.533	0.143	0.059
AK106239	CG	LH	20651103	-	3119	4	P	2636.818	0.196	0.333
Chr10_3071	UG	HH	20661254	-	5771	4	P	1474.093	0.167	0.167
AK062858	CG	HH	20669714	+	2948	4	P	1499.656	0.389	0.089
AK107851	CG	LH	20673158	-	758	1	P	7153.19	0.118	0
Chr10_3074	EG	HH	20676010	-	462	1	P	949.341	0.364	0
AK102086	CG	LH	20680109	-	748	4	P	3206.372	0.105	0
Chr10_3076	UG	LH	20692355	+	483	1	P	1218.124	0.273	0
Chr10_3077	EG	HH	20694617	-	396	1	P	2707.626	0.2	0
Chr10_3078	EG	HH	20697969	-	396	1	A	587.991	0.222	0
Chr10_3079	EG	HH	20701203	-	411	1	P	2479.552	0.222	0
Chr10_3080	UG	HH	20705383	+	6513	12	P	824.153	0.118	0.056
Chr10_3081	EG	HH	20712076	-	240	1	P	1182.938	0.167	0
AK062381	CG	HH	20715017	-	702	3	P	2112.371	0.471	0
AK068963	CG	HH	20718677	-	4047	14	P	2294.373	0.171	0.064
AK069361	CG	HH	20724993	-	3911	12	P	1089	0.06	0.088
Chr10_3085	UG	HH	20736016	-	1223	3	P	5466.294	0.273	0
AK100717	CG	LH	20741280	+	5679	6	P	715.31	0.172	0.045
Chr10_3087	UG	LH	20752899	+	684	1	P	2915.756	0.25	0
AK071048	CG	HH	20762480	+	2509	8	P	1209.994	0.289	0
AK068409	CG	HH	20768152	-	4134	7	P	2784.96	0.26	0
Chr10_3091	UG	HH	20784598	-	2565	7	P	2311.552	0.314	0
Chr10_3092	UG	HH	20787894	+	1080	2	P	1128.263	0.174	0
AK063684	CG	LH	20792575	+	905	4	P	2585.044	0.429	0.5
AK059811	CG	LH	20794073	-	1967	5	P	2699.551	0.2	0.077
Chr10_3095	UG	HH	20804722	+	3172	5	P	4362.53	0.25	0.059
AK068935	CG	HH	20808374	-	1581	6	P	979.233	0.16	0.2
AK106924	CG	HH	20814899	-	1682	1	P	2413.795	0.108	0
Chr10_3098	EG	LH	20828572	+	756	1	P	2212.681	0.647	0

AK101728	CG	HH	20835712	-	1537	3	P	2116.799	0.241	0.2
AK061068	CG	HH	20843540	-	1719	3	P	1958.485	0.233	0
AK100959	CG	HH	20855435	-	1895	4	P	1196.111	0.29	0
AK101806	CG	LH	20867520	+	1883	2	P	1558.009	0.214	0
Chr10_3103	EG	LH	20871547	-	2201	4	P	2102.883	0.333	0.069
Chr10_3104	EG	LH	20874912	-	759	1	P	4209.83	0.412	0
Chr10_3108	EG	HH	20897106	-	22738	54	P	3081.923	0.074	0.079
Chr10_3109	EG	LH	20922266	+	5050	6	P	1369.075	0.313	0.043
Chr10_3110	UG	HH	20929694	-	4261	4	P	2613.681	0.101	0.042
Chr10_3111	UG	LH	20943555	-	1127	2	P	10153.538	0.4	0.263
Chr10_3112	UG	HH	20946678	-	5952	6	P	1033.82	0.072	0.016
Chr10_3115	UG	LH	20957040	+	2065	4	P	729.901	0.067	0.071
Chr10_3116	EG	LH	20959828	-	2785	3	P	1347.244	0.063	0
Chr10_3117	EG	LH	20965843	+	1345	2	P	2342.116	0.192	0
AK073483	CG	HH	20975843	+	2018	1	P	1340.213	0.178	0
Chr10_3119	EG	HH	20978009	-	1014	1	P	2243.759	0.174	0
Chr10_3120	UG	HH	20981735	+	2238	1	P	1232.097	0.122	0
Chr10_3121	UG	HH	20984726	-	801	1	P	1292.515	0.278	0
Chr10_3122	UG	HH	20987745	-	798	1	A	419.43	0.059	0
Chr10_3123	UG	HH	20989741	-	786	1	P	1857.069	0.111	0
Chr10_3124	UG	HH	20991251	-	1579	4	P	747.106	0.111	0
Chr10_3125	UG	HH	20994170	+	4573	5	P	1562.36	0.169	0.091
Chr10_3126	UG	HH	21000792	-	1831	4	P	5536.295	0.286	0.077
Chr10_3127	UG	HH	21005620	+	2719	4	P	2060.285	0.105	0.5
AK060854	CG	HH	21010424	-	2547	4	P	911.312	0.064	0.111
Chr10_3129	EG	HH	21013349	-	1582	4	A	0	0	0
Chr10_3130	UG	HH	21016378	+	471	1	P	2407.27	0.182	0
AK068525	CG	HH	21017245	-	2046	3	P	982.216	0.071	0
AK066405	CG	HH	21034396	-	1715	5	P	1036.223	0.103	0.1
AK063546	CG	LH	21034947	+	4212	3	A	406.819	0.025	0.057
AK065705	CG	HH	21036772	-	4113	7	P	1422.743	0.129	0
AK072706	CG	HH	21041538	-	3404	4	P	2005.535	0.179	0
Chr10_3134	UG	LH	21041829	+	975	2	P	908.265	0.19	0
AK066219	CG	HH	21046026	-	4659	9	P	988.914	0.302	0.052
AK068632	CG	LH	21052456	-	5616	9	P	2572.258	0.14	0.133
AK105615	CG	HH	21061397	-	3961	10	P	2432.181	0.158	0.034
AK070091	CG	HH	21068062	+	1914	2	P	1696.177	0.364	0
Chr10_3139	UG	LH	21075247	-	3572	10	P	2984.968	0.167	0.103
AK059709	CG	LH	21083330	+	329	1	A	0	0	0
Chr10_3140	UG	HH	21085937	+	1489	2	P	1179.905	0.333	0
AK069913	CG	HH	21089007	-	3474	4	P	2327.255	0.346	0.06
Chr10_3141	UG	LH	21089047	+	1958	4	A	618.016	0.083	0.161
AK109732	CG	LH	21095324	-	2666	2	P	5281.351	0.241	0.034
Chr10_3143	EG	HH	21106046	-	2953	9	P	2637.293	0.207	0.059
Chr10_3144	EG	LH	21109616	+	3933	4	P	1699.737	0.189	0.03
AK064207	CG	LH	21123060	+	1948	6	P	1691.255	0.089	0
Chr10_3145	EG	LH	21125611	-	871	2	P	7199.376	0.263	0
AK071526	CG	HH	21132554	+	3731	12	P	1450.291	0.22	0
Chr10_3147	EG	LH	21139778	-	1113	2	P	2888.899	0.565	0
AK110882	CG	HH	21144307	+	3085	5	P	2523.804	0.122	0.04
AK066634	CG	HH	21148971	-	2027	3	P	3001.753	0.14	0
AK068343	CG	HH	21151432	+	3483	4	P	2854.767	0.2	0.063
AK066961	CG	HH	21155618	-	8273	9	P	1420.665	0.263	0.121
AK099675	CG	HH	21164078	+	4781	11	P	842.456	0.127	0.091
AK065404	CG	HH	21178963	+	1490	3	P	1104.18	0.433	0
AK101939	CG	HH	21187480	-	3890	9	P	4868.602	0.333	0
AK106294	CG	HH	21193200	-	1807	3	P	985.626	0.268	0
Chr10_3157	UG	HH	21195985	+	1143	2	P	3936.174	0.083	0.071
Chr10_3158	UG	HH	21197645	-	963	1	P	3158.048	0.238	0
AK109308	CG	LH	21206107	-	554	1	P	4261.872	0.333	0
Chr10_3160	UG	LH	21212346	-	720	2	A	451.109	0.25	0.077
Chr10_3161	UG	HH	21217502	-	2173	6	P	2442.528	0.067	0.065

Chr10_3162	UG	HH	21219970	+	1605	1	P	2688.657	0.143	0
AK064321	CG	HH	21228897	+	3161	7	P	1733.401	0.171	0.029
Chr10_3164	UG	LH	21235372	+	2698	3	P	1392.201	0.2	0.074
Chr10_3165	UG	LH	21239996	-	584	2	P	7207.587	0.857	0
Chr10_3166	EG	HH	21245098	-	3596	4	P	2957.597	0.283	0.16
Chr10_3167	UG	LH	21253137	-	1280	3	A	0	0	0.048
Chr10_3168	EG	HH	21259416	+	1241	3	P	2317.147	0.35	0
AK107989	CG	LH	21260147	+	382	1	A	0	0	0
AK072479	CG	HH	21262651	+	5844	12	P	1810.518	0.179	0.069
Chr10_3170	UG	HH	21270933	+	1334	2	A	545.5	0.083	0
AK072816	CG	HH	21273872	-	7629	12	P	1159.301	0.316	0.074
Chr10_3172	EG	HH	21283525	-	4620	6	P	890.033	0.216	0.077
Chr10_3173	EG	LH	21290417	+	2714	7	P	930.9	0.087	0.081
AK101930	CG	LH	21293995	+	4420	11	P	1775.279	0.161	0.029
AK109715	CG	HH	21301086	+	1543	1	P	2064.132	0.235	0
AK072751	CG	HH	21304224	-	2411	4	P	900.281	0.333	0.03
Chr10_3177	UG	LH	21308069	-	381	1	P	1800.966	0.333	0
AK107529	CG	HH	21315720	+	1905	2	P	2750.958	0.4	0.045
Chr10_3179	EG	HH	21321685	+	3074	6	P	1794.641	0.235	0.118
AK069258	CG	LH	21325802	-	2681	7	P	2796.944	0.167	0.034
AK072487	CG	HH	21331290	+	1417	1	P	2369.939	0.467	0
AK066340	CG	HH	21335586	-	722	1	P	4155.591	0.353	0
AK109667	CG	HH	21336868	-	3622	5	P	1221.507	0.175	0.051
AK103690	CG	HH	21341818	-	2752	9	P	1501.722	0.25	0.042
Chr10_3186	EG	HH	21352901	+	1558	5	P	1907.024	0.182	0.042
Chr10_3187	EG	HH	21355366	+	13989	17	P	1071.44	0.194	0.118
Chr10_3188	EG	HH	21369655	-	4455	6	P	1645.704	0.213	0.081
Chr10_3191	UG	HH	21389292	+	668	3	A	537.162	0.286	0
AK107429	CG	HH	21395734	-	6942	13	P	1103.034	0.15	0.056
Chr10_3193	EG	LH	21435349	-	2093	4	P	2719.801	0.393	0.167
AK103864	CG	HH	21451460	+	7045	19	P	1372.807	0.077	0.053
AK109505	CG	LH	21463077	+	10557	11	P	1687.374	0.179	0.17
Chr10_3198	EG	HH	21489096	+	526	2	P	1911.798	0.091	0
Chr10_3199	EG	HH	21491103	+	1258	2	P	3155.098	0.25	0
Chr10_3200	EG	LH	21500204	+	1106	2	P	3559.52	0.44	0
Chr10_3201	EG	LH	21515805	+	3093	7	P	2130.4	0.417	0.077
AK063299	CG	HH	21520309	-	3047	4	P	2594.194	0.15	0.038
AK101687	CG	LH	21526958	+	2185	6	P	3175.308	0.222	0
Chr10_3204	UG	HH	21529624	+	1590	1	P	1632.908	0.333	0
AK103373	CG	HH	21531586	-	2349	7	P	1629.982	0.091	0
AK063367	CG	HH	21536163	-	3634	9	P	736.358	0.122	0
AK100517	CG	LH	21536825	+	3482	5	P	865.609	0.072	0
AK070829	CG	HH	21543362	+	1845	6	P	1078.866	0.086	0
AK067730	CG	HH	21545468	-	3411	9	P	1703.611	0.197	0
AK072336	CG	HH	21551841	+	2778	1	P	1541.436	0.295	0
AK105119	CG	LH	21560514	-	1251	1	P	1231.874	0.385	0
Chr10_3210	UG	LH	21565151	-	279	1	P	1562.204	0.286	0
AK061703	CG	HH	21567127	-	1791	7	A	0	0	0.143
Chr10_3212	UG	LH	21574717	-	234	1	P	3105.849	0.5	0
Chr10_3213	EG	LH	21582030	+	1805	3	P	1138.478	0.226	0.111
AK072209	CG	LH	21586176	-	1518	5	P	2858.548	0.171	0
AK111422	CG	LH	21588143	-	2836	7	P	1218.644	0.154	0
AK070073	CG	LH	21593052	+	6009	14	P	1897.325	0.145	0.135
AK100013	CG	LH	21597476	-	3238	8	P	1892.981	0.207	0.077
Chr10_3218	UG	LH	21606944	+	2548	3	P	4201.115	0.4	0.067
Chr10_3219	UG	HH	21611797	-	4128	3	A	606.398	0.061	0.026
Chr10_3220	UG	LH	21616324	-	888	2	P	1077.718	0.263	0
AK103251	CG	HH	21623104	+	1909	5	P	3848.3	0.051	0
Chr10_3222	EG	HH	21625527	+	3335	5	P	1362.665	0.158	0.093
AK059371	CG	HH	21629386	-	826	3	A	649.807	0.059	0
AK067998	CG	HH	21630510	-	4369	13	P	1890.449	0.224	0.033
AK072459	CG	HH	21636029	+	4359	7	P	1480.855	0.158	0.056

Chr10_3225	EG	LH	21642375	+	1002	2	A	0	0	0
Chr10_3226	EG	LH	21648428	+	527	2	P	9365.482	0.286	0
Chr10_3227	UG	LH	21649637	+	368	2	A	309.083	0.333	0
Chr10_3228	UG	HH	21657371	+	1172	3	P	917.452	0.125	0.111
Chr10_3229	EG	HH	21659204	-	2065	6	P	1565.015	0.231	0.211
Chr10_3230	UG	LH	21664451	+	306	2	A	0	0	0
AK063029	CG	LH	21673402	+	528	1	P	1895.45	0.25	0
Chr10_3231	UG	LH	21674848	-	327	1	P	1320.896	0.25	0
Chr10_3233	UG	LH	21681241	+	849	1	P	2464.79	0.2	0
Chr10_3234	UG	LH	21683070	-	1454	3	P	2441.549	0.385	0
Chr10_3235	UG	LH	21686574	-	819	1	P	2069.284	0.211	0
AK070664	CG	LH	21687675	+	4019	5	P	5940.341	0.111	0.043
Chr10_3237	UG	LH	21691702	-	156	1	A	652.392	0.25	0
AK103375	CG	HH	21693768	+	3012	10	P	1176.019	0.15	0.16
AK107462	CG	HH	21702215	+	4225	11	P	2305.591	0.207	0.059
AK110684	CG	LH	21718953	+	1192	2	P	1342.685	0.308	0
Chr10_3241	EG	HH	21721801	-	4154	6	P	2764.506	0.104	0.095
Chr10_3242	EG	HH	21726995	+	2117	8	P	2813.678	0.194	0
AK073002	CG	HH	21729201	-	2474	6	P	819.733	0.089	0
AK102709	CG	HH	21738461	+	4358	5	P	1792.797	0.208	0.106
Chr10_3246	UG	LH	21746085	-	1475	3	P	1140.348	0.333	0
Chr10_3247	UG	LH	21748192	-	840	1	P	2431.341	0.211	0
AK062550	CG	LH	21758293	-	722	1	P	2523.421	0.188	0
AK067954	CG	HH	21761426	-	4832	7	P	3278.408	0.089	0.1
Chr10_3250	EG	HH	21779692	-	4091	7	P	3201.831	0.263	0.059
Chr10_3251	EG	HH	21786802	-	2505	8	P	2326.706	0.095	0.057
AK058588	CG	HH	21790828	-	2136	5	P	2456.702	0.111	0.083
AK072743	CG	HH	21810728	+	38133	5	P	2571.642	0.2	0.169
AK069018	CG	HH	21814644	+	2436	5	P	1154.177	0.269	0.179
Chr10_3257	UG	LH	21817785	-	782	2	P	3565.516	0.077	0
Chr10_3258	UG	LH	21819228	-	1736	3	P	3682.972	0.267	0.217
Chr10_3259	EG	HH	21826859	+	639	1	P	1666.177	0.467	0
Chr10_3260	EG	HH	21832087	-	1718	2	P	4287.559	0.318	0
Chr10_3261	EG	HH	21834854	-	2104	2	P	2862.616	0.2	0
AK101216	CG	HH	21839357	-	2004	3	P	3440.542	0.311	0
Chr10_3264	UG	LH	21852526	-	8917	9	P	2721.966	0.27	0.042
Chr10_3265	UG	LH	21870701	-	1041	3	P	1757.093	0.333	0
AK059974	CG	HH	21875063	+	963	2	P	2577.584	0.435	0
Chr10_3266	UG	LH	21875294	-	5652	13	P	2663.952	0.268	0.061
Chr10_3267	EG	HH	21886849	-	1326	3	P	4591.079	0.444	0
AK072719	CG	LH	21893661	-	4033	9	P	1306.426	0.128	0.136
Chr10_3269	EG	HH	21913213	+	2068	2	P	2197.232	0.295	0
AK100015	CG	HH	21917352	-	9701	17	P	2042.985	0.121	0.013
AK071939	CG	HH	21964019	-	5737	17	P	3367.108	0.304	0.081
Chr10_3275	EG	HH	21973867	+	1951	5	A	0	0	0.056
Chr10_3276	UG	HH	21977159	-	8535	17	P	2406.419	0.129	0.059
Chr10_3277	UG	LH	21987839	+	1137	3	P	2064.907	0.5	0.154
AK058777	CG	LH	21992153	+	1746	4	P	2348.302	0.429	0.077
AK059148	CG	HH	21996706	-	1397	3	P	3971.237	0.188	0
Chr10_3281	UG	HH	22006594	-	1320	1	A	0	0	0
Chr10_3282	UG	HH	22008157	-	1953	1	A	0	0	0
Chr10_3283	UG	HH	22010438	-	1110	2	P	1070.72	0.125	0
Chr10_3285	UG	LH	22016109	-	1654	3	P	1424.934	0.357	0.375
AK061555	CG	LH	22022071	-	1484	2	P	3624.974	0.333	0
AK064038	CG	HH	22041519	+	8868	4	P	2809.421	0.207	0.122
Chr10_3288	UG	LH	22044960	-	891	2	P	2660.355	0.571	0.25
AK059043	CG	HH	22051041	-	2987	7	P	3209.526	0.118	0.031
Chr10_3291	UG	HH	22055172	+	3112	3	P	1933.684	0.194	0
AK106138	CG	LH	22061736	-	1319	2	P	2501.33	0.464	0
AK064295	CG	HH	22065911	-	2135	5	P	2865.694	0.471	0.133
AK068365	CG	HH	22069026	-	3170	3	P	4680.377	0.032	0.079
Chr10_3295	UG	HH	22074657	+	4419	4	P	2541.513	0.143	0.092

Chr10_3296	UG	HH	22081511	+	2442	7	P	3518.311	0.103	0.091
AK073475	CG	HH	22095761	+	7362	14	P	1602.44	0.122	0.167
AK100237	CG	HH	22103687	+	2857	8	P	2320.166	0.263	0.042
AK072769	CG	HH	22106834	-	4364	10	P	1633.646	0.219	0.03
AK065123	CG	HH	22116165	+	6172	16	P	2679.037	0.071	0.064
Chr10_3300	EG	HH	22124601	+	1711	3	P	920.311	0.259	0.091
AK110534	CG	HH	22130874	+	1233	3	P	1021.748	0.259	0
AK111426	CG	LH	22132285	-	3303	7	P	2660.543	0.222	0
Chr10_3302	UG	HH	22135589	-	1896	1	P	3287.682	0.214	0
AK071802	CG	LH	22139575	+	1617	2	P	3354.746	0.444	0
AK107128	CG	HH	22139641	-	1468	1	P	3353.835	0.344	0
AK065615	CG	LH	22141237	-	3687	8	P	1381.793	0.152	0
AK073939	CG	HH	22145338	-	2619	9	P	3419.471	0.2	0.111
Chr10_3306	EG	HH	22149087	-	3050	6	P	2950.717	0.324	0.129
AK102587	CG	HH	22155952	-	5254	14	P	2322.462	0.29	0.085
AK102009	CG	HH	22163013	-	4405	14	P	2675.745	0.123	0.025
AK103692	CG	HH	22168080	-	29576	5	P	2447.861	0.381	0.163
AK108346	CG	LH	22174612	+	1126	3	P	2716.61	0.263	0.333
Chr10_3311	EG	LH	22179346	-	1556	3	P	2305.173	0.25	0.091
AK070527	CG	HH	22181801	+	3036	5	P	2295.542	0.277	0
Chr10_3313	UG	HH	22185222	-	6466	12	P	2059.012	0.136	0.226
Chr10_3314	EG	LH	22194952	-	387	1	P	4010.128	0.667	0
Chr10_3316	UG	LH	22199048	-	4538	5	P	2125.096	0.286	0.108

[1] see main text for the classification of gene models; [2] Homology to Arabidopsis genes; [3] Starting position of the gene models on chromosome 10 of the BGI *indica* genome sequence released on 8/1/2003; [4] P, tiling array detected, A, tiling array undetected; [5] HR, hybridization rate, see main text for detail.
