# Supplementary material for: Tiling microarray analysis of rice chromosome 10 to identify the transcriptome and relate its expression to chromosomal architecture
Source: Genome Biol. 2005 May 27;6(6):R52. doi: 10.1186/gb-2005-6-6-r52 (PMC1175972; doi:10.1186/gb-2005-6-6-r52)
Supplement: Additional File 3 — Table S3: Sequence analysis of cloned UG models. Sequence analysis of cloned UG models. [file gb-2005-6-6-r52-S3.pdf]

Supplemental Table 3. Sequence analysis of cloned UG models

UG Clone	Length [1]	Identity	Strand	Exon #	Position [2]
OsJapC10-UG841-X6	475	99.6%	+	1	10669061
OsJapC10-UG1205-4	940	99.8%	-	1	22131960
OsJapC10-UG47-5	845	100.0%	+	1	19990467
OsJapC10-UG104-6	743	99.9%	-	1	14958130
OsJapC10-UG160-6	954	29.4%	-	7	5921618
OsJapC10-UG12-2	1144	100.0%	+	1	14717490
OsJapC10-UG105-3	503	99.2%	-	1	14765804
OsJapC10-UG1221-5	915	100.0%	+	2	2223357
OsJapC10-UG856-7	593	100.0%	+	1	11035986
OsJapC10-UG896-2	723	99.9%	-	4	1174086
OsJapC10-UG938-5	328	99.7%	+	2	13425611
OsJapC10-UG1219-1	636	99.8%	-	2	22682211
OsJapC10-UG25-B1	1217	99.8%	-	1	510288
OsJapC10-UG947-8	872	81.7%	-	7	13829897
OsJapC10-UG804-3	578	100.0%	+	1	9490533
OsJapC10-UG154-7	1254	99.8%	-	4	21297326
OsJapC10-UG824-1	408	100.0%	-	1	10057380
OsJapC10-UG42-1	246	100.0%	+	1	20659903
OsJapC10-UG86-7	319	93.4%	+	4	18787066
OsJapC10-UG1200-3	411	99.8%	+	2	22092040
OsJapC10-UG25-A1	1219	99.5%	+	2	510288
OsJapC10-UG67-1	584	100.0%	+	1	4099485
OsJapC10-UG196-B6	1156	99.9%	-	1	14758709
OsJapC10-UG2-1	786	100.0%	+	1	20128212
OsJapC10-UG1099-8	669	100.0%	+	1	18163261
OsJapC10-UG824-2	408	99.8%	+	1	10057380
OsJapC10-UG278-7	625	94.7%	-	4	9738946
OsJapC10-UG51-5	429	91.4%	+	5	11720871
OsJapC10-UG939-6	425	99.8%	-	1	13439328
OsJapC10-UG1194-2	1036	100.0%	-	1	2127534
OsJapC10-UG1005-A5	504	100.0%	+	1	15322815
OsJapC10-UG776-6	879	100.0%	+	1	8672186
OsJapC10-UG9-8	579	99.7%	+	2	17619810
OsJapC10-UG1186-A2	1067	100.0%	-	1	21517063
OsJapC10-UG1194-1	1036	100.0%	+	1	2127534
OsJapC10-UG25-A3	1217	99.8%	-	1	510288

OsJapC10-UG1009-7	733	95.2%	-	6	15468478
OsJapC10-UG1202-B1	590	100.0%	+	3	22126843
OsJapC10-UG956-7	737	99.7%	+	2	1343627
OsJapC10-UG51-2	429	91.4%	+	5	11720871
OsJapC10-UG1110-1	552	100.0%	+	1	18244593
OsJapC10-UG9-4	576	100.0%	-	1	17619810
OsJapC10-UG25-C1	1217	99.3%	+	3	510288
OsJapC10-UG369-2	940	99.9%	+	1	12375317
OsJapC10-UG816-6	941	98.4%	+	3	9845614
OsJapC10-UG105-1	503	99.8%	-	1	14765804
OsJapC10-UG1196-A1	1017	100.0%	-	2	2132740
OsJapC10-UG1237-A1	701	99.7%	+	1	244012
OsJapC10-UG91-2	527	99.6%	+	1	10941489
OsJapC10-UG1216-4	740	99.9%	+	2	22637991
OsJapC10-UG744-4	1092	99.8%	-	1	7626407
OsJapC10-UG747-8	36	94.4%	-	1	10288044
OsJapC10-UG1180-4	461	99.8%	+	2	19954432
OsJapC10-UG816-2	946	99.2%	-	2	9845614
OsJapC10-UG1037-4	361	99.2%	-	2	10399247
OsJapC10-UG867-1	677	90.7%	+	6	11238546
OsJapC10-UG956-6	737	100.0%	-	2	1343627
OsJapC10-UG1216-2	739	100.0%	+	3	22637991
OsJapC10-UG1009-6	733	95.4%	-	6	15468478
OsJapC10-UG119-2	840	100.0%	-	1	18158784
OsJapC10-UG12-1	1141	99.8%	+	1	14717490
OsJapC10-UG755-X2	673	99.7%	+	1	8024565
OsJapC10-UG414-3	1170	99.7%	-	3	17348488
OsJapC10-UG1232-2	629	99.8%	-	2	2392829
OsJapC10-UG21-2	786	91.0%	+	5	6372339
OsJapC10-UG67-2	584	100.0%	+	1	4099485
OsJapC10-UG154-8	1254	99.8%	-	4	21297326
OsJapC10-UG278-8	626	94.7%	-	4	9738945
OsJapC10-UG119-3	840	100.0%	+	1	18158784
OsJapC10-UG104-5	743	99.9%	-	1	14958130
OsJapC10-UG21-1	786	91.0%	-	5	6372339
OsJapC10-UG1205-5	940	100.0%	+	1	22131960
OsJapC10-UG1216-3	830	99.9%	-	1	22637991
OsJapC10-UG1080-1	867	99.9%	-	1	17775780
OsJapC10-UG872-2	967	100.0%	+	3	11297196

OsJapC10-UG42-3	246	100.0%	+	1	20659903
OsJapC10-UG865-5	672	99.9%	-	2	11227496
OsJapC10-UG251-8	836	99.5%	-	2	17645280
OsJapC10-UG298-5	1246	99.6%	+	2	960766
OsJapC10-UG947-7	872	81.8%	-	7	13829897
OsJapC10-UG105-1	503	99.8%	-	1	14765804
OsJapC10-UG867-2	677	90.7%	-	6	11238546
OsJapC10-UG970-5	339	100.0%	+	1	14272155
OsJapC10-UG1147-6	354	99.7%	+	1	19987071
OsJapC10-UG943-C4	320	100.0%	+	1	13734594
OsJapC10-UG111-2	461	100.0%	-	1	18165548
OsJapC10-UG311-8	579	99.7%	-	4	361544
OsJapC10-UG111-6	461	100.0%	-	1	18165548
OsJapC10-UG196-B6	1156	99.9%	-	1	14758709
OsJapC10-UG251-7	813	99.8%	-	2	17645279
OsJapC10-UG298-4	1246	99.6%	+	2	960766
OsJapC10-UG982-6	315	100.0%	-	1	14685997
OsJapC10-UG311-7	579	99.8%	-	4	361544
OsJapC10-UG369-2	940	99.9%	+	1	12375317
OsJapC10-UG105-3	503	99.2%	-	1	14765804
OsJapC10-UG123-2	320	100.0%	-	1	18798635
OsJapC10-UG212-1	838	98.0%	+	9	17668162
OsJapC10-SG13-1	662	99.8%	-	3	9117057
OsJapC10-UG978-5	311	99.7%	-	2	14363680
OsJapC10-UG1382-C7	273	100.0%	+	2	6377903
OsJapC10-SG190-2	994	99.7%	+	10	21577263
OsJapC10-SG59-1	1135	99.7%	+	2	13668621
OsJapC10-UG631-7	774	99.6%	-	3	20924019
OsJapC10-UG1382-A8	595	58.3%	+	2	6377904
OsJapC10-SG29-7	1078	100.0%	+	5	1077385
OsJapC10-SG102-1	205	100.0%	+	1	16931945
OsJapC10-UG703-8	611	99.7%	-	1	5523464
OsJapC10-UG511-7	1108	99.7%	-	1	12289975
OsJapC10-UG1081-3	657	99.5%	-	2	17780638
OsJapC10-SG25-4	688	99.7%	-	5	10801285
OsJapC10-SG211-2	534	100.0%	+	2	22178637
OsJapC10-UG926-1	497	94.0%	+	5	13089278
OsJapC10-SG207-5	410	99.8%	-	6	21950184
OsJapC10-UG217-B1	780	99.9%	+	4	7222521

OsJapC10-UG1459-2	510	99.4%	-	1	10062365
OsJapC10-UG1225-3	983	100.0%	-	2	2273318
OsJapC10-SG17-1	302	100.0%	-	2	9853832
OsJapC10-UG934-1	644	99.8%	-	2	13309165
OsJapC10-SG219-1	334	100.0%	+	2	22079319
OsJapC10-SG26-1	703	99.7%	-	2	10886579
OsJapC10-UG1454-A1	667	31.3%	+	5	9903495
OsJapC10-SG97-1	467	100.0%	+	4	16726773
OsJapC10-UG1436-A3	307	100.0%	+	1	9163606
OsJapC10-SG185-1	1196	99.8%	-	2	2082433
OsJapC10-SG154-1	2376	68.6%	-	8	19891949
OsJapC10-UG127-3	793	100.0%	+	2	20050744
OsJapC10-SG177-1	422	100.0%	+	1	21167806
OsJapC10-UG640-6	994	89.0%	+	3	2142155
OsJapC10-UG64-1	1376	99.9%	+	1	575475
OsJapC10-UG1427-A3	474	100.0%	+	1	8371354
OsJapC10-SG157-2	819	99.8%	+	2	20097956
OsJapC10-SG206-3	1294	99.6%	-	2	21923344
OsJapC10-SG124-1	190	100.0%	+	3	18167259
OsJapC10-UG599-2	821	100.0%	+	4	18044100
OsJapC10-UG1259-1	414	100.0%	-	1	305259
OsJapC10-SG87-1	587	99.8%	-	1	16081013
OsJapC10-SG89-3	584	99.8%	-	1	16125227
OsJapC10-UG713-1	554	98.7%	+	5	6065936
OsJapC10-SG7-2	1246	99.8%	+	3	7653687
OsJapC10-UG644-8	1245	99.7%	+	10	212886
OsJapC10-UG135-3	527	100.0%	+	2	20835364
OsJapC10-UG897-3	1256	99.9%	+	1	11969615
OsJapC10-SG23-7	269	99.6%	-	2	10688186
OsJapC10-SG37-1	825	100.0%	-	1	12185505
OsJapC10-UG688-1	445	100.0%	+	1	5025963
OsJapC10-UG526-1	373	99.7%	-	2	13219559
OsJapC10-UG582-8	702	99.9%	-	2	16690421
OsJapC10-UG1382-B4	349	100.0%	-	1	6377904
OsJapC10-SG196-1	1933	62.0%	-	23	21734304
OsJapC10-SG92-1	770	99.7%	-	6	16480947
OsJapC10-UG663-7	585	80.3%	-	8	3700562
OsJapC10-SG231-1	784	99.7%	-	2	3004837
OsJapC10-SG81-1	608	100.0%	-	1	15990636

OsJapC10-SG82-3	354	94.6%	-	2	15994029
OsJapC10-SG142-1	384	100.0%	-	3	18850254
OsJapC10-UG705-6	554	99.6%	-	5	5602896
OsJapC10-UG143-1	728	100.0%	+	2	20104531
OsJapC10-UG693-1	217	100.0%	+	1	5226284
OsJapC10-UG539-4	1242	100.0%	+	2	13737360
OsJapC10-UG650-1	703	99.6%	+	1	2508067
OsJapC10-SG189-1	1093	99.8%	+	4	21488721
OsJapC10-SG46-A2	403	100.0%	-	1	12717352
OsJapC10-SG83-1	1597	99.8%	+	3	1517068
OsJapC10-UG684-6	438	100.0%	-	1	4712048
OsJapC10-UG632-2	424	99.8%	-	1	20928499
OsJapC10-UG545-2	524	99.6%	-	1	143887
OsJapC10-SG14-3	637	100.0%	+	3	9150641
OsJapC10-SG212-1	136	93.4%	+	2	22227229
OsJapC10-SG70-1	1290	99.9%	-	1	1428179
OsJapC10-UG1282-1	388	98.7%	-	2	3849497
OsJapC10-SG115-1	332	99.1%	-	1	17818208
OsJapC10-UG517-8	474	91.4%	+	1	12693625
OsJapC10-SG22-1	566	100.0%	+	1	10681514
OsJapC10-UG1395-A3	350	99.7%	+	1	6675204
OsJapC10-UG583-4	736	99.7%	-	2	16690421
OsJapC10-UG1473-2	858	99.8%	+	2	10838478
OsJapC10-SG141-4	375	96.5%	-	2	18806767
OsJapC10-UG591-1	411	100.0%	+	2	17484660
OsJapC10-UG567-8	985	98.4%	+	1	15796844
OsJapC10-UG964-A2	317	100.0%	+	1	14162678
OsJapC10-SG209-1	290	100.0%	+	2	22075945
OsJapC10-UG879-A2	423	99.8%	+	2	11396128
OsJapC10-UG807-4	488	99.6%	-	6	9575237
OsJapC10-SG30-1	490	97.3%	-	5	11039308
OsJapC10-UG607-2	1273	99.6%	-	1	18691346
OsJapC10-UG640-4	994	89.4%	+	3	2142155
OsJapC10-SG204-1	750	99.7%	+	2	2131113
OsJapC10-UG1443-1	202	59.9%	-	3	9322057
OsJapC10-UG1342-1	389	99.7%	+	1	5126383
OsJapC10-SG244-2	1014	99.7%	+	5	5498296
OsJapC10-SG165-B4	1396	99.8%	+	3	20386831
OsJapC10-UG171-B2	571	99.3%	+	5	20224866

OsJapC10-SG165-A1	1069	99.6%	-	4	20386831
OsJapC10-UG209-1	932	99.8%	+	9	6603829
OsJapC10-SG200-1	361	100.0%	+	5	21803778
OsJapC10-SG98-1	718	100.0%	+	5	16757514
OsJapC10-UG538-B2	558	99.8%	-	2	13735494
OsJapC10-UG1309-6	565	100.0%	+	1	4389699

[1] Length of the clone that could be mapped to the target UG model. [2] Starting positi
