# Supplementary material for: Tiling microarray analysis of rice chromosome 10 to identify the transcriptome and relate its expression to chromosomal architecture
Source: Genome Biol. 2005 May 27;6(6):R52. doi: 10.1186/gb-2005-6-6-r52 (PMC1175972; doi:10.1186/gb-2005-6-6-r52)
Supplement: Additional File 4 — Table S4: Japonica chromosome 10 intergenic TARs. Japonica chromosome 10 intergenic TARs. [file gb-2005-6-6-r52-S4.pdf]

Supplemental Table 4. *Japonica* chromosome 10 intergenic TARs

Intergenic TAR	Position [1]	Length	Strand	Probe #	Signal Probe #	Log2 intensity
OSJapC10_N_TAR_592	44421	322	-	7	7	8.331
OSJapC10_N_TAR_166	47477	452	+	10	10	8.820
OSJapC10_N_TAR_593	47570	359	-	8	8	8.488
OSJapC10_N_TAR_167	73955	268	+	6	6	8.066
OSJapC10_N_TAR_168	141396	589	+	13	10	9.202
OSJapC10_N_TAR_169	231931	711	+	15	14	9.474
OSJapC10_N_TAR_594	241313	509	-	11	11	8.992
OSJapC10_N_TAR_170	259404	492	+	11	11	8.943
OSJapC10_N_TAR_171	275046	557	+	12	12	9.122
OSJapC10_N_TAR_595	275230	465	-	10	10	8.861
OSJapC10_N_TAR_173	308719	652	+	14	14	9.349
OSJapC10_N_TAR_596	308719	541	-	12	12	9.079
OSJapC10_N_TAR_174	424375	488	+	11	10	8.931
OSJapC10_N_TAR_175	447685	567	+	12	10	9.147
OSJapC10_N_TAR_176	482608	404	+	9	6	8.658
OSJapC10_N_TAR_178	533083	430	+	9	9	8.748
OSJapC10_N_TAR_599	656362	236	-	5	5	7.883
OSJapC10_N_TAR_182	763925	681	+	15	15	9.412
OSJapC10_N_TAR_601	839980	354	-	8	8	8.468
OSJapC10_N_TAR_184	840072	506	+	11	10	8.983
OSJapC10_N_TAR_188	904312	1108	+	24	24	10.114
OSJapC10_N_TAR_603	905241	547	-	12	12	9.095
OSJapC10_N_TAR_190	1014316	497	+	11	11	8.957
OSJapC10_N_TAR_191	1056089	827	+	18	16	9.692
OSJapC10_N_TAR_605	1056591	325	-	7	7	8.344
OSJapC10_N_TAR_192	1058691	271	+	6	6	8.082
OSJapC10_N_TAR_193	1087003	877	+	19	17	9.776
OSJapC10_N_TAR_606	1087003	350	-	8	6	8.451
OSJapC10_N_TAR_194	1103277	652	+	14	13	9.349
OSJapC10_N_TAR_195	1154120	875	+	19	15	9.773
OSJapC10_N_TAR_607	1154120	791	-	17	14	9.628
OSJapC10_N_TAR_608	1157065	267	-	6	6	8.061
OSJapC10_N_TAR_196	1186715	621	+	14	13	9.278
OSJapC10_N_TAR_609	1186849	809	-	18	17	9.660
OSJapC10_N_TAR_610	1214133	390	-	8	8	8.607
OSJapC10_N_TAR_197	1214363	436	+	9	9	8.768
OSJapC10_N_TAR_198	1252155	764	+	17	15	9.577
OSJapC10_N_TAR_611	1252155	393	-	9	8	8.618
OSJapC10_N_TAR_612	1282376	543	-	12	12	9.085
OSJapC10_N_TAR_201	1402108	505	+	11	11	8.980
OSJapC10_N_TAR_614	1402154	413	-	9	9	8.690
OSJapC10_N_TAR_202	1409473	464	+	10	5	8.858
OSJapC10_N_TAR_203	1418461	290	+	6	6	8.180
OSJapC10_N_TAR_615	1479268	334	-	7	6	8.384
OSJapC10_N_TAR_204	1493430	859	+	19	17	9.747
OSJapC10_N_TAR_616	1493971	318	-	7	7	8.313
OSJapC10_N_TAR_617	1503024	651	-	14	13	9.347
OSJapC10_N_TAR_205	1621541	405	+	9	9	8.662
OSJapC10_N_TAR_206	1636369	2738	+	60	42	11.419
OSJapC10_N_TAR_618	1637664	1351	-	29	25	10.400
OSJapC10_N_TAR_208	1780017	643	+	14	11	9.329
OSJapC10_N_TAR_619	1780017	308	-	7	6	8.267
OSJapC10_N_TAR_620	1807315	221	-	5	5	7.788
OSJapC10_N_TAR_621	1920913	221	-	5	5	7.788
OSJapC10_N_TAR_213	2089698	499	+	11	11	8.963
OSJapC10_N_TAR_214	2120093	1663	+	36	30	10.700
OSJapC10_N_TAR_623	2123597	419	-	9	9	8.711
OSJapC10_N_TAR_215	2150808	858	+	19	11	9.745

OSJapC10_N_TAR_217	2278999	316	+	7	7	8.304
OSJapC10_N_TAR_218	2288524	595	+	13	6	9.217
OSJapC10_N_TAR_219	2309160	3374	+	73	45	11.720
OSJapC10_N_TAR_625	2309429	1973	-	43	28	10.946
OSJapC10_N_TAR_626	2327995	310	-	7	6	8.276
OSJapC10_N_TAR_222	2340831	635	+	14	14	9.311
OSJapC10_N_TAR_223	2363504	483	+	11	7	8.916
OSJapC10_N_TAR_224	2374779	318	+	7	7	8.313
OSJapC10_N_TAR_226	2426943	561	+	12	12	9.132
OSJapC10_N_TAR_227	2459166	351	+	8	5	8.455
OSJapC10_N_TAR_632	2566461	221	-	5	5	7.788
OSJapC10_N_TAR_231	2753856	911	+	20	20	9.831
OSJapC10_N_TAR_634	2753856	405	-	9	9	8.662
OSJapC10_N_TAR_233	2775959	406	+	9	9	8.665
OSJapC10_N_TAR_234	2783833	270	+	6	6	8.077
OSJapC10_N_TAR_636	2845139	552	-	12	11	9.109
OSJapC10_N_TAR_236	2849506	831	+	18	16	9.699
OSJapC10_N_TAR_637	2849552	228	-	5	5	7.833
OSJapC10_N_TAR_237	2851741	722	+	16	16	9.496
OSJapC10_N_TAR_638	2852109	308	-	7	7	8.267
OSJapC10_N_TAR_639	2902915	713	-	16	11	9.478
OSJapC10_N_TAR_239	2930349	459	+	10	10	8.842
OSJapC10_N_TAR_240	2938026	1360	+	30	24	10.409
OSJapC10_N_TAR_640	3008042	361	-	8	8	8.496
OSJapC10_N_TAR_241	3008134	361	+	8	8	8.496
OSJapC10_N_TAR_242	3020632	497	+	11	11	8.957
OSJapC10_N_TAR_243	3023339	417	+	9	5	8.704
OSJapC10_N_TAR_244	3072092	267	+	6	6	8.061
OSJapC10_N_TAR_245	3084516	761	+	17	10	9.572
OSJapC10_N_TAR_642	3084516	427	-	9	8	8.738
OSJapC10_N_TAR_643	3112808	1019	-	22	22	9.993
OSJapC10_N_TAR_246	3113042	1948	+	42	38	10.928
OSJapC10_N_TAR_247	3129738	609	+	13	6	9.250
OSJapC10_N_TAR_649	3293407	911	-	20	15	9.831
OSJapC10_N_TAR_251	3293597	721	+	16	14	9.494
OSJapC10_N_TAR_252	3364784	544	+	12	10	9.087
OSJapC10_N_TAR_253	3367659	543	+	12	12	9.085
OSJapC10_N_TAR_254	3425234	646	+	14	13	9.335
OSJapC10_N_TAR_650	3425590	290	-	6	6	8.180
OSJapC10_N_TAR_256	3473703	1522	+	33	21	10.572
OSJapC10_N_TAR_257	3477564	451	+	10	10	8.817
OSJapC10_N_TAR_652	3477610	2075	-	45	43	11.019
OSJapC10_N_TAR_653	3529355	603	-	13	12	9.236
OSJapC10_N_TAR_258	3529495	463	+	10	9	8.855
OSJapC10_N_TAR_654	3588775	1408	-	31	19	10.459
OSJapC10_N_TAR_259	3588913	1316	+	29	18	10.362
OSJapC10_N_TAR_655	3593681	658	-	14	12	9.362
OSJapC10_N_TAR_656	3615644	2166	-	47	46	11.081
OSJapC10_N_TAR_263	3675714	1051	+	23	23	10.038
OSJapC10_N_TAR_264	3689116	267	+	6	6	8.061
OSJapC10_N_TAR_659	3725183	523	-	11	7	9.031
OSJapC10_N_TAR_265	3791274	708	+	15	12	9.468
OSJapC10_N_TAR_660	3791507	475	-	10	8	8.892
OSJapC10_N_TAR_266	3809872	1043	+	23	20	10.027
OSJapC10_N_TAR_661	3809872	781	-	17	15	9.609
OSJapC10_N_TAR_268	3883854	538	+	12	12	9.071
OSJapC10_N_TAR_663	3883854	538	-	12	12	9.071
OSJapC10_N_TAR_664	3912437	1592	-	35	28	10.637
OSJapC10_N_TAR_269	3912529	1152	+	25	19	10.170
OSJapC10_N_TAR_270	3922405	703	+	15	14	9.457
OSJapC10_N_TAR_271	3945542	359	+	8	8	8.488
OSJapC10_N_TAR_272	3959341	677	+	15	15	9.403

OSJapC10_N_TAR_665	3959341	631	-	14	14	9.301
OSJapC10_N_TAR_273	3983834	891	+	19	17	9.799
OSJapC10_N_TAR_666	3983834	845	-	18	16	9.723
OSJapC10_N_TAR_274	4004916	823	+	18	18	9.685
OSJapC10_N_TAR_275	4019373	692	+	15	14	9.435
OSJapC10_N_TAR_667	4019373	494	-	11	10	8.948
OSJapC10_N_TAR_669	4146918	275	-	6	6	8.103
OSJapC10_N_TAR_277	4166144	1164	+	25	25	10.185
OSJapC10_N_TAR_670	4166185	469	-	10	10	8.873
OSJapC10_N_TAR_278	4174863	527	+	11	11	9.042
OSJapC10_N_TAR_279	4206859	249	+	5	5	7.960
OSJapC10_N_TAR_671	4206859	653	-	14	12	9.351
OSJapC10_N_TAR_280	4254800	279	+	6	6	8.124
OSJapC10_N_TAR_672	4265992	736	-	16	11	9.524
OSJapC10_N_TAR_281	4266183	697	+	15	9	9.445
OSJapC10_N_TAR_675	4361148	405	-	9	9	8.662
OSJapC10_N_TAR_283	4362066	1463	+	32	32	10.515
OSJapC10_N_TAR_286	4522700	221	+	5	5	7.788
OSJapC10_N_TAR_677	4646438	320	-	7	7	8.322
OSJapC10_N_TAR_678	4664345	452	-	10	8	8.820
OSJapC10_N_TAR_288	4811241	458	+	10	9	8.839
OSJapC10_N_TAR_681	4862975	316	-	7	7	8.304
OSJapC10_N_TAR_289	4907821	977	+	21	21	9.932
OSJapC10_N_TAR_682	4907821	1741	-	38	34	10.766
OSJapC10_N_TAR_291	4929693	599	+	13	12	9.226
OSJapC10_N_TAR_683	4929693	342	-	7	7	8.418
OSJapC10_N_TAR_684	4938430	359	-	8	8	8.488
OSJapC10_N_TAR_292	4938568	403	+	9	9	8.655
OSJapC10_N_TAR_294	5049067	267	+	6	6	8.061
OSJapC10_N_TAR_295	5078311	327	+	7	7	8.353
OSJapC10_N_TAR_686	5081310	787	-	17	8	9.620
OSJapC10_N_TAR_687	5096713	453	-	10	9	8.823
OSJapC10_N_TAR_297	5097025	232	+	5	5	7.858
OSJapC10_N_TAR_298	5114799	589	+	13	13	9.202
OSJapC10_N_TAR_299	5131041	2383	+	52	51	11.219
OSJapC10_N_TAR_301	5149508	773	+	17	17	9.594
OSJapC10_N_TAR_302	5161815	654	+	14	14	9.353
OSJapC10_N_TAR_691	5183277	257	-	6	5	8.006
OSJapC10_N_TAR_305	5246087	461	+	10	10	8.849
OSJapC10_N_TAR_692	5246087	369	-	8	8	8.527
OSJapC10_N_TAR_306	5329890	914	+	20	20	9.836
OSJapC10_N_TAR_307	5347620	428	+	9	9	8.741
OSJapC10_N_TAR_694	5411829	377	-	8	8	8.558
OSJapC10_N_TAR_308	5434528	664	+	14	14	9.375
OSJapC10_N_TAR_309	5462714	497	+	11	11	8.957
OSJapC10_N_TAR_311	5569855	313	+	7	7	8.290
OSJapC10_N_TAR_312	5581800	600	+	13	13	9.229
OSJapC10_N_TAR_313	5586574	477	+	10	10	8.898
OSJapC10_N_TAR_314	5633866	497	+	11	11	8.957
OSJapC10_N_TAR_315	5729856	400	+	9	7	8.644
OSJapC10_N_TAR_316	5815942	451	+	10	10	8.817
OSJapC10_N_TAR_317	5926272	1395	+	30	13	10.446
OSJapC10_N_TAR_318	6012749	320	+	7	7	8.322
OSJapC10_N_TAR_320	6217351	541	+	12	5	9.079
OSJapC10_N_TAR_699	6217351	541	-	12	5	9.079
OSJapC10_N_TAR_700	6231053	454	-	10	10	8.827
OSJapC10_N_TAR_701	6304218	1310	-	28	28	10.355
OSJapC10_N_TAR_321	6305169	497	+	11	11	8.957
OSJapC10_N_TAR_322	6348533	267	+	6	6	8.061
OSJapC10_N_TAR_323	6368282	782	+	17	15	9.611
OSJapC10_N_TAR_324	6401488	819	+	18	18	9.678
OSJapC10_N_TAR_702	6401488	497	-	11	11	8.957

OSJapC10_N_TAR_703	6432604	329	-	7	6	8.362
OSJapC10_N_TAR_325	6432650	645	+	14	11	9.333
OSJapC10_N_TAR_704	6537049	353	-	8	7	8.464
OSJapC10_N_TAR_327	6537187	412	+	9	6	8.687
OSJapC10_N_TAR_328	6556375	496	+	11	10	8.954
OSJapC10_N_TAR_331	6847843	267	+	6	6	8.061
OSJapC10_N_TAR_332	6880896	2141	+	47	35	11.064
OSJapC10_N_TAR_333	6896794	937	+	20	20	9.872
OSJapC10_N_TAR_708	6896794	502	-	11	11	8.972
OSJapC10_N_TAR_334	6943817	819	+	18	18	9.678
OSJapC10_N_TAR_336	7178460	639	+	14	14	9.320
OSJapC10_N_TAR_337	7251435	347	+	8	7	8.439
OSJapC10_N_TAR_342	7424149	541	+	12	11	9.079
OSJapC10_N_TAR_715	7424149	449	-	10	10	8.811
OSJapC10_N_TAR_716	7428138	267	-	6	6	8.061
OSJapC10_N_TAR_344	7524408	959	+	21	16	9.905
OSJapC10_N_TAR_717	7524408	334	-	7	6	8.384
OSJapC10_N_TAR_348	7538875	929	+	20	16	9.860
OSJapC10_N_TAR_349	7543265	333	+	7	7	8.379
OSJapC10_N_TAR_719	7543265	333	-	7	7	8.379
OSJapC10_N_TAR_721	7636794	461	-	10	7	8.849
OSJapC10_N_TAR_352	7961773	463	+	10	8	8.855
OSJapC10_N_TAR_727	7979783	2767	-	60	26	11.434
OSJapC10_N_TAR_355	7982169	550	+	12	11	9.103
OSJapC10_N_TAR_728	8000909	359	-	8	8	8.488
OSJapC10_N_TAR_729	8055750	219	-	5	5	7.775
OSJapC10_N_TAR_358	8120881	1968	+	43	42	10.943
OSJapC10_N_TAR_730	8124717	573	-	12	11	9.162
OSJapC10_N_TAR_359	8124855	259	+	6	5	8.017
OSJapC10_N_TAR_731	8127016	493	-	11	11	8.945
OSJapC10_N_TAR_363	8194175	470	+	10	10	8.877
OSJapC10_N_TAR_734	8194378	267	-	6	6	8.061
OSJapC10_N_TAR_364	8222849	798	+	17	16	9.640
OSJapC10_N_TAR_366	8347034	681	+	15	15	9.412
OSJapC10_N_TAR_736	8347126	497	-	11	11	8.957
OSJapC10_N_TAR_367	8378844	1058	+	23	23	10.047
OSJapC10_N_TAR_368	8403545	561	+	12	12	9.132
OSJapC10_N_TAR_370	8506478	1409	+	31	13	10.460
OSJapC10_N_TAR_740	8509515	682	-	15	12	9.414
OSJapC10_N_TAR_371	8509561	682	+	15	12	9.414
OSJapC10_N_TAR_372	8604289	851	+	19	18	9.733
OSJapC10_N_TAR_373	8608531	530	+	12	11	9.050
OSJapC10_N_TAR_741	8608531	392	-	9	8	8.615
OSJapC10_N_TAR_375	8683775	289	+	6	5	8.175
OSJapC10_N_TAR_376	8709752	403	+	9	9	8.655
OSJapC10_N_TAR_377	8824818	603	+	13	13	9.236
OSJapC10_N_TAR_743	8892355	758	-	16	16	9.566
OSJapC10_N_TAR_379	9071463	771	+	17	16	9.591
OSJapC10_N_TAR_745	9071614	712	-	15	15	9.476
OSJapC10_N_TAR_746	9086682	643	-	14	14	9.329
OSJapC10_N_TAR_380	9086828	267	+	6	6	8.061
OSJapC10_N_TAR_381	9090473	1697	+	37	36	10.729
OSJapC10_N_TAR_747	9091762	408	-	9	9	8.672
OSJapC10_N_TAR_748	9113954	468	-	10	5	8.870
OSJapC10_N_TAR_749	9131319	481	-	10	9	8.910
OSJapC10_N_TAR_382	9133198	1233	+	27	27	10.268
OSJapC10_N_TAR_750	9138385	536	-	12	11	9.066
OSJapC10_N_TAR_383	9296243	852	+	19	17	9.735
OSJapC10_N_TAR_752	9296243	806	-	18	16	9.655
OSJapC10_N_TAR_384	9302526	470	+	10	10	8.877
OSJapC10_N_TAR_387	9446560	313	+	7	7	8.290
OSJapC10_N_TAR_388	9452092	656	+	14	14	9.358

OSJapC10_N_TAR_755	9452092	468	-	10	10	8.870
OSJapC10_N_TAR_389	9467503	541	+	12	7	9.079
OSJapC10_N_TAR_756	9480011	1841	-	40	29	10.846
OSJapC10_N_TAR_390	9481120	2371	+	52	30	11.211
OSJapC10_N_TAR_391	9504023	221	+	5	5	7.788
OSJapC10_N_TAR_393	9717255	229	+	5	5	7.839
OSJapC10_N_TAR_757	9764285	323	-	7	6	8.335
OSJapC10_N_TAR_394	9897497	321	+	7	7	8.326
OSJapC10_N_TAR_760	9901613	454	-	10	10	8.827
OSJapC10_N_TAR_395	9919459	681	+	15	15	9.412
OSJapC10_N_TAR_761	9919643	497	-	11	11	8.957
OSJapC10_N_TAR_762	9930209	267	-	6	6	8.061
OSJapC10_N_TAR_396	9958542	3712	+	81	55	11.858
OSJapC10_N_TAR_397	9967354	730	+	16	15	9.512
OSJapC10_N_TAR_765	9967354	404	-	9	9	8.658
OSJapC10_N_TAR_398	9973992	267	+	6	6	8.061
OSJapC10_N_TAR_399	9986054	490	+	11	9	8.937
OSJapC10_N_TAR_400	9994245	695	+	15	15	9.441
OSJapC10_N_TAR_768	9994614	717	-	16	15	9.486
OSJapC10_N_TAR_401	10001124	253	+	6	5	7.983
OSJapC10_N_TAR_402	10080640	235	+	5	5	7.877
OSJapC10_N_TAR_403	10145938	543	+	12	12	9.085
OSJapC10_N_TAR_405	10253460	591	+	13	13	9.207
OSJapC10_N_TAR_769	10262101	454	-	10	10	8.827
OSJapC10_N_TAR_406	10271493	468	+	10	10	8.870
OSJapC10_N_TAR_410	10307103	441	+	10	9	8.785
OSJapC10_N_TAR_773	10320167	522	-	11	10	9.028
OSJapC10_N_TAR_411	10349546	275	+	6	5	8.103
OSJapC10_N_TAR_416	10473727	294	+	6	6	8.200
OSJapC10_N_TAR_417	10567290	831	+	18	16	9.699
OSJapC10_N_TAR_777	10603363	616	-	13	13	9.267
OSJapC10_N_TAR_418	10677107	267	+	6	6	8.061
OSJapC10_N_TAR_419	10735889	367	+	8	8	8.520
OSJapC10_N_TAR_781	11074217	1175	-	26	25	10.198
OSJapC10_N_TAR_422	11074278	1572	+	34	34	10.618
OSJapC10_N_TAR_423	11108858	267	+	6	6	8.061
OSJapC10_N_TAR_424	11116070	797	+	17	14	9.638
OSJapC10_N_TAR_782	11171586	1136	-	25	25	10.150
OSJapC10_N_TAR_425	11328849	816	+	18	17	9.672
OSJapC10_N_TAR_1	11369632	551	+	12	12	9.106
OSJapC10_N_TAR_2	11386995	497	+	11	11	8.957
OSJapC10_N_TAR_3	11407856	372	+	8	8	8.539
OSJapC10_N_TAR_426	11408007	221	-	5	5	7.788
OSJapC10_N_TAR_4	11418507	496	+	11	7	8.954
OSJapC10_N_TAR_427	11419909	783	-	17	12	9.613
OSJapC10_N_TAR_5	11420655	323	+	7	5	8.335
OSJapC10_N_TAR_428	11422675	565	-	12	12	9.142
OSJapC10_N_TAR_6	11571835	803	+	17	17	9.649
OSJapC10_N_TAR_7	11698228	451	+	10	10	8.817
OSJapC10_N_TAR_8	11704403	994	+	22	21	9.957
OSJapC10_N_TAR_429	11704403	948	-	21	20	9.889
OSJapC10_N_TAR_9	11714261	408	+	9	9	8.672
OSJapC10_N_TAR_10	11717508	282	+	6	6	8.140
OSJapC10_N_TAR_430	11729222	1189	-	26	20	10.216
OSJapC10_N_TAR_431	11775726	389	-	8	7	8.604
OSJapC10_N_TAR_432	11795998	3638	-	79	73	11.829
OSJapC10_N_TAR_12	11796338	3344	+	73	67	11.707
OSJapC10_N_TAR_435	12145415	575	-	13	12	9.167
OSJapC10_N_TAR_18	12259410	1824	+	40	39	10.833
OSJapC10_N_TAR_438	12300254	539	-	12	12	9.074
OSJapC10_N_TAR_19	12300530	452	+	10	10	8.820
OSJapC10_N_TAR_439	12302494	405	-	9	9	8.662

OSJapC10_N_TAR_440	12307645	790	-	17	17	9.626
OSJapC10_N_TAR_20	12307978	503	+	11	11	8.974
OSJapC10_N_TAR_441	12372549	417	-	9	9	8.704
OSJapC10_N_TAR_21	12378526	2276	+	49	47	11.152
OSJapC10_N_TAR_442	12446000	3172	-	69	45	11.631
OSJapC10_N_TAR_443	12453340	415	-	9	9	8.697
OSJapC10_N_TAR_24	12521292	461	+	10	7	8.849
OSJapC10_N_TAR_444	12523150	449	-	10	9	8.811
OSJapC10_N_TAR_25	12574777	508	+	11	11	8.989
OSJapC10_N_TAR_26	12617247	1588	+	35	34	10.633
OSJapC10_N_TAR_445	12618514	321	-	7	7	8.326
OSJapC10_N_TAR_27	12664928	1795	+	39	36	10.810
OSJapC10_N_TAR_446	12829010	275	-	6	6	8.103
OSJapC10_N_TAR_28	12829064	267	+	6	6	8.061
OSJapC10_N_TAR_447	12861946	513	-	11	11	9.003
OSJapC10_N_TAR_29	12902246	2687	+	58	49	11.392
OSJapC10_N_TAR_448	12904275	502	-	11	9	8.972
OSJapC10_N_TAR_449	12922736	367	-	8	7	8.520
OSJapC10_N_TAR_30	12922842	532	+	12	7	9.055
OSJapC10_N_TAR_31	13030313	454	+	10	10	8.827
OSJapC10_N_TAR_450	13030313	359	-	8	8	8.488
OSJapC10_N_TAR_453	13125343	3053	-	66	62	11.576
OSJapC10_N_TAR_33	13127116	1372	+	30	28	10.422
OSJapC10_N_TAR_454	13135073	456	-	10	10	8.833
OSJapC10_N_TAR_34	13152780	1611	+	35	19	10.654
OSJapC10_N_TAR_35	13195525	359	+	8	8	8.488
OSJapC10_N_TAR_455	13295776	497	-	11	11	8.957
OSJapC10_N_TAR_37	13390157	569	+	12	8	9.152
OSJapC10_N_TAR_456	13390157	464	-	10	6	8.858
OSJapC10_N_TAR_457	13399864	640	-	14	14	9.322
OSJapC10_N_TAR_38	13399910	686	+	15	15	9.422
OSJapC10_N_TAR_458	13428310	452	-	10	10	8.820
OSJapC10_N_TAR_40	13471045	335	+	7	7	8.388
OSJapC10_N_TAR_459	13516044	405	-	9	9	8.662
OSJapC10_N_TAR_41	13525029	512	+	11	11	9.000
OSJapC10_N_TAR_460	13526558	568	-	12	12	9.150
OSJapC10_N_TAR_461	13541524	337	-	7	7	8.397
OSJapC10_N_TAR_42	13541570	429	+	9	9	8.745
OSJapC10_N_TAR_43	13546870	322	+	7	6	8.331
OSJapC10_N_TAR_462	13553896	1340	-	29	29	10.388
OSJapC10_N_TAR_44	13554960	322	+	7	7	8.331
OSJapC10_N_TAR_45	13605985	1805	+	39	39	10.818
OSJapC10_N_TAR_464	13674717	453	-	10	9	8.823
OSJapC10_N_TAR_46	13712072	879	+	19	14	9.780
OSJapC10_N_TAR_466	13714346	727	-	16	16	9.506
OSJapC10_N_TAR_47	13714716	357	+	8	8	8.480
OSJapC10_N_TAR_467	13995850	460	-	10	10	8.845
OSJapC10_N_TAR_469	14066566	451	-	10	10	8.817
OSJapC10_N_TAR_470	14084580	457	-	10	10	8.836
OSJapC10_N_TAR_49	14084672	457	+	10	10	8.836
OSJapC10_N_TAR_51	14118775	405	+	9	9	8.662
OSJapC10_N_TAR_52	14123618	900	+	20	19	9.814
OSJapC10_N_TAR_471	14123903	1603	-	35	30	10.647
OSJapC10_N_TAR_53	14148877	405	+	9	9	8.662
OSJapC10_N_TAR_54	14153924	267	+	6	6	8.061
OSJapC10_N_TAR_472	14165443	456	-	10	5	8.833
OSJapC10_N_TAR_57	14174076	635	+	14	14	9.311
OSJapC10_N_TAR_58	14190055	1235	+	27	27	10.270
OSJapC10_N_TAR_473	14190709	3515	-	76	73	11.779
OSJapC10_N_TAR_59	14192734	787	+	17	17	9.620
OSJapC10_N_TAR_60	14291464	267	+	6	6	8.061
OSJapC10_N_TAR_63	14345158	405	+	9	9	8.662

OSJapC10_N_TAR_474	14451782	439	-	10	9	8.778
OSJapC10_N_TAR_65	14487471	230	+	5	5	7.845
OSJapC10_N_TAR_475	14487572	507	-	11	11	8.986
OSJapC10_N_TAR_476	14530312	486	-	11	9	8.925
OSJapC10_N_TAR_478	14658364	311	-	7	6	8.281
OSJapC10_N_TAR_66	14786729	457	+	10	10	8.836
OSJapC10_N_TAR_480	14886961	454	-	10	10	8.827
OSJapC10_N_TAR_68	14976668	941	+	20	11	9.878
OSJapC10_N_TAR_482	15011434	300	-	7	5	8.229
OSJapC10_N_TAR_69	15034629	2346	+	51	45	11.196
OSJapC10_N_TAR_483	15035918	965	-	21	20	9.914
OSJapC10_N_TAR_485	15095579	1047	-	23	16	10.032
OSJapC10_N_TAR_70	15096308	318	+	7	6	8.313
OSJapC10_N_TAR_486	15117297	469	-	10	9	8.873
OSJapC10_N_TAR_71	15117562	250	+	5	5	7.966
OSJapC10_N_TAR_487	15170604	425	-	9	8	8.731
OSJapC10_N_TAR_488	15185637	657	-	14	12	9.360
OSJapC10_N_TAR_73	15209020	566	+	12	11	9.145
OSJapC10_N_TAR_489	15209071	327	-	7	7	8.353
OSJapC10_N_TAR_490	15262090	451	-	10	8	8.817
OSJapC10_N_TAR_492	15332816	2048	-	45	34	11.000
OSJapC10_N_TAR_74	15339763	453	+	10	10	8.823
OSJapC10_N_TAR_493	15342603	497	-	11	11	8.957
OSJapC10_N_TAR_75	15343799	1682	+	37	29	10.716
OSJapC10_N_TAR_494	15347926	490	-	11	6	8.937
OSJapC10_N_TAR_76	15348229	233	+	5	5	7.864
OSJapC10_N_TAR_495	15350401	1648	-	36	27	10.687
OSJapC10_N_TAR_77	15351315	826	+	18	18	9.690
OSJapC10_N_TAR_496	15407362	497	-	11	11	8.957
OSJapC10_N_TAR_78	15472479	405	+	9	9	8.662
OSJapC10_N_TAR_498	15565641	458	-	10	10	8.839
OSJapC10_N_TAR_499	15572309	359	-	8	8	8.488
OSJapC10_N_TAR_500	15578068	1036	-	23	22	10.017
OSJapC10_N_TAR_79	15578160	2524	+	55	49	11.301
OSJapC10_N_TAR_80	15601644	770	+	17	14	9.589
OSJapC10_N_TAR_501	15676447	445	-	10	9	8.798
OSJapC10_N_TAR_81	15696607	634	+	14	14	9.308
OSJapC10_N_TAR_502	15710529	2808	-	61	49	11.455
OSJapC10_N_TAR_82	15711560	1777	+	39	37	10.795
OSJapC10_N_TAR_85	15778088	924	+	20	20	9.852
OSJapC10_N_TAR_86	15783662	359	+	8	8	8.488
OSJapC10_N_TAR_87	15804936	322	+	7	7	8.331
OSJapC10_N_TAR_505	15804936	276	-	6	6	8.109
OSJapC10_N_TAR_88	15808425	357	+	8	8	8.480
OSJapC10_N_TAR_507	15810459	313	-	7	7	8.290
OSJapC10_N_TAR_508	15830686	497	-	11	11	8.957
OSJapC10_N_TAR_89	15831744	406	+	9	8	8.665
OSJapC10_N_TAR_509	15852137	313	-	7	7	8.290
OSJapC10_N_TAR_510	15902835	377	-	8	8	8.558
OSJapC10_N_TAR_90	15902927	653	+	14	14	9.351
OSJapC10_N_TAR_513	15970343	386	-	8	8	8.592
OSJapC10_N_TAR_514	15972787	462	-	10	5	8.852
OSJapC10_N_TAR_515	16024042	727	-	16	16	9.506
OSJapC10_N_TAR_92	16033396	875	+	19	18	9.773
OSJapC10_N_TAR_93	16083490	543	+	12	12	9.085
OSJapC10_N_TAR_517	16095396	783	-	17	14	9.613
OSJapC10_N_TAR_520	16369169	636	-	14	13	9.313
OSJapC10_N_TAR_97	16588842	359	+	8	8	8.488
OSJapC10_N_TAR_523	16800381	563	-	12	11	9.137
OSJapC10_N_TAR_524	16803578	569	-	12	12	9.152
OSJapC10_N_TAR_99	16851357	3305	+	72	50	11.690
OSJapC10_N_TAR_100	16901934	486	+	11	7	8.925

OSJapC10_N_TAR_101	17011592	696	+	15	14	9.443
OSJapC10_N_TAR_526	17011592	558	-	12	11	9.124
OSJapC10_N_TAR_102	17054918	1187	+	26	26	10.213
OSJapC10_N_TAR_103	17212526	313	+	7	7	8.290
OSJapC10_N_TAR_527	17304779	465	-	10	9	8.861
OSJapC10_N_TAR_528	17310435	722	-	16	16	9.496
OSJapC10_N_TAR_104	17331645	357	+	8	8	8.480
OSJapC10_N_TAR_530	17352508	333	-	7	7	8.379
OSJapC10_N_TAR_531	17419494	481	-	10	10	8.910
OSJapC10_N_TAR_532	17493846	420	-	9	9	8.714
OSJapC10_N_TAR_107	17528300	497	+	11	11	8.957
OSJapC10_N_TAR_108	17547955	370	+	8	8	8.531
OSJapC10_N_TAR_533	17597474	2077	-	45	41	11.020
OSJapC10_N_TAR_534	17616921	415	-	9	8	8.697
OSJapC10_N_TAR_110	17616967	530	+	12	10	9.050
OSJapC10_N_TAR_535	17635871	323	-	7	7	8.335
OSJapC10_N_TAR_111	17660441	543	+	12	12	9.085
OSJapC10_N_TAR_112	17681137	366	+	8	8	8.516
OSJapC10_N_TAR_536	17895495	368	-	8	8	8.524
OSJapC10_N_TAR_537	18084793	315	-	7	7	8.299
OSJapC10_N_TAR_113	18084885	223	+	5	5	7.801
OSJapC10_N_TAR_538	18144557	519	-	11	10	9.020
OSJapC10_N_TAR_114	18145624	401	+	9	6	8.647
OSJapC10_N_TAR_539	18150716	659	-	14	13	9.364
OSJapC10_N_TAR_540	18229088	313	-	7	7	8.290
OSJapC10_N_TAR_115	18262663	506	+	11	6	8.983
OSJapC10_N_TAR_116	18462409	798	+	17	17	9.640
OSJapC10_N_TAR_541	18462409	1070	-	23	23	10.063
OSJapC10_N_TAR_117	18835703	532	+	12	10	9.055
OSJapC10_N_TAR_542	18975675	686	-	15	15	9.422
OSJapC10_N_TAR_543	19084400	793	-	17	13	9.631
OSJapC10_N_TAR_119	19084458	735	+	16	12	9.522
OSJapC10_N_TAR_544	19132220	422	-	9	9	8.721
OSJapC10_N_TAR_120	19160530	235	+	5	5	7.877
OSJapC10_N_TAR_545	19237124	259	-	6	6	8.017
OSJapC10_N_TAR_546	19387232	228	-	5	5	7.833
OSJapC10_N_TAR_123	19402607	359	+	8	8	8.488
OSJapC10_N_TAR_124	19475949	497	+	11	11	8.957
OSJapC10_N_TAR_547	19480375	267	-	6	6	8.061
OSJapC10_N_TAR_548	19554201	313	-	7	7	8.290
OSJapC10_N_TAR_125	19614882	1135	+	25	12	10.148
OSJapC10_N_TAR_549	19615375	642	-	14	8	9.326
OSJapC10_N_TAR_126	19746838	587	+	13	11	9.197
OSJapC10_N_TAR_551	19810694	420	-	9	5	8.714
OSJapC10_N_TAR_552	19917870	2191	-	48	33	11.097
OSJapC10_N_TAR_127	19917965	413	+	9	9	8.690
OSJapC10_N_TAR_128	19939967	497	+	11	5	8.957
OSJapC10_N_TAR_553	19939967	497	-	11	5	8.957
OSJapC10_N_TAR_132	20122819	810	+	18	15	9.662
OSJapC10_N_TAR_555	20122819	537	-	12	10	9.069
OSJapC10_N_TAR_134	20374134	893	+	19	18	9.803
OSJapC10_N_TAR_557	20374852	489	-	11	10	8.934
OSJapC10_N_TAR_558	20424222	359	-	8	8	8.488
OSJapC10_N_TAR_559	20483962	1183	-	26	21	10.208
OSJapC10_N_TAR_138	20497458	478	+	10	8	8.901
OSJapC10_N_TAR_560	20520759	636	-	14	13	9.313
OSJapC10_N_TAR_561	20596461	1042	-	23	18	10.025
OSJapC10_N_TAR_562	20708105	1668	-	36	28	10.704
OSJapC10_N_TAR_139	20708284	760	+	17	13	9.570
OSJapC10_N_TAR_567	20911733	441	-	10	8	8.785
OSJapC10_N_TAR_568	21047573	267	-	6	6	8.061
OSJapC10_N_TAR_142	21063486	751	+	16	16	9.553

OSJapC10_N_TAR_569	21063486	751	-	16	16	9.553
OSJapC10_N_TAR_570	21182178	2095	-	46	27	11.033
OSJapC10_N_TAR_144	21330946	437	+	10	9	8.771
OSJapC10_N_TAR_145	21349112	367	+	8	8	8.520
OSJapC10_N_TAR_146	21352997	359	+	8	8	8.488
OSJapC10_N_TAR_147	21367562	221	+	5	5	7.788
OSJapC10_N_TAR_148	21373867	500	+	11	9	8.966
OSJapC10_N_TAR_149	21425385	451	+	10	10	8.817
OSJapC10_N_TAR_573	21444099	267	-	6	6	8.061
OSJapC10_N_TAR_150	21506667	313	+	7	7	8.290
OSJapC10_N_TAR_151	21644981	221	+	5	5	7.788
OSJapC10_N_TAR_574	21645165	526	-	11	9	9.039
OSJapC10_N_TAR_152	21656513	267	+	6	6	8.061
OSJapC10_N_TAR_576	21826386	359	-	8	8	8.488
OSJapC10_N_TAR_577	21876349	533	-	12	6	9.058
OSJapC10_N_TAR_578	21880940	451	-	10	10	8.817
OSJapC10_N_TAR_155	22207184	1028	+	22	15	10.006
OSJapC10_N_TAR_156	22220838	465	+	10	10	8.861
OSJapC10_N_TAR_583	22283518	291	-	6	6	8.185
OSJapC10_N_TAR_157	22293639	412	+	9	8	8.687
OSJapC10_N_TAR_584	22293685	1562	-	34	30	10.609
OSJapC10_N_TAR_158	22310458	454	+	10	6	8.827
OSJapC10_N_TAR_585	22310458	454	-	10	6	8.827
OSJapC10_N_TAR_586	22346565	628	-	14	9	9.295
OSJapC10_N_TAR_587	22349650	451	-	10	10	8.817
OSJapC10_N_TAR_159	22372482	982	+	21	20	9.940
OSJapC10_N_TAR_588	22372574	843	-	18	17	9.719
OSJapC10_N_TAR_160	22376223	703	+	15	15	9.457
OSJapC10_N_TAR_589	22376223	2287	-	50	34	11.159
OSJapC10_N_TAR_164	22518578	1069	+	23	13	10.062
OSJapC10_S_TAR_329	26784	221	+	5	5	7.788
OSJapC10_S_TAR_2	71857	322	-	7	7	8.331
OSJapC10_S_TAR_330	72556	313	+	7	7	8.290
OSJapC10_S_TAR_3	92208	283	-	6	6	8.145
OSJapC10_S_TAR_331	135203	347	+	8	7	8.439
OSJapC10_S_TAR_4	141626	451	-	10	7	8.817
OSJapC10_S_TAR_5	231885	757	-	16	15	9.564
OSJapC10_S_TAR_332	277765	272	+	6	6	8.087
OSJapC10_S_TAR_6	280808	457	-	10	10	8.836
OSJapC10_S_TAR_333	282182	603	+	13	12	9.236
OSJapC10_S_TAR_7	298330	366	-	8	8	8.516
OSJapC10_S_TAR_8	341806	370	-	8	8	8.531
OSJapC10_S_TAR_334	341852	616	+	13	13	9.267
OSJapC10_S_TAR_9	447814	392	-	9	7	8.615
OSJapC10_S_TAR_335	477749	1271	+	28	27	10.312
OSJapC10_S_TAR_10	477979	1616	-	35	27	10.658
OSJapC10_S_TAR_11	499650	1923	-	42	42	10.909
OSJapC10_S_TAR_13	530699	2104	-	46	35	11.039
OSJapC10_S_TAR_14	654619	306	-	7	6	8.257
OSJapC10_S_TAR_16	697807	497	-	11	11	8.957
OSJapC10_S_TAR_338	743324	266	+	6	6	8.055
OSJapC10_S_TAR_18	829765	453	-	10	8	8.823
OSJapC10_S_TAR_339	830135	589	+	13	13	9.202
OSJapC10_S_TAR_340	900541	405	+	9	9	8.662
OSJapC10_S_TAR_19	901760	359	-	8	8	8.488
OSJapC10_S_TAR_341	964689	658	+	14	14	9.362
OSJapC10_S_TAR_21	964735	750	-	16	16	9.551
OSJapC10_S_TAR_22	1001683	223	-	5	5	7.801
OSJapC10_S_TAR_343	1001683	223	+	5	5	7.801
OSJapC10_S_TAR_23	1096106	972	-	21	21	9.925
OSJapC10_S_TAR_24	1103477	1730	-	38	27	10.757
OSJapC10_S_TAR_25	1108006	899	-	20	19	9.812

OSJapC10_S_TAR_345	1108052	1267	+	28	27	10.307
OSJapC10_S_TAR_26	1183804	643	-	14	14	9.329
OSJapC10_S_TAR_27	1191369	498	-	11	11	8.960
OSJapC10_S_TAR_346	1282560	405	+	9	9	8.662
OSJapC10_S_TAR_29	1403502	546	-	12	12	9.093
OSJapC10_S_TAR_30	1553507	885	-	19	19	9.790
OSJapC10_S_TAR_347	1554125	403	+	9	9	8.655
OSJapC10_S_TAR_348	1568904	485	+	11	9	8.922
OSJapC10_S_TAR_31	1569194	609	-	13	12	9.250
OSJapC10_S_TAR_33	1788360	871	-	19	18	9.767
OSJapC10_S_TAR_34	1796462	471	-	10	10	8.880
OSJapC10_S_TAR_350	1851452	367	+	8	6	8.520
OSJapC10_S_TAR_35	1941550	340	-	7	5	8.409
OSJapC10_S_TAR_352	2080089	497	+	11	11	8.957
OSJapC10_S_TAR_38	2097302	413	-	9	9	8.690
OSJapC10_S_TAR_354	2192800	497	+	11	11	8.957
OSJapC10_S_TAR_355	2316215	440	+	10	5	8.781
OSJapC10_S_TAR_42	2340509	681	-	15	15	9.412
OSJapC10_S_TAR_43	2342685	461	-	10	10	8.849
OSJapC10_S_TAR_44	2374779	472	-	10	10	8.883
OSJapC10_S_TAR_356	2420660	822	+	18	17	9.683
OSJapC10_S_TAR_357	2433660	499	+	11	11	8.963
OSJapC10_S_TAR_358	2565126	497	+	11	11	8.957
OSJapC10_S_TAR_46	2586562	448	-	10	6	8.807
OSJapC10_S_TAR_47	2678645	300	-	7	5	8.229
OSJapC10_S_TAR_359	2770983	497	+	11	11	8.957
OSJapC10_S_TAR_49	2821513	362	-	8	8	8.500
OSJapC10_S_TAR_360	2821513	1280	+	28	28	10.322
OSJapC10_S_TAR_361	2845139	322	+	7	6	8.331
OSJapC10_S_TAR_362	2875556	418	+	9	8	8.707
OSJapC10_S_TAR_363	2886735	864	+	19	19	9.755
OSJapC10_S_TAR_50	2887102	313	-	7	7	8.290
OSJapC10_S_TAR_51	2930395	459	-	10	10	8.842
OSJapC10_S_TAR_52	2938625	455	-	10	10	8.830
OSJapC10_S_TAR_364	2977282	610	+	13	13	9.253
OSJapC10_S_TAR_53	3012541	402	-	9	9	8.651
OSJapC10_S_TAR_54	3018328	447	-	10	9	8.804
OSJapC10_S_TAR_55	3023119	637	-	14	6	9.315
OSJapC10_S_TAR_56	3055439	425	-	9	6	8.731
OSJapC10_S_TAR_57	3072000	451	-	10	10	8.817
OSJapC10_S_TAR_58	3384674	405	-	9	9	8.662
OSJapC10_S_TAR_366	3385180	2907	+	63	61	11.505
OSJapC10_S_TAR_59	3387205	836	-	18	18	9.707
OSJapC10_S_TAR_60	3532235	316	-	7	5	8.304
OSJapC10_S_TAR_368	3617435	375	+	8	8	8.551
OSJapC10_S_TAR_369	3652989	313	+	7	7	8.290
OSJapC10_S_TAR_61	3656095	615	-	13	8	9.264
OSJapC10_S_TAR_62	3676036	359	-	8	8	8.488
OSJapC10_S_TAR_371	3705720	343	+	7	7	8.422
OSJapC10_S_TAR_63	3747253	405	-	9	9	8.662
OSJapC10_S_TAR_372	3751235	625	+	14	13	9.288
OSJapC10_S_TAR_64	3922633	327	-	7	7	8.353
OSJapC10_S_TAR_65	4004732	313	-	7	7	8.290
OSJapC10_S_TAR_66	4148938	406	-	9	9	8.665
OSJapC10_S_TAR_375	4149172	264	+	6	6	8.044
OSJapC10_S_TAR_67	4163673	610	-	13	10	9.253
OSJapC10_S_TAR_68	4174863	481	-	10	10	8.910
OSJapC10_S_TAR_69	4200328	1257	-	27	27	10.296
OSJapC10_S_TAR_376	4200374	475	+	10	10	8.892
OSJapC10_S_TAR_70	4408776	2105	-	46	42	11.040
OSJapC10_S_TAR_378	4412400	396	+	9	8	8.629
OSJapC10_S_TAR_71	4524760	727	-	16	16	9.506

OSJapC10_S_TAR_379	4525036	572	+	12	12	9.160
OSJapC10_S_TAR_380	4533615	481	+	10	8	8.910
OSJapC10_S_TAR_382	4806445	245	+	5	5	7.937
OSJapC10_S_TAR_384	4863021	2068	+	45	36	11.014
OSJapC10_S_TAR_73	4880418	267	-	6	6	8.061
OSJapC10_S_TAR_385	4920897	1945	+	42	19	10.926
OSJapC10_S_TAR_74	4922180	662	-	14	13	9.371
OSJapC10_S_TAR_386	5088160	1711	+	37	24	10.741
OSJapC10_S_TAR_76	5149462	635	-	14	14	9.311
OSJapC10_S_TAR_77	5153313	622	-	14	7	9.281
OSJapC10_S_TAR_78	5162248	1669	-	36	34	10.705
OSJapC10_S_TAR_79	5166167	267	-	6	6	8.061
OSJapC10_S_TAR_80	5208283	675	-	15	9	9.399
OSJapC10_S_TAR_81	5329890	822	-	18	18	9.683
OSJapC10_S_TAR_389	5411773	341	+	7	7	8.414
OSJapC10_S_TAR_82	5437286	354	-	8	8	8.468
OSJapC10_S_TAR_390	5437470	630	+	14	14	9.299
OSJapC10_S_TAR_83	5462714	451	-	10	10	8.817
OSJapC10_S_TAR_391	5468296	359	+	8	8	8.488
OSJapC10_S_TAR_392	5567966	482	+	10	10	8.913
OSJapC10_S_TAR_84	5569855	359	-	8	8	8.488
OSJapC10_S_TAR_394	5621746	313	+	7	7	8.290
OSJapC10_S_TAR_395	5633222	451	+	10	10	8.817
OSJapC10_S_TAR_396	5690954	221	+	5	5	7.788
OSJapC10_S_TAR_88	5731580	543	-	12	12	9.085
OSJapC10_S_TAR_89	5774808	430	-	9	9	8.748
OSJapC10_S_TAR_90	5849196	705	-	15	15	9.461
OSJapC10_S_TAR_398	5849242	659	+	14	14	9.364
OSJapC10_S_TAR_91	5927025	743	-	16	10	9.537
OSJapC10_S_TAR_92	6012611	458	-	10	10	8.839
OSJapC10_S_TAR_93	6022105	957	-	21	20	9.902
OSJapC10_S_TAR_400	6139139	617	+	13	11	9.269
OSJapC10_S_TAR_95	6191284	1597	-	35	35	10.641
OSJapC10_S_TAR_401	6194091	1206	+	26	15	10.236
OSJapC10_S_TAR_96	6226810	635	-	14	14	9.311
OSJapC10_S_TAR_402	6226994	497	+	11	11	8.957
OSJapC10_S_TAR_97	6250533	1264	-	27	21	10.304
OSJapC10_S_TAR_98	6253871	247	-	5	5	7.948
OSJapC10_S_TAR_403	6256034	543	+	12	12	9.085
OSJapC10_S_TAR_405	6345081	1676	+	36	32	10.711
OSJapC10_S_TAR_99	6352881	464	-	10	10	8.858
OSJapC10_S_TAR_406	6364516	2059	+	45	44	11.008
OSJapC10_S_TAR_407	6413680	603	+	13	6	9.236
OSJapC10_S_TAR_101	6540900	399	-	9	8	8.640
OSJapC10_S_TAR_408	6540900	399	+	9	8	8.640
OSJapC10_S_TAR_102	6565732	617	-	13	13	9.269
OSJapC10_S_TAR_409	6615031	997	+	22	17	9.961
OSJapC10_S_TAR_105	6880993	314	-	7	6	8.295
OSJapC10_S_TAR_410	6926459	417	+	9	5	8.704
OSJapC10_S_TAR_106	6946643	555	-	12	11	9.116
OSJapC10_S_TAR_107	6976089	497	-	11	11	8.957
OSJapC10_S_TAR_109	7031647	2093	-	46	40	11.031
OSJapC10_S_TAR_110	7077149	313	-	7	7	8.290
OSJapC10_S_TAR_112	7138169	467	-	10	10	8.867
OSJapC10_S_TAR_113	7163052	652	-	14	14	9.349
OSJapC10_S_TAR_414	7164301	393	+	9	8	8.618
OSJapC10_S_TAR_114	7178460	455	-	10	10	8.830
OSJapC10_S_TAR_115	7196194	542	-	12	11	9.082
OSJapC10_S_TAR_116	7246326	2213	-	48	33	11.112
OSJapC10_S_TAR_415	7246381	2158	+	47	32	11.075
OSJapC10_S_TAR_117	7251116	758	-	16	15	9.566
OSJapC10_S_TAR_416	7263056	299	+	7	6	8.224

OSJapC10_S_TAR_119	7419929	1436	-	31	30	10.488
OSJapC10_S_TAR_417	7420114	1021	+	22	21	9.996
OSJapC10_S_TAR_418	7426189	681	+	15	15	9.412
OSJapC10_S_TAR_121	7443285	271	-	6	6	8.082
OSJapC10_S_TAR_419	7522711	285	+	6	6	8.155
OSJapC10_S_TAR_124	7680158	1882	-	41	39	10.878
OSJapC10_S_TAR_422	7718905	481	+	10	10	8.910
OSJapC10_S_TAR_424	8055796	495	+	11	11	8.951
OSJapC10_S_TAR_127	8121111	1876	-	41	40	10.873
OSJapC10_S_TAR_128	8174713	497	-	11	11	8.957
OSJapC10_S_TAR_130	8223048	681	-	15	13	9.412
OSJapC10_S_TAR_131	8487289	267	-	6	6	8.061
OSJapC10_S_TAR_132	8507482	359	-	8	8	8.488
OSJapC10_S_TAR_134	8600028	462	-	10	10	8.852
OSJapC10_S_TAR_135	8604335	345	-	8	7	8.430
OSJapC10_S_TAR_428	8821448	313	+	7	7	8.290
OSJapC10_S_TAR_138	9005972	390	-	8	5	8.607
OSJapC10_S_TAR_139	9024062	866	-	19	13	9.758
OSJapC10_S_TAR_140	9032491	1156	-	25	19	10.175
OSJapC10_S_TAR_141	9044315	589	-	13	13	9.202
OSJapC10_S_TAR_143	9133750	589	-	13	13	9.202
OSJapC10_S_TAR_144	9174522	313	-	7	5	8.290
OSJapC10_S_TAR_145	9388235	1653	-	36	16	10.691
OSJapC10_S_TAR_431	9389437	497	+	11	11	8.957
OSJapC10_S_TAR_146	9461777	359	-	8	8	8.488
OSJapC10_S_TAR_432	9505590	372	+	8	7	8.539
OSJapC10_S_TAR_147	9505636	326	-	7	6	8.349
OSJapC10_S_TAR_149	9595359	834	-	18	18	9.704
OSJapC10_S_TAR_433	9652566	308	+	7	7	8.267
OSJapC10_S_TAR_150	9718516	724	-	16	8	9.500
OSJapC10_S_TAR_434	9764230	332	+	7	6	8.375
OSJapC10_S_TAR_435	9870708	537	+	12	7	9.069
OSJapC10_S_TAR_151	9894102	339	-	7	7	8.405
OSJapC10_S_TAR_155	10080691	1107	-	24	20	10.112
OSJapC10_S_TAR_156	10146214	359	-	8	8	8.488
OSJapC10_S_TAR_437	10232502	225	+	5	5	7.814
OSJapC10_S_TAR_438	10234911	738	+	16	16	9.527
OSJapC10_S_TAR_157	10236524	451	-	10	10	8.817
OSJapC10_S_TAR_158	10253692	403	-	9	9	8.655
OSJapC10_S_TAR_439	10262242	267	+	6	6	8.061
OSJapC10_S_TAR_441	10319503	639	+	14	14	9.320
OSJapC10_S_TAR_442	10360250	925	+	20	18	9.853
OSJapC10_S_TAR_443	10366833	1987	+	43	36	10.956
OSJapC10_S_TAR_444	10409704	429	+	9	9	8.745
OSJapC10_S_TAR_167	10413513	465	-	10	10	8.861
OSJapC10_S_TAR_445	10436447	545	+	12	11	9.090
OSJapC10_S_TAR_168	10468274	1145	-	25	13	10.161
OSJapC10_S_TAR_446	10468394	448	+	10	5	8.807
OSJapC10_S_TAR_169	10539782	578	-	13	12	9.175
OSJapC10_S_TAR_448	10539984	1277	+	28	27	10.319
OSJapC10_S_TAR_170	10558445	1237	-	27	14	10.273
OSJapC10_S_TAR_171	10566371	362	-	8	6	8.500
OSJapC10_S_TAR_450	10603557	468	+	10	10	8.870
OSJapC10_S_TAR_172	10610718	451	-	10	10	8.817
OSJapC10_S_TAR_174	10672090	961	-	21	21	9.908
OSJapC10_S_TAR_176	10753374	762	-	17	16	9.574
OSJapC10_S_TAR_454	10753422	1003	+	22	19	9.970
OSJapC10_S_TAR_177	10756329	776	-	17	17	9.600
OSJapC10_S_TAR_178	10768503	559	-	12	12	9.127
OSJapC10_S_TAR_455	10768503	789	+	17	17	9.624
OSJapC10_S_TAR_456	10775786	457	+	10	10	8.836
OSJapC10_S_TAR_457	10907728	719	+	16	15	9.490

OSJapC10_S_TAR_458	10921300	506	+	11	11	8.983
OSJapC10_S_TAR_180	10974945	235	-	5	5	7.877
OSJapC10_S_TAR_182	11049073	492	-	11	11	8.943
OSJapC10_S_TAR_459	11104557	794	+	17	17	9.633
OSJapC10_S_TAR_183	11104603	405	-	9	9	8.662
OSJapC10_S_TAR_184	11116355	512	-	11	9	9.000
OSJapC10_S_TAR_185	11204971	753	-	16	16	9.557
OSJapC10_S_TAR_460	11204971	689	+	15	15	9.428
OSJapC10_S_TAR_186	11258696	594	-	13	9	9.214
OSJapC10_S_TAR_461	11258936	354	+	8	5	8.468
OSJapC10_S_TAR_187	11273013	635	-	14	14	9.311
OSJapC10_S_TAR_462	11451402	497	+	11	11	8.957
OSJapC10_S_TAR_463	11469332	359	+	8	8	8.488
OSJapC10_S_TAR_464	11708836	667	+	15	10	9.382
OSJapC10_S_TAR_190	11717568	432	-	9	8	8.755
OSJapC10_S_TAR_191	11725683	221	-	5	5	7.788
OSJapC10_S_TAR_465	11965430	407	+	9	5	8.669
OSJapC10_S_TAR_192	12359373	496	-	11	11	8.954
OSJapC10_S_TAR_469	12469913	316	+	7	7	8.304
OSJapC10_S_TAR_470	12483404	417	+	9	9	8.704
OSJapC10_S_TAR_471	12501673	769	+	17	17	9.587
OSJapC10_S_TAR_193	12501765	631	-	14	14	9.301
OSJapC10_S_TAR_194	12574914	463	-	10	10	8.855
OSJapC10_S_TAR_196	12664785	632	-	14	13	9.304
OSJapC10_S_TAR_197	12789214	375	-	8	8	8.551
OSJapC10_S_TAR_472	12789306	358	+	8	7	8.484
OSJapC10_S_TAR_198	12810187	1691	-	37	19	10.724
OSJapC10_S_TAR_473	12810509	476	+	10	6	8.895
OSJapC10_S_TAR_474	12862313	443	+	10	8	8.791
OSJapC10_S_TAR_201	13027166	442	-	10	5	8.788
OSJapC10_S_TAR_202	13116591	451	-	10	10	8.817
OSJapC10_S_TAR_476	13139692	850	+	18	17	9.731
OSJapC10_S_TAR_477	13151442	272	+	6	5	8.087
OSJapC10_S_TAR_478	13198880	414	+	9	9	8.693
OSJapC10_S_TAR_203	13354025	1480	-	32	27	10.531
OSJapC10_S_TAR_479	13354025	1480	+	32	27	10.531
OSJapC10_S_TAR_480	13404610	2049	+	45	36	11.001
OSJapC10_S_TAR_204	13471905	451	-	10	10	8.817
OSJapC10_S_TAR_205	13490278	487	-	11	9	8.928
OSJapC10_S_TAR_206	13513884	359	-	8	8	8.488
OSJapC10_S_TAR_481	13513884	359	+	8	8	8.488
OSJapC10_S_TAR_483	13603875	359	+	8	8	8.488
OSJapC10_S_TAR_208	13606787	819	-	18	18	9.678
OSJapC10_S_TAR_484	13610462	1778	+	39	26	10.796
OSJapC10_S_TAR_485	13677311	594	+	13	7	9.214
OSJapC10_S_TAR_486	13698606	463	+	10	7	8.855
OSJapC10_S_TAR_210	13698698	371	-	8	5	8.535
OSJapC10_S_TAR_211	13795771	313	-	7	7	8.290
OSJapC10_S_TAR_212	13814528	697	-	15	15	9.445
OSJapC10_S_TAR_213	13850385	649	-	14	14	9.342
OSJapC10_S_TAR_487	13959101	2232	+	49	47	11.124
OSJapC10_S_TAR_214	13959883	1358	-	30	28	10.407
OSJapC10_S_TAR_215	13977873	543	-	12	12	9.085
OSJapC10_S_TAR_488	13979717	451	+	10	10	8.817
OSJapC10_S_TAR_216	14009950	680	-	15	15	9.409
OSJapC10_S_TAR_218	14064292	666	-	14	14	9.379
OSJapC10_S_TAR_489	14064292	2495	+	54	53	11.285
OSJapC10_S_TAR_219	14070283	501	-	11	11	8.969
OSJapC10_S_TAR_491	14167363	360	+	8	6	8.492
OSJapC10_S_TAR_222	14324506	411	-	9	9	8.683
OSJapC10_S_TAR_492	14324598	319	+	7	7	8.317
OSJapC10_S_TAR_223	14411710	512	-	11	11	9.000

OSJapC10_S_TAR_493	14411710	655	+	14	14	9.355
OSJapC10_S_TAR_494	14415325	439	+	10	9	8.778
OSJapC10_S_TAR_495	14438880	789	+	17	15	9.624
OSJapC10_S_TAR_224	14469411	599	-	13	13	9.226
OSJapC10_S_TAR_498	14583167	554	+	12	8	9.114
OSJapC10_S_TAR_499	14597926	432	+	9	9	8.755
OSJapC10_S_TAR_227	14702336	411	-	9	6	8.683
OSJapC10_S_TAR_500	14702336	411	+	9	6	8.683
OSJapC10_S_TAR_228	14752937	401	-	9	9	8.647
OSJapC10_S_TAR_230	14823508	384	-	8	8	8.585
OSJapC10_S_TAR_501	14852672	405	+	9	9	8.662
OSJapC10_S_TAR_231	14853657	421	-	9	9	8.718
OSJapC10_S_TAR_232	15086291	978	-	21	16	9.934
OSJapC10_S_TAR_502	15086291	1048	+	23	17	10.033
OSJapC10_S_TAR_503	15170512	2129	+	46	45	11.056
OSJapC10_S_TAR_234	15259203	446	-	10	9	8.801
OSJapC10_S_TAR_235	15336313	451	-	10	10	8.817
OSJapC10_S_TAR_236	15339533	683	-	15	15	9.416
OSJapC10_S_TAR_506	15407638	359	+	8	8	8.488
OSJapC10_S_TAR_237	15410245	716	-	16	15	9.484
OSJapC10_S_TAR_238	15472157	497	-	11	11	8.957
OSJapC10_S_TAR_239	15493415	755	-	16	16	9.560
OSJapC10_S_TAR_507	15493507	617	+	13	13	9.269
OSJapC10_S_TAR_509	15504103	267	+	6	6	8.061
OSJapC10_S_TAR_510	15530122	659	+	14	14	9.364
OSJapC10_S_TAR_241	15601963	564	-	12	9	9.140
OSJapC10_S_TAR_513	15801475	400	+	9	9	8.644
OSJapC10_S_TAR_242	15842254	637	-	14	14	9.315
OSJapC10_S_TAR_514	15843244	313	+	7	7	8.290
OSJapC10_S_TAR_515	15861172	316	+	7	7	8.304
OSJapC10_S_TAR_243	15895609	415	-	9	8	8.697
OSJapC10_S_TAR_516	15895769	335	+	7	6	8.388
OSJapC10_S_TAR_244	15898988	1372	-	30	29	10.422
OSJapC10_S_TAR_517	15898988	1540	+	33	32	10.589
OSJapC10_S_TAR_518	15969995	2829	+	62	29	11.466
OSJapC10_S_TAR_246	16083720	405	-	9	9	8.662
OSJapC10_S_TAR_521	16130833	456	+	10	6	8.833
OSJapC10_S_TAR_522	16241330	617	+	13	13	9.269
OSJapC10_S_TAR_523	16263904	540	+	12	12	9.077
OSJapC10_S_TAR_524	16314368	296	+	6	6	8.209
OSJapC10_S_TAR_525	16369622	460	+	10	8	8.845
OSJapC10_S_TAR_526	16495837	267	+	6	6	8.061
OSJapC10_S_TAR_249	16513904	627	-	14	7	9.292
OSJapC10_S_TAR_527	16803902	475	+	10	10	8.892
OSJapC10_S_TAR_529	16868852	751	+	16	13	9.553
OSJapC10_S_TAR_252	16869312	853	-	19	9	9.736
OSJapC10_S_TAR_253	16879653	421	-	9	9	8.718
OSJapC10_S_TAR_255	16886387	2410	-	52	46	11.235
OSJapC10_S_TAR_256	16905055	415	-	9	9	8.697
OSJapC10_S_TAR_531	16936832	263	+	6	6	8.039
OSJapC10_S_TAR_257	16980812	494	-	11	11	8.948
OSJapC10_S_TAR_532	17025956	611	+	13	9	9.255
OSJapC10_S_TAR_258	17028510	272	-	6	6	8.087
OSJapC10_S_TAR_533	17033408	3929	+	85	78	11.940
OSJapC10_S_TAR_259	17034896	2599	-	57	52	11.344
OSJapC10_S_TAR_260	17054872	681	-	15	15	9.412
OSJapC10_S_TAR_534	17061042	329	+	7	6	8.362
OSJapC10_S_TAR_261	17095517	738	-	16	16	9.527
OSJapC10_S_TAR_535	17096494	635	+	14	14	9.311
OSJapC10_S_TAR_536	17141489	373	+	8	5	8.543
OSJapC10_S_TAR_262	17209094	563	-	12	11	9.137
OSJapC10_S_TAR_537	17209326	423	+	9	9	8.725

OSJapC10_S_TAR_538	17304825	419	+	9	8	8.711
OSJapC10_S_TAR_539	17379472	2074	+	45	39	11.018
OSJapC10_S_TAR_540	17385115	359	+	8	8	8.488
OSJapC10_S_TAR_541	17396538	305	+	7	6	8.253
OSJapC10_S_TAR_542	17434738	789	+	17	17	9.624
OSJapC10_S_TAR_263	17434784	497	-	11	11	8.957
OSJapC10_S_TAR_264	17497099	829	-	18	18	9.695
OSJapC10_S_TAR_543	17498212	451	+	10	10	8.817
OSJapC10_S_TAR_544	17521163	476	+	10	10	8.895
OSJapC10_S_TAR_265	17528714	461	-	10	10	8.849
OSJapC10_S_TAR_266	17530636	1240	-	27	15	10.276
OSJapC10_S_TAR_545	17530636	1394	+	30	18	10.445
OSJapC10_S_TAR_267	17542672	361	-	8	8	8.496
OSJapC10_S_TAR_268	17569445	516	-	11	11	9.011
OSJapC10_S_TAR_269	17573245	655	-	14	13	9.355
OSJapC10_S_TAR_546	17609665	1785	+	39	39	10.802
OSJapC10_S_TAR_547	17626050	598	+	13	13	9.224
OSJapC10_S_TAR_549	17676986	451	+	10	10	8.817
OSJapC10_S_TAR_270	17681137	458	-	10	10	8.839
OSJapC10_S_TAR_271	17728575	681	-	15	13	9.412
OSJapC10_S_TAR_550	17728575	549	+	12	11	9.101
OSJapC10_S_TAR_272	17838996	550	-	12	10	9.103
OSJapC10_S_TAR_551	17838996	1517	+	33	21	10.567
OSJapC10_S_TAR_273	18029268	928	-	20	20	9.858
OSJapC10_S_TAR_552	18029452	744	+	16	16	9.539
OSJapC10_S_TAR_274	18076207	443	-	10	9	8.791
OSJapC10_S_TAR_553	18076253	271	+	6	6	8.082
OSJapC10_S_TAR_275	18079081	818	-	18	16	9.676
OSJapC10_S_TAR_554	18079327	526	+	11	11	9.039
OSJapC10_S_TAR_555	18144557	519	+	11	10	9.020
OSJapC10_S_TAR_556	18150670	506	+	11	11	8.983
OSJapC10_S_TAR_276	18405450	753	-	16	14	9.557
OSJapC10_S_TAR_557	18405450	856	+	19	16	9.741
OSJapC10_S_TAR_277	18459976	666	-	14	9	9.379
OSJapC10_S_TAR_278	18527281	379	-	8	8	8.566
OSJapC10_S_TAR_279	18537098	275	-	6	6	8.103
OSJapC10_S_TAR_558	18537098	3090	+	67	62	11.593
OSJapC10_S_TAR_559	18774860	1812	+	39	38	10.823
OSJapC10_S_TAR_280	18783960	267	-	6	6	8.061
OSJapC10_S_TAR_560	18790769	330	+	7	7	8.366
OSJapC10_S_TAR_561	18830376	221	+	5	5	7.788
OSJapC10_S_TAR_281	18835703	824	-	18	16	9.687
OSJapC10_S_TAR_562	18881755	267	+	6	6	8.061
OSJapC10_S_TAR_563	19048342	1432	+	31	31	10.484
OSJapC10_S_TAR_282	19057459	1989	-	43	39	10.958
OSJapC10_S_TAR_564	19057459	1989	+	43	39	10.958
OSJapC10_S_TAR_283	19086869	355	-	8	8	8.472
OSJapC10_S_TAR_565	19149811	369	+	8	5	8.527
OSJapC10_S_TAR_566	19191287	1424	+	31	26	10.476
OSJapC10_S_TAR_284	19287810	313	-	7	7	8.290
OSJapC10_S_TAR_567	19287810	451	+	10	10	8.817
OSJapC10_S_TAR_285	19290572	446	-	10	10	8.801
OSJapC10_S_TAR_568	19291487	789	+	17	17	9.624
OSJapC10_S_TAR_569	19293682	497	+	11	11	8.957
OSJapC10_S_TAR_286	19329215	393	-	9	8	8.618
OSJapC10_S_TAR_570	19329433	369	+	8	8	8.527
OSJapC10_S_TAR_287	19364581	1078	-	23	22	10.074
OSJapC10_S_TAR_571	19364581	1078	+	23	22	10.074
OSJapC10_S_TAR_288	19368322	1148	-	25	23	10.165
OSJapC10_S_TAR_573	19372959	2184	+	47	45	11.093
OSJapC10_S_TAR_289	19374307	981	-	21	21	9.938
OSJapC10_S_TAR_290	19388082	299	-	7	6	8.224

OSJapC10_S_TAR_291	19471668	870	-	19	19	9.765
OSJapC10_S_TAR_574	19471990	502	+	11	11	8.972
OSJapC10_S_TAR_292	19488273	1416	-	31	28	10.468
OSJapC10_S_TAR_575	19488736	644	+	14	14	9.331
OSJapC10_S_TAR_577	19539539	484	+	11	9	8.919
OSJapC10_S_TAR_578	19554201	313	+	7	7	8.290
OSJapC10_S_TAR_293	19575269	496	-	11	10	8.954
OSJapC10_S_TAR_294	19700298	373	-	8	6	8.543
OSJapC10_S_TAR_579	19701318	697	+	15	14	9.445
OSJapC10_S_TAR_295	19718259	873	-	19	15	9.770
OSJapC10_S_TAR_580	19718518	614	+	13	12	9.262
OSJapC10_S_TAR_296	19787579	477	-	10	5	8.898
OSJapC10_S_TAR_298	20069208	678	-	15	10	9.405
OSJapC10_S_TAR_581	20163795	789	+	17	17	9.624
OSJapC10_S_TAR_582	20249257	424	+	9	9	8.728
OSJapC10_S_TAR_583	20313089	826	+	18	16	9.690
OSJapC10_S_TAR_584	20315701	1565	+	34	22	10.612
OSJapC10_S_TAR_585	20423299	730	+	16	16	9.512
OSJapC10_S_TAR_586	20444563	3333	+	72	54	11.703
OSJapC10_S_TAR_299	20444797	3981	-	87	57	11.959
OSJapC10_S_TAR_587	20521128	497	+	11	11	8.957
OSJapC10_S_TAR_301	20542292	635	-	14	14	9.311
OSJapC10_S_TAR_302	20586385	489	-	11	8	8.934
OSJapC10_S_TAR_588	20586431	443	+	10	7	8.791
OSJapC10_S_TAR_589	20597052	359	+	8	8	8.488
OSJapC10_S_TAR_304	20682999	1133	-	25	20	10.146
OSJapC10_S_TAR_590	20683587	1892	+	41	35	10.886
OSJapC10_S_TAR_591	20732916	485	+	11	10	8.922
OSJapC10_S_TAR_306	20734032	313	-	7	7	8.290
OSJapC10_S_TAR_307	20746765	464	-	10	10	8.858
OSJapC10_S_TAR_592	20785263	359	+	8	8	8.488
OSJapC10_S_TAR_308	20860342	452	-	10	10	8.820
OSJapC10_S_TAR_593	20911733	614	+	13	11	9.262
OSJapC10_S_TAR_594	21047665	451	+	10	10	8.817
OSJapC10_S_TAR_595	21069904	359	+	8	8	8.488
OSJapC10_S_TAR_309	21245728	1736	-	38	35	10.762
OSJapC10_S_TAR_597	21245919	986	+	21	20	9.945
OSJapC10_S_TAR_598	21313955	773	+	17	17	9.594
OSJapC10_S_TAR_310	21316654	543	-	12	12	9.085
OSJapC10_S_TAR_311	21323451	497	-	11	11	8.957
OSJapC10_S_TAR_312	21337897	267	-	6	6	8.061
OSJapC10_S_TAR_314	21369385	695	-	15	12	9.441
OSJapC10_S_TAR_599	21369431	649	+	14	11	9.342
OSJapC10_S_TAR_315	21499744	1593	-	35	29	10.638
OSJapC10_S_TAR_601	21499744	1756	+	38	32	10.778
OSJapC10_S_TAR_316	21503356	1969	-	43	30	10.943
OSJapC10_S_TAR_317	21693776	405	-	9	9	8.662
OSJapC10_S_TAR_603	21762399	752	+	16	11	9.555
OSJapC10_S_TAR_318	21762786	779	-	17	17	9.605
OSJapC10_S_TAR_604	21861614	1265	+	28	27	10.305
OSJapC10_S_TAR_319	21861715	612	-	13	13	9.257
OSJapC10_S_TAR_320	21913211	1623	-	35	28	10.664
OSJapC10_S_TAR_606	21913211	1623	+	35	28	10.664
OSJapC10_S_TAR_321	21926453	221	-	5	5	7.788
OSJapC10_S_TAR_607	22101548	1000	+	22	18	9.966
OSJapC10_S_TAR_322	22156266	323	-	7	7	8.335
OSJapC10_S_TAR_324	22220792	782	-	17	15	9.611
OSJapC10_S_TAR_608	22340737	498	+	11	9	8.960
OSJapC10_S_TAR_609	22346473	536	+	12	7	9.066
OSJapC10_S_TAR_610	22515483	1142	+	25	25	10.157
OSJapC10_S_TAR_327	22515897	406	-	9	9	8.665
OSJapC10_S_TAR_328	22517796	1282	-	28	22	10.324

[1] Starting position of the TAR in the chromosome 10 sequence of the TIGR Rice Pseudomolecule release 2.
