# Supplementary material for: Tiling microarray analysis of rice chromosome 10 to identify the transcriptome and relate its expression to chromosomal architecture
Source: Genome Biol. 2005 May 27;6(6):R52. doi: 10.1186/gb-2005-6-6-r52 (PMC1175972; doi:10.1186/gb-2005-6-6-r52)
Supplement: Additional File 5 — Table S5: Sequence analysis of cloned intergenic TARs. Sequence analysis of cloned intergenic TARs. [file gb-2005-6-6-r52-S5.pdf]

Supplemental Table 5. Sequence analysis of cloned intergenic TARs

Clone	Length [1]	Identity	Strand	Position [2]	Overlap [3]
OsJapC10-NSC92	862	95.2%	+	15332324	None
OsJapC10-109_1	577	99.8%	+	7030732	None
OsJapC10-C181_1	1068	96.3%	+	5190911	None
OsJapC10-351	531	96.4%	+	2013854	None
OsJapC10-C301	846	100.0%	+	10329577	9638.m01768
OsJapC10-ZN185F3-2	392	99.7%	+	11204658	None
OsJapC10-288	353	99.7%	+	19369118	None
OsJapC10-NG239-1-SP6	619	94.8%	-	576216	None
OsJapC10-C31	653	99.4%	+	1037805	9638.m00173
OsJapC10-NG385-1	338	91.1%	-	3149445	None
OsJapC10-C282	1252	99.7%	+	9430385	Chr10_1469
OsJapC10-302	922	95.0%	+	20585975	9638.m03570
OsJapC10-30	524	98.3%	+	1553917	None
OsJapC10-NG330-1	729	99.7%	-	71785	None
OsJapC10-ZN376F3-2	1003	97.2%	+	4200587	None
OsJapC10-C198	1587	99.2%	+	5759822	9638.m00983
OsJapC10-NSC36	165	98.8%	+	2343060	9638.m00397
OsJapC10-C153	703	100.0%	+	4286061	9638.m00722
OsJapC10-C325	647	99.5%	-	11101115	9638.m01893
OsJapC10-NG570-1	664	99.8%	+	19329197	9638.m03306
OsJapC10-C183	991	99.1%	+	5283676	None
OsJapC10-30_1	524	98.3%	+	1553917	None
OsJapC10-C353_1	482	100.0%	+	12872588	9638.m02191
OsJapC10-314_2	1212	99.8%	+	21370560	9638.m03701
OsJapC10-96	403	100.0%	+	6227215	9638.m01067
OsJapC10-NG341-2	352	99.4%	+	10358277	9638.m01774
OsJapC10-203	636	99.7%	+	13354746	None
OsJapC10-NSC214-2	817	98.8%	-	22316268	None
OsJapC10-278-1	955	99.8%	+	18527230	9638.m03150
OsJapC10-S403	231	99.6%	+	6256026	9638.m01072
OsJapC10-C47_1	1529	99.9%	+	1448790	9638.m00250
OsJapC10-112	862	99.7%	+	7138217	9638.m01221
OsJapC10-NSC63F-2	282	98.9%	-	12366865	9638.m02106
OsJapC10-NSC184	980	96.5%	+	10494879	None
OsJapC10-314_1	746	99.7%	+	21370560	9638.m03701
OsJapC10-C242	501	99.8%	-	15928409	AK064310
OsJapC10-58	830	99.9%	+	3384412	None
OsJapC10-ZN49F1-2	827	99.5%	+	10312200	9638.m01767
OsJapC10-NSC306	541	99.8%	+	10769807	9638.m01840
OsJapC10-C564	816	99.5%	+	22607113	None
OsJapC10-59_1	550	99.8%	+	3387531	9638.m00577

OsJapC10-NG50	209	98.1%	+	16973905	9638.m02905
OsJapC10-NG50	209	98.1%	+	16973905	9638.m02905
OsJapC10-NG408-7	500	99.8%	+	10358129	9638.m01774
OsJapC10-NSC77	798	100.0%	+	14785752	None
OsJapC10-NG30-C2	306	99.3%	+	15928366	None
OsJapC10-NSC39	491	100.0%	+	13354649	None
OsJapC10-NG226-2	538	95.7%	-	2665329	None
OsJapC10-NG26-C1	188	98.4%	+	1184254	None
OsJapC10-C47_2	853	99.8%	+	1448789	9638.m00250
OsJapC10-347	536	97.9%	+	1553917	None
OsJapC10-NSC129_2	1191	99.3%	+	4200571	None
OsJapC10-NSC164	877	99.7%	+	19718735	9638.m03387
OsJapC10-43_2	373	98.1%	+	2343067	9638.m00397
OsJapC10-NG24-B1	535	99.8%	-	1102241	None
OsJapC10-NSC273_2	659	92.7%	+	6644414	None
OsJapC10-S376_2	914	99.6%	+	4200576	None
OsJapC10-C126_1	395	99.0%	-	3674140	None
OsJapC10-NG570-8	293	84.6%	+	11748779	None
OsJapC10-C204	271	100.0%	+	6167698	None
OsJapC10-NG334-1	354	99.7%	+	341692	None
OsJapC10-NSC37	200	100.0%	+	1215301	None
OsJapC10-NSC145_1	634	97.6%	+	5498803	9638.m00933
OsJapC10-NSC102	527	97.3%	-	15928405	AK064310
OsJapC10-NG185-2	178	99.4%	+	11204872	None
OsJapC10-ZN197F3-2	892	99.7%	-	12786906	9638.m02176
OsJapC10-NSC75F-6	313	97.1%	-	3005808	None
OsJapC10-NG239-1-T7	693	98.0%	+	575483	9638.m00096
OsJapC10-NG2-A1	795	99.4%	-	71768	None
OsJapC10-NSC199_3	1011	98.9%	+	13749843	None
OsJapC10-NSC111	910	99.2%	+	16024719	None
OsJapC10-C286	647	100.0%	+	9569166	9638.m01649
OsJapC10-318_1	631	100.0%	+	21763926	None
OsJapC10-NG503-1	1171	100.0%	+	616281	9638.m00103
OsJapC10-286	802	99.6%	+	19329060	9638.m03306
OsJapC10-C114	470	100.0%	+	3325835	9638.m00567
OsJapC10-NSC595F-2	1064	99.8%	+	21069995	None
OsJapC10-NSC98F1	169	100.0%	+	6253808	None
OsJapC10-NSC71F-1	539	99.8%	+	4524472	None
OsJapC10-NSC199F-3	548	99.8%	+	12537148	None

[1] Length of the clone that could be mapped to the target TAR. [2] Starting position of the mapped sequence in the chromosome 10 sequence of the TIGR Rice Pseudomolecule release 2. [3] Neiboring models with > 100 bp overlap.
