# Supplementary material for: Tiling microarray analysis of rice chromosome 10 to identify the transcriptome and relate its expression to chromosomal architecture
Source: Genome Biol. 2005 May 27;6(6):R52. doi: 10.1186/gb-2005-6-6-r52 (PMC1175972; doi:10.1186/gb-2005-6-6-r52)
Supplement: Additional File 6 — Table S6: Comparison of BGI and TIGR japonica chromosome 10 gene models. Comparison of BGI and TIGR japonica chromosome 10 gene models. [file gb-2005-6-6-r52-S6.pdf]

Supplemental Table 6. Comparison of BGI and TIGR *japonica* chromosome 10 gene models

BGI *japonica*										TIGR *japonica*									
Common					Unique					Common					Unique				
2323 (84.0)					441 (16.1)					2488 (82.4)					531 (17.6)				
Classification			Homology		Classification			Homology		Classification			Homology		Classification			Homology	
CG	EG	UG	HH	LH	CG	EG	UG	HH	LH	EP	PP	HP	HH	LH	EP	PP	HP	HH	LH
813 (35.0)	260 (11.2)	1250 (53.8)	1210 (52.1)	1113 (47.9)	130 (29.5)	12 (2.7)	299 (67.8)	156 (35.4)	285 (64.6)	655 (26.3)	878 (35.3)	955 (38.4)	1361 (54.7)	1127 (45.3)	44 (8.3)	111 (20.9)	376 (70.8)	142 (26.7)	389 (73.3)

TIGR *japonica* chromosome 10 non-TE protein-coding gene models were classified as 699 (18.1%) expressed proteins (EP; with expression evidence), 989 (25.6%) putative proteins (PP; with hits in non-redundant protein databases), and 1331 (34.5%) hypothetical proteins (HP; predicted genes otherwise without supporting evidence). See http://www.tigr.org/tdb/e2k1/osa1 for additional detail. BGI *japonica* models were classified into CG, EG and UG groups based on alignment to available full-length cDNA and EST sequences. See main text for detail. BGI and TIGR *japonica* chromosome 10 gene models were mapped against each other using BLAT. Gene models were considered common if they overlap > 100 bp in their annotated ORFs. Models overlap < 100 bp were regarded as unique.
