# Supplementary material for: Tiling microarray analysis of rice chromosome 10 to identify the transcriptome and relate its expression to chromosomal architecture
Source: Genome Biol. 2005 May 27;6(6):R52. doi: 10.1186/gb-2005-6-6-r52 (PMC1175972; doi:10.1186/gb-2005-6-6-r52)
Supplement: Additional File 7 — Table S7: Comparison of BGI indica and japonica chromosome 10 gene models. Comparison of BGI indica and japonica chromosome 10 gene models. [file gb-2005-6-6-r52-S7.pdf]

Supplemental Table 7. Comparison of BGI *indica* and *japonica* chromosome 10 gene models

		CG	EG	UG	All
	Total	821	328	1660	2809
	I	791 (96.4%)	303 (92.4%)	1326 (79.9%)	2420 (86.2%)
indica	II	25 (3.1%)	12 (3.7%)	102 (6.1%)	139 (5.0%)
	III	5 (0.6%)	13 (4.0%)	232 (14.0%)	250 (8.9%)
	IV	25	18	177	220
	Total	934	272	1549	2764
	I	860 (91.2%)	237 (87.1%)	1125 (72.6%)	2222 (80.4%)
japonica	II	33 (3.5%)	12 (4.4%)	207 (13.4%)	252 (9.1%)
	III	50 (5.3%)	23 (8.5%)	217 (14.0%)	290 (10.5%)
	IV	32	8	127	167

BGI *indica* and *japonica* chromosome 10 gene models were mapped to all the gene models of the reciprocal genomes, respectively, using BLAT. Gene models were considered mapped if they have > 90% identity over their entire lengths. (I) Chromosome 10 models mapped to chromosome 10 in the reciprocal genome; (II) Chromosome 10 models mapped to other chromosomes in the reciprocal genome; (III) Chromosome 10 models not mapped to any chromosome in the reciprocal genome; (IV) Models of other chromosomes in the reciprocal genome mapped to chromosome 10.
